# MAGOH is correlated with poor prognosis and is essential for cell proliferation in lower-grade glioma

**DOI:** 10.18632/aging.204823

**Published:** 2023-06-30

**Authors:** Feng Xiao, Zhenli Long, Yun Guo, Hong Zhu, Zhe Zhang, Yao Xiao, Guowen Hu, Qing Yang, Kai Huang, Hua Guo

**Affiliations:** 1 Department of Neurosurgery, The Second Affiliated Hospital of Nanchang University, Nanchang, China; 2 Jiangxi Key Laboratory of Neurological Tumors and Cerebrovascular Diseases, Nanchang, China; 3 Jiangxi Health Commission Key Laboratory of Neurological Medicine, Nanchang, China; 4 Institute of Neuroscience, Nanchang University, Nanchang, China; 5 Queen Marry College, School of Medicine, Nanchang University, Nanchang, China; 6 Department of Respiratory Medicine, The Second Affiliated Hospital of Nanchang University, Nanchang, China

**Keywords:** MAGOH, lower-grade glioma, prognosis, immunotherapy, chemotherapy, cell proliferation

## Abstract

Objective: Mago-nashi homolog (MAGOH) has been shown to play a pivotal part in various tumors. However, its specific contribution in lower-grade glioma (LGG) is still unknown.

Methods: Pan-cancer analysis was implemented to inspect the expression characteristics and prognostic significance of MAGOH in multiple tumors. The associations between MAGOH expression patterns and the pathological features of LGG were analyzed, as were the connections between MAGOH expression and the clinical traits, prognosis, biological activities, immune features, genomic variations, and responses to treatment in LGG. Additionally, *in vitro* studies were performed to detect the expression levels and biomedical functions of MAGOH in LGG.

Results: Abnormally increased levels of MAGOH expression were connected with adverse prognosis in patients with several types of tumors, including LGG. Importantly, we found that levels of MAGOH expression were independent prognostic biomarker of patients with LGG. Increased MAGOH expression was also highly associated with several immune-related markers, immune cell infiltration, immune checkpoint genes (ICPGs), gene mutations, and responses to chemotherapy in patients with LGG. *In vitro* studies ascertained that abnormally increased MAGOH was essential for cell proliferation in LGG.

Conclusion: MAGOH is a valid predictive biomarker in LGG and may become a novel therapeutic target in these patients.

## INTRODUCTION

Glioma is the most common brain tumor [1]. The World Health Organization (WHO) classification system establishes the standard categorization of gliomas into grades ranging from I to IV [2] Notably, grade II and grade III gliomas are regarded as LGG by The Cancer Genome Atlas (TCGA). LGGs constitute about 20% of primary brain tumors, with these tumors being especially predominant during the fourth decade of life. Treatment options include surgery, chemotherapy, and radiotherapy, with treatment decisions being especially influenced by molecular markers. Identification of biomarkers useful in evaluating the consequences of interventions and in guiding therapeutic decisions is therefore important [3].

Mago-nashi homolog (MAGOH) is a protein that forms part of the exon junction complex (EJC), which also consists of the proteins EIF4A3 and RBM8A. MAGOH plays essential roles in EJC functions, such as mRNA splicing, export and translation. MAGOH also contributes to embryonic development and cellular functioning [4, 5]. It has been reported that MAGOH was closely connected with the tumorigenesis of several tumors [6–8], although its specific functions in LGG remain unclear. The functional activities of MAGOH in patients with LGG were therefore evaluated by bioinformatics analyses and *in vitro* experiments.

The prognostic significance of MAGOH in LGG was analyzed in the TCGA and Chinese Glioma Genome Atlas (CGGA) cohorts. LGG samples were categorized into high-MAGOH and low-MAGOH expression subsets according to the median expression of MAGOH. Survival analysis demonstrated that the high-MAGOH subgroup was interrelated with poorer prognosis than the low-MAGOH subgroup. Cox regression analyses were exploited to inspect the associations of MAGOH expression level with gender, age, WHO grade, isocitrate dehydrogenase (*IDH*) level, 1p/19q status, and O6-methylguanine-DNA methyltransferase (MGMT) status, thus allowing determination of the underlying prognostic significance of MAGOH expression in LGG on the grounds of these clinically relevant biomarkers.

Functional enrichment analyses were implemented to ascertain the potential functions of MAGOH in LGG. The correlations of MAGOH expression with immunological characteristics; genomic alterations; and responses to chemotherapy, were also evaluated. *In vitro* studies were implemented to confirm the levels of expression and biological functions of MAGOH in LGG. Taken together, these findings showed that MAGOH is independently predictive of outcomes in patients with LGG and may be a valuable therapeutic target in these patients.

## RESULTS

### Pan-cancer analysis of MAGOH

**Figure 1:**
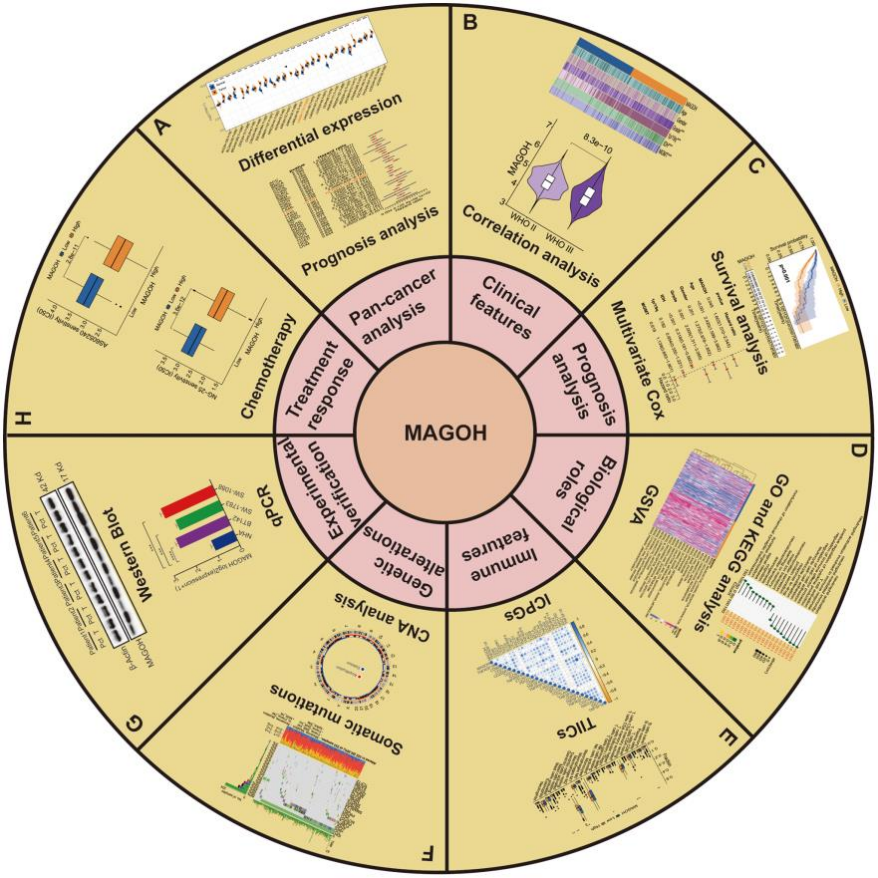
**Flow diagram of this research.** (**A**) Pan-cancer analysis. (**B**) Clinical features. (**C**) Prognosis analysis. (**D**) Biological roles. (**E**) Immune features. (**F**) Genetic alterations. (**G**) Experimental verification. (**H**) Treatment response of MAGOH in LGG.

**Figure 2:**
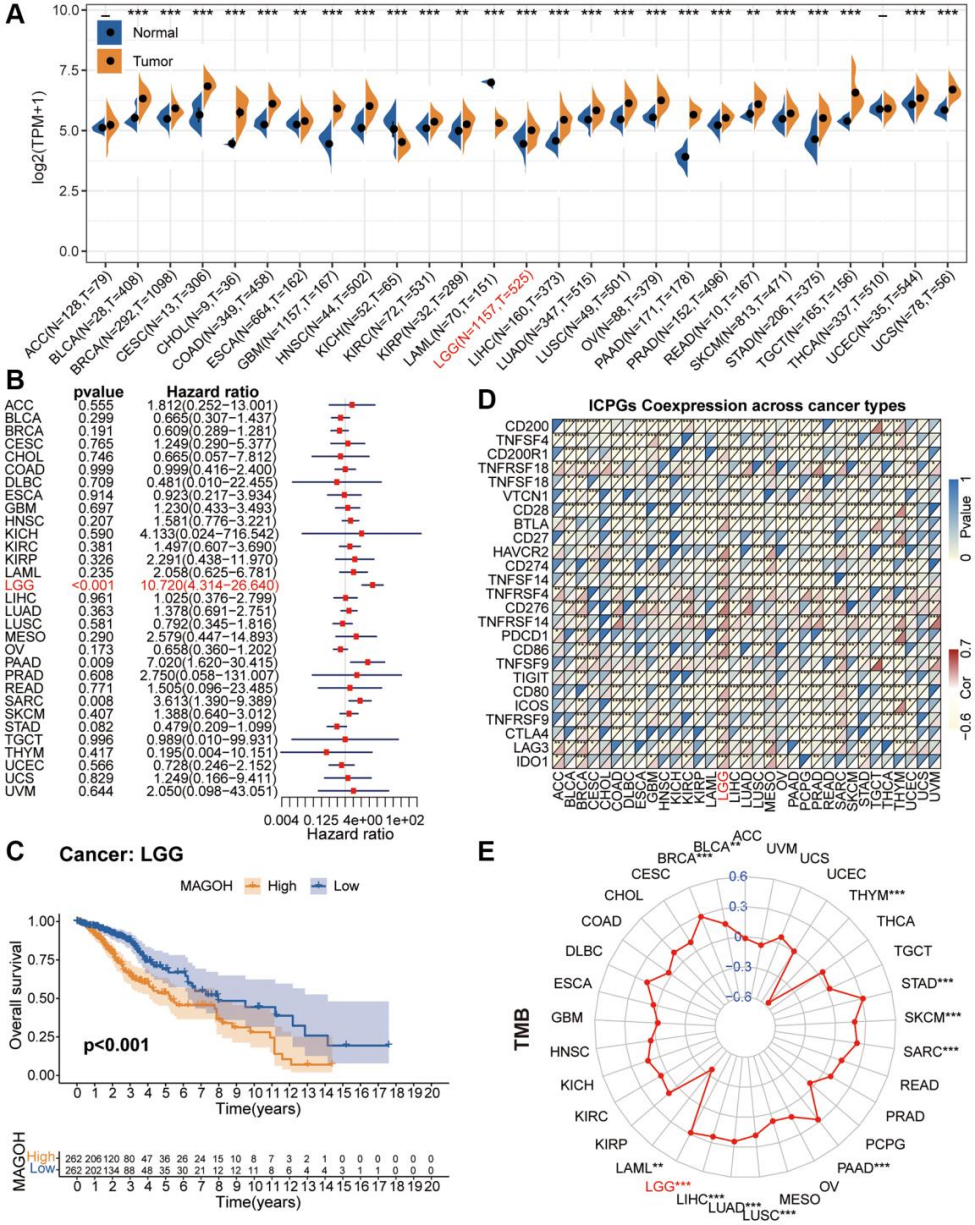
**Pan-cancer analysis of MAGOH.** (**A**) Differential expression of MAGOH in normal and cancer tissues. (**B**) Univariate Cox regression analysis of MAGOH expression in numerous tumors. (**C**) Kaplan-Meier analysis of MAGOH in pan-LGG. (**D**) Co-expression of MAGOH and ICPGs in various cancers. (**E**) Differential TMB in multiple cancers. ^*^P < 0.05, ^**^P < 0.01, ^***^P < 0.001.

The study flow chart is shown in Figure 1. Analysis of pan-cancer gene expression data indicated that MAGOH was prodigiously expressed in several types of cancer. MAGOH expression was markedly increased in 19 kinds of cancer, including BLCA, BRCA, CESC, CHOL, COAD, GBM, HNSC, KIRC, LGG, LIHC, LUAD, LUSC, OV, PAAD, PRAD, SKCM, STAD, UCEC and UCS; and mildly increased in three others, ESCA, KIRP, and READ (Figure 2A). In contrast, MAGOH expression was markedly decreased in KICH and LAML.

We performed the univariate Cox regression analysis to detect the connection between MAGOH expression and overall survival (OS) in 33 kinds of tumors. Forest plots displayed that high MAGOH expression was inversely connected with OS in LGG, PAAD and SARC (Figure 2B). Additionally, survival analysis verified that elevated MAGOH expression was interrelated with inferior survival in LGG patients (Figure 2C).

Evaluation of the associations between MAGOH expression and the expression of ICPGs in 33 types of tumors showed that MAGOH was closely connected with the levels of most ICPGs, in several kinds of tumors, including BRCA, COAD, DLBC, ESCA, HNSC, LGG, LIHC, LUAD, LUSC, MESO, PCPG, PRAD, READ, SARC, SKCM, STAD, TGCT, THCA, THYM, and UCEC (Figure 2D). Evaluation of the connection between MAGOH expression and tumor mutation burden (TMB) in these 33 cancer types showed that MAGOH expression correlated positively with TMB in BLCA, BRCA, LGG, LIHC, LUAD, LUSC, PAAD, SARC, and STAD, whereas MAGOH expression correlated negatively with TMB in LAML and THYM (Figure 2E).

### Connection between MAGOH and clinicopathologic features in LGG

**Figure 3:**
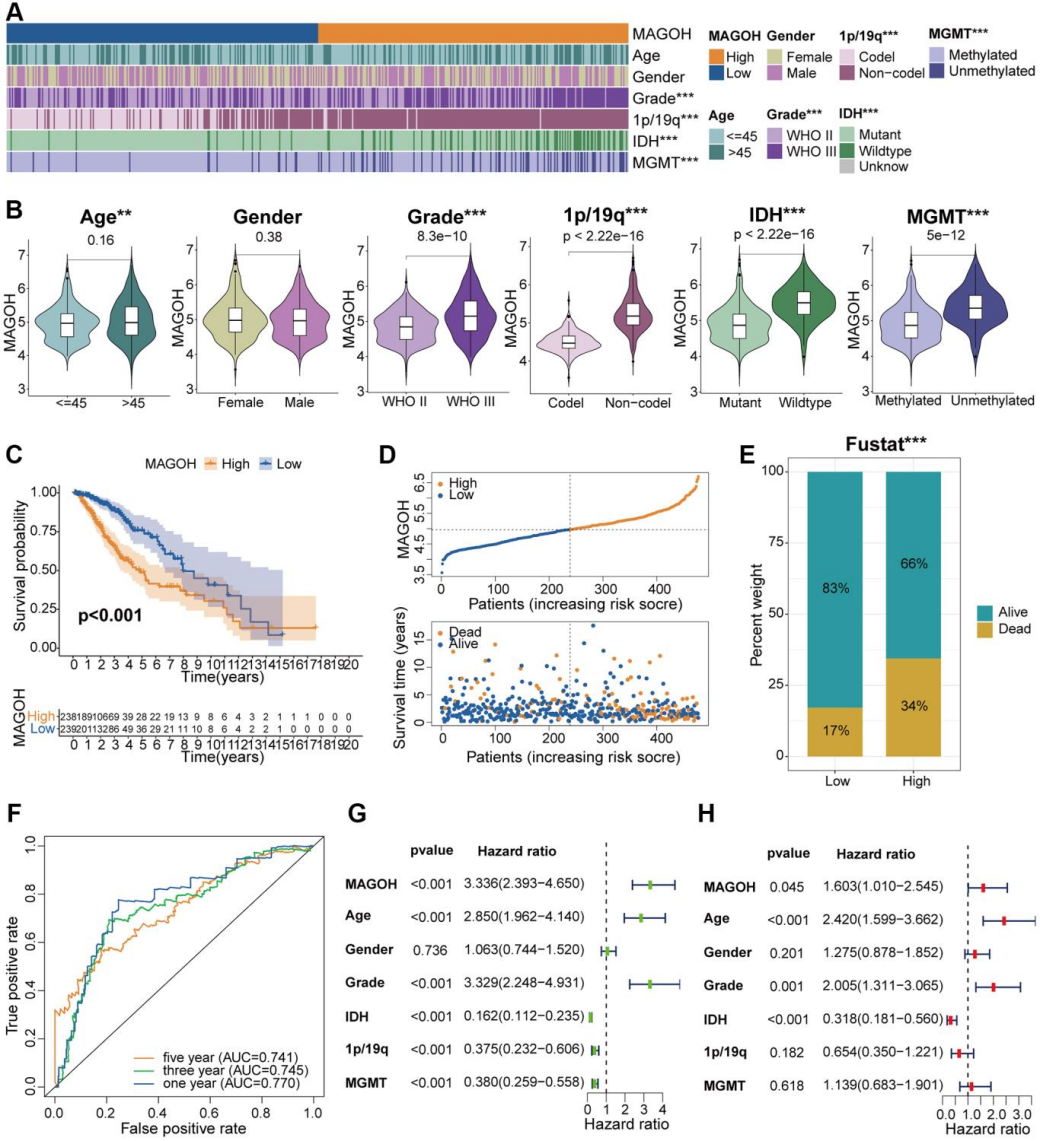
**Clinical correlation analysis of MAGOH expression in LGG samples in the TCGA database.** (**A**) Correlations between MAGOH expression and clinical features of LGG. (**B**) Analysis of MAGOH expression as a function of distinct clinical traits, including gender, age, tumor grade, 1p/19q, *IDH* status, and MGMT status. (**C**) Kaplan-Meier analysis of OS in LGG patients with high and low MAGOH expression. (**D**) Distribution of risk score, OS, and OS status in the high and low MAGOH subgroups. (**E**) Detailed survival analysis of LGG patients in the high and low MAGOH subgroups. (**F**) ROC curves of the association between risk scores and survival in the high and low MAGOH subgroups. (**G**, **H**) Univariate and multivariate Cox analyses of the associations of MAGOH expression and clinical characteristics with OS. ^*^P < 0.05, ^**^P < 0.01, ^***^P < 0.001.

LGG patients were split into high-MAGOH and low-MAGOH subsets on the grounds of the median MAGOH expression, and the correlations between MAGOH expression and clinical properties were examined in the TCGA and CGGA datasets. High MAGOH expression level correlated with older age, *IDH* wildtype, 1p/19q non-codel, and MGMT non-methylation in both the TCGA (Figure 3A, 3B) and CGGA (Supplementary Figure 1A, 1B) datasets. These findings indicated that MAGOH expression was associated with the clinicopathologic traits of LGG patients.

### Increased MAGOH mRNA expression connects with poor prognosis of LGG

The Kaplan-Meier analysis was implemented to investigate the differential OS prognosis between the two subtypes in LGG patients. The results illustrated that the OS of the high-MAGOH subset was evidently shorter than low-MAGOH subset in the TCGA (Figure 3C) and CGGA (Supplementary Figure 1C) cohorts. Up-regulated MAGOH expression interrelated with higher risk score and poorer OS status in LGG patients in the TCGA (Figure 3D) and CGGA (Supplementary Figure 1D) datasets. Detailed survival status of LGG samples was also analyzed in the TCGA (Figure 3E) and CGGA (Supplementary Figure 1E) cohorts. Additionally, receiver operating characteristics (ROC) curve analysis of the correlations between MAGOH expression and OS in LGG samples showed that the areas under the curves (AUCs) for 1-, 3-, and 5-year OS were 0.770, 0.745, and 0.741, respectively, in the TCGA dataset (Figure 3F) and 0.826, 0.872, and 0.851, respectively, in the CGGA dataset (Supplementary Figure 1F). These findings indicated that MAGOH may be an accurate prognostic factor of OS in LGG patients.

### Independent prognostic role of MAGOH in LGG

Univariate and multivariate Cox regression analyses were implemented to determine whether MAGOH was an independent predictive biomarker of OS in the two cohorts. Multivariate analysis showed that MAGOH expression, age, WHO grade, and *IDH* status were independently prognostic of OS in the TCGA cohort (Figure 3G, 3H). In addition, MAGOH expression was independently prognostic of OS in LGG patients in the CGGA dataset (Supplementary Figure 1G, 1H). These findings further indicated that MAGOH expression was independently predictive of OS in LGG patients.

### Functional annotations of MAGOH

**Figure 4:**
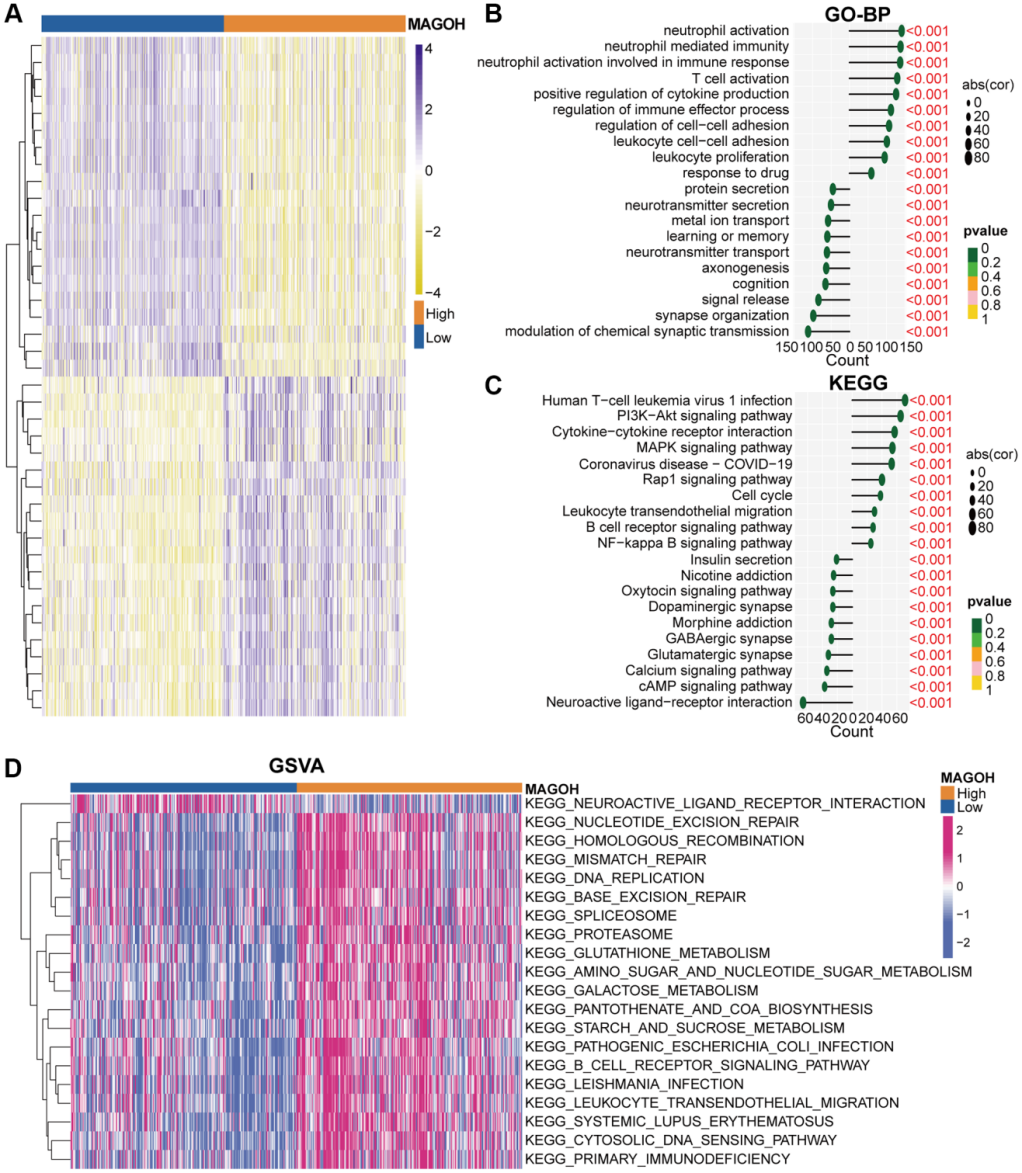
**Biological functions of MAGOH in LGG samples in the TCGA database**. (**A**) DEGs in groups of LGG patients with low and high MAGOH expression. (**B**–**D**) GO-BP (**B**), KEGG (**C**), and (**D**) GSVA analyses of MAGOH in LGG patients.

The value of MAGOH in the differential prognosis of OS in LGG patients was assessed by analyzing differentially-expressed genes (DEGs) in LGG patients dichotomized by mean MAGOH expression, with |log2 (fold change) | >0.5 and P < 0.05 considered significant. In total, 1203 down-regulated (Supplementary Table 1) and 2360 up-regulated (Supplementary Table 2) DEGs were screened in the TCGA dataset, and 1192 down-regulated (Supplementary Table 3) and 2575 up-regulated (Supplementary Table 4) DEGs were screened in the CGGA dataset. The heatmaps show apparent DEGs in the TCGA (Figure 4A) and CGGA (Supplementary Figure 2A) cohorts. These down- and up-regulated DEGs were subsequently utilized to execute Gene Ontology biological process (GO-BP) and Kyoto Encyclopedia of Genes and Genomes (KEGG) analyses. Interestingly, the GO-BP results of down-regulated DEGs in the TCGA dataset intimated that reduced expression of MAGOH correlated markedly with the modulation of chemical synaptic transmission, synapse organization, and cognition; whereas up-regulated DEGs were primarily enriched in genes associated with neutrophil activation, T cell activation, and response to drug (Figure 4B). Analogical outcomes were ascertained in the CGGA cohort (Supplementary Figure 2B). KEGG analysis in the TCGA (Figure 4C) and CGGA (Supplementary Figure 2C) datasets showed that down-regulated DEGs included those involved in neuroactive ligand-receptor reaction, and the cAMP signaling pathway; whereas up-regulated DEGs included those involved in the cell cycle, leukocyte transendothelial migration, PI3K-Akt and MAPK signaling pathways, and B cell receptor signaling pathway.

Additionally, we implemented gene set variation analysis (GSVA) to further identify the underlying molecular mechanisms differing in the high-MAGOH and low-MAGOH subgroups of patients with LGG. The high-MAGOH subtype was chiefly associated with DNA replication, leukocyte transendothelial migration, and the B cell receptor signaling pathway in the TCGA (Figure 4D) and CGGA (Supplementary Figure 2D) datasets. These results elaborated that MAGOH was strongly connected with immune regulation in LGG.

### Interrelation between MAGOH expression and immune features

**Figure 5:**
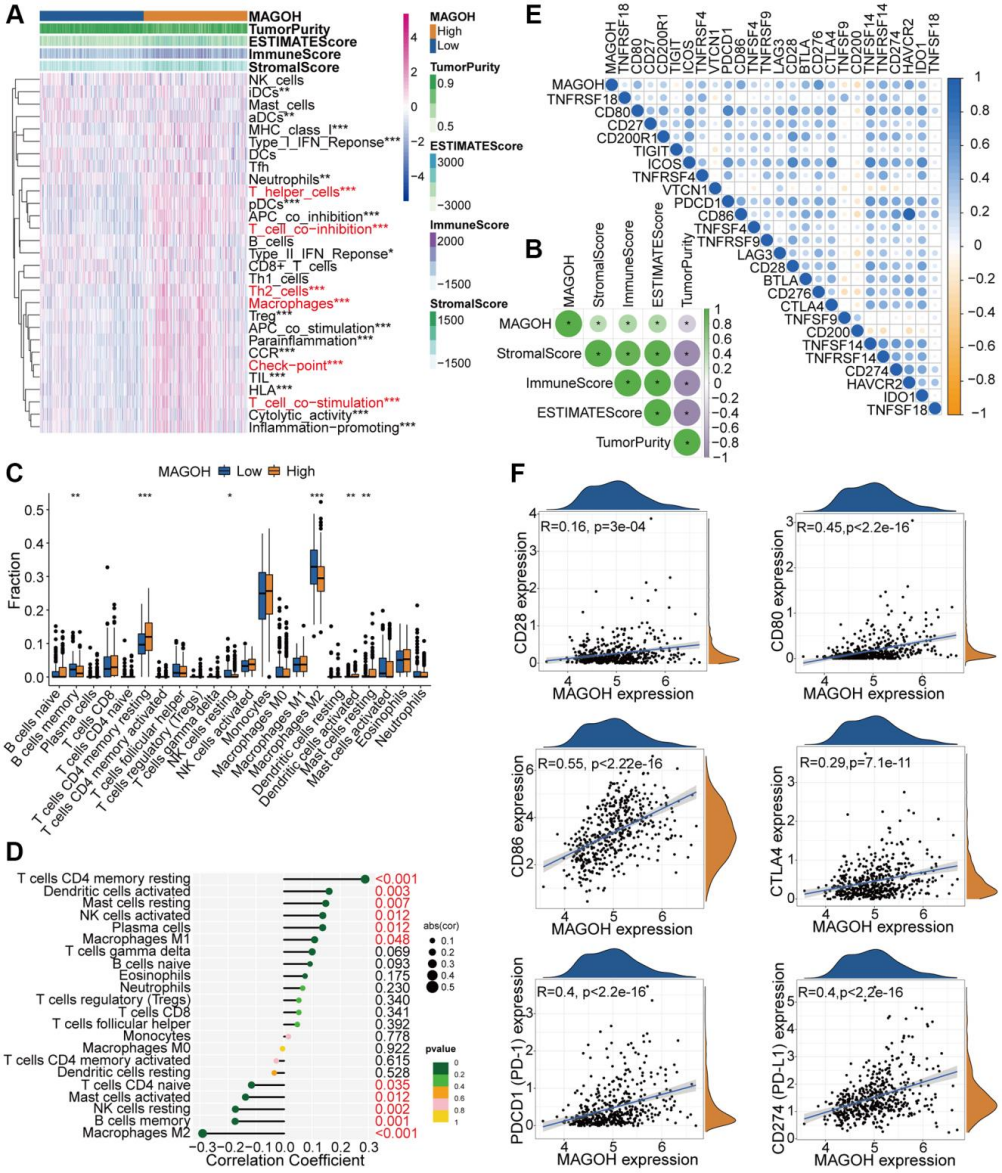
**Distinct TME and immunological features of LGG samples in the low and high MAGOH subgroups in the TCGA database.** (**A**, **B**) Associations between MAGOH expression and 29 immune-interrelated signatures, as determined by ESTIMATE, immune, stromal, and tumor purity scores. (**C**) Comparisons of the infiltration of 22 types of immune cells into LGG tumors with low and high MAGOH expression. (**D**) Lollipop plots showing the associations between MAGOH expression and TIICs. (**E**, **F**) Analysis of the co-expression of MAGOH and 25 ICPGs in LGG samples. ^*^P < 0.05, ^**^P < 0.01, ^***^P < 0.001.

The associations between MAGOH expression and immune cell infiltration were inspected by exploiting the single-sample GSEA (ssGSEA) algorithm, which quantitates 29 immune-related factors. Most of the immune-associated signatures were lower in the low-MAGOH than in the high-MAGOH subtype in the TCGA (Figure 5A) and CGGA (Supplementary Figure 3A) datasets. MAGOH expression was positively associated with ESTIMATE, stromal and immune scores, but inversely associated with tumor purity in the TCGA (Figure 5B) and CGGA (Supplementary Figure 3B) datasets. Estimation of the infiltration of tumor-infiltrating immune cells (TIICs) in the two subgroups using the CIBERSORT algorithm disclosed that MAGOH expression was positively connected with the infiltration of resting memory CD4^+^ T cells and activated dendritic cells, but negatively interrelated with infiltration of M2 macrophages, resting NK cells, and memory B cells in the TCGA cohort (Figure 5C, 5D). Analogical outcomes were detected in the CGGA dataset (Supplementary Figure 3C, 3D).

Differential correlation analyses were performed to further check the differences in expression of ICPGs and MAGOH in LGG patients. MAGOH expression was found to correlate positively with most ICPGs in the TCGA dataset (Figure 5E). Details of the interrelation between MAGOH and several prominent ICPGs (including CD28, CD80, CD86, PD1, PD-L1, and CTLA4) were determined by connection analysis in TCGA cohort (Figure 5F). Analogical outcomes were obtained in CGGA dataset (Supplementary Figure 3E, 3F). These findings provide further evidence for the association of MAGOH with the immune microenvironment.

### Association between MAGOH expression and genomic variations in LGG

**Figure 6:**
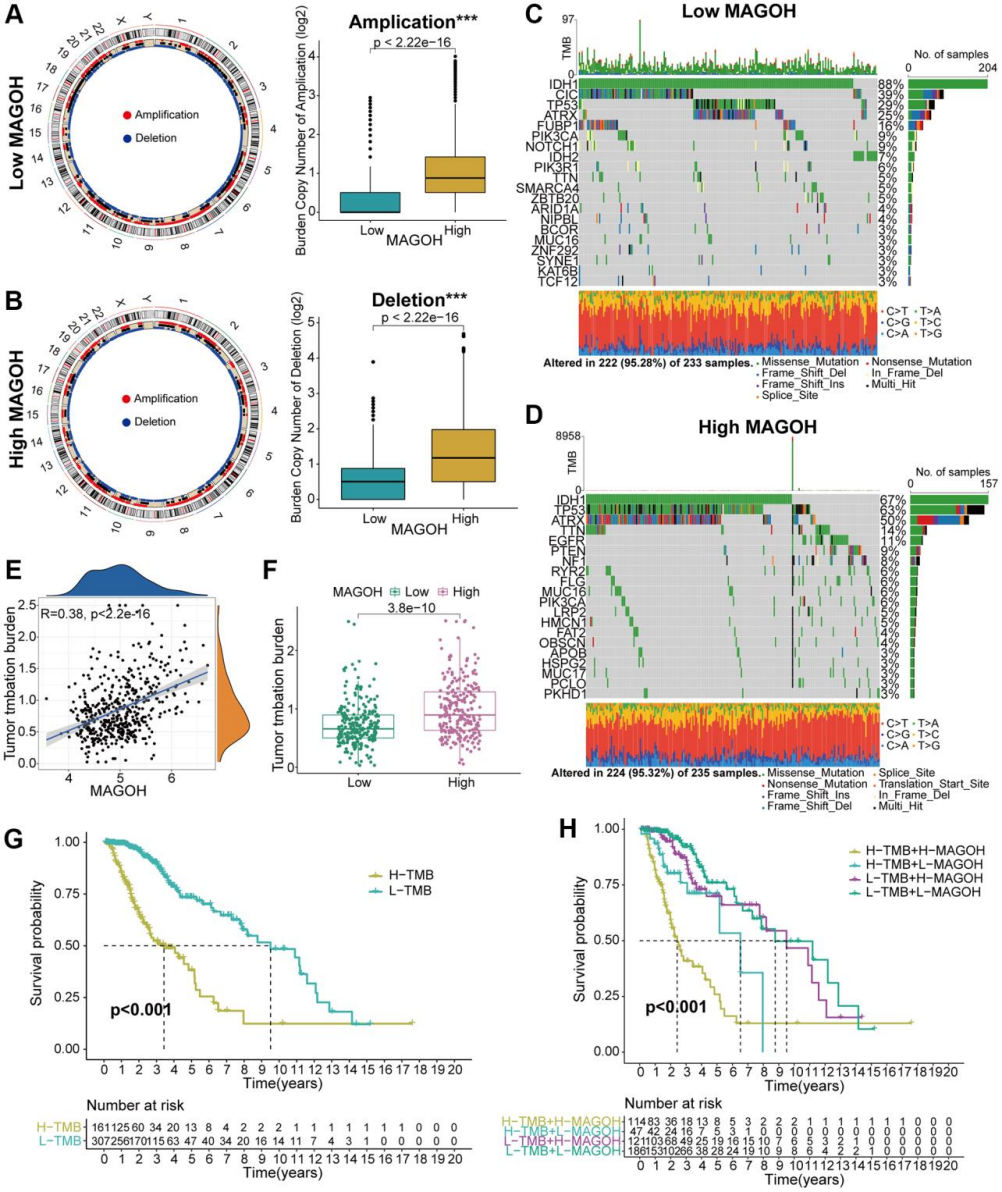
**Comparison of genomic mutations in LGG samples from the low and high MAGOH subgroups in the TCGA dataset.** (**A**, **B**) Circos plots of low and high MAGOH subtypes of LGG, showing the amplifications and deletions of chromosomes, with boxplots showing that copy number amplifications and deletions were lower in the low than in the high MAGOH subgroup. (**C**, **D**) Waterfall plots showing mutated genes in the low (**C**) and high (**D**) MAGOH subgroups. (**E**, **F**) Association of MAGOH expression and TMB levels in patients with LGG. (**G**, **H**) Association between TMB level and patient prognosis (**G**) and the differential prognostic value of TMB level in the low and high MAGOH subtypes of patients with LGG (**H**). ^*^P < 0.05, ^**^P < 0.01, ^***^P < 0.001.

Genomic variations may play an essential part in adjusting immune cell infiltration and tumor immunity [9, 10]. Copy number alteration (CNA) and somatic mutation analyses were therefore performed to differentiate genomic mutations in the high-MAGOH and low-MAGOH expression subsets. The frequency of CNAs, both amplifications and deletions, was lower in groups with low than high MAGOH expression (Figure 6A, 6B). The “waterfall” plot of somatic variations was developed to display that specific mutated genes were present in the two subtypes. For example, the variation frequencies of *IDH1* and *CIC* were higher in the low-MAGOH than in the high-MAGOH subtype, whereas the variation frequencies of *TP53* and *ATRX* were similar in these two groups (Figure 6C, 6D). In addition, MAGOH expression was found to correlate positively with TMB level in LGG patients (Figure 6E, 6F). Evaluation of the association of differential OS with MAGOH expression level in patients with low and high TMB indicated that the integration of higher MAGOH expression and higher TMB level was connected with poorer OS in patients with LGG (Figure 6G, 6H), suggesting that LGG patients with high MAGOH expression might present with specific immune traits.

### *In vitro* experiments of MAGOH in LGG

**Figure 7:**
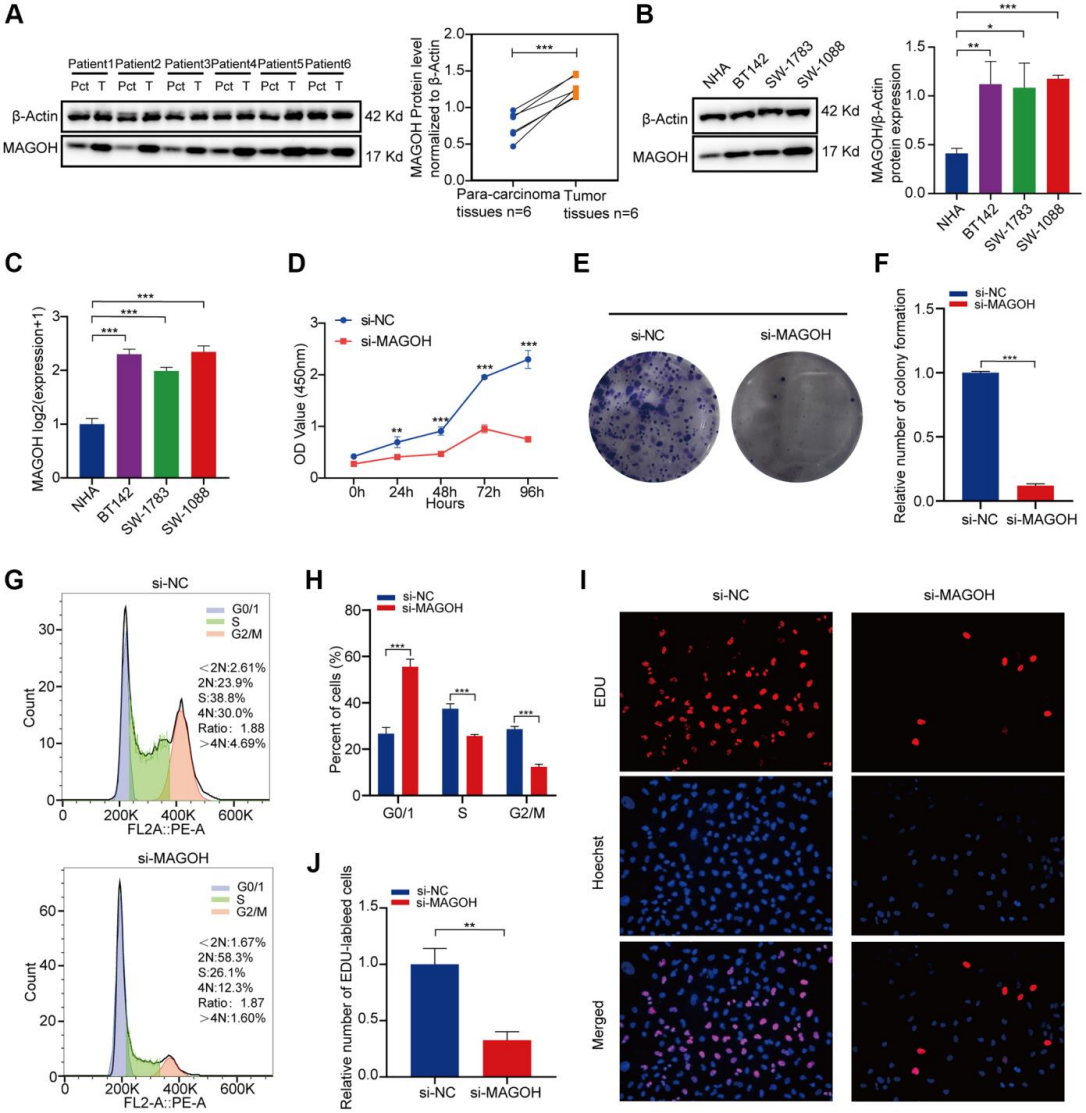
***In vitro* confirmation of the expression of MAGOH in LGG.** (**A**) Western blot analysis of MAGOH expression in LGG tissues and corresponding para-carcinoma tissues. (**B**) Western blot and (**C**) qRT-PCR analysis of MAGOH expression in NHA and LGG cell lines. (**D**) Viability of SW1088 cells transfected with si-MAGOH or si-NC, as determined by CCK-8 assays. (**E**, **F**) Effect of MAGOH knockdown on colony formation by SW1088 cells. (**G**, **H**) Cell cycle distributions of SW1088 cells transfected with lentiviruses encoding si-MAGOH or si-NC. (**I**, **J**) Representative images (**I**) and histogram analysis (**J**) of EdU assays after silencing MAGOH in SW1088 cells. ^*^P < 0.05, ^**^P < 0.01, ^***^P < 0.001.

The levels of MAGOH protein were discovered to be higher in LGG tissues than in tumor-adjacent tissues, with the results quantified by ImageJ software (Figure 7A). Analysis of MAGOH mRNA and protein levels in LGG cell lines, such as SW-1088, SW-1783 and BT142 cells, and in an NHA cell line illustrated that MAGOH expression was distinctly higher in the LGG cell lines when compared to the NHA line (Figure 7B, 7C).

Correlations between MAGOH expression and LGG cells were also assessed *in vitro*. CCK-8 (Figure 7D) and colony formation (Figure 7E, 7F) assays showed that transfection of si-MAGOH into SW1088 cells reduced their proliferative capacity when compared with transfection of si-NC. Downregulation of MAGOH expression in SW1088 cells also affected the cell cycle, reducing the numbers of cells in S and G2/M phases were decreased and increasing the number in G0/G1 phases (Figure 7G, 7H). Moreover, EdU assays showed that silencing MAGOH expression in SW1088 cells inhibited their proliferation (Figure 7I, 7J), further indicating that MAGOH may contribute to cell proliferation in LGG.

### Associations between MAGOH expression in LGG and responses to treatment

**Figure 8:**
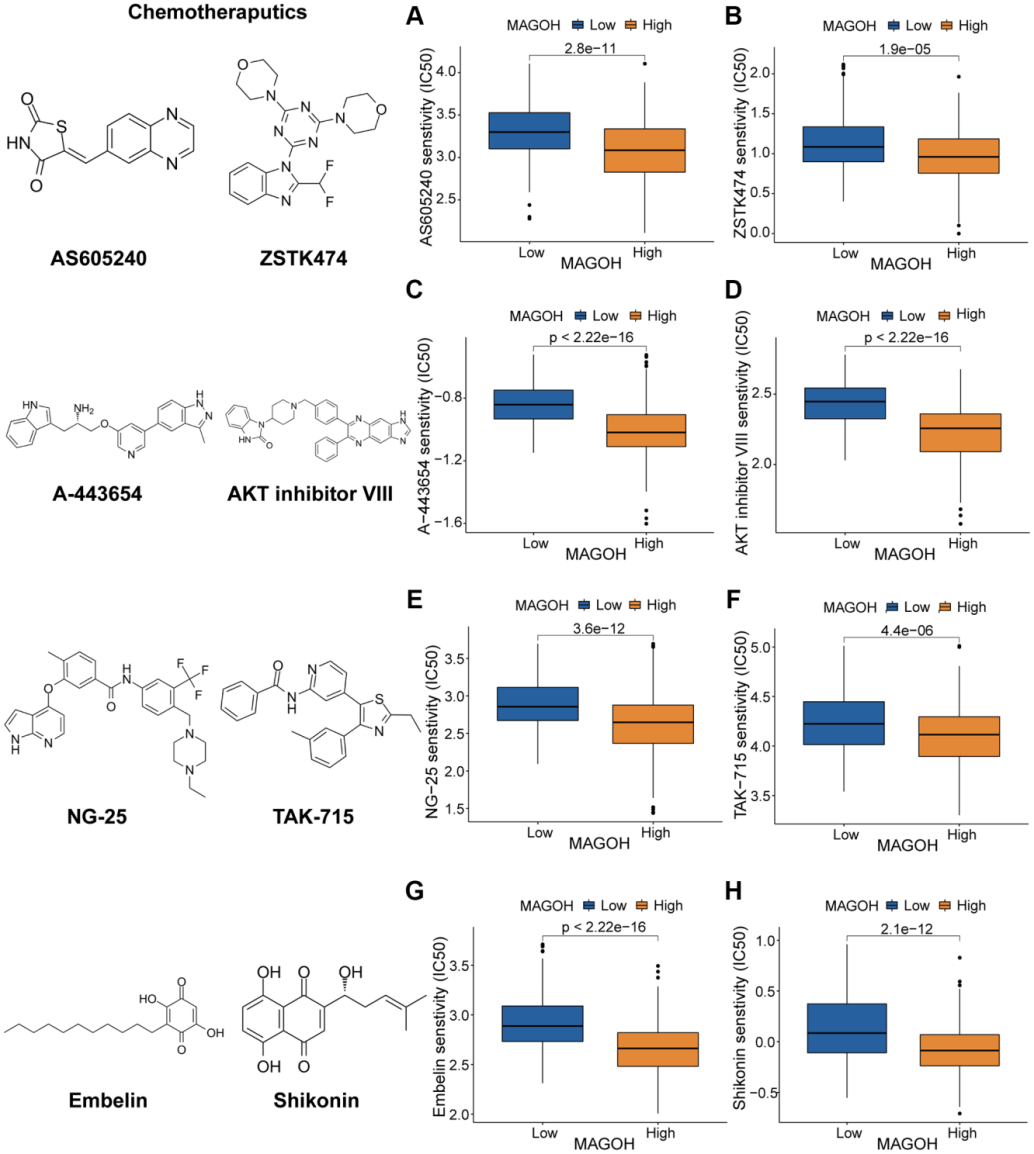
**Prediction of response to chemotherapeutics drugs in LGG subtypes.** (**A**–**D**) Effects of the PI3K/AKT inhibitors AS605240 (**A**), ZSTK474 (**B**), A-443654 (**C**), and AKT inhibitor VIII (**D**). (**E**, **F**) Effects of the MAPK inhibitors NG-25 (**E**) and TAK-715 (**F**). (**G**, **H**) Effects of the NF kappaB inhibitors embelin (**G**) and shikonin (**H**).

To guide treatment of patients with LGG based on levels of MAGOH expression, the correlations between MAGOH expression and anticarcinogens were evaluated. High MAGOH expression was associated with lower inhibitory centration (IC50) of the PI3K/AKT inhibitors AS605240 (Figure 8A), ZSTK474 (Figure 8B), A-443654 (Figure 8C), and AKT inhibitor VIII (Figure 8D); the MAPK inhibitors NG-25 (Figure 8E) and TAK-715 (Figure 8F); and the NF kappaB inhibitors Embelin (Figure 8G) and Shikonin (Figure 8H). These findings illustrated that patients with high MAGOH expression were more sensitive to treatment with these anticarcinogens, suggesting that these agents may contribute to future chemotherapy regimens for LGG patients with high MAGOH expression.

## DISCUSSION

Despite the general effectiveness of cancer treatment regimens, such as surgery, radiotherapy, and chemotherapy, LGG is still characterized by a poor prognosis [11–13]. These traditional treatments have limited effects in patients with LGG, suggesting the need to identify new prognostic factors and methods of treatment. MAGOH is a protein that has been linked to cell proliferation and the cell cycle. High MAGOH expression has been interrelated with the malignant progression of multiple tumors, including gastric cancer, melanoma, and breast cancer [14–16], but its role in patients with LGG remains undiscovered. The present study therefore comprehensively inspected the associations of MAGOH expression with the clinical features, prognosis, biological activities, tumor immunity, gene variations, and treatment responses in patients with LGG.

Pan-cancer analysis of MAGOH expression verified that higher MAGOH expression was interrelated with shorter OS, higher expression of ICPGs, and a greater TMB burden in pan-LGG. Survival analysis of LGG samples in the TCGA cohort showed that prognosis was poorer in the high than in the low MAGOH subtype, with Kaplan-Meier analysis showing that up-regulation MAGOH expression was significantly connected with reduced OS. ROC analysis also found that MAGOH expression was interrelated with OS in LGG patients. MAGOH expression was also connected with clinical features in LGG patients, and multivariate Cox regression analyses found that MAGOH was independently prognostic of survival in LGG patients. Similar results were ascertained in the CGGA dataset.

GO-BP and KEGG analyses of up-regulated DEGs in the TCGA and CGGA cohorts elucidated that MAGOH expression was connected with increased expression of DEGs associated with immunoregulation, cell cycle, and PI3K-Akt signaling pathway. GSVA analysis also found that high-MAGOH expression correlated with immune responses and cancer-connected signaling pathways.

The ssGSEA algorithm was utilized to detect differences in immune-connected signatures between the two isoforms in the TCGA and CGGA datasets. Furthermore, the ESTIMATE and CIBERSORT algorithms were also utilized to identify the compositions of TME and TIICs in the two MAGOH subgroups. MAGOH expression was discovered to be connected with immune cell infiltration into LGGs. Because the novel immunotherapy agent ICB has shown curative activity in the treatment of variant tumor types [17, 18], the connection between MAGOH expression and ICPGs expression was evaluated in LGG patients. MAGOH expression was discovered to correlate positively with the expression of some common ICPGs, including CD28, CD80, CD86, PD1, PD-L1, and CTLA4, in the TCGA and CGGA datasets. Moreover, the somatic mutation and CNA analyses ascertained that the high-MAGOH expression subtype possessed higher TBM and CNA burden than the low-MAGOH expression subtype.

Although TMZ-targeting chemotherapy is the most common method of treating patients with glioma, its efficacy is limited. Analyses of their sensitivity to chemotherapeutic agents showed that the high-MAGOH expression subgroup was more sensitive to several of these agents, including AS605240, ZSTK474, A-443654, AKT inhibitor VIII, NG-25, TAK-715, Embelin, and Shikoin, than the low-MAGOH expression subgroup. These findings suggest that MAGOH expression level may predict the chemosensitivity of patients with LGG.

**Figure 9:**
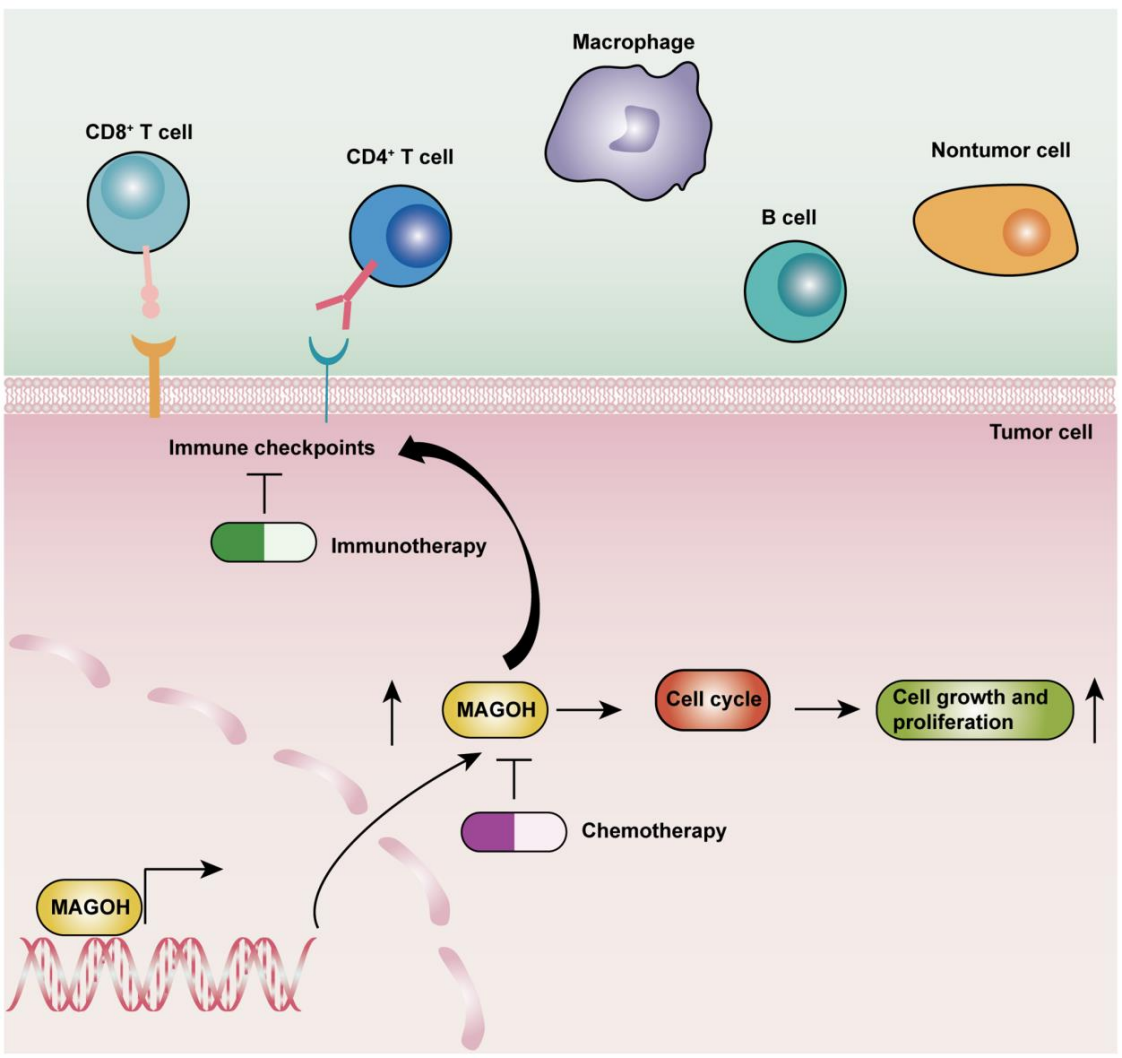
Schematic diagram illustrating the underlying biological functions of MAGOH in LGG.

To assess whether MAGOH was linked to cell proliferation and the cell cycle in LGG patients, cell lines expressing MAGOH were transfected *in vitro* with a specific siRNA to inhibit MAGOH expression. The underlying biological functions of MAGOH are summarized schematically in Figure 9.

This study had several limitations. Additional independent LGG datasets should be evaluated to ascertain the prognostic significance of MAGOH in LGG. Additional experiments should be implemented to examine the molecular mechanism of MAGOH in LGG.

## CONCLUSION

This study indicated that MAGOH was a prognostic factor that was related to cell proliferation in LGG. MAGOH may be a vital therapeutic target in patients with LGG.

## METHODS

### Data collection and collation

MAGOH expression, survival, clinicopathological, and TMB data in 33 kinds of tumors were collected from the TCGA database and MAGOH expression data in normal tissue was attained from the Genotype-Tissue Expression (GTEx) database.

The mRNA expression, survival, and clinicopathological data of two independent cohorts of LGG patients were obtained from the TCGA and CGGA (CGGA_325) databases. The mRNA expression data in the fragments per kilobase million format were transformed to transcripts per kilobase million values. Then, these values were log2 transformed for easier comparisons. Data on genomic mutations of LGG samples were achieved from the TCGA database.

### Inclusion criteria for samples

LGG patients were included if they had (1) WHO grade information; (2) gene expression data; (3) OS > 30 days. In total, 477 (Supplementary Table 5) and 170 (Supplementary Table 6) LGG patients were screened out from TCGA and CGGA databases, respectively. Pan-cancer analysis of MAGOH expression included LGG patients with OS <30 days to guarantee the consistency of survival information in the 33 tumors.

### Prognostic role of MAGOH and corroboration

LGG samples were categorized into high-MAGOH and low-MAGOH subsets in accordance with the median MAGOH concentrations in the two cohorts. The Kaplan-Meier analysis was employed to inspect the OS of LGG patients in the two subtypes. Additionally, the ability of MAGOH expression to estimate the OS of LGG patients in the two datasets were analyzed by determining the survival state ratio, drawing ROC curves, and calculating AUCs. Univariate and multivariate Cox regression analyses were exploited to examine whether MAGOH expression was an independent biomarker of LGG patients.

### Functional annotations

DEGs in high-MAGOH and low-MAGOH expression isoforms were ascertained by exploiting the R package limma, with |log2 FC| > 0.5 and a false-discovery rate (FDR) < 0.05 regarded as statistically significant [19]. DEGs were employed to GO-BP and KEGG enrichment analyses by implementing the R package clusterProfiler [20]. Highly enriched molecular pathways in the two subtypes were assessed by GSVA [21]. The correlations of the most highly enriched molecular pathways in the two subtypes with the results of KEGG analysis (c2.cp.kegg.v7.2.symbols) were analyzed, with |log2 FC| > 0.1, *p* < 0.05, and FDR < 0.05 regarded as significant.

### Immunological characteristics of LGG

The differential abundance of 29 previously determined immune-associated biomarkers [22, 23], in the two subgroups were inspected by implementing the ssGSEA algorithm. In agreement with expression levels in LGG patients, the ESTIMATE algorithm was exploited to examine the abundance of immune cells, stromal cells, and tumor purity [24]. Four categories of scores, including tumor purity, ESTIMATE score (representing nontumor composites), immune score (representing the abundance of immune cells) and stromal score (representing the abundance of stromal cells) were determined. The penetration profile of TIICs on the basis of the gene expression data in LGG patients was ascertained using the CIBERSORT algorithm [25]. Additionally, the correlations of MAGOH expression with the expression of 25 previously identified ICPGs [26] were analyzed.

### Genomic mutation analysis

Conspicuous deletions and amplifications in the whole genome that differed in high-MAGOH and low-MAGOH expression subtypes were inspected using the RCircos tool [27]. Mutation categories and prevalence of genes differing in the two subtypes were assessed using Maftools and GenVisR [28, 29]. The associations between MAGOH expression and TMB level in 33 kinds of tumors were assessed by conducting the R package fmsb, whereas the connection between MAGOH expression and TMB level in LGG patients in the TCGA dataset were assessed by implementing the R package ggplot2.

### Evaluation of MAGOH expression and treatment response

Differences between high-MAGOH and low-MAGOH expression subgroups in sensitivity to several chemotherapeutic agents, including PI3K/AKT inhibitors (AS605240, ZSTK474, A-443654, and AKT inhibitor VIII), MAPK inhibitors (NG-25 and TAK-715), and NF-kappa B inhibitors (embelin and shikonin), were analyzed using the R package pRRophetic [30].

### Cell culture and transfection

Three LGG lines, SW1088, BT142, and SW1783, were gathered from the American Type Culture Collection. Normal human astrocyte (NHA) cell line was obtained from the Culture Collection of the Chinese Academy of Sciences (Shanghai, China). SW-1783 and SW-1088 cells were cultured in Leibovitz’s L-15 medium supplemented with 10% fetal bovine serum (Gibco), whereas BT142 and NHA cells were cultured in Dulbecco’s modified Eagle’s medium/F12 medium, at 37°C and 5% CO2. SW1088 cells were transfected with lentiviral vector containing a MAGOH-specific shRNA (5′-GCAGAGAAGGGTGCACCTAAC-3′) or negative control (NC) vector alone at a multiplicity of infection (MOIs) of 10. Cells were treated with polybrene to assess transfection efficiency and with puromycin to pick out positive cells.

### Western blot analysis

LGG and tumor-adjacent tissue samples (*n* = 6 each) were acquired from the Second Affiliated Hospital of Nanchang University. Brain tissues were isolated, and cells lysed in radioimmunoprecipitation assay buffer (Solarbio, China) mixed with proteinase inhibitors. Lysates were separated by 10% SDS–PAGE and proteins transferred onto polyvinylidene fluoride membranes. Subsequently, primary antibodies MAGOH (1:1000, 12347-1-AP, Proteintech, China) and β-actin (1:10,000; 66009-1-lg, Proteintech, China) were added and incubated at 4°C. Then, the membranes were incubated with the relevant secondary antibodies. Ultimately, the proteins were visualized on a GV6000M imaging system (GelView 6000pro).

### Quantitative real-time PCR (qRT-PCR)

Total RNA was isolated from cells using Simply P Total RNA Extraction Kits (Bioflux, China) and reverse-transcribed to complementary DNA with HiScript III-RT SuperMix (Vazyme, China). PCR reactions were performed using specific primer sequences for MAGOH (forward, 5′-GGCAGGAGCTTGAAATCGTC-3′, reverse, 5′-GCCTTCTGGATCCTTGGATTG-3′) and β-actin (forward, 5′-TGACGTGGACATCCGCAAAG-3′; reverse, 5′-CTGGAAGGTGGACAGCGAGG-3′). MAGOH expression was normalized to β-actin expression by utilizing the 2^−ΔΔCT^ method.

### CCK-8 assay

Transfected SW1088 cells (2 × 10^3^ peer well) were plated in 96-well plates. The cell proliferation was estimated by calculating the numbers of cells at 0, 24, 48, 72, and 96h using Cell Counting Kit 8 assays (Glpbio, GK10001) according to the manufacturer’s protocol.

### Colony formation assay

Transfected SW1088 cells (2 × 10^3^ peer well) were seeded in 6-well plates and incubated for 14 days. Afterwards, 0.1% crystal violet stain solution was utilized to stain the cells, and the number of colonies was calculated by ImageJ.

### Cell cycle analysis

Transfected SW1088 cells were fixed with 70% ethanol at 4°C overnight. Then, the cells were stained by exploiting RNase A containing propidium iodide (Suzhou, China), and the distribution was examined by implementing flow cytometry.

### EdU assay

Transfected SW1088 cells (2 × 10^4^ peer well) were plated in 24-well plates and incubated for 72 h. The cells were subsequently cultured with EdU reagent for 2 h; fixed in 4% paraformaldehyde, 0.5% Triton X-100; and subjected to Hoechst staining. The EdU incorporation rate was quantified using ImageJ software.

### Statistical analysis

The Kaplan-Meier analysis was exploited to discriminate the prognosis between high-MAGOH and low-MAGOH subgroups by utilizing a two-sided log-rank test. The capacity of MAGOH expression to assess prognosis was analyzed by determining the AUC of ROC curves. The independent predictive significance of MAGOH was assessed by Cox regression analyses. Immune-associated factors, such as the 29 immune-associated signatures, TIICs, 25 ICPGs, TMB, and CNA burden, were compared in the two subtypes by Student’s *t* tests. Correlations between variables were estimated by Pearson’s or Spearman’s correlation test. Differences in sensitivity to anticancer drugs in the two subtypes were determined by Wilcoxon signed-rank tests. All statistical analyses were conducted by exploiting R programming version 4.1.0, SPSS Statistics, and GraphPad Prism 8 (GraphPad Software), with *p*-values < 0.05 defined as statistically significant.

### Availability of data and materials

The data analyzed in this research can be acquired in the TCGA (https://portal.gdc.cancer.gov/) and CGGA (http://www.cgga.org.cn/) databases.

## Supplementary Materials

Supplementary Figures

Supplementary Table 1

Supplementary Table 2

Supplementary Table 3

Supplementary Table 4

Supplementary Tables 5 and 6
