# Supplementary material for: MAGOH is correlated with poor prognosis and is essential for cell proliferation in lower-grade glioma
Source: Aging (Albany NY). 2023 Jun 30;15(12):5713–33. doi: 10.18632/aging.204823 (PMC10333088; doi:10.18632/aging.204823)
Supplement: Supplementary Figures [file aging-15-204823-s001.pdf]

SUPPLEMENTARY FIGURES

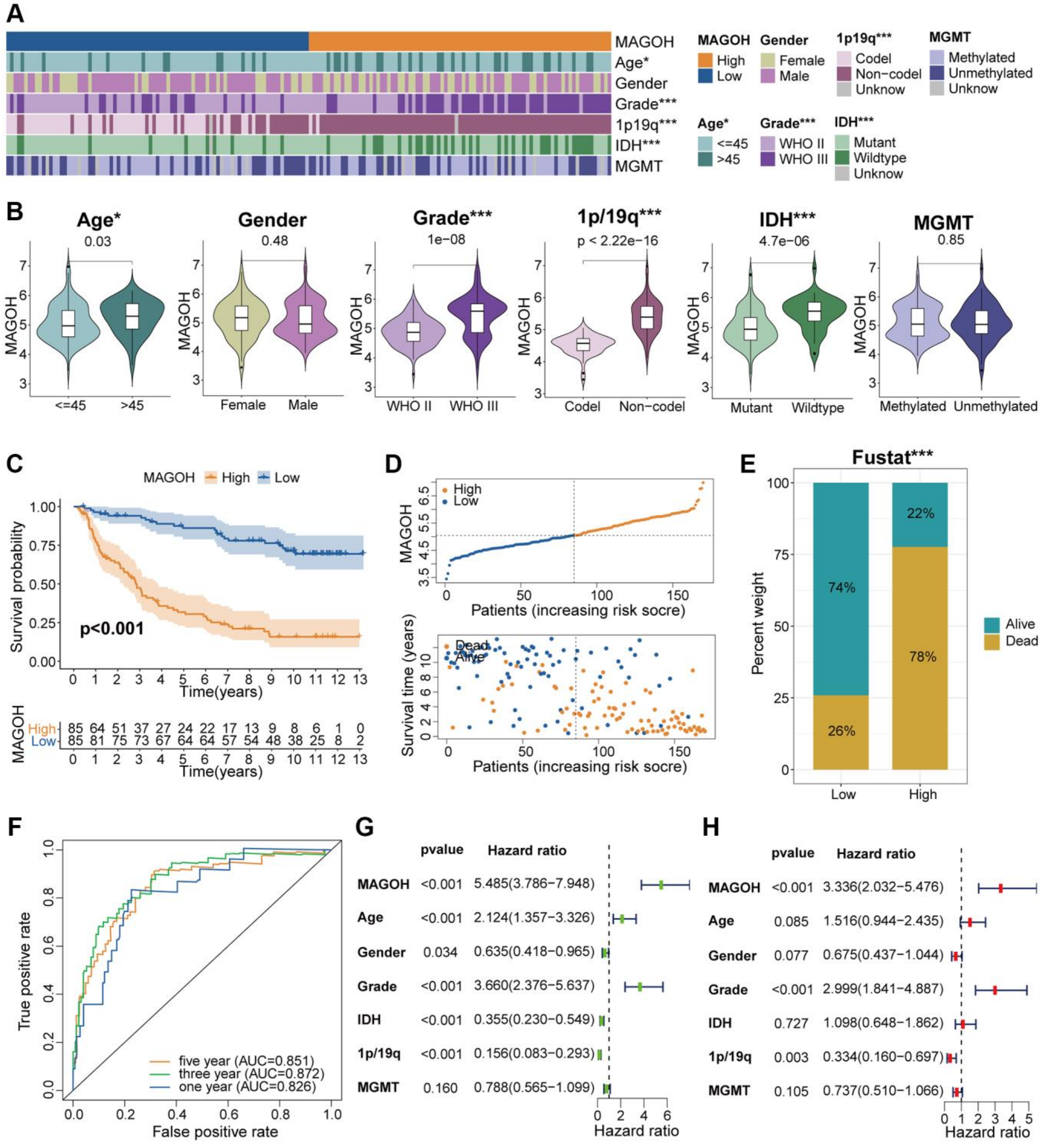

Supplementary Figure 1. Clinical correlation analysis of MAGOH in CGGA. (**A**) Connection between MAGOH expression and clinical features of LGG in CGGA. (**B**) Variance analysis of MAGOH expression in distinct clinical traits (including age, gender, grade, and 1p/19q, *IDH*, and MGMT statuses) in CGGA. (**C**) Prognostic analysis of high-MAGOH and low-MAGOH subgroups in CGGA. (**D**) Distribution of risk score, OS, and OS status of high-MAGOH and low-MAGOH subtypes in CGGA. (**E**) Distinct proportions of the living situation between the two subgroups. (**F**) ROC curves reflecting the predictive role of the risk score in CGGA. (**G**, **H**) Univariate and multivariate Cox analyses of MAGOH expression and clinical characteristics in CGGA. $^{*}P < 0.05$, $^{**}P < 0.01$, $^{***}P < 0.001$.

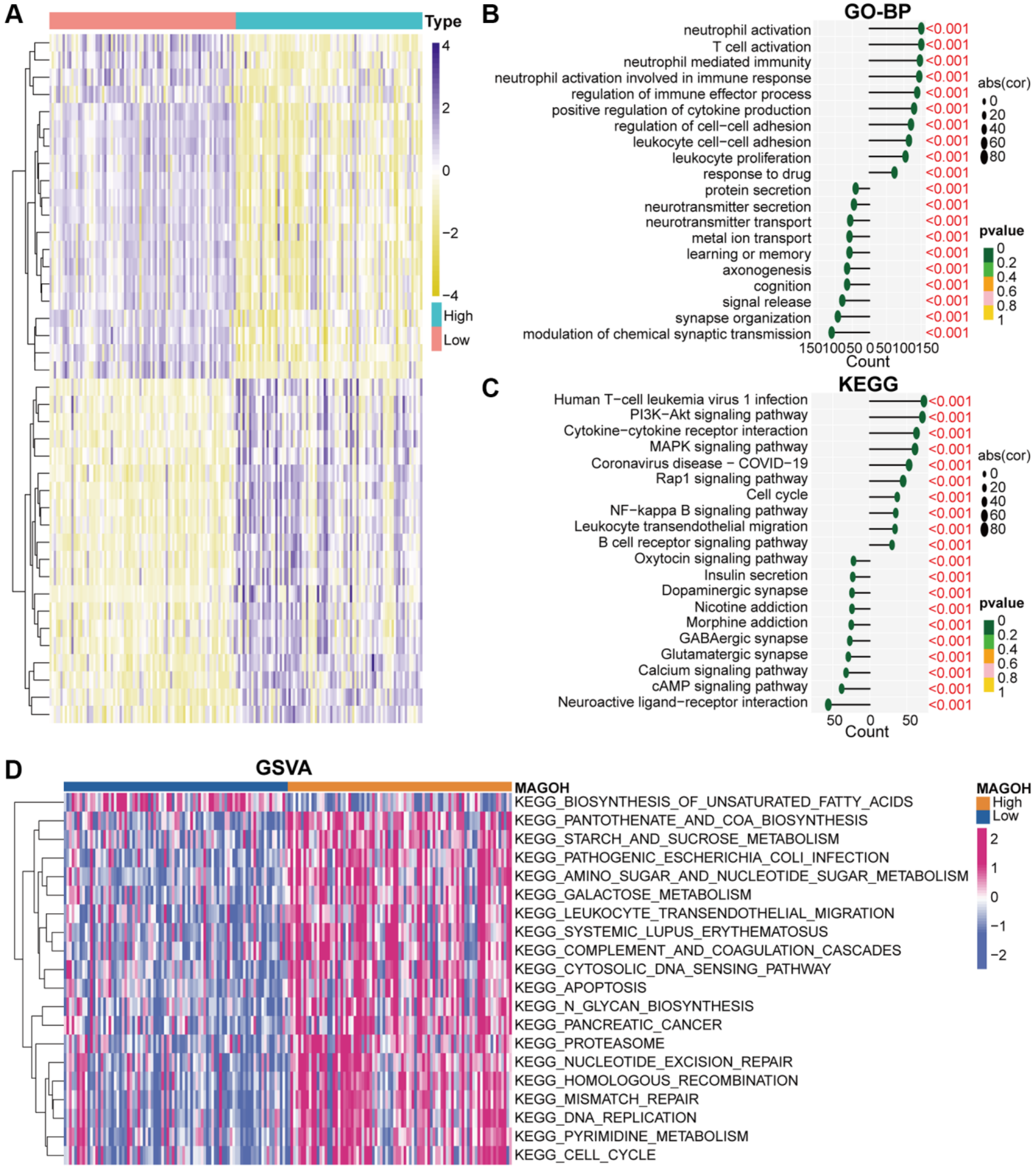

Supplementary Figure 2. Biological functions of MAGOH in LGG in CGGA. (**A**) DEGs between the low-MAGOH and high-MAGOH expression LGG subtypes. (**B**, **C**) The GO-BP (**B**) and KEGG (**C**) analyses for MAGOH in LGG patients in CGGA. (**D**) GSVA analysis in CGGA.

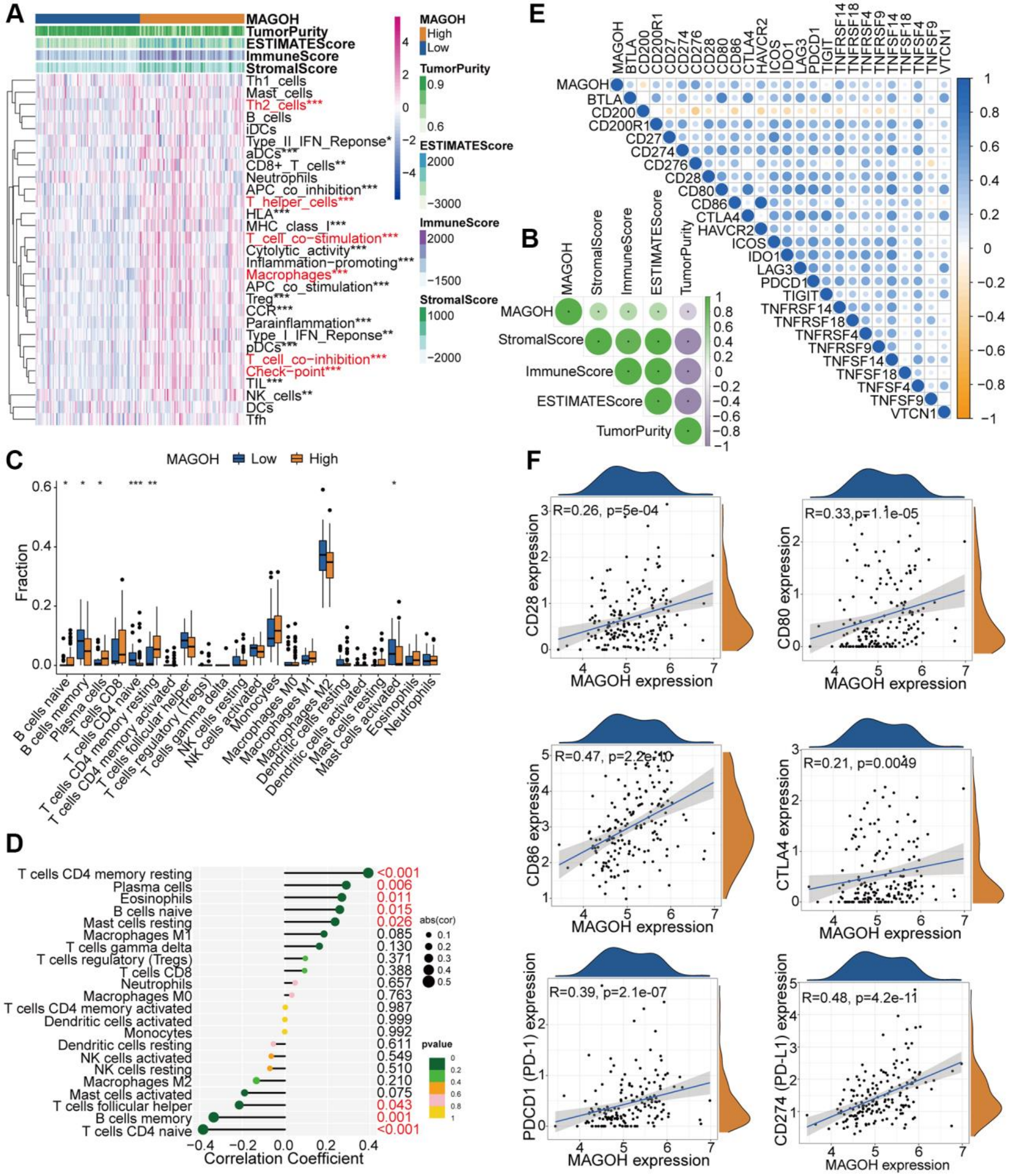

Supplementary Figure 3. Distinct TME and immunological features of the low-MAGOH and high-MAGOH subgroups in CGGA. (**A**, **B**) Connection between MAGOH expression and 29 immune-interrelated signatures, ESTIMATE, immune, stromal scores, and tumor purity. (**C**) Comparisons of infiltration of 22 types of immune cells in the two subtypes. (**D**) Lollipop plots displayed the association between MAGOH expression and TIICs. (**E**, **F**) Co-expression analysis of MAGOH and 25 ICPGs. $^{*}P$ < 0.05, $^{**}P$ < 0.01, $^{***}P$ < 0.001.
