# Supplementary material for: MAGOH is correlated with poor prognosis and is essential for cell proliferation in lower-grade glioma
Source: Aging (Albany NY). 2023 Jun 30;15(12):5713–33. doi: 10.18632/aging.204823 (PMC10333088; doi:10.18632/aging.204823)
Supplement: Supplementary Table 1 [file aging-15-204823-s002.docx]

**Supplementary Table 1. Down-regulated DEGs in TCGA cohort.**

| **id** | **logFC** | **AveExpr** | **t** | **P.Value** | **adj.P.Val** | **B** |
| --- | --- | --- | --- | --- | --- | --- |
| TRIM67 | -2.17221252 | 2.869696061 | -13.12244949 | 8.59E-34 | 9.05E-32 | 65.9489 |
| L1CAM | -2.150749422 | 4.246966352 | -13.22046403 | 3.32E-34 | 3.66E-32 | 66.8914 |
| GABRG2 | -2.109715385 | 3.597075921 | -13.38469003 | 6.69E-35 | 7.86E-33 | 68.4779 |
| CPLX2 | -2.032849085 | 5.510399153 | -12.06668843 | 1.95E-29 | 1.23E-27 | 56.0152 |
| INA | -1.990206079 | 5.606853012 | -12.8831897 | 8.64E-33 | 7.80E-31 | 63.6619 |
| AL022313.4 | -1.989432677 | 3.945487335 | -13.66635166 | 4.20E-36 | 5.59E-34 | 71.2191 |
| SYT4 | -1.943899847 | 3.556194341 | -12.37060044 | 1.13E-30 | 8.12E-29 | 58.8317 |
| SVOP | -1.918463379 | 3.244808796 | -13.77093606 | 1.50E-36 | 2.10E-34 | 72.2432 |
| CACNG2 | -1.8657077 | 2.731106153 | -15.05358249 | 3.64E-42 | 8.95E-40 | 85.0552 |
| CHGB | -1.825638284 | 5.868204758 | -13.97731549 | 1.93E-37 | 2.97E-35 | 74.2739 |
| GRIN1 | -1.818179074 | 4.335483441 | -10.63108655 | 7.91E-24 | 2.76E-22 | 43.2437 |
| KCNIP2 | -1.815578287 | 5.166370751 | -15.15184907 | 1.33E-42 | 3.46E-40 | 86.0542 |
| SNAP25 | -1.794692041 | 7.091278332 | -10.37231729 | 7.32E-23 | 2.31E-21 | 41.0445 |
| AC062021.1 | -1.792000571 | 3.592076509 | -9.398438671 | 2.33E-19 | 5.20E-18 | 33.0845 |
| LHX5-AS1 | -1.784247526 | 2.293097299 | -10.26392249 | 1.84E-22 | 5.55E-21 | 40.1334 |
| CHGA | -1.76497962 | 5.19790331 | -10.89898046 | 7.63E-25 | 2.96E-23 | 45.5553 |
| SNCB | -1.762250401 | 5.719118017 | -10.54462617 | 1.67E-23 | 5.62E-22 | 42.5052 |
| NSG2 | -1.741142618 | 6.766044236 | -12.08538858 | 1.64E-29 | 1.04E-27 | 56.1874 |
| VSTM2A | -1.730652835 | 3.376335731 | -12.56625762 | 1.78E-31 | 1.39E-29 | 60.6639 |
| TMEM151B | -1.708462684 | 4.357000028 | -14.83120845 | 3.53E-41 | 7.84E-39 | 82.8031 |
| SCRT1 | -1.69829956 | 3.902986333 | -12.47390282 | 4.28E-31 | 3.22E-29 | 59.7973 |
| MARCHF4 | -1.693685888 | 2.683854107 | -12.54119536 | 2.26E-31 | 1.75E-29 | 60.4284 |
| SCN3B | -1.680252564 | 4.452063458 | -14.2539986 | 1.21E-38 | 2.16E-36 | 77.0158 |
| GRIN3A | -1.663554853 | 2.210002757 | -15.15416447 | 1.30E-42 | 3.39E-40 | 86.0778 |
| MYT1L | -1.6494211 | 2.378502775 | -12.88507203 | 8.48E-33 | 7.71E-31 | 63.6799 |
| VSNL1 | -1.625366077 | 4.581628101 | -7.619881596 | 1.38E-13 | 1.67E-12 | 20.0018 |
| CAMK2A | -1.613885319 | 4.698479171 | -7.702805767 | 7.79E-14 | 9.72E-13 | 20.5659 |
| HRH3 | -1.606096425 | 3.029020419 | -12.69732 | 5.12E-32 | 4.16E-30 | 61.8993 |
| FAM163B | -1.60292061 | 4.506695582 | -10.02848061 | 1.34E-21 | 3.70E-20 | 38.1756 |
| CHRM1 | -1.599290412 | 3.404036117 | -11.96881622 | 4.84E-29 | 2.92E-27 | 55.1159 |
| SHISAL1 | -1.58360409 | 3.323926103 | -12.55313429 | 2.02E-31 | 1.57E-29 | 60.5406 |
| AL049749.1 | -1.579895282 | 2.568528434 | -12.82331177 | 1.53E-32 | 1.33E-30 | 63.0928 |
| ACTL6B | -1.579621055 | 4.58083732 | -11.88731005 | 1.03E-28 | 6.00E-27 | 54.3701 |
| KCNK3 | -1.578550275 | 2.987469268 | -13.63085251 | 5.97E-36 | 7.84E-34 | 70.8722 |
| PTPRN | -1.570434663 | 4.302290036 | -10.5604167 | 1.46E-23 | 4.93E-22 | 42.6398 |
| CALN1 | -1.548957269 | 3.665768664 | -11.61560912 | 1.24E-27 | 6.43E-26 | 51.9039 |
| CDK5R2 | -1.545094777 | 4.292845676 | -11.63204024 | 1.07E-27 | 5.59E-26 | 52.0522 |
| SLC7A14 | -1.537274373 | 3.494934913 | -15.47459967 | 4.79E-44 | 1.47E-41 | 89.3508 |
| PRLHR | -1.534963038 | 2.761246648 | -9.553282149 | 6.68E-20 | 1.57E-18 | 34.3151 |
| ABCC8 | -1.534550333 | 3.75967261 | -13.14642821 | 6.80E-34 | 7.19E-32 | 66.1792 |
| SLC17A7 | -1.533279678 | 4.603526008 | -6.941039621 | 1.28E-11 | 1.23E-10 | 15.5675 |
| SYT13 | -1.533031436 | 3.281219035 | -9.732046006 | 1.56E-20 | 3.90E-19 | 35.7528 |
| TMEM130 | -1.530993942 | 4.296659823 | -9.178311287 | 1.34E-18 | 2.76E-17 | 31.3588 |
| SYN1 | -1.52989366 | 6.005985409 | -11.29271725 | 2.31E-26 | 1.04E-24 | 49.0146 |
| DDN | -1.529303276 | 3.82583132 | -8.711916304 | 4.97E-17 | 8.67E-16 | 27.798 |
| STMN2 | -1.521436148 | 5.948608466 | -7.649504907 | 1.13E-13 | 1.38E-12 | 20.2028 |
| GABRA1 | -1.521148688 | 2.769562864 | -8.934756375 | 8.99E-18 | 1.70E-16 | 29.4828 |
| SYT1 | -1.504588934 | 4.895077532 | -8.081322615 | 5.33E-15 | 7.51E-14 | 23.2 |
| MMD2 | -1.503907515 | 5.065492461 | -12.47047526 | 4.42E-31 | 3.31E-29 | 59.7652 |
| IQSEC3 | -1.502184497 | 3.084101111 | -11.53194432 | 2.66E-27 | 1.33E-25 | 51.1508 |
| KCNJ11 | -1.502037089 | 4.173143017 | -12.70335982 | 4.83E-32 | 3.94E-30 | 61.9564 |
| ST8SIA3 | -1.499525841 | 3.410750608 | -12.47794498 | 4.12E-31 | 3.10E-29 | 59.8351 |
| PACSIN1 | -1.491284402 | 4.143944094 | -7.533320448 | 2.51E-13 | 2.95E-12 | 19.4182 |
| NEFL | -1.490853837 | 4.277054708 | -6.733175803 | 4.80E-11 | 4.29E-10 | 14.2768 |
| SULT4A1 | -1.47913776 | 4.083633747 | -8.008833598 | 8.98E-15 | 1.23E-13 | 22.6881 |
| RAB3C | -1.477194405 | 3.308332011 | -12.03868367 | 2.53E-29 | 1.57E-27 | 55.7575 |
| AMER3 | -1.470404818 | 2.358925268 | -13.26318162 | 2.19E-34 | 2.47E-32 | 67.3032 |
| ATCAY | -1.453294471 | 6.670190042 | -11.77344701 | 2.93E-28 | 1.63E-26 | 53.3328 |
| PCSK2 | -1.450600234 | 3.429944182 | -10.40029059 | 5.76E-23 | 1.83E-21 | 41.2806 |
| RPH3A | -1.442005144 | 3.583799585 | -9.590388624 | 4.95E-20 | 1.18E-18 | 34.6121 |
| CA10 | -1.441549151 | 4.567347182 | -10.72026859 | 3.64E-24 | 1.32E-22 | 44.0093 |
| NEFM | -1.437829351 | 3.430654172 | -7.328174764 | 1.01E-12 | 1.11E-11 | 18.056 |
| CRTAC1 | -1.436844135 | 5.545226122 | -11.78881732 | 2.55E-28 | 1.43E-26 | 53.4725 |
| CAMK4 | -1.427188661 | 2.342546402 | -13.1994889 | 4.07E-34 | 4.43E-32 | 66.6895 |
| SNAP91 | -1.426273591 | 5.16443939 | -13.40194337 | 5.65E-35 | 6.70E-33 | 68.6451 |
| GABRA3 | -1.411269502 | 4.352965483 | -12.91541158 | 6.34E-33 | 5.92E-31 | 63.9688 |
| NRSN1 | -1.400639528 | 4.627004542 | -12.37098557 | 1.13E-30 | 8.10E-29 | 58.8353 |
| WSCD2 | -1.388450688 | 2.092049369 | -12.66079797 | 7.25E-32 | 5.80E-30 | 61.5544 |
| XKR7 | -1.385244061 | 1.928790317 | -12.3567455 | 1.29E-30 | 9.18E-29 | 58.7025 |
| NRGN | -1.374658767 | 6.868152964 | -6.407751318 | 3.56E-10 | 2.85E-09 | 12.3216 |
| AL355916.2 | -1.371806699 | 3.802927028 | -8.819438641 | 2.19E-17 | 3.96E-16 | 28.6071 |
| GABRB3 | -1.370436785 | 3.94903599 | -13.19811157 | 4.12E-34 | 4.48E-32 | 66.6762 |
| SHC3 | -1.366383889 | 4.740381102 | -10.14450969 | 5.05E-22 | 1.46E-20 | 39.1368 |
| CELF4 | -1.365377977 | 3.514281576 | -10.20535919 | 3.02E-22 | 8.89E-21 | 39.6437 |
| RIMS2 | -1.364755957 | 2.458793939 | -13.97554908 | 1.96E-37 | 3.01E-35 | 74.2565 |
| SLC6A17 | -1.363795691 | 3.300620904 | -8.458603965 | 3.35E-16 | 5.34E-15 | 25.9203 |
| HPCAL4 | -1.361724013 | 5.135069068 | -9.786635239 | 9.94E-21 | 2.54E-19 | 36.1953 |
| SUSD5 | -1.356156306 | 3.890571896 | -9.248401075 | 7.70E-19 | 1.62E-17 | 31.9052 |
| CUX2 | -1.327927129 | 2.724105316 | -11.17555843 | 6.58E-26 | 2.83E-24 | 47.9777 |
| GFRA1 | -1.32681367 | 3.817813104 | -11.23372899 | 3.91E-26 | 1.72E-24 | 48.4918 |
| WNT7B | -1.323013408 | 3.549577932 | -10.51282459 | 2.20E-23 | 7.32E-22 | 42.2344 |
| GPR17 | -1.31967367 | 6.194965222 | -8.16266674 | 2.96E-15 | 4.28E-14 | 23.7787 |
| CACNG3 | -1.318646812 | 2.44127623 | -8.212697301 | 2.06E-15 | 3.01E-14 | 24.1367 |
| HPCA | -1.318142194 | 4.46520031 | -6.998432482 | 8.84E-12 | 8.67E-11 | 15.9295 |
| PVALB | -1.313986829 | 1.929256868 | -8.884238399 | 1.33E-17 | 2.47E-16 | 29.0982 |
| PHYHIP | -1.313542207 | 5.263286166 | -8.65004425 | 7.96E-17 | 1.36E-15 | 27.3356 |
| TNNT1 | -1.306045178 | 2.183191012 | -12.77621831 | 2.41E-32 | 2.03E-30 | 62.646 |
| FAM155A | -1.303996442 | 3.780941149 | -14.20161888 | 2.05E-38 | 3.58E-36 | 76.4951 |
| SEZ6L | -1.303580966 | 6.864836539 | -10.46867102 | 3.21E-23 | 1.05E-21 | 41.8594 |
| RFPL1S | -1.303094374 | 2.475125705 | -11.49217722 | 3.82E-27 | 1.87E-25 | 50.794 |
| LHX5 | -1.301255542 | 1.574881239 | -9.848042816 | 5.99E-21 | 1.56E-19 | 36.6951 |
| KCNC2 | -1.299931 | 2.010976421 | -9.869397827 | 5.02E-21 | 1.32E-19 | 36.8694 |
| SLC4A10 | -1.28928426 | 2.839272577 | -10.09223217 | 7.84E-22 | 2.22E-20 | 38.7028 |
| JPH3 | -1.288839087 | 4.811672671 | -11.26673564 | 2.91E-26 | 1.29E-24 | 48.7841 |
| TMEM132D | -1.288484941 | 1.556768835 | -11.27921644 | 2.60E-26 | 1.16E-24 | 48.8948 |
| SHISA9 | -1.287476444 | 2.680475751 | -10.85929135 | 1.08E-24 | 4.12E-23 | 45.2106 |
| GABRB2 | -1.286513658 | 2.185866718 | -8.760082889 | 3.45E-17 | 6.11E-16 | 28.1596 |
| SEZ6L2 | -1.281041426 | 6.176715404 | -10.7869573 | 2.04E-24 | 7.51E-23 | 44.5844 |
| SSTR2 | -1.28047083 | 3.670205164 | -11.01113928 | 2.84E-25 | 1.15E-23 | 46.5333 |
| VWC2L | -1.277046986 | 1.591237549 | -13.38290064 | 6.80E-35 | 7.98E-33 | 68.4605 |
| GABRD | -1.275798952 | 4.79830766 | -8.903751977 | 1.14E-17 | 2.14E-16 | 29.2466 |
| SLC12A5 | -1.274711877 | 3.235115233 | -8.841897277 | 1.84E-17 | 3.36E-16 | 28.777 |
| ATP8A2 | -1.272775786 | 2.27319848 | -11.32438392 | 1.74E-26 | 7.90E-25 | 49.2959 |
| CELF3 | -1.265905107 | 4.803137883 | -11.01611567 | 2.71E-25 | 1.11E-23 | 46.5769 |
| PSD | -1.265691841 | 5.445245045 | -11.62770774 | 1.11E-27 | 5.81E-26 | 52.013 |
| UNC79 | -1.261420066 | 3.375840568 | -13.30556492 | 1.45E-34 | 1.66E-32 | 67.7124 |
| ZFR2 | -1.261193187 | 1.770225276 | -11.36198065 | 1.24E-26 | 5.72E-25 | 49.6305 |
| SLC8A2 | -1.260434002 | 3.668357868 | -10.29364362 | 1.43E-22 | 4.37E-21 | 40.3826 |
| FRRS1L | -1.259773347 | 3.790901091 | -11.78865633 | 2.55E-28 | 1.43E-26 | 53.471 |
| ASIC4-AS1 | -1.256125927 | 4.207011703 | -8.289460859 | 1.17E-15 | 1.76E-14 | 24.6893 |
| AC122707.1 | -1.254248335 | 2.282884411 | -10.40377604 | 5.60E-23 | 1.78E-21 | 41.3101 |
| NMNAT2 | -1.253600657 | 4.95572331 | -11.82776668 | 1.78E-28 | 1.02E-26 | 53.827 |
| SRRM4 | -1.253528799 | 2.284469244 | -11.47892559 | 4.30E-27 | 2.09E-25 | 50.6752 |
| RTP5 | -1.250315369 | 4.694043332 | -8.659243468 | 7.42E-17 | 1.27E-15 | 27.4042 |
| HMGA1P7 | -1.248447882 | 2.053237378 | -11.65066366 | 9.03E-28 | 4.75E-26 | 52.2203 |
| CPLX1 | -1.241653668 | 5.393202235 | -10.29135326 | 1.46E-22 | 4.44E-21 | 40.3634 |
| TLX1 | -1.240539251 | 1.672566967 | -9.484826549 | 1.16E-19 | 2.66E-18 | 33.7694 |
| RGR | -1.239982061 | 3.183046353 | -9.759072467 | 1.25E-20 | 3.16E-19 | 35.9717 |
| TNR | -1.236350107 | 7.183007305 | -9.685646742 | 2.28E-20 | 5.59E-19 | 35.3779 |
| SYNGR3 | -1.23230408 | 3.590570829 | -8.963717756 | 7.18E-18 | 1.38E-16 | 29.704 |
| CREG2 | -1.228992153 | 2.837636268 | -7.188728064 | 2.55E-12 | 2.68E-11 | 17.1471 |
| BRINP1 | -1.228307485 | 5.027763787 | -10.3378962 | 9.82E-23 | 3.04E-21 | 40.7545 |
| AC012213.4 | -1.223776635 | 2.484026386 | -10.47644243 | 3.00E-23 | 9.83E-22 | 41.9254 |
| CCK | -1.221737894 | 3.519325976 | -6.620185621 | 9.70E-11 | 8.35E-10 | 13.5888 |
| HTR2A | -1.217381448 | 2.301877993 | -12.58786727 | 1.45E-31 | 1.15E-29 | 60.8672 |
| ZDHHC22 | -1.215080594 | 6.542652454 | -11.05633448 | 1.90E-25 | 7.87E-24 | 46.9291 |
| TAFA2 | -1.209819711 | 2.149240418 | -13.04984786 | 1.73E-33 | 1.74E-31 | 65.2528 |
| CAMK1G | -1.20669141 | 2.540346325 | -7.334987424 | 9.63E-13 | 1.06E-11 | 18.1007 |
| PDE2A | -1.206005056 | 4.808438373 | -11.98131948 | 4.31E-29 | 2.63E-27 | 55.2306 |
| PTPN5 | -1.20269112 | 2.966420427 | -8.261855054 | 1.43E-15 | 2.14E-14 | 24.4901 |
| CALY | -1.197643049 | 3.12186726 | -7.615758666 | 1.42E-13 | 1.72E-12 | 19.9739 |
| CHRNB2 | -1.197582348 | 3.786657369 | -11.85845136 | 1.34E-28 | 7.74E-27 | 54.1067 |
| PPP2R2C | -1.194326142 | 4.166479531 | -9.148288828 | 1.70E-18 | 3.47E-17 | 31.1256 |
| CAMKV | -1.193774962 | 4.158688456 | -8.473671862 | 3.00E-16 | 4.81E-15 | 26.0308 |
| AC023421.1 | -1.188561798 | 1.686189499 | -10.24548206 | 2.15E-22 | 6.43E-21 | 39.979 |
| HTR1A | -1.188127395 | 1.407474372 | -11.69331275 | 6.11E-28 | 3.29E-26 | 52.606 |
| HAPLN1 | -1.187664888 | 3.359656827 | -9.282310551 | 5.88E-19 | 1.26E-17 | 32.1706 |
| TMEM271 | -1.18634012 | 3.324900087 | -10.04085847 | 1.21E-21 | 3.35E-20 | 38.2778 |
| CNNM1 | -1.179186391 | 2.004628954 | -12.41694606 | 7.33E-31 | 5.36E-29 | 59.2644 |
| SYN2 | -1.17884263 | 4.598127383 | -7.880400298 | 2.24E-14 | 2.97E-13 | 21.7897 |
| KCNJ9 | -1.17059493 | 5.195120795 | -12.73213248 | 3.67E-32 | 3.04E-30 | 62.2285 |
| SV2B | -1.163184425 | 2.463698717 | -7.299831409 | 1.22E-12 | 1.33E-11 | 17.8701 |
| RGS4 | -1.16291146 | 3.569239757 | -6.410344111 | 3.50E-10 | 2.81E-09 | 12.3369 |
| UNC13A | -1.161833037 | 4.680069193 | -12.10809953 | 1.33E-29 | 8.52E-28 | 56.3968 |
| CDH18 | -1.160337574 | 2.41539023 | -11.06519593 | 1.76E-25 | 7.29E-24 | 47.0068 |
| AC012213.1 | -1.160229358 | 1.783386114 | -12.1326028 | 1.06E-29 | 6.83E-28 | 56.6229 |
| F5 | -1.159930193 | 2.544824571 | -7.13177498 | 3.71E-12 | 3.82E-11 | 16.7799 |
| SPHKAP | -1.15931794 | 3.358694568 | -8.315441397 | 9.68E-16 | 1.47E-14 | 24.8772 |
| SCN2B | -1.158645734 | 3.805081033 | -11.05037988 | 2.00E-25 | 8.27E-24 | 46.8769 |
| PTPRT | -1.157500862 | 3.60754724 | -9.825857724 | 7.19E-21 | 1.87E-19 | 36.5143 |
| ATP2B3 | -1.153945102 | 2.256355776 | -9.180455176 | 1.32E-18 | 2.72E-17 | 31.3755 |
| SHISA6 | -1.153201669 | 3.094165992 | -7.731286898 | 6.39E-14 | 8.06E-13 | 20.7607 |
| DACH2 | -1.150789156 | 1.996090416 | -10.45457176 | 3.62E-23 | 1.18E-21 | 41.7399 |
| CBLN1 | -1.149834929 | 3.104169746 | -10.65594501 | 6.37E-24 | 2.25E-22 | 43.4567 |
| GABBR1 | -1.149287501 | 7.48783659 | -13.90641214 | 3.90E-37 | 5.80E-35 | 73.5748 |
| DGKK | -1.148188886 | 1.360365468 | -11.57685177 | 1.77E-27 | 9.03E-26 | 51.5547 |
| CNTNAP2 | -1.147098887 | 3.640379182 | -10.07684935 | 8.92E-22 | 2.51E-20 | 38.5754 |
| RGS7 | -1.145420706 | 3.731094985 | -10.67548383 | 5.38E-24 | 1.92E-22 | 43.6244 |
| NAP1L2 | -1.144608003 | 5.12521798 | -12.31610077 | 1.89E-30 | 1.32E-28 | 58.324 |
| MAL2 | -1.143890702 | 2.210172174 | -7.665776235 | 1.01E-13 | 1.24E-12 | 20.3134 |
| CPNE5 | -1.143046895 | 5.57250893 | -11.07271032 | 1.64E-25 | 6.85E-24 | 47.0728 |
| RBFOX3 | -1.142963543 | 2.132830525 | -8.949449695 | 8.02E-18 | 1.53E-16 | 29.595 |
| MSX2 | -1.141701034 | 1.935458726 | -8.961987174 | 7.28E-18 | 1.39E-16 | 29.6908 |
| MIR6071 | -1.141043657 | 2.99539604 | -8.999104252 | 5.45E-18 | 1.06E-16 | 29.975 |
| ACSL6 | -1.140494857 | 4.118572095 | -14.01319526 | 1.35E-37 | 2.11E-35 | 74.6282 |
| FXYD7 | -1.136343371 | 3.984488226 | -7.116019113 | 4.11E-12 | 4.22E-11 | 16.6788 |
| GPR27 | -1.132973938 | 4.380128219 | -10.16607285 | 4.21E-22 | 1.22E-20 | 39.3162 |
| UNC5A | -1.130201742 | 4.407894449 | -10.85569711 | 1.12E-24 | 4.23E-23 | 45.1794 |
| ELAVL2 | -1.126562314 | 3.274969265 | -11.04797985 | 2.05E-25 | 8.45E-24 | 46.8559 |
| AC104051.2 | -1.123225253 | 2.335273469 | -5.040756868 | 6.60E-07 | 3.47E-06 | 5.02492 |
| AC109439.1 | -1.123037016 | 2.178320565 | -9.017958851 | 4.71E-18 | 9.20E-17 | 30.1197 |
| TMEM196 | -1.120039808 | 1.995444932 | -9.319170686 | 4.38E-19 | 9.50E-18 | 32.4598 |
| CHRDL1 | -1.118840734 | 4.227011439 | -8.279919542 | 1.26E-15 | 1.88E-14 | 24.6204 |
| TESPA1 | -1.114938731 | 1.629095718 | -6.852449871 | 2.26E-11 | 2.10E-10 | 15.0135 |
| MIR7-3HG | -1.112273353 | 1.896646676 | -9.361371151 | 3.13E-19 | 6.91E-18 | 32.7919 |
| SMIM18 | -1.109038431 | 3.705269814 | -10.01185451 | 1.54E-21 | 4.23E-20 | 38.0384 |
| SYNPR | -1.106747693 | 2.666039862 | -7.031401406 | 7.14E-12 | 7.11E-11 | 16.1386 |
| MPPED1 | -1.104774028 | 2.108875515 | -7.866544919 | 2.47E-14 | 3.27E-13 | 21.6934 |
| CABP1 | -1.103024391 | 3.017663925 | -9.310124763 | 4.71E-19 | 1.02E-17 | 32.3887 |
| NEURL1 | -1.102903431 | 2.831513706 | -8.544483639 | 1.76E-16 | 2.90E-15 | 26.5523 |
| RAB3A | -1.101499017 | 5.917120775 | -8.926450128 | 9.59E-18 | 1.81E-16 | 29.4195 |
| GUCY1A1 | -1.100131997 | 3.331302932 | -11.69749833 | 5.88E-28 | 3.18E-26 | 52.6439 |
| LINC00599 | -1.100050513 | 3.137680123 | -9.259455111 | 7.05E-19 | 1.49E-17 | 31.9916 |
| CDHR1 | -1.097747361 | 4.294378309 | -10.48175039 | 2.87E-23 | 9.42E-22 | 41.9704 |
| CLVS2 | -1.095186313 | 2.480464188 | -9.282377521 | 5.88E-19 | 1.26E-17 | 32.1711 |
| LRRTM4 | -1.090336951 | 3.413400307 | -12.21036406 | 5.11E-30 | 3.40E-28 | 57.3422 |
| NPTX1 | -1.087638431 | 4.035670113 | -6.467216143 | 2.48E-10 | 2.03E-09 | 12.6729 |
| JPH4 | -1.0850253 | 6.031879754 | -10.97860175 | 3.78E-25 | 1.52E-23 | 46.249 |
| AC068057.2 | -1.083975355 | 2.862167264 | -7.427738253 | 5.15E-13 | 5.86E-12 | 18.7134 |
| MCF2 | -1.082585707 | 2.061949229 | -13.61325254 | 7.10E-36 | 9.24E-34 | 70.7004 |
| SRRM3 | -1.077595663 | 3.499838035 | -9.825619453 | 7.21E-21 | 1.87E-19 | 36.5124 |
| CRYM | -1.076059684 | 2.825981744 | -6.277189546 | 7.77E-10 | 5.98E-09 | 11.56 |
| SLC32A1 | -1.074490273 | 2.459060741 | -6.921845943 | 1.45E-11 | 1.39E-10 | 15.447 |
| KSR2 | -1.074353739 | 1.811504345 | -13.01915131 | 2.33E-33 | 2.30E-31 | 64.9591 |
| NOG | -1.074285642 | 4.387801194 | -9.251050301 | 7.54E-19 | 1.59E-17 | 31.9259 |
| LINC02440 | -1.073444356 | 2.849910468 | -9.365185488 | 3.04E-19 | 6.72E-18 | 32.822 |
| DOK6 | -1.073399387 | 3.000235815 | -11.64489978 | 9.52E-28 | 4.99E-26 | 52.1683 |
| CLVS1 | -1.071479813 | 1.440004986 | -12.94197587 | 4.91E-33 | 4.64E-31 | 64.222 |
| BSN | -1.070158548 | 3.736826421 | -11.32216264 | 1.77E-26 | 8.04E-25 | 49.2761 |
| GAD2 | -1.069872138 | 2.277649542 | -7.256118307 | 1.63E-12 | 1.75E-11 | 17.5846 |
| AP005901.3 | -1.068469106 | 1.223931437 | -12.77803646 | 2.37E-32 | 2.01E-30 | 62.6632 |
| CHRM4 | -1.067737023 | 2.46357986 | -11.74472122 | 3.82E-28 | 2.10E-26 | 53.0719 |
| RIT2 | -1.063739079 | 2.644774444 | -7.20006802 | 2.36E-12 | 2.50E-11 | 17.2205 |
| ATP6V1G2 | -1.062213875 | 7.21144629 | -12.73785481 | 3.48E-32 | 2.89E-30 | 62.2826 |
| RGS7BP | -1.061734375 | 2.909773151 | -9.718914257 | 1.73E-20 | 4.33E-19 | 35.6466 |
| TUNAR | -1.061270752 | 1.751783508 | -9.492514033 | 1.09E-19 | 2.51E-18 | 33.8305 |
| ACBD7 | -1.060137791 | 4.429141794 | -8.219107347 | 1.96E-15 | 2.88E-14 | 24.1827 |
| SLC1A2 | -1.055792041 | 7.681880391 | -9.429293463 | 1.82E-19 | 4.10E-18 | 33.3286 |
| GABRA4 | -1.054791852 | 1.369675633 | -10.14061521 | 5.22E-22 | 1.50E-20 | 39.1044 |
| CDH22 | -1.054481014 | 3.088580698 | -9.894627709 | 4.07E-21 | 1.08E-19 | 37.0757 |
| ARHGDIG | -1.053872592 | 4.508012981 | -8.93930047 | 8.68E-18 | 1.65E-16 | 29.5175 |
| GDAP1L1 | -1.049363774 | 5.399706304 | -9.524015522 | 8.47E-20 | 1.97E-18 | 34.0815 |
| GPR158 | -1.048913652 | 4.557416156 | -12.04717216 | 2.34E-29 | 1.46E-27 | 55.8355 |
| SLIT1 | -1.048872492 | 6.135433371 | -8.720786411 | 4.65E-17 | 8.13E-16 | 27.8645 |
| INSM2 | -1.047096696 | 1.204925279 | -11.85737473 | 1.36E-28 | 7.81E-27 | 54.0969 |
| GNAL | -1.046169294 | 3.526938935 | -13.01026985 | 2.54E-33 | 2.50E-31 | 64.8742 |
| SHANK1 | -1.043331955 | 3.177582507 | -9.10106868 | 2.46E-18 | 4.95E-17 | 30.76 |
| SLC17A8 | -1.043331103 | 1.62238783 | -7.605458841 | 1.53E-13 | 1.84E-12 | 19.9042 |
| PPP1R1A | -1.04240502 | 3.884810485 | -9.698105588 | 2.06E-20 | 5.07E-19 | 35.4785 |
| RUNDC3A | -1.041695355 | 6.232960231 | -11.67800353 | 7.03E-28 | 3.76E-26 | 52.4675 |
| ADARB2 | -1.03542639 | 3.12337477 | -9.752147172 | 1.32E-20 | 3.34E-19 | 35.9156 |
| ASIC4 | -1.031140353 | 5.44543276 | -6.970045088 | 1.06E-11 | 1.03E-10 | 15.7502 |
| C1QL3 | -1.029865258 | 1.719491992 | -6.991571532 | 9.24E-12 | 9.04E-11 | 15.8861 |
| SHANK2 | -1.02859051 | 2.548645899 | -12.04814206 | 2.32E-29 | 1.45E-27 | 55.8445 |
| NELL1 | -1.027431128 | 1.818934194 | -8.696527975 | 5.59E-17 | 9.70E-16 | 27.6828 |
| SGSM1 | -1.027079665 | 3.123457499 | -12.80097082 | 1.90E-32 | 1.63E-30 | 62.8807 |
| MIR124-2HG | -1.026689999 | 2.367522537 | -11.03647699 | 2.27E-25 | 9.32E-24 | 46.7551 |
| SH3GL2 | -1.025564477 | 6.436623359 | -8.537307421 | 1.86E-16 | 3.05E-15 | 26.4993 |
| TMEM155 | -1.024629214 | 1.942578 | -7.576401613 | 1.87E-13 | 2.22E-12 | 19.708 |
| TAFA1 | -1.023819227 | 2.003404555 | -8.445698622 | 3.69E-16 | 5.86E-15 | 25.8257 |
| PAK3 | -1.02238494 | 3.377650896 | -11.03243849 | 2.35E-25 | 9.64E-24 | 46.7197 |
| OLFM1 | -1.019223469 | 6.244856995 | -9.530444595 | 8.04E-20 | 1.88E-18 | 34.1328 |
| NCR3LG1 | -1.01716914 | 1.900832403 | -12.84051712 | 1.30E-32 | 1.14E-30 | 63.2562 |
| SSTR1 | -1.016520129 | 3.119608619 | -7.46944862 | 3.88E-13 | 4.48E-12 | 18.9909 |
| PHF24 | -1.014991332 | 3.228790616 | -6.625497099 | 9.39E-11 | 8.10E-10 | 13.621 |
| MMP24 | -1.014562728 | 4.002083101 | -9.973487561 | 2.12E-21 | 5.75E-20 | 37.7225 |
| CBLN2 | -1.012535095 | 1.93832496 | -9.298619391 | 5.16E-19 | 1.11E-17 | 32.2984 |
| RASD2 | -1.009758145 | 3.12020215 | -9.497111324 | 1.05E-19 | 2.42E-18 | 33.8671 |
| RBFOX1 | -1.009061564 | 2.498487553 | -7.024631091 | 7.46E-12 | 7.40E-11 | 16.0956 |
| RIMS1 | -1.002741549 | 2.073007265 | -10.58715463 | 1.16E-23 | 3.96E-22 | 42.868 |
| RTN4R | -1.001439586 | 3.452576074 | -10.45193334 | 3.70E-23 | 1.20E-21 | 41.7175 |
| KCNAB1 | -0.998013471 | 3.053237148 | -11.18195933 | 6.22E-26 | 2.68E-24 | 48.0342 |
| CACNA2D3 | -0.995455915 | 2.592312845 | -9.923484912 | 3.21E-21 | 8.57E-20 | 37.312 |
| GABRA5 | -0.995267606 | 2.421526938 | -6.541882832 | 1.57E-10 | 1.32E-09 | 13.1177 |
| HAR1A | -0.994741909 | 2.090528567 | -10.6867558 | 4.88E-24 | 1.75E-22 | 43.7212 |
| MAP7D2 | -0.993545329 | 2.402909766 | -6.405556938 | 3.60E-10 | 2.89E-09 | 12.3087 |
| SYT7 | -0.99102519 | 3.700732264 | -6.853308524 | 2.24E-11 | 2.09E-10 | 15.0189 |
| SCG2 | -0.99006179 | 6.709623389 | -7.278103152 | 1.41E-12 | 1.52E-11 | 17.728 |
| CAMSAP3 | -0.989482892 | 3.374894891 | -10.7718652 | 2.32E-24 | 8.52E-23 | 44.4541 |
| BMP2 | -0.986886289 | 5.439489233 | -7.627258216 | 1.32E-13 | 1.59E-12 | 20.0518 |
| SCG3 | -0.986545549 | 8.851355439 | -9.122109262 | 2.08E-18 | 4.23E-17 | 30.9228 |
| CSMD3 | -0.986266158 | 2.5990017 | -9.991505981 | 1.82E-21 | 4.98E-20 | 37.8708 |
| FAM81A | -0.985143543 | 3.094810413 | -10.51115585 | 2.23E-23 | 7.41E-22 | 42.2203 |
| AC104083.1 | -0.985080535 | 3.589882549 | -11.46804672 | 4.75E-27 | 2.30E-25 | 50.5778 |
| MIR7158 | -0.984992924 | 2.422359845 | -8.483343387 | 2.79E-16 | 4.49E-15 | 26.1019 |
| LINC02283 | -0.983935484 | 5.255938068 | -5.75422859 | 1.56E-08 | 1.02E-07 | 8.64404 |
| UBE2QL1 | -0.983517271 | 4.031871368 | -9.702915715 | 1.98E-20 | 4.90E-19 | 35.5173 |
| KCNK12 | -0.981175442 | 1.884525142 | -10.93776322 | 5.42E-25 | 2.13E-23 | 45.8928 |
| GRIA1 | -0.981082792 | 5.738316894 | -7.828259032 | 3.24E-14 | 4.22E-13 | 21.4282 |
| CORO6 | -0.980609879 | 2.440832899 | -9.114400103 | 2.22E-18 | 4.48E-17 | 30.8631 |
| GALNT13 | -0.980446979 | 5.87004609 | -7.80835 | 3.72E-14 | 4.82E-13 | 21.2906 |
| GLRA3 | -0.98031799 | 1.906418996 | -9.278454465 | 6.06E-19 | 1.29E-17 | 32.1404 |
| SLITRK1 | -0.980139972 | 3.782747391 | -9.1877372 | 1.24E-18 | 2.57E-17 | 31.4321 |
| NEUROD6 | -0.978453742 | 1.396377814 | -7.215761063 | 2.13E-12 | 2.26E-11 | 17.3222 |
| SCN2A | -0.977757815 | 3.284899089 | -9.478196934 | 1.23E-19 | 2.80E-18 | 33.7167 |
| GABRG1 | -0.977701537 | 3.624659027 | -7.158094384 | 3.12E-12 | 3.24E-11 | 16.9493 |
| GDA | -0.977431612 | 2.521455393 | -6.134396025 | 1.80E-09 | 1.33E-08 | 10.7423 |
| ANK3 | -0.97625528 | 2.69479689 | -10.16976901 | 4.08E-22 | 1.19E-20 | 39.347 |
| PCDHGC4 | -0.975354576 | 3.76095732 | -8.079917027 | 5.38E-15 | 7.58E-14 | 23.1901 |
| SLC6A7 | -0.97411091 | 1.489402247 | -7.205319913 | 2.28E-12 | 2.42E-11 | 17.2545 |
| PRKCB | -0.971271143 | 4.281533472 | -8.269655404 | 1.36E-15 | 2.02E-14 | 24.5463 |
| SYT16 | -0.970657473 | 2.242190895 | -9.32270408 | 4.26E-19 | 9.27E-18 | 32.4876 |
| STXBP5L | -0.969338665 | 2.138920976 | -9.799743017 | 8.92E-21 | 2.29E-19 | 36.3019 |
| SNCG | -0.963139971 | 5.251108954 | -6.73207836 | 4.83E-11 | 4.32E-10 | 14.2701 |
| STX1B | -0.962004535 | 5.416858309 | -10.48087167 | 2.89E-23 | 9.47E-22 | 41.963 |
| CCKBR | -0.960508564 | 1.657781333 | -8.039122505 | 7.22E-15 | 1.00E-13 | 22.9016 |
| GJD2 | -0.959939953 | 1.23188638 | -9.669965574 | 2.59E-20 | 6.31E-19 | 35.2515 |
| FBXO41 | -0.959818647 | 4.075616817 | -10.82132121 | 1.51E-24 | 5.65E-23 | 44.8816 |
| STXBP6 | -0.95791479 | 2.985807994 | -11.56652875 | 1.94E-27 | 9.87E-26 | 51.4618 |
| PCP4L1 | -0.957037736 | 2.995432996 | -6.406314397 | 3.59E-10 | 2.87E-09 | 12.3132 |
| ANO5 | -0.955932978 | 2.697875732 | -10.8782094 | 9.15E-25 | 3.53E-23 | 45.3748 |
| GRM5 | -0.955107001 | 1.377262837 | -9.852422252 | 5.78E-21 | 1.51E-19 | 36.7309 |
| NGEF | -0.954378345 | 4.162487423 | -6.545975285 | 1.53E-10 | 1.29E-09 | 13.1422 |
| AL354798.1 | -0.95370002 | 2.499835719 | -11.66598224 | 7.85E-28 | 4.18E-26 | 52.3588 |
| CLEC2L | -0.953303355 | 2.13283164 | -7.205221469 | 2.29E-12 | 2.42E-11 | 17.2539 |
| TAGLN3 | -0.953284834 | 6.557167543 | -8.526694497 | 2.02E-16 | 3.29E-15 | 26.421 |
| AC005696.4 | -0.952346612 | 4.122001277 | -9.261593183 | 6.93E-19 | 1.47E-17 | 32.0083 |
| SFRP2 | -0.949163668 | 5.963051848 | -4.046374824 | 6.07E-05 | 0.0002368 | 0.70699 |
| KLRC2 | -0.9434082 | 4.261502932 | -5.39378875 | 1.09E-07 | 6.37E-07 | 6.76282 |
| SEZ6 | -0.942692775 | 5.686484133 | -7.831000171 | 3.18E-14 | 4.14E-13 | 21.4471 |
| KCNC1 | -0.941698099 | 3.036208592 | -10.87650602 | 9.29E-25 | 3.57E-23 | 45.36 |
| SLC2A13 | -0.93944718 | 3.605720907 | -10.60329273 | 1.01E-23 | 3.47E-22 | 43.0059 |
| HCN1 | -0.93868386 | 1.593619365 | -7.467636057 | 3.93E-13 | 4.53E-12 | 18.9788 |
| STXBP1 | -0.938380929 | 6.316218248 | -10.44929447 | 3.79E-23 | 1.23E-21 | 41.6952 |
| ELFN2 | -0.937569363 | 4.798883681 | -7.994685532 | 9.93E-15 | 1.36E-13 | 22.5886 |
| NSG1 | -0.937110939 | 4.325761083 | -7.672138591 | 9.64E-14 | 1.19E-12 | 20.3567 |
| SLC6A1 | -0.936492043 | 7.080928227 | -12.24586902 | 3.66E-30 | 2.48E-28 | 57.6713 |
| ICAM5 | -0.933163005 | 2.788479271 | -6.786288679 | 3.43E-11 | 3.13E-10 | 14.6036 |
| ARHGAP44 | -0.932803589 | 2.518051912 | -10.70527821 | 4.15E-24 | 1.49E-22 | 43.8804 |
| RTN1 | -0.932346958 | 7.330221054 | -9.999969316 | 1.70E-21 | 4.66E-20 | 37.9405 |
| KCNT1 | -0.931110326 | 1.565123901 | -9.434616902 | 1.74E-19 | 3.94E-18 | 33.3708 |
| SMOC1 | -0.931013265 | 8.411645016 | -6.052088047 | 2.90E-09 | 2.08E-08 | 10.2782 |
| ARFGEF3 | -0.928532261 | 4.2306179 | -11.55460739 | 2.17E-27 | 1.09E-25 | 51.3545 |
| MFSD4A | -0.928419566 | 3.283627047 | -8.943481497 | 8.40E-18 | 1.60E-16 | 29.5494 |
| TLX1NB | -0.928340855 | 0.916594721 | -10.59164058 | 1.11E-23 | 3.81E-22 | 42.9063 |
| PAK5 | -0.927344052 | 2.800788952 | -10.0862149 | 8.24E-22 | 2.33E-20 | 38.653 |
| BCYRN1 | -0.92728157 | 2.318352784 | -8.031144401 | 7.65E-15 | 1.06E-13 | 22.8453 |
| DGKG | -0.925653443 | 3.617722019 | -8.623394716 | 9.73E-17 | 1.65E-15 | 27.1372 |
| SNCA | -0.923258224 | 4.426722259 | -7.230837564 | 1.93E-12 | 2.06E-11 | 17.4201 |
| HS3ST2 | -0.920937872 | 2.920170238 | -7.066944687 | 5.67E-12 | 5.72E-11 | 16.3649 |
| ANK1 | -0.92066301 | 2.372316223 | -11.50219341 | 3.49E-27 | 1.72E-25 | 50.8838 |
| SLC30A3 | -0.920385592 | 1.700291994 | -6.520223148 | 1.80E-10 | 1.49E-09 | 12.9882 |
| UNC80 | -0.920217116 | 3.34408996 | -11.16031527 | 7.54E-26 | 3.23E-24 | 47.8433 |
| ASPHD1 | -0.918499921 | 5.376816122 | -9.401116021 | 2.28E-19 | 5.10E-18 | 33.1056 |
| PRKCG | -0.917726462 | 2.967165772 | -5.615128237 | 3.34E-08 | 2.10E-07 | 7.90541 |
| LHFPL3 | -0.915764758 | 6.537496377 | -6.308448327 | 6.45E-10 | 5.01E-09 | 11.7412 |
| SERPINI1 | -0.915476294 | 5.383357379 | -7.093934295 | 4.75E-12 | 4.84E-11 | 16.5373 |
| AMZ1 | -0.914439451 | 2.792164595 | -10.45473342 | 3.62E-23 | 1.17E-21 | 41.7413 |
| ATOH8 | -0.912499723 | 4.923572747 | -8.377826072 | 6.11E-16 | 9.50E-15 | 25.3301 |
| MYH7 | -0.911374472 | 3.98478314 | -7.766533292 | 4.99E-14 | 6.38E-13 | 21.0025 |
| AK5 | -0.91027392 | 3.92512697 | -6.140654257 | 1.74E-09 | 1.28E-08 | 10.7778 |
| LINGO1 | -0.909634178 | 6.457310537 | -9.514494854 | 9.15E-20 | 2.12E-18 | 34.0056 |
| VSTM2L | -0.909310463 | 4.452506675 | -6.508052213 | 1.93E-10 | 1.60E-09 | 12.9156 |
| SMIM10L2B | -0.908541677 | 3.365413258 | -9.002786026 | 5.30E-18 | 1.03E-16 | 30.0032 |
| VAT1L | -0.90821876 | 5.563832423 | -6.972003961 | 1.05E-11 | 1.02E-10 | 15.7625 |
| CELSR3 | -0.907886929 | 3.67534314 | -9.570731167 | 5.80E-20 | 1.38E-18 | 34.4547 |
| SYP | -0.906193042 | 6.459307486 | -9.492794703 | 1.09E-19 | 2.50E-18 | 33.8328 |
| NRXN3 | -0.905827462 | 2.351135732 | -8.927882352 | 9.48E-18 | 1.79E-16 | 29.4304 |
| RASL10A | -0.905029165 | 4.974262065 | -7.227730778 | 1.97E-12 | 2.10E-11 | 17.3999 |
| AC027130.1 | -0.904918652 | 2.39269025 | -10.56557258 | 1.39E-23 | 4.72E-22 | 42.6837 |
| EDA2R | -0.90324652 | 2.401914999 | -6.740280375 | 4.59E-11 | 4.12E-10 | 14.3204 |
| PRMT8 | -0.90252444 | 1.495399666 | -9.341127501 | 3.68E-19 | 8.06E-18 | 32.6325 |
| LRTM2 | -0.902391741 | 2.217729339 | -7.126338895 | 3.84E-12 | 3.96E-11 | 16.745 |
| AL513217.1 | -0.902265206 | 2.236025493 | -9.586360172 | 5.11E-20 | 1.22E-18 | 34.5798 |
| GJB6 | -0.902137979 | 2.45379173 | -5.028315088 | 7.02E-07 | 3.68E-06 | 4.9656 |
| HECW1 | -0.901127775 | 2.004099584 | -10.67852979 | 5.24E-24 | 1.87E-22 | 43.6505 |
| ASIC2 | -0.900238205 | 1.61333962 | -9.605882491 | 4.36E-20 | 1.05E-18 | 34.7363 |
| PLPPR3 | -0.900013744 | 3.098329446 | -7.390969729 | 6.61E-13 | 7.44E-12 | 18.4698 |
| C11orf87 | -0.899989074 | 1.81627063 | -6.479613956 | 2.30E-10 | 1.89E-09 | 12.7464 |
| MIR124-2 | -0.89807289 | 1.463416387 | -9.637452113 | 3.37E-20 | 8.15E-19 | 34.9898 |
| NT5C1A | -0.89767474 | 1.871233545 | -10.66392044 | 5.95E-24 | 2.10E-22 | 43.5251 |
| YPEL4 | -0.897500339 | 3.707392002 | -11.03802781 | 2.23E-25 | 9.20E-24 | 46.7687 |
| LINC00672 | -0.897303718 | 4.815600481 | -8.841093559 | 1.85E-17 | 3.38E-16 | 28.771 |
| PPP1R14C | -0.897271973 | 3.258098156 | -7.409223706 | 5.84E-13 | 6.61E-12 | 18.5906 |
| FAM133A | -0.897164844 | 3.02335028 | -10.08662663 | 8.22E-22 | 2.32E-20 | 38.6564 |
| TAC1 | -0.896313849 | 2.450003314 | -5.6553206 | 2.69E-08 | 1.71E-07 | 8.11721 |
| INSYN2B | -0.895978783 | 2.331885828 | -9.375399934 | 2.80E-19 | 6.21E-18 | 32.9025 |
| KRT222 | -0.894025075 | 1.166459446 | -10.07013231 | 9.43E-22 | 2.64E-20 | 38.5198 |
| AL354863.1 | -0.893979215 | 1.208993329 | -10.88481958 | 8.64E-25 | 3.34E-23 | 45.4322 |
| OLFM4 | -0.893368953 | 1.346458079 | -10.37432937 | 7.20E-23 | 2.27E-21 | 41.0615 |
| CNTNAP5 | -0.892750959 | 1.703654214 | -9.533921671 | 7.82E-20 | 1.83E-18 | 34.1605 |
| OGDHL | -0.888716776 | 2.857290373 | -6.993365571 | 9.14E-12 | 8.94E-11 | 15.8975 |
| CACNA1I | -0.888147352 | 1.65150194 | -9.798558429 | 9.01E-21 | 2.31E-19 | 36.2922 |
| OLFM3 | -0.886921456 | 1.564697254 | -7.475288174 | 3.73E-13 | 4.31E-12 | 19.0298 |
| DOC2A | -0.886293057 | 2.436233697 | -7.338975778 | 9.37E-13 | 1.04E-11 | 18.1269 |
| DIO2 | -0.885387629 | 3.316981833 | -6.810330532 | 2.95E-11 | 2.71E-10 | 14.7522 |
| SHD | -0.884755249 | 6.584927546 | -5.832862514 | 1.01E-08 | 6.77E-08 | 9.06854 |
| GNG3 | -0.883403503 | 5.157287082 | -4.657070309 | 4.17E-06 | 1.95E-05 | 3.25641 |
| ALDOC | -0.883289408 | 9.933016282 | -9.04073696 | 3.94E-18 | 7.76E-17 | 30.2948 |
| SHISAL2B | -0.882820566 | 0.969103269 | -7.944120728 | 1.42E-14 | 1.92E-13 | 22.234 |
| FBXL16 | -0.88258 | 6.567739626 | -8.325011758 | 9.02E-16 | 1.38E-14 | 24.9465 |
| DYNC1I1 | -0.881504192 | 4.018908622 | -8.258775191 | 1.47E-15 | 2.18E-14 | 24.4679 |
| ATP1A3 | -0.881498743 | 7.298627713 | -8.346251302 | 7.71E-16 | 1.19E-14 | 25.1005 |
| GPRIN1 | -0.881411619 | 4.909484735 | -9.885382595 | 4.40E-21 | 1.16E-19 | 37.0001 |
| ATRNL1 | -0.879924536 | 3.659931558 | -9.51913429 | 8.81E-20 | 2.05E-18 | 34.0426 |
| DLGAP3 | -0.87872549 | 3.573106224 | -8.357301808 | 7.11E-16 | 1.10E-14 | 25.1808 |
| TMEM63C | -0.878264613 | 3.503708206 | -11.55111766 | 2.24E-27 | 1.13E-25 | 51.3231 |
| AC107398.3 | -0.875998451 | 3.879385813 | -8.15680625 | 3.09E-15 | 4.45E-14 | 23.7368 |
| PGAP4 | -0.875734626 | 4.299654348 | -10.07929413 | 8.74E-22 | 2.46E-20 | 38.5957 |
| SH2D5 | -0.873955258 | 1.915996793 | -7.15595104 | 3.16E-12 | 3.29E-11 | 16.9355 |
| AL136964.1 | -0.873199792 | 1.349018461 | -14.54327814 | 6.57E-40 | 1.31E-37 | 79.9053 |
| GOLGA7B | -0.87279605 | 3.954704233 | -8.568002257 | 1.48E-16 | 2.45E-15 | 26.7262 |
| HLF | -0.872795566 | 4.473885349 | -12.36774415 | 1.17E-30 | 8.32E-29 | 58.8051 |
| CHD5 | -0.872740528 | 3.019612711 | -6.227504152 | 1.04E-09 | 7.89E-09 | 11.2737 |
| SCGN | -0.872423418 | 1.005884684 | -8.159654149 | 3.02E-15 | 4.37E-14 | 23.7572 |
| CISTR | -0.871911315 | 1.737113392 | -9.982747896 | 1.96E-21 | 5.33E-20 | 37.7987 |
| OPCML | -0.868219772 | 4.308140815 | -7.602114812 | 1.57E-13 | 1.88E-12 | 19.8816 |
| PPFIA2 | -0.867886882 | 2.885101274 | -9.518975455 | 8.82E-20 | 2.05E-18 | 34.0413 |
| KCNJ16 | -0.867538937 | 4.196117286 | -6.408830888 | 3.53E-10 | 2.83E-09 | 12.328 |
| MARCHF11 | -0.866417179 | 1.057167774 | -12.12048448 | 1.18E-29 | 7.63E-28 | 56.5111 |
| SILC1 | -0.865274504 | 3.568256781 | -9.084663736 | 2.80E-18 | 5.60E-17 | 30.6333 |
| TPTE2P1 | -0.864218189 | 2.274542264 | -11.74557281 | 3.79E-28 | 2.09E-26 | 53.0797 |
| NEFH | -0.863563179 | 3.291803628 | -5.865672996 | 8.38E-09 | 5.70E-08 | 9.24714 |
| ARPP21 | -0.863309054 | 4.071018941 | -9.792385737 | 9.48E-21 | 2.43E-19 | 36.2421 |
| FRMPD4 | -0.86155554 | 1.506096408 | -8.118893599 | 4.06E-15 | 5.78E-14 | 23.4667 |
| EPHA10 | -0.860761154 | 1.877784006 | -10.71277747 | 3.89E-24 | 1.40E-22 | 43.9449 |
| SYT5 | -0.860473012 | 3.082961934 | -6.488975368 | 2.17E-10 | 1.79E-09 | 12.8021 |
| VIP | -0.859449255 | 1.728084761 | -7.387767702 | 6.75E-13 | 7.59E-12 | 18.4486 |
| SPRN | -0.858844892 | 3.716529651 | -11.36901356 | 1.16E-26 | 5.38E-25 | 49.6931 |
| RTN4RL2 | -0.858622208 | 3.705517301 | -8.834677573 | 1.95E-17 | 3.54E-16 | 28.7224 |
| SLC38A1 | -0.857500418 | 5.619767381 | -8.682611739 | 6.22E-17 | 1.07E-15 | 27.5787 |
| AC022239.1 | -0.856442196 | 0.871751036 | -8.748939543 | 3.75E-17 | 6.61E-16 | 28.0758 |
| SLC1A1 | -0.856439492 | 4.919589279 | -9.225528598 | 9.22E-19 | 1.93E-17 | 31.7266 |
| CDK5R1 | -0.855872829 | 6.025720032 | -9.515216599 | 9.09E-20 | 2.11E-18 | 34.0113 |
| GRIK2 | -0.855192509 | 4.581955503 | -8.694328378 | 5.69E-17 | 9.86E-16 | 27.6663 |
| CASKIN1 | -0.854361601 | 4.322227001 | -10.47609039 | 3.01E-23 | 9.85E-22 | 41.9224 |
| SST | -0.853302078 | 4.302029966 | -4.974567764 | 9.15E-07 | 4.71E-06 | 4.71082 |
| KCNV1 | -0.852756407 | 1.097309366 | -7.363142669 | 7.97E-13 | 8.88E-12 | 18.286 |
| CNTN4 | -0.852562589 | 1.430420442 | -11.45966256 | 5.12E-27 | 2.47E-25 | 50.5027 |
| SCG5 | -0.852088936 | 6.472037275 | -7.147172122 | 3.35E-12 | 3.48E-11 | 16.879 |
| HTR5A | -0.852086844 | 1.175706443 | -8.404490677 | 5.01E-16 | 7.85E-15 | 25.5245 |
| RBP4 | -0.850882893 | 2.338123144 | -6.321632104 | 5.96E-10 | 4.65E-09 | 11.8178 |
| SAPCD2 | -0.849002397 | 5.275579917 | -6.988070666 | 9.46E-12 | 9.23E-11 | 15.864 |
| TLCD3B | -0.84844896 | 4.420337831 | -9.40750088 | 2.16E-19 | 4.85E-18 | 33.1561 |
| CHRNA4 | -0.847617829 | 2.726410826 | -9.128990381 | 1.98E-18 | 4.02E-17 | 30.976 |
| CACNG8 | -0.847476613 | 3.344512649 | -8.313024838 | 9.85E-16 | 1.50E-14 | 24.8597 |
| MCHR1 | -0.846274848 | 2.663753718 | -6.932255789 | 1.35E-11 | 1.30E-10 | 15.5123 |
| ANKRD55 | -0.845622578 | 1.206922877 | -10.04618515 | 1.15E-21 | 3.22E-20 | 38.3218 |
| SLC8A3 | -0.84503801 | 3.375131655 | -8.860788471 | 1.59E-17 | 2.93E-16 | 28.9202 |
| GLT1D1 | -0.844978111 | 2.190503497 | -6.929224223 | 1.38E-11 | 1.33E-10 | 15.4933 |
| SCN8A | -0.844116287 | 2.986440815 | -9.9376449 | 2.85E-21 | 7.65E-20 | 37.4281 |
| AL139246.5 | -0.843499282 | 1.951870425 | -7.559806446 | 2.09E-13 | 2.48E-12 | 19.5962 |
| AMPH | -0.842635005 | 4.092122513 | -8.009277651 | 8.95E-15 | 1.23E-13 | 22.6912 |
| ST6GAL2 | -0.841685213 | 3.718807021 | -7.635466517 | 1.24E-13 | 1.51E-12 | 20.1075 |
| PID1 | -0.840199034 | 6.424263558 | -9.424591856 | 1.89E-19 | 4.25E-18 | 33.2914 |
| AC124798.1 | -0.839924743 | 2.498760304 | -10.91271964 | 6.76E-25 | 2.63E-23 | 45.6748 |
| CAMK2B | -0.837402418 | 4.380129734 | -6.641984799 | 8.47E-11 | 7.35E-10 | 13.7208 |
| LINC00643 | -0.836318956 | 2.950020457 | -6.742623622 | 4.52E-11 | 4.06E-10 | 14.3348 |
| SPOCK1 | -0.83607619 | 6.420037558 | -8.578776461 | 1.36E-16 | 2.27E-15 | 26.806 |
| SOWAHA | -0.835281382 | 3.095520349 | -6.649248342 | 8.10E-11 | 7.05E-10 | 13.7649 |
| CELF5 | -0.834326699 | 4.256641491 | -7.827510419 | 3.25E-14 | 4.24E-13 | 21.423 |
| ZMAT4 | -0.833417189 | 1.600572953 | -8.539550035 | 1.83E-16 | 3.00E-15 | 26.5159 |
| STYK1 | -0.83315 | 1.039237642 | -8.878837638 | 1.38E-17 | 2.57E-16 | 29.0572 |
| MAPK8IP2 | -0.831630685 | 5.795825207 | -10.69355551 | 4.60E-24 | 1.65E-22 | 43.7796 |
| NGB | -0.830304911 | 1.480308002 | -6.118266077 | 1.98E-09 | 1.45E-08 | 10.6509 |
| HAR1B | -0.83018337 | 1.397161186 | -11.08579997 | 1.46E-25 | 6.13E-24 | 47.1877 |
| CSDC2 | -0.83015897 | 5.909449926 | -5.920417947 | 6.15E-09 | 4.26E-08 | 9.54706 |
| MYT1 | -0.830077755 | 4.427327215 | -6.702020458 | 5.83E-11 | 5.16E-10 | 14.0862 |
| AL033519.1 | -0.829735947 | 2.027953592 | -6.542082623 | 1.57E-10 | 1.32E-09 | 13.1189 |
| RCAN2 | -0.828860835 | 4.733682746 | -7.252005432 | 1.68E-12 | 1.80E-11 | 17.5578 |
| ANKRD33B | -0.827941611 | 2.001978596 | -9.351686014 | 3.38E-19 | 7.45E-18 | 32.7156 |
| AGAP2 | -0.8253334 | 5.186674109 | -7.517450828 | 2.80E-13 | 3.27E-12 | 19.3117 |
| C1orf115 | -0.825045784 | 4.011283503 | -6.632626296 | 8.98E-11 | 7.77E-10 | 13.6641 |
| ATP8A1 | -0.824670745 | 4.817642271 | -10.88064314 | 8.96E-25 | 3.46E-23 | 45.396 |
| TPPP | -0.823979335 | 6.287595451 | -7.724557098 | 6.70E-14 | 8.43E-13 | 20.7146 |
| AC124312.2 | -0.823770272 | 4.136611888 | -7.218880306 | 2.09E-12 | 2.22E-11 | 17.3424 |
| PPP4R4 | -0.823264272 | 2.46490557 | -7.468526882 | 3.91E-13 | 4.50E-12 | 18.9847 |
| DUSP26 | -0.822257191 | 6.175890642 | -9.565377019 | 6.06E-20 | 1.43E-18 | 34.4118 |
| VSTM2A-OT1 | -0.82208364 | 1.022769072 | -11.72603116 | 4.53E-28 | 2.48E-26 | 52.9024 |
| PPP1R16B | -0.821365367 | 4.519264692 | -7.672163091 | 9.64E-14 | 1.19E-12 | 20.3569 |
| CDH9 | -0.820797471 | 1.04478256 | -10.1445229 | 5.05E-22 | 1.46E-20 | 39.1369 |
| PCDH8 | -0.820575304 | 2.565472385 | -7.164599146 | 2.99E-12 | 3.11E-11 | 16.9913 |
| FAIM2 | -0.819666356 | 7.615607333 | -10.41523338 | 5.07E-23 | 1.62E-21 | 41.4069 |
| AL133163.3 | -0.819511024 | 0.92711309 | -11.15714216 | 7.76E-26 | 3.32E-24 | 47.8153 |
| MIR770 | -0.817367579 | 3.694421648 | -5.292751137 | 1.84E-07 | 1.05E-06 | 6.25474 |
| USH1C | -0.817082022 | 4.217871788 | -4.946563909 | 1.05E-06 | 5.36E-06 | 4.57905 |
| PDE1A | -0.815464333 | 2.760062729 | -7.907502996 | 1.85E-14 | 2.47E-13 | 21.9784 |
| SMIM32 | -0.815366349 | 1.301505118 | -8.336891687 | 8.26E-16 | 1.27E-14 | 25.0326 |
| GRIN2B | -0.81520501 | 1.537101055 | -7.868984499 | 2.43E-14 | 3.21E-13 | 21.7104 |
| GALNT17 | -0.814133211 | 3.663614523 | -5.435193673 | 8.76E-08 | 5.19E-07 | 6.97348 |
| RIMS4 | -0.813400875 | 4.351878986 | -6.792709801 | 3.30E-11 | 3.01E-10 | 14.6432 |
| PDZD2 | -0.811799767 | 3.914689194 | -9.1147607 | 2.21E-18 | 4.47E-17 | 30.8659 |
| SLC14A2 | -0.811078731 | 0.8780518 | -10.44568631 | 3.91E-23 | 1.26E-21 | 41.6646 |
| KIF5A | -0.810982164 | 7.144164126 | -7.33580184 | 9.57E-13 | 1.06E-11 | 18.1061 |
| NAPB | -0.810586677 | 5.39842844 | -6.983555613 | 9.74E-12 | 9.50E-11 | 15.8355 |
| SCD | -0.81039453 | 9.49970623 | -9.615399952 | 4.04E-20 | 9.70E-19 | 34.8127 |
| CHST1 | -0.810383217 | 5.465782188 | -7.682369639 | 8.98E-14 | 1.11E-12 | 20.4264 |
| BICDL1 | -0.809741185 | 1.867081179 | -9.180533643 | 1.32E-18 | 2.72E-17 | 31.3761 |
| FBLL1 | -0.808601031 | 4.968899301 | -8.121166991 | 4.00E-15 | 5.69E-14 | 23.4829 |
| PDZD4 | -0.808431421 | 7.473279148 | -10.35481066 | 8.50E-23 | 2.65E-21 | 40.897 |
| KIF5C | -0.80684271 | 6.44938746 | -10.11640528 | 6.40E-22 | 1.82E-20 | 38.9033 |
| GPR22 | -0.806507379 | 1.251420919 | -7.739638924 | 6.03E-14 | 7.63E-13 | 20.8179 |
| CYGB | -0.806332391 | 2.308448772 | -7.755683091 | 5.39E-14 | 6.86E-13 | 20.928 |
| GAD1 | -0.806307287 | 4.401706873 | -6.957973392 | 1.15E-11 | 1.11E-10 | 15.6741 |
| RIMBP2 | -0.806103194 | 2.060753247 | -7.12884095 | 3.78E-12 | 3.89E-11 | 16.7611 |
| DNM1 | -0.804791368 | 4.935950157 | -5.530108791 | 5.29E-08 | 3.23E-07 | 7.46175 |
| SDS | -0.804717669 | 3.323075619 | -7.784855829 | 4.39E-14 | 5.64E-13 | 21.1286 |
| KCNIP3 | -0.804598994 | 4.956909285 | -8.700354298 | 5.43E-17 | 9.44E-16 | 27.7114 |
| PRR36 | -0.803585221 | 5.094980428 | -9.505442385 | 9.84E-20 | 2.27E-18 | 33.9335 |
| LGI3 | -0.803298518 | 4.921312753 | -6.435224512 | 3.01E-10 | 2.43E-09 | 12.4836 |
| NCAN | -0.803104256 | 8.066284704 | -7.005871355 | 8.43E-12 | 8.29E-11 | 15.9766 |
| DLGAP1-AS4 | -0.802632497 | 1.591030906 | -6.796128203 | 3.23E-11 | 2.95E-10 | 14.6643 |
| RTN4RL1 | -0.801673143 | 2.009408599 | -6.606331127 | 1.06E-10 | 9.05E-10 | 13.5051 |
| SLC35F3 | -0.801427691 | 1.605654332 | -8.617362044 | 1.02E-16 | 1.72E-15 | 27.0924 |
| PCSK1 | -0.800866823 | 2.585062777 | -5.652317661 | 2.73E-08 | 1.73E-07 | 8.10134 |
| RASAL1 | -0.8007792 | 2.043986796 | -6.153929385 | 1.61E-09 | 1.19E-08 | 10.8532 |
| SLC1A6 | -0.800573015 | 2.039142386 | -9.967204238 | 2.23E-21 | 6.04E-20 | 37.6709 |
| KCNS1 | -0.8000272 | 1.47758781 | -5.595581859 | 3.72E-08 | 2.32E-07 | 7.80289 |
| SEPTIN3 | -0.79991711 | 7.505414784 | -11.48769522 | 3.98E-27 | 1.94E-25 | 50.7538 |
| KIF3C | -0.798933652 | 6.251519426 | -10.53772307 | 1.77E-23 | 5.95E-22 | 42.4463 |
| MAST1 | -0.79847664 | 4.46549236 | -8.82791931 | 2.05E-17 | 3.72E-16 | 28.6713 |
| AL353597.2 | -0.797755757 | 1.470202576 | -9.018365117 | 4.69E-18 | 9.18E-17 | 30.1228 |
| TBR1 | -0.797199572 | 1.408239162 | -7.182443816 | 2.66E-12 | 2.79E-11 | 17.1065 |
| CPNE6 | -0.796966562 | 2.798882667 | -5.259555847 | 2.19E-07 | 1.23E-06 | 6.08968 |
| PTPRR | -0.796939264 | 1.374561996 | -8.300971438 | 1.08E-15 | 1.63E-14 | 24.7725 |
| AC016717.2 | -0.795909835 | 1.298666998 | -10.35578342 | 8.43E-23 | 2.63E-21 | 40.9052 |
| FGF13 | -0.795594305 | 2.018664248 | -10.03262747 | 1.29E-21 | 3.58E-20 | 38.2098 |
| MEGF11 | -0.794638315 | 4.2867358 | -6.947830005 | 1.23E-11 | 1.18E-10 | 15.6102 |
| LRP4 | -0.794434379 | 6.626435092 | -9.853094714 | 5.74E-21 | 1.50E-19 | 36.7364 |
| TUB | -0.794192385 | 6.017759463 | -11.35246612 | 1.35E-26 | 6.19E-25 | 49.5457 |
| GRAMD1B | -0.793530588 | 4.607245691 | -11.61856505 | 1.21E-27 | 6.27E-26 | 51.9306 |
| SLC17A6 | -0.793454375 | 1.26604432 | -7.934658063 | 1.52E-14 | 2.05E-13 | 22.1679 |
| SHISA7 | -0.793453876 | 4.415678869 | -8.21696471 | 1.99E-15 | 2.92E-14 | 24.1673 |
| HSPA12A | -0.792864652 | 4.370545532 | -9.177431713 | 1.35E-18 | 2.78E-17 | 31.3519 |
| HMGCLL1 | -0.792100509 | 2.056915075 | -8.691565716 | 5.81E-17 | 1.01E-15 | 27.6456 |
| BTBD17 | -0.792068088 | 5.685901784 | -6.624333735 | 9.46E-11 | 8.15E-10 | 13.6139 |
| MTMR7 | -0.791314983 | 2.943630865 | -9.828755199 | 7.02E-21 | 1.82E-19 | 36.5379 |
| OR2L13 | -0.791232885 | 1.610136921 | -8.352466535 | 7.37E-16 | 1.14E-14 | 25.1457 |
| TMEM100 | -0.791007629 | 6.215946326 | -5.540216195 | 5.01E-08 | 3.07E-07 | 7.51418 |
| PRSS3 | -0.78987825 | 2.272941363 | -7.318314386 | 1.08E-12 | 1.18E-11 | 17.9912 |
| CCDC92B | -0.789418438 | 2.586610885 | -9.694754948 | 2.11E-20 | 5.21E-19 | 35.4514 |
| KCNQ2 | -0.788207713 | 5.522782104 | -8.345068653 | 7.78E-16 | 1.20E-14 | 25.0919 |
| GRM3 | -0.784787466 | 3.894916799 | -6.373450944 | 4.37E-10 | 3.47E-09 | 12.1203 |
| PNMA3 | -0.784508462 | 3.707092724 | -7.387890475 | 6.74E-13 | 7.58E-12 | 18.4494 |
| LINC00507 | -0.782803336 | 0.820757263 | -6.787743833 | 3.40E-11 | 3.10E-10 | 14.6126 |
| KCNJ3 | -0.780995083 | 2.588248034 | -6.697941235 | 5.98E-11 | 5.29E-10 | 14.0613 |
| AL031658.2 | -0.780055724 | 1.234064915 | -8.904766301 | 1.13E-17 | 2.12E-16 | 29.2543 |
| NRXN1 | -0.779849336 | 4.793897607 | -8.662474671 | 7.24E-17 | 1.24E-15 | 27.4283 |
| REPS2 | -0.779411682 | 3.75233843 | -8.529125202 | 1.98E-16 | 3.23E-15 | 26.439 |
| AC026790.2 | -0.779352697 | 1.20378834 | -9.973534351 | 2.11E-21 | 5.75E-20 | 37.7229 |
| AL353597.1 | -0.778876499 | 1.146899508 | -10.37763956 | 7.00E-23 | 2.21E-21 | 41.0894 |
| AC021683.1 | -0.778474854 | 1.238338049 | -8.896536497 | 1.21E-17 | 2.26E-16 | 29.1917 |
| VWA5B2 | -0.778459457 | 2.488660456 | -9.124061516 | 2.05E-18 | 4.17E-17 | 30.9379 |
| ZNF488 | -0.778307646 | 5.191343076 | -6.346821083 | 5.13E-10 | 4.03E-09 | 11.9645 |
| NPTXR | -0.778025149 | 6.534995184 | -8.141299981 | 3.45E-15 | 4.95E-14 | 23.6262 |
| CLEC4GP1 | -0.777311707 | 1.021719136 | -8.618638971 | 1.01E-16 | 1.70E-15 | 27.1019 |
| AC097468.2 | -0.776650161 | 0.971638118 | -12.95679392 | 4.25E-33 | 4.03E-31 | 64.3634 |
| CPNE9 | -0.775900927 | 1.576323133 | -7.671290464 | 9.70E-14 | 1.20E-12 | 20.351 |
| LIMS2 | -0.77570737 | 4.98052986 | -6.783895666 | 3.48E-11 | 3.17E-10 | 14.5888 |
| SOHLH1 | -0.775464524 | 1.431313275 | -6.904155345 | 1.62E-11 | 1.54E-10 | 15.3362 |
| PNMA6F | -0.77503325 | 1.243802381 | -6.954074888 | 1.18E-11 | 1.14E-10 | 15.6495 |
| CARMIL2 | -0.773494824 | 1.692988312 | -9.437180315 | 1.71E-19 | 3.86E-18 | 33.3911 |
| AC005740.1 | -0.77295722 | 1.216148867 | -9.512588271 | 9.29E-20 | 2.15E-18 | 33.9904 |
| ENTPD3 | -0.772637995 | 1.569035901 | -7.07313613 | 5.44E-12 | 5.50E-11 | 16.4044 |
| KCNH7 | -0.772366984 | 0.966936371 | -10.39322296 | 6.12E-23 | 1.94E-21 | 41.2209 |
| SYN3 | -0.771243317 | 2.177439088 | -9.06308681 | 3.31E-18 | 6.58E-17 | 30.4669 |
| TCEAL6 | -0.770840811 | 3.521157008 | -5.289801048 | 1.87E-07 | 1.06E-06 | 6.24004 |
| BEX5 | -0.770541289 | 4.358412074 | -5.514724893 | 5.74E-08 | 3.50E-07 | 7.3821 |
| WNT10B | -0.768558297 | 1.631124641 | -6.663351888 | 7.42E-11 | 6.49E-10 | 13.8505 |
| AC092720.2 | -0.768387359 | 1.684914563 | -9.708467041 | 1.89E-20 | 4.69E-19 | 35.5621 |
| TMEM35A | -0.768386307 | 5.139095982 | -9.67578789 | 2.47E-20 | 6.05E-19 | 35.2984 |
| ADGRB3 | -0.768090128 | 4.947928533 | -10.24860774 | 2.10E-22 | 6.28E-21 | 40.0052 |
| PHYHIPL | -0.767664612 | 7.675583338 | -9.816317756 | 7.78E-21 | 2.01E-19 | 36.4367 |
| ANO4 | -0.767470438 | 2.171934744 | -9.485246372 | 1.16E-19 | 2.65E-18 | 33.7727 |
| RXFP1 | -0.767459586 | 1.090637792 | -8.813298445 | 2.29E-17 | 4.14E-16 | 28.5607 |
| CKMT1B | -0.764994914 | 2.05563103 | -8.970143441 | 6.83E-18 | 1.31E-16 | 29.7532 |
| CACNA1E | -0.764670355 | 2.322113052 | -7.75453634 | 5.43E-14 | 6.91E-13 | 20.9201 |
| FGFBP3 | -0.764455574 | 4.812937427 | -7.66280755 | 1.03E-13 | 1.26E-12 | 20.2932 |
| RNU6-353P | -0.76406654 | 1.797667133 | -7.865785916 | 2.48E-14 | 3.28E-13 | 21.6882 |
| KIT | -0.763943748 | 3.465191866 | -7.8937891 | 2.04E-14 | 2.72E-13 | 21.8828 |
| SCRT2 | -0.763464399 | 2.142984007 | -6.73374563 | 4.78E-11 | 4.28E-10 | 14.2803 |
| HKDC1 | -0.761383573 | 2.048591735 | -8.357491782 | 7.10E-16 | 1.10E-14 | 25.1822 |
| GLRB | -0.761362169 | 4.864211545 | -10.21463442 | 2.80E-22 | 8.26E-21 | 39.7212 |
| DGCR9 | -0.761215516 | 3.003062192 | -8.759927617 | 3.45E-17 | 6.11E-16 | 28.1584 |
| EPHB6 | -0.760387313 | 3.517703102 | -5.308182094 | 1.70E-07 | 9.68E-07 | 6.33179 |
| DUSP9 | -0.760123074 | 2.493030422 | -7.119012863 | 4.03E-12 | 4.14E-11 | 16.698 |
| GRIA2 | -0.760101565 | 6.06790266 | -8.105280362 | 4.48E-15 | 6.35E-14 | 23.37 |
| DSCAML1 | -0.759965827 | 4.708342335 | -7.362664461 | 7.99E-13 | 8.90E-12 | 18.2829 |
| ISLR2 | -0.759723812 | 1.83430336 | -6.798826931 | 3.17E-11 | 2.90E-10 | 14.681 |
| HIP1R | -0.759293097 | 6.648950276 | -8.124879438 | 3.89E-15 | 5.55E-14 | 23.5093 |
| AP003355.2 | -0.759192894 | 1.118896743 | -7.034228936 | 7.01E-12 | 6.99E-11 | 16.1566 |
| SLC24A4 | -0.758445872 | 2.562701838 | -6.705991387 | 5.69E-11 | 5.04E-10 | 14.1104 |
| FERMT1 | -0.758125396 | 5.116907589 | -5.06184914 | 5.94E-07 | 3.15E-06 | 5.1258 |
| PHACTR1 | -0.757876393 | 3.830948498 | -9.728758942 | 1.60E-20 | 4.00E-19 | 35.7262 |
| KCNA1 | -0.757538097 | 1.622999998 | -6.971246424 | 1.05E-11 | 1.02E-10 | 15.7578 |
| MIAT | -0.757437006 | 3.838636791 | -6.370086679 | 4.46E-10 | 3.53E-09 | 12.1006 |
| GUCY1B1 | -0.75721997 | 4.334854331 | -8.266289404 | 1.39E-15 | 2.07E-14 | 24.5221 |
| KCNB1 | -0.756984128 | 4.455521187 | -9.231437641 | 8.80E-19 | 1.85E-17 | 31.7727 |
| KCTD16 | -0.756373186 | 1.602782771 | -9.012868714 | 4.90E-18 | 9.56E-17 | 30.0806 |
| PEX5L | -0.755984841 | 3.342979536 | -6.881829991 | 1.87E-11 | 1.77E-10 | 15.1966 |
| DCX | -0.755773144 | 4.383526063 | -5.915289791 | 6.34E-09 | 4.38E-08 | 9.51886 |
| SNORC | -0.755324917 | 3.793649963 | -5.634584796 | 3.01E-08 | 1.90E-07 | 8.00778 |
| FRMPD1 | -0.75499079 | 1.997203124 | -11.0535144 | 1.95E-25 | 8.05E-24 | 46.9044 |
| ETNPPL | -0.75294317 | 6.57444393 | -4.350713467 | 1.66E-05 | 7.11E-05 | 1.9362 |
| VEPH1 | -0.752930573 | 2.473213785 | -4.998898833 | 8.12E-07 | 4.21E-06 | 4.82585 |
| RYR2 | -0.752163185 | 1.313942079 | -7.155613645 | 3.17E-12 | 3.29E-11 | 16.9333 |
| LINC00634 | -0.752003483 | 5.769414676 | -10.01835003 | 1.46E-21 | 4.02E-20 | 38.092 |
| GABRB1 | -0.75151357 | 2.540987165 | -7.875893581 | 2.31E-14 | 3.06E-13 | 21.7584 |
| STEAP2 | -0.751084006 | 2.514978991 | -9.16335779 | 1.51E-18 | 3.09E-17 | 31.2426 |
| KCNF1 | -0.7506902 | 5.07585324 | -6.257739272 | 8.72E-10 | 6.66E-09 | 11.4477 |
| ABTB2 | -0.750638179 | 3.619088946 | -8.972831487 | 6.69E-18 | 1.29E-16 | 29.7738 |
| LRRTM3 | -0.750452635 | 4.34720797 | -8.728770367 | 4.38E-17 | 7.67E-16 | 27.9243 |
| KCNJ4 | -0.749650281 | 3.381791681 | -5.083745982 | 5.33E-07 | 2.84E-06 | 5.23093 |
| CEND1 | -0.749635017 | 7.055789454 | -8.398003928 | 5.26E-16 | 8.22E-15 | 25.4771 |
| KCNH1 | -0.74843562 | 1.718463051 | -8.621892531 | 9.84E-17 | 1.66E-15 | 27.126 |
| AC008780.1 | -0.747312592 | 1.188653587 | -6.823022551 | 2.72E-11 | 2.51E-10 | 14.8308 |
| HIPK4 | -0.746066358 | 1.174355417 | -7.028064567 | 7.30E-12 | 7.25E-11 | 16.1174 |
| KRT8P30 | -0.745679593 | 1.037995603 | -11.89921189 | 9.21E-29 | 5.40E-27 | 54.4788 |
| MAP3K9 | -0.744122264 | 1.789928955 | -8.485763801 | 2.74E-16 | 4.41E-15 | 26.1197 |
| MTCO3P12 | -0.742642249 | 4.12804367 | -5.150526251 | 3.81E-07 | 2.07E-06 | 5.55405 |
| DEFB131E | -0.741728803 | 1.292249688 | -10.80210136 | 1.78E-24 | 6.64E-23 | 44.7153 |
| RAP1GAP2 | -0.740821008 | 3.437057441 | -6.999667847 | 8.77E-12 | 8.61E-11 | 15.9374 |
| STAR | -0.740586313 | 1.277605992 | -12.16205735 | 8.02E-30 | 5.22E-28 | 56.8951 |
| DMTN | -0.740546142 | 4.948916251 | -5.688520354 | 2.24E-08 | 1.44E-07 | 8.29316 |
| LRRTM2 | -0.740248206 | 4.6236881 | -9.283289114 | 5.83E-19 | 1.25E-17 | 32.1782 |
| TEF | -0.740043263 | 5.697619321 | -11.86776901 | 1.23E-28 | 7.13E-27 | 54.1917 |
| TRPC3 | -0.739850388 | 1.767580696 | -10.55986408 | 1.46E-23 | 4.95E-22 | 42.635 |
| MAGEE1 | -0.739572756 | 4.244925237 | -8.904895236 | 1.13E-17 | 2.12E-16 | 29.2553 |
| GALNT16 | -0.73946332 | 3.537042095 | -9.932601138 | 2.97E-21 | 7.97E-20 | 37.3867 |
| CPNE7 | -0.737741058 | 1.7585885 | -6.951467368 | 1.20E-11 | 1.16E-10 | 15.6331 |
| TMEM132B | -0.736929287 | 3.866969379 | -7.092225009 | 4.80E-12 | 4.89E-11 | 16.5264 |
| AP001972.5 | -0.736210905 | 5.443593209 | -7.234412023 | 1.88E-12 | 2.01E-11 | 17.4433 |
| AC074286.1 | -0.736163164 | 2.048223419 | -7.864934154 | 2.50E-14 | 3.30E-13 | 21.6823 |
| SLAIN1 | -0.733816555 | 6.843249539 | -9.507516701 | 9.68E-20 | 2.24E-18 | 33.95 |
| CADM2 | -0.733467293 | 6.005615568 | -9.34518684 | 3.56E-19 | 7.82E-18 | 32.6644 |
| CRY2 | -0.732947882 | 5.671348645 | -12.92106515 | 6.00E-33 | 5.62E-31 | 64.0226 |
| SH3GL3 | -0.732728412 | 3.866616195 | -7.058198632 | 6.00E-12 | 6.02E-11 | 16.3091 |
| SMPD3 | -0.732046387 | 3.413575362 | -8.644222731 | 8.31E-17 | 1.41E-15 | 27.2922 |
| CACNA2D1 | -0.732036232 | 3.670296448 | -7.97902038 | 1.11E-14 | 1.51E-13 | 22.4786 |
| CSPG5 | -0.731280141 | 7.531393887 | -7.168670412 | 2.91E-12 | 3.03E-11 | 17.0175 |
| SEPTIN5 | -0.731261898 | 5.778233804 | -8.00016138 | 9.55E-15 | 1.31E-13 | 22.6271 |
| CKMT1A | -0.731252466 | 1.77132989 | -9.021941169 | 4.57E-18 | 8.93E-17 | 30.1503 |
| SLC24A2 | -0.731131325 | 3.617955195 | -6.256737273 | 8.77E-10 | 6.70E-09 | 11.4419 |
| LINC00839 | -0.731125243 | 3.619101354 | -5.549280372 | 4.77E-08 | 2.94E-07 | 7.56127 |
| AC125616.1 | -0.731055469 | 1.247207488 | -7.928626814 | 1.59E-14 | 2.14E-13 | 22.1257 |
| STUM | -0.730400516 | 4.606373438 | -6.955808409 | 1.16E-11 | 1.13E-10 | 15.6605 |
| PCDH7 | -0.730254851 | 3.778791584 | -8.666553687 | 7.02E-17 | 1.20E-15 | 27.4588 |
| LRRTM1 | -0.730173066 | 2.940113624 | -8.280826571 | 1.25E-15 | 1.87E-14 | 24.6269 |
| KIAA1549L | -0.729577023 | 3.995675062 | -8.603600482 | 1.13E-16 | 1.90E-15 | 26.9901 |
| RIIAD1 | -0.729289674 | 1.568080441 | -10.42182947 | 4.79E-23 | 1.54E-21 | 41.4627 |
| NAP1L3 | -0.726494467 | 6.873042943 | -10.97428494 | 3.93E-25 | 1.57E-23 | 46.2113 |
| MYOD1 | -0.726274313 | 0.835798724 | -7.98001727 | 1.10E-14 | 1.50E-13 | 22.4856 |
| AL136114.1 | -0.726008381 | 1.761948358 | -4.819124882 | 1.94E-06 | 9.54E-06 | 3.9879 |
| AC092681.1 | -0.725933924 | 2.54707312 | -8.011400654 | 8.81E-15 | 1.21E-13 | 22.7062 |
| SELL | -0.725736631 | 5.527587223 | -3.74982726 | 0.0001987 | 0.0007089 | -0.4106 |
| NECAB2 | -0.725418666 | 4.62109101 | -7.338154937 | 9.42E-13 | 1.04E-11 | 18.1215 |
| DOCK3 | -0.723635555 | 4.377695709 | -10.36646836 | 7.70E-23 | 2.42E-21 | 40.9952 |
| NEUROD2 | -0.722847186 | 1.706112928 | -5.884287688 | 7.55E-09 | 5.17E-08 | 9.34885 |
| SLC26A4-AS1 | -0.72277542 | 1.574125087 | -5.545777197 | 4.86E-08 | 2.99E-07 | 7.54307 |
| SLC22A6 | -0.722565512 | 2.509706848 | -6.595096807 | 1.13E-10 | 9.66E-10 | 13.4374 |
| BASP1-AS1 | -0.721525304 | 1.092194122 | -11.45333315 | 5.43E-27 | 2.61E-25 | 50.4461 |
| SPTBN2 | -0.721062301 | 5.258585712 | -8.035232494 | 7.43E-15 | 1.03E-13 | 22.8741 |
| LINC00943 | -0.719985692 | 1.043398752 | -8.798016958 | 2.58E-17 | 4.63E-16 | 28.4454 |
| CYP4X1 | -0.719623906 | 1.769284887 | -7.996141206 | 9.83E-15 | 1.35E-13 | 22.5988 |
| THSD4 | -0.719406272 | 2.115859369 | -9.223982372 | 9.34E-19 | 1.95E-17 | 31.7145 |
| LRFN5 | -0.719398733 | 2.153760475 | -6.912113756 | 1.54E-11 | 1.47E-10 | 15.386 |
| PCBP3 | -0.718920612 | 2.957455082 | -8.910155608 | 1.09E-17 | 2.05E-16 | 29.2953 |
| RHBDL1 | -0.716931519 | 3.438583583 | -8.646560975 | 8.17E-17 | 1.39E-15 | 27.3097 |
| CRLF1 | -0.716477229 | 4.353197583 | -4.090523917 | 5.05E-05 | 0.0001999 | 0.88017 |
| ADRA1B | -0.716301197 | 2.626408872 | -6.91189037 | 1.54E-11 | 1.47E-10 | 15.3846 |
| PLPPR1 | -0.716036716 | 5.993780615 | -5.943293501 | 5.41E-09 | 3.77E-08 | 9.6731 |
| EEF1A2 | -0.715672467 | 7.202496334 | -5.330581043 | 1.51E-07 | 8.69E-07 | 6.44398 |
| AP000843.1 | -0.714941516 | 1.49010936 | -7.781425748 | 4.50E-14 | 5.77E-13 | 21.105 |
| RHBDL3 | -0.714935517 | 5.083160863 | -7.356328492 | 8.34E-13 | 9.27E-12 | 18.2411 |
| CRH | -0.714673326 | 0.965104924 | -8.155962399 | 3.11E-15 | 4.48E-14 | 23.7308 |
| CYP26B1 | -0.712636618 | 2.175401587 | -6.119096043 | 1.97E-09 | 1.44E-08 | 10.6556 |
| PGBD5 | -0.711942385 | 3.70920915 | -9.276746602 | 6.15E-19 | 1.31E-17 | 32.127 |
| TPD52L1 | -0.710812192 | 3.220104755 | -7.44474358 | 4.59E-13 | 5.25E-12 | 18.8264 |
| MRVI1 | -0.71036451 | 5.082622427 | -8.146401474 | 3.33E-15 | 4.78E-14 | 23.6626 |
| CTXN3 | -0.710042762 | 0.790631584 | -6.755622156 | 4.16E-11 | 3.76E-10 | 14.4147 |
| ENHO | -0.710014449 | 8.55842127 | -7.36916152 | 7.65E-13 | 8.54E-12 | 18.3257 |
| RIPPLY2 | -0.709398356 | 4.120777431 | -8.997108449 | 5.54E-18 | 1.07E-16 | 29.9597 |
| WASF1 | -0.707934645 | 6.206227435 | -9.299138202 | 5.14E-19 | 1.11E-17 | 32.3025 |
| CACNA2D2 | -0.707144305 | 2.747337704 | -10.02067133 | 1.43E-21 | 3.94E-20 | 38.1112 |
| HSD11B1 | -0.706821148 | 2.166383776 | -6.064726466 | 2.70E-09 | 1.95E-08 | 10.3491 |
| COL26A1 | -0.705937993 | 2.688606392 | -6.471377579 | 2.42E-10 | 1.98E-09 | 12.6975 |
| HPSE2 | -0.705628446 | 3.522400228 | -4.353816721 | 1.64E-05 | 7.02E-05 | 1.94916 |
| STX1A | -0.705456544 | 4.206629366 | -5.515608539 | 5.71E-08 | 3.48E-07 | 7.38667 |
| KCNQ5 | -0.704602267 | 3.12224574 | -7.00476729 | 8.49E-12 | 8.35E-11 | 15.9696 |
| UNC13C | -0.704450555 | 1.303108362 | -6.79526858 | 3.24E-11 | 2.97E-10 | 14.659 |
| BEX1 | -0.703820597 | 9.079677099 | -9.106741958 | 2.35E-18 | 4.75E-17 | 30.8039 |
| MADCAM1 | -0.703676431 | 1.791637752 | -10.20408242 | 3.06E-22 | 8.98E-21 | 39.6331 |
| FUT9 | -0.703620942 | 3.941817303 | -8.802950218 | 2.48E-17 | 4.47E-16 | 28.4826 |
| AF106564.1 | -0.703269173 | 1.764708497 | -8.80416482 | 2.46E-17 | 4.43E-16 | 28.4918 |
| TUBB4A | -0.703107206 | 8.092544756 | -6.60299876 | 1.08E-10 | 9.23E-10 | 13.485 |
| PKIA | -0.702677251 | 5.809877498 | -8.380758459 | 5.98E-16 | 9.30E-15 | 25.3514 |
| SEMA4A | -0.70216306 | 3.143838861 | -9.212842993 | 1.02E-18 | 2.13E-17 | 31.6276 |
| LINC00320 | -0.701302962 | 3.841314995 | -7.066635256 | 5.68E-12 | 5.73E-11 | 16.3629 |
| SEMA6B | -0.701039491 | 5.623998183 | -8.261723566 | 1.44E-15 | 2.14E-14 | 24.4892 |
| PCLO | -0.700416676 | 1.492824828 | -7.37674713 | 7.27E-13 | 8.14E-12 | 18.3758 |
| HMGCS1 | -0.700322409 | 6.54805746 | -8.834807062 | 1.94E-17 | 3.54E-16 | 28.7234 |
| PRKCE | -0.699815673 | 3.797850675 | -9.286613277 | 5.68E-19 | 1.22E-17 | 32.2043 |
| AL118505.1 | -0.698203434 | 4.375869717 | -4.865800716 | 1.55E-06 | 7.73E-06 | 4.20279 |
| STARD4 | -0.697512953 | 3.218693175 | -7.935209712 | 1.52E-14 | 2.05E-13 | 22.1717 |
| INHBA-AS1 | -0.696910224 | 0.825178164 | -12.15419593 | 8.63E-30 | 5.61E-28 | 56.8224 |
| LINC02559 | -0.696316932 | 1.77465143 | -7.533614378 | 2.51E-13 | 2.95E-12 | 19.4202 |
| CALB2 | -0.695770388 | 2.996003195 | -4.650003489 | 4.31E-06 | 2.01E-05 | 3.22503 |
| SLC9A7 | -0.695600996 | 4.021883429 | -9.824843097 | 7.25E-21 | 1.88E-19 | 36.5061 |
| SLC25A18 | -0.695504872 | 5.959318755 | -7.83189911 | 3.16E-14 | 4.12E-13 | 21.4533 |
| CRHR1 | -0.693367839 | 2.210062602 | -9.202690128 | 1.11E-18 | 2.30E-17 | 31.5485 |
| LINC01551 | -0.693035304 | 1.858082201 | -7.857152546 | 2.64E-14 | 3.48E-13 | 21.6283 |
| KCNB2 | -0.692958298 | 0.796273776 | -9.918475615 | 3.34E-21 | 8.92E-20 | 37.2709 |
| CHN1 | -0.69294842 | 6.041129404 | -5.792486422 | 1.26E-08 | 8.37E-08 | 8.84995 |
| DGCR5 | -0.692746763 | 3.20762368 | -9.31516599 | 4.53E-19 | 9.79E-18 | 32.4283 |
| SPOCK3 | -0.692375728 | 4.496097867 | -5.409292317 | 1.00E-07 | 5.90E-07 | 6.84153 |
| SLC7A4 | -0.692070621 | 1.295720765 | -8.140329079 | 3.48E-15 | 4.98E-14 | 23.6193 |
| JAKMIP1 | -0.691943137 | 3.428747081 | -6.053312574 | 2.88E-09 | 2.07E-08 | 10.2851 |
| RNU6-1028P | -0.69125473 | 1.722562591 | -6.486174489 | 2.21E-10 | 1.82E-09 | 12.7854 |
| AC104024.2 | -0.690779611 | 0.89951473 | -7.073573413 | 5.43E-12 | 5.48E-11 | 16.4072 |
| ADGRV1 | -0.690056738 | 3.903072586 | -6.055817126 | 2.84E-09 | 2.04E-08 | 10.2991 |
| GRIP1 | -0.689482391 | 1.344672954 | -11.77336444 | 2.94E-28 | 1.63E-26 | 53.332 |
| ACVR1C | -0.689139238 | 1.207391878 | -9.899955255 | 3.90E-21 | 1.04E-19 | 37.1193 |
| GRM2 | -0.688503118 | 1.278465771 | -8.261609123 | 1.44E-15 | 2.14E-14 | 24.4883 |
| EMX2OS | -0.688470252 | 2.934297423 | -6.361447882 | 4.70E-10 | 3.71E-09 | 12.05 |
| CD300LG | -0.688239796 | 0.804713262 | -10.11141582 | 6.67E-22 | 1.90E-20 | 38.8619 |
| DGCR10 | -0.687659377 | 2.239441429 | -8.309553575 | 1.01E-15 | 1.53E-14 | 24.8345 |
| MCHR2 | -0.687039574 | 0.727064244 | -7.362044141 | 8.03E-13 | 8.94E-12 | 18.2788 |
| FGF12 | -0.686762182 | 4.600354471 | -7.558591712 | 2.11E-13 | 2.50E-12 | 19.588 |
| NTM | -0.68658295 | 5.562937719 | -10.11653738 | 6.39E-22 | 1.82E-20 | 38.9044 |
| KCNN1 | -0.686115453 | 3.713384647 | -8.037253534 | 7.32E-15 | 1.02E-13 | 22.8884 |
| CAMK2N2 | -0.68571777 | 4.898369737 | -8.033773944 | 7.51E-15 | 1.04E-13 | 22.8638 |
| PRKAR2B | -0.684968798 | 4.148126965 | -8.396466862 | 5.32E-16 | 8.30E-15 | 25.4659 |
| SYNGR1 | -0.68482112 | 5.790853454 | -8.928041215 | 9.47E-18 | 1.79E-16 | 29.4316 |
| DRAIC | -0.684515313 | 1.639883342 | -10.62920839 | 8.04E-24 | 2.80E-22 | 43.2276 |
| KCNS2 | -0.684479472 | 0.981862945 | -7.140949553 | 3.49E-12 | 3.61E-11 | 16.8389 |
| RAB11FIP4 | -0.684053265 | 3.992513338 | -8.703907144 | 5.29E-17 | 9.20E-16 | 27.738 |
| KIF21B | -0.683959636 | 5.16418319 | -8.042469604 | 7.05E-15 | 9.81E-14 | 22.9252 |
| AKAP5 | -0.683427865 | 1.903531146 | -7.810174925 | 3.68E-14 | 4.76E-13 | 21.3032 |
| GRIP2 | -0.682669965 | 1.130174286 | -10.80185785 | 1.79E-24 | 6.65E-23 | 44.7132 |
| TENM2 | -0.681342521 | 1.66487757 | -6.486022429 | 2.21E-10 | 1.82E-09 | 12.7845 |
| AL121956.4 | -0.681167414 | 1.383620214 | -9.574997587 | 5.60E-20 | 1.33E-18 | 34.4888 |
| CPNE4 | -0.681074491 | 1.974737873 | -5.446209704 | 8.26E-08 | 4.91E-07 | 7.02977 |
| DISP2 | -0.680590512 | 2.928198072 | -9.750590885 | 1.34E-20 | 3.38E-19 | 35.9029 |
| FHDC1 | -0.67996832 | 3.105767674 | -6.367511999 | 4.53E-10 | 3.59E-09 | 12.0855 |
| AC113346.1 | -0.679913092 | 0.772158 | -8.794315273 | 2.65E-17 | 4.76E-16 | 28.4174 |
| KLK7 | -0.679576501 | 1.00068249 | -6.530813205 | 1.68E-10 | 1.40E-09 | 13.0515 |
| PRDM8 | -0.679303892 | 3.002548817 | -6.626265213 | 9.34E-11 | 8.06E-10 | 13.6256 |
| SHANK3 | -0.678697989 | 4.452578167 | -9.296078194 | 5.27E-19 | 1.13E-17 | 32.2785 |
| VGF | -0.678623694 | 4.48215917 | -3.536854477 | 0.0004446 | 0.0014932 | -1.1637 |
| PTER | -0.678370753 | 2.533076839 | -4.984844332 | 8.70E-07 | 4.50E-06 | 4.75934 |
| AC012558.1 | -0.678356731 | 2.045843418 | -7.406005032 | 5.97E-13 | 6.75E-12 | 18.5693 |
| PSD2 | -0.678246317 | 6.794492332 | -7.158383151 | 3.11E-12 | 3.24E-11 | 16.9512 |
| SRCIN1 | -0.677958767 | 3.871158489 | -6.693881704 | 6.13E-11 | 5.41E-10 | 14.0365 |
| LINC01602 | -0.677914775 | 1.589960255 | -3.192171389 | 0.0015055 | 0.0045265 | -2.2938 |
| RAPGEF4 | -0.677137066 | 5.02892947 | -6.629497794 | 9.16E-11 | 7.91E-10 | 13.6452 |
| LINC02607 | -0.675376211 | 1.080100795 | -6.342281565 | 5.27E-10 | 4.14E-09 | 11.9381 |
| PRKAR1B | -0.674853989 | 6.156173515 | -6.854108915 | 2.23E-11 | 2.08E-10 | 15.0238 |
| HTR5A-AS1 | -0.674385176 | 0.901894107 | -7.078757417 | 5.25E-12 | 5.31E-11 | 16.4403 |
| HS6ST3 | -0.674030038 | 1.957179284 | -4.95435705 | 1.01E-06 | 5.17E-06 | 4.61565 |
| SYT14 | -0.67330051 | 1.880724107 | -9.374496524 | 2.82E-19 | 6.25E-18 | 32.8954 |
| SLC25A21-AS1 | -0.673202358 | 2.82373056 | -7.823898117 | 3.34E-14 | 4.34E-13 | 21.398 |
| SCARA5 | -0.673002625 | 0.879135273 | -8.119196868 | 4.05E-15 | 5.77E-14 | 23.4689 |
| AL135999.3 | -0.672415466 | 0.926720305 | -13.53047487 | 1.60E-35 | 1.99E-33 | 69.8935 |
| ITPKA | -0.672410898 | 3.509434422 | -6.16848502 | 1.47E-09 | 1.10E-08 | 10.9361 |
| SLC35F1 | -0.672351729 | 5.215255156 | -6.317342219 | 6.12E-10 | 4.76E-09 | 11.7928 |
| MADD-AS1 | -0.671955722 | 1.185261667 | -8.397358271 | 5.28E-16 | 8.25E-15 | 25.4724 |
| Z82196.1 | -0.671707026 | 0.591243028 | -13.08876765 | 1.19E-33 | 1.22E-31 | 65.6258 |
| TSPYL2 | -0.670423962 | 6.234965177 | -8.368333091 | 6.55E-16 | 1.02E-14 | 25.261 |
| SPTB | -0.670041722 | 1.183186171 | -8.654885876 | 7.67E-17 | 1.31E-15 | 27.3717 |
| BRD9P2 | -0.670008203 | 1.828574304 | -8.44813274 | 3.62E-16 | 5.75E-15 | 25.8435 |
| GLP1R | -0.668624781 | 1.026715961 | -6.680179318 | 6.68E-11 | 5.87E-10 | 13.9529 |
| ADAM11 | -0.668003091 | 3.09735709 | -5.991454909 | 4.11E-09 | 2.91E-08 | 9.93982 |
| KCNK1 | -0.667680008 | 3.538694032 | -5.556165125 | 4.60E-08 | 2.84E-07 | 7.59709 |
| RASGRF1 | -0.66631449 | 3.341664865 | -5.529339829 | 5.31E-08 | 3.24E-07 | 7.45776 |
| ANKS1B | -0.666260639 | 3.675988227 | -7.197016353 | 2.41E-12 | 2.54E-11 | 17.2007 |
| KCNK10 | -0.665804047 | 2.274714102 | -7.700966046 | 7.89E-14 | 9.83E-13 | 20.5533 |
| CNTN6 | -0.665754453 | 1.270953523 | -7.860264813 | 2.58E-14 | 3.41E-13 | 21.6499 |
| NALCN | -0.664804363 | 4.28814288 | -9.837931848 | 6.51E-21 | 1.69E-19 | 36.6127 |
| DLGAP1 | -0.663225745 | 4.619346188 | -6.808064517 | 2.99E-11 | 2.75E-10 | 14.7381 |
| CA7 | -0.662456969 | 1.222883706 | -6.608145776 | 1.05E-10 | 8.95E-10 | 13.5161 |
| PNMA6A | -0.66236204 | 3.422085733 | -7.264576358 | 1.54E-12 | 1.66E-11 | 17.6397 |
| FA2H | -0.662272074 | 4.618524693 | -4.991736072 | 8.41E-07 | 4.36E-06 | 4.79193 |
| ADCY5 | -0.660812704 | 4.127553785 | -10.30495311 | 1.30E-22 | 3.98E-21 | 40.4776 |
| ATP2B2 | -0.660235087 | 4.826252024 | -6.888056959 | 1.80E-11 | 1.70E-10 | 15.2355 |
| INSYN2A | -0.660196433 | 2.498968793 | -6.356799776 | 4.83E-10 | 3.81E-09 | 12.0228 |
| GRIA4 | -0.660018839 | 5.778057477 | -6.890276053 | 1.77E-11 | 1.68E-10 | 15.2494 |
| CDH13 | -0.659188018 | 3.532549374 | -6.917346081 | 1.49E-11 | 1.42E-10 | 15.4188 |
| GPR26 | -0.658960156 | 0.744849782 | -7.423080808 | 5.32E-13 | 6.04E-12 | 18.6825 |
| LRRC4C | -0.658787084 | 4.52369322 | -9.517713454 | 8.91E-20 | 2.07E-18 | 34.0312 |
| AP3B2 | -0.658637574 | 4.216971791 | -8.828819761 | 2.04E-17 | 3.70E-16 | 28.6781 |
| MYOZ1 | -0.65776847 | 2.557201364 | -7.477839658 | 3.67E-13 | 4.24E-12 | 19.0468 |
| TAC3 | -0.657324415 | 1.791464121 | -5.15511768 | 3.72E-07 | 2.03E-06 | 5.5764 |
| INSYN1-AS1 | -0.657068651 | 1.971369433 | -8.35267303 | 7.36E-16 | 1.13E-14 | 25.1472 |
| PTPRN2 | -0.656860276 | 6.206787896 | -8.498484789 | 2.49E-16 | 4.03E-15 | 26.2132 |
| ADGRA1 | -0.656816985 | 4.453125847 | -7.738072593 | 6.09E-14 | 7.71E-13 | 20.8072 |
| CSMD1 | -0.656764288 | 2.31042084 | -7.862732038 | 2.54E-14 | 3.35E-13 | 21.667 |
| LINC01007 | -0.656270224 | 0.603639985 | -6.389337787 | 3.97E-10 | 3.17E-09 | 12.2134 |
| GNG4 | -0.656216643 | 5.486182996 | -7.635919963 | 1.24E-13 | 1.51E-12 | 20.1106 |
| FGF14 | -0.65584416 | 2.44729281 | -8.459479043 | 3.33E-16 | 5.31E-15 | 25.9267 |
| CSRNP3 | -0.655555027 | 3.260461968 | -9.155039652 | 1.61E-18 | 3.29E-17 | 31.178 |
| GREM2 | -0.655522832 | 1.325898157 | -7.018001473 | 7.79E-12 | 7.70E-11 | 16.0535 |
| DLG2 | -0.655051043 | 3.545965265 | -7.868306277 | 2.44E-14 | 3.23E-13 | 21.7057 |
| IDS | -0.654226443 | 6.838445382 | -6.879345236 | 1.90E-11 | 1.79E-10 | 15.1811 |
| TSPOAP1 | -0.652358637 | 4.708201394 | -8.277387015 | 1.28E-15 | 1.92E-14 | 24.6021 |
| NDRG4 | -0.6520038 | 7.071517845 | -8.456645282 | 3.40E-16 | 5.42E-15 | 25.9059 |
| LMO3 | -0.65170398 | 4.848640769 | -6.114908254 | 2.02E-09 | 1.48E-08 | 10.6319 |
| AC080100.1 | -0.650834144 | 2.461148949 | -11.32874737 | 1.67E-26 | 7.62E-25 | 49.3347 |
| MAP2 | -0.650305385 | 7.611520618 | -7.840101842 | 2.98E-14 | 3.89E-13 | 21.5101 |
| C3orf80 | -0.650191969 | 1.417966496 | -6.145899902 | 1.68E-09 | 1.25E-08 | 10.8076 |
| SNAP25-AS1 | -0.64821663 | 1.227681017 | -11.20464211 | 5.08E-26 | 2.21E-24 | 48.2345 |
| DGCR6 | -0.647624104 | 3.606755366 | -6.576774423 | 1.27E-10 | 1.08E-09 | 13.3271 |
| SCAMP5 | -0.646763922 | 7.208740846 | -10.29546489 | 1.41E-22 | 4.31E-21 | 40.3979 |
| HS6ST2 | -0.646318072 | 2.989739562 | -7.247988213 | 1.72E-12 | 1.85E-11 | 17.5316 |
| AP006333.1 | -0.645331831 | 4.511796028 | -7.688123922 | 8.63E-14 | 1.07E-12 | 20.4657 |
| FNDC9 | -0.644571483 | 1.976919429 | -5.338594446 | 1.45E-07 | 8.36E-07 | 6.48421 |
| MT-TM | -0.644399295 | 2.800556115 | -4.794420883 | 2.19E-06 | 1.07E-05 | 3.87492 |
| AC124854.1 | -0.643823523 | 2.131938272 | -7.289106037 | 1.31E-12 | 1.42E-11 | 17.7999 |
| G6PC2 | -0.643405624 | 0.621458405 | -8.959354657 | 7.43E-18 | 1.42E-16 | 29.6707 |
| MDGA2 | -0.642721048 | 2.435829263 | -7.722731708 | 6.78E-14 | 8.53E-13 | 20.7021 |
| BEX2 | -0.642365149 | 6.802020712 | -6.764465303 | 3.94E-11 | 3.56E-10 | 14.4691 |
| AL021395.1 | -0.642123871 | 1.324358794 | -7.039665559 | 6.77E-12 | 6.75E-11 | 16.1911 |
| C17orf102 | -0.641813414 | 0.920439867 | -12.1977322 | 5.75E-30 | 3.80E-28 | 57.2252 |
| CACNG4 | -0.640984005 | 7.063433494 | -5.189290454 | 3.13E-07 | 1.72E-06 | 5.74334 |
| MIR486-1 | -0.640499237 | 0.76500781 | -9.818919532 | 7.62E-21 | 1.97E-19 | 36.4578 |
| OMG | -0.640324476 | 6.121633805 | -8.509300859 | 2.30E-16 | 3.73E-15 | 26.2928 |
| ZNF365 | -0.640116752 | 3.360707209 | -8.084638405 | 5.20E-15 | 7.34E-14 | 23.2235 |
| SERTM1 | -0.640036775 | 1.357140547 | -5.45730664 | 7.79E-08 | 4.65E-07 | 7.08657 |
| OTOG | -0.63942148 | 0.57326745 | -7.678474697 | 9.23E-14 | 1.14E-12 | 20.3999 |
| GFOD1 | -0.639084431 | 2.977921159 | -8.074825758 | 5.59E-15 | 7.85E-14 | 23.154 |
| AC104072.1 | -0.63880394 | 2.699443764 | -4.068412158 | 5.54E-05 | 0.0002177 | 0.79322 |
| VXN | -0.637626101 | 6.013963873 | -8.558442292 | 1.59E-16 | 2.63E-15 | 26.6555 |
| EHD3 | -0.637566751 | 5.488006848 | -8.917262077 | 1.03E-17 | 1.94E-16 | 29.3495 |
| WASF3 | -0.637134296 | 6.671669032 | -9.946752371 | 2.64E-21 | 7.12E-20 | 37.5028 |
| TTC9B | -0.636872102 | 4.095372803 | -6.164537574 | 1.51E-09 | 1.12E-08 | 10.9136 |
| CARTPT | -0.635692764 | 0.85410663 | -4.654180097 | 4.22E-06 | 1.98E-05 | 3.24357 |
| SMIM10L2A | -0.634673222 | 3.8627596 | -8.001372762 | 9.47E-15 | 1.30E-13 | 22.6356 |
| EMX1 | -0.634624194 | 1.092649984 | -6.403030923 | 3.66E-10 | 2.93E-09 | 12.2938 |
| SOX8 | -0.63453669 | 8.763549807 | -5.12527458 | 4.33E-07 | 2.34E-06 | 5.43142 |
| PLEKHA6 | -0.633889905 | 4.636155272 | -7.774235129 | 4.73E-14 | 6.06E-13 | 21.0555 |
| DLGAP2 | -0.633800094 | 1.245175344 | -7.413786492 | 5.66E-13 | 6.41E-12 | 18.6208 |
| CPEB3 | -0.633768434 | 3.194037927 | -9.652110142 | 2.99E-20 | 7.25E-19 | 35.1077 |
| LINC02058 | -0.633649694 | 1.965350226 | -5.808510996 | 1.15E-08 | 7.68E-08 | 8.93654 |
| PPM1H | -0.633322147 | 3.569310053 | -9.256460086 | 7.22E-19 | 1.53E-17 | 31.9682 |
| CAVIN2 | -0.632548904 | 4.871904231 | -6.292604668 | 7.09E-10 | 5.48E-09 | 11.6492 |
| TRHDE | -0.63129757 | 0.844491323 | -8.16260219 | 2.96E-15 | 4.28E-14 | 23.7782 |
| GABBR2 | -0.63084167 | 5.658996347 | -4.633553151 | 4.65E-06 | 2.16E-05 | 3.15215 |
| SYCE1 | -0.630551213 | 0.731911618 | -9.077840886 | 2.95E-18 | 5.89E-17 | 30.5806 |
| AMER2 | -0.630410642 | 5.925826457 | -8.157015129 | 3.08E-15 | 4.44E-14 | 23.7383 |
| SLC25A48 | -0.62964844 | 3.62105696 | -4.78709423 | 2.26E-06 | 1.10E-05 | 3.84151 |
| ELOVL4 | -0.629469087 | 3.563385271 | -9.030741105 | 4.26E-18 | 8.36E-17 | 30.2179 |
| MRO | -0.629235224 | 5.440222887 | -6.588045504 | 1.18E-10 | 1.01E-09 | 13.3949 |
| TACR1 | -0.628816962 | 2.26644585 | -5.363945098 | 1.27E-07 | 7.38E-07 | 6.61186 |
| NYAP2 | -0.628595859 | 0.757012458 | -11.1001727 | 1.29E-25 | 5.41E-24 | 47.3139 |
| KLHL32 | -0.628390576 | 4.469285612 | -7.544852953 | 2.32E-13 | 2.73E-12 | 19.4956 |
| SLITRK5 | -0.628371122 | 2.633132098 | -10.04538275 | 1.16E-21 | 3.24E-20 | 38.3152 |
| PLCB1 | -0.627910379 | 4.092569744 | -8.22975723 | 1.81E-15 | 2.67E-14 | 24.2592 |
| HSD17B6 | -0.627700058 | 3.559471409 | -6.853847841 | 2.24E-11 | 2.09E-10 | 15.0222 |
| HMGCR | -0.627597911 | 5.492823408 | -8.609037225 | 1.08E-16 | 1.82E-15 | 27.0305 |
| AC140912.1 | -0.627345799 | 1.949482274 | -8.441897145 | 3.80E-16 | 6.02E-15 | 25.7979 |
| CXCL14 | -0.626989892 | 4.472541764 | -3.371700373 | 0.0008079 | 0.0025755 | -1.719 |
| CA11 | -0.626047086 | 6.678272744 | -6.302459265 | 6.69E-10 | 5.18E-09 | 11.7064 |
| PART1 | -0.625147711 | 1.216831409 | -6.692593556 | 6.18E-11 | 5.46E-10 | 14.0286 |
| LPO | -0.624295005 | 0.650019282 | -12.99070388 | 3.07E-33 | 2.98E-31 | 64.6872 |
| BRSK2 | -0.6242918 | 4.528447748 | -8.058102142 | 6.30E-15 | 8.82E-14 | 23.0357 |
| AC126564.1 | -0.623591023 | 0.793288323 | -7.690775817 | 8.47E-14 | 1.05E-12 | 20.4837 |
| PNMA5 | -0.623416645 | 1.397593896 | -6.552385088 | 1.47E-10 | 1.24E-09 | 13.1806 |
| CBLN4 | -0.623049904 | 1.603553733 | -4.314346086 | 1.95E-05 | 8.26E-05 | 1.78497 |
| MT-TL1 | -0.622587929 | 4.333072373 | -5.396244388 | 1.08E-07 | 6.30E-07 | 6.77527 |
| PHACTR3-AS1 | -0.622261459 | 2.223374564 | -7.977245598 | 1.13E-14 | 1.53E-13 | 22.4661 |
| OPALIN | -0.62203206 | 3.343579637 | -3.059307922 | 0.0023437 | 0.0067739 | -2.6998 |
| SOWAHB | -0.621061805 | 0.857258145 | -6.872312087 | 1.99E-11 | 1.87E-10 | 15.1372 |
| RAB33A | -0.620915702 | 5.415982403 | -7.507463045 | 3.00E-13 | 3.49E-12 | 19.2448 |
| GPR179 | -0.619996647 | 1.270069464 | -9.59410151 | 4.80E-20 | 1.15E-18 | 34.6418 |
| MICAL2 | -0.619784529 | 2.257655274 | -5.339917992 | 1.44E-07 | 8.31E-07 | 6.49087 |
| ENC1 | -0.619446722 | 5.705987131 | -3.850415315 | 0.000134 | 0.0004927 | -0.0404 |
| TNK2 | -0.619429092 | 6.534450517 | -7.406933169 | 5.93E-13 | 6.70E-12 | 18.5754 |
| DNAJC6 | -0.619381789 | 5.358480767 | -7.694336797 | 8.27E-14 | 1.03E-12 | 20.508 |
| MT-RNR2 | -0.618942334 | 14.79945136 | -10.64311268 | 7.12E-24 | 2.50E-22 | 43.3467 |
| THSD7A | -0.618897943 | 3.330236482 | -7.088305183 | 4.93E-12 | 5.01E-11 | 16.5013 |
| EYA1 | -0.618622727 | 3.78447671 | -5.133016642 | 4.16E-07 | 2.25E-06 | 5.46896 |
| RASGRF2 | -0.618448496 | 2.672608048 | -6.912723866 | 1.54E-11 | 1.47E-10 | 15.3898 |
| AC021613.1 | -0.617364545 | 0.936789197 | -6.300120433 | 6.78E-10 | 5.25E-09 | 11.6928 |
| ASPDH | -0.616381033 | 3.275585377 | -6.809146799 | 2.97E-11 | 2.73E-10 | 14.7448 |
| PRRT1 | -0.616356307 | 4.420934115 | -8.630224469 | 9.24E-17 | 1.57E-15 | 27.188 |
| LYNX1 | -0.61438438 | 5.589141294 | -7.570396491 | 1.95E-13 | 2.32E-12 | 19.6675 |
| AC134312.3 | -0.614270384 | 0.88842001 | -6.593292441 | 1.15E-10 | 9.76E-10 | 13.4265 |
| NECAB1 | -0.613780198 | 3.550300737 | -5.166218019 | 3.52E-07 | 1.92E-06 | 5.63052 |
| KCTD4 | -0.613726444 | 3.16487187 | -6.440867628 | 2.91E-10 | 2.36E-09 | 12.5169 |
| SSTR3 | -0.613430924 | 0.914529457 | -7.048208171 | 6.40E-12 | 6.41E-11 | 16.2455 |
| RUNDC3A-AS1 | -0.611899729 | 1.409466692 | -11.82838602 | 1.77E-28 | 1.01E-26 | 53.8326 |
| MN1 | -0.61150814 | 3.992451507 | -7.008216869 | 8.30E-12 | 8.18E-11 | 15.9915 |
| ETV4 | -0.611186075 | 2.510649436 | -3.409824872 | 0.0007054 | 0.0022752 | -1.593 |
| SYT9 | -0.610883047 | 2.771156554 | -7.428027399 | 5.14E-13 | 5.85E-12 | 18.7153 |
| DRP2 | -0.610652827 | 3.429601534 | -8.644444488 | 8.30E-17 | 1.41E-15 | 27.2939 |
| SOX1-OT | -0.61051226 | 3.995966407 | -6.381927722 | 4.16E-10 | 3.30E-09 | 12.1699 |
| KIF1A | -0.610027108 | 7.51450506 | -9.499470496 | 1.03E-19 | 2.38E-18 | 33.8859 |
| TUBA4A | -0.609079629 | 4.600422493 | -4.764137848 | 2.52E-06 | 1.22E-05 | 3.73714 |
| KALRN | -0.608241608 | 2.939045574 | -8.664816924 | 7.11E-17 | 1.22E-15 | 27.4458 |
| KIAA0513 | -0.60818551 | 4.964465909 | -7.079579326 | 5.22E-12 | 5.29E-11 | 16.4455 |
| ALDH5A1 | -0.608167773 | 6.340871754 | -11.49903895 | 3.59E-27 | 1.76E-25 | 50.8555 |
| SLITRK4 | -0.607770911 | 1.590138534 | -6.202661281 | 1.21E-09 | 9.07E-09 | 11.1312 |
| SORCS3 | -0.6072258 | 3.830676626 | -7.017482631 | 7.82E-12 | 7.73E-11 | 16.0502 |
| JAKMIP3 | -0.606789977 | 2.286541299 | -8.680920089 | 6.30E-17 | 1.09E-15 | 27.5661 |
| RALYL | -0.606608491 | 2.531013571 | -5.643677317 | 2.86E-08 | 1.81E-07 | 8.05572 |
| AC243562.2 | -0.60604013 | 2.661247531 | -7.052182982 | 6.24E-12 | 6.25E-11 | 16.2708 |
| MIR1249 | -0.605695745 | 2.720267486 | -6.363192157 | 4.65E-10 | 3.68E-09 | 12.0602 |
| AC006058.1 | -0.605375073 | 2.707145219 | -5.260111353 | 2.18E-07 | 1.22E-06 | 6.09244 |
| LHX6 | -0.604766007 | 1.34687987 | -6.686914407 | 6.41E-11 | 5.64E-10 | 13.994 |
| C1QTNF4 | -0.604722966 | 3.099248252 | -5.784907345 | 1.32E-08 | 8.70E-08 | 8.80906 |
| AC120036.3 | -0.604573615 | 3.882315432 | -8.57034115 | 1.45E-16 | 2.41E-15 | 26.7436 |
| CGREF1 | -0.604491949 | 4.345838892 | -7.838698064 | 3.01E-14 | 3.93E-13 | 21.5004 |
| MT-RNR1 | -0.604445411 | 12.94463561 | -8.83123831 | 2.00E-17 | 3.63E-16 | 28.6964 |
| PNCK | -0.604366618 | 3.199931047 | -6.485475286 | 2.22E-10 | 1.83E-09 | 12.7813 |
| ASIC1 | -0.604170501 | 6.322905933 | -6.951531627 | 1.20E-11 | 1.16E-10 | 15.6335 |
| REEP1 | -0.604060004 | 4.573564251 | -7.212023509 | 2.18E-12 | 2.32E-11 | 17.298 |
| HTR6 | -0.602873101 | 0.643663524 | -11.69007764 | 6.30E-28 | 3.38E-26 | 52.5767 |
| FAM201A | -0.602770582 | 1.535645215 | -8.041522895 | 7.10E-15 | 9.87E-14 | 22.9185 |
| CPAMD8 | -0.602585535 | 0.976061151 | -6.513612932 | 1.87E-10 | 1.55E-09 | 12.9488 |
| PCDHA1 | -0.602506654 | 1.425272522 | -6.993802423 | 9.11E-12 | 8.91E-11 | 15.9002 |
| ACAN | -0.602039591 | 1.36827674 | -5.412270028 | 9.88E-08 | 5.82E-07 | 6.85667 |
| PDYN | -0.601870653 | 2.24865333 | -3.342739383 | 0.000895 | 0.0028224 | -1.8137 |
| AL589765.1 | -0.600866493 | 1.373227068 | -8.470776198 | 3.06E-16 | 4.90E-15 | 26.0096 |
| GPR155 | -0.600755998 | 4.29184004 | -9.344107709 | 3.59E-19 | 7.88E-18 | 32.6559 |
| AL022344.1 | -0.600441155 | 1.544433869 | -7.222961775 | 2.03E-12 | 2.16E-11 | 17.3689 |
| FP325335.1 | -0.600397371 | 1.748977892 | -8.239400992 | 1.69E-15 | 2.50E-14 | 24.3285 |
| RCOR2 | -0.600350792 | 5.740663628 | -5.07555276 | 5.55E-07 | 2.95E-06 | 5.19155 |
| MYO5A | -0.600339352 | 5.11610635 | -8.085899228 | 5.16E-15 | 7.28E-14 | 23.2325 |
| CREB3L1 | -0.600079524 | 2.438901596 | -4.156181279 | 3.84E-05 | 0.0001547 | 1.14095 |
| DGKB | -0.599623091 | 3.557359157 | -6.292003575 | 7.12E-10 | 5.49E-09 | 11.6458 |
| RASSF2 | -0.599150554 | 7.880342865 | -6.954283099 | 1.18E-11 | 1.14E-10 | 15.6508 |
| CYFIP2 | -0.59892298 | 5.829119131 | -9.36639589 | 3.01E-19 | 6.65E-18 | 32.8315 |
| CA4 | -0.598533674 | 3.568238156 | -5.785490961 | 1.31E-08 | 8.68E-08 | 8.81221 |
| HCN2 | -0.59847536 | 5.488807876 | -8.191043513 | 2.41E-15 | 3.51E-14 | 23.9815 |
| PNPLA3 | -0.598374453 | 1.297945166 | -8.820066147 | 2.18E-17 | 3.94E-16 | 28.6119 |
| THY1 | -0.597831201 | 6.724132509 | -6.790445341 | 3.34E-11 | 3.05E-10 | 14.6292 |
| RAB9B | -0.597618551 | 3.740296421 | -9.188197803 | 1.24E-18 | 2.56E-17 | 31.4357 |
| RADX | -0.597102687 | 3.361570187 | -6.396881546 | 3.80E-10 | 3.03E-09 | 12.2577 |
| AC006058.4 | -0.596676578 | 1.938518033 | -5.569976771 | 4.27E-08 | 2.65E-07 | 7.66906 |
| OLMALINC | -0.596502752 | 4.695844005 | -8.837054064 | 1.91E-17 | 3.48E-16 | 28.7404 |
| SAMD12 | -0.595457755 | 1.757851643 | -8.129020747 | 3.78E-15 | 5.39E-14 | 23.5388 |
| BASP1 | -0.595352562 | 7.542851149 | -6.677380629 | 6.80E-11 | 5.97E-10 | 13.9359 |
| ZC3H12B | -0.595150476 | 2.375053595 | -8.917014941 | 1.03E-17 | 1.94E-16 | 29.3476 |
| RGMB | -0.595043617 | 4.518617258 | -7.4957793 | 3.24E-13 | 3.77E-12 | 19.1667 |
| MATK | -0.594469348 | 2.632875925 | -5.675303632 | 2.41E-08 | 1.54E-07 | 8.22301 |
| IL34 | -0.594275566 | 3.140755974 | -5.901767862 | 6.84E-09 | 4.70E-08 | 9.44461 |
| AC004816.1 | -0.594141795 | 2.005102557 | -6.845307527 | 2.36E-11 | 2.20E-10 | 14.9691 |
| SLC6A1-AS1 | -0.59367298 | 2.343818996 | -8.738722766 | 4.06E-17 | 7.13E-16 | 27.999 |
| PIGCP1 | -0.591863668 | 3.978705274 | -8.222332081 | 1.92E-15 | 2.82E-14 | 24.2059 |
| RAP1GAP | -0.59176772 | 5.156307172 | -6.57656079 | 1.27E-10 | 1.08E-09 | 13.3258 |
| IFITM10 | -0.591533877 | 5.311283505 | -5.91158685 | 6.47E-09 | 4.46E-08 | 9.49852 |
| SNTG1 | -0.590948818 | 3.21474555 | -6.399807082 | 3.73E-10 | 2.98E-09 | 12.2749 |
| ELAVL4 | -0.590714497 | 3.975246825 | -5.793060728 | 1.26E-08 | 8.34E-08 | 8.85305 |
| PCDHGC3 | -0.590616981 | 7.534130461 | -5.846716572 | 9.32E-09 | 6.29E-08 | 9.14384 |
| KCNA4 | -0.590376809 | 1.022518904 | -8.397543718 | 5.28E-16 | 8.24E-15 | 25.4738 |
| NELL2 | -0.589958922 | 4.318897183 | -3.714445307 | 0.0002278 | 0.000804 | -0.5386 |
| EPHB1 | -0.589303661 | 5.250961901 | -5.85027535 | 9.14E-09 | 6.18E-08 | 9.16321 |
| LINC01411 | -0.589092188 | 1.534223751 | -5.213043419 | 2.77E-07 | 1.54E-06 | 5.85996 |
| AL731533.2 | -0.58872315 | 2.931686899 | -8.478718549 | 2.88E-16 | 4.64E-15 | 26.0679 |
| INSYN1 | -0.588280963 | 5.051293392 | -7.85568822 | 2.67E-14 | 3.51E-13 | 21.6181 |
| ATP9A | -0.588213875 | 7.222861872 | -9.119235697 | 2.13E-18 | 4.32E-17 | 30.9005 |
| NXPH1 | -0.58801632 | 5.272429896 | -4.689614053 | 3.58E-06 | 1.69E-05 | 3.40148 |
| GPR83 | -0.587742445 | 1.155845348 | -6.005308317 | 3.79E-09 | 2.70E-08 | 10.0169 |
| ST8SIA2 | -0.587677492 | 1.547884582 | -5.907736771 | 6.61E-09 | 4.55E-08 | 9.47737 |
| IQSEC2 | -0.587104141 | 4.308305195 | -6.994722217 | 9.06E-12 | 8.87E-11 | 15.9061 |
| CMBL | -0.586556697 | 4.841106359 | -6.804726765 | 3.05E-11 | 2.80E-10 | 14.7175 |
| IL1RAPL1 | -0.586387519 | 1.751761152 | -9.30012835 | 5.10E-19 | 1.10E-17 | 32.3103 |
| SLC25A27 | -0.5850736 | 4.52676552 | -8.046400205 | 6.85E-15 | 9.55E-14 | 22.953 |
| PCDHGB5 | -0.584511342 | 1.80092367 | -3.754560606 | 0.0001951 | 0.0006968 | -0.3934 |
| GOT1 | -0.5844986 | 6.042525442 | -7.365808645 | 7.83E-13 | 8.73E-12 | 18.3036 |
| NCAM2 | -0.584299435 | 5.387603428 | -8.4069851 | 4.92E-16 | 7.72E-15 | 25.5427 |
| PCDH1 | -0.584267422 | 5.125208509 | -8.53264069 | 1.93E-16 | 3.15E-15 | 26.4649 |
| AC097641.1 | -0.583968377 | 0.955240672 | -9.622338912 | 3.81E-20 | 9.18E-19 | 34.8684 |
| ANGPTL2 | -0.583746895 | 7.175657959 | -5.332616791 | 1.50E-07 | 8.61E-07 | 6.45419 |
| RNU6-277P | -0.583255517 | 2.631134625 | -5.478888798 | 6.95E-08 | 4.18E-07 | 7.19733 |
| THRB | -0.583229288 | 3.363536437 | -7.267480337 | 1.51E-12 | 1.63E-11 | 17.6587 |
| KIF3A | -0.583047706 | 5.321324979 | -9.859102015 | 5.47E-21 | 1.43E-19 | 36.7854 |
| FAXC | -0.582644521 | 2.998754503 | -9.252715772 | 7.44E-19 | 1.57E-17 | 31.9389 |
| DLG4 | -0.581744066 | 6.063111139 | -8.593235025 | 1.22E-16 | 2.05E-15 | 26.9132 |
| UGT8 | -0.581516795 | 5.968607421 | -5.331707567 | 1.51E-07 | 8.65E-07 | 6.44963 |
| SNX22 | -0.581429769 | 5.237791243 | -4.494151051 | 8.78E-06 | 3.92E-05 | 2.54406 |
| DOC2B | -0.581090206 | 2.915336044 | -6.074116412 | 2.55E-09 | 1.85E-08 | 10.4019 |
| BRINP2 | -0.580848334 | 5.744721803 | -6.206040415 | 1.18E-09 | 8.89E-09 | 11.1506 |
| NECTIN1 | -0.580612328 | 4.999070253 | -8.326621247 | 8.91E-16 | 1.36E-14 | 24.9581 |
| C2CD4C | -0.580548581 | 3.37880701 | -6.570737703 | 1.32E-10 | 1.11E-09 | 13.2908 |
| ADGRF5P1 | -0.580482568 | 1.467445679 | -8.874258684 | 1.43E-17 | 2.65E-16 | 29.0224 |
| ACSBG1 | -0.580200951 | 4.062082296 | -6.186006778 | 1.33E-09 | 9.95E-09 | 11.036 |
| AC068308.1 | -0.579789326 | 2.580795647 | -3.993165097 | 7.55E-05 | 0.0002898 | 0.50061 |
| PNMA8B | -0.579433278 | 4.066197898 | -7.037425483 | 6.87E-12 | 6.85E-11 | 16.1769 |
| ATRX | -0.578934276 | 3.825701776 | -7.51238936 | 2.90E-13 | 3.38E-12 | 19.2778 |
| MFSD6 | -0.578644776 | 5.160108711 | -9.406756047 | 2.18E-19 | 4.88E-18 | 33.1502 |
| AC018358.1 | -0.578573259 | 0.727519325 | -6.890441066 | 1.77E-11 | 1.68E-10 | 15.2504 |
| FAM13C | -0.576812507 | 4.161787712 | -8.320823267 | 9.30E-16 | 1.42E-14 | 24.9161 |
| TMEM151A | -0.576541747 | 4.830281484 | -4.891279304 | 1.37E-06 | 6.90E-06 | 4.32089 |
| CD8B2 | -0.57623584 | 1.520539128 | -8.831594591 | 1.99E-17 | 3.62E-16 | 28.6991 |
| SUSD4 | -0.575338232 | 4.883121363 | -7.44162606 | 4.69E-13 | 5.36E-12 | 18.8056 |
| TCEAL5 | -0.57525676 | 6.130948023 | -4.960722812 | 9.79E-07 | 5.02E-06 | 4.64559 |
| CA15P1 | -0.575123861 | 1.328810593 | -7.117086918 | 4.08E-12 | 4.19E-11 | 16.6856 |
| XKR4 | -0.574795488 | 1.732369187 | -8.715134723 | 4.85E-17 | 8.46E-16 | 27.8221 |
| TMEM121B | -0.574113734 | 3.955874093 | -9.031928399 | 4.22E-18 | 8.29E-17 | 30.227 |
| RAI2 | -0.573902218 | 3.714968591 | -9.663963785 | 2.72E-20 | 6.61E-19 | 35.2032 |
| NRIP3 | -0.573757247 | 2.946395081 | -4.484944277 | 9.15E-06 | 4.07E-05 | 2.5045 |
| AL022337.1 | -0.573647771 | 1.611738587 | -6.405262845 | 3.61E-10 | 2.89E-09 | 12.307 |
| PEG10 | -0.573564687 | 6.311992378 | -5.958179506 | 4.97E-09 | 3.48E-08 | 9.75534 |
| RAPGEF5 | -0.57353359 | 3.986899176 | -5.202743989 | 2.92E-07 | 1.62E-06 | 5.80933 |
| ZNF727 | -0.5735017 | 1.674918343 | -6.568359851 | 1.34E-10 | 1.13E-09 | 13.2765 |
| ABCG4 | -0.573407573 | 1.228532611 | -7.471290807 | 3.83E-13 | 4.43E-12 | 19.0032 |
| AC026790.1 | -0.572209 | 0.742625716 | -8.056462851 | 6.38E-15 | 8.92E-14 | 23.0241 |
| AC129929.1 | -0.571945615 | 1.204291298 | -7.605559518 | 1.53E-13 | 1.84E-12 | 19.9049 |
| NEUROD1 | -0.571557031 | 2.268103312 | -5.640262642 | 2.92E-08 | 1.84E-07 | 8.03771 |
| MEPE | -0.571427735 | 0.967435746 | -5.48903779 | 6.58E-08 | 3.98E-07 | 7.24955 |
| MCF2L2 | -0.57122464 | 2.04277491 | -8.540550656 | 1.82E-16 | 2.98E-15 | 26.5233 |
| AC073349.2 | -0.570598744 | 2.694195563 | -6.300432167 | 6.77E-10 | 5.24E-09 | 11.6946 |
| KCNMA1 | -0.569874835 | 3.292264506 | -7.253288057 | 1.66E-12 | 1.79E-11 | 17.5661 |
| HR | -0.569664774 | 3.759499099 | -6.68996022 | 6.29E-11 | 5.54E-10 | 14.0126 |
| GPR61 | -0.569024308 | 1.347807088 | -8.894274332 | 1.23E-17 | 2.29E-16 | 29.1745 |
| CACNA1B | -0.569001486 | 2.03988412 | -5.167866693 | 3.49E-07 | 1.91E-06 | 5.63857 |
| FXYD6 | -0.568435107 | 8.505717437 | -6.579135233 | 1.25E-10 | 1.06E-09 | 13.3413 |
| INSIG1 | -0.568346658 | 5.74879789 | -7.496274129 | 3.23E-13 | 3.76E-12 | 19.17 |
| MTCO1P12 | -0.56780949 | 7.908425657 | -7.081225708 | 5.16E-12 | 5.23E-11 | 16.456 |
| MIR4462 | -0.56771796 | 0.51420542 | -8.312692011 | 9.88E-16 | 1.50E-14 | 24.8573 |
| AC004158.1 | -0.567615886 | 3.627858122 | -8.120956954 | 4.00E-15 | 5.70E-14 | 23.4814 |
| NPY | -0.567372482 | 4.576563471 | -3.60554548 | 0.0003444 | 0.0011808 | -0.9253 |
| VN1R85P | -0.566974567 | 1.130116806 | -5.323423844 | 1.57E-07 | 9.00E-07 | 6.40808 |
| HCN4 | -0.566716065 | 0.949245293 | -8.832835369 | 1.97E-17 | 3.59E-16 | 28.7084 |
| LINC01102 | -0.566670317 | 2.865231967 | -6.586736951 | 1.19E-10 | 1.01E-09 | 13.387 |
| SLC9A2 | -0.566256466 | 0.859977953 | -12.87617332 | 9.24E-33 | 8.32E-31 | 63.5952 |
| NBEA | -0.565769261 | 3.524602113 | -7.639120535 | 1.21E-13 | 1.48E-12 | 20.1323 |
| LINC02731 | -0.565553786 | 2.918567576 | -8.060880524 | 6.18E-15 | 8.65E-14 | 23.0553 |
| DLL3 | -0.565336783 | 7.554727195 | -3.395056714 | 0.0007436 | 0.0023848 | -1.642 |
| TMEM97 | -0.565174794 | 5.201306995 | -7.197207347 | 2.41E-12 | 2.54E-11 | 17.202 |
| AC120036.4 | -0.56516273 | 3.487580087 | -10.87657022 | 9.29E-25 | 3.57E-23 | 45.3606 |
| ARRB1 | -0.564721411 | 4.261804241 | -6.945270908 | 1.25E-11 | 1.20E-10 | 15.5941 |
| KCNIP1 | -0.564041124 | 5.365871021 | -5.39008646 | 1.11E-07 | 6.48E-07 | 6.74405 |
| ADAM22 | -0.563683446 | 5.167472173 | -8.180546884 | 2.60E-15 | 3.78E-14 | 23.9064 |
| CACNA1A | -0.562975208 | 3.775446783 | -6.533565077 | 1.65E-10 | 1.38E-09 | 13.068 |
| GDAP1 | -0.562782668 | 5.557300164 | -9.743459218 | 1.42E-20 | 3.57E-19 | 35.8452 |
| PNOC | -0.56277825 | 1.670160228 | -4.054540262 | 5.87E-05 | 0.0002295 | 0.73889 |
| CMTM5 | -0.561921072 | 5.850666653 | -5.994873306 | 4.03E-09 | 2.85E-08 | 9.95882 |
| MTND2P28 | -0.561908958 | 8.939606896 | -5.182962252 | 3.23E-07 | 1.78E-06 | 5.71235 |
| DNAJC12 | -0.561297937 | 4.847981354 | -9.569631943 | 5.85E-20 | 1.39E-18 | 34.4459 |
| MAP1A | -0.559835056 | 7.199121646 | -8.295633487 | 1.12E-15 | 1.69E-14 | 24.7339 |
| C2orf80 | -0.5593786 | 4.702417181 | -5.37659559 | 1.19E-07 | 6.93E-07 | 6.67576 |
| BLACAT1 | -0.559321816 | 1.31897843 | -9.328301887 | 4.08E-19 | 8.89E-18 | 32.5316 |
| PCDHGA3 | -0.557982863 | 1.609432067 | -5.152746797 | 3.77E-07 | 2.05E-06 | 5.56486 |
| SLC6A15 | -0.557829867 | 1.576628096 | -5.202662829 | 2.93E-07 | 1.62E-06 | 5.80893 |
| ETV5 | -0.557707317 | 5.025048032 | -4.294929438 | 2.12E-05 | 8.93E-05 | 1.70471 |
| SLC24A3 | -0.557657539 | 5.594167607 | -7.098878659 | 4.60E-12 | 4.69E-11 | 16.5689 |
| LINC02151 | -0.55742565 | 0.8405955 | -7.069851801 | 5.56E-12 | 5.62E-11 | 16.3834 |
| NSF | -0.557374476 | 5.669450414 | -7.044391418 | 6.56E-12 | 6.56E-11 | 16.2212 |
| RASGEF1C | -0.557141153 | 4.184085968 | -6.770114394 | 3.80E-11 | 3.45E-10 | 14.5039 |
| AC009227.1 | -0.556909682 | 1.876557232 | -7.061973597 | 5.85E-12 | 5.89E-11 | 16.3332 |
| TGFA | -0.556819416 | 3.280141971 | -6.504400462 | 1.98E-10 | 1.64E-09 | 12.8939 |
| AFF2 | -0.55620408 | 1.794286962 | -7.002386679 | 8.62E-12 | 8.46E-11 | 15.9546 |
| GPR45 | -0.555878892 | 1.839953435 | -6.940310999 | 1.29E-11 | 1.24E-10 | 15.563 |
| EPB41L3 | -0.554948985 | 4.360669877 | -6.211035959 | 1.15E-09 | 8.65E-09 | 11.1792 |
| NAT16 | -0.554937859 | 2.517943075 | -5.288657055 | 1.88E-07 | 1.07E-06 | 6.23434 |
| KCNH3 | -0.554800181 | 3.272660294 | -4.208204166 | 3.08E-05 | 0.0001262 | 1.35032 |
| GRID1 | -0.554636035 | 4.933597938 | -8.257087474 | 1.49E-15 | 2.21E-14 | 24.4558 |
| RAB15 | -0.554496505 | 4.333848995 | -5.77175792 | 1.42E-08 | 9.34E-08 | 8.73824 |
| ALOX12B | -0.554469946 | 0.774520102 | -9.986374609 | 1.90E-21 | 5.18E-20 | 37.8285 |
| LRRN1 | -0.553548215 | 6.490103398 | -5.804430157 | 1.18E-08 | 7.85E-08 | 8.91447 |
| SEC14L5 | -0.552920488 | 2.872474818 | -4.310158954 | 1.98E-05 | 8.40E-05 | 1.76764 |
| FADS6 | -0.552826378 | 0.690237938 | -7.176331665 | 2.77E-12 | 2.89E-11 | 17.067 |
| ANO3 | -0.55256119 | 1.346515535 | -5.831798647 | 1.01E-08 | 6.81E-08 | 9.06276 |
| ESRRG | -0.552456213 | 1.480704119 | -8.495441468 | 2.55E-16 | 4.12E-15 | 26.1908 |
| AL359091.1 | -0.552328468 | 4.306843911 | -4.576294218 | 6.05E-06 | 2.77E-05 | 2.90032 |
| MTUS2 | -0.551889206 | 0.926978115 | -7.532867209 | 2.52E-13 | 2.96E-12 | 19.4151 |
| GAREM2 | -0.551809158 | 5.575117895 | -6.880269161 | 1.89E-11 | 1.78E-10 | 15.1869 |
| NPM2 | -0.551120862 | 2.771897697 | -4.471334967 | 9.73E-06 | 4.31E-05 | 2.44616 |
| INPP5F | -0.550048266 | 4.865456519 | -7.264205249 | 1.54E-12 | 1.66E-11 | 17.6373 |
| DNM3 | -0.549634957 | 4.604069281 | -7.751303294 | 5.56E-14 | 7.06E-13 | 20.8979 |
| PPFIA4 | -0.549307885 | 2.955115143 | -6.187980976 | 1.32E-09 | 9.84E-09 | 11.0473 |
| TSPAN7 | -0.549180908 | 8.657042868 | -9.593071628 | 4.84E-20 | 1.16E-18 | 34.6336 |
| CASQ1 | -0.549053261 | 4.526295865 | -6.523642998 | 1.76E-10 | 1.46E-09 | 13.0087 |
| GAL3ST1 | -0.547818658 | 3.737764145 | -5.616043688 | 3.33E-08 | 2.09E-07 | 7.91022 |
| TMEM169 | -0.547170133 | 3.65129225 | -7.424030688 | 5.28E-13 | 6.00E-12 | 18.6888 |
| CHADL | -0.546960127 | 5.562385329 | -6.116511863 | 2.00E-09 | 1.46E-08 | 10.641 |
| MYO16 | -0.546170533 | 3.02572404 | -5.832573375 | 1.01E-08 | 6.78E-08 | 9.06697 |
| DIRAS2 | -0.546103737 | 4.673239339 | -4.525741827 | 7.61E-06 | 3.43E-05 | 2.68037 |
| AP000282.1 | -0.545684955 | 1.171978172 | -8.689419604 | 5.90E-17 | 1.02E-15 | 27.6296 |
| ZCCHC12 | -0.545444277 | 2.702440021 | -3.737073079 | 0.0002088 | 0.0007419 | -0.4568 |
| LAMP5 | -0.54535691 | 4.761416237 | -4.488420317 | 9.01E-06 | 4.01E-05 | 2.51943 |
| KHDRBS2 | -0.545081197 | 2.172950463 | -5.13046132 | 4.22E-07 | 2.28E-06 | 5.45657 |
| KNDC1 | -0.544919937 | 4.217203594 | -5.631126257 | 3.07E-08 | 1.93E-07 | 7.98956 |
| AP000688.2 | -0.544640799 | 1.64243995 | -6.180610313 | 1.37E-09 | 1.03E-08 | 11.0052 |
| ABLIM2 | -0.543965581 | 3.046006153 | -6.520626956 | 1.79E-10 | 1.49E-09 | 12.9907 |
| HIVEP2 | -0.543711472 | 4.118143611 | -7.856251548 | 2.66E-14 | 3.50E-13 | 21.622 |
| LINC02051 | -0.543669336 | 1.307213841 | -6.300148438 | 6.78E-10 | 5.25E-09 | 11.693 |
| DLG3 | -0.543546191 | 3.957960471 | -7.868383776 | 2.44E-14 | 3.23E-13 | 21.7062 |
| CCDC85A | -0.543420783 | 2.185464528 | -6.43079661 | 3.09E-10 | 2.50E-09 | 12.4574 |
| PANK1 | -0.543259743 | 2.506267783 | -9.371615666 | 2.88E-19 | 6.39E-18 | 32.8727 |
| CDH8 | -0.542960649 | 1.328464329 | -6.349371136 | 5.05E-10 | 3.97E-09 | 11.9794 |
| RN7SL417P | -0.542354244 | 1.626810445 | -6.684192352 | 6.52E-11 | 5.73E-10 | 13.9774 |
| NPPA | -0.542230573 | 4.273680467 | -3.575562814 | 0.0003852 | 0.0013091 | -1.0299 |
| LCN9 | -0.542081533 | 0.825683206 | -6.269850045 | 8.12E-10 | 6.23E-09 | 11.5176 |
| FRMPD2B | -0.542012715 | 0.812187319 | -7.222734803 | 2.04E-12 | 2.17E-11 | 17.3675 |
| NTNG2 | -0.541675708 | 4.063581588 | -7.083976714 | 5.07E-12 | 5.14E-11 | 16.4736 |
| RND1 | -0.541555978 | 4.701225014 | -7.277930474 | 1.41E-12 | 1.53E-11 | 17.7269 |
| GALNT18 | -0.541441993 | 4.369109568 | -8.906924131 | 1.11E-17 | 2.09E-16 | 29.2707 |
| RGS20 | -0.541334859 | 2.9903177 | -5.686497545 | 2.27E-08 | 1.45E-07 | 8.28242 |
| MT-ND1 | -0.540792064 | 13.70091615 | -9.301544277 | 5.05E-19 | 1.09E-17 | 32.3214 |
| EIF4E1B | -0.540395143 | 0.658767053 | -8.731896905 | 4.27E-17 | 7.50E-16 | 27.9478 |
| ATL1 | -0.540235673 | 4.641806086 | -7.683231825 | 8.93E-14 | 1.11E-12 | 20.4323 |
| LINC01785 | -0.540156748 | 0.813596135 | -7.464452999 | 4.02E-13 | 4.62E-12 | 18.9576 |
| LHX2 | -0.539731411 | 4.536423584 | -5.261002228 | 2.17E-07 | 1.22E-06 | 6.09685 |
| CHRNA2 | -0.539622756 | 0.721604948 | -7.351497505 | 8.62E-13 | 9.56E-12 | 18.2093 |
| PITPNM3 | -0.539223095 | 2.657389976 | -4.701322593 | 3.39E-06 | 1.61E-05 | 3.4539 |
| CDKL5 | -0.539112455 | 2.478205511 | -6.291530486 | 7.14E-10 | 5.51E-09 | 11.643 |
| CELF2-DT | -0.538684265 | 0.857165047 | -5.848217694 | 9.24E-09 | 6.25E-08 | 9.15201 |
| TDRD9 | -0.53781373 | 1.210273241 | -7.707509873 | 7.54E-14 | 9.43E-13 | 20.598 |
| SCN3A | -0.537679481 | 4.635999217 | -4.953702403 | 1.01E-06 | 5.19E-06 | 4.61258 |
| PELI3 | -0.536999033 | 4.219751429 | -9.689015589 | 2.21E-20 | 5.45E-19 | 35.4051 |
| P2RX6 | -0.536656509 | 3.16538982 | -7.087720349 | 4.95E-12 | 5.03E-11 | 16.4975 |
| ACAT2 | -0.536608244 | 4.848893571 | -7.44784213 | 4.50E-13 | 5.15E-12 | 18.847 |
| ANKRD24 | -0.536288246 | 3.331311974 | -7.374815875 | 7.37E-13 | 8.24E-12 | 18.3631 |
| B4GALT6 | -0.535835584 | 3.244177453 | -7.015156118 | 7.94E-12 | 7.84E-11 | 16.0355 |
| AC009878.1 | -0.535738399 | 1.272793683 | -8.182023287 | 2.57E-15 | 3.74E-14 | 23.917 |
| TMEM179 | -0.535187949 | 4.190993472 | -4.065624287 | 5.60E-05 | 0.00022 | 0.78228 |
| PWWP3B | -0.535139654 | 1.426956869 | -6.357853505 | 4.80E-10 | 3.79E-09 | 12.029 |
| RGS5 | -0.535118509 | 5.731119678 | -7.609966646 | 1.48E-13 | 1.78E-12 | 19.9347 |
| FEZF2 | -0.534989843 | 2.154701583 | -5.347981465 | 1.38E-07 | 7.98E-07 | 6.53142 |
| CCT7P2 | -0.534892002 | 1.101354861 | -6.850967131 | 2.28E-11 | 2.12E-10 | 15.0043 |
| CHL1 | -0.53450198 | 4.772928992 | -4.284261533 | 2.22E-05 | 9.33E-05 | 1.66076 |
| MSMO1 | -0.534071107 | 6.501275162 | -7.167952982 | 2.92E-12 | 3.05E-11 | 17.0129 |
| USP54 | -0.533382302 | 5.643661002 | -7.30819626 | 1.15E-12 | 1.26E-11 | 17.9249 |
| AQP6 | -0.533243234 | 2.095265902 | -6.927913173 | 1.39E-11 | 1.34E-10 | 15.4851 |
| SV2A | -0.532817681 | 6.848856769 | -8.391254155 | 5.53E-16 | 8.62E-15 | 25.4279 |
| MPP7 | -0.532812744 | 1.041291963 | -8.006481174 | 9.13E-15 | 1.25E-13 | 22.6715 |
| TMOD2 | -0.532531994 | 6.234129164 | -9.025531311 | 4.44E-18 | 8.69E-17 | 30.1779 |
| FAM222A-AS1 | -0.532043302 | 1.904468732 | -6.341348227 | 5.30E-10 | 4.16E-09 | 11.9326 |
| B3GALT2 | -0.531754035 | 3.848099204 | -6.57330067 | 1.30E-10 | 1.10E-09 | 13.3062 |
| EXTL1 | -0.53173391 | 3.088216809 | -5.331463639 | 1.51E-07 | 8.66E-07 | 6.44841 |
| MT-CO1 | -0.531601354 | 14.89867916 | -11.40753345 | 8.21E-27 | 3.85E-25 | 50.0367 |
| CACNB4 | -0.53157558 | 2.774013143 | -7.84378952 | 2.90E-14 | 3.80E-13 | 21.5356 |
| MGAT5B | -0.531444502 | 3.786994195 | -5.437727842 | 8.64E-08 | 5.12E-07 | 6.98642 |
| HRK | -0.53086222 | 2.62337372 | -7.468735947 | 3.90E-13 | 4.50E-12 | 18.9861 |
| STXBP5 | -0.530541776 | 2.80652435 | -7.50637791 | 3.02E-13 | 3.52E-12 | 19.2376 |
| ANKRD29 | -0.530472529 | 1.462472062 | -7.958150334 | 1.29E-14 | 1.75E-13 | 22.3322 |
| ADRA1A | -0.530332222 | 1.932214569 | -6.594283965 | 1.14E-10 | 9.70E-10 | 13.4325 |
| CNTFR | -0.528991983 | 6.731476714 | -5.645804325 | 2.83E-08 | 1.79E-07 | 8.06695 |
| RAB6B | -0.528742005 | 6.788301443 | -7.470891662 | 3.84E-13 | 4.43E-12 | 19.0005 |
| LINC02217 | -0.526937893 | 0.808666907 | -6.445377173 | 2.83E-10 | 2.30E-09 | 12.5435 |
| TRIM17 | -0.526465597 | 1.822817469 | -8.558008156 | 1.59E-16 | 2.64E-15 | 26.6523 |
| MICU3 | -0.526362081 | 3.414623131 | -8.322807895 | 9.17E-16 | 1.40E-14 | 24.9305 |
| KCNAB2 | -0.526123296 | 4.344932555 | -4.821798051 | 1.92E-06 | 9.43E-06 | 4.00015 |
| CACNA1G | -0.525878561 | 2.119468604 | -5.08638414 | 5.26E-07 | 2.81E-06 | 5.24362 |
| MT-TF | -0.525319068 | 4.783587791 | -3.654120352 | 0.0002868 | 0.0009959 | -0.7542 |
| PSD3 | -0.525298012 | 4.407250298 | -6.441739377 | 2.90E-10 | 2.34E-09 | 12.522 |
| WDR86 | -0.525117879 | 2.622887478 | -6.894480107 | 1.73E-11 | 1.64E-10 | 15.2756 |
| LRFN2 | -0.524801518 | 1.860342796 | -5.600529006 | 3.62E-08 | 2.26E-07 | 7.82881 |
| AL121827.1 | -0.524685601 | 0.754364299 | -10.82398785 | 1.47E-24 | 5.53E-23 | 44.9047 |
| AC134312.1 | -0.524466055 | 0.778780563 | -6.740922574 | 4.57E-11 | 4.10E-10 | 14.3244 |
| RUNDC3B | -0.524336979 | 2.847307953 | -8.980053903 | 6.33E-18 | 1.22E-16 | 29.829 |
| CEROX1 | -0.523770574 | 7.472528775 | -4.182135384 | 3.44E-05 | 0.0001398 | 1.2451 |
| BARHL1 | -0.523168777 | 0.642252672 | -6.476688728 | 2.34E-10 | 1.92E-09 | 12.7291 |
| AC022893.2 | -0.521871563 | 0.618703638 | -10.15250213 | 4.72E-22 | 1.37E-20 | 39.2033 |
| ME1 | -0.521698983 | 3.421621496 | -4.960374557 | 9.81E-07 | 5.03E-06 | 4.64395 |
| STXBP5-AS1 | -0.52149761 | 1.062413635 | -10.1409349 | 5.21E-22 | 1.50E-20 | 39.1071 |
| USP27X | -0.52138183 | 3.804685684 | -10.03576988 | 1.26E-21 | 3.49E-20 | 38.2358 |
| CTNNA3 | -0.521365232 | 2.726574506 | -6.246001118 | 9.35E-10 | 7.12E-09 | 11.3801 |
| DNMBP-AS1 | -0.521044257 | 1.455019584 | -5.829232325 | 1.03E-08 | 6.90E-08 | 9.04883 |
| HDAC4 | -0.521028973 | 4.288095292 | -8.194222482 | 2.35E-15 | 3.43E-14 | 24.0043 |
| MYORG | -0.520894652 | 5.397964412 | -6.734257297 | 4.76E-11 | 4.27E-10 | 14.2835 |
| AP000763.2 | -0.520845486 | 2.570058565 | -6.355223085 | 4.88E-10 | 3.84E-09 | 12.0136 |
| AC025211.1 | -0.520704967 | 2.220810179 | -5.515031837 | 5.73E-08 | 3.49E-07 | 7.38369 |
| CDH10 | -0.520601994 | 5.002969788 | -6.171817082 | 1.45E-09 | 1.08E-08 | 10.955 |
| ABAT | -0.520415386 | 7.409785425 | -7.698812523 | 8.01E-14 | 9.98E-13 | 20.5386 |
| PCDHAC2 | -0.520132641 | 1.570591132 | -7.635328069 | 1.24E-13 | 1.51E-12 | 20.1065 |
| LRIT2 | -0.519784034 | 1.231143482 | -8.675529338 | 6.56E-17 | 1.13E-15 | 27.5258 |
| MAGEL2 | -0.519474361 | 2.463498746 | -7.317884544 | 1.08E-12 | 1.18E-11 | 17.9884 |
| ASTN1 | -0.5193902 | 6.257380593 | -7.883597425 | 2.19E-14 | 2.91E-13 | 21.8119 |
| TCEAL2 | -0.51925122 | 7.519206281 | -7.280298249 | 1.39E-12 | 1.50E-11 | 17.7423 |
| MIR5695 | -0.519215695 | 1.494765398 | -5.830916994 | 1.02E-08 | 6.84E-08 | 9.05797 |
| NRG3 | -0.518662681 | 3.805285497 | -5.929620526 | 5.84E-09 | 4.05E-08 | 9.59771 |
| AL391840.1 | -0.518598828 | 2.446862047 | -6.689312073 | 6.31E-11 | 5.56E-10 | 14.0086 |
| EGR4 | -0.518286493 | 0.927866459 | -5.292480914 | 1.85E-07 | 1.05E-06 | 6.2534 |
| AL031710.1 | -0.518270621 | 1.037351491 | -6.615521476 | 9.99E-11 | 8.58E-10 | 13.5607 |
| ZBTB16 | -0.517265435 | 3.680263314 | -4.673784545 | 3.86E-06 | 1.81E-05 | 3.3308 |
| NRXN2 | -0.517023687 | 6.425898504 | -7.711719363 | 7.32E-14 | 9.17E-13 | 20.6268 |
| AFF3 | -0.516832894 | 3.289637265 | -7.414089423 | 5.65E-13 | 6.40E-12 | 18.6228 |
| ZNF676 | -0.51652297 | 0.973892976 | -5.67256945 | 2.45E-08 | 1.56E-07 | 8.20852 |
| DRD1 | -0.516229598 | 1.20964116 | -5.579924353 | 4.04E-08 | 2.51E-07 | 7.72099 |
| RAB3IP | -0.515902211 | 4.724150564 | -5.670807089 | 2.47E-08 | 1.57E-07 | 8.19918 |
| AP000866.4 | -0.515749687 | 3.615819933 | -7.447031554 | 4.52E-13 | 5.18E-12 | 18.8416 |
| PPM1L | -0.515281737 | 4.595018604 | -8.473462652 | 3.00E-16 | 4.81E-15 | 26.0293 |
| LONRF2 | -0.515067515 | 4.316646011 | -6.670884109 | 7.08E-11 | 6.20E-10 | 13.8964 |
| FAM155B | -0.514981611 | 2.694481827 | -7.234350363 | 1.88E-12 | 2.01E-11 | 17.4429 |
| STMN4 | -0.514366395 | 6.426137749 | -5.465872081 | 7.45E-08 | 4.46E-07 | 7.13048 |
| AC007938.1 | -0.513771136 | 2.234312764 | -7.174904791 | 2.79E-12 | 2.92E-11 | 17.0578 |
| CYRIA | -0.513630218 | 5.269231118 | -7.132209663 | 3.70E-12 | 3.81E-11 | 16.7827 |
| LINC00641 | -0.513360376 | 5.280477165 | -8.314409698 | 9.75E-16 | 1.48E-14 | 24.8697 |
| ANKRD34A | -0.513239303 | 2.438165794 | -5.702606028 | 2.07E-08 | 1.34E-07 | 8.36808 |
| HHATL | -0.513229937 | 4.520327975 | -4.077516218 | 5.34E-05 | 0.0002103 | 0.82896 |
| GRIN2A | -0.513077395 | 2.167054696 | -4.008733328 | 7.09E-05 | 0.0002732 | 0.56073 |
| PDZD8 | -0.513039668 | 4.460005408 | -9.750272647 | 1.34E-20 | 3.39E-19 | 35.9004 |
| KCNJ12 | -0.51282241 | 1.158502133 | -7.395139461 | 6.42E-13 | 7.25E-12 | 18.4973 |
| GNAI1 | -0.512660613 | 5.346840743 | -7.496053355 | 3.24E-13 | 3.76E-12 | 19.1685 |
| CACNB1 | -0.512640765 | 3.77440617 | -6.769814257 | 3.81E-11 | 3.45E-10 | 14.502 |
| PDE4A | -0.512585779 | 4.06380729 | -7.89564495 | 2.01E-14 | 2.69E-13 | 21.8958 |
| CNRIP1 | -0.512129835 | 6.263765311 | -9.558688469 | 6.40E-20 | 1.51E-18 | 34.3584 |
| SYBU | -0.511795967 | 5.450348339 | -7.045039117 | 6.54E-12 | 6.53E-11 | 16.2253 |
| AC026620.1 | -0.511403295 | 1.056033515 | -6.75397902 | 4.21E-11 | 3.80E-10 | 14.4046 |
| NEXMIF | -0.511335908 | 2.201259515 | -7.130874384 | 3.73E-12 | 3.85E-11 | 16.7742 |
| BMPER | -0.511104512 | 2.611082629 | -5.127213924 | 4.29E-07 | 2.32E-06 | 5.44082 |
| NDRG2 | -0.511046427 | 9.358184711 | -6.294732399 | 7.00E-10 | 5.41E-09 | 11.6616 |
| AKR1C3 | -0.510452744 | 4.150416727 | -5.178611989 | 3.31E-07 | 1.81E-06 | 5.69107 |
| IQSEC1 | -0.509620716 | 5.311257069 | -6.37218566 | 4.41E-10 | 3.49E-09 | 12.1128 |
| LINC01476 | -0.509618658 | 0.538107892 | -6.528982168 | 1.70E-10 | 1.42E-09 | 13.0406 |
| KIAA1755 | -0.509304796 | 4.431482484 | -6.689533687 | 6.30E-11 | 5.56E-10 | 14.01 |
| PLCH2 | -0.509288523 | 2.534088083 | -5.822665485 | 1.07E-08 | 7.15E-08 | 9.01321 |
| ST6GALNAC5 | -0.508878285 | 1.626639921 | -4.230699347 | 2.80E-05 | 0.0001154 | 1.4416 |
| HTR1E | -0.508610455 | 0.635686145 | -7.381507604 | 7.04E-13 | 7.90E-12 | 18.4072 |
| DCTN1-AS1 | -0.50845172 | 0.846486205 | -8.016183573 | 8.51E-15 | 1.17E-13 | 22.7398 |
| MTARC1 | -0.508332179 | 3.225030049 | -6.79241405 | 3.30E-11 | 3.02E-10 | 14.6414 |
| LINC02520 | -0.508211223 | 0.939170935 | -5.729143635 | 1.79E-08 | 1.16E-07 | 8.50967 |
| AC025263.1 | -0.508152794 | 1.109202164 | -8.029868307 | 7.72E-15 | 1.07E-13 | 22.8363 |
| GDNF-AS1 | -0.507937972 | 2.062730635 | -5.481551277 | 6.85E-08 | 4.13E-07 | 7.21103 |
| RAB6C-AS1 | -0.507530928 | 1.043997683 | -8.143241642 | 3.41E-15 | 4.89E-14 | 23.6401 |
| RPSAP69 | -0.506939428 | 0.679037577 | -7.068428755 | 5.61E-12 | 5.66E-11 | 16.3743 |
| CPE | -0.506760149 | 10.42987917 | -6.140053659 | 1.74E-09 | 1.29E-08 | 10.7744 |
| SLC16A7 | -0.506715571 | 1.817986255 | -9.320992945 | 4.32E-19 | 9.39E-18 | 32.4741 |
| AL353751.1 | -0.506505886 | 1.782819595 | -10.19669197 | 3.25E-22 | 9.54E-21 | 39.5714 |
| AC025811.1 | -0.506408296 | 0.415878499 | -10.97033142 | 4.07E-25 | 1.62E-23 | 46.1768 |
| AKAIN1 | -0.506347316 | 0.652009823 | -8.25979026 | 1.46E-15 | 2.16E-14 | 24.4752 |
| LINC00404 | -0.506223534 | 3.319362793 | -5.077618188 | 5.50E-07 | 2.92E-06 | 5.20147 |
| EPAS1 | -0.505896481 | 6.601873547 | -8.055639666 | 6.41E-15 | 8.97E-14 | 23.0182 |
| ZDHHC11B | -0.505866376 | 3.423370778 | -6.226753304 | 1.05E-09 | 7.92E-09 | 11.2694 |
| PYDC1 | -0.505419341 | 0.673560284 | -7.268304088 | 1.50E-12 | 1.62E-11 | 17.664 |
| AL031593.1 | -0.505209877 | 0.421376958 | -7.577211418 | 1.86E-13 | 2.21E-12 | 19.7135 |
| GPR6 | -0.505102739 | 0.606522977 | -7.41494441 | 5.62E-13 | 6.37E-12 | 18.6285 |
| MT-TP | -0.504654592 | 10.96846772 | -6.462076148 | 2.56E-10 | 2.09E-09 | 12.6424 |
| SMAD9 | -0.504574625 | 5.264678813 | -6.412253855 | 3.46E-10 | 2.78E-09 | 12.3481 |
| BSPRY | -0.504257322 | 1.46433624 | -7.595247238 | 1.64E-13 | 1.97E-12 | 19.8352 |
| MADD | -0.503813722 | 5.621691222 | -10.15580718 | 4.59E-22 | 1.33E-20 | 39.2308 |
| Z82196.2 | -0.50365603 | 0.359604205 | -12.49940354 | 3.36E-31 | 2.55E-29 | 60.0363 |
| DRG2 | -0.503621934 | 3.303409224 | -7.265092776 | 1.54E-12 | 1.66E-11 | 17.6431 |
| MIR1250 | -0.503038547 | 1.737338041 | -5.592203245 | 3.79E-08 | 2.36E-07 | 7.7852 |
| LINC02223 | -0.502579936 | 0.598975072 | -8.032678176 | 7.56E-15 | 1.05E-13 | 22.8561 |
| LY86-AS1 | -0.5023491 | 0.65422255 | -6.824111801 | 2.70E-11 | 2.50E-10 | 14.8375 |
| KIF26A | -0.502332566 | 3.005857693 | -4.833833421 | 1.81E-06 | 8.94E-06 | 4.05541 |
| FAM222A | -0.501140931 | 5.68943306 | -5.487724074 | 6.63E-08 | 4.00E-07 | 7.24279 |
| PABPC5 | -0.500919581 | 2.82609911 | -5.710874773 | 1.98E-08 | 1.28E-07 | 8.41214 |
| CLGN | -0.500576485 | 3.153507833 | -5.315656001 | 1.64E-07 | 9.34E-07 | 6.36918 |
