# Supplementary material for: MAGOH is correlated with poor prognosis and is essential for cell proliferation in lower-grade glioma
Source: Aging (Albany NY). 2023 Jun 30;15(12):5713–33. doi: 10.18632/aging.204823 (PMC10333088; doi:10.18632/aging.204823)
Supplement: Supplementary Table 2 [file aging-15-204823-s003.docx]

**Supplementary Table 2. Up-regulated DEGs in TCGA cohort.**

| **id** | **logFC** | **AveExpr** | **t** | **P.Value** | **adj.P.Val** | **B** |
| --- | --- | --- | --- | --- | --- | --- |
| ABCD1 | 0.50012 | 3.535013 | 9.67452 | 2.49E-20 | 6.10E-19 | 35.2882 |
| PSMC3IP | 0.50013 | 3.041823 | 10.8039 | 1.76E-24 | 6.54E-23 | 44.7306 |
| IFRD1 | 0.50015 | 4.674409 | 11.7018 | 5.66E-28 | 3.07E-26 | 52.6829 |
| AC012150.2 | 0.50031 | 1.494594 | 6.03118 | 3.27E-09 | 2.34E-08 | 10.1612 |
| DEFB124 | 0.50034 | 0.650201 | 5.37361 | 1.21E-07 | 7.03E-07 | 6.66066 |
| AL121894.2 | 0.50047 | 3.393678 | 4.99522 | 8.27E-07 | 4.29E-06 | 4.80843 |
| ZNF644 | 0.50048 | 4.356714 | 10.0016 | 1.67E-21 | 4.60E-20 | 37.9539 |
| VRK3 | 0.501 | 3.344106 | 15.8588 | 8.89E-46 | 3.10E-43 | 93.304 |
| PCDH18 | 0.50102 | 2.890944 | 6.95854 | 1.14E-11 | 1.11E-10 | 15.6777 |
| VWA3A | 0.50103 | 1.651655 | 4.95227 | 1.02E-06 | 5.22E-06 | 4.60585 |
| IER2 | 0.50111 | 5.682406 | 7.56413 | 2.03E-13 | 2.41E-12 | 19.6253 |
| MGST1 | 0.50111 | 4.28425 | 5.25161 | 2.28E-07 | 1.28E-06 | 6.05033 |
| LINC01896 | 0.5014 | 5.330734 | 5.09636 | 5.00E-07 | 2.67E-06 | 5.29168 |
| DHRSX | 0.50141 | 4.137737 | 9.93845 | 2.83E-21 | 7.61E-20 | 37.4347 |
| ZNF613 | 0.50144 | 2.289934 | 14.5554 | 5.81E-40 | 1.17E-37 | 80.0265 |
| INPP5B | 0.50157 | 3.109313 | 14.6026 | 3.60E-40 | 7.48E-38 | 80.5004 |
| TWF2 | 0.50176 | 5.715991 | 12.2318 | 4.18E-30 | 2.82E-28 | 57.5407 |
| RPS11 | 0.5019 | 10.57884 | 10.2662 | 1.81E-22 | 5.45E-21 | 40.1527 |
| ZNF875 | 0.50198 | 4.106948 | 14.8482 | 2.97E-41 | 6.62E-39 | 82.9749 |
| AC069281.2 | 0.50218 | 2.393595 | 7.97433 | 1.15E-14 | 1.56E-13 | 22.4456 |
| BMP1 | 0.50227 | 3.803299 | 8.98733 | 5.98E-18 | 1.16E-16 | 29.8848 |
| TRIM5 | 0.50231 | 2.999682 | 6.40899 | 3.53E-10 | 2.83E-09 | 12.3289 |
| ZNF260 | 0.50231 | 4.22403 | 9.67707 | 2.44E-20 | 5.99E-19 | 35.3088 |
| LINC01341 | 0.50242 | 1.373123 | 9.45973 | 1.42E-19 | 3.23E-18 | 33.57 |
| SEM1 | 0.50254 | 4.015024 | 13.609 | 7.40E-36 | 9.59E-34 | 70.659 |
| NOP10 | 0.50265 | 7.448576 | 13.2595 | 2.27E-34 | 2.55E-32 | 67.2675 |
| ITPRIP | 0.50295 | 3.188766 | 7.91449 | 1.76E-14 | 2.36E-13 | 22.0271 |
| XRCC2 | 0.50301 | 1.464416 | 7.19589 | 2.43E-12 | 2.56E-11 | 17.1935 |
| KMT2B | 0.5033 | 4.271149 | 10.7187 | 3.69E-24 | 1.34E-22 | 43.9955 |
| AP000439.2 | 0.5035 | 1.632758 | 6.76122 | 4.02E-11 | 3.63E-10 | 14.4491 |
| MAD2L1 | 0.50366 | 2.724292 | 7.11982 | 4.01E-12 | 4.12E-11 | 16.7032 |
| CFAP100 | 0.50368 | 0.568564 | 6.75329 | 4.23E-11 | 3.81E-10 | 14.4004 |
| IGHG4 | 0.50371 | 0.778923 | 4.93647 | 1.10E-06 | 5.61E-06 | 4.53172 |
| WDR76 | 0.50376 | 2.811579 | 6.87255 | 1.99E-11 | 1.87E-10 | 15.1387 |
| LMNA | 0.50386 | 5.332641 | 7.82604 | 3.29E-14 | 4.28E-13 | 21.4128 |
| IL4R | 0.50404 | 3.283207 | 7.84992 | 2.78E-14 | 3.65E-13 | 21.5782 |
| AC138207.4 | 0.50416 | 1.22734 | 11.1586 | 7.66E-26 | 3.28E-24 | 47.8278 |
| LOXL4 | 0.50421 | 0.777549 | 7.76072 | 5.20E-14 | 6.63E-13 | 20.9626 |
| DONSON | 0.5043 | 3.621304 | 9.98739 | 1.88E-21 | 5.15E-20 | 37.8369 |
| MREG | 0.50435 | 3.707467 | 7.11404 | 4.17E-12 | 4.27E-11 | 16.6661 |
| PPIE | 0.50437 | 3.940113 | 16.6066 | 3.49E-49 | 1.65E-46 | 101.081 |
| LBX2-AS1 | 0.50438 | 1.364624 | 10.2071 | 2.98E-22 | 8.78E-21 | 39.6579 |
| SNIP1 | 0.50447 | 3.597881 | 14.798 | 4.95E-41 | 1.09E-38 | 82.4677 |
| PDGFB | 0.50482 | 4.334662 | 8.21239 | 2.06E-15 | 3.02E-14 | 24.1345 |
| LRRC23 | 0.50482 | 4.620414 | 9.12504 | 2.04E-18 | 4.14E-17 | 30.9454 |
| ZNF761 | 0.50504 | 2.715029 | 11.3616 | 1.24E-26 | 5.74E-25 | 49.6267 |
| ZNF419 | 0.50511 | 2.639763 | 11.7976 | 2.35E-28 | 1.33E-26 | 53.5528 |
| ISL2 | 0.50516 | 0.49554 | 8.43199 | 4.09E-16 | 6.46E-15 | 25.7254 |
| CCN4 | 0.50524 | 0.877769 | 5.45333 | 7.96E-08 | 4.75E-07 | 7.06621 |
| SLC66A3 | 0.5053 | 3.468591 | 9.52564 | 8.36E-20 | 1.95E-18 | 34.0945 |
| PLEKHO1 | 0.50532 | 4.871158 | 11.7216 | 4.72E-28 | 2.58E-26 | 52.8621 |
| CA13 | 0.50546 | 1.565111 | 9.7485 | 1.36E-20 | 3.43E-19 | 35.886 |
| BRIP1 | 0.50563 | 0.913412 | 7.22129 | 2.05E-12 | 2.19E-11 | 17.3581 |
| RHPN1-AS1 | 0.50602 | 1.316743 | 10.7158 | 3.79E-24 | 1.37E-22 | 43.9705 |
| P2RY6 | 0.50602 | 1.014863 | 8.85871 | 1.62E-17 | 2.97E-16 | 28.9044 |
| OTX1 | 0.50623 | 1.162787 | 5.70615 | 2.03E-08 | 1.31E-07 | 8.38697 |
| GRB14 | 0.50644 | 1.031084 | 5.64101 | 2.90E-08 | 1.84E-07 | 8.04166 |
| SLC35D2 | 0.50651 | 4.570526 | 9.96731 | 2.23E-21 | 6.04E-20 | 37.6717 |
| AC117490.2 | 0.50663 | 1.693325 | 7.86162 | 2.56E-14 | 3.38E-13 | 21.6592 |
| ABCD3 | 0.50668 | 5.002553 | 10.8584 | 1.09E-24 | 4.14E-23 | 45.203 |
| UNC45A | 0.5068 | 4.475745 | 11.8034 | 2.23E-28 | 1.26E-26 | 53.6054 |
| SLC25A19 | 0.50688 | 2.701531 | 12.1036 | 1.38E-29 | 8.86E-28 | 56.3556 |
| ZNRF2 | 0.50715 | 2.983896 | 10.3498 | 8.87E-23 | 2.76E-21 | 40.8548 |
| PPP1R8 | 0.50718 | 5.943617 | 14.8969 | 1.81E-41 | 4.11E-39 | 83.4669 |
| ESM1 | 0.50732 | 0.787114 | 4.81205 | 2.01E-06 | 9.85E-06 | 3.95551 |
| TNFRSF25 | 0.50775 | 2.149792 | 6.99488 | 9.05E-12 | 8.86E-11 | 15.9071 |
| MAP3K6 | 0.50777 | 3.309889 | 7.632 | 1.27E-13 | 1.55E-12 | 20.084 |
| TMEM220 | 0.50784 | 2.282399 | 7.70241 | 7.81E-14 | 9.75E-13 | 20.5632 |
| GALK1 | 0.50798 | 4.576353 | 9.15592 | 1.60E-18 | 3.27E-17 | 31.1849 |
| LINC02611 | 0.50799 | 0.973533 | 12.5398 | 2.29E-31 | 1.77E-29 | 60.415 |
| DNAJC22 | 0.50824 | 1.394118 | 7.6662 | 1.00E-13 | 1.24E-12 | 20.3163 |
| ARSI | 0.50835 | 1.539298 | 6.48068 | 2.29E-10 | 1.88E-09 | 12.7528 |
| AC008537.3 | 0.50839 | 2.230579 | 8.74625 | 3.83E-17 | 6.74E-16 | 28.0556 |
| CYHR1 | 0.50864 | 4.694086 | 12.7778 | 2.37E-32 | 2.01E-30 | 62.6606 |
| FSTL3 | 0.50869 | 3.499098 | 8.26112 | 1.44E-15 | 2.14E-14 | 24.4848 |
| VWA1 | 0.50877 | 4.537256 | 5.08231 | 5.37E-07 | 2.86E-06 | 5.22403 |
| FBXO46 | 0.50888 | 3.839425 | 12.076 | 1.79E-29 | 1.14E-27 | 56.1007 |
| HOXD11 | 0.50892 | 0.316366 | 6.8088 | 2.98E-11 | 2.74E-10 | 14.7427 |
| FLII | 0.50894 | 5.546645 | 14.0193 | 1.27E-37 | 2.00E-35 | 74.6882 |
| MIR5581 | 0.50897 | 2.511876 | 5.07583 | 5.54E-07 | 2.95E-06 | 5.1929 |
| PARS2 | 0.50918 | 3.323709 | 13.4309 | 4.25E-35 | 5.10E-33 | 68.9261 |
| FAM78A | 0.50923 | 3.155197 | 10.4896 | 2.68E-23 | 8.85E-22 | 42.0372 |
| RWDD3 | 0.5096 | 3.253492 | 13.7331 | 2.18E-36 | 2.99E-34 | 71.8719 |
| AC009902.2 | 0.50963 | 1.58666 | 4.4275 | 1.18E-05 | 5.18E-05 | 2.25933 |
| MRGPRF | 0.50969 | 2.214458 | 5.53056 | 5.27E-08 | 3.23E-07 | 7.4641 |
| SMIM4 | 0.5097 | 3.085285 | 13.4988 | 2.19E-35 | 2.68E-33 | 69.5849 |
| EGLN2 | 0.50978 | 3.132843 | 12.477 | 4.15E-31 | 3.13E-29 | 59.8267 |
| SNRPA | 0.51002 | 5.971538 | 14.0239 | 1.21E-37 | 1.92E-35 | 74.7337 |
| RPL13AP20 | 0.51015 | 2.933443 | 8.3063 | 1.04E-15 | 1.57E-14 | 24.811 |
| ATP23 | 0.51026 | 2.921703 | 7.76135 | 5.18E-14 | 6.61E-13 | 20.9669 |
| AC020907.4 | 0.51029 | 1.254767 | 9.98705 | 1.89E-21 | 5.16E-20 | 37.8341 |
| AC127502.2 | 0.51037 | 1.173242 | 9.16895 | 1.44E-18 | 2.96E-17 | 31.286 |
| GSTM4 | 0.51064 | 4.173812 | 12.2005 | 5.60E-30 | 3.72E-28 | 57.2509 |
| COPZ2 | 0.51066 | 4.065443 | 6.45775 | 2.63E-10 | 2.14E-09 | 12.6168 |
| AL445307.1 | 0.51067 | 2.169182 | 7.40785 | 5.89E-13 | 6.67E-12 | 18.5815 |
| AC107959.1 | 0.51082 | 1.556984 | 8.50459 | 2.38E-16 | 3.86E-15 | 26.2581 |
| SLFN12 | 0.51089 | 1.320677 | 8.89331 | 1.24E-17 | 2.31E-16 | 29.1671 |
| RPL28 | 0.51109 | 7.398086 | 10.9075 | 7.07E-25 | 2.75E-23 | 45.6296 |
| RILPL2 | 0.5111 | 3.111813 | 12.3793 | 1.05E-30 | 7.52E-29 | 58.9126 |
| LPAR1 | 0.51115 | 4.827108 | 3.95707 | 8.74E-05 | 0.000332 | 0.36207 |
| AP001025.1 | 0.51123 | 0.950693 | 7.62039 | 1.38E-13 | 1.67E-12 | 20.0053 |
| AMZ2P1 | 0.51138 | 4.320561 | 8.85922 | 1.61E-17 | 2.96E-16 | 28.9083 |
| LINC01094 | 0.51153 | 4.807047 | 4.74061 | 2.82E-06 | 1.35E-05 | 3.63066 |
| TMEM220-AS1 | 0.51165 | 0.989547 | 7.10819 | 4.33E-12 | 4.43E-11 | 16.6286 |
| CCDC106 | 0.51169 | 5.312556 | 11.9736 | 4.63E-29 | 2.81E-27 | 55.1594 |
| AC003070.1 | 0.51169 | 1.299241 | 8.67221 | 6.73E-17 | 1.16E-15 | 27.501 |
| U2AF2 | 0.51181 | 7.006249 | 13.5959 | 8.42E-36 | 1.08E-33 | 70.5309 |
| AL109811.2 | 0.51203 | 4.026548 | 8.64115 | 8.51E-17 | 1.45E-15 | 27.2694 |
| BCAS2 | 0.51224 | 6.335127 | 14.5997 | 3.71E-40 | 7.67E-38 | 80.4716 |
| PDIK1L | 0.51229 | 3.5483 | 11.3444 | 1.45E-26 | 6.63E-25 | 49.4739 |
| MIER1 | 0.51236 | 3.647497 | 12.72 | 4.12E-32 | 3.39E-30 | 62.1139 |
| CDCA7L | 0.51237 | 3.3403 | 4.95048 | 1.03E-06 | 5.26E-06 | 4.59742 |
| PHLDB3 | 0.51237 | 2.403172 | 13.8447 | 7.20E-37 | 1.05E-34 | 72.9676 |
| GINS4 | 0.51237 | 1.352755 | 9.9344 | 2.93E-21 | 7.86E-20 | 37.4015 |
| STMP1 | 0.5124 | 5.43947 | 11.4963 | 3.68E-27 | 1.81E-25 | 50.8307 |
| ITGB8 | 0.51251 | 5.203904 | 5.96972 | 4.65E-09 | 3.27E-08 | 9.81923 |
| DRC1 | 0.51262 | 0.813465 | 4.88775 | 1.40E-06 | 7.01E-06 | 4.30449 |
| AL162724.2 | 0.51267 | 1.41917 | 9.04918 | 3.69E-18 | 7.29E-17 | 30.3598 |
| PAFAH2 | 0.5127 | 3.524716 | 13.9796 | 1.88E-37 | 2.91E-35 | 74.2962 |
| TSPAN12 | 0.51271 | 4.794125 | 5.3839 | 1.15E-07 | 6.68E-07 | 6.7127 |
| CCDC28B | 0.51272 | 4.442278 | 11.6921 | 6.18E-28 | 3.32E-26 | 52.5953 |
| CYP2S1 | 0.51274 | 1.302279 | 10.4834 | 2.83E-23 | 9.31E-22 | 41.9846 |
| MIR155HG | 0.51292 | 0.739714 | 9.97069 | 2.17E-21 | 5.87E-20 | 37.6995 |
| SLC44A3-AS1 | 0.51304 | 1.163521 | 8.06013 | 6.21E-15 | 8.70E-14 | 23.05 |
| FBXL7 | 0.51306 | 4.621926 | 8.37284 | 6.34E-16 | 9.84E-15 | 25.2938 |
| PPP1R15A | 0.51318 | 4.904443 | 7.4846 | 3.50E-13 | 4.05E-12 | 19.092 |
| FLT3LG | 0.51328 | 1.627599 | 13.2375 | 2.81E-34 | 3.12E-32 | 67.0557 |
| MAPKAPK3 | 0.51359 | 4.84919 | 10.0452 | 1.16E-21 | 3.24E-20 | 38.3138 |
| DM1-AS | 0.51359 | 1.339288 | 11.4223 | 7.18E-27 | 3.40E-25 | 50.1687 |
| AC060766.4 | 0.51361 | 1.167844 | 9.33822 | 3.77E-19 | 8.24E-18 | 32.6096 |
| TLE1 | 0.51364 | 5.174179 | 7.29873 | 1.23E-12 | 1.34E-11 | 17.8629 |
| AC060766.1 | 0.51369 | 1.448015 | 8.21819 | 1.97E-15 | 2.90E-14 | 24.1761 |
| PRSS23 | 0.51373 | 2.598888 | 6.10706 | 2.11E-09 | 1.54E-08 | 10.5876 |
| KIAA0355 | 0.51377 | 4.097956 | 10.3133 | 1.21E-22 | 3.72E-21 | 40.5477 |
| SNRPC | 0.5138 | 6.89751 | 14.8943 | 1.86E-41 | 4.21E-39 | 83.4404 |
| ZNF211 | 0.51387 | 3.951417 | 10.3578 | 8.29E-23 | 2.59E-21 | 40.9218 |
| THAP3 | 0.51405 | 3.857533 | 15.4633 | 5.38E-44 | 1.63E-41 | 89.2352 |
| HFE | 0.5141 | 1.481331 | 8.28438 | 1.22E-15 | 1.83E-14 | 24.6526 |
| AC010655.2 | 0.51422 | 0.752014 | 11.9054 | 8.70E-29 | 5.11E-27 | 54.5354 |
| ACTN4 | 0.51424 | 6.747555 | 10.4427 | 4.01E-23 | 1.30E-21 | 41.6393 |
| CPQ | 0.51426 | 3.779405 | 5.94476 | 5.36E-09 | 3.74E-08 | 9.68121 |
| LINC02803 | 0.51438 | 1.414838 | 9.9154 | 3.43E-21 | 9.12E-20 | 37.2457 |
| PIPOX | 0.51438 | 3.971155 | 4.99303 | 8.36E-07 | 4.33E-06 | 4.79804 |
| SLC17A9 | 0.51438 | 1.142602 | 10.4353 | 4.27E-23 | 1.38E-21 | 41.577 |
| AKR1A1 | 0.51448 | 6.144291 | 15.2399 | 5.38E-43 | 1.48E-40 | 86.9513 |
| TMOD3 | 0.5145 | 3.47517 | 10.2882 | 1.50E-22 | 4.56E-21 | 40.3372 |
| AL365330.1 | 0.51461 | 2.252346 | 8.26718 | 1.38E-15 | 2.06E-14 | 24.5285 |
| TM2D1 | 0.51462 | 3.824206 | 15.7897 | 1.82E-45 | 6.10E-43 | 92.5905 |
| CYP27A1 | 0.51472 | 5.00303 | 4.69832 | 3.44E-06 | 1.63E-05 | 3.44045 |
| DPH2 | 0.51498 | 4.627138 | 13.6439 | 5.25E-36 | 6.93E-34 | 70.9995 |
| ATP8B4 | 0.51552 | 1.509909 | 9.10268 | 2.43E-18 | 4.89E-17 | 30.7725 |
| STX11 | 0.51567 | 1.657998 | 7.19015 | 2.52E-12 | 2.66E-11 | 17.1563 |
| MIR4648 | 0.51589 | 1.54022 | 4.83108 | 1.83E-06 | 9.05E-06 | 4.04276 |
| ZNF226 | 0.51589 | 3.334837 | 12.8351 | 1.37E-32 | 1.20E-30 | 63.2048 |
| PHYKPL | 0.51596 | 3.433991 | 10.9502 | 4.86E-25 | 1.92E-23 | 46.001 |
| SHC1 | 0.51601 | 4.699138 | 8.78695 | 2.81E-17 | 5.02E-16 | 28.3619 |
| MTFR1L | 0.51608 | 5.151436 | 17.5916 | 9.95E-54 | 6.91E-51 | 111.461 |
| ZNF776 | 0.51624 | 3.51066 | 10.2238 | 2.59E-22 | 7.67E-21 | 39.7977 |
| LINC00662 | 0.51625 | 2.822861 | 11.533 | 2.64E-27 | 1.32E-25 | 51.1604 |
| CNOT3 | 0.51628 | 4.313185 | 13.3198 | 1.26E-34 | 1.45E-32 | 67.8501 |
| TMBIM1 | 0.51659 | 5.221882 | 6.24543 | 9.38E-10 | 7.14E-09 | 11.3768 |
| NMNAT3 | 0.51659 | 2.197526 | 4.85734 | 1.62E-06 | 8.03E-06 | 4.16368 |
| FAP | 0.51664 | 0.795771 | 9.86151 | 5.36E-21 | 1.40E-19 | 36.805 |
| CD274 | 0.51664 | 1.51949 | 7.59044 | 1.70E-13 | 2.03E-12 | 19.8028 |
| LMO1 | 0.51676 | 2.856148 | 4.61994 | 4.95E-06 | 2.29E-05 | 3.09204 |
| GLB1L | 0.51685 | 3.059023 | 10.8862 | 8.53E-25 | 3.30E-23 | 45.444 |
| TMEM165 | 0.51715 | 4.337806 | 9.7256 | 1.64E-20 | 4.10E-19 | 35.7006 |
| ZWINT | 0.5172 | 3.591578 | 5.37107 | 1.23E-07 | 7.12E-07 | 6.64785 |
| AC234582.1 | 0.51725 | 5.163891 | 7.29529 | 1.26E-12 | 1.37E-11 | 17.8404 |
| STAT1 | 0.5173 | 5.881738 | 6.09035 | 2.32E-09 | 1.69E-08 | 10.4933 |
| HEBP2 | 0.51785 | 2.551866 | 12.3894 | 9.50E-31 | 6.90E-29 | 59.007 |
| RNU4ATAC | 0.51785 | 1.987811 | 6.14556 | 1.69E-09 | 1.25E-08 | 10.8057 |
| GLIPR1 | 0.51787 | 2.999234 | 6.57985 | 1.24E-10 | 1.06E-09 | 13.3456 |
| ZNF599 | 0.51798 | 2.170159 | 15.8429 | 1.05E-45 | 3.64E-43 | 93.1405 |
| PHKG1 | 0.51804 | 5.234142 | 5.0489 | 6.34E-07 | 3.35E-06 | 5.06381 |
| SIX6 | 0.5182 | 0.386386 | 6.4568 | 2.64E-10 | 2.15E-09 | 12.6111 |
| PIEZO1 | 0.51831 | 4.077547 | 8.40676 | 4.93E-16 | 7.72E-15 | 25.541 |
| OCIAD2 | 0.51837 | 2.83867 | 4.14261 | 4.06E-05 | 0.000163 | 1.08674 |
| SNORA73B | 0.51839 | 1.505699 | 5.56331 | 4.42E-08 | 2.74E-07 | 7.63431 |
| SBNO2 | 0.51853 | 3.755338 | 8.89915 | 1.18E-17 | 2.21E-16 | 29.2116 |
| ZNF473 | 0.51855 | 2.586806 | 11.9199 | 7.61E-29 | 4.50E-27 | 54.6678 |
| TUBB4B | 0.51865 | 7.917822 | 9.69958 | 2.03E-20 | 5.02E-19 | 35.4904 |
| TMEM222 | 0.51874 | 5.488412 | 17.4955 | 2.78E-53 | 1.84E-50 | 110.443 |
| TAS1R1 | 0.51906 | 0.723344 | 12.1802 | 6.77E-30 | 4.43E-28 | 57.0625 |
| AL513320.1 | 0.51917 | 2.444145 | 7.73588 | 6.19E-14 | 7.82E-13 | 20.7921 |
| SH3PXD2B | 0.51967 | 4.818952 | 8.22602 | 1.86E-15 | 2.74E-14 | 24.2324 |
| ZNF362 | 0.52005 | 5.666023 | 14.3346 | 5.39E-39 | 1.00E-36 | 77.8188 |
| RAB20 | 0.52007 | 4.350454 | 6.27718 | 7.77E-10 | 5.98E-09 | 11.56 |
| PTTG1IP | 0.52013 | 8.698963 | 9.29711 | 5.23E-19 | 1.12E-17 | 32.2866 |
| SOCS6 | 0.52028 | 4.583408 | 8.29998 | 1.08E-15 | 1.64E-14 | 24.7653 |
| MEX3D | 0.52036 | 4.190403 | 9.59302 | 4.84E-20 | 1.16E-18 | 34.6332 |
| ZFAND6 | 0.52063 | 5.228924 | 13.6257 | 6.27E-36 | 8.21E-34 | 70.8223 |
| ZNF345 | 0.52067 | 1.939558 | 13.4458 | 3.67E-35 | 4.43E-33 | 69.0708 |
| FBXO17 | 0.52071 | 1.949814 | 4.69269 | 3.53E-06 | 1.67E-05 | 3.41525 |
| FRMD8 | 0.52075 | 4.794587 | 11.193 | 5.63E-26 | 2.44E-24 | 48.1318 |
| EPHB4 | 0.52087 | 3.271251 | 8.91001 | 1.09E-17 | 2.05E-16 | 29.2942 |
| CTSW | 0.52087 | 1.044746 | 8.47305 | 3.01E-16 | 4.82E-15 | 26.0263 |
| ZNF783 | 0.5209 | 3.397521 | 10.4328 | 4.36E-23 | 1.41E-21 | 41.5556 |
| COLEC12 | 0.52094 | 3.11141 | 5.46387 | 7.53E-08 | 4.51E-07 | 7.12021 |
| TRIM28 | 0.52101 | 7.456669 | 11.8055 | 2.19E-28 | 1.24E-26 | 53.6238 |
| AMY2B | 0.52108 | 2.114166 | 8.4254 | 4.29E-16 | 6.77E-15 | 25.6772 |
| AC011468.1 | 0.5211 | 2.379544 | 10.4112 | 5.25E-23 | 1.68E-21 | 41.373 |
| FAM234A | 0.52111 | 5.051891 | 11.3819 | 1.03E-26 | 4.82E-25 | 49.8077 |
| TUBB | 0.52115 | 8.781661 | 9.73627 | 1.50E-20 | 3.78E-19 | 35.787 |
| IFITM2 | 0.52128 | 5.388558 | 6.7294 | 4.91E-11 | 4.38E-10 | 14.2537 |
| ADPGK | 0.52134 | 4.155954 | 13.8973 | 4.27E-37 | 6.33E-35 | 73.4854 |
| H2BC5 | 0.5217 | 3.770878 | 5.8337 | 1.00E-08 | 6.74E-08 | 9.0731 |
| STXBP3 | 0.52179 | 4.820233 | 12.0177 | 3.08E-29 | 1.90E-27 | 55.5645 |
| DPY19L1 | 0.52188 | 5.007357 | 6.74423 | 4.47E-11 | 4.02E-10 | 14.3446 |
| AC112184.1 | 0.52194 | 3.360549 | 7.35627 | 8.34E-13 | 9.27E-12 | 18.2408 |
| VIM-AS1 | 0.52203 | 0.789062 | 13.563 | 1.16E-35 | 1.47E-33 | 70.2099 |
| ATP6V0B | 0.52211 | 6.431286 | 14.3735 | 3.65E-39 | 6.96E-37 | 78.2064 |
| AC007786.3 | 0.52232 | 0.905914 | 10.9743 | 3.93E-25 | 1.57E-23 | 46.2116 |
| C1orf112 | 0.52247 | 1.856325 | 11.8821 | 1.08E-28 | 6.27E-27 | 54.3229 |
| CCDC81 | 0.52248 | 0.92594 | 11.5627 | 2.01E-27 | 1.02E-25 | 51.4269 |
| PPIC | 0.52252 | 3.179155 | 6.07977 | 2.47E-09 | 1.79E-08 | 10.4337 |
| CYB561D1 | 0.52262 | 3.350058 | 10.7944 | 1.91E-24 | 7.08E-23 | 44.6485 |
| COL6A1 | 0.52283 | 6.556066 | 6.95896 | 1.14E-11 | 1.11E-10 | 15.6803 |
| DPH5 | 0.52288 | 4.648617 | 13.797 | 1.16E-36 | 1.65E-34 | 72.499 |
| CDT1 | 0.52295 | 2.906918 | 5.6357 | 2.99E-08 | 1.89E-07 | 8.01365 |
| OSBPL3 | 0.52302 | 3.340438 | 6.87098 | 2.01E-11 | 1.88E-10 | 15.1289 |
| IMPA2 | 0.52307 | 1.858079 | 10.0997 | 7.36E-22 | 2.09E-20 | 38.7644 |
| RBM38 | 0.52312 | 5.304992 | 10.0118 | 1.54E-21 | 4.23E-20 | 38.038 |
| ADGRE4P | 0.52316 | 1.08525 | 8.21686 | 1.99E-15 | 2.92E-14 | 24.1666 |
| AC061992.2 | 0.52333 | 0.546254 | 9.50258 | 1.01E-19 | 2.32E-18 | 33.9106 |
| TNNI2 | 0.52363 | 0.944526 | 10.5069 | 2.31E-23 | 7.68E-22 | 42.184 |
| SPRY1 | 0.52375 | 3.086609 | 5.27674 | 2.00E-07 | 1.13E-06 | 6.17501 |
| LRRC66 | 0.5238 | 0.834096 | 10.9524 | 4.77E-25 | 1.89E-23 | 46.02 |
| FAM76A | 0.52392 | 3.482225 | 12.3225 | 1.78E-30 | 1.24E-28 | 58.3832 |
| ENPP1 | 0.52393 | 0.866894 | 10.2426 | 2.21E-22 | 6.58E-21 | 39.9549 |
| RAD54B | 0.52398 | 1.025727 | 12.6898 | 5.50E-32 | 4.43E-30 | 61.8285 |
| SNRPGP10 | 0.52404 | 2.268941 | 5.77274 | 1.41E-08 | 9.29E-08 | 8.74353 |
| BRICD5 | 0.52428 | 3.508766 | 7.11609 | 4.11E-12 | 4.22E-11 | 16.6792 |
| AC092919.2 | 0.52447 | 1.302636 | 8.97509 | 6.57E-18 | 1.27E-16 | 29.7911 |
| RTCA | 0.52451 | 4.645263 | 13.5104 | 1.95E-35 | 2.41E-33 | 69.6987 |
| VKORC1 | 0.52467 | 4.510451 | 12.0946 | 1.50E-29 | 9.60E-28 | 56.2725 |
| ERVK3-1 | 0.52485 | 3.379114 | 11.2857 | 2.46E-26 | 1.10E-24 | 48.9519 |
| ZNF790 | 0.52485 | 2.760778 | 12.874 | 9.43E-33 | 8.48E-31 | 63.5746 |
| SOAT1 | 0.52502 | 4.134765 | 10.202 | 3.11E-22 | 9.14E-21 | 39.6155 |
| SPINK8 | 0.52502 | 2.335905 | 5.35449 | 1.34E-07 | 7.73E-07 | 6.56418 |
| ERI1 | 0.52529 | 3.024104 | 10.9185 | 6.42E-25 | 2.51E-23 | 45.7251 |
| ZNF234 | 0.52536 | 2.739183 | 9.47294 | 1.28E-19 | 2.91E-18 | 33.6749 |
| MORN2 | 0.52553 | 5.422547 | 12.9971 | 2.88E-33 | 2.81E-31 | 64.7485 |
| HOXC9 | 0.52683 | 0.322253 | 7.50643 | 3.02E-13 | 3.52E-12 | 19.2379 |
| AC015912.3 | 0.52691 | 1.302687 | 8.21601 | 2.01E-15 | 2.94E-14 | 24.1605 |
| POLR2I | 0.52704 | 5.912415 | 13.0229 | 2.25E-33 | 2.23E-31 | 64.9945 |
| HOXA1 | 0.52715 | 0.416521 | 9.14978 | 1.68E-18 | 3.43E-17 | 31.1372 |
| H2BC21 | 0.52717 | 5.009784 | 6.6296 | 9.15E-11 | 7.90E-10 | 13.6458 |
| CCDC77 | 0.52717 | 3.129268 | 10.6726 | 5.52E-24 | 1.96E-22 | 43.5994 |
| LAMTOR2 | 0.52727 | 5.995423 | 14.9405 | 1.16E-41 | 2.71E-39 | 83.9083 |
| LENG9 | 0.52771 | 1.537776 | 9.89452 | 4.08E-21 | 1.08E-19 | 37.0748 |
| LRMDA | 0.5279 | 1.407592 | 12.1027 | 1.40E-29 | 8.93E-28 | 56.3467 |
| IGSF3 | 0.52823 | 3.806073 | 4.39337 | 1.38E-05 | 5.97E-05 | 2.11508 |
| WDR3 | 0.52829 | 3.665532 | 10.3543 | 8.54E-23 | 2.66E-21 | 40.8924 |
| SKP2 | 0.52836 | 3.717075 | 10.1366 | 5.40E-22 | 1.55E-20 | 39.0708 |
| RBBP8 | 0.52855 | 3.636841 | 7.94758 | 1.39E-14 | 1.88E-13 | 22.2583 |
| AL139231.1 | 0.5286 | 0.858242 | 4.48009 | 9.35E-06 | 4.16E-05 | 2.48368 |
| GGH | 0.52874 | 3.813639 | 7.92474 | 1.64E-14 | 2.20E-13 | 22.0986 |
| ZNF181 | 0.52901 | 3.525077 | 12.3242 | 1.76E-30 | 1.23E-28 | 58.399 |
| PDGFA | 0.52905 | 4.277021 | 5.36176 | 1.29E-07 | 7.46E-07 | 6.60085 |
| ZNF146 | 0.52915 | 5.87565 | 10.6377 | 7.46E-24 | 2.61E-22 | 43.3006 |
| CDC7 | 0.52923 | 3.490338 | 7.45946 | 4.15E-13 | 4.77E-12 | 18.9243 |
| ENG | 0.52931 | 5.457214 | 8.02662 | 7.90E-15 | 1.09E-13 | 22.8134 |
| AL512329.2 | 0.52931 | 1.594955 | 4.88914 | 1.39E-06 | 6.96E-06 | 4.31096 |
| GNB2 | 0.52946 | 7.950669 | 14.8898 | 1.94E-41 | 4.38E-39 | 83.3956 |
| EFNB1 | 0.52955 | 4.327619 | 10.224 | 2.58E-22 | 7.67E-21 | 39.7995 |
| ZNF69 | 0.52957 | 2.229581 | 11.4149 | 7.68E-27 | 3.61E-25 | 50.1028 |
| CALU | 0.5296 | 6.04556 | 7.6362 | 1.24E-13 | 1.50E-12 | 20.1125 |
| CTHRC1 | 0.52979 | 1.786824 | 5.32381 | 1.57E-07 | 8.98E-07 | 6.41003 |
| PRICKLE3 | 0.52998 | 1.245492 | 9.45329 | 1.50E-19 | 3.40E-18 | 33.5188 |
| AC010331.1 | 0.53005 | 1.399093 | 9.31929 | 4.38E-19 | 9.50E-18 | 32.4607 |
| PPP1R14B | 0.53007 | 6.522739 | 8.3129 | 9.86E-16 | 1.50E-14 | 24.8587 |
| NME2 | 0.53023 | 5.195654 | 10.293 | 1.44E-22 | 4.39E-21 | 40.3773 |
| SEC22B | 0.5303 | 3.669581 | 14.0921 | 6.13E-38 | 1.02E-35 | 75.4088 |
| MXRA5 | 0.53039 | 1.475305 | 5.12432 | 4.35E-07 | 2.35E-06 | 5.42682 |
| AL021707.6 | 0.53043 | 2.16538 | 7.68419 | 8.87E-14 | 1.10E-12 | 20.4388 |
| HK3 | 0.53045 | 1.343163 | 8.21634 | 2.00E-15 | 2.94E-14 | 24.1629 |
| CD3E | 0.53053 | 0.883391 | 7.30135 | 1.21E-12 | 1.32E-11 | 17.88 |
| ZNF593 | 0.53055 | 2.127932 | 14.3547 | 4.41E-39 | 8.28E-37 | 78.0188 |
| AC004852.2 | 0.53055 | 0.705677 | 6.79577 | 3.23E-11 | 2.96E-10 | 14.6621 |
| RPN2 | 0.5306 | 7.785744 | 11.5836 | 1.66E-27 | 8.53E-26 | 51.6151 |
| AL606970.4 | 0.53066 | 0.398877 | 8.21144 | 2.07E-15 | 3.04E-14 | 24.1277 |
| UBA2 | 0.53069 | 6.145879 | 10.0236 | 1.39E-21 | 3.85E-20 | 38.135 |
| HAUS8 | 0.53077 | 1.996368 | 10.9407 | 5.28E-25 | 2.08E-23 | 45.918 |
| IRAK1 | 0.5312 | 6.589532 | 12.3102 | 2.00E-30 | 1.38E-28 | 58.2687 |
| HM13 | 0.5315 | 5.183552 | 13.5592 | 1.21E-35 | 1.51E-33 | 70.1731 |
| RPA2 | 0.53158 | 6.500317 | 15.1069 | 2.11E-42 | 5.28E-40 | 85.5969 |
| NRDC | 0.53164 | 5.690634 | 14.5618 | 5.45E-40 | 1.11E-37 | 80.0909 |
| PAM | 0.53197 | 5.782476 | 8.14047 | 3.48E-15 | 4.98E-14 | 23.6203 |
| ZNF684 | 0.53228 | 2.363511 | 16.2542 | 1.42E-47 | 5.82E-45 | 97.4035 |
| NTN1 | 0.53239 | 5.685424 | 6.2093 | 1.16E-09 | 8.73E-09 | 11.1692 |
| P2RX4 | 0.53243 | 2.829402 | 12.4365 | 6.09E-31 | 4.51E-29 | 59.4473 |
| RPS8 | 0.53246 | 9.31118 | 9.76267 | 1.21E-20 | 3.08E-19 | 36.0009 |
| AC105942.1 | 0.53248 | 5.144296 | 10.9878 | 3.49E-25 | 1.41E-23 | 46.3294 |
| PRMT6 | 0.5326 | 4.445092 | 10.982 | 3.67E-25 | 1.48E-23 | 46.2788 |
| IGHM | 0.53261 | 1.023224 | 4.72432 | 3.04E-06 | 1.45E-05 | 3.5572 |
| FOLR2 | 0.53265 | 5.467694 | 4.48641 | 9.09E-06 | 4.05E-05 | 2.51081 |
| HSPBAP1 | 0.53272 | 2.76557 | 16.5559 | 5.96E-49 | 2.70E-46 | 100.551 |
| LINC02285 | 0.53282 | 0.989437 | 11.2959 | 2.24E-26 | 1.01E-24 | 49.0428 |
| HSPB1P1 | 0.53284 | 2.080716 | 6.74032 | 4.59E-11 | 4.12E-10 | 14.3207 |
| PRMT1 | 0.53341 | 6.019333 | 13.8131 | 9.85E-37 | 1.41E-34 | 72.6574 |
| TGFBR2 | 0.53375 | 4.852704 | 7.41503 | 5.62E-13 | 6.36E-12 | 18.629 |
| ANGPT1 | 0.53388 | 1.64675 | 5.64405 | 2.86E-08 | 1.81E-07 | 8.0577 |
| AP001527.2 | 0.5339 | 0.861431 | 10.2821 | 1.58E-22 | 4.79E-21 | 40.2856 |
| LINC00665 | 0.53407 | 3.380708 | 13.1177 | 8.99E-34 | 9.44E-32 | 65.9031 |
| NETO2 | 0.53422 | 3.364656 | 5.37465 | 1.20E-07 | 7.00E-07 | 6.66591 |
| SPSB2 | 0.53433 | 3.779123 | 10.2053 | 3.02E-22 | 8.89E-21 | 39.6436 |
| SRSF10 | 0.53435 | 4.472654 | 13.5862 | 9.26E-36 | 1.18E-33 | 70.4367 |
| TP53I3 | 0.53442 | 4.097424 | 6.6479 | 8.17E-11 | 7.11E-10 | 13.7567 |
| FAM20A | 0.53446 | 1.143957 | 7.47004 | 3.87E-13 | 4.46E-12 | 18.9948 |
| ODF2 | 0.53449 | 4.298456 | 11.6502 | 9.07E-28 | 4.77E-26 | 52.216 |
| SLC25A10 | 0.53458 | 2.34362 | 11.522 | 2.91E-27 | 1.45E-25 | 51.0611 |
| TRIP13 | 0.53485 | 2.075088 | 7.08175 | 5.15E-12 | 5.22E-11 | 16.4594 |
| CPT2 | 0.53497 | 4.397486 | 13.4892 | 2.40E-35 | 2.93E-33 | 69.4916 |
| NPHP1 | 0.53501 | 1.623196 | 13.1129 | 9.42E-34 | 9.83E-32 | 65.8572 |
| SGO2 | 0.53529 | 1.897193 | 8.01977 | 8.30E-15 | 1.15E-13 | 22.7651 |
| PROB1 | 0.5353 | 2.540841 | 9.27411 | 6.28E-19 | 1.34E-17 | 32.1063 |
| PSMB8 | 0.53546 | 5.753926 | 7.17855 | 2.73E-12 | 2.85E-11 | 17.0813 |
| CFAP52 | 0.53554 | 1.750641 | 5.95316 | 5.11E-09 | 3.57E-08 | 9.7276 |
| CD3D | 0.53556 | 0.820327 | 7.35415 | 8.46E-13 | 9.40E-12 | 18.2268 |
| CDKN2B | 0.53557 | 3.791808 | 4.56539 | 6.36E-06 | 2.90E-05 | 2.85271 |
| EYA4 | 0.53564 | 0.687486 | 6.22216 | 1.08E-09 | 8.13E-09 | 11.243 |
| C1QL4 | 0.53582 | 2.703142 | 3.52936 | 0.000457 | 0.001531 | -1.1895 |
| TCTN1 | 0.5359 | 3.603917 | 10.6833 | 5.02E-24 | 1.80E-22 | 43.6916 |
| PSMA5 | 0.53614 | 4.910823 | 18.2291 | 1.06E-56 | 1.04E-53 | 118.247 |
| HGFAC | 0.53624 | 1.629365 | 7.50308 | 3.09E-13 | 3.59E-12 | 19.2155 |
| PRC1 | 0.53635 | 3.356271 | 5.67179 | 2.46E-08 | 1.57E-07 | 8.20441 |
| MBNL3 | 0.53642 | 1.252422 | 9.13643 | 1.86E-18 | 3.80E-17 | 31.0337 |
| PLOD3 | 0.53659 | 5.132095 | 9.64009 | 3.30E-20 | 7.98E-19 | 35.011 |
| AL645608.7 | 0.53666 | 2.627848 | 4.65984 | 4.11E-06 | 1.93E-05 | 3.26871 |
| CCL8 | 0.53692 | 0.985721 | 5.53781 | 5.07E-08 | 3.11E-07 | 7.50167 |
| PSMB8-AS1 | 0.53695 | 3.351854 | 7.15942 | 3.09E-12 | 3.21E-11 | 16.9579 |
| LINC02777 | 0.53698 | 1.954191 | 8.36912 | 6.51E-16 | 1.01E-14 | 25.2668 |
| SRRM1 | 0.53706 | 4.886058 | 12.3845 | 9.95E-31 | 7.19E-29 | 58.9614 |
| ICMT | 0.53724 | 5.206972 | 13.2138 | 3.54E-34 | 3.89E-32 | 66.8268 |
| RUVBL2 | 0.53733 | 6.050458 | 14.452 | 1.65E-39 | 3.19E-37 | 78.9912 |
| HOXB4 | 0.53742 | 0.371959 | 6.92719 | 1.40E-11 | 1.34E-10 | 15.4805 |
| MOK | 0.53747 | 2.143605 | 10.9321 | 5.70E-25 | 2.23E-23 | 45.8438 |
| FZD1 | 0.5375 | 3.028581 | 8.61916 | 1.00E-16 | 1.70E-15 | 27.1057 |
| NUP85 | 0.53765 | 4.272263 | 13.3806 | 6.96E-35 | 8.15E-33 | 68.4379 |
| AC106865.1 | 0.53765 | 0.69989 | 8.24316 | 1.65E-15 | 2.43E-14 | 24.3555 |
| AL731567.1 | 0.53779 | 0.902884 | 9.93698 | 2.87E-21 | 7.69E-20 | 37.4226 |
| OTP | 0.53781 | 0.316043 | 7.32036 | 1.06E-12 | 1.17E-11 | 18.0046 |
| CRABP2 | 0.53786 | 3.261779 | 6.36341 | 4.64E-10 | 3.67E-09 | 12.0615 |
| CCDC9 | 0.53794 | 3.961299 | 13.7139 | 2.63E-36 | 3.59E-34 | 71.6846 |
| VEGFA | 0.53798 | 3.217954 | 5.61194 | 3.40E-08 | 2.13E-07 | 7.88867 |
| POU4F1 | 0.53802 | 0.648345 | 7.87669 | 2.30E-14 | 3.05E-13 | 21.7639 |
| SLCO2B1 | 0.53805 | 5.515398 | 8.01187 | 8.78E-15 | 1.21E-13 | 22.7094 |
| CSDE1 | 0.53807 | 7.703794 | 12.8446 | 1.25E-32 | 1.10E-30 | 63.2945 |
| EVC2 | 0.53817 | 0.674739 | 8.47868 | 2.89E-16 | 4.64E-15 | 26.0676 |
| TMX1 | 0.5385 | 5.247803 | 10.6656 | 5.86E-24 | 2.08E-22 | 43.5394 |
| ADGRE2 | 0.53855 | 0.986747 | 8.49177 | 2.62E-16 | 4.23E-15 | 26.1638 |
| AC006942.1 | 0.53893 | 2.04275 | 8.7114 | 4.99E-17 | 8.70E-16 | 27.7941 |
| EYA2 | 0.53905 | 3.179494 | 4.84737 | 1.70E-06 | 8.40E-06 | 4.11771 |
| SASS6 | 0.53922 | 2.223504 | 13.185 | 4.68E-34 | 5.05E-32 | 66.5504 |
| MED25 | 0.53926 | 4.663685 | 14.1522 | 3.36E-38 | 5.74E-36 | 76.0044 |
| AC005674.2 | 0.53928 | 1.518031 | 11.2913 | 2.34E-26 | 1.05E-24 | 49.0018 |
| CCDC18-AS1 | 0.5393 | 3.236575 | 8.97772 | 6.44E-18 | 1.24E-16 | 29.8111 |
| SERPINF1 | 0.53937 | 4.196058 | 4.88713 | 1.40E-06 | 7.03E-06 | 4.3016 |
| TLR8 | 0.53948 | 0.892369 | 8.51085 | 2.27E-16 | 3.69E-15 | 26.3043 |
| ZBTB17 | 0.53948 | 4.787904 | 15.1167 | 1.91E-42 | 4.82E-40 | 85.6962 |
| PRRT3-AS1 | 0.53951 | 1.600494 | 11.124 | 1.04E-25 | 4.41E-24 | 47.5233 |
| CPED1 | 0.53997 | 1.465673 | 8.52594 | 2.03E-16 | 3.31E-15 | 26.4155 |
| CREB5 | 0.54017 | 3.569243 | 6.69985 | 5.91E-11 | 5.23E-10 | 14.073 |
| CHODL | 0.54025 | 1.170156 | 6.30235 | 6.69E-10 | 5.18E-09 | 11.7057 |
| AGRN | 0.54037 | 5.940864 | 8.57685 | 1.38E-16 | 2.30E-15 | 26.7918 |
| TNFRSF8 | 0.54055 | 0.790852 | 6.72487 | 5.05E-11 | 4.50E-10 | 14.226 |
| HMMR | 0.54071 | 1.567149 | 6.21859 | 1.10E-09 | 8.29E-09 | 11.2225 |
| NOL9 | 0.54093 | 3.596661 | 14.0971 | 5.83E-38 | 9.74E-36 | 75.4587 |
| LOXL1 | 0.54117 | 1.391924 | 5.64102 | 2.90E-08 | 1.84E-07 | 8.04171 |
| OSR1 | 0.54127 | 0.88451 | 6.46816 | 2.47E-10 | 2.02E-09 | 12.6784 |
| ECT2 | 0.54135 | 2.808463 | 6.60979 | 1.03E-10 | 8.87E-10 | 13.526 |
| LINC00926 | 0.54137 | 1.762448 | 10.0742 | 9.12E-22 | 2.56E-20 | 38.5536 |
| SELENOKP1 | 0.54142 | 2.031526 | 10.8293 | 1.41E-24 | 5.29E-23 | 44.9505 |
| GTF2B | 0.54144 | 5.014619 | 17.2097 | 5.86E-52 | 3.61E-49 | 107.42 |
| NXT1 | 0.54156 | 4.984184 | 11.434 | 6.46E-27 | 3.08E-25 | 50.2734 |
| CDCA5 | 0.54158 | 3.003299 | 5.20492 | 2.89E-07 | 1.60E-06 | 5.82001 |
| DENND2A | 0.54164 | 5.636184 | 7.14034 | 3.51E-12 | 3.63E-11 | 16.835 |
| CACNA1C-AS2 | 0.54167 | 1.814801 | 8.8913 | 1.26E-17 | 2.34E-16 | 29.1519 |
| KIF18B | 0.54199 | 2.641614 | 4.92309 | 1.18E-06 | 5.96E-06 | 4.46911 |
| KTI12 | 0.54203 | 3.863705 | 16.8609 | 2.38E-50 | 1.28E-47 | 103.747 |
| MYO15B | 0.54204 | 2.815258 | 6.93541 | 1.33E-11 | 1.28E-10 | 15.5321 |
| ALDH3B1 | 0.54237 | 2.645878 | 9.07605 | 2.99E-18 | 5.97E-17 | 30.5668 |
| KNTC1 | 0.54243 | 2.097282 | 8.57468 | 1.41E-16 | 2.34E-15 | 26.7757 |
| ANKRD10 | 0.54257 | 5.948719 | 8.86058 | 1.59E-17 | 2.93E-16 | 28.9186 |
| MKRN7P | 0.5426 | 0.966813 | 9.54677 | 7.04E-20 | 1.66E-18 | 34.2631 |
| SRGN | 0.54262 | 6.947666 | 6.39066 | 3.94E-10 | 3.15E-09 | 12.2212 |
| AC246787.1 | 0.54285 | 2.967377 | 9.12188 | 2.09E-18 | 4.24E-17 | 30.921 |
| USF2 | 0.54294 | 6.681359 | 15.3605 | 1.55E-43 | 4.56E-41 | 88.1831 |
| LINC02308 | 0.54301 | 1.322708 | 6.19832 | 1.24E-09 | 9.29E-09 | 11.1064 |
| ERMAP | 0.54302 | 3.583991 | 10.0135 | 1.51E-21 | 4.17E-20 | 38.0522 |
| NOD1 | 0.54307 | 1.886537 | 12.7607 | 2.79E-32 | 2.34E-30 | 62.4993 |
| PSMB2 | 0.54312 | 5.975472 | 18.1366 | 2.88E-56 | 2.72E-53 | 117.26 |
| PRR5L | 0.54321 | 3.276846 | 7.01224 | 8.09E-12 | 7.98E-11 | 16.017 |
| HLA-U | 0.54348 | 1.455683 | 6.81572 | 2.85E-11 | 2.63E-10 | 14.7855 |
| OSCP1 | 0.54402 | 3.501474 | 10.242 | 2.22E-22 | 6.61E-21 | 39.9496 |
| AC084036.1 | 0.54415 | 4.611012 | 7.77667 | 4.65E-14 | 5.96E-13 | 21.0723 |
| SMIM5 | 0.54427 | 1.601131 | 7.61226 | 1.46E-13 | 1.76E-12 | 19.9503 |
| FBN2 | 0.54436 | 1.668097 | 6.87136 | 2.00E-11 | 1.88E-10 | 15.1313 |
| ARSA | 0.54442 | 4.866624 | 9.36714 | 2.99E-19 | 6.62E-18 | 32.8374 |
| TMEM147-AS1 | 0.54442 | 2.762923 | 10.5535 | 1.55E-23 | 5.22E-22 | 42.581 |
| FAM81B | 0.54442 | 0.559461 | 5.75612 | 1.54E-08 | 1.01E-07 | 8.6542 |
| PPP4R1L | 0.54466 | 1.458302 | 14.1965 | 2.16E-38 | 3.75E-36 | 76.4439 |
| KMT5C | 0.54471 | 3.009377 | 10.4748 | 3.04E-23 | 9.95E-22 | 41.9113 |
| FREM2 | 0.5449 | 1.510933 | 5.16347 | 3.57E-07 | 1.95E-06 | 5.6171 |
| EXTL2 | 0.54499 | 4.233421 | 10.6304 | 7.95E-24 | 2.77E-22 | 43.2381 |
| LRRC46 | 0.54515 | 1.524823 | 9.26912 | 6.53E-19 | 1.39E-17 | 32.0673 |
| SYCE1L | 0.54533 | 1.523948 | 8.48159 | 2.82E-16 | 4.54E-15 | 26.089 |
| AGO3 | 0.54562 | 2.6798 | 11.6203 | 1.19E-27 | 6.18E-26 | 51.9465 |
| CFAP53 | 0.54575 | 2.411043 | 8.50758 | 2.33E-16 | 3.77E-15 | 26.2802 |
| CABP4 | 0.54596 | 0.905756 | 11.514 | 3.13E-27 | 1.55E-25 | 50.9897 |
| EVI5 | 0.54617 | 3.725211 | 11.9788 | 4.41E-29 | 2.69E-27 | 55.2076 |
| AKR7A2 | 0.54624 | 6.140241 | 14.8014 | 4.79E-41 | 1.05E-38 | 82.502 |
| AC012254.5 | 0.54653 | 0.957173 | 9.64824 | 3.09E-20 | 7.47E-19 | 35.0766 |
| MYL6 | 0.54665 | 8.160113 | 12.6923 | 5.37E-32 | 4.35E-30 | 61.8523 |
| SYF2 | 0.54686 | 5.391318 | 19.145 | 5.35E-61 | 7.33E-58 | 128.065 |
| OSTC | 0.54695 | 5.598016 | 12.3139 | 1.93E-30 | 1.34E-28 | 58.3039 |
| CHST14 | 0.54708 | 4.3409 | 10.2564 | 1.96E-22 | 5.90E-21 | 40.0706 |
| TKTL1 | 0.54712 | 1.377245 | 5.98156 | 4.35E-09 | 3.07E-08 | 9.88487 |
| REXO2 | 0.54733 | 4.086884 | 13.314 | 1.33E-34 | 1.54E-32 | 67.7936 |
| TENT5B | 0.54733 | 1.205716 | 6.07557 | 2.53E-09 | 1.84E-08 | 10.4101 |
| ELOA | 0.54734 | 4.904501 | 13.8376 | 7.73E-37 | 1.13E-34 | 72.8979 |
| MIR7111 | 0.54751 | 1.279832 | 6.46052 | 2.58E-10 | 2.11E-09 | 12.6331 |
| C1orf54 | 0.54756 | 4.332769 | 8.64921 | 8.01E-17 | 1.36E-15 | 27.3294 |
| C16orf54 | 0.54758 | 1.000683 | 11.3258 | 1.71E-26 | 7.80E-25 | 49.3089 |
| TMEM107 | 0.54776 | 3.612763 | 12.9612 | 4.08E-33 | 3.88E-31 | 64.4051 |
| ATF5 | 0.5479 | 4.464055 | 9.11358 | 2.23E-18 | 4.51E-17 | 30.8567 |
| CKLF | 0.54801 | 3.912982 | 12.0643 | 2.00E-29 | 1.26E-27 | 55.9933 |
| ZNF428 | 0.54813 | 6.869361 | 11.6969 | 5.92E-28 | 3.19E-26 | 52.6384 |
| AC073655.2 | 0.54822 | 1.484464 | 9.32165 | 4.30E-19 | 9.35E-18 | 32.4793 |
| LINC01354 | 0.54829 | 3.062543 | 5.80162 | 1.20E-08 | 7.97E-08 | 8.89927 |
| AL671277.1 | 0.54838 | 2.811736 | 3.8298 | 0.000145 | 0.000531 | -0.117 |
| RDH5 | 0.54854 | 1.270984 | 9.35139 | 3.39E-19 | 7.46E-18 | 32.7133 |
| ZIK1 | 0.54888 | 2.996971 | 10.2997 | 1.36E-22 | 4.16E-21 | 40.4332 |
| MMP19 | 0.5489 | 0.904022 | 6.4059 | 3.60E-10 | 2.88E-09 | 12.3107 |
| HOXA11-AS | 0.54896 | 0.455352 | 7.7094 | 7.44E-14 | 9.31E-13 | 20.6109 |
| APOBEC3F | 0.549 | 1.139716 | 10.2277 | 2.50E-22 | 7.44E-21 | 39.83 |
| C1orf159 | 0.54912 | 2.47017 | 12.5745 | 1.65E-31 | 1.29E-29 | 60.7412 |
| SLC16A1 | 0.54917 | 5.996538 | 9.39967 | 2.30E-19 | 5.15E-18 | 33.0942 |
| IL6 | 0.54941 | 0.877435 | 5.9473 | 5.28E-09 | 3.69E-08 | 9.69523 |
| RNA5SP498 | 0.54949 | 1.242511 | 7.4576 | 4.21E-13 | 4.83E-12 | 18.9119 |
| C1orf158 | 0.54949 | 0.479055 | 6.36624 | 4.57E-10 | 3.61E-09 | 12.078 |
| SH3BP5-AS1 | 0.54959 | 2.398597 | 8.90226 | 1.16E-17 | 2.16E-16 | 29.2353 |
| RFTN1 | 0.5496 | 4.413362 | 6.30974 | 6.40E-10 | 4.97E-09 | 11.7487 |
| ANGPT2 | 0.5496 | 2.281965 | 4.95462 | 1.01E-06 | 5.17E-06 | 4.61687 |
| PEF1 | 0.54966 | 6.564788 | 16.0603 | 1.08E-46 | 4.07E-44 | 95.3897 |
| LATS2 | 0.54974 | 2.18281 | 7.54485 | 2.32E-13 | 2.73E-12 | 19.4956 |
| HES1 | 0.54991 | 4.929703 | 7.06068 | 5.90E-12 | 5.94E-11 | 16.3249 |
| DR1 | 0.55005 | 3.788951 | 13.5628 | 1.17E-35 | 1.47E-33 | 70.2083 |
| OVGP1 | 0.55006 | 2.247565 | 8.58307 | 1.32E-16 | 2.21E-15 | 26.8378 |
| AC243960.1 | 0.55014 | 1.325087 | 11.1358 | 9.38E-26 | 3.99E-24 | 47.6275 |
| GEMIN8P4 | 0.55038 | 2.418603 | 12.8057 | 1.82E-32 | 1.56E-30 | 62.926 |
| RIT1 | 0.5505 | 4.594972 | 14.0359 | 1.07E-37 | 1.72E-35 | 74.8531 |
| NMNAT1 | 0.55062 | 2.905013 | 15.7143 | 4.00E-45 | 1.31E-42 | 91.8134 |
| GEMIN7-AS1 | 0.55062 | 1.884649 | 13.6897 | 3.34E-36 | 4.48E-34 | 71.447 |
| LUM | 0.55063 | 1.896734 | 5.13236 | 4.18E-07 | 2.26E-06 | 5.46576 |
| SWAP70 | 0.55077 | 3.586007 | 7.51874 | 2.77E-13 | 3.25E-12 | 19.3204 |
| PKIB | 0.55079 | 1.923562 | 5.11606 | 4.53E-07 | 2.44E-06 | 5.38679 |
| AC005393.1 | 0.55103 | 2.397518 | 9.80961 | 8.23E-21 | 2.12E-19 | 36.3821 |
| RPS2P5 | 0.55118 | 6.060276 | 6.34104 | 5.31E-10 | 4.16E-09 | 11.9308 |
| SMC5 | 0.55124 | 4.018117 | 10.0115 | 1.54E-21 | 4.24E-20 | 38.0356 |
| P2RY12 | 0.55135 | 6.052385 | 4.28903 | 2.17E-05 | 9.15E-05 | 1.68039 |
| UTP11 | 0.5514 | 4.60543 | 17.9462 | 2.23E-55 | 1.82E-52 | 115.23 |
| AURKAIP1 | 0.55141 | 6.886492 | 14.3545 | 4.41E-39 | 8.28E-37 | 78.0173 |
| PAF1 | 0.55176 | 6.729816 | 15.9766 | 2.60E-46 | 9.45E-44 | 94.522 |
| KLHL17 | 0.55192 | 3.256031 | 9.48409 | 1.17E-19 | 2.67E-18 | 33.7635 |
| AL160006.1 | 0.55211 | 3.310013 | 12.937 | 5.15E-33 | 4.86E-31 | 64.1744 |
| GLRX | 0.55223 | 3.055683 | 9.84316 | 6.24E-21 | 1.62E-19 | 36.6553 |
| TCF19 | 0.55231 | 3.274626 | 7.00317 | 8.58E-12 | 8.43E-11 | 15.9595 |
| OMA1 | 0.55245 | 3.074272 | 16.1544 | 4.05E-47 | 1.56E-44 | 96.3661 |
| PRDM16 | 0.55261 | 2.257091 | 7.28864 | 1.31E-12 | 1.43E-11 | 17.7969 |
| IQCG | 0.55304 | 3.095669 | 9.12867 | 1.98E-18 | 4.03E-17 | 30.9736 |
| PPP1R14B-AS1 | 0.55311 | 1.668227 | 9.41873 | 1.98E-19 | 4.45E-18 | 33.245 |
| MED18 | 0.5533 | 3.930681 | 17.8252 | 8.16E-55 | 6.30E-52 | 113.943 |
| TMTC2 | 0.55335 | 3.338662 | 6.07032 | 2.61E-09 | 1.89E-08 | 10.3806 |
| RPL18 | 0.55345 | 8.245247 | 10.3745 | 7.18E-23 | 2.27E-21 | 41.063 |
| H2BC9 | 0.55374 | 0.428025 | 9.09383 | 2.60E-18 | 5.23E-17 | 30.704 |
| CHRNA9 | 0.55381 | 0.546862 | 6.6323 | 9.00E-11 | 7.78E-10 | 13.6621 |
| VAMP3 | 0.55394 | 6.758503 | 11.4749 | 4.46E-27 | 2.17E-25 | 50.6393 |
| NOSIP | 0.55407 | 4.951773 | 14.0369 | 1.06E-37 | 1.71E-35 | 74.8623 |
| NKG7 | 0.55409 | 1.769232 | 6.60129 | 1.09E-10 | 9.31E-10 | 13.4747 |
| ALG6 | 0.55409 | 2.872595 | 11.4529 | 5.45E-27 | 2.62E-25 | 50.4423 |
| IBSP | 0.5542 | 0.610226 | 4.80328 | 2.09E-06 | 1.02E-05 | 3.91538 |
| H2AC6 | 0.55423 | 5.201933 | 6.3718 | 4.42E-10 | 3.50E-09 | 12.1106 |
| PGAP6 | 0.55432 | 4.909689 | 12.9779 | 3.47E-33 | 3.35E-31 | 64.5651 |
| INTS11 | 0.55433 | 5.13536 | 15.9573 | 3.18E-46 | 1.15E-43 | 94.3226 |
| SDC1 | 0.55448 | 2.163034 | 5.74923 | 1.60E-08 | 1.05E-07 | 8.61723 |
| PIK3CD | 0.55485 | 2.823906 | 8.73389 | 4.21E-17 | 7.39E-16 | 27.9628 |
| GALNT2 | 0.55487 | 5.264163 | 10.8905 | 8.22E-25 | 3.18E-23 | 45.4813 |
| HAUS1 | 0.55512 | 4.178325 | 11.6237 | 1.16E-27 | 6.01E-26 | 51.9765 |
| TENT5C | 0.5552 | 1.282232 | 7.40854 | 5.87E-13 | 6.64E-12 | 18.5861 |
| CD7 | 0.55522 | 0.954298 | 11.0672 | 1.73E-25 | 7.17E-24 | 47.024 |
| DERL3 | 0.55551 | 1.152568 | 10.2116 | 2.87E-22 | 8.47E-21 | 39.6956 |
| APOL2 | 0.55553 | 5.69042 | 8.01311 | 8.70E-15 | 1.20E-13 | 22.7182 |
| PSMC4 | 0.55564 | 6.64638 | 16.7953 | 4.76E-50 | 2.51E-47 | 103.058 |
| THRAP3 | 0.55567 | 6.506071 | 14.4954 | 1.07E-39 | 2.10E-37 | 79.4252 |
| SBSPON | 0.55575 | 1.366757 | 7.42593 | 5.22E-13 | 5.93E-12 | 18.7013 |
| MAP3K19 | 0.55593 | 0.56524 | 6.89666 | 1.70E-11 | 1.62E-10 | 15.2893 |
| MUL1 | 0.55618 | 5.598475 | 15.1001 | 2.26E-42 | 5.63E-40 | 85.528 |
| YIF1B | 0.55628 | 5.279694 | 15.4524 | 6.02E-44 | 1.80E-41 | 89.1231 |
| AL513327.1 | 0.55645 | 1.446394 | 11.9276 | 7.09E-29 | 4.20E-27 | 54.7382 |
| NKX2-5 | 0.55646 | 0.485536 | 6.77005 | 3.80E-11 | 3.45E-10 | 14.5035 |
| BGN | 0.55665 | 6.503714 | 4.9994 | 8.10E-07 | 4.20E-06 | 4.82822 |
| AC009902.3 | 0.55695 | 1.457977 | 4.79108 | 2.22E-06 | 1.08E-05 | 3.85967 |
| DDIAS | 0.557 | 1.295355 | 8.24921 | 1.57E-15 | 2.33E-14 | 24.3991 |
| F11R | 0.55718 | 2.595659 | 8.1271 | 3.83E-15 | 5.46E-14 | 23.5252 |
| AC011899.2 | 0.55723 | 0.884189 | 11.0389 | 2.22E-25 | 9.14E-24 | 46.7766 |
| IL1A | 0.55742 | 0.974264 | 8.31875 | 9.45E-16 | 1.44E-14 | 24.9011 |
| TRABD | 0.55751 | 4.736142 | 10.1219 | 6.11E-22 | 1.75E-20 | 38.949 |
| CDK11B | 0.55758 | 4.986582 | 19.2795 | 1.24E-61 | 1.84E-58 | 129.513 |
| TMEM109 | 0.55787 | 5.85047 | 12.7408 | 3.38E-32 | 2.82E-30 | 62.3107 |
| E2F7 | 0.55788 | 0.877468 | 7.03197 | 7.11E-12 | 7.08E-11 | 16.1422 |
| PLB1 | 0.55829 | 1.510126 | 10.5738 | 1.30E-23 | 4.41E-22 | 42.7539 |
| SNORD104 | 0.55836 | 4.80278 | 5.12231 | 4.39E-07 | 2.37E-06 | 5.41706 |
| FANCC | 0.55838 | 2.205319 | 9.96016 | 2.36E-21 | 6.39E-20 | 37.613 |
| TGFB1I1 | 0.55838 | 3.110305 | 7.73938 | 6.04E-14 | 7.64E-13 | 20.8161 |
| OR7E14P | 0.55861 | 1.171417 | 10.1844 | 3.61E-22 | 1.06E-20 | 39.4689 |
| SH3BP2 | 0.55863 | 4.333143 | 8.68886 | 5.93E-17 | 1.03E-15 | 27.6254 |
| HES4 | 0.55895 | 4.950109 | 5.0599 | 6.00E-07 | 3.18E-06 | 5.11645 |
| GRWD1 | 0.55898 | 3.871843 | 14.1246 | 4.43E-38 | 7.45E-36 | 75.731 |
| RASSF10 | 0.55909 | 0.626946 | 7.65156 | 1.11E-13 | 1.36E-12 | 20.2168 |
| AC005261.1 | 0.55913 | 4.588122 | 10.8575 | 1.10E-24 | 4.17E-23 | 45.1948 |
| DAB2 | 0.55981 | 4.043559 | 7.77078 | 4.85E-14 | 6.20E-13 | 21.0317 |
| H3C10 | 0.55983 | 1.04166 | 8.51791 | 2.15E-16 | 3.50E-15 | 26.3562 |
| AC118549.1 | 0.56006 | 3.767656 | 12.0369 | 2.57E-29 | 1.60E-27 | 55.7413 |
| TRAF3IP3 | 0.56026 | 1.003809 | 12.3741 | 1.10E-30 | 7.87E-29 | 58.8648 |
| BST1 | 0.56029 | 1.5309 | 10.0829 | 8.48E-22 | 2.39E-20 | 38.6253 |
| LILRB2 | 0.5603 | 1.333692 | 9.21195 | 1.03E-18 | 2.14E-17 | 31.6206 |
| SPNS3 | 0.56046 | 1.544603 | 9.39445 | 2.40E-19 | 5.36E-18 | 33.053 |
| GSTM2 | 0.56055 | 4.385032 | 9.38122 | 2.67E-19 | 5.94E-18 | 32.9485 |
| MIB2 | 0.56061 | 4.141831 | 12.3453 | 1.44E-30 | 1.02E-28 | 58.5956 |
| CAMTA1-DT | 0.56072 | 2.521431 | 8.61622 | 1.03E-16 | 1.73E-15 | 27.0838 |
| B3GLCT | 0.56073 | 4.2931 | 8.40975 | 4.82E-16 | 7.57E-15 | 25.5628 |
| RNASEH2A | 0.56079 | 4.832033 | 8.87389 | 1.44E-17 | 2.66E-16 | 29.0196 |
| MTMR11 | 0.56103 | 3.07335 | 7.52219 | 2.71E-13 | 3.17E-12 | 19.3435 |
| PLAAT1 | 0.56146 | 3.598922 | 5.19565 | 3.03E-07 | 1.67E-06 | 5.77452 |
| AL627309.7 | 0.56177 | 1.837651 | 6.79789 | 3.19E-11 | 2.92E-10 | 14.6752 |
| NRAS | 0.56186 | 5.327374 | 11.3175 | 1.85E-26 | 8.36E-25 | 49.2351 |
| POP4 | 0.56193 | 3.524564 | 18.4616 | 8.69E-58 | 9.11E-55 | 120.732 |
| MZF1-AS1 | 0.56194 | 1.911692 | 12.8161 | 1.64E-32 | 1.42E-30 | 63.0243 |
| CAPNS1 | 0.56233 | 7.528689 | 14.9863 | 7.25E-42 | 1.72E-39 | 84.3727 |
| RPS2P32 | 0.56236 | 1.300874 | 7.17365 | 2.82E-12 | 2.94E-11 | 17.0496 |
| POMGNT1 | 0.56236 | 5.632375 | 16.4951 | 1.13E-48 | 4.96E-46 | 99.9147 |
| AKIRIN1 | 0.56236 | 6.499257 | 12.3298 | 1.66E-30 | 1.17E-28 | 58.4519 |
| AC027281.1 | 0.56249 | 0.875744 | 6.02863 | 3.32E-09 | 2.37E-08 | 10.147 |
| ZNF547 | 0.56253 | 2.315151 | 13.5969 | 8.33E-36 | 1.07E-33 | 70.5412 |
| SCMH1 | 0.56257 | 4.206648 | 13.0407 | 1.89E-33 | 1.88E-31 | 65.1654 |
| GALE | 0.56272 | 3.622797 | 16.2576 | 1.37E-47 | 5.66E-45 | 97.4385 |
| CPTP | 0.56283 | 4.939955 | 15.2845 | 3.40E-43 | 9.56E-41 | 87.4067 |
| PLPP3 | 0.56283 | 6.998925 | 7.1202 | 4.00E-12 | 4.11E-11 | 16.7056 |
| KPNA2 | 0.56297 | 5.592686 | 8.2288 | 1.83E-15 | 2.69E-14 | 24.2523 |
| HP1BP3 | 0.56319 | 6.498644 | 12.035 | 2.62E-29 | 1.62E-27 | 55.7239 |
| ZMPSTE24 | 0.56327 | 5.568445 | 12.4915 | 3.62E-31 | 2.74E-29 | 59.9624 |
| AC025176.1 | 0.56347 | 0.61378 | 8.88958 | 1.27E-17 | 2.37E-16 | 29.1388 |
| CNN2 | 0.56349 | 3.755835 | 5.5439 | 4.91E-08 | 3.02E-07 | 7.53333 |
| CDCA4 | 0.56385 | 2.764281 | 9.06259 | 3.32E-18 | 6.60E-17 | 30.4631 |
| ARHGAP27 | 0.56432 | 2.495861 | 11.6652 | 7.90E-28 | 4.20E-26 | 52.352 |
| NUMBL | 0.56447 | 4.605358 | 8.97052 | 6.81E-18 | 1.31E-16 | 29.7561 |
| TRMT13 | 0.56467 | 3.337658 | 11.714 | 5.06E-28 | 2.75E-26 | 52.7934 |
| CHAF1B | 0.56467 | 1.800795 | 7.03132 | 7.15E-12 | 7.11E-11 | 16.138 |
| INMT | 0.56482 | 1.12309 | 6.04593 | 3.01E-09 | 2.16E-08 | 10.2437 |
| FAIM | 0.56486 | 3.495544 | 12.4391 | 5.94E-31 | 4.41E-29 | 59.4717 |
| LINC02712 | 0.56491 | 2.984034 | 6.00137 | 3.88E-09 | 2.76E-08 | 9.99498 |
| HLX | 0.56492 | 1.659898 | 10.3049 | 1.30E-22 | 3.98E-21 | 40.4773 |
| RUNX3 | 0.56494 | 1.505358 | 8.13969 | 3.50E-15 | 5.00E-14 | 23.6148 |
| CYP27B1 | 0.56502 | 0.842117 | 7.16739 | 2.93E-12 | 3.06E-11 | 17.0093 |
| ZNF530 | 0.56505 | 1.640553 | 14.471 | 1.36E-39 | 2.65E-37 | 79.1808 |
| C15orf65 | 0.56528 | 2.360042 | 10.5603 | 1.46E-23 | 4.93E-22 | 42.639 |
| AC022182.2 | 0.56534 | 1.066981 | 10.8569 | 1.10E-24 | 4.19E-23 | 45.1895 |
| MCM8 | 0.56537 | 2.131316 | 9.48582 | 1.15E-19 | 2.64E-18 | 33.7773 |
| PPP1R42 | 0.56542 | 1.387752 | 9.75929 | 1.24E-20 | 3.16E-19 | 35.9735 |
| NFKB2 | 0.56548 | 3.544166 | 10.5847 | 1.18E-23 | 4.03E-22 | 42.8468 |
| CHST2 | 0.56559 | 4.286199 | 7.49607 | 3.24E-13 | 3.76E-12 | 19.1686 |
| CARD8-AS1 | 0.5656 | 1.997043 | 12.7092 | 4.57E-32 | 3.73E-30 | 62.0119 |
| FAM177B | 0.56563 | 0.860926 | 11.1758 | 6.57E-26 | 2.83E-24 | 47.98 |
| NMRAL1 | 0.56571 | 4.488705 | 6.68368 | 6.54E-11 | 5.75E-10 | 13.9743 |
| MX2 | 0.5659 | 1.076251 | 7.70151 | 7.86E-14 | 9.80E-13 | 20.557 |
| SIGLEC1 | 0.56603 | 1.877044 | 5.33241 | 1.50E-07 | 8.62E-07 | 6.45315 |
| ZNF45 | 0.56633 | 3.431457 | 11.4155 | 7.64E-27 | 3.60E-25 | 50.1074 |
| HECTD3 | 0.5664 | 4.938945 | 17.5276 | 1.97E-53 | 1.34E-50 | 110.783 |
| LINC02488 | 0.56643 | 1.21056 | 4.85249 | 1.66E-06 | 8.21E-06 | 4.14131 |
| AC089999.1 | 0.56657 | 0.90039 | 4.88206 | 1.44E-06 | 7.19E-06 | 4.27809 |
| BICRA | 0.56663 | 3.00711 | 13.4671 | 2.98E-35 | 3.60E-33 | 69.2769 |
| MXRA8 | 0.56671 | 4.47983 | 7.95747 | 1.30E-14 | 1.76E-13 | 22.3274 |
| RNU5B-2P | 0.56676 | 2.422338 | 7.99443 | 9.95E-15 | 1.36E-13 | 22.5868 |
| RFX2 | 0.56679 | 3.38142 | 8.13346 | 3.66E-15 | 5.23E-14 | 23.5704 |
| AC098851.1 | 0.56715 | 0.986434 | 10.1422 | 5.15E-22 | 1.48E-20 | 39.1173 |
| CDK6-AS1 | 0.56726 | 0.532783 | 7.29773 | 1.24E-12 | 1.35E-11 | 17.8563 |
| LINC02019 | 0.56734 | 1.491321 | 12.2074 | 5.25E-30 | 3.50E-28 | 57.315 |
| PRPF38A | 0.56758 | 5.471493 | 12.3621 | 1.23E-30 | 8.76E-29 | 58.7523 |
| GNG12-AS1 | 0.56772 | 1.585042 | 12.0721 | 1.86E-29 | 1.17E-27 | 56.0653 |
| AL109659.2 | 0.56786 | 0.871491 | 9.98972 | 1.85E-21 | 5.05E-20 | 37.8561 |
| POLR2J | 0.56793 | 6.681993 | 13.8468 | 7.05E-37 | 1.03E-34 | 72.9881 |
| GINS1 | 0.56795 | 2.847124 | 7.16169 | 3.05E-12 | 3.17E-11 | 16.9725 |
| NEIL3 | 0.56811 | 0.917318 | 7.53544 | 2.47E-13 | 2.91E-12 | 19.4324 |
| C19orf54 | 0.56826 | 4.155574 | 13.4148 | 4.98E-35 | 5.93E-33 | 68.7699 |
| ZNF367 | 0.56904 | 2.660244 | 7.14369 | 3.43E-12 | 3.55E-11 | 16.8565 |
| C1orf109 | 0.56907 | 3.875475 | 12.5698 | 1.72E-31 | 1.35E-29 | 60.6972 |
| AC112484.3 | 0.56919 | 2.622683 | 8.96142 | 7.31E-18 | 1.40E-16 | 29.6865 |
| CNPY1 | 0.56961 | 0.912374 | 7.79385 | 4.12E-14 | 5.32E-13 | 21.1906 |
| ZDHHC18 | 0.56968 | 3.689427 | 15.8625 | 8.55E-46 | 3.00E-43 | 93.3426 |
| RNFT1 | 0.5697 | 2.969169 | 11.7917 | 2.48E-28 | 1.39E-26 | 53.4986 |
| TNXA | 0.56971 | 0.869282 | 9.24835 | 7.70E-19 | 1.62E-17 | 31.9048 |
| NBPF8 | 0.56985 | 1.785853 | 10.6088 | 9.59E-24 | 3.31E-22 | 43.053 |
| ZNF816 | 0.57007 | 1.839223 | 12.1786 | 6.88E-30 | 4.49E-28 | 57.0478 |
| CROCCP2 | 0.57012 | 4.259115 | 10.7636 | 2.50E-24 | 9.13E-23 | 44.3824 |
| HSPG2 | 0.57019 | 2.370424 | 5.50117 | 6.17E-08 | 3.74E-07 | 7.31209 |
| AC007344.1 | 0.57029 | 0.62686 | 8.98116 | 6.27E-18 | 1.21E-16 | 29.8375 |
| PTP4A2 | 0.57036 | 6.578119 | 13.0526 | 1.69E-33 | 1.70E-31 | 65.2792 |
| FRMD3 | 0.57049 | 3.373917 | 7.03656 | 6.91E-12 | 6.88E-11 | 16.1714 |
| GLI3 | 0.57101 | 2.462131 | 7.66303 | 1.03E-13 | 1.26E-12 | 20.2947 |
| MAP3K1 | 0.57104 | 3.338883 | 8.07234 | 5.69E-15 | 7.98E-14 | 23.1364 |
| NFKBID | 0.57119 | 2.223786 | 7.7918 | 4.18E-14 | 5.39E-13 | 21.1764 |
| ZNF837 | 0.57122 | 2.063902 | 11.0669 | 1.73E-25 | 7.18E-24 | 47.0218 |
| GSX2 | 0.57124 | 0.727149 | 6.35891 | 4.77E-10 | 3.77E-09 | 12.0352 |
| MED8 | 0.57128 | 4.681007 | 18.6983 | 6.74E-59 | 7.87E-56 | 123.267 |
| PPP1R35 | 0.57131 | 4.736365 | 11.425 | 7.01E-27 | 3.33E-25 | 50.1929 |
| CCDC57 | 0.57141 | 2.709393 | 11.0854 | 1.47E-25 | 6.15E-24 | 47.1845 |
| POC1A | 0.57154 | 2.674527 | 7.4011 | 6.17E-13 | 6.97E-12 | 18.5368 |
| LINC01909 | 0.57165 | 1.360446 | 11.3029 | 2.11E-26 | 9.49E-25 | 49.1047 |
| PHF11 | 0.5719 | 3.074278 | 8.47095 | 3.06E-16 | 4.89E-15 | 26.0109 |
| HLA-E | 0.57212 | 8.6926 | 8.35142 | 7.42E-16 | 1.14E-14 | 25.1381 |
| TTF2 | 0.5722 | 1.910391 | 11.4527 | 5.46E-27 | 2.62E-25 | 50.4408 |
| INPPL1 | 0.57224 | 6.135938 | 11.6604 | 8.26E-28 | 4.36E-26 | 52.3081 |
| CFAP43 | 0.57225 | 1.158227 | 8.42762 | 4.22E-16 | 6.67E-15 | 25.6934 |
| NES | 0.57229 | 8.163706 | 5.42531 | 9.23E-08 | 5.45E-07 | 6.92308 |
| HS3ST3B1 | 0.57264 | 0.922992 | 5.92736 | 5.92E-09 | 4.10E-08 | 9.58529 |
| MAP2K3 | 0.57266 | 3.706803 | 10.5119 | 2.21E-23 | 7.37E-22 | 42.2268 |
| TNF | 0.57315 | 2.140038 | 4.42452 | 1.20E-05 | 5.25E-05 | 2.2467 |
| MRPL37 | 0.57323 | 5.788355 | 16.2146 | 2.16E-47 | 8.51E-45 | 96.9912 |
| H2BP1 | 0.57327 | 1.290107 | 10.087 | 8.19E-22 | 2.31E-20 | 38.6595 |
| CRYBG1 | 0.57346 | 1.031655 | 8.6208 | 9.93E-17 | 1.68E-15 | 27.1179 |
| ETV6 | 0.5735 | 3.548154 | 11.3557 | 1.31E-26 | 6.02E-25 | 49.5744 |
| CDK4 | 0.57362 | 6.24616 | 6.68634 | 6.43E-11 | 5.66E-10 | 13.9905 |
| IGLC3 | 0.5737 | 0.917224 | 4.54642 | 6.93E-06 | 3.14E-05 | 2.77008 |
| OXER1 | 0.57419 | 1.342392 | 10.8235 | 1.48E-24 | 5.55E-23 | 44.9008 |
| AC015922.2 | 0.57426 | 3.114716 | 5.72436 | 1.84E-08 | 1.19E-07 | 8.48412 |
| HOMER3 | 0.57446 | 5.207044 | 10.5931 | 1.10E-23 | 3.77E-22 | 42.9184 |
| RPS2 | 0.57469 | 9.127467 | 9.85917 | 5.46E-21 | 1.43E-19 | 36.7859 |
| PVT1 | 0.57474 | 0.95535 | 10.1202 | 6.20E-22 | 1.77E-20 | 38.9345 |
| AC125807.2 | 0.57481 | 1.103347 | 7.6861 | 8.75E-14 | 1.08E-12 | 20.4519 |
| RNU4-62P | 0.57482 | 1.171547 | 6.82746 | 2.64E-11 | 2.45E-10 | 14.8583 |
| SPINT1 | 0.57483 | 1.206703 | 11.2415 | 3.65E-26 | 1.61E-24 | 48.5604 |
| CD109 | 0.57486 | 1.929563 | 7.32529 | 1.03E-12 | 1.13E-11 | 18.037 |
| ACADM | 0.57496 | 5.083655 | 12.8586 | 1.09E-32 | 9.72E-31 | 63.4279 |
| SDHAF1 | 0.57502 | 5.016005 | 14.7815 | 5.86E-41 | 1.28E-38 | 82.3009 |
| PHYH | 0.57545 | 4.328509 | 9.12929 | 1.97E-18 | 4.01E-17 | 30.9784 |
| GPR37 | 0.57569 | 5.261388 | 3.98501 | 7.81E-05 | 0.000299 | 0.46921 |
| LRRC41 | 0.57572 | 5.012121 | 15.4442 | 6.55E-44 | 1.95E-41 | 89.0396 |
| STK38L | 0.57609 | 4.64579 | 9.53954 | 7.47E-20 | 1.75E-18 | 34.2053 |
| DSN1 | 0.57634 | 3.631657 | 9.63372 | 3.48E-20 | 8.39E-19 | 34.9598 |
| U2AF1L4 | 0.57649 | 3.467322 | 13.0706 | 1.42E-33 | 1.44E-31 | 65.4518 |
| LINC01235 | 0.57679 | 2.261159 | 4.45706 | 1.04E-05 | 4.58E-05 | 2.38512 |
| RAP1A | 0.57737 | 5.318426 | 15.3084 | 2.66E-43 | 7.63E-41 | 87.6504 |
| RASSF9 | 0.57738 | 0.617412 | 6.72445 | 5.07E-11 | 4.52E-10 | 14.2234 |
| PPM1M | 0.57743 | 3.906998 | 11.1961 | 5.48E-26 | 2.38E-24 | 48.1589 |
| HMGB1P1 | 0.5775 | 2.01352 | 7.44916 | 4.46E-13 | 5.11E-12 | 18.8557 |
| MCM3 | 0.5776 | 5.674403 | 11.391 | 9.53E-27 | 4.45E-25 | 49.8887 |
| AL450998.1 | 0.57794 | 2.077951 | 5.93219 | 5.76E-09 | 4.00E-08 | 9.61187 |
| SPACA6 | 0.57848 | 2.651653 | 9.77016 | 1.14E-20 | 2.90E-19 | 36.0616 |
| HMGN2P17 | 0.57888 | 2.104044 | 10.4384 | 4.16E-23 | 1.34E-21 | 41.6029 |
| IGHA1 | 0.57888 | 1.652801 | 3.52603 | 0.000463 | 0.001548 | -1.2009 |
| ATAD3B | 0.57901 | 2.826561 | 9.32793 | 4.09E-19 | 8.91E-18 | 32.5286 |
| MSLN | 0.57904 | 1.250972 | 6.93311 | 1.35E-11 | 1.30E-10 | 15.5177 |
| FANCI | 0.57918 | 2.663886 | 6.42824 | 3.14E-10 | 2.53E-09 | 12.4423 |
| CMTM6 | 0.57947 | 4.906582 | 10.3637 | 7.88E-23 | 2.48E-21 | 40.972 |
| NOC2L | 0.57974 | 5.420261 | 15.0375 | 4.30E-42 | 1.04E-39 | 84.8916 |
| FGF20 | 0.57999 | 1.204523 | 10.5133 | 2.19E-23 | 7.29E-22 | 42.2382 |
| CFAP45 | 0.5801 | 0.574917 | 6.92578 | 1.41E-11 | 1.35E-10 | 15.4717 |
| RABAC1 | 0.58011 | 6.988932 | 13.5496 | 1.33E-35 | 1.66E-33 | 70.08 |
| KLHL6 | 0.58022 | 1.467901 | 8.94523 | 8.29E-18 | 1.58E-16 | 29.5628 |
| DMAP1 | 0.58024 | 5.03946 | 16.7564 | 7.18E-50 | 3.64E-47 | 102.65 |
| FBXO42 | 0.58051 | 3.781386 | 14.7722 | 6.44E-41 | 1.40E-38 | 82.207 |
| ZNF28 | 0.58056 | 2.723099 | 10.949 | 4.91E-25 | 1.94E-23 | 45.9908 |
| COTL1 | 0.58072 | 6.125786 | 8.90936 | 1.09E-17 | 2.06E-16 | 29.2893 |
| SPATA17 | 0.58089 | 0.936015 | 9.64935 | 3.06E-20 | 7.41E-19 | 35.0855 |
| AC103810.3 | 0.58115 | 2.378384 | 5.01709 | 7.42E-07 | 3.87E-06 | 4.9122 |
| RIBC1 | 0.5816 | 1.960686 | 7.84437 | 2.89E-14 | 3.79E-13 | 21.5397 |
| RER1 | 0.58175 | 5.043935 | 19.1271 | 6.50E-61 | 8.68E-58 | 127.872 |
| KDELR2 | 0.58185 | 6.45647 | 11.97 | 4.79E-29 | 2.89E-27 | 55.1268 |
| LYG1 | 0.58198 | 1.693182 | 11.1603 | 7.54E-26 | 3.23E-24 | 47.843 |
| ZNF787 | 0.58243 | 4.227675 | 14.074 | 7.35E-38 | 1.20E-35 | 75.2293 |
| IL12RB1 | 0.58244 | 1.450497 | 11.1392 | 9.10E-26 | 3.87E-24 | 47.6575 |
| HOXA3 | 0.58279 | 0.376104 | 7.0922 | 4.81E-12 | 4.89E-11 | 16.5262 |
| AC008735.2 | 0.58281 | 2.743969 | 7.39005 | 6.65E-13 | 7.48E-12 | 18.4637 |
| ROMO1 | 0.58298 | 7.365683 | 13.0923 | 1.15E-33 | 1.18E-31 | 65.6593 |
| NFIL3 | 0.58311 | 4.959314 | 9.06346 | 3.30E-18 | 6.56E-17 | 30.4698 |
| NAMPT | 0.58331 | 4.456867 | 6.62272 | 9.55E-11 | 8.23E-10 | 13.6042 |
| HACD4 | 0.5837 | 1.413682 | 10.8447 | 1.23E-24 | 4.64E-23 | 45.0845 |
| NPEPL1 | 0.58371 | 2.647814 | 8.11104 | 4.30E-15 | 6.10E-14 | 23.4109 |
| RRAGC | 0.58403 | 4.085909 | 16.5315 | 7.71E-49 | 3.44E-46 | 100.295 |
| MYO9B | 0.58432 | 5.342057 | 10.6263 | 8.24E-24 | 2.86E-22 | 43.2024 |
| LTBP4 | 0.58433 | 4.791843 | 5.60501 | 3.53E-08 | 2.21E-07 | 7.85231 |
| SCX | 0.58444 | 2.283292 | 6.2843 | 7.45E-10 | 5.74E-09 | 11.6012 |
| TSEN34 | 0.58448 | 5.070096 | 14.9761 | 8.05E-42 | 1.89E-39 | 84.2688 |
| ARHGAP11A | 0.58468 | 2.04483 | 6.70831 | 5.60E-11 | 4.98E-10 | 14.1246 |
| SMO | 0.58502 | 4.720463 | 8.41096 | 4.78E-16 | 7.50E-15 | 25.5717 |
| SSU72 | 0.58512 | 5.42925 | 19.0299 | 1.86E-60 | 2.43E-57 | 126.827 |
| BRCA1 | 0.58533 | 2.310533 | 10.1378 | 5.35E-22 | 1.54E-20 | 39.0807 |
| NUCB1 | 0.58537 | 7.195598 | 14.1669 | 2.90E-38 | 4.99E-36 | 76.1499 |
| GLA | 0.58549 | 4.207822 | 12.8967 | 7.59E-33 | 6.98E-31 | 63.7907 |
| PARP10 | 0.58552 | 4.250151 | 8.09828 | 4.72E-15 | 6.66E-14 | 23.3203 |
| SMS | 0.58553 | 5.968631 | 10.6434 | 7.11E-24 | 2.49E-22 | 43.349 |
| RFC2 | 0.58559 | 5.197461 | 11.9221 | 7.46E-29 | 4.41E-27 | 54.6885 |
| ZNF580 | 0.58574 | 4.792277 | 13.2328 | 2.94E-34 | 3.26E-32 | 67.0103 |
| SNORD14E | 0.58578 | 2.492081 | 4.89732 | 1.33E-06 | 6.71E-06 | 4.34899 |
| NEDD1 | 0.58578 | 3.599146 | 9.96058 | 2.36E-21 | 6.37E-20 | 37.6164 |
| PTBP1 | 0.58579 | 5.928092 | 10.9751 | 3.90E-25 | 1.56E-23 | 46.2182 |
| ZNF577 | 0.58607 | 2.661987 | 11.513 | 3.16E-27 | 1.57E-25 | 50.981 |
| AL513477.1 | 0.58609 | 1.934912 | 10.592 | 1.11E-23 | 3.80E-22 | 42.9095 |
| PIK3CG | 0.58612 | 1.224474 | 8.86922 | 1.49E-17 | 2.75E-16 | 28.9842 |
| SIGLEC11 | 0.58626 | 1.266814 | 10.03 | 1.32E-21 | 3.66E-20 | 38.1878 |
| LAMC1 | 0.5863 | 4.215442 | 6.73628 | 4.70E-11 | 4.22E-10 | 14.2959 |
| PRPS2 | 0.58631 | 3.300092 | 5.91657 | 6.29E-09 | 4.35E-08 | 9.52588 |
| GEMIN7 | 0.58635 | 3.842703 | 16.5679 | 5.25E-49 | 2.40E-46 | 100.676 |
| DDX60L | 0.58641 | 2.005822 | 9.3098 | 4.72E-19 | 1.02E-17 | 32.3862 |
| RTP4 | 0.58651 | 2.935047 | 7.62886 | 1.30E-13 | 1.58E-12 | 20.0627 |
| BTN2A3P | 0.5866 | 1.090226 | 13.5736 | 1.05E-35 | 1.33E-33 | 70.3139 |
| VANGL1 | 0.58663 | 2.03005 | 9.36252 | 3.10E-19 | 6.86E-18 | 32.801 |
| AL359504.1 | 0.58671 | 2.452825 | 9.72302 | 1.68E-20 | 4.19E-19 | 35.6798 |
| LPXN | 0.58706 | 4.186038 | 12.8226 | 1.54E-32 | 1.34E-30 | 63.0863 |
| CC2D1B | 0.58715 | 3.942832 | 16.215 | 2.15E-47 | 8.51E-45 | 96.9954 |
| IGLC2 | 0.58718 | 1.219419 | 4.01408 | 6.93E-05 | 0.000268 | 0.58143 |
| DPEP1 | 0.5872 | 1.047969 | 5.2386 | 2.43E-07 | 1.36E-06 | 5.98596 |
| RBBP4 | 0.58721 | 5.858106 | 14.0412 | 1.02E-37 | 1.64E-35 | 74.9054 |
| CEP112 | 0.58734 | 1.684372 | 10.0895 | 8.02E-22 | 2.27E-20 | 38.6802 |
| EMB | 0.58743 | 2.061025 | 7.92567 | 1.62E-14 | 2.18E-13 | 22.1051 |
| TMEM54 | 0.58765 | 5.999683 | 10.814 | 1.61E-24 | 6.01E-23 | 44.818 |
| CLEC11A | 0.58767 | 3.700205 | 10.1581 | 4.50E-22 | 1.31E-20 | 39.2499 |
| JOSD2 | 0.58774 | 5.095997 | 10.6657 | 5.85E-24 | 2.07E-22 | 43.5408 |
| YTHDF2 | 0.58787 | 5.517824 | 15.2165 | 6.85E-43 | 1.88E-40 | 86.7122 |
| SIMC1 | 0.5879 | 1.77976 | 7.44114 | 4.70E-13 | 5.37E-12 | 18.8024 |
| ZNF865 | 0.58794 | 4.066691 | 12.4494 | 5.39E-31 | 4.01E-29 | 59.5682 |
| CD151 | 0.58811 | 6.000616 | 8.82755 | 2.06E-17 | 3.73E-16 | 28.6685 |
| PIK3CD-AS2 | 0.5882 | 0.773784 | 10.5236 | 2.00E-23 | 6.70E-22 | 42.3258 |
| CTTNBP2NL | 0.58842 | 3.886659 | 10.6024 | 1.01E-23 | 3.49E-22 | 42.998 |
| TOE1 | 0.58844 | 3.66039 | 18.1006 | 4.24E-56 | 3.94E-53 | 116.876 |
| MCM5 | 0.58847 | 4.03472 | 10.0715 | 9.33E-22 | 2.61E-20 | 38.5311 |
| ACSM5 | 0.58862 | 3.275022 | 5.88235 | 7.63E-09 | 5.22E-08 | 9.33824 |
| SLC34A2 | 0.58866 | 0.529507 | 8.52047 | 2.11E-16 | 3.44E-15 | 26.3751 |
| FKBP10 | 0.58868 | 5.804477 | 6.24844 | 9.21E-10 | 7.02E-09 | 11.3941 |
| GPN2 | 0.5887 | 4.465544 | 18.3929 | 1.82E-57 | 1.85E-54 | 119.997 |
| PAQR7 | 0.58912 | 3.960984 | 11.9989 | 3.66E-29 | 2.24E-27 | 55.3922 |
| SFT2D2 | 0.58945 | 4.249114 | 11.6971 | 5.91E-28 | 3.19E-26 | 52.64 |
| MGME1 | 0.58973 | 4.327572 | 12.5168 | 2.85E-31 | 2.19E-29 | 60.1994 |
| LHFPL6 | 0.58975 | 6.494352 | 8.296 | 1.12E-15 | 1.68E-14 | 24.7365 |
| NPM1P25 | 0.59001 | 1.280785 | 10.5031 | 2.39E-23 | 7.94E-22 | 42.1515 |
| RUNX2 | 0.59003 | 1.368528 | 11.0129 | 2.79E-25 | 1.14E-23 | 46.5488 |
| SLC47A2 | 0.59022 | 1.243954 | 5.34975 | 1.37E-07 | 7.91E-07 | 6.54034 |
| CCDC89 | 0.59026 | 1.659654 | 7.36608 | 7.81E-13 | 8.71E-12 | 18.3054 |
| PALM3 | 0.59058 | 1.890778 | 6.62046 | 9.68E-11 | 8.34E-10 | 13.5905 |
| PPP4C | 0.59058 | 6.12594 | 16.2048 | 2.39E-47 | 9.36E-45 | 96.8896 |
| HOXA13 | 0.5906 | 0.681732 | 6.67638 | 6.84E-11 | 6.00E-10 | 13.9298 |
| NDE1 | 0.59065 | 3.308669 | 9.24956 | 7.62E-19 | 1.61E-17 | 31.9143 |
| HAAO | 0.59089 | 1.948025 | 11.0138 | 2.77E-25 | 1.13E-23 | 46.5562 |
| AP001350.2 | 0.59099 | 2.2698 | 8.61078 | 1.07E-16 | 1.80E-15 | 27.0435 |
| AL627309.6 | 0.59109 | 1.716604 | 7.95749 | 1.30E-14 | 1.76E-13 | 22.3276 |
| PLA2G5 | 0.59114 | 2.227034 | 4.23369 | 2.76E-05 | 0.000114 | 1.45377 |
| AC110995.1 | 0.59129 | 1.819799 | 8.98264 | 6.20E-18 | 1.20E-16 | 29.8489 |
| TRIM45 | 0.59141 | 2.553367 | 11.3083 | 2.01E-26 | 9.05E-25 | 49.153 |
| TM4SF1 | 0.59149 | 3.691036 | 5.56609 | 4.36E-08 | 2.70E-07 | 7.64877 |
| AC233728.1 | 0.59156 | 1.778116 | 9.13099 | 1.94E-18 | 3.96E-17 | 30.9916 |
| F13A1 | 0.59157 | 2.34988 | 4.39832 | 1.35E-05 | 5.85E-05 | 2.13595 |
| RRAS | 0.59171 | 5.242489 | 10.9482 | 4.94E-25 | 1.95E-23 | 45.984 |
| CDK11A | 0.59178 | 2.234282 | 13.0061 | 2.64E-33 | 2.59E-31 | 64.8344 |
| ID4 | 0.59181 | 8.761365 | 6.21347 | 1.13E-09 | 8.53E-09 | 11.1931 |
| PTOV1-AS1 | 0.59237 | 3.308165 | 15.4167 | 8.71E-44 | 2.57E-41 | 88.7575 |
| ATG4C | 0.59256 | 4.286298 | 12.1051 | 1.36E-29 | 8.75E-28 | 56.3691 |
| CD63 | 0.59263 | 8.763172 | 10.7889 | 2.00E-24 | 7.40E-23 | 44.6009 |
| GLI2 | 0.59264 | 2.0734 | 8.40691 | 4.92E-16 | 7.72E-15 | 25.5421 |
| SMG9 | 0.59273 | 4.111695 | 15.327 | 2.20E-43 | 6.37E-41 | 87.8403 |
| IL10RB | 0.59273 | 4.459892 | 12.9717 | 3.69E-33 | 3.53E-31 | 64.5057 |
| LDHAP4 | 0.593 | 1.679394 | 9.17532 | 1.37E-18 | 2.82E-17 | 31.3355 |
| TPST1 | 0.59301 | 5.752938 | 9.18229 | 1.30E-18 | 2.68E-17 | 31.3898 |
| CDCA3 | 0.59314 | 2.127872 | 8.41464 | 4.65E-16 | 7.31E-15 | 25.5985 |
| TICRR | 0.5932 | 1.488986 | 10.3219 | 1.13E-22 | 3.47E-21 | 40.6198 |
| SIPA1L3 | 0.59324 | 3.461086 | 11.5087 | 3.28E-27 | 1.62E-25 | 50.9426 |
| GBP1P1 | 0.59378 | 1.329102 | 6.61784 | 9.84E-11 | 8.46E-10 | 13.5747 |
| IKBIP | 0.59382 | 2.676219 | 9.43295 | 1.76E-19 | 3.99E-18 | 33.3576 |
| MAP3K14 | 0.59402 | 3.456007 | 11.5915 | 1.55E-27 | 7.95E-26 | 51.6865 |
| PNKP | 0.5941 | 3.884362 | 13.612 | 7.19E-36 | 9.33E-34 | 70.6878 |
| KNL1 | 0.59423 | 1.189545 | 7.1158 | 4.12E-12 | 4.22E-11 | 16.6774 |
| CYTOR | 0.59425 | 1.235258 | 7.30806 | 1.15E-12 | 1.26E-11 | 17.924 |
| HHLA3 | 0.59443 | 4.949762 | 13.7542 | 1.76E-36 | 2.46E-34 | 72.0791 |
| ZBTB48 | 0.59444 | 3.786839 | 16.7601 | 6.90E-50 | 3.56E-47 | 102.689 |
| IFITM3P6 | 0.59488 | 1.034734 | 11.5492 | 2.28E-27 | 1.15E-25 | 51.3057 |
| KDM4A | 0.59548 | 4.989873 | 12.0327 | 2.68E-29 | 1.66E-27 | 55.702 |
| MAP10 | 0.59552 | 2.302468 | 8.36481 | 6.72E-16 | 1.04E-14 | 25.2354 |
| LINC02328 | 0.59556 | 1.516992 | 7.8485 | 2.81E-14 | 3.68E-13 | 21.5683 |
| CD99P1 | 0.59568 | 1.007783 | 14.6067 | 3.46E-40 | 7.24E-38 | 80.5414 |
| PLEKHA8P1 | 0.59585 | 1.673887 | 13.201 | 4.01E-34 | 4.37E-32 | 66.7038 |
| MAN2B1 | 0.59587 | 4.778668 | 9.84162 | 6.32E-21 | 1.64E-19 | 36.6427 |
| IFITM3 | 0.5959 | 7.706486 | 6.12846 | 1.86E-09 | 1.37E-08 | 10.7087 |
| EPB41 | 0.59598 | 3.589749 | 8.03583 | 7.40E-15 | 1.03E-13 | 22.8783 |
| CYBRD1 | 0.59607 | 5.390282 | 7.02324 | 7.53E-12 | 7.46E-11 | 16.0867 |
| DAPP1 | 0.59633 | 0.954073 | 11.2718 | 2.78E-26 | 1.24E-24 | 48.8288 |
| SOD3 | 0.59633 | 2.894886 | 5.04661 | 6.41E-07 | 3.38E-06 | 5.05288 |
| GRN | 0.59658 | 7.011392 | 10.6939 | 4.58E-24 | 1.64E-22 | 43.7826 |
| RNU6-510P | 0.59667 | 2.648982 | 8.88991 | 1.27E-17 | 2.37E-16 | 29.1413 |
| VMO1 | 0.59674 | 3.40815 | 10.5023 | 2.40E-23 | 7.98E-22 | 42.1449 |
| RNVU1-30 | 0.59675 | 1.781496 | 7.63346 | 1.26E-13 | 1.53E-12 | 20.0939 |
| DISP1 | 0.59677 | 3.202434 | 11.0771 | 1.58E-25 | 6.60E-24 | 47.1114 |
| EHD2 | 0.59684 | 4.945764 | 7.84618 | 2.85E-14 | 3.74E-13 | 21.5522 |
| GUSB | 0.59694 | 4.783109 | 11.6433 | 9.66E-28 | 5.06E-26 | 52.1536 |
| PLCG2 | 0.59701 | 1.799749 | 11.3177 | 1.84E-26 | 8.35E-25 | 49.2369 |
| C8orf34-AS1 | 0.59706 | 0.908943 | 6.68615 | 6.44E-11 | 5.66E-10 | 13.9893 |
| F2R | 0.59712 | 5.422002 | 6.17877 | 1.39E-09 | 1.04E-08 | 10.9947 |
| MCM10 | 0.59713 | 1.323006 | 6.51164 | 1.89E-10 | 1.57E-09 | 12.9371 |
| USP1 | 0.59717 | 5.021592 | 11.9537 | 5.57E-29 | 3.35E-27 | 54.977 |
| ZNF581 | 0.59721 | 4.405961 | 12.3233 | 1.77E-30 | 1.24E-28 | 58.3906 |
| LINC01116 | 0.59732 | 1.634585 | 5.42377 | 9.30E-08 | 5.49E-07 | 6.91523 |
| TEKT1 | 0.59744 | 1.203122 | 5.45042 | 8.08E-08 | 4.82E-07 | 7.0513 |
| IFNGR2 | 0.59749 | 5.586358 | 12.0313 | 2.71E-29 | 1.68E-27 | 55.6899 |
| TBC1D1 | 0.59789 | 3.759733 | 9.56334 | 6.16E-20 | 1.46E-18 | 34.3956 |
| SERBP1 | 0.5982 | 6.325803 | 14.8134 | 4.23E-41 | 9.36E-39 | 82.6236 |
| RAD9A | 0.59841 | 3.488496 | 14.3576 | 4.28E-39 | 8.09E-37 | 78.0479 |
| BX284668.4 | 0.59843 | 2.248839 | 6.46684 | 2.49E-10 | 2.03E-09 | 12.6706 |
| ECE1 | 0.5985 | 5.271176 | 9.16665 | 1.47E-18 | 3.01E-17 | 31.2682 |
| PABPC1L | 0.5986 | 2.85166 | 6.07201 | 2.59E-09 | 1.87E-08 | 10.3901 |
| PGM5 | 0.5991 | 2.45074 | 8.81075 | 2.34E-17 | 4.21E-16 | 28.5415 |
| TIMELESS | 0.59947 | 3.590061 | 7.4673 | 3.94E-13 | 4.54E-12 | 18.9766 |
| TMEM39B | 0.59984 | 4.036901 | 17.7652 | 1.55E-54 | 1.16E-51 | 113.305 |
| DTX2 | 0.60028 | 2.46184 | 15.213 | 7.10E-43 | 1.93E-40 | 86.677 |
| MRTO4 | 0.60033 | 5.557153 | 15.2995 | 2.92E-43 | 8.24E-41 | 87.5591 |
| ANTXR2 | 0.60086 | 2.382492 | 8.57336 | 1.42E-16 | 2.36E-15 | 26.7659 |
| PTPRF | 0.6009 | 5.709562 | 9.07016 | 3.13E-18 | 6.24E-17 | 30.5214 |
| ADM | 0.60113 | 2.49617 | 4.93228 | 1.13E-06 | 5.71E-06 | 4.51211 |
| RIPK3 | 0.60145 | 1.30268 | 12.7796 | 2.33E-32 | 1.98E-30 | 62.6779 |
| RHPN1 | 0.60145 | 4.960464 | 7.17906 | 2.72E-12 | 2.85E-11 | 17.0846 |
| LENG8 | 0.6017 | 6.157387 | 7.49324 | 3.30E-13 | 3.83E-12 | 19.1497 |
| HCP5 | 0.60173 | 2.769105 | 6.73325 | 4.79E-11 | 4.29E-10 | 14.2773 |
| CZIB | 0.6019 | 5.119063 | 20.4555 | 3.43E-67 | 8.55E-64 | 142.208 |
| CRYBB1 | 0.60193 | 2.797385 | 7.48317 | 3.54E-13 | 4.09E-12 | 19.0824 |
| RNF19B | 0.60193 | 4.769131 | 12.0831 | 1.68E-29 | 1.07E-27 | 56.166 |
| AC137630.3 | 0.60195 | 1.096008 | 10.3485 | 8.97E-23 | 2.79E-21 | 40.8438 |
| HOXA2 | 0.60233 | 0.372707 | 6.86028 | 2.15E-11 | 2.01E-10 | 15.0623 |
| BTG3 | 0.60252 | 5.084021 | 10.4356 | 4.26E-23 | 1.38E-21 | 41.5789 |
| FANCD2 | 0.60262 | 1.863117 | 8.65868 | 7.45E-17 | 1.27E-15 | 27.4 |
| FBLIM1 | 0.60278 | 2.228987 | 7.19503 | 2.44E-12 | 2.58E-11 | 17.1879 |
| LRIG3 | 0.60294 | 2.289231 | 9.10382 | 2.41E-18 | 4.85E-17 | 30.7813 |
| AL591895.1 | 0.60299 | 4.749916 | 7.41927 | 5.46E-13 | 6.19E-12 | 18.6572 |
| KIF15 | 0.60303 | 2.34256 | 6.9794 | 1.00E-11 | 9.75E-11 | 15.8093 |
| LMCD1 | 0.60376 | 2.517907 | 9.39326 | 2.43E-19 | 5.41E-18 | 33.0436 |
| CDCP1 | 0.60379 | 1.263138 | 9.88965 | 4.25E-21 | 1.12E-19 | 37.035 |
| AL136380.1 | 0.60389 | 1.663508 | 10.5837 | 1.19E-23 | 4.06E-22 | 42.8389 |
| MND1 | 0.6039 | 1.590292 | 7.88703 | 2.14E-14 | 2.85E-13 | 21.8358 |
| EHBP1L1 | 0.604 | 3.584772 | 11.0622 | 1.80E-25 | 7.48E-24 | 46.9802 |
| TMEM167B | 0.60424 | 5.338038 | 14.028 | 1.16E-37 | 1.85E-35 | 74.7745 |
| CHEK1 | 0.60429 | 2.364265 | 9.37998 | 2.70E-19 | 6.00E-18 | 32.9387 |
| ARHGEF10L | 0.6043 | 5.554971 | 9.2939 | 5.36E-19 | 1.15E-17 | 32.2614 |
| DTL | 0.60447 | 1.994989 | 5.65004 | 2.77E-08 | 1.75E-07 | 8.0893 |
| AKT2 | 0.60485 | 4.516348 | 14.2447 | 1.33E-38 | 2.36E-36 | 76.9237 |
| HMGN2P3 | 0.60492 | 2.209879 | 9.32096 | 4.32E-19 | 9.39E-18 | 32.4739 |
| SLC44A3 | 0.60512 | 3.21046 | 7.02832 | 7.29E-12 | 7.24E-11 | 16.119 |
| RMST | 0.60521 | 1.407891 | 7.43071 | 5.05E-13 | 5.75E-12 | 18.7331 |
| AC003072.1 | 0.60529 | 3.876758 | 9.96655 | 2.24E-21 | 6.07E-20 | 37.6655 |
| SEL1L3 | 0.60536 | 2.4282 | 4.83593 | 1.79E-06 | 8.85E-06 | 4.06507 |
| AC090559.1 | 0.60559 | 2.765572 | 7.12987 | 3.75E-12 | 3.87E-11 | 16.7677 |
| CDKN2A | 0.60577 | 2.822909 | 5.19977 | 2.97E-07 | 1.64E-06 | 5.79474 |
| KCTD12 | 0.60598 | 6.218193 | 7.37717 | 7.25E-13 | 8.12E-12 | 18.3786 |
| CH25H | 0.6061 | 4.061245 | 3.99366 | 7.54E-05 | 0.000289 | 0.50253 |
| HOXB-AS1 | 0.60618 | 0.548285 | 8.58635 | 1.29E-16 | 2.16E-15 | 26.8622 |
| SERPINB6 | 0.60637 | 5.830343 | 10.2956 | 1.41E-22 | 4.30E-21 | 40.3994 |
| ACAA2 | 0.60685 | 3.909663 | 8.21116 | 2.08E-15 | 3.04E-14 | 24.1257 |
| ANKRD53 | 0.60687 | 1.163758 | 10.7198 | 3.66E-24 | 1.32E-22 | 44.0049 |
| CXCL11 | 0.60688 | 0.882529 | 6.64568 | 8.28E-11 | 7.20E-10 | 13.7433 |
| NUDT19 | 0.60713 | 3.396236 | 14.9795 | 7.77E-42 | 1.84E-39 | 84.3039 |
| HLA-F | 0.60718 | 3.937702 | 7.50959 | 2.95E-13 | 3.44E-12 | 19.2591 |
| LFNG | 0.60728 | 6.181942 | 5.55753 | 4.56E-08 | 2.82E-07 | 7.6042 |
| THBS3 | 0.60733 | 3.798345 | 9.5394 | 7.48E-20 | 1.75E-18 | 34.2043 |
| AC012073.1 | 0.60733 | 1.717554 | 11.4469 | 5.75E-27 | 2.75E-25 | 50.3888 |
| NEK11 | 0.60736 | 3.446208 | 8.758 | 3.50E-17 | 6.20E-16 | 28.1439 |
| UBALD2 | 0.60742 | 6.278616 | 11.0139 | 2.77E-25 | 1.13E-23 | 46.5578 |
| CEP135 | 0.60751 | 1.726993 | 11.4715 | 4.60E-27 | 2.23E-25 | 50.6084 |
| PTGES3P1 | 0.60761 | 4.233253 | 8.28184 | 1.24E-15 | 1.86E-14 | 24.6343 |
| NCF1B | 0.60777 | 1.012134 | 12.7928 | 2.06E-32 | 1.76E-30 | 62.803 |
| PINLYP | 0.60816 | 1.49026 | 6.51352 | 1.87E-10 | 1.55E-09 | 12.9482 |
| CXCL16 | 0.60842 | 6.064917 | 8.75875 | 3.48E-17 | 6.16E-16 | 28.1495 |
| PTGFRN | 0.60847 | 4.429092 | 6.36965 | 4.47E-10 | 3.54E-09 | 12.098 |
| PNRC2 | 0.60848 | 5.635649 | 14.9223 | 1.39E-41 | 3.21E-39 | 83.7246 |
| DTX3L | 0.609 | 4.112368 | 8.03819 | 7.27E-15 | 1.01E-13 | 22.895 |
| GDPD2 | 0.60901 | 3.568236 | 5.83193 | 1.01E-08 | 6.80E-08 | 9.06349 |
| C1orf174 | 0.60903 | 3.496495 | 17.335 | 1.54E-52 | 9.82E-50 | 108.744 |
| LMNB2 | 0.60936 | 5.082778 | 10.3972 | 5.92E-23 | 1.88E-21 | 41.2548 |
| SAT1 | 0.60952 | 7.538879 | 10.769 | 2.38E-24 | 8.73E-23 | 44.4294 |
| HEBP1 | 0.60956 | 4.069758 | 7.51562 | 2.83E-13 | 3.31E-12 | 19.2995 |
| BCL10 | 0.60968 | 3.542468 | 16.6867 | 1.50E-49 | 7.31E-47 | 101.919 |
| WARS2 | 0.60974 | 3.083049 | 13.7348 | 2.14E-36 | 2.94E-34 | 71.8889 |
| PRR15 | 0.60995 | 0.96848 | 11.9722 | 4.69E-29 | 2.84E-27 | 55.1467 |
| PCNA | 0.6101 | 6.781405 | 10.1814 | 3.70E-22 | 1.08E-20 | 39.4441 |
| ARAP3 | 0.61019 | 2.911708 | 7.48573 | 3.47E-13 | 4.02E-12 | 19.0995 |
| AL391244.2 | 0.61031 | 2.780593 | 13.6895 | 3.35E-36 | 4.48E-34 | 71.4455 |
| SKA3 | 0.61051 | 1.790446 | 6.23709 | 9.85E-10 | 7.47E-09 | 11.3288 |
| C19orf44 | 0.61053 | 3.069461 | 13.9346 | 2.95E-37 | 4.45E-35 | 73.8525 |
| TWSG1 | 0.61105 | 4.779488 | 9.51265 | 9.28E-20 | 2.15E-18 | 33.9909 |
| ADAMTSL4 | 0.6112 | 1.435514 | 9.31949 | 4.37E-19 | 9.49E-18 | 32.4623 |
| DTYMK | 0.6116 | 4.903739 | 11.721 | 4.75E-28 | 2.59E-26 | 52.8568 |
| FNDC3B | 0.61172 | 2.506928 | 10.3646 | 7.82E-23 | 2.46E-21 | 40.9795 |
| MYZAP | 0.61187 | 0.878083 | 8.34859 | 7.58E-16 | 1.17E-14 | 25.1175 |
| ITGB5 | 0.61209 | 4.973301 | 7.98238 | 1.08E-14 | 1.48E-13 | 22.5021 |
| WARS2-AS1 | 0.61214 | 2.096151 | 15.9566 | 3.20E-46 | 1.15E-43 | 94.3155 |
| PGD | 0.61226 | 6.982394 | 15.0023 | 6.16E-42 | 1.47E-39 | 84.5344 |
| TCTN2 | 0.61245 | 3.378031 | 10.949 | 4.91E-25 | 1.94E-23 | 45.9905 |
| AC243962.1 | 0.61273 | 1.508359 | 8.749 | 3.75E-17 | 6.61E-16 | 28.0762 |
| TP53INP1 | 0.61278 | 4.266985 | 10.0387 | 1.23E-21 | 3.41E-20 | 38.2596 |
| H2AC8 | 0.61283 | 2.303618 | 7.2962 | 1.25E-12 | 1.36E-11 | 17.8463 |
| CYP4F12 | 0.61295 | 0.923669 | 6.95622 | 1.16E-11 | 1.12E-10 | 15.663 |
| IGFBP7 | 0.61315 | 8.528315 | 7.92845 | 1.59E-14 | 2.14E-13 | 22.1245 |
| PIWIL4 | 0.61319 | 1.374128 | 9.81624 | 7.79E-21 | 2.01E-19 | 36.4361 |
| ITGB2-AS1 | 0.61342 | 1.323578 | 10.1083 | 6.85E-22 | 1.95E-20 | 38.8361 |
| GCK | 0.61363 | 1.391129 | 7.96259 | 1.25E-14 | 1.70E-13 | 22.3633 |
| GPX1P1 | 0.61372 | 2.29359 | 5.35123 | 1.36E-07 | 7.86E-07 | 6.54775 |
| KLHL4 | 0.61389 | 2.832094 | 5.66454 | 2.56E-08 | 1.63E-07 | 8.16598 |
| C1orf122 | 0.61396 | 6.292503 | 13.1914 | 4.40E-34 | 4.77E-32 | 66.6113 |
| LPIN3 | 0.61409 | 1.227457 | 8.12255 | 3.96E-15 | 5.64E-14 | 23.4928 |
| NLRC4 | 0.61416 | 1.583785 | 12.452 | 5.26E-31 | 3.92E-29 | 59.5923 |
| AC008750.1 | 0.61478 | 1.197622 | 10.1598 | 4.44E-22 | 1.29E-20 | 39.2639 |
| ELN | 0.6148 | 5.094504 | 6.38348 | 4.12E-10 | 3.28E-09 | 12.179 |
| MIS18BP1 | 0.6149 | 2.437928 | 9.27154 | 6.40E-19 | 1.36E-17 | 32.0862 |
| PHETA2 | 0.61503 | 2.052878 | 10.7901 | 1.98E-24 | 7.33E-23 | 44.6116 |
| AL122010.1 | 0.61512 | 2.537407 | 15.1396 | 1.51E-42 | 3.88E-40 | 85.9292 |
| MGST2 | 0.61517 | 3.526984 | 11.9491 | 5.81E-29 | 3.48E-27 | 54.9355 |
| TP73-AS1 | 0.61527 | 2.546811 | 6.59136 | 1.16E-10 | 9.87E-10 | 13.4148 |
| UROD | 0.6153 | 6.264732 | 19.6886 | 1.46E-63 | 2.58E-60 | 133.921 |
| FUT4 | 0.61546 | 1.716476 | 10.8152 | 1.59E-24 | 5.95E-23 | 44.8282 |
| FUCA1 | 0.61554 | 4.606944 | 10.5355 | 1.81E-23 | 6.06E-22 | 42.4273 |
| TGFBR1 | 0.6157 | 4.704927 | 9.54001 | 7.44E-20 | 1.75E-18 | 34.2091 |
| MRPS12 | 0.61591 | 4.566237 | 15.1702 | 1.10E-42 | 2.92E-40 | 86.2413 |
| OR52K3P | 0.61614 | 0.663388 | 11.8041 | 2.21E-28 | 1.25E-26 | 53.6112 |
| PTGER4 | 0.61625 | 1.383646 | 8.53351 | 1.91E-16 | 3.14E-15 | 26.4713 |
| MOB1A | 0.6163 | 5.202636 | 12.9677 | 3.83E-33 | 3.66E-31 | 64.4677 |
| AC092718.4 | 0.61636 | 3.185686 | 8.55612 | 1.62E-16 | 2.67E-15 | 26.6383 |
| CD40 | 0.61638 | 2.55329 | 10.6318 | 7.86E-24 | 2.74E-22 | 43.2498 |
| CAP1 | 0.61654 | 7.457514 | 14.167 | 2.90E-38 | 4.99E-36 | 76.1513 |
| CNTRL | 0.61667 | 2.870196 | 11.7931 | 2.45E-28 | 1.38E-26 | 53.5118 |
| DDAH2 | 0.61678 | 6.724182 | 11.4511 | 5.54E-27 | 2.65E-25 | 50.4262 |
| MIR4435-2HG | 0.61683 | 1.195232 | 8.13137 | 3.71E-15 | 5.30E-14 | 23.5555 |
| HAS2-AS1 | 0.61722 | 2.079634 | 9.41382 | 2.06E-19 | 4.62E-18 | 33.2061 |
| ADGRE1 | 0.61725 | 0.71276 | 8.29067 | 1.16E-15 | 1.75E-14 | 24.698 |
| ZYX | 0.61732 | 6.832735 | 9.42984 | 1.81E-19 | 4.08E-18 | 33.3329 |
| DAP | 0.61757 | 6.190629 | 14.475 | 1.31E-39 | 2.56E-37 | 79.2209 |
| MX1 | 0.61758 | 3.704997 | 5.93221 | 5.76E-09 | 4.00E-08 | 9.61196 |
| SALL1 | 0.6176 | 5.654758 | 7.80144 | 3.91E-14 | 5.05E-13 | 21.2429 |
| RINL | 0.61772 | 1.960075 | 10.3891 | 6.34E-23 | 2.01E-21 | 41.186 |
| IL18BP | 0.61786 | 3.636484 | 10.1554 | 4.61E-22 | 1.34E-20 | 39.227 |
| SNRNP70 | 0.61864 | 7.174122 | 8.8958 | 1.21E-17 | 2.27E-16 | 29.1861 |
| AL645608.1 | 0.61894 | 1.75262 | 6.87539 | 1.95E-11 | 1.84E-10 | 15.1564 |
| PSMB9 | 0.61921 | 4.233944 | 7.43424 | 4.93E-13 | 5.62E-12 | 18.7565 |
| DEPDC1B | 0.61927 | 1.314785 | 7.88198 | 2.22E-14 | 2.95E-13 | 21.8007 |
| SNORD46 | 0.6194 | 1.55931 | 7.87642 | 2.30E-14 | 3.05E-13 | 21.762 |
| CISH | 0.61978 | 1.014345 | 11.4913 | 3.85E-27 | 1.88E-25 | 50.7861 |
| TREM1 | 0.6198 | 0.662218 | 8.73129 | 4.29E-17 | 7.53E-16 | 27.9433 |
| HLA-DQB1-AS1 | 0.61981 | 1.074919 | 7.73638 | 6.17E-14 | 7.79E-13 | 20.7956 |
| A2M | 0.61981 | 8.117309 | 8.60879 | 1.09E-16 | 1.83E-15 | 27.0286 |
| ZNF474 | 0.6202 | 0.641237 | 9.74134 | 1.44E-20 | 3.63E-19 | 35.828 |
| HSD17B3 | 0.6205 | 1.44765 | 8.39906 | 5.22E-16 | 8.16E-15 | 25.4848 |
| PLA2G4A | 0.62077 | 3.018167 | 7.44684 | 4.53E-13 | 5.18E-12 | 18.8403 |
| TTC25 | 0.62104 | 2.655886 | 11.0837 | 1.49E-25 | 6.24E-24 | 47.169 |
| PALLD | 0.62121 | 4.842532 | 10.4579 | 3.52E-23 | 1.14E-21 | 41.7685 |
| BCAT2 | 0.62133 | 3.528562 | 13.0823 | 1.27E-33 | 1.30E-31 | 65.5641 |
| FAAP24 | 0.62152 | 1.698821 | 13.5062 | 2.03E-35 | 2.50E-33 | 69.6571 |
| CCDC8 | 0.62168 | 1.842252 | 5.73197 | 1.76E-08 | 1.15E-07 | 8.52477 |
| B4GALT1 | 0.62184 | 3.886514 | 9.6668 | 2.65E-20 | 6.47E-19 | 35.226 |
| AL096840.1 | 0.62186 | 1.663376 | 7.56043 | 2.08E-13 | 2.47E-12 | 19.6004 |
| FBXW9 | 0.62192 | 4.058099 | 13.7393 | 2.05E-36 | 2.82E-34 | 71.9329 |
| PBXIP1 | 0.62193 | 7.891308 | 8.06393 | 6.04E-15 | 8.47E-14 | 23.0769 |
| TMEM69 | 0.62198 | 4.70026 | 17.5982 | 9.28E-54 | 6.52E-51 | 111.531 |
| LIG1 | 0.62199 | 3.648605 | 11.0006 | 3.11E-25 | 1.26E-23 | 46.4409 |
| LGALS1 | 0.62205 | 7.750475 | 6.73949 | 4.61E-11 | 4.14E-10 | 14.3156 |
| TGIF2 | 0.62212 | 3.634607 | 9.12802 | 1.99E-18 | 4.05E-17 | 30.9685 |
| AC138207.8 | 0.62226 | 2.181764 | 7.85529 | 2.68E-14 | 3.52E-13 | 21.6153 |
| HYI | 0.62226 | 3.658788 | 14.1625 | 3.03E-38 | 5.20E-36 | 76.1063 |
| TNFRSF14 | 0.62239 | 2.723589 | 9.29335 | 5.39E-19 | 1.16E-17 | 32.2571 |
| SIX1 | 0.62251 | 2.228279 | 5.64579 | 2.83E-08 | 1.79E-07 | 8.06685 |
| GATM | 0.62286 | 6.287011 | 5.91572 | 6.32E-09 | 4.37E-08 | 9.52124 |
| PEPD | 0.62291 | 6.076528 | 15.2125 | 7.14E-43 | 1.93E-40 | 86.6718 |
| HNRNPCP1 | 0.62295 | 1.647066 | 12.5548 | 1.99E-31 | 1.55E-29 | 60.5559 |
| ZNF217 | 0.62309 | 2.865004 | 8.90796 | 1.11E-17 | 2.08E-16 | 29.2786 |
| ZNF529-AS1 | 0.62358 | 2.425081 | 15.3262 | 2.22E-43 | 6.39E-41 | 87.8317 |
| CAPN5 | 0.62368 | 4.835605 | 7.38984 | 6.66E-13 | 7.49E-12 | 18.4623 |
| FAH | 0.62378 | 2.676306 | 10.8577 | 1.10E-24 | 4.17E-23 | 45.1964 |
| ERCC1 | 0.62403 | 5.13405 | 17.3468 | 1.36E-52 | 8.76E-50 | 108.869 |
| HOXA6 | 0.62406 | 0.370708 | 7.83688 | 3.05E-14 | 3.98E-13 | 21.4878 |
| TMSB4XP4 | 0.62414 | 1.889044 | 11.4613 | 5.05E-27 | 2.44E-25 | 50.5174 |
| CHST6 | 0.62445 | 2.750614 | 5.92657 | 5.94E-09 | 4.12E-08 | 9.58092 |
| STK32B | 0.62449 | 2.232163 | 5.99137 | 4.11E-09 | 2.91E-08 | 9.93934 |
| SNAI1 | 0.62462 | 1.390275 | 8.42604 | 4.27E-16 | 6.74E-15 | 25.6818 |
| GSAP | 0.62472 | 1.968418 | 8.78115 | 2.93E-17 | 5.24E-16 | 28.3182 |
| EYA3 | 0.62472 | 3.459639 | 15.5531 | 2.13E-44 | 6.70E-42 | 90.1557 |
| FSCN1 | 0.62494 | 7.424786 | 8.43732 | 3.93E-16 | 6.22E-15 | 25.7644 |
| LRGUK | 0.62554 | 0.738928 | 12.2679 | 2.98E-30 | 2.03E-28 | 57.8754 |
| B3GALT6 | 0.62563 | 5.067983 | 14.0097 | 1.40E-37 | 2.18E-35 | 74.5934 |
| IQCC | 0.62586 | 2.841958 | 17.2194 | 5.28E-52 | 3.29E-49 | 107.523 |
| PRDX6 | 0.62603 | 7.660008 | 9.52618 | 8.32E-20 | 1.94E-18 | 34.0988 |
| TWIST1 | 0.62607 | 1.597133 | 7.14202 | 3.47E-12 | 3.59E-11 | 16.8458 |
| MICALL2 | 0.62611 | 3.305351 | 7.51166 | 2.91E-13 | 3.40E-12 | 19.2729 |
| MOB3C | 0.62616 | 3.269688 | 13.873 | 5.44E-37 | 8.01E-35 | 73.2462 |
| MTF2 | 0.62647 | 3.403192 | 14.2048 | 1.98E-38 | 3.48E-36 | 76.5268 |
| DNAJC8 | 0.6265 | 6.764209 | 19.603 | 3.70E-63 | 5.97E-60 | 132.998 |
| WDR90 | 0.62656 | 2.635706 | 9.72438 | 1.66E-20 | 4.14E-19 | 35.6907 |
| LINC01748 | 0.62667 | 1.67109 | 8.13997 | 3.49E-15 | 5.00E-14 | 23.6168 |
| TRIM22 | 0.6268 | 4.278533 | 6.89126 | 1.76E-11 | 1.67E-10 | 15.2555 |
| TLR3 | 0.62683 | 2.155515 | 9.10097 | 2.46E-18 | 4.96E-17 | 30.7592 |
| H2AC20 | 0.62685 | 1.249404 | 11.1918 | 5.69E-26 | 2.46E-24 | 48.1215 |
| CCDC24 | 0.62722 | 3.083441 | 11.5369 | 2.54E-27 | 1.27E-25 | 51.1952 |
| EXO1 | 0.62724 | 1.301788 | 7.02607 | 7.39E-12 | 7.34E-11 | 16.1047 |
| AHR | 0.62736 | 2.941518 | 6.94478 | 1.25E-11 | 1.21E-10 | 15.591 |
| KCNJ5 | 0.62752 | 2.208658 | 7.58539 | 1.76E-13 | 2.10E-12 | 19.7686 |
| TMUB1 | 0.62758 | 5.451893 | 15.1267 | 1.72E-42 | 4.39E-40 | 85.7986 |
| OTOS | 0.62785 | 1.43553 | 4.76648 | 2.49E-06 | 1.21E-05 | 3.74776 |
| IL2RG | 0.62786 | 1.564897 | 8.88886 | 1.28E-17 | 2.38E-16 | 29.1334 |
| COL14A1 | 0.62801 | 1.806846 | 6.04958 | 2.94E-09 | 2.11E-08 | 10.2642 |
| ETHE1 | 0.62873 | 4.78047 | 15.8414 | 1.06E-45 | 3.67E-43 | 93.1249 |
| TUT4 | 0.62887 | 3.059553 | 12.3492 | 1.39E-30 | 9.83E-29 | 58.632 |
| IL16 | 0.62899 | 1.586148 | 11.2901 | 2.36E-26 | 1.06E-24 | 48.9917 |
| HILPDA | 0.62904 | 4.2589 | 10.0142 | 1.51E-21 | 4.15E-20 | 38.058 |
| CEBPG | 0.6292 | 5.21131 | 13.2271 | 3.11E-34 | 3.43E-32 | 66.9558 |
| PAK4 | 0.62922 | 3.98526 | 16.658 | 2.03E-49 | 9.77E-47 | 101.618 |
| NUP62 | 0.6293 | 5.012762 | 14.7471 | 8.31E-41 | 1.80E-38 | 81.9549 |
| RPS16 | 0.62948 | 8.840969 | 11.8127 | 2.05E-28 | 1.16E-26 | 53.6897 |
| TMED5 | 0.6297 | 4.474135 | 11.5063 | 3.36E-27 | 1.66E-25 | 50.9204 |
| ZMYND10 | 0.62977 | 2.065934 | 6.90121 | 1.65E-11 | 1.57E-10 | 15.3178 |
| GBP5 | 0.6298 | 0.684971 | 9.32076 | 4.33E-19 | 9.40E-18 | 32.4723 |
| RIBC2 | 0.62989 | 0.73175 | 8.44008 | 3.85E-16 | 6.10E-15 | 25.7846 |
| GALNT3 | 0.62991 | 0.84062 | 9.03346 | 4.17E-18 | 8.19E-17 | 30.2388 |
| CASP8 | 0.63051 | 1.707176 | 11.8459 | 1.51E-28 | 8.65E-27 | 53.9925 |
| CDC42 | 0.63117 | 6.984261 | 15.8322 | 1.17E-45 | 3.97E-43 | 93.0294 |
| CHIC2 | 0.63131 | 4.523164 | 10.6677 | 5.75E-24 | 2.04E-22 | 43.5577 |
| ITPRIPL2 | 0.63158 | 3.060187 | 10.0519 | 1.10E-21 | 3.07E-20 | 38.3694 |
| AC005041.1 | 0.6316 | 3.400182 | 11.3979 | 8.95E-27 | 4.19E-25 | 49.9509 |
| EIF3I | 0.63194 | 7.837078 | 21.2656 | 4.94E-71 | 1.93E-67 | 150.981 |
| DEPP1 | 0.63225 | 5.031157 | 6.35005 | 5.03E-10 | 3.96E-09 | 11.9834 |
| LINC02798 | 0.63234 | 1.955916 | 10.1835 | 3.64E-22 | 1.06E-20 | 39.4613 |
| JAG1 | 0.63283 | 4.225007 | 7.46661 | 3.96E-13 | 4.56E-12 | 18.9719 |
| CDK6 | 0.6329 | 3.493505 | 5.98235 | 4.33E-09 | 3.05E-08 | 9.88924 |
| CDKN3 | 0.63291 | 2.218512 | 7.34942 | 8.74E-13 | 9.68E-12 | 18.1957 |
| ZBTB8OS | 0.63313 | 3.595709 | 20.8665 | 3.86E-69 | 1.32E-65 | 146.657 |
| AMIGO2 | 0.63333 | 1.894823 | 6.34452 | 5.20E-10 | 4.08E-09 | 11.9511 |
| C1orf53 | 0.63339 | 3.199073 | 9.99906 | 1.71E-21 | 4.69E-20 | 37.933 |
| SNRPD2 | 0.63342 | 7.223526 | 16.6863 | 1.51E-49 | 7.31E-47 | 101.915 |
| TMEM35B | 0.63381 | 2.926623 | 13.7864 | 1.28E-36 | 1.82E-34 | 72.395 |
| LINC01088 | 0.63412 | 4.420159 | 3.79768 | 0.000165 | 0.000597 | -0.2356 |
| CARD8 | 0.63428 | 3.246908 | 13.7702 | 1.51E-36 | 2.11E-34 | 72.2363 |
| NCAPD2 | 0.63453 | 4.845041 | 10.1662 | 4.21E-22 | 1.22E-20 | 39.3177 |
| C12orf75 | 0.63471 | 3.171557 | 9.33639 | 3.82E-19 | 8.35E-18 | 32.5952 |
| RGS18 | 0.63487 | 1.928697 | 9.07202 | 3.09E-18 | 6.15E-17 | 30.5357 |
| DDX20 | 0.63488 | 3.090655 | 15.2472 | 4.99E-43 | 1.38E-40 | 87.0259 |
| PINK1-AS | 0.63489 | 1.956347 | 16.7005 | 1.30E-49 | 6.40E-47 | 102.064 |
| RIOX2 | 0.63508 | 3.067066 | 8.71636 | 4.81E-17 | 8.39E-16 | 27.8313 |
| VCL | 0.63509 | 4.279817 | 8.78022 | 2.95E-17 | 5.27E-16 | 28.3112 |
| IER5L | 0.63513 | 3.107404 | 7.68044 | 9.10E-14 | 1.13E-12 | 20.4133 |
| AC145098.1 | 0.63553 | 1.374783 | 10.3632 | 7.91E-23 | 2.48E-21 | 40.9679 |
| HYI-AS1 | 0.63581 | 1.548577 | 12.7181 | 4.20E-32 | 3.44E-30 | 62.0959 |
| ADAMTS7 | 0.63583 | 1.157024 | 9.08059 | 2.89E-18 | 5.77E-17 | 30.6018 |
| ALDH16A1 | 0.63631 | 3.647529 | 12.3384 | 1.54E-30 | 1.08E-28 | 58.5318 |
| CD2 | 0.63632 | 0.947151 | 7.68821 | 8.63E-14 | 1.07E-12 | 20.4663 |
| KLK14 | 0.63651 | 1.565091 | 10.4473 | 3.85E-23 | 1.25E-21 | 41.6782 |
| CCDC88B | 0.63653 | 3.021129 | 12.0135 | 3.20E-29 | 1.97E-27 | 55.5257 |
| RCSD1 | 0.63678 | 3.482098 | 9.70594 | 1.93E-20 | 4.78E-19 | 35.5417 |
| MS4A14 | 0.63685 | 1.314242 | 10.6481 | 6.82E-24 | 2.40E-22 | 43.3891 |
| C10orf105 | 0.63685 | 1.081391 | 6.23887 | 9.75E-10 | 7.40E-09 | 11.339 |
| B2M | 0.63731 | 9.68731 | 8.71602 | 4.82E-17 | 8.41E-16 | 27.8288 |
| IMPACT | 0.63738 | 4.580564 | 7.9198 | 1.69E-14 | 2.27E-13 | 22.0641 |
| PABPC4 | 0.63743 | 5.43174 | 17.3571 | 1.22E-52 | 7.94E-50 | 108.978 |
| GZMA | 0.63743 | 1.121184 | 7.13999 | 3.51E-12 | 3.63E-11 | 16.8328 |
| MMP7 | 0.6376 | 0.655898 | 7.40042 | 6.20E-13 | 7.00E-12 | 18.5323 |
| LITAF | 0.63764 | 5.470438 | 7.68846 | 8.61E-14 | 1.07E-12 | 20.468 |
| DENND3 | 0.63783 | 3.034448 | 10.0256 | 1.37E-21 | 3.79E-20 | 38.1518 |
| POGLUT3 | 0.63787 | 3.42497 | 7.52423 | 2.67E-13 | 3.13E-12 | 19.3572 |
| TMEM67 | 0.63793 | 2.192814 | 11.1753 | 6.60E-26 | 2.84E-24 | 47.9755 |
| SERPINB8 | 0.63839 | 2.526972 | 10.305 | 1.30E-22 | 3.98E-21 | 40.4779 |
| MMRN1 | 0.63841 | 1.349281 | 8.50293 | 2.41E-16 | 3.90E-15 | 26.2459 |
| HOXD-AS2 | 0.63852 | 1.004341 | 6.96036 | 1.13E-11 | 1.10E-10 | 15.6891 |
| GBE1 | 0.63907 | 4.040874 | 11.5579 | 2.10E-27 | 1.07E-25 | 51.3838 |
| C9orf64 | 0.63927 | 2.930818 | 6.46271 | 2.55E-10 | 2.08E-09 | 12.6462 |
| CLSPN | 0.63933 | 1.15752 | 8.03469 | 7.46E-15 | 1.03E-13 | 22.8703 |
| MCM2 | 0.63964 | 4.057274 | 7.64112 | 1.20E-13 | 1.46E-12 | 20.1458 |
| AC016590.3 | 0.6399 | 2.804142 | 8.57723 | 1.38E-16 | 2.30E-15 | 26.7945 |
| NCF2 | 0.64034 | 3.259312 | 8.43698 | 3.94E-16 | 6.23E-15 | 25.7619 |
| LGALS3BP | 0.6406 | 7.4831 | 8.91021 | 1.09E-17 | 2.05E-16 | 29.2958 |
| C19orf38 | 0.6406 | 2.673856 | 10.1432 | 5.11E-22 | 1.47E-20 | 39.1261 |
| ISYNA1 | 0.64137 | 4.16802 | 10.7813 | 2.14E-24 | 7.87E-23 | 44.5354 |
| NUAK2 | 0.64139 | 1.76262 | 9.02534 | 4.45E-18 | 8.70E-17 | 30.1764 |
| NDC1 | 0.64151 | 3.672418 | 13.0426 | 1.86E-33 | 1.85E-31 | 65.1831 |
| MT2A | 0.64157 | 9.35866 | 7.37027 | 7.59E-13 | 8.48E-12 | 18.333 |
| MUTYH | 0.64163 | 3.596811 | 16.3479 | 5.32E-48 | 2.24E-45 | 98.3792 |
| ZNF436-AS1 | 0.64173 | 3.186613 | 13.1104 | 9.65E-34 | 1.01E-31 | 65.8328 |
| IGHG2 | 0.64185 | 1.1717 | 4.29974 | 2.08E-05 | 8.76E-05 | 1.72458 |
| FN1 | 0.64203 | 5.719356 | 6.24458 | 9.43E-10 | 7.18E-09 | 11.3718 |
| XKR8 | 0.64224 | 2.523166 | 7.05344 | 6.19E-12 | 6.21E-11 | 16.2788 |
| SLC44A5 | 0.6423 | 2.868785 | 6.38949 | 3.97E-10 | 3.17E-09 | 12.2143 |
| AL512785.1 | 0.64275 | 0.845699 | 7.06228 | 5.84E-12 | 5.88E-11 | 16.3351 |
| TMEM255A | 0.64277 | 4.90809 | 6.70951 | 5.56E-11 | 4.94E-10 | 14.1319 |
| ADAM12 | 0.64284 | 1.057144 | 7.19897 | 2.38E-12 | 2.51E-11 | 17.2134 |
| C4orf48 | 0.64303 | 5.362541 | 7.69261 | 8.37E-14 | 1.04E-12 | 20.4963 |
| AC125494.2 | 0.64322 | 2.406533 | 10.956 | 4.62E-25 | 1.83E-23 | 46.0513 |
| FTLP3 | 0.64325 | 2.833412 | 8.85574 | 1.65E-17 | 3.03E-16 | 28.8819 |
| GPBP1L1 | 0.64346 | 4.718138 | 16.0827 | 8.58E-47 | 3.24E-44 | 95.622 |
| ATF3 | 0.64363 | 3.483662 | 5.81582 | 1.11E-08 | 7.40E-08 | 8.9761 |
| PIK3R6 | 0.64379 | 1.418186 | 12.2112 | 5.07E-30 | 3.38E-28 | 57.3501 |
| AC010255.2 | 0.64399 | 0.642 | 8.9701 | 6.83E-18 | 1.31E-16 | 29.7529 |
| DLGAP1-AS1 | 0.64406 | 2.703608 | 10.8562 | 1.11E-24 | 4.21E-23 | 45.1841 |
| AC092807.3 | 0.6442 | 0.965289 | 12.2276 | 4.35E-30 | 2.93E-28 | 57.5018 |
| OASL | 0.64438 | 1.405668 | 6.71618 | 5.33E-11 | 4.75E-10 | 14.1727 |
| MYD88 | 0.64455 | 3.949349 | 7.60653 | 1.52E-13 | 1.82E-12 | 19.9115 |
| CGAS | 0.64488 | 1.262483 | 13.7958 | 1.17E-36 | 1.67E-34 | 72.4876 |
| ATL3 | 0.64516 | 4.602933 | 11.3556 | 1.31E-26 | 6.02E-25 | 49.574 |
| BTN3A2 | 0.64525 | 4.008293 | 7.46039 | 4.13E-13 | 4.74E-12 | 18.9305 |
| CCDC163 | 0.64544 | 3.794887 | 6.64331 | 8.40E-11 | 7.30E-10 | 13.7289 |
| SIGLEC7 | 0.64566 | 1.472863 | 8.92334 | 9.82E-18 | 1.85E-16 | 29.3958 |
| AC092675.1 | 0.64573 | 1.665947 | 8.74273 | 3.93E-17 | 6.92E-16 | 28.0292 |
| TTC7A | 0.64581 | 4.120068 | 13.957 | 2.36E-37 | 3.59E-35 | 74.0738 |
| CXCL9 | 0.64606 | 0.962416 | 6.61517 | 1.00E-10 | 8.59E-10 | 13.5585 |
| PTCRA | 0.64629 | 0.836596 | 8.97678 | 6.49E-18 | 1.25E-16 | 29.804 |
| DHX34 | 0.64659 | 3.447201 | 14.5001 | 1.02E-39 | 2.01E-37 | 79.4731 |
| AC010973.2 | 0.64701 | 1.547187 | 12.1842 | 6.52E-30 | 4.29E-28 | 57.0999 |
| OSM | 0.64716 | 1.62607 | 6.21645 | 1.11E-09 | 8.40E-09 | 11.2102 |
| S100A8 | 0.64755 | 2.625894 | 4.55936 | 6.53E-06 | 2.97E-05 | 2.82639 |
| GSN | 0.64817 | 6.715063 | 8.50211 | 2.42E-16 | 3.92E-15 | 26.2399 |
| AL133371.2 | 0.64832 | 1.881841 | 9.00711 | 5.13E-18 | 9.98E-17 | 30.0364 |
| CD101 | 0.6484 | 1.317013 | 8.88278 | 1.34E-17 | 2.49E-16 | 29.0871 |
| TLR10 | 0.64847 | 1.504135 | 9.14324 | 1.77E-18 | 3.61E-17 | 31.0865 |
| HMGB2 | 0.64888 | 5.978076 | 9.48222 | 1.19E-19 | 2.71E-18 | 33.7486 |
| FBL | 0.64899 | 6.639533 | 12.424 | 6.85E-31 | 5.03E-29 | 59.3306 |
| BLVRB | 0.6491 | 5.494067 | 9.26861 | 6.56E-19 | 1.39E-17 | 32.0633 |
| CFAP73 | 0.64918 | 0.906731 | 6.7609 | 4.03E-11 | 3.64E-10 | 14.4471 |
| SLC35D1 | 0.64961 | 3.31869 | 12.8349 | 1.37E-32 | 1.20E-30 | 63.2025 |
| EPHB2 | 0.64964 | 2.766203 | 8.52992 | 1.97E-16 | 3.22E-15 | 26.4448 |
| INKA1 | 0.64977 | 3.906053 | 9.59321 | 4.83E-20 | 1.16E-18 | 34.6347 |
| VAMP5 | 0.6498 | 6.274859 | 8.69455 | 5.68E-17 | 9.85E-16 | 27.668 |
| PRPF38B | 0.6502 | 4.893645 | 15.2746 | 3.77E-43 | 1.05E-40 | 87.3057 |
| CTPS1 | 0.6504 | 3.763824 | 11.8617 | 1.30E-28 | 7.53E-27 | 54.1359 |
| C8orf88 | 0.65045 | 2.046367 | 8.78604 | 2.83E-17 | 5.05E-16 | 28.355 |
| SGO1 | 0.6505 | 1.061803 | 8.52785 | 2.00E-16 | 3.26E-15 | 26.4296 |
| LRR1 | 0.65122 | 2.641016 | 12.963 | 4.01E-33 | 3.82E-31 | 64.4222 |
| WRAP73 | 0.6513 | 3.277438 | 17.5315 | 1.89E-53 | 1.30E-50 | 110.824 |
| IFIH1 | 0.65153 | 3.195328 | 8.36017 | 6.96E-16 | 1.08E-14 | 25.2016 |
| RPL11 | 0.65156 | 9.358927 | 13.259 | 2.28E-34 | 2.56E-32 | 67.2633 |
| FBLN5 | 0.65183 | 3.248421 | 6.09178 | 2.31E-09 | 1.68E-08 | 10.5014 |
| AC093673.1 | 0.65196 | 4.837239 | 9.1258 | 2.03E-18 | 4.11E-17 | 30.9513 |
| SOCS1 | 0.65208 | 1.659984 | 8.31809 | 9.49E-16 | 1.45E-14 | 24.8963 |
| TCF7 | 0.65211 | 1.366342 | 11.5348 | 2.59E-27 | 1.30E-25 | 51.1762 |
| ZMYM1 | 0.65251 | 2.637341 | 13.8916 | 4.52E-37 | 6.68E-35 | 73.4289 |
| MRPL20 | 0.65251 | 5.954322 | 20.657 | 3.80E-68 | 9.93E-65 | 144.389 |
| TSTD1 | 0.65259 | 2.60148 | 4.96358 | 9.66E-07 | 4.96E-06 | 4.65905 |
| AC025171.5 | 0.65268 | 0.913419 | 9.51836 | 8.86E-20 | 2.06E-18 | 34.0364 |
| HGF | 0.65273 | 1.227988 | 8.18964 | 2.43E-15 | 3.54E-14 | 23.9715 |
| MKNK1 | 0.65293 | 3.448672 | 12.3075 | 2.05E-30 | 1.41E-28 | 58.2441 |
| CENPK | 0.65305 | 1.314827 | 8.04126 | 7.11E-15 | 9.89E-14 | 22.9167 |
| TMEM71 | 0.65329 | 1.015045 | 8.79738 | 2.59E-17 | 4.66E-16 | 28.4405 |
| TNFSF8 | 0.65334 | 1.436495 | 9.6599 | 2.81E-20 | 6.82E-19 | 35.1704 |
| MSX1 | 0.65342 | 4.45834 | 9.31914 | 4.39E-19 | 9.50E-18 | 32.4596 |
| CITED1 | 0.65368 | 3.693564 | 5.91173 | 6.46E-09 | 4.46E-08 | 9.4993 |
| FGFBP2 | 0.65372 | 1.574198 | 6.08796 | 2.36E-09 | 1.71E-08 | 10.4798 |
| HOXB3 | 0.65393 | 0.480432 | 7.06077 | 5.90E-12 | 5.93E-11 | 16.3255 |
| LIMD2 | 0.65429 | 3.555338 | 12.0463 | 2.36E-29 | 1.47E-27 | 55.8275 |
| TMSB4XP1 | 0.65434 | 1.614382 | 9.59539 | 4.75E-20 | 1.14E-18 | 34.6521 |
| S100PBP | 0.65447 | 3.386545 | 16.1983 | 2.56E-47 | 9.95E-45 | 96.8224 |
| GAB3 | 0.65454 | 2.123369 | 11.9489 | 5.82E-29 | 3.49E-27 | 54.9333 |
| AL359921.2 | 0.65455 | 2.462716 | 12.7606 | 2.80E-32 | 2.34E-30 | 62.4979 |
| NOTCH2 | 0.65481 | 5.086637 | 8.42373 | 4.34E-16 | 6.85E-15 | 25.665 |
| ANKRD62P1-PARP4P3 | 0.65485 | 0.928843 | 5.81347 | 1.12E-08 | 7.49E-08 | 8.96337 |
| LAMB1 | 0.65504 | 3.246525 | 6.12642 | 1.89E-09 | 1.39E-08 | 10.6971 |
| ACCS | 0.65521 | 1.713287 | 8.8618 | 1.58E-17 | 2.91E-16 | 28.9278 |
| ZNF436 | 0.65547 | 4.569071 | 10.5089 | 2.27E-23 | 7.55E-22 | 42.2015 |
| ZFP36L1 | 0.65569 | 6.935903 | 8.936 | 8.90E-18 | 1.69E-16 | 29.4923 |
| HTRA3 | 0.65591 | 1.179284 | 7.78698 | 4.33E-14 | 5.56E-13 | 21.1432 |
| GMPR | 0.65598 | 4.250939 | 5.52419 | 5.46E-08 | 3.33E-07 | 7.43107 |
| ECHDC2 | 0.65618 | 2.010372 | 8.41174 | 4.75E-16 | 7.46E-15 | 25.5774 |
| LAMTOR5 | 0.65619 | 6.131442 | 22.0187 | 1.32E-74 | 1.03E-70 | 159.142 |
| CFD | 0.65663 | 2.908823 | 8.69829 | 5.52E-17 | 9.58E-16 | 27.6959 |
| AURKA | 0.65664 | 2.340212 | 8.11403 | 4.21E-15 | 5.98E-14 | 23.4321 |
| ARL11 | 0.65706 | 1.315983 | 12.6667 | 6.86E-32 | 5.50E-30 | 61.6099 |
| ITGA2 | 0.65785 | 2.263836 | 7.29468 | 1.26E-12 | 1.37E-11 | 17.8364 |
| MIR25 | 0.65815 | 3.963056 | 7.5999 | 1.59E-13 | 1.91E-12 | 19.8667 |
| MAF | 0.65817 | 4.547881 | 10.2671 | 1.79E-22 | 5.42E-21 | 40.16 |
| HSD3B7 | 0.65851 | 2.615525 | 10.4857 | 2.77E-23 | 9.14E-22 | 42.0038 |
| ASIP | 0.65874 | 1.719041 | 8.7728 | 3.13E-17 | 5.56E-16 | 28.2553 |
| LRRC17 | 0.6588 | 3.546479 | 5.97534 | 4.50E-09 | 3.17E-08 | 9.85036 |
| AC022432.1 | 0.65883 | 1.27446 | 9.19809 | 1.15E-18 | 2.38E-17 | 31.5127 |
| SLC66A1 | 0.65895 | 4.426616 | 20.3197 | 1.51E-66 | 3.18E-63 | 140.739 |
| WDR45P1 | 0.65924 | 1.037246 | 9.98486 | 1.92E-21 | 5.24E-20 | 37.8161 |
| COL4A6 | 0.65927 | 1.231671 | 8.76514 | 3.31E-17 | 5.88E-16 | 28.1976 |
| CTBS | 0.65935 | 2.536575 | 11.5703 | 1.88E-27 | 9.57E-26 | 51.4956 |
| FAM181A-AS1 | 0.65991 | 1.392772 | 7.48541 | 3.48E-13 | 4.03E-12 | 19.0974 |
| TRIM38 | 0.66004 | 1.842589 | 12.5493 | 2.09E-31 | 1.63E-29 | 60.5047 |
| STK33 | 0.66006 | 2.974293 | 11.5727 | 1.84E-27 | 9.37E-26 | 51.517 |
| ACY3 | 0.66047 | 3.5572 | 4.40805 | 1.29E-05 | 5.61E-05 | 2.17701 |
| SNHG3 | 0.66071 | 3.484293 | 12.193 | 6.01E-30 | 3.96E-28 | 57.1814 |
| LINC00339 | 0.66094 | 4.211224 | 16.9474 | 9.50E-51 | 5.37E-48 | 104.656 |
| C21orf62 | 0.661 | 2.900641 | 4.89522 | 1.35E-06 | 6.78E-06 | 4.33919 |
| EPSTI1 | 0.6611 | 1.991575 | 7.7695 | 4.89E-14 | 6.26E-13 | 21.0229 |
| WDR34 | 0.6611 | 5.590765 | 12.2115 | 5.05E-30 | 3.38E-28 | 57.3531 |
| PRKCD | 0.6613 | 4.074194 | 8.3612 | 6.91E-16 | 1.07E-14 | 25.2091 |
| CAPZB | 0.66133 | 7.694919 | 19.0269 | 1.92E-60 | 2.45E-57 | 126.795 |
| AC131097.3 | 0.66147 | 0.693547 | 8.97785 | 6.44E-18 | 1.24E-16 | 29.8122 |
| AC093627.7 | 0.66167 | 1.768185 | 8.86561 | 1.53E-17 | 2.83E-16 | 28.9567 |
| AC099791.2 | 0.66271 | 1.439957 | 10.1726 | 3.99E-22 | 1.16E-20 | 39.3707 |
| JAK3 | 0.66288 | 1.68168 | 8.26292 | 1.42E-15 | 2.12E-14 | 24.4978 |
| EFEMP2 | 0.66295 | 3.653889 | 6.17389 | 1.43E-09 | 1.06E-08 | 10.9669 |
| AGPAT2 | 0.66318 | 4.12293 | 10.6542 | 6.47E-24 | 2.28E-22 | 43.4415 |
| OSBPL9 | 0.66329 | 4.055325 | 11.3134 | 1.92E-26 | 8.65E-25 | 49.1985 |
| CKS2 | 0.6633 | 5.186447 | 7.18778 | 2.56E-12 | 2.70E-11 | 17.1409 |
| SRM | 0.66382 | 6.713905 | 16.723 | 1.02E-49 | 5.09E-47 | 102.3 |
| ESPL1 | 0.66408 | 1.199167 | 7.82232 | 3.38E-14 | 4.39E-13 | 21.3871 |
| GBX2 | 0.66432 | 2.649623 | 5.24711 | 2.33E-07 | 1.30E-06 | 6.02803 |
| DENND2D | 0.66436 | 1.294844 | 10.276 | 1.66E-22 | 5.04E-21 | 40.2346 |
| LIPG | 0.66465 | 1.706854 | 8.14174 | 3.44E-15 | 4.94E-14 | 23.6294 |
| AL645924.1 | 0.6647 | 1.422739 | 7.42087 | 5.40E-13 | 6.13E-12 | 18.6678 |
| CACHD1 | 0.66478 | 4.351094 | 9.60827 | 4.28E-20 | 1.03E-18 | 34.7555 |
| PPP1R13L | 0.66532 | 2.784864 | 8.99783 | 5.51E-18 | 1.07E-16 | 29.9652 |
| ARHGAP6 | 0.66533 | 2.331126 | 9.09961 | 2.49E-18 | 5.00E-17 | 30.7487 |
| TYMS | 0.66536 | 3.872871 | 5.78661 | 1.30E-08 | 8.63E-08 | 8.81826 |
| MRPL20-AS1 | 0.6657 | 4.897885 | 20.3375 | 1.24E-66 | 2.84E-63 | 140.932 |
| C1S | 0.66578 | 4.569503 | 5.81832 | 1.09E-08 | 7.31E-08 | 8.98968 |
| MFAP4 | 0.66641 | 4.512766 | 5.96202 | 4.86E-09 | 3.41E-08 | 9.77658 |
| PLTP | 0.66667 | 8.172556 | 8.51559 | 2.19E-16 | 3.56E-15 | 26.3392 |
| STAC | 0.66667 | 0.740868 | 6.57603 | 1.27E-10 | 1.08E-09 | 13.3226 |
| TCEA3 | 0.66755 | 1.837553 | 7.472 | 3.82E-13 | 4.40E-12 | 19.0079 |
| NAT14 | 0.668 | 5.596589 | 13.8265 | 8.62E-37 | 1.25E-34 | 72.789 |
| DRAM2 | 0.66813 | 4.988456 | 16.2321 | 1.80E-47 | 7.18E-45 | 97.1734 |
| SYTL3 | 0.66901 | 2.302507 | 9.86617 | 5.16E-21 | 1.35E-19 | 36.8431 |
| CXCR4 | 0.66907 | 4.984135 | 6.73124 | 4.85E-11 | 4.34E-10 | 14.265 |
| PI4K2B | 0.66937 | 2.574795 | 13.5722 | 1.06E-35 | 1.35E-33 | 70.2997 |
| C19orf48 | 0.66963 | 4.537642 | 11.9508 | 5.72E-29 | 3.43E-27 | 54.9508 |
| AC005261.3 | 0.6697 | 3.209378 | 14.4862 | 1.17E-39 | 2.30E-37 | 79.3331 |
| PPP1R3B | 0.66989 | 2.617406 | 10.0796 | 8.71E-22 | 2.45E-20 | 38.5986 |
| NFE2L3 | 0.67 | 2.191293 | 11.1646 | 7.26E-26 | 3.12E-24 | 47.8808 |
| OXTR | 0.67016 | 2.740773 | 5.28908 | 1.88E-07 | 1.06E-06 | 6.23646 |
| AL354760.1 | 0.67031 | 1.988561 | 12.869 | 9.90E-33 | 8.85E-31 | 63.5271 |
| KIF18A | 0.67039 | 0.976464 | 9.95556 | 2.46E-21 | 6.63E-20 | 37.5752 |
| SVBP | 0.67074 | 4.96548 | 17.1823 | 7.84E-52 | 4.77E-49 | 107.131 |
| CENPE | 0.67085 | 1.090901 | 8.68534 | 6.09E-17 | 1.05E-15 | 27.5991 |
| COL9A3 | 0.67115 | 3.529496 | 4.83076 | 1.84E-06 | 9.06E-06 | 4.04127 |
| KIF14 | 0.67145 | 1.092046 | 8.40591 | 4.96E-16 | 7.77E-15 | 25.5348 |
| PDCD5 | 0.67157 | 5.529914 | 20.7459 | 1.44E-68 | 4.39E-65 | 145.351 |
| UBE2S | 0.67162 | 4.034526 | 10.404 | 5.58E-23 | 1.78E-21 | 41.3122 |
| PIRT | 0.6718 | 2.52231 | 4.78907 | 2.24E-06 | 1.09E-05 | 3.85051 |
| VENTX | 0.67182 | 1.044116 | 10.1225 | 6.08E-22 | 1.74E-20 | 38.9536 |
| RSPH1 | 0.67189 | 3.066049 | 6.73454 | 4.75E-11 | 4.26E-10 | 14.2852 |
| SYNGR2 | 0.67239 | 5.031309 | 9.50878 | 9.58E-20 | 2.21E-18 | 33.96 |
| DUSP23 | 0.67281 | 4.524268 | 10.0981 | 7.46E-22 | 2.11E-20 | 38.7513 |
| NSUN7 | 0.67295 | 0.70158 | 10.2156 | 2.77E-22 | 8.20E-21 | 39.7293 |
| AL392172.1 | 0.67322 | 3.604352 | 12.3887 | 9.56E-31 | 6.93E-29 | 59.0007 |
| FOLR1 | 0.67355 | 1.802812 | 6.97579 | 1.02E-11 | 9.97E-11 | 15.7864 |
| TAF13 | 0.67357 | 5.959505 | 15.4836 | 4.36E-44 | 1.35E-41 | 89.4426 |
| AC103591.3 | 0.67366 | 1.643287 | 9.49326 | 1.09E-19 | 2.49E-18 | 33.8364 |
| AC099792.1 | 0.67395 | 2.358498 | 8.04436 | 6.96E-15 | 9.69E-14 | 22.9386 |
| IGFBP7-AS1 | 0.67456 | 1.880959 | 6.75332 | 4.23E-11 | 3.81E-10 | 14.4005 |
| ABHD15 | 0.67567 | 3.165905 | 13.912 | 3.69E-37 | 5.51E-35 | 73.6299 |
| BTBD19 | 0.67578 | 1.972964 | 10.7656 | 2.45E-24 | 8.98E-23 | 44.4 |
| BARD1 | 0.67586 | 2.806078 | 10.3579 | 8.28E-23 | 2.59E-21 | 40.9226 |
| AL604028.1 | 0.67587 | 3.583486 | 17.6925 | 3.38E-54 | 2.47E-51 | 112.533 |
| RNY3P16 | 0.67603 | 1.724417 | 6.92888 | 1.38E-11 | 1.33E-10 | 15.4911 |
| KDM1A | 0.67668 | 5.816963 | 12.8529 | 1.16E-32 | 1.02E-30 | 63.3737 |
| SPATS2L | 0.67672 | 4.304369 | 11.3234 | 1.75E-26 | 7.96E-25 | 49.2868 |
| TGFBI | 0.6769 | 3.423529 | 5.08297 | 5.35E-07 | 2.85E-06 | 5.22721 |
| CD302 | 0.677 | 2.270468 | 8.7887 | 2.77E-17 | 4.96E-16 | 28.3751 |
| LSM14A | 0.67702 | 6.14513 | 16.1533 | 4.10E-47 | 1.57E-44 | 96.3545 |
| OSR2 | 0.67719 | 0.516464 | 9.64641 | 3.13E-20 | 7.58E-19 | 35.0619 |
| GJC1 | 0.6772 | 2.055578 | 8.71529 | 4.85E-17 | 8.46E-16 | 27.8233 |
| SF3A3 | 0.67737 | 5.579614 | 19.6207 | 3.05E-63 | 5.07E-60 | 133.188 |
| ITGA5 | 0.67745 | 3.060782 | 7.81641 | 3.52E-14 | 4.56E-13 | 21.3462 |
| SCP2 | 0.67771 | 5.387181 | 16.572 | 5.03E-49 | 2.32E-46 | 100.719 |
| SH3GLB1 | 0.67825 | 5.712561 | 13.5133 | 1.90E-35 | 2.35E-33 | 69.726 |
| BCAT1 | 0.67828 | 2.595445 | 6.07571 | 2.53E-09 | 1.83E-08 | 10.4109 |
| FIBIN | 0.67846 | 6.394969 | 5.2119 | 2.79E-07 | 1.55E-06 | 5.85431 |
| SMIM3 | 0.67872 | 4.324812 | 8.68271 | 6.21E-17 | 1.07E-15 | 27.5795 |
| FCHSD1 | 0.67937 | 2.190149 | 11.2564 | 3.19E-26 | 1.42E-24 | 48.6926 |
| PARP12 | 0.67938 | 2.847119 | 9.1004 | 2.47E-18 | 4.98E-17 | 30.7549 |
| UBE2J2 | 0.67946 | 4.740386 | 21.6271 | 9.50E-73 | 5.80E-69 | 154.899 |
| CENPW | 0.67976 | 2.848567 | 8.87035 | 1.48E-17 | 2.73E-16 | 28.9927 |
| SCIMP | 0.68046 | 1.696929 | 9.78326 | 1.02E-20 | 2.61E-19 | 36.1679 |
| AL133517.1 | 0.68075 | 1.267178 | 15.2156 | 6.91E-43 | 1.88E-40 | 86.704 |
| PHF13 | 0.68114 | 4.467407 | 15.6497 | 7.81E-45 | 2.52E-42 | 91.1484 |
| DLEC1 | 0.68124 | 1.507264 | 8.59223 | 1.23E-16 | 2.06E-15 | 26.9058 |
| CENPM | 0.68145 | 2.51935 | 7.75289 | 5.49E-14 | 6.99E-13 | 20.9088 |
| GAS1 | 0.68161 | 4.279858 | 6.85826 | 2.17E-11 | 2.03E-10 | 15.0497 |
| AC007786.2 | 0.6817 | 1.792923 | 11.1825 | 6.19E-26 | 2.67E-24 | 48.0386 |
| COLGALT1 | 0.68198 | 5.090985 | 13.2711 | 2.02E-34 | 2.31E-32 | 67.3801 |
| FKBP5 | 0.68202 | 4.022105 | 5.36132 | 1.29E-07 | 7.48E-07 | 6.59861 |
| MKI67 | 0.68202 | 2.284385 | 5.37231 | 1.22E-07 | 7.08E-07 | 6.65408 |
| CYFIP1 | 0.68211 | 5.519419 | 10.4476 | 3.85E-23 | 1.25E-21 | 41.6806 |
| CDC25C | 0.68218 | 1.135558 | 8.86282 | 1.57E-17 | 2.88E-16 | 28.9356 |
| ZIC5 | 0.68235 | 1.713459 | 9.24365 | 7.99E-19 | 1.68E-17 | 31.8681 |
| RNPEP | 0.68257 | 5.069206 | 18.3246 | 3.81E-57 | 3.79E-54 | 119.267 |
| DRC7 | 0.68273 | 0.839798 | 10.1454 | 5.01E-22 | 1.45E-20 | 39.1442 |
| ARHGAP15 | 0.68279 | 1.29458 | 13.0147 | 2.43E-33 | 2.39E-31 | 64.9169 |
| SECTM1 | 0.6829 | 1.840872 | 7.01898 | 7.74E-12 | 7.66E-11 | 16.0597 |
| SCPEP1 | 0.68314 | 4.680401 | 12.7635 | 2.72E-32 | 2.28E-30 | 62.5251 |
| STING1 | 0.68364 | 3.662541 | 10.6532 | 6.53E-24 | 2.30E-22 | 43.4328 |
| DCLRE1B | 0.68372 | 3.479883 | 13.3627 | 8.29E-35 | 9.64E-33 | 68.265 |
| CLEC2B | 0.68386 | 2.069532 | 10.2491 | 2.09E-22 | 6.26E-21 | 40.0089 |
| PLCE1 | 0.68398 | 2.878906 | 10.533 | 1.85E-23 | 6.19E-22 | 42.4058 |
| JUNB | 0.68406 | 6.689614 | 6.01767 | 3.54E-09 | 2.52E-08 | 10.0858 |
| AC079015.1 | 0.68412 | 2.381079 | 9.21623 | 9.93E-19 | 2.07E-17 | 31.654 |
| SMCO4 | 0.68462 | 3.901359 | 12.0054 | 3.45E-29 | 2.12E-27 | 55.4512 |
| TMCO4 | 0.68488 | 2.791289 | 9.68026 | 2.38E-20 | 5.84E-19 | 35.3345 |
| PKN2 | 0.68528 | 4.319412 | 13.1065 | 1.00E-33 | 1.04E-31 | 65.7954 |
| GPR132 | 0.68531 | 1.403376 | 11.7773 | 2.83E-28 | 1.58E-26 | 53.3675 |
| TRAPPC3 | 0.68566 | 5.377375 | 21.0013 | 8.86E-70 | 3.24E-66 | 148.118 |
| SMC4 | 0.68566 | 2.469226 | 7.8091 | 3.70E-14 | 4.80E-13 | 21.2958 |
| CRISPLD1 | 0.68664 | 5.013715 | 7.09705 | 4.66E-12 | 4.75E-11 | 16.5572 |
| CROT | 0.68683 | 3.954054 | 11.2818 | 2.54E-26 | 1.14E-24 | 48.9176 |
| HMGCL | 0.68686 | 4.022066 | 18.4797 | 7.15E-58 | 7.68E-55 | 120.926 |
| MXD3 | 0.6878 | 2.218622 | 8.47178 | 3.04E-16 | 4.87E-15 | 26.0169 |
| REM1 | 0.68794 | 1.944311 | 5.23471 | 2.48E-07 | 1.39E-06 | 5.96676 |
| MAP7D3 | 0.68822 | 2.198215 | 12.1915 | 6.09E-30 | 4.01E-28 | 57.1672 |
| ADPRH | 0.68834 | 2.24536 | 11.6767 | 7.12E-28 | 3.80E-26 | 52.4554 |
| STIL | 0.6884 | 1.407518 | 11.4891 | 3.92E-27 | 1.92E-25 | 50.7667 |
| HOXD13 | 0.68852 | 0.4016 | 7.29477 | 1.26E-12 | 1.37E-11 | 17.8369 |
| TNFRSF19 | 0.68867 | 3.729485 | 6.52217 | 1.77E-10 | 1.48E-09 | 12.9999 |
| IGKC | 0.68882 | 1.6119 | 4.5779 | 6.00E-06 | 2.75E-05 | 2.90733 |
| SRSF11 | 0.6893 | 5.65653 | 14.1702 | 2.81E-38 | 4.86E-36 | 76.1828 |
| ZNF296 | 0.68946 | 1.909168 | 12.6909 | 5.45E-32 | 4.40E-30 | 61.8382 |
| BHLHE41 | 0.68954 | 5.992554 | 7.61457 | 1.44E-13 | 1.73E-12 | 19.9659 |
| CENPS | 0.6903 | 1.910544 | 17.8647 | 5.34E-55 | 4.18E-52 | 114.363 |
| BAX | 0.69045 | 5.555291 | 14.5686 | 5.08E-40 | 1.04E-37 | 80.1594 |
| TLR6 | 0.69045 | 1.507373 | 11.5006 | 3.54E-27 | 1.74E-25 | 50.8695 |
| YBX3 | 0.69073 | 3.900431 | 9.56119 | 6.27E-20 | 1.48E-18 | 34.3783 |
| HOXD10 | 0.69101 | 0.54187 | 6.95404 | 1.18E-11 | 1.14E-10 | 15.6493 |
| MYOF | 0.69103 | 3.081192 | 7.78823 | 4.29E-14 | 5.52E-13 | 21.1518 |
| ZNF432 | 0.69117 | 2.954452 | 15.1627 | 1.19E-42 | 3.12E-40 | 86.1646 |
| MLXIPL | 0.69126 | 2.689111 | 8.37564 | 6.21E-16 | 9.65E-15 | 25.3142 |
| PDLIM4 | 0.69166 | 2.40714 | 5.10779 | 4.73E-07 | 2.54E-06 | 5.34681 |
| MIR223HG | 0.69214 | 1.928616 | 9.57574 | 5.57E-20 | 1.32E-18 | 34.4948 |
| RELB | 0.69231 | 3.069168 | 11.3786 | 1.07E-26 | 4.95E-25 | 49.7788 |
| BVES | 0.69241 | 2.451187 | 8.26914 | 1.36E-15 | 2.03E-14 | 24.5426 |
| BTN2A2 | 0.69243 | 2.599529 | 11.2114 | 4.78E-26 | 2.09E-24 | 48.2943 |
| LAP3 | 0.69267 | 6.258886 | 11.4864 | 4.02E-27 | 1.96E-25 | 50.7423 |
| CARD19 | 0.69271 | 3.511526 | 8.68082 | 6.30E-17 | 1.09E-15 | 27.5653 |
| DENND1C | 0.69272 | 2.004565 | 12.7119 | 4.46E-32 | 3.64E-30 | 62.0372 |
| FKBP9 | 0.69282 | 4.455072 | 7.71071 | 7.38E-14 | 9.23E-13 | 20.6198 |
| CLEC17A | 0.69334 | 0.97513 | 8.68635 | 6.04E-17 | 1.04E-15 | 27.6066 |
| CD93 | 0.69358 | 3.190174 | 6.09331 | 2.29E-09 | 1.67E-08 | 10.51 |
| SLC8B1 | 0.6937 | 3.518273 | 12.2965 | 2.28E-30 | 1.57E-28 | 58.1413 |
| MNS1 | 0.69395 | 3.089478 | 11.1442 | 8.71E-26 | 3.71E-24 | 47.701 |
| MRPS15 | 0.69442 | 4.687482 | 18.6187 | 1.59E-58 | 1.78E-55 | 122.415 |
| OAS2 | 0.6945 | 2.845675 | 6.67184 | 7.04E-11 | 6.16E-10 | 13.9022 |
| TMEM91 | 0.69475 | 2.922603 | 11.1299 | 9.89E-26 | 4.20E-24 | 47.5752 |
| CDC6 | 0.69528 | 1.655195 | 8.89752 | 1.20E-17 | 2.24E-16 | 29.1992 |
| SERPINA3 | 0.69578 | 1.13002 | 7.38146 | 7.04E-13 | 7.90E-12 | 18.4069 |
| CNPY4 | 0.69588 | 4.842122 | 14.2607 | 1.13E-38 | 2.04E-36 | 77.0827 |
| FES | 0.69598 | 2.887857 | 12.2165 | 4.82E-30 | 3.23E-28 | 57.3988 |
| CD248 | 0.69646 | 2.705331 | 6.37802 | 4.25E-10 | 3.38E-09 | 12.147 |
| H19 | 0.6965 | 0.744637 | 5.39585 | 1.08E-07 | 6.31E-07 | 6.77329 |
| COL5A2 | 0.69651 | 3.028377 | 6.07731 | 2.51E-09 | 1.82E-08 | 10.4199 |
| SERPINF2 | 0.69657 | 1.992163 | 12.2716 | 2.88E-30 | 1.96E-28 | 57.9099 |
| LYZ | 0.69669 | 3.059093 | 4.70729 | 3.30E-06 | 1.57E-05 | 3.48068 |
| AC087273.2 | 0.69698 | 1.750844 | 9.91712 | 3.38E-21 | 9.01E-20 | 37.2598 |
| GNGT2 | 0.69701 | 1.767458 | 12.4063 | 8.10E-31 | 5.91E-29 | 59.1646 |
| MAATS1 | 0.69711 | 1.706704 | 9.38143 | 2.67E-19 | 5.94E-18 | 32.9501 |
| CEACAM21 | 0.69732 | 1.821237 | 10.8861 | 8.54E-25 | 3.30E-23 | 45.443 |
| SPEF1 | 0.69755 | 2.347463 | 6.68268 | 6.58E-11 | 5.78E-10 | 13.9682 |
| TEX26 | 0.69771 | 1.349858 | 6.22737 | 1.04E-09 | 7.90E-09 | 11.2729 |
| AL139287.1 | 0.69796 | 4.704638 | 12.391 | 9.36E-31 | 6.80E-29 | 59.0221 |
| SPN | 0.69803 | 1.429818 | 11.7456 | 3.79E-28 | 2.09E-26 | 53.0797 |
| AC015922.3 | 0.69824 | 3.033697 | 7.9331 | 1.54E-14 | 2.08E-13 | 22.157 |
| SELENOF | 0.69875 | 7.208788 | 17.0927 | 2.03E-51 | 1.20E-48 | 106.187 |
| MAP4K1 | 0.69897 | 1.966824 | 15.026 | 4.84E-42 | 1.17E-39 | 84.7747 |
| YBX1P10 | 0.69903 | 3.072662 | 11.5888 | 1.59E-27 | 8.14E-26 | 51.6624 |
| COL1A2 | 0.69904 | 3.450604 | 5.83948 | 9.71E-09 | 6.54E-08 | 9.10449 |
| AL355922.1 | 0.6992 | 1.067739 | 8.57594 | 1.39E-16 | 2.32E-15 | 26.785 |
| ANO6 | 0.69955 | 4.589969 | 9.44637 | 1.58E-19 | 3.59E-18 | 33.4639 |
| ZNF691 | 0.69956 | 4.056701 | 20.2506 | 3.20E-66 | 6.50E-63 | 139.992 |
| DDOST | 0.69972 | 6.981539 | 16.6038 | 3.60E-49 | 1.69E-46 | 101.052 |
| PARP14 | 0.70046 | 3.865775 | 9.07209 | 3.09E-18 | 6.15E-17 | 30.5363 |
| NEK6 | 0.70136 | 5.11183 | 9.05931 | 3.41E-18 | 6.76E-17 | 30.4378 |
| KCNE3 | 0.70152 | 1.823523 | 11.1922 | 5.67E-26 | 2.46E-24 | 48.125 |
| FAM111B | 0.70208 | 1.510245 | 7.28421 | 1.35E-12 | 1.47E-11 | 17.7679 |
| TFEC | 0.70229 | 1.408266 | 11.6624 | 8.11E-28 | 4.29E-26 | 52.3267 |
| H2AC11 | 0.70241 | 0.974113 | 11.5099 | 3.25E-27 | 1.61E-25 | 50.953 |
| RASGRP4 | 0.70287 | 1.814509 | 11.6649 | 7.93E-28 | 4.20E-26 | 52.3493 |
| PFN1 | 0.70318 | 8.559887 | 16.9632 | 8.03E-51 | 4.58E-48 | 104.823 |
| MMP2 | 0.70331 | 4.856895 | 7.29238 | 1.28E-12 | 1.39E-11 | 17.8213 |
| TMEM52B | 0.70366 | 1.624526 | 10.5151 | 2.15E-23 | 7.19E-22 | 42.2539 |
| AOAH | 0.70369 | 3.291339 | 8.89049 | 1.27E-17 | 2.36E-16 | 29.1457 |
| TRIP10 | 0.7039 | 3.736438 | 10.2711 | 1.73E-22 | 5.25E-21 | 40.1936 |
| TMEM106C | 0.70402 | 4.850956 | 12.054 | 2.19E-29 | 1.38E-27 | 55.8987 |
| FAAP20 | 0.70403 | 3.364596 | 18.0444 | 7.76E-56 | 6.86E-53 | 116.277 |
| CAPZA1 | 0.70418 | 5.501547 | 16.263 | 1.30E-47 | 5.39E-45 | 97.4955 |
| SLC39A1 | 0.70462 | 6.239103 | 16.7275 | 9.75E-50 | 4.90E-47 | 102.347 |
| CYTIP | 0.70463 | 1.620066 | 10.7893 | 2.00E-24 | 7.37E-23 | 44.6045 |
| SH3BGRL3 | 0.70509 | 7.34225 | 17.0075 | 5.02E-51 | 2.90E-48 | 105.289 |
| TTK | 0.70529 | 1.383982 | 7.55373 | 2.18E-13 | 2.58E-12 | 19.5553 |
| EME1 | 0.70551 | 1.870804 | 10.0363 | 1.25E-21 | 3.48E-20 | 38.2403 |
| AC064875.1 | 0.70573 | 0.839569 | 7.27357 | 1.45E-12 | 1.57E-11 | 17.6984 |
| LYPLA2 | 0.70577 | 5.464959 | 18.0225 | 9.82E-56 | 8.54E-53 | 116.043 |
| MFSD10 | 0.70578 | 5.403895 | 14.2824 | 9.11E-39 | 1.65E-36 | 77.2989 |
| GNLY | 0.70633 | 1.273646 | 8.26557 | 1.40E-15 | 2.08E-14 | 24.5169 |
| CD180 | 0.70647 | 1.590402 | 9.70634 | 1.92E-20 | 4.77E-19 | 35.545 |
| SH2D4A | 0.70656 | 0.941009 | 8.55615 | 1.62E-16 | 2.67E-15 | 26.6385 |
| MYBPH | 0.70716 | 0.882216 | 9.35189 | 3.38E-19 | 7.44E-18 | 32.7172 |
| EGR2 | 0.70722 | 3.588675 | 4.94819 | 1.04E-06 | 5.32E-06 | 4.58667 |
| PHACTR4 | 0.70763 | 4.273942 | 15.3067 | 2.71E-43 | 7.73E-41 | 87.6331 |
| GBGT1 | 0.70774 | 2.35815 | 12.4147 | 7.49E-31 | 5.47E-29 | 59.2432 |
| MMP9 | 0.70775 | 1.099773 | 5.68227 | 2.32E-08 | 1.48E-07 | 8.25995 |
| SOWAHD | 0.70798 | 1.390302 | 12.2218 | 4.59E-30 | 3.08E-28 | 57.4485 |
| CEBPB | 0.70802 | 4.719936 | 9.89736 | 3.98E-21 | 1.06E-19 | 37.0981 |
| TC2N | 0.70883 | 1.265399 | 8.78974 | 2.75E-17 | 4.93E-16 | 28.3829 |
| FCGR2C | 0.70911 | 0.748974 | 9.37874 | 2.72E-19 | 6.06E-18 | 32.9289 |
| HS2ST1 | 0.70939 | 4.519309 | 12.3791 | 1.05E-30 | 7.52E-29 | 58.9106 |
| AL035461.3 | 0.71007 | 2.655876 | 11.3843 | 1.01E-26 | 4.72E-25 | 49.8294 |
| CDCA7 | 0.71014 | 2.943438 | 7.51968 | 2.76E-13 | 3.23E-12 | 19.3267 |
| NCAPH | 0.71041 | 2.207485 | 6.90806 | 1.58E-11 | 1.51E-10 | 15.3606 |
| OR4K6P | 0.71047 | 0.700467 | 8.51853 | 2.14E-16 | 3.49E-15 | 26.3608 |
| COQ8B | 0.71092 | 4.525803 | 17.0967 | 1.95E-51 | 1.16E-48 | 106.228 |
| IGF2BP3 | 0.71093 | 0.474906 | 9.4616 | 1.40E-19 | 3.18E-18 | 33.5848 |
| HMGN2P5 | 0.71097 | 2.462366 | 10.6652 | 5.88E-24 | 2.08E-22 | 43.5357 |
| BMF | 0.71175 | 2.773019 | 8.90176 | 1.16E-17 | 2.17E-16 | 29.2315 |
| LINC01579 | 0.71198 | 0.97884 | 5.97626 | 4.48E-09 | 3.16E-08 | 9.85548 |
| CDCA2 | 0.71217 | 1.069682 | 9.06761 | 3.20E-18 | 6.36E-17 | 30.5017 |
| MATN2 | 0.7122 | 5.374021 | 7.20694 | 2.26E-12 | 2.39E-11 | 17.265 |
| PPIAP39 | 0.7125 | 1.342013 | 8.57435 | 1.41E-16 | 2.34E-15 | 26.7732 |
| SAA1 | 0.71281 | 0.604498 | 6.06302 | 2.72E-09 | 1.96E-08 | 10.3396 |
| GNA15 | 0.71323 | 2.772575 | 10.2667 | 1.80E-22 | 5.43E-21 | 40.1565 |
| NLRP3 | 0.71335 | 2.557558 | 9.53198 | 7.94E-20 | 1.86E-18 | 34.145 |
| EXOSC10 | 0.71341 | 4.995748 | 16.2333 | 1.77E-47 | 7.15E-45 | 97.1857 |
| TAF12 | 0.71341 | 4.495789 | 21.5026 | 3.71E-72 | 1.69E-68 | 153.549 |
| TRNAU1AP | 0.71356 | 3.498321 | 23.2138 | 2.83E-80 | 5.16E-76 | 172.08 |
| AC020916.1 | 0.71381 | 2.784596 | 8.80474 | 2.45E-17 | 4.41E-16 | 28.4961 |
| COL27A1 | 0.71394 | 1.683621 | 9.81298 | 8.00E-21 | 2.07E-19 | 36.4095 |
| MYL12A | 0.714 | 5.106896 | 10.2824 | 1.57E-22 | 4.78E-21 | 40.2883 |
| DEPDC1 | 0.71435 | 0.770522 | 9.95264 | 2.52E-21 | 6.79E-20 | 37.5512 |
| IGFBP3 | 0.71441 | 4.637248 | 4.59869 | 5.46E-06 | 2.51E-05 | 2.99849 |
| NAGA | 0.71474 | 4.731217 | 13.0571 | 1.62E-33 | 1.63E-31 | 65.3223 |
| BX284668.5 | 0.71485 | 3.060276 | 8.26561 | 1.40E-15 | 2.08E-14 | 24.5172 |
| TMSB10 | 0.7149 | 10.6154 | 10.4099 | 5.31E-23 | 1.69E-21 | 41.3614 |
| STK38 | 0.71491 | 4.644124 | 15.8359 | 1.13E-45 | 3.84E-43 | 93.0673 |
| SERPINB1 | 0.71493 | 4.0282 | 9.8319 | 6.84E-21 | 1.78E-19 | 36.5636 |
| TRAM2 | 0.71501 | 3.112836 | 10.738 | 3.12E-24 | 1.14E-22 | 44.1619 |
| CLIC4 | 0.71537 | 7.524552 | 10.3514 | 8.75E-23 | 2.73E-21 | 40.8683 |
| C4orf47 | 0.71549 | 2.174693 | 9.00628 | 5.16E-18 | 1.00E-16 | 30.03 |
| PLA2G2A | 0.71581 | 0.584454 | 5.96453 | 4.79E-09 | 3.36E-08 | 9.79049 |
| MYO7A | 0.71585 | 2.166076 | 11.845 | 1.52E-28 | 8.72E-27 | 53.984 |
| CHEK2 | 0.71666 | 1.830043 | 13.1612 | 5.90E-34 | 6.28E-32 | 66.3209 |
| LYN | 0.71705 | 3.651442 | 9.6647 | 2.70E-20 | 6.57E-19 | 35.209 |
| PHC2 | 0.71721 | 6.837571 | 16.8699 | 2.16E-50 | 1.20E-47 | 103.841 |
| ODF3B | 0.71764 | 2.261628 | 7.73971 | 6.03E-14 | 7.63E-13 | 20.8184 |
| PIM1 | 0.71776 | 4.360932 | 10.3163 | 1.18E-22 | 3.63E-21 | 40.5728 |
| TMEM234 | 0.71812 | 2.927685 | 19.7216 | 1.02E-63 | 1.86E-60 | 134.277 |
| INAFM1 | 0.71818 | 4.565241 | 10.8653 | 1.03E-24 | 3.92E-23 | 45.2624 |
| CSF2RB | 0.7182 | 1.560596 | 10.9412 | 5.26E-25 | 2.07E-23 | 45.9231 |
| CTF1 | 0.71823 | 2.549692 | 8.73714 | 4.10E-17 | 7.21E-16 | 27.9872 |
| LOXL3 | 0.71868 | 3.651295 | 10.1178 | 6.32E-22 | 1.81E-20 | 38.9147 |
| SLC25A43 | 0.71892 | 2.802187 | 7.88066 | 2.24E-14 | 2.97E-13 | 21.7915 |
| OIP5 | 0.71962 | 1.851748 | 9.29512 | 5.31E-19 | 1.14E-17 | 32.271 |
| AC025265.1 | 0.71968 | 1.415886 | 9.35041 | 3.42E-19 | 7.51E-18 | 32.7056 |
| PLAC8 | 0.71976 | 0.835576 | 10.3563 | 8.39E-23 | 2.62E-21 | 40.9096 |
| COL9A2 | 0.71982 | 5.192995 | 7.35739 | 8.28E-13 | 9.21E-12 | 18.2481 |
| TMEM51 | 0.71997 | 3.96951 | 11.4805 | 4.24E-27 | 2.07E-25 | 50.6889 |
| WASF2 | 0.72005 | 6.289113 | 13.3689 | 7.80E-35 | 9.10E-33 | 68.3245 |
| AC004687.1 | 0.72012 | 1.3203 | 10.7103 | 3.97E-24 | 1.43E-22 | 43.9238 |
| SAMD9 | 0.7203 | 2.562879 | 8.23476 | 1.75E-15 | 2.58E-14 | 24.2952 |
| HSPB1 | 0.7209 | 7.543652 | 7.22581 | 1.99E-12 | 2.13E-11 | 17.3874 |
| ERF | 0.72106 | 5.932481 | 14.2249 | 1.62E-38 | 2.87E-36 | 76.7266 |
| HLA-C | 0.7212 | 8.689781 | 8.84563 | 1.79E-17 | 3.27E-16 | 28.8053 |
| B3GNT7 | 0.72129 | 1.57878 | 7.80781 | 3.74E-14 | 4.84E-13 | 21.2869 |
| NPNT | 0.72139 | 2.616432 | 5.21321 | 2.77E-07 | 1.54E-06 | 5.86077 |
| SELENON | 0.72158 | 7.069672 | 12.8903 | 8.07E-33 | 7.37E-31 | 63.7296 |
| COL5A1 | 0.72173 | 1.499718 | 6.94715 | 1.23E-11 | 1.19E-10 | 15.606 |
| TMEM147 | 0.72189 | 6.408542 | 19.19 | 3.29E-61 | 4.62E-58 | 128.548 |
| PUSL1 | 0.7219 | 3.406002 | 17.9177 | 3.03E-55 | 2.44E-52 | 114.926 |
| RPS19 | 0.72192 | 8.518792 | 12.866 | 1.02E-32 | 9.09E-31 | 63.4982 |
| KIAA2013 | 0.72242 | 5.605316 | 17.9135 | 3.17E-55 | 2.51E-52 | 114.882 |
| IRF1 | 0.7225 | 2.779637 | 9.5022 | 1.01E-19 | 2.33E-18 | 33.9076 |
| CORO1A | 0.72263 | 5.120019 | 10.7798 | 2.17E-24 | 7.97E-23 | 44.5222 |
| PRKAR1B-AS1 | 0.72314 | 1.581204 | 10.8126 | 1.63E-24 | 6.07E-23 | 44.8061 |
| PHEX | 0.72318 | 1.573053 | 10.3428 | 9.42E-23 | 2.92E-21 | 40.7958 |
| SCN7A | 0.7234 | 1.133549 | 6.90179 | 1.65E-11 | 1.57E-10 | 15.3214 |
| WNT5A-AS1 | 0.72341 | 1.638604 | 9.73525 | 1.52E-20 | 3.81E-19 | 35.7787 |
| FGFRL1 | 0.72355 | 4.051413 | 7.492 | 3.33E-13 | 3.86E-12 | 19.1414 |
| CKAP2L | 0.7236 | 1.463633 | 7.30731 | 1.16E-12 | 1.27E-11 | 17.9191 |
| WDR77 | 0.72404 | 4.678644 | 20.0057 | 4.62E-65 | 9.05E-62 | 137.345 |
| PCLAF | 0.72433 | 1.758283 | 7.17244 | 2.84E-12 | 2.96E-11 | 17.0418 |
| GPR82 | 0.72436 | 0.811445 | 9.77174 | 1.12E-20 | 2.86E-19 | 36.0744 |
| HOXA11 | 0.7245 | 0.589747 | 7.95479 | 1.32E-14 | 1.79E-13 | 22.3087 |
| TNFAIP2 | 0.72479 | 3.468932 | 8.49168 | 2.62E-16 | 4.23E-15 | 26.1632 |
| SPA17 | 0.7252 | 2.50747 | 10.1031 | 7.15E-22 | 2.03E-20 | 38.7932 |
| SNHG9 | 0.7258 | 4.614519 | 11.529 | 2.73E-27 | 1.36E-25 | 51.1247 |
| PLK3 | 0.72588 | 3.334489 | 12.2035 | 5.44E-30 | 3.62E-28 | 57.2789 |
| SOX9 | 0.72595 | 7.312714 | 9.94408 | 2.70E-21 | 7.27E-20 | 37.4809 |
| PPIH | 0.72602 | 5.063649 | 22.0761 | 7.02E-75 | 6.41E-71 | 159.764 |
| RNF149 | 0.72607 | 3.483892 | 12.2034 | 5.45E-30 | 3.62E-28 | 57.2779 |
| F3 | 0.72625 | 5.806918 | 5.81716 | 1.10E-08 | 7.35E-08 | 8.98339 |
| YAP1 | 0.72677 | 4.301924 | 7.83478 | 3.09E-14 | 4.04E-13 | 21.4733 |
| ACTN1 | 0.72688 | 4.416941 | 6.78859 | 3.38E-11 | 3.09E-10 | 14.6178 |
| PLIN3 | 0.72717 | 6.107386 | 10.4301 | 4.47E-23 | 1.44E-21 | 41.5327 |
| RAB13 | 0.72721 | 6.344701 | 12.1845 | 6.51E-30 | 4.28E-28 | 57.1024 |
| GFAP | 0.72723 | 12.9259 | 5.80874 | 1.15E-08 | 7.68E-08 | 8.93781 |
| FCGR1B | 0.72729 | 1.855783 | 10.1364 | 5.41E-22 | 1.55E-20 | 39.0691 |
| CDK1 | 0.72743 | 2.588331 | 6.18858 | 1.31E-09 | 9.81E-09 | 11.0507 |
| PIK3AP1 | 0.72785 | 3.181948 | 9.17601 | 1.36E-18 | 2.81E-17 | 31.3409 |
| IGHG1 | 0.72794 | 1.266872 | 4.56849 | 6.27E-06 | 2.86E-05 | 2.86622 |
| GIMAP2 | 0.72801 | 3.229297 | 10.3274 | 1.07E-22 | 3.31E-21 | 40.6663 |
| AC009812.4 | 0.72869 | 2.205982 | 14.0266 | 1.18E-37 | 1.87E-35 | 74.761 |
| NEK2 | 0.72888 | 1.569797 | 7.30935 | 1.14E-12 | 1.25E-11 | 17.9324 |
| VAV3 | 0.729 | 1.197098 | 6.97723 | 1.01E-11 | 9.88E-11 | 15.7956 |
| APOL1 | 0.72913 | 3.286158 | 8.01313 | 8.70E-15 | 1.20E-13 | 22.7183 |
| AGAP2-AS1 | 0.72918 | 2.083904 | 6.59437 | 1.14E-10 | 9.70E-10 | 13.433 |
| ST8SIA4 | 0.72975 | 2.297743 | 11.1194 | 1.09E-25 | 4.58E-24 | 47.4831 |
| IQGAP3 | 0.72981 | 1.61023 | 7.47648 | 3.70E-13 | 4.28E-12 | 19.0378 |
| HAND2 | 0.72987 | 0.571387 | 9.07513 | 3.01E-18 | 6.01E-17 | 30.5597 |
| PRPF31 | 0.7308 | 5.810295 | 19.0177 | 2.13E-60 | 2.65E-57 | 126.696 |
| ICAM1 | 0.7309 | 3.081381 | 6.96526 | 1.10E-11 | 1.06E-10 | 15.72 |
| RAD51 | 0.73169 | 1.586038 | 9.57503 | 5.60E-20 | 1.33E-18 | 34.4891 |
| SDF4 | 0.73183 | 6.05626 | 20.6728 | 3.20E-68 | 8.77E-65 | 144.56 |
| LGR6 | 0.73194 | 1.218821 | 5.18002 | 3.28E-07 | 1.80E-06 | 5.69795 |
| CAVIN1 | 0.73218 | 5.539558 | 7.73151 | 6.38E-14 | 8.05E-13 | 20.7622 |
| LAMA4 | 0.73258 | 3.485331 | 9.17185 | 1.41E-18 | 2.90E-17 | 31.3086 |
| HLA-A | 0.7327 | 8.894674 | 9.70869 | 1.88E-20 | 4.69E-19 | 35.5639 |
| TRIT1 | 0.73289 | 4.249864 | 14.663 | 1.95E-40 | 4.15E-38 | 81.1071 |
| AC011445.2 | 0.73297 | 2.977958 | 9.42011 | 1.96E-19 | 4.40E-18 | 33.2559 |
| HPSE | 0.73308 | 2.431372 | 8.88279 | 1.34E-17 | 2.49E-16 | 29.0872 |
| PALD1 | 0.73325 | 5.617102 | 12.536 | 2.38E-31 | 1.84E-29 | 60.3796 |
| MAP1LC3C | 0.73346 | 0.843333 | 7.37446 | 7.38E-13 | 8.26E-12 | 18.3607 |
| PARP9 | 0.73354 | 3.725618 | 9.54738 | 7.01E-20 | 1.65E-18 | 34.268 |
| JPT2 | 0.73397 | 4.360031 | 12.382 | 1.02E-30 | 7.34E-29 | 58.9376 |
| LTBR | 0.73406 | 2.807548 | 11.8979 | 9.33E-29 | 5.46E-27 | 54.4668 |
| PTOV1-AS2 | 0.73458 | 3.040735 | 9.7119 | 1.84E-20 | 4.57E-19 | 35.5898 |
| CMTM3 | 0.7346 | 5.542587 | 11.3205 | 1.80E-26 | 8.15E-25 | 49.2616 |
| AL021807.1 | 0.73464 | 1.048309 | 12.8694 | 9.87E-33 | 8.83E-31 | 63.5304 |
| CAPS | 0.73464 | 3.97109 | 9.11993 | 2.12E-18 | 4.30E-17 | 30.9059 |
| CD300LF | 0.735 | 1.60937 | 10.183 | 3.65E-22 | 1.07E-20 | 39.4574 |
| MICB | 0.73516 | 1.593246 | 11.4828 | 4.16E-27 | 2.02E-25 | 50.71 |
| FOS | 0.7354 | 7.074231 | 5.1462 | 3.89E-07 | 2.11E-06 | 5.533 |
| ST20 | 0.73543 | 2.138802 | 12.1836 | 6.56E-30 | 4.30E-28 | 57.0939 |
| GIMAP4 | 0.73577 | 4.797087 | 10.3891 | 6.34E-23 | 2.01E-21 | 41.186 |
| ARMH1 | 0.73592 | 1.975882 | 11.9714 | 4.72E-29 | 2.86E-27 | 55.1401 |
| GPC4 | 0.73676 | 4.344606 | 9.00466 | 5.22E-18 | 1.02E-16 | 30.0176 |
| HLA-H | 0.73757 | 5.14868 | 8.35614 | 7.17E-16 | 1.11E-14 | 25.1723 |
| TOR4A | 0.73806 | 2.324142 | 13.6489 | 4.99E-36 | 6.62E-34 | 71.0489 |
| HSPB11 | 0.73816 | 3.544903 | 21.6227 | 9.96E-73 | 5.80E-69 | 154.851 |
| FPGT | 0.73855 | 2.89772 | 14.0062 | 1.45E-37 | 2.25E-35 | 74.5592 |
| GLMP | 0.739 | 3.826225 | 11.5676 | 1.93E-27 | 9.79E-26 | 51.471 |
| NINJ1 | 0.74008 | 6.154517 | 10.6852 | 4.94E-24 | 1.77E-22 | 43.7074 |
| TUSC1 | 0.74018 | 3.980035 | 9.08402 | 2.81E-18 | 5.62E-17 | 30.6283 |
| RNF122 | 0.74094 | 3.934552 | 9.39696 | 2.35E-19 | 5.26E-18 | 33.0728 |
| EEF1AKMT3 | 0.74156 | 3.064262 | 9.60888 | 4.26E-20 | 1.02E-18 | 34.7603 |
| TUBA1C | 0.74161 | 3.187109 | 8.54778 | 1.72E-16 | 2.84E-15 | 26.5767 |
| ALDH4A1 | 0.74213 | 5.949334 | 9.76295 | 1.21E-20 | 3.07E-19 | 36.0031 |
| GPNMB | 0.74215 | 3.645654 | 4.90325 | 1.30E-06 | 6.53E-06 | 4.37659 |
| S100A9 | 0.7423 | 4.202804 | 4.66054 | 4.10E-06 | 1.92E-05 | 3.27185 |
| LILRA1 | 0.74266 | 1.699908 | 10.9413 | 5.26E-25 | 2.07E-23 | 45.9233 |
| IL1B | 0.74271 | 2.624363 | 5.10583 | 4.77E-07 | 2.56E-06 | 5.33734 |
| ARHGAP25 | 0.74282 | 2.7453 | 11.6651 | 7.91E-28 | 4.20E-26 | 52.3512 |
| SCGB1D2 | 0.7429 | 1.214822 | 6.62444 | 9.45E-11 | 8.15E-10 | 13.6146 |
| CEP89 | 0.7429 | 3.263055 | 19.0123 | 2.25E-60 | 2.75E-57 | 126.638 |
| SOCS2 | 0.74318 | 2.488652 | 7.57285 | 1.91E-13 | 2.28E-12 | 19.6841 |
| DDR2 | 0.74319 | 3.757018 | 6.55807 | 1.42E-10 | 1.20E-09 | 13.2148 |
| DLGAP1-AS2 | 0.74337 | 1.982527 | 11.5769 | 1.77E-27 | 9.03E-26 | 51.5553 |
| PSENEN | 0.74379 | 5.749112 | 14.9288 | 1.31E-41 | 3.04E-39 | 83.79 |
| NPC2 | 0.74477 | 6.573277 | 11.2743 | 2.72E-26 | 1.21E-24 | 48.8512 |
| OSMR | 0.74537 | 2.944291 | 7.66703 | 9.99E-14 | 1.23E-12 | 20.3219 |
| IFI16 | 0.74578 | 5.159343 | 9.99804 | 1.72E-21 | 4.73E-20 | 37.9246 |
| ARHGAP45 | 0.7464 | 4.02036 | 13.6947 | 3.18E-36 | 4.29E-34 | 71.496 |
| HSPA1A | 0.74667 | 5.803533 | 7.64263 | 1.18E-13 | 1.44E-12 | 20.1561 |
| DNAAF3 | 0.74709 | 0.86839 | 11.2865 | 2.44E-26 | 1.09E-24 | 48.9592 |
| RBP1 | 0.74718 | 3.253077 | 5.18577 | 3.19E-07 | 1.75E-06 | 5.72608 |
| ITGB3BP | 0.74774 | 2.357006 | 15.3296 | 2.14E-43 | 6.24E-41 | 87.8664 |
| CD33 | 0.74778 | 1.684084 | 12.7509 | 3.07E-32 | 2.56E-30 | 62.4062 |
| P2RY13 | 0.74785 | 3.879016 | 7.82432 | 3.33E-14 | 4.33E-13 | 21.4009 |
| GNAI3 | 0.74832 | 3.337705 | 17.6491 | 5.38E-54 | 3.83E-51 | 112.071 |
| PILRA | 0.74921 | 3.588142 | 12.1689 | 7.53E-30 | 4.91E-28 | 56.958 |
| PDLIM1 | 0.74994 | 2.975107 | 6.71098 | 5.51E-11 | 4.90E-10 | 14.1409 |
| SLAMF8 | 0.75002 | 1.763949 | 7.96739 | 1.21E-14 | 1.64E-13 | 22.397 |
| FGD2 | 0.75053 | 2.253617 | 10.7683 | 2.40E-24 | 8.77E-23 | 44.4234 |
| GPX1 | 0.75075 | 8.650611 | 13.9839 | 1.81E-37 | 2.79E-35 | 74.3386 |
| TNFSF13B | 0.75161 | 3.11249 | 5.59045 | 3.82E-08 | 2.38E-07 | 7.77603 |
| ATAD3A | 0.75178 | 4.568618 | 18.5498 | 3.35E-58 | 3.68E-55 | 121.677 |
| AC133065.3 | 0.75201 | 1.853081 | 10.8396 | 1.28E-24 | 4.85E-23 | 45.0396 |
| WDR62 | 0.75204 | 1.346531 | 9.89189 | 4.17E-21 | 1.10E-19 | 37.0532 |
| PDIA4 | 0.75261 | 6.465939 | 11.5442 | 2.38E-27 | 1.20E-25 | 51.2605 |
| GDF15 | 0.75321 | 1.280611 | 6.90793 | 1.58E-11 | 1.51E-10 | 15.3598 |
| STAC3 | 0.75331 | 2.769661 | 13.26 | 2.26E-34 | 2.55E-32 | 67.2723 |
| TMEM176A | 0.75343 | 3.99336 | 6.87804 | 1.92E-11 | 1.81E-10 | 15.1729 |
| LSM10 | 0.75344 | 5.68984 | 22.1413 | 3.44E-75 | 3.77E-71 | 160.471 |
| WWTR1 | 0.75374 | 4.407913 | 7.31867 | 1.07E-12 | 1.18E-11 | 17.9936 |
| TXNDC12 | 0.7542 | 5.665827 | 19.644 | 2.37E-63 | 4.06E-60 | 133.44 |
| EVA1B | 0.75427 | 3.18746 | 11.8261 | 1.81E-28 | 1.03E-26 | 53.8118 |
| AL133415.1 | 0.75475 | 0.793378 | 13.1099 | 9.70E-34 | 1.01E-31 | 65.828 |
| HOXA4 | 0.75482 | 0.453057 | 7.93938 | 1.47E-14 | 1.99E-13 | 22.2009 |
| SHMT1 | 0.7549 | 3.071937 | 15.6456 | 8.15E-45 | 2.61E-42 | 91.1066 |
| AL390755.1 | 0.755 | 3.95202 | 5.49595 | 6.35E-08 | 3.84E-07 | 7.28516 |
| CYP21A2 | 0.75521 | 1.270771 | 9.95437 | 2.48E-21 | 6.69E-20 | 37.5654 |
| SRSF4 | 0.75525 | 6.341374 | 20.7302 | 1.71E-68 | 4.93E-65 | 145.182 |
| EMILIN2 | 0.75543 | 1.742383 | 7.84084 | 2.96E-14 | 3.88E-13 | 21.5152 |
| B9D2 | 0.75571 | 3.331013 | 13.925 | 3.24E-37 | 4.86E-35 | 73.7582 |
| AC093157.1 | 0.75669 | 3.051075 | 15.6003 | 1.30E-44 | 4.15E-42 | 90.6406 |
| CARD11 | 0.75694 | 2.584112 | 11.3915 | 9.48E-27 | 4.43E-25 | 49.894 |
| SLC2A4RG | 0.75743 | 4.670801 | 14.0564 | 8.76E-38 | 1.43E-35 | 75.0551 |
| SIX5 | 0.75795 | 1.823337 | 13.1205 | 8.75E-34 | 9.20E-32 | 65.9303 |
| EGR1 | 0.75807 | 6.847936 | 4.85561 | 1.63E-06 | 8.09E-06 | 4.15571 |
| CHST9 | 0.75825 | 3.50663 | 5.85408 | 8.95E-09 | 6.06E-08 | 9.18391 |
| APOBEC3G | 0.75839 | 1.648485 | 11.7748 | 2.90E-28 | 1.61E-26 | 53.3453 |
| MORN5 | 0.75886 | 2.602441 | 8.6562 | 7.59E-17 | 1.30E-15 | 27.3815 |
| AQP5 | 0.75947 | 0.912133 | 6.58819 | 1.18E-10 | 1.01E-09 | 13.3958 |
| SNX20 | 0.7597 | 1.179909 | 12.3593 | 1.26E-30 | 8.97E-29 | 58.7267 |
| SZRD1 | 0.75984 | 5.88083 | 19.5629 | 5.73E-63 | 8.97E-60 | 132.565 |
| DPYD | 0.76009 | 2.458752 | 8.50771 | 2.32E-16 | 3.77E-15 | 26.2811 |
| NFKBIB | 0.76073 | 5.138593 | 16.3932 | 3.31E-48 | 1.42E-45 | 98.8517 |
| AC099850.4 | 0.76108 | 3.04298 | 8.4801 | 2.86E-16 | 4.59E-15 | 26.0781 |
| PROSER3 | 0.76109 | 2.259628 | 14.6551 | 2.12E-40 | 4.46E-38 | 81.0281 |
| LCP2 | 0.76111 | 2.996934 | 10.5646 | 1.40E-23 | 4.76E-22 | 42.6758 |
| SERINC2 | 0.7618 | 1.985175 | 8.12514 | 3.88E-15 | 5.54E-14 | 23.5112 |
| DUSP10 | 0.76188 | 3.005613 | 9.66615 | 2.67E-20 | 6.50E-19 | 35.2208 |
| RBM42 | 0.76191 | 6.538709 | 18.6322 | 1.38E-58 | 1.57E-55 | 122.559 |
| SLC30A7 | 0.76219 | 2.756453 | 16.5363 | 7.33E-49 | 3.29E-46 | 100.346 |
| MARVELD1 | 0.76228 | 3.046783 | 8.94937 | 8.03E-18 | 1.53E-16 | 29.5944 |
| HAUS5 | 0.76262 | 3.584366 | 15.117 | 1.90E-42 | 4.82E-40 | 85.6996 |
| BUB1 | 0.76325 | 1.783074 | 7.71169 | 7.33E-14 | 9.17E-13 | 20.6266 |
| SNAPC2 | 0.76355 | 4.816389 | 16.7628 | 6.71E-50 | 3.50E-47 | 102.718 |
| CENPH | 0.76378 | 3.175401 | 11.3428 | 1.47E-26 | 6.72E-25 | 49.4593 |
| WDFY4 | 0.76411 | 1.876494 | 11.4141 | 7.73E-27 | 3.64E-25 | 50.0955 |
| CCL5 | 0.7648 | 2.173303 | 7.13439 | 3.65E-12 | 3.76E-11 | 16.7967 |
| CSTA | 0.76489 | 1.192458 | 8.72037 | 4.66E-17 | 8.15E-16 | 27.8614 |
| PPIAP48 | 0.7651 | 0.84981 | 10.2689 | 1.76E-22 | 5.34E-21 | 40.1753 |
| SLC6A9 | 0.76625 | 5.290717 | 7.8147 | 3.56E-14 | 4.62E-13 | 21.3344 |
| TTC26 | 0.76634 | 2.291466 | 12.8172 | 1.63E-32 | 1.41E-30 | 63.0351 |
| ELK3 | 0.76657 | 4.051212 | 11.0565 | 1.90E-25 | 7.86E-24 | 46.9308 |
| ANXA2R | 0.76692 | 2.070714 | 12.3115 | 1.98E-30 | 1.37E-28 | 58.2811 |
| NASP | 0.76737 | 5.475785 | 15.4542 | 5.91E-44 | 1.78E-41 | 89.1414 |
| NRROS | 0.76741 | 3.058001 | 9.66456 | 2.70E-20 | 6.58E-19 | 35.2079 |
| AC138207.3 | 0.76742 | 3.220312 | 7.01034 | 8.19E-12 | 8.07E-11 | 16.005 |
| B3GALT5-AS1 | 0.76748 | 1.704369 | 11.0176 | 2.68E-25 | 1.09E-23 | 46.59 |
| SHCBP1 | 0.7675 | 1.78694 | 9.49381 | 1.08E-19 | 2.49E-18 | 33.8409 |
| SKA1 | 0.76764 | 1.273558 | 9.06081 | 3.37E-18 | 6.69E-17 | 30.4493 |
| IRF3 | 0.76772 | 4.724213 | 18.0519 | 7.16E-56 | 6.43E-53 | 116.357 |
| HOXC10 | 0.76786 | 0.49417 | 7.17712 | 2.75E-12 | 2.88E-11 | 17.0721 |
| DMPK | 0.76962 | 5.228262 | 12.8291 | 1.45E-32 | 1.26E-30 | 63.1481 |
| AC080038.2 | 0.76995 | 1.664674 | 10.3744 | 7.19E-23 | 2.27E-21 | 41.0619 |
| KDELR1 | 0.76997 | 6.831885 | 17.9867 | 1.44E-55 | 1.22E-52 | 115.661 |
| NCF1 | 0.77 | 1.282392 | 14.2902 | 8.42E-39 | 1.55E-36 | 77.3763 |
| SP140L | 0.77006 | 1.759089 | 13.0522 | 1.69E-33 | 1.70E-31 | 65.2751 |
| RPF1 | 0.77009 | 5.370032 | 21.4308 | 8.12E-72 | 3.42E-68 | 152.771 |
| H6PD | 0.77021 | 4.408004 | 15.4674 | 5.15E-44 | 1.57E-41 | 89.2775 |
| FHOD1 | 0.77028 | 3.037398 | 14.8706 | 2.36E-41 | 5.31E-39 | 83.201 |
| PDLIM7 | 0.77115 | 3.713666 | 11.963 | 5.11E-29 | 3.08E-27 | 55.0626 |
| HPD | 0.7715 | 0.899908 | 9.16861 | 1.45E-18 | 2.97E-17 | 31.2834 |
| EZH2 | 0.77151 | 3.368986 | 8.37553 | 6.21E-16 | 9.65E-15 | 25.3134 |
| H2AX | 0.77213 | 6.661801 | 13.406 | 5.43E-35 | 6.45E-33 | 68.6842 |
| IKZF1 | 0.77258 | 2.087596 | 10.8291 | 1.41E-24 | 5.30E-23 | 44.9491 |
| BCL3 | 0.77272 | 2.768499 | 12.4289 | 6.55E-31 | 4.83E-29 | 59.376 |
| LINC01355 | 0.77274 | 2.012224 | 11.9443 | 6.08E-29 | 3.62E-27 | 54.891 |
| LINC01480 | 0.77306 | 2.241574 | 9.10253 | 2.43E-18 | 4.90E-17 | 30.7713 |
| RSRP1 | 0.77376 | 4.281469 | 11.0725 | 1.65E-25 | 6.86E-24 | 47.0709 |
| PRKD2 | 0.77382 | 3.822406 | 16.4981 | 1.10E-48 | 4.85E-46 | 99.9466 |
| LTBP1 | 0.77386 | 3.836655 | 7.88945 | 2.10E-14 | 2.80E-13 | 21.8527 |
| CCDC40 | 0.77421 | 2.069856 | 11.2588 | 3.13E-26 | 1.39E-24 | 48.7139 |
| NRM | 0.77433 | 4.086687 | 11.2206 | 4.40E-26 | 1.92E-24 | 48.3756 |
| TENT5A | 0.77452 | 2.684452 | 9.92819 | 3.08E-21 | 8.26E-20 | 37.3506 |
| RN7SL138P | 0.77458 | 2.202514 | 12.2404 | 3.85E-30 | 2.60E-28 | 57.6209 |
| SNRNP40 | 0.77514 | 4.452692 | 21.6172 | 1.06E-72 | 5.80E-69 | 154.791 |
| HMG20B | 0.77583 | 5.341955 | 16.5974 | 3.85E-49 | 1.79E-46 | 100.984 |
| HHEX | 0.77598 | 2.567443 | 12.6051 | 1.23E-31 | 9.75E-30 | 61.0295 |
| GPX8 | 0.77599 | 1.115361 | 8.27654 | 1.29E-15 | 1.93E-14 | 24.596 |
| LINC02587 | 0.7761 | 0.882262 | 5.5023 | 6.13E-08 | 3.72E-07 | 7.31795 |
| OSCAR | 0.77643 | 2.526098 | 10.8084 | 1.69E-24 | 6.30E-23 | 44.7696 |
| H3C6 | 0.77655 | 1.595 | 8.63914 | 8.64E-17 | 1.47E-15 | 27.2544 |
| PLVAP | 0.77707 | 3.975636 | 8.29876 | 1.09E-15 | 1.65E-14 | 24.7565 |
| CYP21A1P | 0.77724 | 1.764376 | 8.76297 | 3.37E-17 | 5.98E-16 | 28.1813 |
| SOD2 | 0.77822 | 5.461826 | 7.49141 | 3.34E-13 | 3.88E-12 | 19.1375 |
| NMB | 0.7783 | 7.849377 | 5.23063 | 2.54E-07 | 1.41E-06 | 5.94661 |
| RBMS1 | 0.77892 | 2.529808 | 13.2053 | 3.84E-34 | 4.20E-32 | 66.7458 |
| PHTF1 | 0.77926 | 2.668078 | 15.8047 | 1.56E-45 | 5.24E-43 | 92.7461 |
| ZFP36L2 | 0.77932 | 6.850456 | 11.6885 | 6.39E-28 | 3.42E-26 | 52.5626 |
| LAMB2 | 0.77973 | 6.100903 | 12.885 | 8.49E-33 | 7.71E-31 | 63.6788 |
| ARC | 0.78012 | 5.013251 | 5.53165 | 5.24E-08 | 3.21E-07 | 7.46973 |
| MAOB | 0.78016 | 5.838347 | 4.11822 | 4.50E-05 | 0.000179 | 0.98971 |
| CASP6 | 0.78079 | 2.930207 | 13.5394 | 1.47E-35 | 1.83E-33 | 69.98 |
| HOXA7 | 0.78129 | 0.491356 | 8.58054 | 1.34E-16 | 2.24E-15 | 26.8191 |
| IFNLR1 | 0.7822 | 1.607526 | 10.7626 | 2.52E-24 | 9.20E-23 | 44.3745 |
| KCNK13 | 0.78262 | 1.923852 | 11.4376 | 6.26E-27 | 2.99E-25 | 50.305 |
| RNASE3 | 0.78306 | 1.357713 | 9.54378 | 7.22E-20 | 1.70E-18 | 34.2392 |
| SIRPB2 | 0.78353 | 1.65268 | 11.5941 | 1.51E-27 | 7.78E-26 | 51.7096 |
| NCF1C | 0.78369 | 1.439643 | 13.2294 | 3.04E-34 | 3.37E-32 | 66.9772 |
| FTL | 0.78483 | 12.01203 | 11.8302 | 1.74E-28 | 9.95E-27 | 53.8491 |
| UPP1 | 0.78516 | 3.222573 | 10.5985 | 1.05E-23 | 3.60E-22 | 42.9652 |
| PTPN7 | 0.78528 | 1.026359 | 12.7193 | 4.15E-32 | 3.41E-30 | 62.1071 |
| PPCS | 0.78574 | 4.055943 | 11.1209 | 1.07E-25 | 4.52E-24 | 47.4963 |
| GFPT2 | 0.78615 | 4.744323 | 8.04516 | 6.92E-15 | 9.63E-14 | 22.9442 |
| P3H1 | 0.78619 | 3.008683 | 17.7167 | 2.61E-54 | 1.93E-51 | 112.789 |
| LMNB1 | 0.78621 | 4.247986 | 8.02247 | 8.14E-15 | 1.12E-13 | 22.7841 |
| EMC1-AS1 | 0.78642 | 2.756646 | 16.7595 | 6.95E-50 | 3.56E-47 | 102.683 |
| TP53I13 | 0.78658 | 3.820531 | 15.1656 | 1.16E-42 | 3.04E-40 | 86.1939 |
| GNL2 | 0.78662 | 4.262762 | 21.5094 | 3.44E-72 | 1.69E-68 | 153.623 |
| HLA-DQB2 | 0.78693 | 1.721505 | 6.20333 | 1.20E-09 | 9.03E-09 | 11.1351 |
| RAB42 | 0.78703 | 1.54573 | 10.5008 | 2.43E-23 | 8.07E-22 | 42.1323 |
| EVA1C | 0.78746 | 2.853263 | 9.65625 | 2.89E-20 | 7.01E-19 | 35.141 |
| LINC01176 | 0.78832 | 2.573758 | 11.6501 | 9.07E-28 | 4.77E-26 | 52.2156 |
| AL355488.1 | 0.78861 | 2.233127 | 12.44 | 5.89E-31 | 4.38E-29 | 59.48 |
| H2BC11 | 0.78866 | 1.262854 | 12.9792 | 3.43E-33 | 3.31E-31 | 64.5775 |
| NLRC5 | 0.78891 | 2.486734 | 11.5575 | 2.11E-27 | 1.07E-25 | 51.3805 |
| PRDX1 | 0.78892 | 8.531312 | 9.05938 | 3.41E-18 | 6.76E-17 | 30.4383 |
| GJA1 | 0.78895 | 8.550875 | 5.89818 | 6.98E-09 | 4.79E-08 | 9.42491 |
| PLXDC2 | 0.78957 | 4.135984 | 9.01106 | 4.97E-18 | 9.69E-17 | 30.0667 |
| PLK1 | 0.79079 | 2.637768 | 9.78455 | 1.01E-20 | 2.59E-19 | 36.1784 |
| ANXA2P2 | 0.79081 | 1.262584 | 9.77727 | 1.07E-20 | 2.74E-19 | 36.1193 |
| CARD9 | 0.79215 | 1.953083 | 14.5201 | 8.30E-40 | 1.65E-37 | 79.6732 |
| PTX3 | 0.79287 | 1.996733 | 7.19607 | 2.43E-12 | 2.56E-11 | 17.1946 |
| GTSE1 | 0.79436 | 1.879185 | 7.64567 | 1.16E-13 | 1.41E-12 | 20.1767 |
| FAM20C | 0.79453 | 5.258398 | 11.3276 | 1.69E-26 | 7.69E-25 | 49.3248 |
| ANXA5 | 0.79477 | 7.942014 | 10.8801 | 9.00E-25 | 3.47E-23 | 45.3916 |
| RNU6-8 | 0.79501 | 2.407563 | 7.64619 | 1.15E-13 | 1.41E-12 | 20.1803 |
| FZD2 | 0.79509 | 2.537244 | 10.2917 | 1.45E-22 | 4.43E-21 | 40.3662 |
| SPAG1 | 0.79527 | 2.618436 | 11.2764 | 2.67E-26 | 1.19E-24 | 48.8695 |
| HCG22 | 0.79563 | 1.704931 | 7.81563 | 3.54E-14 | 4.59E-13 | 21.3409 |
| LINC02828 | 0.79568 | 1.496242 | 11.5216 | 2.92E-27 | 1.45E-25 | 51.0578 |
| RAD51AP1 | 0.79593 | 2.390513 | 9.06996 | 3.14E-18 | 6.25E-17 | 30.5199 |
| C1RL | 0.79613 | 2.788454 | 8.54075 | 1.81E-16 | 2.98E-15 | 26.5247 |
| GINS2 | 0.79625 | 2.548089 | 9.98256 | 1.96E-21 | 5.34E-20 | 37.7972 |
| CPVL | 0.79654 | 4.229588 | 7.68263 | 8.97E-14 | 1.11E-12 | 20.4282 |
| BATF | 0.79704 | 1.28247 | 10.744 | 2.96E-24 | 1.08E-22 | 44.2137 |
| CAV1 | 0.79738 | 4.267502 | 6.74715 | 4.39E-11 | 3.95E-10 | 14.3626 |
| CASP4 | 0.79918 | 1.777904 | 12.4186 | 7.21E-31 | 5.29E-29 | 59.2797 |
| FOSL1 | 0.79923 | 1.883727 | 8.02346 | 8.08E-15 | 1.12E-13 | 22.7911 |
| CCDC18 | 0.80068 | 1.288294 | 17.1494 | 1.11E-51 | 6.70E-49 | 106.784 |
| S100A2 | 0.80133 | 1.975604 | 10.6981 | 4.42E-24 | 1.59E-22 | 43.819 |
| PRR11 | 0.80207 | 2.816904 | 10.8963 | 7.81E-25 | 3.03E-23 | 45.5317 |
| KANSL1L | 0.80229 | 2.826482 | 12.2881 | 2.47E-30 | 1.69E-28 | 58.0632 |
| PLOD2 | 0.80296 | 3.967081 | 11.1842 | 6.09E-26 | 2.63E-24 | 48.0537 |
| TMEM156 | 0.80308 | 2.185486 | 10.8483 | 1.19E-24 | 4.51E-23 | 45.115 |
| FAM229A | 0.80374 | 2.273258 | 13.2663 | 2.12E-34 | 2.41E-32 | 67.3338 |
| LIF | 0.80409 | 1.160317 | 7.8017 | 3.90E-14 | 5.04E-13 | 21.2447 |
| SLC4A2 | 0.80458 | 4.936274 | 14.3336 | 5.44E-39 | 1.01E-36 | 77.809 |
| NFATC2 | 0.80569 | 2.362081 | 11.7241 | 4.61E-28 | 2.52E-26 | 52.8851 |
| MUC1 | 0.80583 | 2.318378 | 12.0414 | 2.47E-29 | 1.54E-27 | 55.7825 |
| FAM83D | 0.80603 | 1.78354 | 8.70087 | 5.41E-17 | 9.40E-16 | 27.7153 |
| GALM | 0.80672 | 3.22416 | 12.887 | 8.33E-33 | 7.60E-31 | 63.698 |
| CEP55 | 0.80679 | 1.43643 | 8.79308 | 2.68E-17 | 4.81E-16 | 28.4081 |
| CDC45 | 0.80796 | 1.87498 | 7.85495 | 2.68E-14 | 3.53E-13 | 21.613 |
| KIF23 | 0.80797 | 1.357645 | 9.05681 | 3.48E-18 | 6.89E-17 | 30.4185 |
| SERPINA5 | 0.80901 | 0.868752 | 7.88794 | 2.12E-14 | 2.83E-13 | 21.8421 |
| SKAP2 | 0.80941 | 3.553385 | 8.45484 | 3.45E-16 | 5.49E-15 | 25.8927 |
| TMEM106A | 0.80944 | 2.224408 | 12.507 | 3.13E-31 | 2.39E-29 | 60.1079 |
| LDHA | 0.81014 | 5.926185 | 10.4557 | 3.59E-23 | 1.17E-21 | 41.7493 |
| SLC16A4 | 0.81036 | 3.460618 | 9.54622 | 7.08E-20 | 1.66E-18 | 34.2587 |
| LOX | 0.81089 | 1.522183 | 10.4245 | 4.68E-23 | 1.50E-21 | 41.4855 |
| H1-2 | 0.81108 | 5.297692 | 9.00835 | 5.08E-18 | 9.89E-17 | 30.0459 |
| C7orf31 | 0.81125 | 1.981097 | 13.2112 | 3.63E-34 | 3.98E-32 | 66.8017 |
| MACORIS | 0.81132 | 3.503467 | 9.57348 | 5.67E-20 | 1.35E-18 | 34.4767 |
| AJUBA | 0.81142 | 2.011164 | 12.9081 | 6.80E-33 | 6.32E-31 | 63.8995 |
| ARHGEF1 | 0.81172 | 4.577698 | 14.9205 | 1.42E-41 | 3.26E-39 | 83.7059 |
| NADK | 0.8118 | 4.799937 | 18.9001 | 7.60E-60 | 9.06E-57 | 125.432 |
| TNFAIP6 | 0.81196 | 1.729264 | 7.25368 | 1.66E-12 | 1.78E-11 | 17.5687 |
| NIBAN1 | 0.81272 | 2.36081 | 8.40125 | 5.13E-16 | 8.03E-15 | 25.5008 |
| AC027307.2 | 0.81353 | 3.273881 | 14.2829 | 9.07E-39 | 1.65E-36 | 77.3034 |
| POLD1 | 0.81362 | 3.427404 | 14.7153 | 1.15E-40 | 2.47E-38 | 81.6343 |
| ADA2 | 0.81459 | 3.862322 | 10.1286 | 5.77E-22 | 1.66E-20 | 39.0045 |
| GSDMD | 0.81528 | 3.165606 | 11.1929 | 5.64E-26 | 2.45E-24 | 48.1304 |
| FPR3 | 0.81553 | 2.163526 | 7.1222 | 3.95E-12 | 4.06E-11 | 16.7184 |
| IRX5 | 0.81585 | 0.731293 | 10.9792 | 3.76E-25 | 1.51E-23 | 46.2544 |
| ARHGAP30 | 0.8163 | 3.399702 | 11.5491 | 2.28E-27 | 1.15E-25 | 51.305 |
| TLR5 | 0.81661 | 2.056814 | 11.4829 | 4.15E-27 | 2.02E-25 | 50.711 |
| PLEKHG2 | 0.81695 | 3.523084 | 12.2776 | 2.72E-30 | 1.86E-28 | 57.9656 |
| HMGN2 | 0.81736 | 7.279494 | 17.063 | 2.79E-51 | 1.62E-48 | 105.873 |
| SLC43A3 | 0.81759 | 2.486289 | 8.45363 | 3.48E-16 | 5.53E-15 | 25.8838 |
| AC011462.4 | 0.81822 | 2.436018 | 10.6662 | 5.83E-24 | 2.07E-22 | 43.5445 |
| RDH10 | 0.81825 | 3.280766 | 9.13536 | 1.88E-18 | 3.83E-17 | 31.0254 |
| PLOD1 | 0.81901 | 5.656816 | 16.8982 | 1.60E-50 | 8.94E-48 | 104.14 |
| RHPN2 | 0.82197 | 3.97939 | 9.40733 | 2.17E-19 | 4.86E-18 | 33.1548 |
| UBXN11 | 0.82216 | 4.002252 | 13.103 | 1.04E-33 | 1.07E-31 | 65.7621 |
| ZNF560 | 0.82216 | 1.487963 | 6.68747 | 6.38E-11 | 5.62E-10 | 13.9974 |
| IL4I1 | 0.82219 | 1.360847 | 11.4552 | 5.33E-27 | 2.57E-25 | 50.4631 |
| COL22A1 | 0.82327 | 1.802242 | 6.32634 | 5.80E-10 | 4.52E-09 | 11.8452 |
| CRYZ | 0.82393 | 3.917461 | 12.0568 | 2.14E-29 | 1.34E-27 | 55.924 |
| KCNQ1 | 0.82412 | 2.975961 | 12.5058 | 3.16E-31 | 2.41E-29 | 60.0962 |
| FOXM1 | 0.82478 | 3.068525 | 6.32129 | 5.98E-10 | 4.65E-09 | 11.8158 |
| HLA-B | 0.82522 | 8.835342 | 9.21319 | 1.02E-18 | 2.12E-17 | 31.6304 |
| NEXN | 0.82573 | 2.00468 | 12.1702 | 7.43E-30 | 4.85E-28 | 56.9707 |
| LILRA2 | 0.82577 | 1.938511 | 11.4514 | 5.52E-27 | 2.65E-25 | 50.4288 |
| IGFLR1 | 0.82595 | 1.946218 | 16.4096 | 2.78E-48 | 1.20E-45 | 99.0224 |
| B3GNT5 | 0.82615 | 1.799159 | 10.9197 | 6.36E-25 | 2.48E-23 | 45.7352 |
| FXYD5 | 0.82643 | 4.216135 | 10.4791 | 2.93E-23 | 9.61E-22 | 41.9477 |
| SNX7 | 0.82765 | 4.997394 | 16.1296 | 5.26E-47 | 2.00E-44 | 96.1084 |
| APOC2 | 0.82802 | 1.629746 | 11.5128 | 3.17E-27 | 1.57E-25 | 50.9788 |
| VASP | 0.82849 | 4.592397 | 16.7988 | 4.58E-50 | 2.44E-47 | 103.095 |
| TMC8 | 0.8285 | 2.505551 | 12.4293 | 6.52E-31 | 4.82E-29 | 59.3798 |
| GAS2L3 | 0.8286 | 1.151583 | 10.7564 | 2.66E-24 | 9.70E-23 | 44.321 |
| ADPRS | 0.82861 | 5.947584 | 23.9125 | 1.40E-83 | 3.83E-79 | 179.623 |
| SYNM | 0.82928 | 5.271864 | 8.7269 | 4.44E-17 | 7.78E-16 | 27.9103 |
| ARID5A | 0.83009 | 4.427627 | 12.1578 | 8.35E-30 | 5.43E-28 | 56.856 |
| CELSR1 | 0.83018 | 0.966652 | 10.9657 | 4.24E-25 | 1.69E-23 | 46.136 |
| SERTAD1 | 0.8303 | 4.001711 | 12.5668 | 1.77E-31 | 1.39E-29 | 60.6694 |
| CSF1 | 0.83035 | 5.626948 | 10.7828 | 2.11E-24 | 7.78E-23 | 44.5482 |
| HOXB2 | 0.83097 | 0.827656 | 8.78565 | 2.83E-17 | 5.06E-16 | 28.3521 |
| PTCH2 | 0.83252 | 2.749624 | 12.1987 | 5.70E-30 | 3.77E-28 | 57.2339 |
| YBX1 | 0.83283 | 8.970929 | 14.9877 | 7.15E-42 | 1.70E-39 | 84.3869 |
| OAS1 | 0.8338 | 3.075679 | 7.85018 | 2.77E-14 | 3.64E-13 | 21.5799 |
| CROCC | 0.83394 | 3.501488 | 14.661 | 1.99E-40 | 4.22E-38 | 81.0872 |
| THEMIS2 | 0.83404 | 3.635303 | 9.79811 | 9.04E-21 | 2.32E-19 | 36.2886 |
| KANK2 | 0.83421 | 4.299038 | 11.5454 | 2.36E-27 | 1.18E-25 | 51.2717 |
| SNHG12 | 0.83451 | 3.352531 | 14.687 | 1.53E-40 | 3.26E-38 | 81.3494 |
| MFAP2 | 0.83458 | 1.034503 | 9.48795 | 1.13E-19 | 2.60E-18 | 33.7943 |
| PBK | 0.83498 | 2.85034 | 5.86932 | 8.21E-09 | 5.59E-08 | 9.26706 |
| DOCK8 | 0.83503 | 2.577863 | 10.4812 | 2.88E-23 | 9.45E-22 | 41.966 |
| RSPH4A | 0.8351 | 1.514227 | 9.91698 | 3.38E-21 | 9.02E-20 | 37.2587 |
| IER3 | 0.83556 | 3.925066 | 7.95802 | 1.29E-14 | 1.75E-13 | 22.3313 |
| ITPKC | 0.83572 | 4.337201 | 13.9544 | 2.42E-37 | 3.67E-35 | 74.0475 |
| KLHDC8A | 0.83712 | 4.764474 | 7.10051 | 4.55E-12 | 4.65E-11 | 16.5794 |
| AC009005.1 | 0.83713 | 2.197188 | 11.6597 | 8.31E-28 | 4.38E-26 | 52.3022 |
| CBX2 | 0.83724 | 2.441722 | 10.8749 | 9.42E-25 | 3.61E-23 | 45.3459 |
| FHL3 | 0.83739 | 4.793985 | 13.3594 | 8.56E-35 | 9.94E-33 | 68.2332 |
| RCC2 | 0.83791 | 6.260328 | 16.0244 | 1.58E-46 | 5.81E-44 | 95.0175 |
| SQOR | 0.83801 | 2.672414 | 12.5336 | 2.43E-31 | 1.88E-29 | 60.3575 |
| PLBD1 | 0.83819 | 1.901187 | 10.9517 | 4.80E-25 | 1.90E-23 | 46.0139 |
| TAGLN2 | 0.83831 | 6.341035 | 8.45127 | 3.54E-16 | 5.63E-15 | 25.8665 |
| ASPM | 0.83887 | 1.135268 | 9.04145 | 3.92E-18 | 7.72E-17 | 30.3003 |
| CCNB1 | 0.84009 | 3.591426 | 9.32755 | 4.10E-19 | 8.94E-18 | 32.5257 |
| IGF2BP2 | 0.84075 | 1.000981 | 8.10422 | 4.52E-15 | 6.40E-14 | 23.3625 |
| CXorf21 | 0.84184 | 2.027192 | 10.6384 | 7.42E-24 | 2.60E-22 | 43.3063 |
| NUF2 | 0.84184 | 2.072836 | 8.92802 | 9.47E-18 | 1.79E-16 | 29.4315 |
| UBA7 | 0.84192 | 4.481959 | 12.272 | 2.87E-30 | 1.96E-28 | 57.914 |
| ADAM28 | 0.84194 | 2.603315 | 10.569 | 1.35E-23 | 4.59E-22 | 42.7126 |
| SHOX2 | 0.842 | 0.678925 | 7.69484 | 8.24E-14 | 1.02E-12 | 20.5115 |
| NFAM1 | 0.84358 | 2.416641 | 11.7816 | 2.72E-28 | 1.52E-26 | 53.407 |
| SIGLEC8 | 0.84452 | 4.322771 | 7.85597 | 2.66E-14 | 3.51E-13 | 21.6201 |
| ZBTB42 | 0.84473 | 1.973222 | 12.0952 | 1.50E-29 | 9.56E-28 | 56.2775 |
| PLAAT4 | 0.8448 | 6.09393 | 9.29414 | 5.35E-19 | 1.15E-17 | 32.2633 |
| ARHGAP9 | 0.84529 | 2.366081 | 14.1755 | 2.66E-38 | 4.62E-36 | 76.2359 |
| VASN | 0.84539 | 2.468353 | 6.88908 | 1.79E-11 | 1.69E-10 | 15.2419 |
| SPC24 | 0.84543 | 2.235054 | 7.67232 | 9.63E-14 | 1.19E-12 | 20.358 |
| HSPA1B | 0.84556 | 5.624898 | 7.64986 | 1.13E-13 | 1.38E-12 | 20.2052 |
| HJURP | 0.84559 | 1.719816 | 7.82676 | 3.27E-14 | 4.26E-13 | 21.4178 |
| CITED4 | 0.84641 | 2.795744 | 10.4655 | 3.30E-23 | 1.08E-21 | 41.8322 |
| CD4 | 0.8467 | 4.99528 | 10.8763 | 9.31E-25 | 3.57E-23 | 45.3584 |
| SLC37A2 | 0.84806 | 2.508915 | 10.6774 | 5.29E-24 | 1.89E-22 | 43.6406 |
| MAD2L2 | 0.84834 | 5.287246 | 16.0393 | 1.35E-46 | 5.04E-44 | 95.1722 |
| EMILIN1 | 0.84939 | 4.410591 | 10.6342 | 7.70E-24 | 2.69E-22 | 43.27 |
| TNFRSF1A | 0.84958 | 5.471564 | 10.448 | 3.83E-23 | 1.24E-21 | 41.6839 |
| HOXB7 | 0.85024 | 1.113957 | 9.49808 | 1.04E-19 | 2.41E-18 | 33.8748 |
| COL8A1 | 0.85093 | 0.976426 | 8.01248 | 8.74E-15 | 1.20E-13 | 22.7137 |
| CD276 | 0.85152 | 4.812796 | 13.8214 | 9.08E-37 | 1.32E-34 | 72.7381 |
| CD69 | 0.85253 | 1.337561 | 10.5864 | 1.16E-23 | 3.98E-22 | 42.8612 |
| SYDE1 | 0.85291 | 3.905125 | 14.4322 | 2.02E-39 | 3.88E-37 | 78.7927 |
| FCGR1CP | 0.85351 | 2.258591 | 9.81507 | 7.86E-21 | 2.03E-19 | 36.4265 |
| SP100 | 0.85362 | 2.148397 | 12.8657 | 1.02E-32 | 9.09E-31 | 63.4954 |
| MSTN | 0.85366 | 3.616937 | 5.0987 | 4.95E-07 | 2.64E-06 | 5.30295 |
| RARRES2 | 0.8538 | 2.721047 | 5.73056 | 1.78E-08 | 1.16E-07 | 8.51724 |
| CD84 | 0.85435 | 2.69966 | 9.40226 | 2.26E-19 | 5.05E-18 | 33.1146 |
| PIK3R5 | 0.85437 | 2.349888 | 12.0079 | 3.37E-29 | 2.07E-27 | 55.4749 |
| CCDC102A | 0.85458 | 2.814271 | 15.5379 | 2.49E-44 | 7.79E-42 | 90.0001 |
| LRAT | 0.85471 | 1.391519 | 9.49221 | 1.09E-19 | 2.51E-18 | 33.8282 |
| FMOD | 0.85475 | 2.349088 | 5.53553 | 5.14E-08 | 3.14E-07 | 7.48985 |
| LY96 | 0.85493 | 3.893165 | 9.26428 | 6.78E-19 | 1.44E-17 | 32.0294 |
| ITPRIPL1 | 0.855 | 1.718131 | 10.6183 | 8.83E-24 | 3.06E-22 | 43.134 |
| MYCBP | 0.85672 | 2.861202 | 18.0815 | 5.21E-56 | 4.76E-53 | 116.672 |
| FZD7 | 0.85678 | 3.887612 | 7.53808 | 2.43E-13 | 2.86E-12 | 19.4502 |
| BUB1B | 0.85775 | 1.884441 | 7.73366 | 6.29E-14 | 7.94E-13 | 20.7769 |
| LHFPL2 | 0.85787 | 4.119875 | 11.775 | 2.89E-28 | 1.61E-26 | 53.347 |
| SERPING1 | 0.8579 | 5.251935 | 6.864 | 2.10E-11 | 1.97E-10 | 15.0854 |
| AL357992.1 | 0.85891 | 1.247874 | 10.0555 | 1.07E-21 | 2.98E-20 | 38.3989 |
| GPR84 | 0.85892 | 1.207741 | 12.5815 | 1.54E-31 | 1.22E-29 | 60.807 |
| MAP3K7CL | 0.8592 | 1.580716 | 9.40333 | 2.24E-19 | 5.01E-18 | 33.1231 |
| FGL2 | 0.85998 | 3.271167 | 8.55698 | 1.61E-16 | 2.66E-15 | 26.6447 |
| MAGOH | 0.86006 | 5.001179 | 28.9641 | 4.42E-107 | 2.42E-102 | 233.203 |
| SH3TC1 | 0.86161 | 2.696713 | 11.6735 | 7.33E-28 | 3.91E-26 | 52.4267 |
| PCOLCE | 0.86163 | 2.937605 | 9.80858 | 8.30E-21 | 2.14E-19 | 36.3737 |
| DOK3 | 0.86249 | 2.452607 | 13.6732 | 3.93E-36 | 5.25E-34 | 71.2857 |
| C19orf18 | 0.86439 | 2.371439 | 11.9465 | 5.95E-29 | 3.55E-27 | 54.911 |
| NCAPG | 0.86529 | 1.662871 | 8.24418 | 1.63E-15 | 2.41E-14 | 24.3629 |
| DOCK2 | 0.86542 | 2.451971 | 11.2891 | 2.38E-26 | 1.07E-24 | 48.9828 |
| ZFP36 | 0.86559 | 6.537177 | 7.08583 | 5.01E-12 | 5.09E-11 | 16.4854 |
| FCGRT | 0.86586 | 6.200137 | 15.4314 | 7.48E-44 | 2.22E-41 | 88.9087 |
| CDKN2C | 0.86625 | 4.363295 | 7.76493 | 5.05E-14 | 6.45E-13 | 20.9915 |
| TEAD4 | 0.86656 | 2.203098 | 8.86321 | 1.56E-17 | 2.88E-16 | 28.9386 |
| G0S2 | 0.86747 | 1.738457 | 6.52758 | 1.72E-10 | 1.43E-09 | 13.0322 |
| SLA | 0.86757 | 3.312112 | 9.91724 | 3.38E-21 | 9.01E-20 | 37.2608 |
| LYL1 | 0.86813 | 2.900945 | 12.9742 | 3.60E-33 | 3.46E-31 | 64.5295 |
| FUCA2 | 0.8682 | 4.584257 | 10.6193 | 8.76E-24 | 3.03E-22 | 43.1425 |
| FABP5 | 0.87091 | 2.748321 | 6.77097 | 3.78E-11 | 3.43E-10 | 14.5091 |
| RBM47 | 0.87127 | 1.699445 | 12.4235 | 6.89E-31 | 5.05E-29 | 59.3254 |
| IL13RA1 | 0.87144 | 5.235681 | 11.0097 | 2.87E-25 | 1.17E-23 | 46.5207 |
| TK1 | 0.87232 | 2.949029 | 8.02657 | 7.90E-15 | 1.09E-13 | 22.813 |
| LRRC42 | 0.87305 | 4.550684 | 20.4302 | 4.52E-67 | 1.08E-63 | 141.935 |
| NEAT1 | 0.8733 | 3.80694 | 9.29251 | 5.42E-19 | 1.16E-17 | 32.2505 |
| TLR1 | 0.87367 | 2.166833 | 10.9698 | 4.09E-25 | 1.63E-23 | 46.1718 |
| KIFC1 | 0.87374 | 2.842828 | 6.95052 | 1.20E-11 | 1.16E-10 | 15.6272 |
| LINC01150 | 0.87378 | 1.968412 | 11.8854 | 1.05E-28 | 6.09E-27 | 54.3529 |
| TRIP6 | 0.87481 | 5.49545 | 10.4173 | 4.99E-23 | 1.60E-21 | 41.4241 |
| AC002398.1 | 0.87491 | 2.020021 | 17.6858 | 3.63E-54 | 2.62E-51 | 112.461 |
| NMI | 0.87522 | 2.833636 | 12.8921 | 7.93E-33 | 7.27E-31 | 63.7464 |
| FAM114A1 | 0.87543 | 2.930968 | 11.7292 | 4.40E-28 | 2.41E-26 | 52.9311 |
| STXBP2 | 0.87571 | 2.320218 | 15.1891 | 9.07E-43 | 2.43E-40 | 86.4339 |
| WARS2-IT1 | 0.87606 | 1.400741 | 13.1502 | 6.56E-34 | 6.94E-32 | 66.2153 |
| JUN | 0.87622 | 7.275427 | 10.4324 | 4.38E-23 | 1.41E-21 | 41.5524 |
| TMSB4XP8 | 0.87697 | 4.978639 | 9.24949 | 7.63E-19 | 1.61E-17 | 31.9137 |
| NECAP2 | 0.87699 | 5.185998 | 17.9644 | 1.83E-55 | 1.52E-52 | 115.424 |
| MMEL1 | 0.87731 | 0.893848 | 12.6109 | 1.17E-31 | 9.26E-30 | 61.0836 |
| ELF4 | 0.87762 | 2.001545 | 12.9735 | 3.62E-33 | 3.48E-31 | 64.5225 |
| CCER2 | 0.87798 | 2.183336 | 8.81679 | 2.23E-17 | 4.03E-16 | 28.5871 |
| AK2 | 0.87815 | 5.012318 | 22.8611 | 1.33E-78 | 1.82E-74 | 168.265 |
| C1orf226 | 0.87829 | 4.174158 | 11.2438 | 3.58E-26 | 1.58E-24 | 48.5808 |
| CLEC5A | 0.87838 | 0.9295 | 9.33717 | 3.80E-19 | 8.30E-18 | 32.6013 |
| EFEMP1 | 0.87858 | 6.37199 | 7.26617 | 1.52E-12 | 1.64E-11 | 17.6501 |
| MNDA | 0.8788 | 3.751968 | 9.65908 | 2.83E-20 | 6.86E-19 | 35.1638 |
| RNF135 | 0.88041 | 3.012784 | 12.9256 | 5.74E-33 | 5.39E-31 | 64.0662 |
| GYPC | 0.88055 | 4.019237 | 10.6295 | 8.02E-24 | 2.79E-22 | 43.2298 |
| GAPT | 0.88073 | 1.209824 | 12.0532 | 2.21E-29 | 1.39E-27 | 55.8909 |
| SLC2A10 | 0.88112 | 2.621868 | 8.94122 | 8.55E-18 | 1.63E-16 | 29.5322 |
| TCTEX1D1 | 0.88132 | 1.126792 | 7.11635 | 4.10E-12 | 4.21E-11 | 16.6809 |
| GMFG | 0.88216 | 4.392773 | 14.294 | 8.11E-39 | 1.50E-36 | 77.4139 |
| RUBCNL | 0.8822 | 2.034894 | 11.1012 | 1.28E-25 | 5.36E-24 | 47.323 |
| SIPA1 | 0.88262 | 4.634404 | 15.1447 | 1.43E-42 | 3.70E-40 | 85.9814 |
| NDNF | 0.88286 | 2.009713 | 7.42658 | 5.19E-13 | 5.91E-12 | 18.7057 |
| TEAD2 | 0.88565 | 3.015714 | 11.5792 | 1.73E-27 | 8.87E-26 | 51.5756 |
| REST | 0.88574 | 3.007477 | 13.6087 | 7.42E-36 | 9.59E-34 | 70.6558 |
| SEC61G | 0.88576 | 5.845392 | 8.83933 | 1.88E-17 | 3.42E-16 | 28.7576 |
| AC103923.1 | 0.88578 | 1.592327 | 13.4365 | 4.03E-35 | 4.84E-33 | 68.9803 |
| KIAA0040 | 0.88635 | 2.917751 | 8.41783 | 4.54E-16 | 7.15E-15 | 25.6218 |
| CTSZ | 0.88972 | 6.472376 | 12.1455 | 9.36E-30 | 6.07E-28 | 56.742 |
| RGS19 | 0.89024 | 4.559355 | 16.6515 | 2.17E-49 | 1.04E-46 | 101.551 |
| CCR5AS | 0.89071 | 1.678321 | 11.6629 | 8.08E-28 | 4.28E-26 | 52.3305 |
| CRB2 | 0.89075 | 3.50087 | 7.33187 | 9.83E-13 | 1.08E-11 | 18.0802 |
| PLAT | 0.89077 | 3.124572 | 7.56398 | 2.03E-13 | 2.42E-12 | 19.6243 |
| RAD54L | 0.89123 | 1.606793 | 11.4934 | 3.78E-27 | 1.85E-25 | 50.8045 |
| CD300C | 0.89196 | 2.095778 | 12.9041 | 7.06E-33 | 6.54E-31 | 63.8611 |
| TREML1 | 0.89273 | 1.982493 | 12.5458 | 2.16E-31 | 1.68E-29 | 60.4721 |
| APOBR | 0.89282 | 2.566141 | 12.2184 | 4.74E-30 | 3.17E-28 | 57.417 |
| WAS | 0.89291 | 4.218359 | 12.764 | 2.71E-32 | 2.28E-30 | 62.5306 |
| IRF7 | 0.89348 | 3.731115 | 10.8504 | 1.17E-24 | 4.43E-23 | 45.1331 |
| ADAP2 | 0.89365 | 3.637933 | 13.1675 | 5.55E-34 | 5.92E-32 | 66.3818 |
| BIN2 | 0.89472 | 2.939446 | 12.3704 | 1.14E-30 | 8.12E-29 | 58.8296 |
| SALL3 | 0.89625 | 4.082434 | 8.59266 | 1.23E-16 | 2.06E-15 | 26.9089 |
| FUOM | 0.89672 | 2.955008 | 13.0772 | 1.33E-33 | 1.36E-31 | 65.5153 |
| IGSF6 | 0.89709 | 3.344141 | 11.0391 | 2.21E-25 | 9.13E-24 | 46.7784 |
| IRF8 | 0.89712 | 3.375869 | 10.4636 | 3.35E-23 | 1.09E-21 | 41.8161 |
| TNFRSF1B | 0.8985 | 4.794772 | 11.7356 | 4.15E-28 | 2.28E-26 | 52.989 |
| CENPA | 0.89913 | 1.378783 | 9.71052 | 1.86E-20 | 4.62E-19 | 35.5787 |
| LINC01023 | 0.89932 | 3.490303 | 12.3113 | 1.98E-30 | 1.37E-28 | 58.2793 |
| IL10RA | 0.90035 | 3.035933 | 10.5772 | 1.26E-23 | 4.29E-22 | 42.7827 |
| STAT5A | 0.90046 | 3.276429 | 13.0076 | 2.61E-33 | 2.56E-31 | 64.8487 |
| C7orf57 | 0.90118 | 0.917365 | 7.87127 | 2.39E-14 | 3.16E-13 | 21.7263 |
| IRF5 | 0.9023 | 3.210434 | 12.8806 | 8.86E-33 | 7.98E-31 | 63.6371 |
| AL355974.2 | 0.90257 | 2.590888 | 7.00075 | 8.71E-12 | 8.55E-11 | 15.9442 |
| WNT5A | 0.9027 | 3.260585 | 10.3202 | 1.14E-22 | 3.52E-21 | 40.6054 |
| TACC3 | 0.90348 | 2.96001 | 9.58389 | 5.21E-20 | 1.24E-18 | 34.56 |
| EBI3 | 0.90446 | 3.782449 | 11.1961 | 5.48E-26 | 2.38E-24 | 48.1593 |
| MFNG | 0.90495 | 3.995888 | 12.8845 | 8.53E-33 | 7.72E-31 | 63.6748 |
| CCNA2 | 0.90564 | 2.538669 | 9.2397 | 8.24E-19 | 1.73E-17 | 31.8372 |
| EFNA4 | 0.90583 | 2.509425 | 15.8387 | 1.09E-45 | 3.75E-43 | 93.0972 |
| GAL3ST4 | 0.90616 | 4.816872 | 13.5932 | 8.65E-36 | 1.11E-33 | 70.5046 |
| TXLNA | 0.90649 | 5.307038 | 20.7672 | 1.14E-68 | 3.68E-65 | 145.582 |
| IL13RA2 | 0.90725 | 1.815502 | 5.65164 | 2.74E-08 | 1.74E-07 | 8.09778 |
| CLEC18B | 0.90776 | 1.206936 | 13.1806 | 4.88E-34 | 5.26E-32 | 66.5082 |
| FAM111A | 0.90807 | 3.287099 | 14.2207 | 1.69E-38 | 2.98E-36 | 76.6849 |
| SNORD99 | 0.90847 | 2.943233 | 9.88281 | 4.49E-21 | 1.18E-19 | 36.979 |
| SHKBP1 | 0.90896 | 4.353262 | 17.8134 | 9.26E-55 | 7.05E-52 | 113.817 |
| SAMD9L | 0.91017 | 3.106961 | 9.46707 | 1.34E-19 | 3.05E-18 | 33.6282 |
| ORC1 | 0.91131 | 1.391068 | 12.3333 | 1.61E-30 | 1.13E-28 | 58.4845 |
| MIIP | 0.91153 | 5.565134 | 15.0931 | 2.43E-42 | 6.03E-40 | 85.4569 |
| AL161785.1 | 0.91166 | 2.561024 | 11.7374 | 4.08E-28 | 2.24E-26 | 53.0053 |
| SIGLEC10 | 0.91188 | 4.070262 | 10.0179 | 1.46E-21 | 4.03E-20 | 38.0884 |
| WEE1 | 0.91234 | 2.484842 | 10.377 | 7.03E-23 | 2.22E-21 | 41.0841 |
| RCN3 | 0.91242 | 3.57816 | 14.0505 | 9.30E-38 | 1.50E-35 | 74.9965 |
| KIF4A | 0.91274 | 2.167521 | 8.55429 | 1.64E-16 | 2.71E-15 | 26.6248 |
| ZDHHC12 | 0.91292 | 3.217017 | 14.3668 | 3.90E-39 | 7.39E-37 | 78.1402 |
| HLA-DQA2 | 0.91398 | 2.445144 | 5.28658 | 1.90E-07 | 1.08E-06 | 6.22401 |
| CMTM7 | 0.91436 | 2.414416 | 13.926 | 3.21E-37 | 4.82E-35 | 73.7681 |
| MEOX2 | 0.9144 | 1.091397 | 5.9997 | 3.92E-09 | 2.78E-08 | 9.98566 |
| SLC15A3 | 0.91576 | 3.35851 | 12.1802 | 6.77E-30 | 4.43E-28 | 57.0626 |
| TMSB4X | 0.91582 | 10.30134 | 15.6967 | 4.80E-45 | 1.56E-42 | 91.6323 |
| PCED1B | 0.91602 | 2.226435 | 14.2703 | 1.03E-38 | 1.86E-36 | 77.1783 |
| SELPLG | 0.9163 | 5.624435 | 9.62679 | 3.68E-20 | 8.86E-19 | 34.9041 |
| CCR5 | 0.91683 | 1.403822 | 11.466 | 4.84E-27 | 2.34E-25 | 50.5596 |
| IQGAP1 | 0.91719 | 4.211903 | 11.7215 | 4.73E-28 | 2.58E-26 | 52.861 |
| COL6A2 | 0.9174 | 3.579202 | 7.31162 | 1.13E-12 | 1.23E-11 | 17.9474 |
| SDC4 | 0.91814 | 5.543027 | 6.0671 | 2.66E-09 | 1.92E-08 | 10.3625 |
| H4C9 | 0.91834 | 2.547291 | 15.0242 | 4.92E-42 | 1.18E-39 | 84.7568 |
| C4A | 0.92031 | 3.150642 | 8.35587 | 7.18E-16 | 1.11E-14 | 25.1704 |
| BTK | 0.92054 | 2.644606 | 12.8834 | 8.62E-33 | 7.80E-31 | 63.6638 |
| FAM183A | 0.92231 | 0.730819 | 7.82744 | 3.26E-14 | 4.24E-13 | 21.4225 |
| AC138207.5 | 0.92284 | 3.063543 | 13.1156 | 9.17E-34 | 9.61E-32 | 65.8832 |
| LPCAT2 | 0.92368 | 3.115667 | 10.6798 | 5.18E-24 | 1.85E-22 | 43.6617 |
| PLAUR | 0.92388 | 2.171583 | 11.4415 | 6.04E-27 | 2.89E-25 | 50.3403 |
| ANG | 0.92442 | 1.805968 | 12.7842 | 2.23E-32 | 1.90E-30 | 62.7216 |
| DMRTA2 | 0.92609 | 0.99097 | 7.61788 | 1.40E-13 | 1.69E-12 | 19.9883 |
| LRRN4CL | 0.92626 | 1.900527 | 7.98068 | 1.10E-14 | 1.50E-13 | 22.4902 |
| ITGAX | 0.9264 | 3.523546 | 10.2191 | 2.69E-22 | 7.97E-21 | 39.7581 |
| FBXO32 | 0.92692 | 4.974626 | 9.3241 | 4.22E-19 | 9.18E-18 | 32.4986 |
| C2 | 0.9276 | 2.14639 | 11.2452 | 3.53E-26 | 1.56E-24 | 48.5933 |
| GADD45A | 0.92895 | 5.414513 | 9.46632 | 1.35E-19 | 3.07E-18 | 33.6223 |
| KIF20A | 0.92938 | 1.676325 | 8.58314 | 1.32E-16 | 2.21E-15 | 26.8383 |
| CFI | 0.92952 | 3.349632 | 7.6115 | 1.47E-13 | 1.76E-12 | 19.9451 |
| WLS | 0.92974 | 7.390286 | 13.759 | 1.68E-36 | 2.35E-34 | 72.1261 |
| PDE8A | 0.93067 | 3.882649 | 11.3003 | 2.16E-26 | 9.71E-25 | 49.0816 |
| LSP1 | 0.93095 | 2.157995 | 9.36964 | 2.93E-19 | 6.49E-18 | 32.8571 |
| PLSCR1 | 0.93101 | 3.731421 | 10.6152 | 9.07E-24 | 3.14E-22 | 43.1073 |
| BATF3 | 0.93171 | 2.095409 | 12.4816 | 3.98E-31 | 3.00E-29 | 59.8698 |
| EN1 | 0.9319 | 0.787804 | 8.5467 | 1.73E-16 | 2.86E-15 | 26.5687 |
| LST1 | 0.93249 | 4.075152 | 12.2574 | 3.29E-30 | 2.23E-28 | 57.7779 |
| RRM2 | 0.93367 | 2.367335 | 7.02184 | 7.60E-12 | 7.52E-11 | 16.0779 |
| ADAMTS15 | 0.93431 | 2.438625 | 7.98882 | 1.04E-14 | 1.41E-13 | 22.5474 |
| WDR38 | 0.93459 | 1.079156 | 8.26718 | 1.38E-15 | 2.06E-14 | 24.5285 |
| E2F2 | 0.936 | 1.372179 | 10.6654 | 5.87E-24 | 2.08E-22 | 43.5381 |
| CSF2RA | 0.93648 | 3.746366 | 10.5824 | 1.20E-23 | 4.11E-22 | 42.8274 |
| BCL2A1 | 0.9369 | 2.081397 | 8.57708 | 1.38E-16 | 2.30E-15 | 26.7935 |
| ZNF518B | 0.93879 | 2.304818 | 11.9385 | 6.41E-29 | 3.81E-27 | 54.8385 |
| PARP4P3 | 0.94011 | 1.407158 | 6.57355 | 1.29E-10 | 1.09E-09 | 13.3077 |
| CSRP2 | 0.94056 | 5.108624 | 8.60314 | 1.13E-16 | 1.90E-15 | 26.9867 |
| IFI6 | 0.94063 | 8.044144 | 8.04281 | 7.03E-15 | 9.79E-14 | 22.9276 |
| RAB34 | 0.94076 | 3.441762 | 9.04019 | 3.96E-18 | 7.78E-17 | 30.2906 |
| MS4A4A | 0.94107 | 3.443363 | 9.09717 | 2.54E-18 | 5.10E-17 | 30.7299 |
| MELK | 0.94126 | 1.839841 | 8.48768 | 2.70E-16 | 4.35E-15 | 26.1338 |
| DOCK7 | 0.94149 | 4.294684 | 12.8375 | 1.34E-32 | 1.18E-30 | 63.2278 |
| LOXL2 | 0.94194 | 2.852381 | 9.71676 | 1.76E-20 | 4.40E-19 | 35.6291 |
| MCUB | 0.94211 | 2.484559 | 10.07 | 9.44E-22 | 2.64E-20 | 38.5191 |
| GPR34 | 0.94242 | 5.500206 | 8.94046 | 8.60E-18 | 1.64E-16 | 29.5264 |
| AC018755.4 | 0.94262 | 2.252111 | 10.4126 | 5.19E-23 | 1.66E-21 | 41.3847 |
| MOXD1 | 0.94302 | 1.856609 | 5.68125 | 2.33E-08 | 1.49E-07 | 8.25457 |
| AJ011932.1 | 0.94324 | 0.921569 | 10.8755 | 9.38E-25 | 3.59E-23 | 45.3511 |
| MILR1 | 0.94347 | 2.838492 | 11.6778 | 7.05E-28 | 3.77E-26 | 52.4658 |
| TMEM176B | 0.9449 | 5.500535 | 8.68922 | 5.91E-17 | 1.02E-15 | 27.6281 |
| PDCD1LG2 | 0.9461 | 1.551543 | 12.0573 | 2.13E-29 | 1.34E-27 | 55.9287 |
| MGP | 0.94631 | 4.239912 | 5.87132 | 8.12E-09 | 5.53E-08 | 9.27795 |
| DRAXIN | 0.94858 | 2.467875 | 9.83866 | 6.47E-21 | 1.68E-19 | 36.6186 |
| DCTD | 0.94991 | 4.836404 | 11.9477 | 5.88E-29 | 3.52E-27 | 54.9227 |
| PLCB2 | 0.9504 | 3.552286 | 12.4544 | 5.14E-31 | 3.84E-29 | 59.6149 |
| WDR78 | 0.95055 | 1.564262 | 13.9331 | 2.99E-37 | 4.51E-35 | 73.8379 |
| CENPU | 0.95067 | 2.408627 | 8.72943 | 4.35E-17 | 7.63E-16 | 27.9293 |
| REEP4 | 0.9532 | 3.975426 | 18.0173 | 1.04E-55 | 8.89E-53 | 115.988 |
| TROAP | 0.95344 | 1.622025 | 9.21812 | 9.78E-19 | 2.04E-17 | 31.6687 |
| AC090692.1 | 0.95573 | 2.427308 | 9.27016 | 6.48E-19 | 1.38E-17 | 32.0754 |
| FOXD3 | 0.95581 | 0.896292 | 11.1892 | 5.82E-26 | 2.52E-24 | 48.0985 |
| RCC1 | 0.9563 | 3.96526 | 14.1305 | 4.18E-38 | 7.04E-36 | 75.7894 |
| TPX2 | 0.95652 | 3.632731 | 7.56462 | 2.03E-13 | 2.41E-12 | 19.6287 |
| FSTL1 | 0.95665 | 4.585006 | 10.3627 | 7.94E-23 | 2.49E-21 | 40.9637 |
| INPP5D | 0.95757 | 3.888131 | 13.2706 | 2.04E-34 | 2.32E-32 | 67.3744 |
| PSRC1 | 0.95771 | 5.201746 | 10.2855 | 1.53E-22 | 4.66E-21 | 40.314 |
| PDIA5 | 0.95772 | 2.148924 | 15.9444 | 3.64E-46 | 1.29E-43 | 94.1896 |
| LRRC25 | 0.96006 | 2.681143 | 11.4375 | 6.26E-27 | 2.99E-25 | 50.3045 |
| CLCF1 | 0.9604 | 1.56726 | 10.546 | 1.65E-23 | 5.56E-22 | 42.5165 |
| STK19B | 0.96044 | 2.171938 | 7.76705 | 4.98E-14 | 6.36E-13 | 21.0061 |
| EN2 | 0.9605 | 1.25664 | 12.1996 | 5.65E-30 | 3.74E-28 | 57.2426 |
| POSTN | 0.96068 | 0.977807 | 6.07047 | 2.61E-09 | 1.89E-08 | 10.3814 |
| CCDC146 | 0.9609 | 2.860457 | 12.4321 | 6.35E-31 | 4.70E-29 | 59.4062 |
| SLC7A7 | 0.96153 | 3.046834 | 12.9961 | 2.91E-33 | 2.83E-31 | 64.7392 |
| LCP1 | 0.96188 | 4.269988 | 11.1284 | 1.00E-25 | 4.25E-24 | 47.5625 |
| NFATC1 | 0.96295 | 2.194225 | 15.1085 | 2.07E-42 | 5.22E-40 | 85.6136 |
| STK40 | 0.96307 | 4.700651 | 18.4603 | 8.81E-58 | 9.11E-55 | 120.719 |
| NFIA | 0.96326 | 5.350923 | 14.9152 | 1.50E-41 | 3.42E-39 | 83.6526 |
| RNASET2 | 0.9633 | 4.43629 | 13.7285 | 2.28E-36 | 3.12E-34 | 71.8275 |
| HPGDS | 0.96345 | 2.98466 | 11.5496 | 2.27E-27 | 1.14E-25 | 51.3091 |
| MOV10 | 0.96423 | 3.891075 | 18.159 | 2.26E-56 | 2.18E-53 | 117.498 |
| ITGAL | 0.96487 | 2.14138 | 11.7277 | 4.46E-28 | 2.44E-26 | 52.9175 |
| HOXA10 | 0.96593 | 0.70459 | 9.00408 | 5.25E-18 | 1.02E-16 | 30.0131 |
| SIGLEC14 | 0.96664 | 2.59452 | 9.53357 | 7.84E-20 | 1.84E-18 | 34.1577 |
| VAV1 | 0.96664 | 2.883669 | 12.9194 | 6.10E-33 | 5.70E-31 | 64.0073 |
| ARHGDIB | 0.96784 | 6.451739 | 13.8198 | 9.22E-37 | 1.33E-34 | 72.7228 |
| HERC5 | 0.96864 | 2.871033 | 10.8844 | 8.67E-25 | 3.35E-23 | 45.4285 |
| DLGAP5 | 0.97033 | 1.366562 | 9.62609 | 3.70E-20 | 8.91E-19 | 34.8985 |
| ITGAM | 0.97239 | 3.19224 | 11.8217 | 1.88E-28 | 1.07E-26 | 53.7722 |
| BLNK | 0.97466 | 2.544005 | 11.8837 | 1.06E-28 | 6.18E-27 | 54.3374 |
| FBP1 | 0.97493 | 2.668886 | 12.4244 | 6.83E-31 | 5.02E-29 | 59.3344 |
| COL3A1 | 0.97522 | 2.895843 | 6.2342 | 1.00E-09 | 7.60E-09 | 11.3121 |
| TNFRSF11B | 0.97542 | 1.25776 | 9.98074 | 1.99E-21 | 5.41E-20 | 37.7822 |
| CTSC | 0.97748 | 3.079417 | 12.3857 | 9.84E-31 | 7.12E-29 | 58.9723 |
| TCIRG1 | 0.97778 | 3.487241 | 14.2525 | 1.23E-38 | 2.19E-36 | 77.0013 |
| PTTG1 | 0.97815 | 3.2565 | 8.98728 | 5.98E-18 | 1.16E-16 | 29.8844 |
| CLEC7A | 0.97819 | 2.283224 | 11.6492 | 9.15E-28 | 4.80E-26 | 52.2072 |
| RAC2 | 0.97829 | 2.768979 | 11.9382 | 6.42E-29 | 3.82E-27 | 54.8358 |
| LPAR6 | 0.97917 | 3.616398 | 13.7872 | 1.27E-36 | 1.81E-34 | 72.4033 |
| TBXAS1 | 0.97942 | 3.49184 | 12.7227 | 4.02E-32 | 3.31E-30 | 62.1395 |
| CALHM6 | 0.98105 | 2.250708 | 10.7291 | 3.37E-24 | 1.23E-22 | 44.0853 |
| CSF3R | 0.98132 | 2.906061 | 12.6907 | 5.45E-32 | 4.40E-30 | 61.837 |
| APOBEC3C | 0.9834 | 3.632842 | 11.3161 | 1.87E-26 | 8.46E-25 | 49.2222 |
| TLR7 | 0.98451 | 2.604524 | 10.6337 | 7.73E-24 | 2.70E-22 | 43.2658 |
| CYTH4 | 0.98458 | 3.025445 | 12.8908 | 8.03E-33 | 7.35E-31 | 63.7339 |
| DDIT4L | 0.98547 | 1.495223 | 7.5201 | 2.75E-13 | 3.22E-12 | 19.3295 |
| STAB1 | 0.98553 | 4.292045 | 10.7524 | 2.75E-24 | 1.01E-22 | 44.2857 |
| CDK2 | 0.98562 | 3.447407 | 13.1866 | 4.61E-34 | 4.98E-32 | 66.5656 |
| AC002456.1 | 0.98586 | 2.512223 | 13.7904 | 1.23E-36 | 1.76E-34 | 72.4346 |
| RASAL3 | 0.98635 | 2.443216 | 14.9261 | 1.34E-41 | 3.10E-39 | 83.763 |
| MS4A7 | 0.9864 | 4.043645 | 10.0512 | 1.11E-21 | 3.08E-20 | 38.3635 |
| HCK | 0.98658 | 3.978742 | 12.081 | 1.71E-29 | 1.08E-27 | 56.1471 |
| TEKT2 | 0.98746 | 2.389222 | 12.3455 | 1.44E-30 | 1.02E-28 | 58.5978 |
| AL035446.1 | 0.98986 | 1.663006 | 8.4225 | 4.38E-16 | 6.91E-15 | 25.656 |
| SIGLEC9 | 0.9914 | 2.324193 | 12.2804 | 2.65E-30 | 1.82E-28 | 57.992 |
| SLC16A3 | 0.99167 | 2.557707 | 13.8468 | 7.05E-37 | 1.03E-34 | 72.9885 |
| CENPF | 0.9924 | 2.407997 | 8.18838 | 2.45E-15 | 3.57E-14 | 23.9625 |
| PTPRC | 0.99276 | 2.817029 | 10.6227 | 8.50E-24 | 2.95E-22 | 43.1716 |
| GPSM3 | 0.99432 | 4.812094 | 13.9104 | 3.75E-37 | 5.59E-35 | 73.614 |
| LILRB1 | 0.9945 | 2.31332 | 12.7854 | 2.21E-32 | 1.88E-30 | 62.7326 |
| ARHGAP4 | 0.99714 | 3.769759 | 15.2742 | 3.78E-43 | 1.05E-40 | 87.3014 |
| ISG15 | 0.99726 | 6.22974 | 8.01944 | 8.32E-15 | 1.15E-13 | 22.7628 |
| DHRS3 | 0.99869 | 5.60003 | 12.1365 | 1.02E-29 | 6.59E-28 | 56.6585 |
| RPS6KA1 | 0.99909 | 3.616935 | 13.488 | 2.43E-35 | 2.96E-33 | 69.4808 |
| PLP2 | 1.00038 | 4.643193 | 9.26377 | 6.81E-19 | 1.44E-17 | 32.0254 |
| TGFB1 | 1.00106 | 5.350195 | 14.8561 | 2.74E-41 | 6.13E-39 | 83.0544 |
| NCKAP1L | 1.00274 | 3.199473 | 12.2367 | 3.99E-30 | 2.69E-28 | 57.5866 |
| SERTAD3 | 1.00325 | 3.755448 | 17.4953 | 2.79E-53 | 1.84E-50 | 110.44 |
| CIITA | 1.00342 | 1.73813 | 11.6119 | 1.29E-27 | 6.65E-26 | 51.87 |
| NDC80 | 1.00527 | 1.848322 | 9.63122 | 3.55E-20 | 8.56E-19 | 34.9397 |
| FYB1 | 1.00542 | 3.448408 | 10.7819 | 2.13E-24 | 7.83E-23 | 44.541 |
| AC008760.2 | 1.00873 | 1.174504 | 11.9036 | 8.85E-29 | 5.19E-27 | 54.5188 |
| IQGAP2 | 1.0093 | 2.226323 | 9.21525 | 1.00E-18 | 2.09E-17 | 31.6464 |
| ST14 | 1.0104 | 2.179745 | 10.294 | 1.43E-22 | 4.36E-21 | 40.3854 |
| CEBPA | 1.0113 | 3.960432 | 11.6243 | 1.15E-27 | 5.98E-26 | 51.9819 |
| SUSD3 | 1.01169 | 3.433136 | 10.7226 | 3.57E-24 | 1.29E-22 | 44.0293 |
| IFI44 | 1.01175 | 4.648992 | 11.8196 | 1.92E-28 | 1.09E-26 | 53.7527 |
| IGFBP5 | 1.01282 | 7.017678 | 9.32543 | 4.17E-19 | 9.08E-18 | 32.509 |
| NCF4 | 1.01384 | 3.516126 | 13.2117 | 3.61E-34 | 3.96E-32 | 66.8074 |
| PDGFD | 1.01511 | 1.652294 | 9.7362 | 1.50E-20 | 3.78E-19 | 35.7864 |
| MAN1C1 | 1.01516 | 3.700411 | 10.5019 | 2.41E-23 | 8.01E-22 | 42.1415 |
| RGS10 | 1.01896 | 5.808714 | 11.7802 | 2.76E-28 | 1.54E-26 | 53.3946 |
| COL28A1 | 1.02072 | 2.443182 | 7.69976 | 7.96E-14 | 9.92E-13 | 20.5451 |
| HDAC1 | 1.0225 | 5.117638 | 20.3329 | 1.31E-66 | 2.87E-63 | 140.881 |
| PIMREG | 1.02254 | 2.583662 | 7.66881 | 9.87E-14 | 1.22E-12 | 20.334 |
| PARVG | 1.02401 | 2.451013 | 14.5749 | 4.77E-40 | 9.75E-38 | 80.2228 |
| CCNL2 | 1.02473 | 5.500461 | 14.6057 | 3.49E-40 | 7.28E-38 | 80.5314 |
| SRPX2 | 1.02499 | 2.214176 | 8.81945 | 2.19E-17 | 3.96E-16 | 28.6072 |
| NUSAP1 | 1.02529 | 3.640789 | 7.65647 | 1.08E-13 | 1.32E-12 | 20.2501 |
| CTSS | 1.02679 | 4.451737 | 10.9619 | 4.38E-25 | 1.74E-23 | 46.1036 |
| CARD16 | 1.02815 | 1.865567 | 12.6857 | 5.72E-32 | 4.61E-30 | 61.7893 |
| AC092131.1 | 1.02904 | 1.583833 | 9.79049 | 9.63E-21 | 2.47E-19 | 36.2267 |
| GPR183 | 1.02948 | 3.14655 | 10.0719 | 9.30E-22 | 2.61E-20 | 38.534 |
| BMP8B | 1.03041 | 2.56628 | 10.5291 | 1.91E-23 | 6.40E-22 | 42.3726 |
| CCNB2 | 1.03056 | 2.277399 | 8.95179 | 7.88E-18 | 1.50E-16 | 29.6129 |
| PTPN6 | 1.0311 | 3.530484 | 14.0223 | 1.23E-37 | 1.94E-35 | 74.7185 |
| AGTRAP | 1.0311 | 4.900523 | 16.8613 | 2.37E-50 | 1.28E-47 | 103.751 |
| PLEK | 1.03121 | 3.662585 | 11.4247 | 7.03E-27 | 3.33E-25 | 50.1902 |
| BST2 | 1.03218 | 6.491625 | 10.9795 | 3.75E-25 | 1.51E-23 | 46.2571 |
| UNC93B1 | 1.03344 | 3.817675 | 13.3558 | 8.86E-35 | 1.03E-32 | 68.1985 |
| CNN3 | 1.03482 | 8.597016 | 16.0327 | 1.45E-46 | 5.36E-44 | 95.1034 |
| SYK | 1.03508 | 3.69157 | 12.1117 | 1.28E-29 | 8.26E-28 | 56.4298 |
| GPX7 | 1.03638 | 3.866759 | 17.2585 | 3.48E-52 | 2.19E-49 | 107.935 |
| HMOX1 | 1.03861 | 5.428248 | 11.9232 | 7.38E-29 | 4.37E-27 | 54.6981 |
| SOCS3 | 1.03982 | 3.544597 | 6.5872 | 1.19E-10 | 1.01E-09 | 13.3898 |
| FERMT3 | 1.04029 | 4.043129 | 13.6246 | 6.34E-36 | 8.28E-34 | 70.8115 |
| ASF1B | 1.04037 | 2.498543 | 9.28564 | 5.73E-19 | 1.23E-17 | 32.1967 |
| C5AR1 | 1.04106 | 3.075976 | 10.0703 | 9.42E-22 | 2.64E-20 | 38.5216 |
| TOP2A | 1.04139 | 3.433001 | 6.47691 | 2.34E-10 | 1.92E-09 | 12.7303 |
| COL4A2 | 1.04168 | 4.475923 | 7.79039 | 4.23E-14 | 5.44E-13 | 21.1667 |
| HLA-DRB6 | 1.04216 | 3.655266 | 7.05942 | 5.95E-12 | 5.98E-11 | 16.3169 |
| GPR65 | 1.04326 | 1.439722 | 12.7984 | 1.95E-32 | 1.67E-30 | 62.8567 |
| MYO1F | 1.04512 | 3.152198 | 14.2845 | 8.93E-39 | 1.63E-36 | 77.319 |
| P3H2 | 1.04543 | 2.181249 | 12.6954 | 5.22E-32 | 4.24E-30 | 61.881 |
| CD99 | 1.04633 | 7.928933 | 9.17328 | 1.39E-18 | 2.87E-17 | 31.3197 |
| RHBDF2 | 1.04745 | 3.76791 | 12.8074 | 1.79E-32 | 1.54E-30 | 62.942 |
| VAMP8 | 1.04909 | 5.321035 | 13.7064 | 2.83E-36 | 3.85E-34 | 71.6113 |
| ZNF662 | 1.05101 | 2.294109 | 12.8659 | 1.02E-32 | 9.09E-31 | 63.4971 |
| LGALS9 | 1.05118 | 4.250576 | 12.9521 | 4.45E-33 | 4.21E-31 | 64.3186 |
| PLD4 | 1.05292 | 3.472455 | 11.121 | 1.07E-25 | 4.52E-24 | 47.4967 |
| CEBPD | 1.05297 | 4.930612 | 10.2207 | 2.66E-22 | 7.87E-21 | 39.7716 |
| SLN | 1.05479 | 2.507671 | 5.97822 | 4.43E-09 | 3.12E-08 | 9.86632 |
| CD86 | 1.05483 | 3.361083 | 11.9866 | 4.10E-29 | 2.51E-27 | 55.2792 |
| CP | 1.05548 | 2.598955 | 7.57833 | 1.84E-13 | 2.20E-12 | 19.7211 |
| PRAM1 | 1.06033 | 2.587698 | 13.6073 | 7.53E-36 | 9.70E-34 | 70.642 |
| SERPINH1 | 1.06102 | 4.26308 | 11.6622 | 8.13E-28 | 4.30E-26 | 52.3247 |
| ANKRD35 | 1.06333 | 2.915638 | 9.62113 | 3.85E-20 | 9.27E-19 | 34.8587 |
| SFRP4 | 1.06527 | 2.894937 | 8.36761 | 6.59E-16 | 1.02E-14 | 25.2557 |
| CD300A | 1.06564 | 3.567446 | 13.4714 | 2.86E-35 | 3.46E-33 | 69.3187 |
| FLNA | 1.06572 | 6.569307 | 13.1753 | 5.14E-34 | 5.52E-32 | 66.4569 |
| EVI2B | 1.06657 | 4.072346 | 11.3671 | 1.18E-26 | 5.47E-25 | 49.6764 |
| ASAP3 | 1.06714 | 5.119209 | 16.0161 | 1.72E-46 | 6.29E-44 | 94.9315 |
| LPAR5 | 1.0674 | 3.849198 | 11.5551 | 2.16E-27 | 1.09E-25 | 51.3592 |
| SAMSN1 | 1.06766 | 3.303151 | 11.842 | 1.56E-28 | 8.94E-27 | 53.9567 |
| RAB32 | 1.06768 | 3.924004 | 14.0561 | 8.78E-38 | 1.43E-35 | 75.0526 |
| TNFAIP8L2 | 1.0678 | 3.872753 | 13.5743 | 1.04E-35 | 1.32E-33 | 70.3203 |
| BCL2L12 | 1.06789 | 2.355691 | 19.2088 | 2.68E-61 | 3.86E-58 | 128.751 |
| PCED1B-AS1 | 1.06934 | 2.595617 | 14.5065 | 9.53E-40 | 1.89E-37 | 79.5366 |
| H2BC12 | 1.06943 | 4.44261 | 13.9543 | 2.42E-37 | 3.67E-35 | 74.0465 |
| ARPC1B | 1.07031 | 5.08166 | 15.693 | 4.99E-45 | 1.62E-42 | 91.594 |
| CSF1R | 1.07129 | 6.517739 | 10.4162 | 5.03E-23 | 1.61E-21 | 41.4149 |
| MSN | 1.07153 | 6.114863 | 10.9006 | 7.52E-25 | 2.92E-23 | 45.5693 |
| RGS1 | 1.07185 | 4.749352 | 7.18502 | 2.61E-12 | 2.74E-11 | 17.1231 |
| RHOC | 1.07648 | 6.786233 | 19.4671 | 1.62E-62 | 2.47E-59 | 131.533 |
| HSPA6 | 1.07745 | 2.065184 | 8.9272 | 9.53E-18 | 1.80E-16 | 29.4252 |
| LY86 | 1.07784 | 4.944714 | 11.5982 | 1.46E-27 | 7.50E-26 | 51.747 |
| HOXD8 | 1.07889 | 1.664381 | 7.68665 | 8.72E-14 | 1.08E-12 | 20.4556 |
| S100A6 | 1.08212 | 8.797642 | 11.7518 | 3.58E-28 | 1.98E-26 | 53.1361 |
| TFPI | 1.0829 | 1.817988 | 10.5645 | 1.41E-23 | 4.76E-22 | 42.6744 |
| GLIS3 | 1.08343 | 3.087758 | 11.7583 | 3.37E-28 | 1.86E-26 | 53.1951 |
| RUNX1 | 1.08622 | 1.738724 | 13.71 | 2.73E-36 | 3.72E-34 | 71.6463 |
| FNBP1L | 1.08687 | 4.42346 | 13.0659 | 1.48E-33 | 1.50E-31 | 65.4063 |
| IL18 | 1.08912 | 3.368528 | 12.1965 | 5.81E-30 | 3.84E-28 | 57.214 |
| FCGR1A | 1.09201 | 3.407526 | 11.5399 | 2.47E-27 | 1.24E-25 | 51.2227 |
| AEBP1 | 1.09419 | 5.530841 | 6.15794 | 1.57E-09 | 1.16E-08 | 10.876 |
| COL1A1 | 1.09502 | 2.61381 | 7.33612 | 9.55E-13 | 1.05E-11 | 18.1082 |
| CD58 | 1.09606 | 2.953826 | 14.0159 | 1.31E-37 | 2.06E-35 | 74.6545 |
| CCR1 | 1.09713 | 3.478667 | 11.4962 | 3.68E-27 | 1.81E-25 | 50.8302 |
| OLFML3 | 1.09737 | 5.125341 | 12.6287 | 9.84E-32 | 7.82E-30 | 61.2521 |
| PYCARD | 1.09771 | 3.483998 | 14.5876 | 4.20E-40 | 8.64E-38 | 80.3498 |
| HSPA7 | 1.09949 | 1.531783 | 10.3065 | 1.28E-22 | 3.94E-21 | 40.4903 |
| COL4A1 | 1.10072 | 4.092979 | 7.39476 | 6.44E-13 | 7.26E-12 | 18.4948 |
| IFI44L | 1.10474 | 3.874662 | 9.05211 | 3.61E-18 | 7.13E-17 | 30.3823 |
| RNASE6 | 1.10516 | 4.821997 | 12.4027 | 8.38E-31 | 6.10E-29 | 59.1314 |
| AR | 1.10823 | 2.319746 | 11.7046 | 5.52E-28 | 3.00E-26 | 52.708 |
| HOXA5 | 1.10871 | 0.731893 | 8.96001 | 7.39E-18 | 1.42E-16 | 29.6757 |
| HAS2 | 1.11122 | 2.453512 | 11.3878 | 9.80E-27 | 4.57E-25 | 49.8608 |
| CD44 | 1.11284 | 5.676043 | 8.76728 | 3.26E-17 | 5.80E-16 | 28.2137 |
| AURKB | 1.11454 | 2.134077 | 9.23685 | 8.43E-19 | 1.77E-17 | 31.815 |
| SLC2A5 | 1.11508 | 4.284671 | 11.5035 | 3.44E-27 | 1.70E-25 | 50.8959 |
| S1PR3 | 1.11601 | 3.477774 | 10.826 | 1.45E-24 | 5.44E-23 | 44.9223 |
| GBP3 | 1.11792 | 3.427299 | 9.2353 | 8.54E-19 | 1.79E-17 | 31.8028 |
| OR4N2 | 1.11821 | 1.355673 | 8.67592 | 6.54E-17 | 1.13E-15 | 27.5287 |
| DEF6 | 1.11832 | 3.519606 | 15.0456 | 3.95E-42 | 9.63E-40 | 84.9747 |
| TCIM | 1.12107 | 4.065146 | 9.19798 | 1.15E-18 | 2.38E-17 | 31.5118 |
| CX3CR1 | 1.12207 | 5.467129 | 8.10489 | 4.50E-15 | 6.37E-14 | 23.3672 |
| TMIGD3 | 1.12593 | 4.505795 | 12.2783 | 2.70E-30 | 1.85E-28 | 57.9726 |
| SPATA6 | 1.12616 | 3.373629 | 11.0097 | 2.87E-25 | 1.17E-23 | 46.5211 |
| LILRB4 | 1.12623 | 3.819589 | 12.1455 | 9.36E-30 | 6.07E-28 | 56.742 |
| HCST | 1.12857 | 3.661806 | 12.9701 | 3.74E-33 | 3.58E-31 | 64.4902 |
| RGS16 | 1.13073 | 3.364377 | 9.80209 | 8.75E-21 | 2.25E-19 | 36.321 |
| LAT2 | 1.13098 | 3.707573 | 14.3464 | 4.79E-39 | 8.96E-37 | 77.9361 |
| PTAFR | 1.13187 | 3.574585 | 12.1099 | 1.31E-29 | 8.39E-28 | 56.4133 |
| CD37 | 1.13279 | 3.774175 | 12.4546 | 5.13E-31 | 3.84E-29 | 59.6168 |
| ABI3 | 1.13569 | 4.015679 | 13.5521 | 1.30E-35 | 1.62E-33 | 70.1038 |
| LAIR1 | 1.13634 | 3.112436 | 13.1745 | 5.18E-34 | 5.56E-32 | 66.4495 |
| CD53 | 1.13662 | 5.557701 | 12.5035 | 3.23E-31 | 2.47E-29 | 60.0743 |
| HAVCR2 | 1.13784 | 4.238815 | 12.4945 | 3.52E-31 | 2.67E-29 | 59.9905 |
| PIFO | 1.14434 | 3.033908 | 11.3474 | 1.41E-26 | 6.46E-25 | 49.5003 |
| HAMP | 1.1445 | 1.889638 | 10.2529 | 2.02E-22 | 6.07E-21 | 40.0412 |
| BIRC5 | 1.14505 | 2.716624 | 8.41918 | 4.49E-16 | 7.08E-15 | 25.6317 |
| ALOX5 | 1.14629 | 3.244256 | 13.0431 | 1.85E-33 | 1.85E-31 | 65.1878 |
| COL8A2 | 1.1463 | 2.534734 | 12.3361 | 1.57E-30 | 1.10E-28 | 58.51 |
| C1orf162 | 1.14752 | 3.688251 | 14.2868 | 8.72E-39 | 1.60E-36 | 77.3421 |
| C3AR1 | 1.1482 | 5.009086 | 11.229 | 4.08E-26 | 1.79E-24 | 48.4496 |
| MSR1 | 1.1488 | 2.524604 | 11.1942 | 5.57E-26 | 2.42E-24 | 48.1424 |
| TSPO | 1.15004 | 5.969866 | 14.0555 | 8.84E-38 | 1.43E-35 | 75.046 |
| HOTAIRM1 | 1.15569 | 1.468628 | 9.89894 | 3.93E-21 | 1.04E-19 | 37.1109 |
| CASP1 | 1.15587 | 2.838972 | 14.7087 | 1.23E-40 | 2.63E-38 | 81.5677 |
| MYBL2 | 1.16016 | 2.573085 | 8.00989 | 8.91E-15 | 1.22E-13 | 22.6955 |
| AQP1 | 1.16246 | 7.46253 | 6.29342 | 7.06E-10 | 5.45E-09 | 11.654 |
| C4B | 1.16344 | 3.275377 | 10.2283 | 2.49E-22 | 7.41E-21 | 39.8357 |
| HOXD9 | 1.16472 | 0.98933 | 9.48964 | 1.12E-19 | 2.56E-18 | 33.8077 |
| ABCC3 | 1.16586 | 1.130626 | 9.55993 | 6.33E-20 | 1.50E-18 | 34.3683 |
| HSPB6 | 1.16633 | 4.617853 | 8.7896 | 2.75E-17 | 4.93E-16 | 28.3818 |
| HK2 | 1.16795 | 3.805019 | 13.9995 | 1.55E-37 | 2.40E-35 | 74.4928 |
| GEM | 1.16942 | 4.202539 | 10.4612 | 3.42E-23 | 1.11E-21 | 41.7964 |
| AIF1 | 1.17004 | 6.08579 | 13.2445 | 2.63E-34 | 2.92E-32 | 67.1227 |
| SLC1A5 | 1.17005 | 3.728038 | 14.5545 | 5.86E-40 | 1.18E-37 | 80.0181 |
| CYBA | 1.17158 | 4.831948 | 14.9278 | 1.32E-41 | 3.06E-39 | 83.7799 |
| CCL2 | 1.17381 | 4.200507 | 7.85531 | 2.67E-14 | 3.52E-13 | 21.6155 |
| CLIC1 | 1.17518 | 5.867621 | 13.165 | 5.68E-34 | 6.06E-32 | 66.3579 |
| SERPINE1 | 1.17582 | 3.683263 | 7.1752 | 2.79E-12 | 2.91E-11 | 17.0597 |
| UCP2 | 1.17682 | 4.93057 | 13.046 | 1.80E-33 | 1.80E-31 | 65.2164 |
| DNALI1 | 1.1797 | 4.493911 | 16.2916 | 9.62E-48 | 4.02E-45 | 97.793 |
| CCN1 | 1.18078 | 5.234555 | 8.0834 | 5.25E-15 | 7.40E-14 | 23.2148 |
| MRC2 | 1.18092 | 4.655127 | 12.4738 | 4.28E-31 | 3.22E-29 | 59.7962 |
| PTGS1 | 1.1837 | 3.275174 | 13.2747 | 1.95E-34 | 2.23E-32 | 67.4147 |
| HLA-DQB1 | 1.18774 | 3.714582 | 8.28518 | 1.21E-15 | 1.82E-14 | 24.6583 |
| APBB1IP | 1.1878 | 4.307275 | 12.4998 | 3.35E-31 | 2.54E-29 | 60.0401 |
| HCLS1 | 1.1894 | 4.721459 | 13.4001 | 5.75E-35 | 6.79E-33 | 68.6271 |
| LAPTM5 | 1.19011 | 7.309949 | 13.0044 | 2.69E-33 | 2.63E-31 | 64.8182 |
| AC026401.3 | 1.19139 | 3.224583 | 15.5086 | 3.37E-44 | 1.05E-41 | 89.699 |
| TGIF1 | 1.1973 | 2.741515 | 16.8646 | 2.28E-50 | 1.25E-47 | 103.786 |
| OLFML2B | 1.19942 | 3.547267 | 11.5681 | 1.92E-27 | 9.75E-26 | 51.4758 |
| CD163 | 1.20131 | 3.178005 | 7.29512 | 1.26E-12 | 1.37E-11 | 17.8393 |
| S100A10 | 1.20271 | 6.527917 | 10.2386 | 2.28E-22 | 6.80E-21 | 39.9216 |
| FAM181A | 1.20461 | 2.804585 | 8.78941 | 2.75E-17 | 4.94E-16 | 28.3804 |
| SPI1 | 1.20507 | 5.072036 | 14.258 | 1.16E-38 | 2.09E-36 | 77.056 |
| C1R | 1.20523 | 4.910229 | 9.98848 | 1.87E-21 | 5.10E-20 | 37.8459 |
| CAPG | 1.20712 | 5.322924 | 11.3802 | 1.05E-26 | 4.88E-25 | 49.7927 |
| MS4A6A | 1.21076 | 3.395942 | 10.1903 | 3.43E-22 | 1.01E-20 | 39.5178 |
| SASH3 | 1.21134 | 3.91628 | 14.0919 | 6.14E-38 | 1.02E-35 | 75.4069 |
| HOXC4 | 1.2157 | 1.381108 | 13.423 | 4.59E-35 | 5.50E-33 | 68.8497 |
| CTSH | 1.21583 | 5.181534 | 10.1547 | 4.63E-22 | 1.34E-20 | 39.2219 |
| AL354919.2 | 1.21678 | 1.21668 | 10.7732 | 2.30E-24 | 8.43E-23 | 44.4653 |
| FCGR2A | 1.21699 | 3.419206 | 13.1043 | 1.02E-33 | 1.06E-31 | 65.7748 |
| HLA-DMA | 1.22032 | 5.169884 | 12.4877 | 3.76E-31 | 2.84E-29 | 59.9262 |
| CDCA8 | 1.22276 | 2.287186 | 12.2675 | 2.99E-30 | 2.03E-28 | 57.8721 |
| C1orf194 | 1.22435 | 2.871867 | 11.5432 | 2.40E-27 | 1.20E-25 | 51.2518 |
| HLA-DPA1 | 1.22531 | 5.392753 | 9.04449 | 3.83E-18 | 7.54E-17 | 30.3237 |
| ANKRD22 | 1.23065 | 2.425002 | 9.96419 | 2.29E-21 | 6.19E-20 | 37.6461 |
| PYGL | 1.23166 | 3.859163 | 15.3006 | 2.88E-43 | 8.19E-41 | 87.5705 |
| S100A4 | 1.23303 | 3.284167 | 10.2515 | 2.05E-22 | 6.14E-21 | 40.0293 |
| PI16 | 1.23692 | 4.395808 | 7.78243 | 4.47E-14 | 5.74E-13 | 21.1119 |
| NNMT | 1.23713 | 2.000651 | 9.74385 | 1.41E-20 | 3.56E-19 | 35.8483 |
| TUBB6 | 1.23742 | 3.666378 | 13.0733 | 1.38E-33 | 1.41E-31 | 65.4776 |
| PLAU | 1.23815 | 2.708551 | 10.7005 | 4.33E-24 | 1.56E-22 | 43.8389 |
| TMEM119 | 1.23867 | 4.639308 | 11.0891 | 1.42E-25 | 5.96E-24 | 47.2168 |
| TGFB2-AS1 | 1.24323 | 2.650242 | 11.3029 | 2.11E-26 | 9.49E-25 | 49.1048 |
| CXCL10 | 1.24502 | 1.8355 | 8.35397 | 7.29E-16 | 1.12E-14 | 25.1566 |
| TYROBP | 1.24529 | 6.99539 | 14.0803 | 6.90E-38 | 1.14E-35 | 75.2915 |
| HLA-DMB | 1.24701 | 3.977446 | 12.6839 | 5.82E-32 | 4.68E-30 | 61.7729 |
| HS3ST1 | 1.25267 | 2.571412 | 12.3609 | 1.24E-30 | 8.85E-29 | 58.741 |
| HLA-DOA | 1.25425 | 3.467159 | 9.89535 | 4.05E-21 | 1.07E-19 | 37.0815 |
| CYTL1 | 1.25696 | 3.571733 | 13.5299 | 1.61E-35 | 2.00E-33 | 69.8875 |
| F2RL1 | 1.25867 | 2.280382 | 11.9734 | 4.64E-29 | 2.82E-27 | 55.1576 |
| SCIN | 1.25872 | 2.56451 | 11.1506 | 8.22E-26 | 3.51E-24 | 47.7574 |
| FLNC | 1.25928 | 3.533048 | 9.12697 | 2.01E-18 | 4.08E-17 | 30.9604 |
| TLR2 | 1.26102 | 3.261778 | 12.351 | 1.36E-30 | 9.67E-29 | 58.6488 |
| TGFB2 | 1.26111 | 3.319504 | 9.24992 | 7.60E-19 | 1.60E-17 | 31.9171 |
| OLR1 | 1.26114 | 4.521389 | 10.1264 | 5.88E-22 | 1.69E-20 | 38.986 |
| GBP1 | 1.26429 | 3.628924 | 10.4253 | 4.65E-23 | 1.49E-21 | 41.4925 |
| DIRAS3 | 1.26847 | 3.064684 | 11.3613 | 1.25E-26 | 5.74E-25 | 49.6244 |
| VSIG4 | 1.26857 | 5.965604 | 10.7464 | 2.90E-24 | 1.06E-22 | 44.2346 |
| LINC02732 | 1.2689 | 1.703397 | 11.792 | 2.47E-28 | 1.39E-26 | 53.5011 |
| HLA-DQA1 | 1.27908 | 2.838389 | 8.96934 | 6.87E-18 | 1.32E-16 | 29.7471 |
| C1QC | 1.28351 | 8.251769 | 13.0581 | 1.60E-33 | 1.62E-31 | 65.3322 |
| TPTEP1 | 1.28508 | 2.890308 | 6.27467 | 7.89E-10 | 6.06E-09 | 11.5455 |
| ANXA2 | 1.28766 | 4.052747 | 12.3114 | 1.98E-30 | 1.37E-28 | 58.2802 |
| FCER1G | 1.28837 | 6.051402 | 13.7795 | 1.37E-36 | 1.94E-34 | 72.3275 |
| ITGB2 | 1.29979 | 4.912263 | 13.4226 | 4.61E-35 | 5.51E-33 | 68.8455 |
| C1QA | 1.30088 | 7.805767 | 13.0852 | 1.23E-33 | 1.26E-31 | 65.5914 |
| GNG5 | 1.30776 | 6.620684 | 19.8725 | 1.97E-64 | 3.73E-61 | 135.906 |
| MMP14 | 1.30987 | 5.13582 | 12.8217 | 1.56E-32 | 1.35E-30 | 63.0778 |
| HLA-DPB1 | 1.30994 | 6.011524 | 9.95191 | 2.53E-21 | 6.83E-20 | 37.5452 |
| COL11A1 | 1.31034 | 2.706451 | 10.5012 | 2.43E-23 | 8.05E-22 | 42.1353 |
| SULF1 | 1.31177 | 3.206292 | 9.68853 | 2.22E-20 | 5.47E-19 | 35.4012 |
| ECM2 | 1.31207 | 3.240608 | 11.6994 | 5.78E-28 | 3.13E-26 | 52.6614 |
| IRX1 | 1.31375 | 3.122307 | 8.11995 | 4.03E-15 | 5.74E-14 | 23.4742 |
| CYBB | 1.31485 | 4.897517 | 12.4047 | 8.23E-31 | 6.00E-29 | 59.1496 |
| CD14 | 1.315 | 6.385504 | 11.9117 | 8.21E-29 | 4.83E-27 | 54.5926 |
| FPR1 | 1.32246 | 3.922619 | 11.0144 | 2.76E-25 | 1.12E-23 | 46.5615 |
| UBE2C | 1.32454 | 3.433359 | 8.88456 | 1.33E-17 | 2.46E-16 | 29.1006 |
| SPP1 | 1.32993 | 8.954714 | 9.12119 | 2.10E-18 | 4.26E-17 | 30.9157 |
| STEAP3 | 1.3319 | 3.119714 | 11.4325 | 6.55E-27 | 3.12E-25 | 50.2593 |
| NFIA-AS2 | 1.33414 | 3.454964 | 12.9144 | 6.40E-33 | 5.97E-31 | 63.959 |
| TYMP | 1.33481 | 3.084205 | 11.6757 | 7.19E-28 | 3.83E-26 | 52.4463 |
| TNC | 1.3373 | 5.808766 | 11.1281 | 1.00E-25 | 4.26E-24 | 47.5591 |
| RPE65 | 1.34347 | 3.469416 | 9.94366 | 2.71E-21 | 7.29E-20 | 37.4775 |
| CA3 | 1.34459 | 1.57169 | 11.366 | 1.19E-26 | 5.52E-25 | 49.6662 |
| C1QB | 1.35099 | 8.076688 | 12.9593 | 4.15E-33 | 3.94E-31 | 64.3877 |
| SLC11A1 | 1.3521 | 2.618899 | 12.3096 | 2.01E-30 | 1.39E-28 | 58.263 |
| KIF2C | 1.3597 | 2.335134 | 13.2973 | 1.57E-34 | 1.80E-32 | 67.6328 |
| GBP2 | 1.3726 | 3.738614 | 10.9218 | 6.24E-25 | 2.44E-23 | 45.7535 |
| LEFTY2 | 1.37346 | 1.639638 | 10.1938 | 3.33E-22 | 9.77E-21 | 39.5475 |
| RNASE2 | 1.3779 | 2.515737 | 11.9172 | 7.80E-29 | 4.60E-27 | 54.6435 |
| S100A3 | 1.37811 | 2.037544 | 11.2522 | 3.32E-26 | 1.47E-24 | 48.6554 |
| IGFBP2 | 1.37975 | 3.750105 | 8.74683 | 3.81E-17 | 6.71E-16 | 28.0599 |
| LTF | 1.381 | 1.663225 | 7.12388 | 3.91E-12 | 4.02E-11 | 16.7292 |
| FOXJ1 | 1.38601 | 3.476257 | 10.7862 | 2.05E-24 | 7.56E-23 | 44.5779 |
| LINC01736 | 1.38648 | 4.483873 | 10.9674 | 4.17E-25 | 1.66E-23 | 46.1513 |
| ID3 | 1.38949 | 8.168403 | 12.8267 | 1.49E-32 | 1.29E-30 | 63.1251 |
| TREM2 | 1.39232 | 6.640954 | 12.8074 | 1.79E-32 | 1.54E-30 | 62.9418 |
| LINC02381 | 1.39409 | 3.792466 | 14.1379 | 3.88E-38 | 6.56E-36 | 75.8625 |
| SERPINA1 | 1.39519 | 3.190844 | 12.7632 | 2.73E-32 | 2.29E-30 | 62.5231 |
| EMP1 | 1.40688 | 4.955281 | 11.578 | 1.75E-27 | 8.95E-26 | 51.5652 |
| FOXD1 | 1.41141 | 2.71331 | 13.5617 | 1.18E-35 | 1.48E-33 | 70.1974 |
| EMP3 | 1.41244 | 3.60591 | 9.24295 | 8.04E-19 | 1.69E-17 | 31.8626 |
| S100A11 | 1.41573 | 6.872417 | 14.4546 | 1.61E-39 | 3.12E-37 | 79.0172 |
| CDC20 | 1.41629 | 2.845754 | 11.9477 | 5.89E-29 | 3.52E-27 | 54.9221 |
| APOL4 | 1.41662 | 2.255877 | 11.2383 | 3.75E-26 | 1.65E-24 | 48.5326 |
| HLA-DRB1 | 1.41696 | 7.561012 | 10.4708 | 3.15E-23 | 1.03E-21 | 41.8774 |
| CD74 | 1.4276 | 9.048877 | 11.8693 | 1.21E-28 | 7.03E-27 | 54.206 |
| FOXD3-AS1 | 1.42916 | 1.730808 | 12.2239 | 4.50E-30 | 3.02E-28 | 57.4681 |
| METTL7B | 1.44422 | 4.005341 | 8.77809 | 3.00E-17 | 5.35E-16 | 28.2951 |
| GNG12 | 1.44509 | 4.659585 | 13.0559 | 1.63E-33 | 1.65E-31 | 65.3111 |
| TIMP1 | 1.44518 | 5.13141 | 8.94132 | 8.54E-18 | 1.63E-16 | 29.5329 |
| ITGB4 | 1.45716 | 5.060992 | 11.5911 | 1.55E-27 | 7.97E-26 | 51.6833 |
| HLA-DRA | 1.46829 | 8.54253 | 10.4757 | 3.02E-23 | 9.88E-22 | 41.9192 |
| HLA-DRB5 | 1.50144 | 5.979075 | 10.318 | 1.16E-22 | 3.58E-21 | 40.5871 |
| CRNDE | 1.50544 | 1.865574 | 13.6444 | 5.22E-36 | 6.91E-34 | 71.0045 |
| C3 | 1.53245 | 7.019144 | 12.1156 | 1.24E-29 | 7.98E-28 | 56.4662 |
| VCAM1 | 1.55253 | 3.965962 | 11.0306 | 2.39E-25 | 9.79E-24 | 46.7038 |
| PLEKHA4 | 1.55426 | 4.240709 | 13.703 | 2.93E-36 | 3.96E-34 | 71.5774 |
| ALOX5AP | 1.56035 | 4.593455 | 14.0797 | 6.94E-38 | 1.14E-35 | 75.2855 |
| FCGR3A | 1.5704 | 5.167167 | 12.4245 | 6.83E-31 | 5.02E-29 | 59.3347 |
| EMILIN3 | 1.59996 | 2.07 | 10.2237 | 2.59E-22 | 7.67E-21 | 39.7973 |
| SPOCD1 | 1.60687 | 2.027913 | 11.3739 | 1.11E-26 | 5.16E-25 | 49.7368 |
| NAPSB | 1.6787 | 3.712827 | 11.6919 | 6.20E-28 | 3.33E-26 | 52.593 |
| TNFRSF12A | 1.68192 | 3.715604 | 12.976 | 3.53E-33 | 3.40E-31 | 64.547 |
| ANXA1 | 1.68642 | 4.41343 | 10.8604 | 1.07E-24 | 4.08E-23 | 45.22 |
| CHI3L2 | 1.7195 | 3.834745 | 10.1305 | 5.68E-22 | 1.63E-20 | 39.0207 |
| FCGBP | 1.77295 | 3.633422 | 11.831 | 1.73E-28 | 9.88E-27 | 53.8567 |
| CHI3L1 | 1.8053 | 4.577935 | 7.0173 | 7.83E-12 | 7.73E-11 | 16.0491 |
| VIM | 1.93832 | 8.379418 | 16.3842 | 3.64E-48 | 1.54E-45 | 98.7577 |
| PDPN | 2.00568 | 3.571739 | 12.9076 | 6.83E-33 | 6.35E-31 | 63.8943 |
