# Supplementary material for: MAGOH is correlated with poor prognosis and is essential for cell proliferation in lower-grade glioma
Source: Aging (Albany NY). 2023 Jun 30;15(12):5713–33. doi: 10.18632/aging.204823 (PMC10333088; doi:10.18632/aging.204823)
Supplement: Supplementary Table 3 [file aging-15-204823-s004.docx]

**Supplementary Table 3. Down-regulated DEGs in CGGA cohort.**

| **id** | **logFC** | **AveExpr** | **t** | **P.Value** | **adj.P.Val** | **B** |
| --- | --- | --- | --- | --- | --- | --- |
| KCNIP2 | -2.21717 | 4.9201816 | -10.672866 | 1.06E-20 | 2.22E-18 | 36.4357 |
| SVOP | -2.18128 | 3.450028 | -8.9409311 | 6.12E-16 | 4.51E-14 | 25.669 |
| NSG2 | -2.15098 | 6.0796313 | -8.8463518 | 1.10E-15 | 7.55E-14 | 25.0981 |
| CPLX2 | -2.0817 | 5.0794592 | -7.4422571 | 4.62E-12 | 1.25E-10 | 16.9235 |
| TRIM67 | -2.08155 | 2.1270493 | -9.9469187 | 1.12E-18 | 1.48E-16 | 31.8612 |
| RP5-1119A7.17 | -2.064 | 2.9019826 | -10.869794 | 2.97E-21 | 7.68E-19 | 37.6879 |
| CHGA | -2.03424 | 4.5319987 | -7.8720669 | 3.83E-13 | 1.39E-11 | 19.3589 |
| CHGB | -2.02784 | 5.3063359 | -9.184685 | 1.35E-16 | 1.12E-14 | 27.1503 |
| GABRG2 | -2.0229 | 3.1674477 | -8.3401912 | 2.38E-14 | 1.16E-12 | 22.0819 |
| SCRT1 | -2.00095 | 3.0441119 | -10.876453 | 2.84E-21 | 7.51E-19 | 37.7303 |
| INA | -1.93956 | 4.5167221 | -7.6925488 | 1.09E-12 | 3.50E-11 | 18.3337 |
| CACNG2 | -1.93863 | 2.3068145 | -11.776098 | 8.07E-24 | 3.39E-21 | 43.4937 |
| L1CAM | -1.93598 | 4.1635438 | -8.5523307 | 6.60E-15 | 3.73E-13 | 23.3375 |
| TNR | -1.9 | 5.5165752 | -7.9272053 | 2.77E-13 | 1.03E-11 | 19.676 |
| RP11-47I22.1 | -1.87648 | 2.1788923 | -8.7770358 | 1.68E-15 | 1.09E-13 | 24.681 |
| PRLHR | -1.84713 | 2.1692083 | -8.2444311 | 4.22E-14 | 1.93E-12 | 21.5193 |
| SFRP2 | -1.83606 | 4.9822934 | -5.1450423 | 7.28E-07 | 4.97E-06 | 5.30042 |
| VSTM2A | -1.82376 | 3.859622 | -7.6846066 | 1.14E-12 | 3.63E-11 | 18.2886 |
| SHISA6 | -1.80562 | 2.6326203 | -8.2950086 | 3.12E-14 | 1.46E-12 | 21.8161 |
| SEZ6L | -1.80019 | 5.6756307 | -8.3096968 | 2.85E-14 | 1.36E-12 | 21.9024 |
| SCN3B | -1.78361 | 4.3015021 | -9.3129364 | 6.08E-17 | 5.46E-15 | 27.9349 |
| MMD2 | -1.77374 | 4.0022726 | -9.8597038 | 1.94E-18 | 2.41E-16 | 31.3168 |
| SNAP25 | -1.7687 | 6.8325729 | -5.8463286 | 2.49E-08 | 2.47E-07 | 8.55909 |
| SNCB | -1.75631 | 5.084756 | -6.281868 | 2.68E-09 | 3.42E-08 | 10.7216 |
| KCNJ11 | -1.74969 | 3.3232061 | -10.201587 | 2.20E-19 | 3.43E-17 | 33.4577 |
| CPLX1 | -1.74573 | 4.7568082 | -8.9404228 | 6.14E-16 | 4.51E-14 | 25.6659 |
| GPR17 | -1.74445 | 4.6760715 | -6.1066341 | 6.65E-09 | 7.63E-08 | 9.83956 |
| F5 | -1.744 | 2.3350256 | -6.5612381 | 6.11E-10 | 9.07E-09 | 12.1593 |
| NRSN1 | -1.71532 | 4.6687175 | -9.9417114 | 1.15E-18 | 1.52E-16 | 31.8287 |
| ACTL6B | -1.7069 | 3.9384538 | -8.1583201 | 7.05E-14 | 3.11E-12 | 21.0158 |
| GRIN1 | -1.70048 | 4.041773 | -5.7164275 | 4.75E-08 | 4.40E-07 | 7.93392 |
| ABCC8 | -1.69717 | 4.4203479 | -8.2886442 | 3.24E-14 | 1.51E-12 | 21.7787 |
| TMEM151B | -1.68148 | 3.8745835 | -9.3574992 | 4.60E-17 | 4.29E-15 | 28.2084 |
| STMN2 | -1.66535 | 5.3338976 | -5.0534838 | 1.11E-06 | 7.23E-06 | 4.89696 |
| SYN1 | -1.6576 | 4.7498719 | -7.3695808 | 7.00E-12 | 1.81E-10 | 16.5184 |
| SYT4 | -1.6554 | 3.2146148 | -7.3680773 | 7.06E-12 | 1.82E-10 | 16.51 |
| AC062021.1 | -1.64557 | 2.1552641 | -7.045581 | 4.34E-11 | 8.99E-10 | 14.7378 |
| RPH3A | -1.64536 | 3.8717212 | -6.8890824 | 1.03E-10 | 1.90E-09 | 13.8933 |
| HRH3 | -1.64499 | 2.1502264 | -9.6067532 | 9.62E-18 | 1.04E-15 | 29.7453 |
| CELF3 | -1.64301 | 4.5880023 | -8.6622012 | 3.38E-15 | 2.06E-13 | 23.9928 |
| GABRB3 | -1.61153 | 4.1053207 | -8.7523506 | 1.95E-15 | 1.24E-13 | 24.5328 |
| CALN1 | -1.60735 | 3.0504633 | -8.3728239 | 1.95E-14 | 9.75E-13 | 22.2742 |
| SELL | -1.60526 | 4.7330823 | -5.4193573 | 2.01E-07 | 1.57E-06 | 6.5404 |
| MYT1L | -1.60339 | 3.0472365 | -7.1344342 | 2.64E-11 | 5.79E-10 | 15.2219 |
| SCG3 | -1.58386 | 7.472059 | -8.2816746 | 3.38E-14 | 1.57E-12 | 21.7378 |
| ZDHHC22 | -1.5829 | 5.2202294 | -7.9829954 | 2.00E-13 | 7.76E-12 | 19.9978 |
| RGR | -1.5714 | 3.2701557 | -7.6119457 | 1.74E-12 | 5.35E-11 | 17.877 |
| ATCAY | -1.57017 | 5.3945999 | -7.6111161 | 1.75E-12 | 5.37E-11 | 17.8724 |
| JPH3 | -1.55777 | 4.2928494 | -8.0957953 | 1.02E-13 | 4.37E-12 | 20.6517 |
| HAPLN1 | -1.5559 | 3.6314393 | -6.769655 | 1.98E-10 | 3.38E-09 | 13.256 |
| GDAP1L1 | -1.54669 | 5.4662942 | -7.9636399 | 2.24E-13 | 8.61E-12 | 19.886 |
| SHD | -1.54347 | 5.7567312 | -5.5807585 | 9.23E-08 | 7.90E-07 | 7.29116 |
| CKMT1B | -1.54206 | 3.4795557 | -7.5346661 | 2.72E-12 | 7.92E-11 | 17.4414 |
| ACBD7 | -1.54091 | 3.6104045 | -7.4115271 | 5.51E-12 | 1.46E-10 | 16.7519 |
| CA10 | -1.5376 | 4.0125807 | -6.4696133 | 9.96E-10 | 1.40E-08 | 11.6836 |
| TNNT1 | -1.5336 | 2.5301538 | -8.2589134 | 3.87E-14 | 1.79E-12 | 21.6042 |
| SLC25A48 | -1.52946 | 4.5245341 | -6.664411 | 3.51E-10 | 5.55E-09 | 12.6997 |
| SLC17A7 | -1.525 | 4.4557609 | -4.0151718 | 8.88E-05 | 0.0003588 | 0.7167 |
| CRTAC1 | -1.52157 | 4.6333223 | -7.6845853 | 1.14E-12 | 3.63E-11 | 18.2885 |
| WNT7B | -1.50501 | 2.6842105 | -7.8666917 | 3.96E-13 | 1.43E-11 | 19.328 |
| CAMK2A | -1.5027 | 4.2506934 | -4.4700752 | 1.42E-05 | 7.08E-05 | 2.45547 |
| SLIT1 | -1.49884 | 4.57104 | -6.9895648 | 5.92E-11 | 1.17E-09 | 14.4343 |
| SNAP91 | -1.48297 | 4.8129661 | -8.0735767 | 1.17E-13 | 4.90E-12 | 20.5226 |
| DGCR6 | -1.47575 | 5.8398213 | -8.4912511 | 9.55E-15 | 5.20E-13 | 22.9747 |
| NRGN | -1.47532 | 6.0651471 | -4.1601474 | 5.03E-05 | 0.0002162 | 1.25442 |
| ACSL6 | -1.47439 | 4.8066517 | -9.5061555 | 1.81E-17 | 1.85E-15 | 29.1236 |
| CXXC11 | -1.46793 | 3.0677991 | -8.0212877 | 1.59E-13 | 6.33E-12 | 20.2193 |
| KIAA1644 | -1.46602 | 2.5603689 | -7.8347923 | 4.77E-13 | 1.69E-11 | 19.1451 |
| SEZ6L2 | -1.46328 | 4.9586787 | -8.2572585 | 3.91E-14 | 1.80E-12 | 21.5945 |
| SRRM3 | -1.45771 | 3.7432074 | -8.5968253 | 5.04E-15 | 2.94E-13 | 23.6025 |
| ASIC4 | -1.45462 | 4.6966039 | -5.7540201 | 3.94E-08 | 3.72E-07 | 8.11387 |
| BRINP1 | -1.45374 | 4.2091949 | -8.0359624 | 1.46E-13 | 5.88E-12 | 20.3043 |
| PACSIN1 | -1.45178 | 3.4622762 | -4.5610781 | 9.67E-06 | 5.02E-05 | 2.8211 |
| PPP2R2C | -1.44141 | 4.6637594 | -6.4153465 | 1.33E-09 | 1.81E-08 | 11.4038 |
| CDK5R2 | -1.43929 | 3.2017553 | -7.4594116 | 4.19E-12 | 1.15E-10 | 17.0194 |
| ETNPPL | -1.4382 | 5.6990469 | -4.5973964 | 8.29E-06 | 4.38E-05 | 2.96863 |
| CPNE5 | -1.43819 | 4.3203952 | -9.3868328 | 3.83E-17 | 3.61E-15 | 28.3886 |
| ATOH8 | -1.43699 | 4.7092461 | -7.453049 | 4.35E-12 | 1.19E-10 | 16.9838 |
| CKMT1A | -1.43054 | 2.9878879 | -6.7265631 | 2.51E-10 | 4.13E-09 | 13.0276 |
| DDN | -1.42769 | 3.2880277 | -4.9778263 | 1.56E-06 | 9.82E-06 | 4.5676 |
| VSNL1 | -1.41047 | 4.6460953 | -3.782778 | 0.000214 | 0.0007812 | -0.1122 |
| GABRD | -1.41006 | 3.6581459 | -6.6509004 | 3.77E-10 | 5.90E-09 | 12.6287 |
| IQSEC3 | -1.40578 | 2.8624023 | -7.0301142 | 4.73E-11 | 9.68E-10 | 14.6539 |
| RBFOX3 | -1.40237 | 2.8135327 | -5.8772669 | 2.13E-08 | 2.15E-07 | 8.70937 |
| FAM163B | -1.40053 | 3.4217951 | -5.4322549 | 1.89E-07 | 1.49E-06 | 6.59983 |
| CELF4 | -1.39816 | 3.8480538 | -5.8170218 | 2.88E-08 | 2.81E-07 | 8.41723 |
| SYT1 | -1.39452 | 4.405864 | -4.264279 | 3.31E-05 | 0.0001493 | 1.65023 |
| GABBR1 | -1.39401 | 7.4234383 | -10.588387 | 1.83E-20 | 3.56E-18 | 35.8999 |
| FAM155A | -1.38959 | 3.7713293 | -8.7654001 | 1.80E-15 | 1.16E-13 | 24.6111 |
| GRIN3A | -1.38729 | 1.7642281 | -9.1511315 | 1.67E-16 | 1.34E-14 | 26.9456 |
| SLC7A14 | -1.38592 | 2.8163789 | -8.9502794 | 5.78E-16 | 4.27E-14 | 25.7256 |
| RASL10A | -1.38414 | 4.8872354 | -6.4263693 | 1.25E-09 | 1.72E-08 | 11.4605 |
| ALDOC | -1.38351 | 9.1630566 | -7.8143789 | 5.37E-13 | 1.87E-11 | 19.0282 |
| TMEM130 | -1.37523 | 3.9230667 | -4.8784314 | 2.43E-06 | 1.47E-05 | 4.14055 |
| CSDC2 | -1.36996 | 4.37838 | -6.8311429 | 1.42E-10 | 2.52E-09 | 13.5833 |
| PTPRN | -1.36187 | 4.719074 | -5.958536 | 1.42E-08 | 1.49E-07 | 9.10659 |
| GABRA3 | -1.35449 | 3.5093883 | -7.7616792 | 7.31E-13 | 2.44E-11 | 18.7271 |
| SHANK1 | -1.3541 | 3.4202738 | -7.356431 | 7.54E-12 | 1.92E-10 | 16.4453 |
| RP1-293L6.1 | -1.35345 | 1.5768558 | -8.9057591 | 7.60E-16 | 5.46E-14 | 25.4564 |
| FAM57B | -1.35007 | 4.2859404 | -8.6510603 | 3.62E-15 | 2.19E-13 | 23.9262 |
| SYN2 | -1.34221 | 3.6991845 | -4.5360396 | 1.08E-05 | 5.52E-05 | 2.71993 |
| AMZ1 | -1.34033 | 2.4591021 | -10.008317 | 7.55E-19 | 1.06E-16 | 32.2452 |
| HPCAL4 | -1.34008 | 4.2099522 | -5.4526399 | 1.71E-07 | 1.36E-06 | 6.69396 |
| RP11-82C23.2 | -1.33917 | 1.5018119 | -6.1833329 | 4.48E-09 | 5.36E-08 | 10.2237 |
| CAMK4 | -1.33705 | 2.4506307 | -8.5512379 | 6.64E-15 | 3.75E-13 | 23.331 |
| RAB3C | -1.33541 | 3.0406306 | -6.8507219 | 1.27E-10 | 2.30E-09 | 13.6879 |
| GNAL | -1.32184 | 3.3935198 | -9.1827419 | 1.37E-16 | 1.13E-14 | 27.1384 |
| RIMS2 | -1.31842 | 2.9080567 | -8.0540363 | 1.31E-13 | 5.39E-12 | 20.4091 |
| NTSR2 | -1.31448 | 3.910072 | -4.8453478 | 2.82E-06 | 1.67E-05 | 3.99985 |
| RUNDC3A | -1.31224 | 6.4402208 | -7.9959191 | 1.85E-13 | 7.24E-12 | 20.0725 |
| RP11-143K11.1 | -1.3082 | 2.8478106 | -7.6017427 | 1.85E-12 | 5.62E-11 | 17.8194 |
| UNC79 | -1.30419 | 2.7708983 | -10.076598 | 4.88E-19 | 6.96E-17 | 32.6729 |
| SULT4A1 | -1.30197 | 3.3629961 | -4.4095997 | 1.83E-05 | 8.81E-05 | 2.21571 |
| SMOC1 | -1.30131 | 7.0839702 | -4.5858839 | 8.70E-06 | 4.57E-05 | 2.92177 |
| CAMSAP3 | -1.30003 | 2.66385 | -9.0833963 | 2.54E-16 | 2.01E-14 | 26.5331 |
| SHANK2 | -1.29978 | 3.1110158 | -8.9172214 | 7.09E-16 | 5.10E-14 | 25.5257 |
| USH1C | -1.29858 | 4.1974055 | -4.5579482 | 9.80E-06 | 5.08E-05 | 2.80843 |
| SSTR2 | -1.29432 | 3.486178 | -7.4720303 | 3.90E-12 | 1.08E-10 | 17.09 |
| NEFM | -1.29418 | 3.6642905 | -3.9747448 | 0.000104 | 0.0004114 | 0.56956 |
| RP11-82L18.4 | -1.2863 | 3.869108 | -6.6588532 | 3.62E-10 | 5.69E-09 | 12.6705 |
| ENHO | -1.28603 | 7.0006361 | -7.2793234 | 1.17E-11 | 2.83E-10 | 16.0181 |
| ADARB2 | -1.2807 | 3.2318404 | -6.8242877 | 1.47E-10 | 2.61E-09 | 13.5468 |
| PPP1R1A | -1.26867 | 3.9927794 | -7.2305145 | 1.54E-11 | 3.60E-10 | 15.7489 |
| SYT13 | -1.26211 | 2.657985 | -5.0763692 | 9.97E-07 | 6.58E-06 | 4.9973 |
| CABP1 | -1.25747 | 3.8203384 | -4.8854208 | 2.36E-06 | 1.43E-05 | 4.17037 |
| CHRM1 | -1.2543 | 2.448056 | -6.2335813 | 3.45E-09 | 4.28E-08 | 10.4769 |
| RP11-2E11.9 | -1.25201 | 2.9656371 | -7.8291939 | 4.93E-13 | 1.74E-11 | 19.113 |
| ST8SIA3 | -1.24738 | 3.0118936 | -6.1964776 | 4.18E-09 | 5.06E-08 | 10.2898 |
| CHRNB2 | -1.23714 | 2.7216397 | -8.4919635 | 9.51E-15 | 5.19E-13 | 22.9789 |
| SLC38A1 | -1.23487 | 4.985856 | -7.3895163 | 6.25E-12 | 1.63E-10 | 16.6293 |
| CSMD3 | -1.23484 | 2.7260462 | -7.8595673 | 4.12E-13 | 1.48E-11 | 19.2871 |
| UNC13A | -1.23059 | 3.9605896 | -8.0412034 | 1.41E-13 | 5.75E-12 | 20.3347 |
| KCNK3 | -1.23049 | 2.0207261 | -8.7844762 | 1.60E-15 | 1.05E-13 | 24.7257 |
| NOG | -1.22985 | 3.2426909 | -6.8606916 | 1.20E-10 | 2.19E-09 | 13.7412 |
| CRLF1 | -1.22906 | 4.4334262 | -4.5823472 | 8.84E-06 | 4.63E-05 | 2.90739 |
| RFPL1S | -1.22241 | 2.4738811 | -6.5899295 | 5.24E-10 | 7.92E-09 | 12.3091 |
| RHBDL3 | -1.21776 | 4.5106105 | -6.9271412 | 8.36E-11 | 1.59E-09 | 14.0977 |
| CUX2 | -1.21722 | 2.016993 | -7.5129606 | 3.08E-12 | 8.84E-11 | 17.3195 |
| SLC12A5 | -1.21651 | 4.1938661 | -6.8170599 | 1.53E-10 | 2.70E-09 | 13.5082 |
| 4-Mar | -1.21617 | 1.9478801 | -7.8755887 | 3.75E-13 | 1.37E-11 | 19.3791 |
| SLC1A2 | -1.21423 | 6.5262203 | -6.693698 | 2.99E-10 | 4.83E-09 | 12.854 |
| CTD-3193O13.11 | -1.21291 | 3.5829921 | -7.5441394 | 2.58E-12 | 7.52E-11 | 17.4947 |
| RP11-231C18.1 | -1.20272 | 3.9184185 | -4.3206243 | 2.63E-05 | 0.0001218 | 1.86768 |
| AMER3 | -1.20205 | 1.7459321 | -8.7393208 | 2.11E-15 | 1.33E-13 | 24.4546 |
| SLC8A2 | -1.20164 | 3.0422151 | -6.1988114 | 4.13E-09 | 5.01E-08 | 10.3015 |
| JPH4 | -1.20015 | 5.172301 | -7.450967 | 4.40E-12 | 1.20E-10 | 16.9722 |
| NEFL | -1.19881 | 3.5365033 | -3.3995986 | 0.00084 | 0.0025993 | -1.3867 |
| UNC5A | -1.19779 | 3.3212241 | -6.9896519 | 5.92E-11 | 1.17E-09 | 14.4348 |
| RP11-229P13.23 | -1.19775 | 1.8389947 | -6.1589913 | 5.08E-09 | 5.99E-08 | 10.1014 |
| ARHGDIG | -1.19195 | 4.591288 | -5.885258 | 2.05E-08 | 2.08E-07 | 8.74827 |
| DLL3 | -1.19183 | 6.0236403 | -3.7232128 | 0.000267 | 0.0009498 | -0.3179 |
| RP11-245J24.1 | -1.19132 | 1.1698815 | -8.6502457 | 3.64E-15 | 2.20E-13 | 23.9213 |
| SEZ6 | -1.1897 | 5.3759402 | -7.2431969 | 1.43E-11 | 3.38E-10 | 15.8188 |
| SOX8 | -1.18939 | 7.5503709 | -5.1561953 | 6.91E-07 | 4.75E-06 | 5.34992 |
| RP11-1112C15.1 | -1.18289 | 1.3369414 | -3.6009373 | 0.000415 | 0.001402 | -0.7316 |
| SH3GL2 | -1.18188 | 5.2843835 | -5.4944195 | 1.40E-07 | 1.14E-06 | 6.88764 |
| SAPCD2 | -1.1816 | 3.7388578 | -6.1148981 | 6.37E-09 | 7.35E-08 | 9.8808 |
| KIF5A | -1.18105 | 6.3541679 | -6.084143 | 7.46E-09 | 8.46E-08 | 9.7275 |
| PSD | -1.18047 | 5.5628665 | -6.4080462 | 1.38E-09 | 1.87E-08 | 11.3662 |
| SLC6A17 | -1.17883 | 2.5302216 | -4.9108068 | 2.11E-06 | 1.29E-05 | 4.27894 |
| CALY | -1.17862 | 3.7892042 | -4.0691948 | 7.20E-05 | 0.0002968 | 0.91524 |
| PHYHIP | -1.17801 | 4.3819296 | -4.8048774 | 3.37E-06 | 1.96E-05 | 3.82871 |
| PTPRT | -1.17398 | 2.7470561 | -6.3058519 | 2.36E-09 | 3.06E-08 | 10.8435 |
| FLJ30594 | -1.17231 | 3.3661207 | -7.5956281 | 1.92E-12 | 5.80E-11 | 17.7849 |
| ELAVL2 | -1.16881 | 3.071027 | -6.3294554 | 2.09E-09 | 2.75E-08 | 10.9638 |
| C15orf59 | -1.16837 | 6.0471466 | -8.4072105 | 1.59E-14 | 8.16E-13 | 22.4772 |
| GALNT13 | -1.16779 | 4.9438029 | -5.378541 | 2.44E-07 | 1.87E-06 | 6.353 |
| GJB6 | -1.16551 | 2.1466279 | -4.7311589 | 4.66E-06 | 2.60E-05 | 3.51978 |
| NMNAT2 | -1.16473 | 4.2674144 | -6.9005678 | 9.68E-11 | 1.80E-09 | 13.9549 |
| SHISA9 | -1.16435 | 2.9032447 | -6.4000615 | 1.44E-09 | 1.95E-08 | 11.3252 |
| LINC00599 | -1.16423 | 3.3870694 | -6.2698846 | 2.85E-09 | 3.62E-08 | 10.6607 |
| CELF5 | -1.16373 | 3.9206603 | -6.302263 | 2.41E-09 | 3.11E-08 | 10.8252 |
| GPR27 | -1.16229 | 3.5355488 | -7.073819 | 3.70E-11 | 7.83E-10 | 14.8913 |
| RP11-307B6.3 | -1.15732 | 1.7679601 | -7.0652589 | 3.89E-11 | 8.16E-10 | 14.8447 |
| SLC24A4 | -1.15622 | 2.4113544 | -7.314396 | 9.58E-12 | 2.37E-10 | 16.2121 |
| PDE2A | -1.15552 | 4.8668716 | -6.7052432 | 2.81E-10 | 4.58E-09 | 12.9149 |
| PDZD4 | -1.15515 | 6.8708936 | -8.9827998 | 4.73E-16 | 3.57E-14 | 25.9225 |
| WSCD2 | -1.15467 | 1.9585891 | -6.9956794 | 5.72E-11 | 1.14E-09 | 14.4674 |
| CHRNA4 | -1.1546 | 2.3379585 | -7.4306997 | 4.94E-12 | 1.33E-10 | 16.8589 |
| ATP6V1G2 | -1.1529 | 6.5751264 | -8.1410674 | 7.82E-14 | 3.40E-12 | 20.9152 |
| MIR219-2 | -1.14832 | 3.7204578 | -6.0970205 | 6.98E-09 | 7.99E-08 | 9.79163 |
| RP11-161M6.2 | -1.14243 | 6.9480571 | -5.5158961 | 1.26E-07 | 1.04E-06 | 6.98761 |
| CDHR1 | -1.142 | 3.6162418 | -7.1512397 | 2.40E-11 | 5.35E-10 | 15.3138 |
| RGS7 | -1.14186 | 2.8981686 | -6.546041 | 6.63E-10 | 9.76E-09 | 12.0801 |
| EMX2OS | -1.13997 | 3.0082919 | -6.1630491 | 4.97E-09 | 5.87E-08 | 10.1218 |
| ST6GAL2 | -1.13885 | 3.2822959 | -6.4867352 | 9.10E-10 | 1.29E-08 | 11.7722 |
| GABRG1 | -1.1383 | 2.7062462 | -5.5551274 | 1.05E-07 | 8.81E-07 | 7.17091 |
| LINGO1 | -1.13445 | 6.0000429 | -7.3635711 | 7.24E-12 | 1.86E-10 | 16.485 |
| GABRA1 | -1.13238 | 2.6366277 | -3.9768954 | 0.000103 | 0.0004085 | 0.57735 |
| SLC25A18 | -1.13125 | 5.6735094 | -7.6070435 | 1.79E-12 | 5.47E-11 | 17.8493 |
| KCNJ16 | -1.13084 | 3.9069887 | -5.3152245 | 3.29E-07 | 2.45E-06 | 6.06427 |
| CCK | -1.12987 | 4.0517081 | -3.3004942 | 0.001175 | 0.0035037 | -1.6971 |
| NDRG2 | -1.12965 | 9.91658 | -7.3470861 | 7.95E-12 | 2.01E-10 | 16.3934 |
| MMP24 | -1.12964 | 3.0630485 | -7.3112255 | 9.75E-12 | 2.41E-10 | 16.1946 |
| XKR7 | -1.12747 | 1.3469182 | -8.8974997 | 8.00E-16 | 5.71E-14 | 25.4066 |
| PCSK2 | -1.12583 | 3.2503256 | -5.3290126 | 3.08E-07 | 2.32E-06 | 6.12694 |
| DUSP26 | -1.12162 | 4.7640653 | -7.4563093 | 4.27E-12 | 1.17E-10 | 17.002 |
| AGAP2 | -1.11994 | 5.0808366 | -5.4298726 | 1.91E-07 | 1.50E-06 | 6.58885 |
| LPPR1 | -1.11923 | 5.584248 | -5.0313745 | 1.22E-06 | 7.92E-06 | 4.80033 |
| KCNC2 | -1.11775 | 2.1981045 | -4.8770908 | 2.45E-06 | 1.47E-05 | 4.13484 |
| KCNIP3 | -1.11717 | 5.1122169 | -6.626895 | 4.30E-10 | 6.62E-09 | 12.5026 |
| SMIM18 | -1.11559 | 2.6382263 | -6.5117964 | 7.96E-10 | 1.15E-08 | 11.9021 |
| CORO6 | -1.1151 | 2.9815195 | -7.9397281 | 2.57E-13 | 9.68E-12 | 19.7481 |
| NELL1 | -1.11383 | 1.9783889 | -6.2240216 | 3.62E-09 | 4.47E-08 | 10.4287 |
| TUBB4A | -1.11349 | 7.3578553 | -5.3661167 | 2.59E-07 | 1.97E-06 | 6.29615 |
| ARPP21 | -1.113 | 4.5685596 | -6.6077258 | 4.76E-10 | 7.27E-09 | 12.4022 |
| CNTNAP2 | -1.11294 | 3.3691584 | -6.7736909 | 1.94E-10 | 3.32E-09 | 13.2774 |
| LINC00320 | -1.11035 | 3.5861727 | -5.7960581 | 3.20E-08 | 3.08E-07 | 8.31604 |
| LPPR3 | -1.10851 | 3.1831707 | -5.4654339 | 1.61E-07 | 1.29E-06 | 6.75316 |
| RIIAD1 | -1.10458 | 2.6321837 | -6.6120836 | 4.65E-10 | 7.12E-09 | 12.425 |
| SGSM1 | -1.10335 | 2.3216143 | -8.6765589 | 3.10E-15 | 1.91E-13 | 24.0786 |
| RTN1 | -1.10314 | 7.7306231 | -5.8196464 | 2.85E-08 | 2.77E-07 | 8.42991 |
| ZFR2 | -1.10001 | 2.2778386 | -6.2698745 | 2.85E-09 | 3.62E-08 | 10.6607 |
| ATP1A3 | -1.09946 | 6.133991 | -5.2007918 | 5.62E-07 | 3.95E-06 | 5.54867 |
| MAPK8IP2 | -1.09395 | 5.3144442 | -7.8161973 | 5.32E-13 | 1.86E-11 | 19.0386 |
| OLFM1 | -1.09085 | 6.868118 | -5.2869559 | 3.76E-07 | 2.76E-06 | 5.93615 |
| BTBD17 | -1.08765 | 4.4021795 | -5.0219437 | 1.28E-06 | 8.24E-06 | 4.75921 |
| HPCA | -1.08421 | 4.1592233 | -3.6350735 | 0.000367 | 0.0012593 | -0.6173 |
| RAB3A | -1.08395 | 4.9571544 | -4.9857699 | 1.51E-06 | 9.53E-06 | 4.60201 |
| ELFN2 | -1.08014 | 3.649352 | -5.7165869 | 4.75E-08 | 4.40E-07 | 7.93468 |
| TNK2 | -1.07937 | 6.897592 | -7.7995645 | 5.86E-13 | 2.02E-11 | 18.9435 |
| LRP4 | -1.07931 | 5.8931479 | -9.6385525 | 7.87E-18 | 8.74E-16 | 29.9422 |
| HIP1R | -1.07831 | 6.2428243 | -7.3972551 | 5.98E-12 | 1.57E-10 | 16.6724 |
| CYP17A1-AS1 | -1.0755 | 1.8477673 | -8.5465892 | 6.83E-15 | 3.82E-13 | 23.3034 |
| PTPN5 | -1.07459 | 2.8486511 | -4.531508 | 1.10E-05 | 5.61E-05 | 2.70166 |
| CTD-2380F24.1 | -1.07448 | 1.8106084 | -7.42429 | 5.12E-12 | 1.37E-10 | 16.8231 |
| PCDHGC4 | -1.07193 | 4.1224962 | -6.1577998 | 5.11E-09 | 6.02E-08 | 10.0955 |
| CNTFR | -1.07179 | 5.3091615 | -6.6256803 | 4.32E-10 | 6.66E-09 | 12.4963 |
| KSR2 | -1.07077 | 1.3361908 | -11.078473 | 7.66E-22 | 2.30E-19 | 39.019 |
| GFRA1 | -1.06859 | 2.8113024 | -6.2178533 | 3.74E-09 | 4.59E-08 | 10.3975 |
| SLC6A1 | -1.06331 | 6.1137799 | -8.2751625 | 3.51E-14 | 1.63E-12 | 21.6996 |
| GAREML | -1.06096 | 4.6930516 | -7.2960633 | 1.06E-11 | 2.60E-10 | 16.1107 |
| MSX2 | -1.05999 | 1.5834872 | -5.9659682 | 1.36E-08 | 1.44E-07 | 9.1431 |
| STXBP6 | -1.05896 | 3.4463023 | -7.2840573 | 1.14E-11 | 2.76E-10 | 16.0443 |
| GPR98 | -1.05892 | 4.7528733 | -5.6520359 | 6.52E-08 | 5.83E-07 | 7.62754 |
| MYT1 | -1.05722 | 3.1764873 | -5.1920457 | 5.86E-07 | 4.10E-06 | 5.50959 |
| C2orf82 | -1.05557 | 3.59522 | -4.615934 | 7.66E-06 | 4.08E-05 | 3.04428 |
| NGEF | -1.05044 | 4.0994545 | -4.4807698 | 1.36E-05 | 6.79E-05 | 2.49814 |
| SHISA7 | -1.04973 | 3.1034346 | -6.9940687 | 5.77E-11 | 1.15E-09 | 14.4587 |
| FA2H | -1.0494 | 3.9194366 | -4.6398942 | 6.91E-06 | 3.72E-05 | 3.14241 |
| FXYD7 | -1.04871 | 4.5557607 | -3.9376038 | 0.00012 | 0.0004675 | 0.43546 |
| RTN4R | -1.04855 | 3.052352 | -6.1262325 | 6.01E-09 | 6.97E-08 | 9.93741 |
| MRVI1 | -1.04827 | 4.533006 | -7.8028136 | 5.75E-13 | 1.99E-11 | 18.9621 |
| CBLN2 | -1.04583 | 2.2720384 | -4.9713058 | 1.61E-06 | 1.01E-05 | 4.53939 |
| SPHKAP | -1.0457 | 2.3662229 | -5.5369618 | 1.14E-07 | 9.47E-07 | 7.08592 |
| TTC9B | -1.04492 | 4.0293014 | -4.9897397 | 1.48E-06 | 9.38E-06 | 4.61922 |
| CTD-2339F6.1 | -1.04356 | 1.851266 | -3.1627509 | 0.00185 | 0.005222 | -2.1151 |
| C1QTNF4 | -1.04153 | 3.5033352 | -5.5149749 | 1.27E-07 | 1.04E-06 | 6.98332 |
| GUCY1A3 | -1.03729 | 3.5058553 | -7.9471532 | 2.46E-13 | 9.33E-12 | 19.7909 |
| LRRTM4 | -1.03596 | 3.081477 | -7.2829886 | 1.14E-11 | 2.77E-10 | 16.0384 |
| PVALB | -1.03588 | 2.0869312 | -4.0558651 | 7.58E-05 | 0.0003112 | 0.86605 |
| RASGRF1 | -1.03329 | 3.4289868 | -5.1718293 | 6.43E-07 | 4.45E-06 | 5.41945 |
| RIMS1 | -1.03102 | 2.5904637 | -5.8890973 | 2.01E-08 | 2.04E-07 | 8.76697 |
| HTR2A | -1.0291 | 1.8777233 | -7.0622561 | 3.95E-11 | 8.26E-10 | 14.8284 |
| SHC3 | -1.02895 | 3.2399118 | -5.6881029 | 5.46E-08 | 4.99E-07 | 7.79886 |
| CLVS1 | -1.02475 | 2.0348314 | -6.8362955 | 1.38E-10 | 2.47E-09 | 13.6108 |
| MEGF11 | -1.02419 | 4.1246177 | -5.4436193 | 1.79E-07 | 1.42E-06 | 6.65227 |
| CAMK2N2 | -1.02084 | 4.3597651 | -6.8991848 | 9.75E-11 | 1.81E-09 | 13.9475 |
| AC053503.11 | -1.0206 | 1.7521204 | -5.4752629 | 1.54E-07 | 1.24E-06 | 6.79871 |
| NRXN1 | -1.02023 | 5.6708848 | -6.380632 | 1.60E-09 | 2.15E-08 | 11.2255 |
| LGI3 | -1.01991 | 4.1608863 | -4.4602768 | 1.48E-05 | 7.32E-05 | 2.41645 |
| GRIA2 | -1.01985 | 6.1916042 | -6.1135059 | 6.42E-09 | 7.40E-08 | 9.87384 |
| TUB | -1.01858 | 4.8982971 | -8.3007667 | 3.01E-14 | 1.42E-12 | 21.8499 |
| SLC1A6 | -1.01839 | 2.4482529 | -5.9207073 | 1.71E-08 | 1.77E-07 | 8.92125 |
| STX1B | -1.01588 | 4.596512 | -7.1070566 | 3.08E-11 | 6.61E-10 | 15.0724 |
| RP11-1263C18.1 | -1.01528 | 1.7365318 | -6.1877559 | 4.37E-09 | 5.26E-08 | 10.2459 |
| DLGAP3 | -1.01338 | 2.9254474 | -5.578724 | 9.32E-08 | 7.96E-07 | 7.2816 |
| HAR1A | -1.00999 | 1.3320856 | -8.843505 | 1.12E-15 | 7.65E-14 | 25.0809 |
| DGCR5 | -1.00774 | 3.6178284 | -7.3436804 | 8.11E-12 | 2.04E-10 | 16.3745 |
| VIPR2 | -1.00762 | 3.6636171 | -4.2674596 | 3.27E-05 | 0.0001478 | 1.66244 |
| ASPHD1 | -1.00456 | 4.7239366 | -6.607398 | 4.77E-10 | 7.27E-09 | 12.4005 |
| SST | -1.00446 | 3.7309632 | -3.5316195 | 0.000531 | 0.0017408 | -0.9608 |
| CDH22 | -1.00317 | 2.4914921 | -6.0408171 | 9.31E-09 | 1.03E-07 | 9.51238 |
| CASKIN1 | -1.00277 | 4.1944843 | -6.39079 | 1.51E-09 | 2.04E-08 | 11.2776 |
| BSN | -1.00224 | 2.7693213 | -7.332745 | 8.63E-12 | 2.16E-10 | 16.3138 |
| NEURL | -1.00109 | 2.6037064 | -5.0312969 | 1.22E-06 | 7.92E-06 | 4.79999 |
| KIAA1045 | -0.99869 | 2.6022811 | -4.1186379 | 5.93E-05 | 0.0002498 | 1.09887 |
| FRRS1L | -0.98982 | 3.1006549 | -5.7984711 | 3.16E-08 | 3.04E-07 | 8.32768 |
| DLGAP1 | -0.98898 | 4.8712247 | -6.4148237 | 1.33E-09 | 1.81E-08 | 11.4011 |
| FBXL16 | -0.98854 | 5.5972928 | -5.0438615 | 1.16E-06 | 7.52E-06 | 4.85486 |
| CTD-3193O13.9 | -0.98716 | 3.5521624 | -6.9904605 | 5.89E-11 | 1.17E-09 | 14.4392 |
| PID1 | -0.98487 | 5.4515309 | -6.3495807 | 1.88E-09 | 2.49E-08 | 11.0665 |
| PHYHIPL | -0.98331 | 7.2680424 | -6.8330282 | 1.40E-10 | 2.50E-09 | 13.5934 |
| MRO | -0.98053 | 4.0875824 | -6.355901 | 1.82E-09 | 2.42E-08 | 11.0988 |
| TPPP | -0.97961 | 5.1999331 | -4.8050716 | 3.37E-06 | 1.95E-05 | 3.82953 |
| BMP2 | -0.9786 | 4.2286954 | -4.3143719 | 2.70E-05 | 0.0001246 | 1.84344 |
| CHADL | -0.97805 | 4.6037957 | -6.0101549 | 1.09E-08 | 1.18E-07 | 9.36074 |
| CAMKV | -0.97588 | 3.880596 | -3.989617 | 9.80E-05 | 0.0003909 | 0.62354 |
| HLF | -0.97306 | 3.9416163 | -6.4015487 | 1.43E-09 | 1.93E-08 | 11.3328 |
| SRRM4 | -0.97275 | 1.6784009 | -6.6939666 | 2.99E-10 | 4.83E-09 | 12.8554 |
| FAIM2 | -0.96936 | 6.9188982 | -7.1404637 | 2.55E-11 | 5.62E-10 | 15.2548 |
| AMER2 | -0.96744 | 5.251438 | -6.6535597 | 3.72E-10 | 5.83E-09 | 12.6426 |
| RASSF2 | -0.96668 | 6.74098 | -6.8740684 | 1.12E-10 | 2.05E-09 | 13.8128 |
| CSPG5 | -0.96647 | 7.0050134 | -4.8493241 | 2.77E-06 | 1.64E-05 | 4.01672 |
| KIF21B | -0.96613 | 4.4308496 | -6.5870373 | 5.32E-10 | 8.03E-09 | 12.294 |
| DGKG | -0.96455 | 3.9088012 | -6.6376653 | 4.05E-10 | 6.27E-09 | 12.5592 |
| GABRA5 | -0.96258 | 2.4506756 | -3.7831935 | 0.000214 | 0.0007803 | -0.1107 |
| WASF3 | -0.96257 | 5.6632273 | -8.7565066 | 1.90E-15 | 1.21E-13 | 24.5577 |
| MN1 | -0.96231 | 2.825975 | -7.5553178 | 2.42E-12 | 7.15E-11 | 17.5576 |
| GRM5 | -0.95863 | 1.9304919 | -5.6254843 | 7.42E-08 | 6.53E-07 | 7.50189 |
| RIT2 | -0.95716 | 2.7902352 | -4.351701 | 2.32E-05 | 0.000109 | 1.98859 |
| PCBP3 | -0.95693 | 3.3451483 | -7.4683315 | 3.98E-12 | 1.10E-10 | 17.0693 |
| SH3GL3 | -0.95628 | 3.9701869 | -4.7721975 | 3.89E-06 | 2.22E-05 | 3.69131 |
| NPY | -0.95562 | 4.5843622 | -3.3082754 | 0.001145 | 0.0034253 | -1.6731 |
| UBE2QL1 | -0.95552 | 2.9150685 | -5.5845154 | 9.07E-08 | 7.78E-07 | 7.30881 |
| 3-Sep | -0.95388 | 6.7613691 | -7.5233528 | 2.90E-12 | 8.38E-11 | 17.3779 |
| FAM19A2 | -0.95348 | 2.64432 | -5.7174791 | 4.72E-08 | 4.38E-07 | 7.93894 |
| GPR123 | -0.95268 | 3.8228829 | -6.8180915 | 1.52E-10 | 2.69E-09 | 13.5137 |
| FBLL1 | -0.95202 | 3.7970717 | -6.3513174 | 1.86E-09 | 2.48E-08 | 11.0754 |
| RP11-116O18.1 | -0.94917 | 1.138646 | -5.8127915 | 2.94E-08 | 2.86E-07 | 8.39679 |
| MCF2 | -0.94911 | 2.0373849 | -7.29065 | 1.10E-11 | 2.67E-10 | 16.0807 |
| RP11-1C8.7 | -0.94767 | 1.3379338 | -6.9644751 | 6.80E-11 | 1.33E-09 | 14.2988 |
| GNG3 | -0.94712 | 4.4521315 | -2.8956077 | 0.004279 | 0.0108288 | -2.8804 |
| EMX2 | -0.94665 | 2.7989951 | -6.0509604 | 8.84E-09 | 9.82E-08 | 9.56266 |
| RBFOX1 | -0.94632 | 2.9176984 | -3.6922272 | 0.000299 | 0.0010485 | -0.4239 |
| MPPED1 | -0.94321 | 2.2988806 | -4.0885708 | 6.67E-05 | 0.0002778 | 0.98699 |
| ATP2B2 | -0.94015 | 4.0605508 | -5.6804059 | 5.67E-08 | 5.16E-07 | 7.76223 |
| RP11-74E22.4 | -0.93789 | 1.4954283 | -8.1484649 | 7.48E-14 | 3.29E-12 | 20.9583 |
| SYT7 | -0.93678 | 3.2212978 | -3.8950397 | 0.000141 | 0.0005394 | 0.28308 |
| SYP | -0.93623 | 6.3558222 | -5.324388 | 3.15E-07 | 2.36E-06 | 6.10591 |
| CTD-2396E7.9 | -0.93599 | 3.4303176 | -4.6005259 | 8.18E-06 | 4.33E-05 | 2.98139 |
| OGDHL | -0.93554 | 2.6720178 | -4.9753732 | 1.58E-06 | 9.92E-06 | 4.55698 |
| UNC80 | -0.93536 | 3.0231613 | -7.9423506 | 2.54E-13 | 9.56E-12 | 19.7632 |
| KCNJ9 | -0.93533 | 4.3840896 | -5.9162764 | 1.75E-08 | 1.80E-07 | 8.89959 |
| GABRB2 | -0.93517 | 2.2301707 | -3.8608984 | 0.00016 | 0.0006044 | 0.16185 |
| RP11-227B21.2 | -0.93434 | 1.633883 | -5.4682386 | 1.59E-07 | 1.27E-06 | 6.76615 |
| VSTM2L | -0.93401 | 3.5515437 | -4.095402 | 6.49E-05 | 0.0002711 | 1.01235 |
| CTB-1I21.1 | -0.93289 | 1.3500105 | -5.5338155 | 1.16E-07 | 9.60E-07 | 7.07122 |
| ATP9A | -0.93226 | 6.1338525 | -8.9520865 | 5.72E-16 | 4.25E-14 | 25.7365 |
| FAM19A5 | -0.93213 | 5.7162934 | -8.0650205 | 1.23E-13 | 5.10E-12 | 20.4729 |
| KCNC1 | -0.93042 | 2.9542833 | -6.1734934 | 4.71E-09 | 5.61E-08 | 10.1742 |
| CADM2 | -0.93001 | 5.5213971 | -6.8500658 | 1.28E-10 | 2.30E-09 | 13.6844 |
| CDR1 | -0.92824 | 5.3178894 | -4.4883912 | 1.31E-05 | 6.61E-05 | 2.5286 |
| CACNA2D3 | -0.92569 | 2.0870422 | -6.2029272 | 4.04E-09 | 4.92E-08 | 10.3223 |
| FGF13 | -0.92544 | 3.6200159 | -4.9564458 | 1.72E-06 | 1.07E-05 | 4.4752 |
| LINC00966 | -0.92467 | 2.239189 | -5.8638137 | 2.28E-08 | 2.28E-07 | 8.64396 |
| LINC00634 | -0.92415 | 4.4638915 | -6.7123991 | 2.71E-10 | 4.42E-09 | 12.9527 |
| PSD2 | -0.92396 | 5.5137652 | -5.8722037 | 2.19E-08 | 2.20E-07 | 8.68474 |
| RAB11FIP4 | -0.92347 | 4.3031078 | -6.0729684 | 7.90E-09 | 8.89E-08 | 9.67192 |
| PTCHD2 | -0.92183 | 3.567925 | -5.3170477 | 3.26E-07 | 2.43E-06 | 6.07255 |
| RP11-1055B8.3 | -0.92078 | 3.0603361 | -5.2266967 | 4.98E-07 | 3.55E-06 | 5.66468 |
| TLX1 | -0.9203 | 1.3720722 | -5.3564528 | 2.71E-07 | 2.06E-06 | 6.252 |
| SLITRK1 | -0.9201 | 2.8282639 | -5.7495006 | 4.03E-08 | 3.79E-07 | 8.09219 |
| VAT1L | -0.91921 | 4.6352276 | -4.6013705 | 8.15E-06 | 4.32E-05 | 2.98483 |
| MCF2L2 | -0.91829 | 3.3165248 | -7.0857373 | 3.47E-11 | 7.36E-10 | 14.9562 |
| GNG4 | -0.91712 | 4.7437967 | -5.5474985 | 1.08E-07 | 9.08E-07 | 7.1352 |
| CELSR3 | -0.91706 | 3.5608017 | -6.7469797 | 2.24E-10 | 3.76E-09 | 13.1357 |
| LHX5 | -0.91541 | 1.0766009 | -6.0366157 | 9.51E-09 | 1.05E-07 | 9.49158 |
| ARHGAP44 | -0.91189 | 2.78785 | -6.1410351 | 5.57E-09 | 6.52E-08 | 10.0115 |
| ASIC1 | -0.91126 | 5.5026783 | -6.2322064 | 3.47E-09 | 4.31E-08 | 10.47 |
| KCNT1 | -0.91088 | 1.8962479 | -5.0182854 | 1.30E-06 | 8.36E-06 | 4.74327 |
| ATP8A1 | -0.90729 | 4.5569418 | -6.1564762 | 5.14E-09 | 6.06E-08 | 10.0888 |
| AC073479.1 | -0.90725 | 3.1173232 | -6.338505 | 1.99E-09 | 2.63E-08 | 11.0099 |
| CTD-3199J23.4 | -0.90655 | 3.7508239 | -5.1708142 | 6.46E-07 | 4.47E-06 | 5.41493 |
| CLEC2L | -0.90573 | 2.0536146 | -4.23509 | 3.73E-05 | 0.0001657 | 1.53848 |
| FXYD6 | -0.90556 | 9.1746955 | -5.6480378 | 6.65E-08 | 5.94E-07 | 7.60859 |
| TEF | -0.90496 | 4.454099 | -8.3111801 | 2.83E-14 | 1.35E-12 | 21.9112 |
| MYH7 | -0.9038 | 2.7305801 | -5.7539818 | 3.94E-08 | 3.72E-07 | 8.11369 |
| BEX2 | -0.90233 | 6.1071668 | -5.632557 | 7.17E-08 | 6.33E-07 | 7.53532 |
| NAP1L2 | -0.90133 | 4.1332927 | -5.5310821 | 1.17E-07 | 9.72E-07 | 7.05846 |
| BRSK2 | -0.90129 | 4.898284 | -6.8600603 | 1.21E-10 | 2.20E-09 | 13.7378 |
| HECW1 | -0.90105 | 1.9043734 | -6.4599892 | 1.05E-09 | 1.47E-08 | 11.6339 |
| FBXO41 | -0.89991 | 3.5369378 | -6.8248323 | 1.47E-10 | 2.61E-09 | 13.5497 |
| KCNN1 | -0.8991 | 3.1577844 | -5.5894108 | 8.85E-08 | 7.63E-07 | 7.33183 |
| UNC13C | -0.89902 | 1.6979637 | -4.7741788 | 3.86E-06 | 2.20E-05 | 3.69962 |
| CEND1 | -0.89836 | 5.5562851 | -6.0363534 | 9.53E-09 | 1.05E-07 | 9.49028 |
| RP11-588K22.2 | -0.89825 | 2.7249069 | -6.8934766 | 1.01E-10 | 1.86E-09 | 13.9169 |
| SCD5 | -0.89623 | 8.7259095 | -7.5737538 | 2.17E-12 | 6.49E-11 | 17.6615 |
| CPE | -0.89567 | 9.667947 | -6.096821 | 6.99E-09 | 7.99E-08 | 9.79063 |
| TAGLN3 | -0.8935 | 5.9066669 | -4.4846686 | 1.34E-05 | 6.70E-05 | 2.51372 |
| RCOR2 | -0.89266 | 4.1907247 | -4.656283 | 6.44E-06 | 3.48E-05 | 3.20976 |
| GRM3 | -0.89122 | 3.9364384 | -4.4681246 | 1.43E-05 | 7.13E-05 | 2.4477 |
| RP11-686F15.3 | -0.89038 | 0.9162865 | -9.0520007 | 3.08E-16 | 2.40E-14 | 26.3423 |
| NT5C1A | -0.88969 | 1.4784368 | -8.0722364 | 1.18E-13 | 4.92E-12 | 20.5148 |
| DACH2 | -0.88927 | 1.8118828 | -5.0915707 | 9.30E-07 | 6.18E-06 | 5.06414 |
| RAPGEF4 | -0.88915 | 5.1858852 | -5.1244836 | 8.00E-07 | 5.40E-06 | 5.20936 |
| KIF1A | -0.88893 | 6.8727808 | -6.8344033 | 1.39E-10 | 2.49E-09 | 13.6007 |
| RND2 | -0.88861 | 7.4495423 | -5.9283267 | 1.65E-08 | 1.71E-07 | 8.95852 |
| VWC2L | -0.88857 | 1.3065712 | -6.4695912 | 9.97E-10 | 1.40E-08 | 11.6835 |
| SLITRK5 | -0.88848 | 3.0052966 | -7.2053387 | 1.77E-11 | 4.09E-10 | 15.6105 |
| MIR7-3HG | -0.88829 | 2.1098714 | -4.6407447 | 6.88E-06 | 3.71E-05 | 3.1459 |
| PRMT8 | -0.88802 | 1.7916315 | -5.1818231 | 6.14E-07 | 4.27E-06 | 5.46398 |
| GRIN2C | -0.88751 | 2.9472782 | -6.1128676 | 6.44E-09 | 7.42E-08 | 9.87066 |
| SCD | -0.88663 | 8.2585988 | -5.8175752 | 2.87E-08 | 2.80E-07 | 8.4199 |
| NPTXR | -0.88564 | 5.0263706 | -5.235501 | 4.78E-07 | 3.42E-06 | 5.7042 |
| CDH18 | -0.88544 | 2.5745236 | -5.0885782 | 9.43E-07 | 6.26E-06 | 5.05097 |
| SYN3 | -0.88486 | 2.6365864 | -6.3872053 | 1.54E-09 | 2.08E-08 | 11.2592 |
| DSCAML1 | -0.88425 | 3.4867033 | -5.6645364 | 6.13E-08 | 5.53E-07 | 7.68683 |
| RIMS4 | -0.88395 | 3.4783315 | -5.3821905 | 2.40E-07 | 1.84E-06 | 6.36971 |
| CHRDL1 | -0.88367 | 3.9647712 | -4.6788446 | 5.84E-06 | 3.19E-05 | 3.30278 |
| GPRIN1 | -0.88139 | 3.7067378 | -6.5808675 | 5.50E-10 | 8.25E-09 | 12.2617 |
| SPOCK1 | -0.88112 | 5.8320548 | -5.4597126 | 1.66E-07 | 1.32E-06 | 6.72667 |
| SYNPR | -0.88059 | 2.6928255 | -3.1885987 | 0.001701 | 0.0048564 | -2.0379 |
| LMO3 | -0.88035 | 5.430724 | -4.7580307 | 4.14E-06 | 2.35E-05 | 3.63197 |
| SYT5 | -0.87901 | 3.6110465 | -3.6917557 | 0.000299 | 0.0010497 | -0.4255 |
| SLAIN1 | -0.87876 | 7.1025826 | -6.9140573 | 8.98E-11 | 1.69E-09 | 14.0274 |
| SYNGR3 | -0.87838 | 3.3067057 | -3.8370541 | 0.000175 | 0.0006545 | 0.07772 |
| LHFPL3 | -0.87837 | 5.8920099 | -3.5130563 | 0.000567 | 0.0018429 | -1.0215 |
| FAM196B | -0.87835 | 1.9184254 | -7.1534193 | 2.37E-11 | 5.30E-10 | 15.3257 |
| CACNG4 | -0.87775 | 5.4710899 | -4.2272777 | 3.84E-05 | 0.0001705 | 1.50868 |
| RLTPR | -0.87601 | 2.3888128 | -5.3534319 | 2.75E-07 | 2.09E-06 | 6.23821 |
| CLVS2 | -0.87556 | 2.0787193 | -5.237934 | 4.73E-07 | 3.39E-06 | 5.71513 |
| TMEM100 | -0.875 | 5.4508604 | -3.3378982 | 0.001036 | 0.0031346 | -1.5809 |
| DOC2A | -0.87412 | 3.2869541 | -3.9592088 | 0.00011 | 0.000434 | 0.51334 |
| MAST1 | -0.87384 | 4.5076487 | -6.0496617 | 8.90E-09 | 9.88E-08 | 9.55622 |
| HSD17B6 | -0.87285 | 3.4915716 | -5.8238902 | 2.79E-08 | 2.72E-07 | 8.45043 |
| CRYM | -0.87252 | 3.1615021 | -2.7923649 | 0.005829 | 0.0141736 | -3.1598 |
| FAM222A | -0.87157 | 4.7000147 | -5.397136 | 2.23E-07 | 1.73E-06 | 6.43825 |
| NPTX1 | -0.87139 | 3.3485799 | -3.208992 | 0.001591 | 0.0045838 | -1.9766 |
| UGT8 | -0.87052 | 5.1708527 | -4.1029709 | 6.30E-05 | 0.000264 | 1.04048 |
| CDK5R1 | -0.86963 | 4.7998353 | -5.5423107 | 1.11E-07 | 9.27E-07 | 7.11093 |
| OPALIN | -0.86962 | 3.0727683 | -2.676927 | 0.008154 | 0.0190413 | -3.4612 |
| CREG2 | -0.86814 | 2.524261 | -3.323569 | 0.001087 | 0.0032731 | -1.6256 |
| LIMS2 | -0.86809 | 4.4264304 | -5.8804766 | 2.10E-08 | 2.12E-07 | 8.72499 |
| KCNB1 | -0.86782 | 3.3310866 | -6.3954256 | 1.48E-09 | 2.00E-08 | 11.3014 |
| DCX | -0.86621 | 3.4875442 | -4.0604223 | 7.45E-05 | 0.0003063 | 0.88285 |
| EYA1 | -0.86594 | 3.1924029 | -4.3122972 | 2.72E-05 | 0.0001256 | 1.8354 |
| RIPPLY2 | -0.86571 | 3.5889662 | -5.9619213 | 1.39E-08 | 1.47E-07 | 9.12322 |
| MIAT | -0.86516 | 4.134002 | -4.978678 | 1.55E-06 | 9.79E-06 | 4.57129 |
| PAK3 | -0.8643 | 2.8189373 | -5.8279471 | 2.73E-08 | 2.68E-07 | 8.47006 |
| ANK3 | -0.86423 | 3.9110871 | -5.7153655 | 4.77E-08 | 4.42E-07 | 7.92884 |
| GRIK2 | -0.86216 | 4.1063308 | -5.0590862 | 1.08E-06 | 7.06E-06 | 4.92149 |
| SLC1A1 | -0.85924 | 3.6926108 | -5.9209529 | 1.71E-08 | 1.77E-07 | 8.92245 |
| SCN2B | -0.85896 | 2.8524332 | -4.975599 | 1.58E-06 | 9.92E-06 | 4.55796 |
| RP11-357K6.1 | -0.85888 | 1.6743677 | -5.7397791 | 4.23E-08 | 3.97E-07 | 8.04561 |
| CASQ1 | -0.85548 | 3.765485 | -5.865209 | 2.27E-08 | 2.27E-07 | 8.65074 |
| SYCE1 | -0.85544 | 1.3373561 | -4.9628564 | 1.67E-06 | 1.04E-05 | 4.50287 |
| TMEM63C | -0.85518 | 2.9216258 | -7.1467339 | 2.46E-11 | 5.45E-10 | 15.2891 |
| SLC22A6 | -0.85389 | 1.5604305 | -6.2674185 | 2.89E-09 | 3.66E-08 | 10.6482 |
| CCDC64 | -0.85388 | 2.5822013 | -5.0577425 | 1.09E-06 | 7.11E-06 | 4.9156 |
| HS3ST2 | -0.85387 | 2.3022296 | -4.4165435 | 1.77E-05 | 8.58E-05 | 2.24311 |
| BAI3 | -0.85353 | 4.6651259 | -7.1949787 | 1.88E-11 | 4.30E-10 | 15.5536 |
| OMG | -0.85202 | 5.9221656 | -5.2328637 | 4.84E-07 | 3.46E-06 | 5.69236 |
| MGAT4C | -0.85048 | 2.815441 | -4.6950516 | 5.45E-06 | 3.00E-05 | 3.36981 |
| CTD-2396E7.10 | -0.84934 | 3.6850892 | -4.3869581 | 2.01E-05 | 9.59E-05 | 2.12661 |
| ATP8A2 | -0.84915 | 1.7348427 | -5.2922122 | 3.67E-07 | 2.70E-06 | 5.95994 |
| BEX1 | -0.84891 | 7.9803036 | -5.4028758 | 2.17E-07 | 1.69E-06 | 6.46461 |
| ABAT | -0.84852 | 7.2578517 | -7.3402679 | 8.27E-12 | 2.08E-10 | 16.3556 |
| AATK | -0.84842 | 5.166714 | -4.6064474 | 7.97E-06 | 4.23E-05 | 3.00554 |
| NEU4 | -0.84801 | 4.5296818 | -3.622191 | 0.000385 | 0.001311 | -0.6605 |
| FHDC1 | -0.84635 | 2.1748201 | -5.7619128 | 3.79E-08 | 3.59E-07 | 8.15175 |
| SDS | -0.84611 | 3.3209333 | -4.8774931 | 2.44E-06 | 1.47E-05 | 4.13655 |
| STXBP1 | -0.84488 | 5.9515534 | -5.502507 | 1.35E-07 | 1.10E-06 | 6.92526 |
| SLC4A10 | -0.84448 | 2.7933607 | -4.096534 | 6.46E-05 | 0.0002702 | 1.01655 |
| SV2B | -0.84357 | 2.5122566 | -3.3507035 | 0.000992 | 0.0030207 | -1.5409 |
| HCN2 | -0.84345 | 4.599066 | -5.8400123 | 2.57E-08 | 2.54E-07 | 8.52848 |
| ATP2B3 | -0.84339 | 1.8684569 | -4.7816312 | 3.73E-06 | 2.14E-05 | 3.7309 |
| CRY2 | -0.84275 | 4.9887142 | -8.5971287 | 5.03E-15 | 2.94E-13 | 23.6043 |
| YPEL4 | -0.84238 | 3.8159351 | -6.5829952 | 5.44E-10 | 8.19E-09 | 12.2729 |
| SRCIN1 | -0.84098 | 4.1990017 | -5.0023851 | 1.40E-06 | 8.92E-06 | 4.67411 |
| KIAA1161 | -0.84068 | 4.1851797 | -6.8127351 | 1.57E-10 | 2.76E-09 | 13.4852 |
| KIAA1244 | -0.83994 | 3.1943228 | -7.2283174 | 1.56E-11 | 3.64E-10 | 15.7368 |
| PHACTR1 | -0.83855 | 4.5697189 | -6.9838325 | 6.11E-11 | 1.20E-09 | 14.4033 |
| HDAC4 | -0.83803 | 3.954402 | -7.869773 | 3.88E-13 | 1.41E-11 | 19.3457 |
| GRIA4 | -0.83762 | 5.6133389 | -4.7472696 | 4.34E-06 | 2.45E-05 | 3.58699 |
| CBLN1 | -0.83707 | 2.538442 | -5.378476 | 2.44E-07 | 1.87E-06 | 6.3527 |
| WDR86 | -0.83699 | 3.1973299 | -6.7558798 | 2.14E-10 | 3.60E-09 | 13.1829 |
| CA4 | -0.83543 | 3.3640865 | -4.8703663 | 2.52E-06 | 1.51E-05 | 4.10618 |
| EEF1A2 | -0.83468 | 5.8498726 | -3.4328478 | 0.000749 | 0.0023483 | -1.2808 |
| FERMT1 | -0.83444 | 3.8783657 | -3.2387174 | 0.001443 | 0.0042066 | -1.8866 |
| CAMK2B | -0.83415 | 4.5045308 | -3.713471 | 0.000276 | 0.0009796 | -0.3513 |
| RGS4 | -0.83276 | 3.8954629 | -2.8271926 | 0.005256 | 0.0129791 | -3.0665 |
| RP11-731J8.2 | -0.83242 | 2.859043 | -5.1516504 | 7.06E-07 | 4.84E-06 | 5.32974 |
| FGF14 | -0.83041 | 2.9122968 | -5.6005141 | 8.39E-08 | 7.27E-07 | 7.3841 |
| SLC29A2 | -0.83033 | 2.6762387 | -6.2358636 | 3.41E-09 | 4.24E-08 | 10.4885 |
| PPP1R16B | -0.82947 | 3.6741384 | -4.3813929 | 2.05E-05 | 9.79E-05 | 2.10477 |
| LINC00925 | -0.82905 | 5.1661019 | -4.073617 | 7.07E-05 | 0.0002923 | 0.93159 |
| VWA5B2 | -0.82748 | 2.7021728 | -5.6825575 | 5.61E-08 | 5.11E-07 | 7.77247 |
| LINC00403 | -0.82728 | 2.6217921 | -5.4715848 | 1.56E-07 | 1.26E-06 | 6.78166 |
| PRKCG | -0.82672 | 2.1454609 | -3.4548462 | 0.000694 | 0.0022013 | -1.2102 |
| PDXP | -0.82658 | 5.3007296 | -5.1148501 | 8.36E-07 | 5.61E-06 | 5.16678 |
| LINC00617 | -0.82657 | 1.5002357 | -5.1120909 | 8.47E-07 | 5.67E-06 | 5.1546 |
| TMEM151A | -0.82567 | 3.9868546 | -3.979519 | 0.000102 | 0.0004051 | 0.58687 |
| CACNG3 | -0.82547 | 1.8258264 | -3.522522 | 0.000548 | 0.0017893 | -0.9906 |
| PPFIA2 | -0.82497 | 3.7764379 | -5.558314 | 1.03E-07 | 8.70E-07 | 7.18584 |
| FAM159B | -0.82409 | 0.8409508 | -5.8087474 | 3.00E-08 | 2.91E-07 | 8.37726 |
| RAP1GAP | -0.82372 | 4.8747671 | -5.6251804 | 7.44E-08 | 6.53E-07 | 7.50045 |
| ATP1A2 | -0.82358 | 7.6584589 | -4.039344 | 8.08E-05 | 0.0003302 | 0.80527 |
| C2orf80 | -0.82138 | 3.8686737 | -4.2891412 | 2.99E-05 | 0.0001365 | 1.74589 |
| PCP4L1 | -0.82083 | 2.4458275 | -3.4322947 | 0.000751 | 0.0023523 | -1.2826 |
| DOK6 | -0.82079 | 2.6063647 | -5.983599 | 1.25E-08 | 1.33E-07 | 9.22982 |
| CACNG8 | -0.81951 | 2.3873664 | -5.4128354 | 2.07E-07 | 1.62E-06 | 6.51039 |
| PRKCB | -0.81895 | 3.9750064 | -4.269011 | 3.25E-05 | 0.000147 | 1.6684 |
| GRIA1 | -0.81893 | 5.4016232 | -4.2723706 | 3.20E-05 | 0.0001453 | 1.68131 |
| ABTB2 | -0.81836 | 2.8855097 | -6.6689448 | 3.42E-10 | 5.42E-09 | 12.7236 |
| CAMK1G | -0.81811 | 1.9931192 | -3.7054081 | 0.000285 | 0.0010051 | -0.3789 |
| TMEM155 | -0.8174 | 1.8903071 | -3.5676572 | 0.000468 | 0.001558 | -0.8421 |
| PEX5L | -0.81511 | 3.3325562 | -4.0632788 | 7.36E-05 | 0.0003032 | 0.89339 |
| HPSE2 | -0.8134 | 2.0159727 | -4.1109244 | 6.11E-05 | 0.0002567 | 1.0701 |
| TPTE2P1 | -0.81254 | 1.8787252 | -6.9690981 | 6.63E-11 | 1.30E-09 | 14.3238 |
| GRAMD1B | -0.81252 | 4.002135 | -8.017527 | 1.63E-13 | 6.47E-12 | 20.1975 |
| ALDH5A1 | -0.81201 | 5.1882785 | -7.9942908 | 1.87E-13 | 7.30E-12 | 20.0631 |
| CHD5 | -0.81179 | 2.4731989 | -3.6714523 | 0.000322 | 0.0011204 | -0.4945 |
| SEMA4A | -0.81057 | 3.191717 | -7.0868436 | 3.45E-11 | 7.33E-10 | 14.9622 |
| CNRIP1 | -0.80983 | 5.9955081 | -7.270716 | 1.23E-11 | 2.94E-10 | 15.9706 |
| ANO5 | -0.8092 | 2.0676858 | -7.0402499 | 4.47E-11 | 9.22E-10 | 14.7088 |
| NCR3LG1 | -0.80912 | 1.7886123 | -6.8884115 | 1.03E-10 | 1.90E-09 | 13.8897 |
| RP11-192H23.5 | -0.80886 | 3.0636144 | -3.1581631 | 0.001877 | 0.0052898 | -2.1288 |
| RP11-423G4.7 | -0.80833 | 1.1392688 | -6.4185458 | 1.31E-09 | 1.78E-08 | 11.4202 |
| FLRT1 | -0.80704 | 3.409111 | -5.8495405 | 2.45E-08 | 2.43E-07 | 8.57467 |
| LRRTM2 | -0.80648 | 4.0890681 | -6.1631664 | 4.97E-09 | 5.87E-08 | 10.1224 |
| JAKMIP1 | -0.8063 | 2.9708028 | -4.217039 | 4.01E-05 | 0.0001767 | 1.46968 |
| MARVELD3 | -0.80623 | 2.193256 | -4.2002955 | 4.28E-05 | 0.0001875 | 1.40609 |
| MAL2 | -0.80479 | 1.9377342 | -3.3667303 | 0.00094 | 0.0028795 | -1.4906 |
| HR | -0.80474 | 3.6839288 | -5.6352751 | 7.08E-08 | 6.26E-07 | 7.54817 |
| GAL3ST1 | -0.80435 | 3.6456212 | -5.4763544 | 1.53E-07 | 1.23E-06 | 6.80377 |
| ALDH2 | -0.80371 | 7.5794089 | -6.6430217 | 3.94E-10 | 6.12E-09 | 12.5873 |
| AC005740.3 | -0.80287 | 0.9030556 | -6.6517645 | 3.76E-10 | 5.88E-09 | 12.6332 |
| FAM110B | -0.80262 | 5.3684597 | -5.097295 | 9.06E-07 | 6.03E-06 | 5.08935 |
| TCEAL5 | -0.80171 | 5.1386775 | -5.3723738 | 2.51E-07 | 1.92E-06 | 6.32477 |
| PKIA | -0.80136 | 5.386139 | -6.0531601 | 8.74E-09 | 9.74E-08 | 9.57357 |
| VSTM2B | -0.80124 | 3.8222375 | -4.6243431 | 7.39E-06 | 3.95E-05 | 3.07868 |
| KCNAB1 | -0.80065 | 2.877624 | -5.7816117 | 3.44E-08 | 3.29E-07 | 8.24645 |
| PCDHGC3 | -0.79697 | 7.8982726 | -5.6960196 | 5.25E-08 | 4.82E-07 | 7.83656 |
| CHRM4 | -0.79624 | 1.9097099 | -6.0773853 | 7.72E-09 | 8.72E-08 | 9.69388 |
| OPCML | -0.79617 | 3.4394729 | -4.3263454 | 2.57E-05 | 0.0001193 | 1.88989 |
| PDZD2 | -0.79473 | 3.7376449 | -6.4224797 | 1.28E-09 | 1.75E-08 | 11.4405 |
| RTN4RL2 | -0.79467 | 3.5299092 | -4.9600157 | 1.69E-06 | 1.06E-05 | 4.49061 |
| RGMB | -0.79369 | 5.2160717 | -5.2880243 | 3.74E-07 | 2.75E-06 | 5.94098 |
| PCDH7 | -0.79364 | 3.8327447 | -5.0099656 | 1.35E-06 | 8.65E-06 | 4.70706 |
| HMGA1P7 | -0.79336 | 1.1324288 | -6.0048784 | 1.12E-08 | 1.21E-07 | 9.3347 |
| AP3B2 | -0.79285 | 4.6937255 | -6.077135 | 7.73E-09 | 8.73E-08 | 9.69264 |
| AK5 | -0.79212 | 4.1838288 | -3.0610205 | 0.002562 | 0.0069214 | -2.4137 |
| DGCR9 | -0.79169 | 2.0798667 | -6.4219331 | 1.28E-09 | 1.75E-08 | 11.4376 |
| KIAA1755 | -0.79157 | 4.4266306 | -6.7517783 | 2.18E-10 | 3.67E-09 | 13.1612 |
| PLP1 | -0.79089 | 9.9001901 | -3.1909367 | 0.001688 | 0.0048244 | -2.0309 |
| CNNM1 | -0.79063 | 1.5993946 | -5.4907788 | 1.43E-07 | 1.16E-06 | 6.87073 |
| TDRD9 | -0.79013 | 1.7240392 | -5.5525012 | 1.06E-07 | 8.90E-07 | 7.15861 |
| CMTM5 | -0.78923 | 5.9070936 | -4.9345956 | 1.89E-06 | 1.17E-05 | 4.38107 |
| REPS2 | -0.78664 | 3.4440088 | -5.6053378 | 8.19E-08 | 7.12E-07 | 7.40682 |
| SNX22 | -0.78644 | 4.7849971 | -3.5597802 | 0.000481 | 0.0015947 | -0.8681 |
| AC068057.1 | -0.78585 | 1.9934992 | -4.1739315 | 4.76E-05 | 0.0002058 | 1.30636 |
| C8orf46 | -0.78579 | 5.9770729 | -7.5278856 | 2.83E-12 | 8.20E-11 | 17.4033 |
| SLC8A3 | -0.78577 | 3.1508166 | -5.2420738 | 4.64E-07 | 3.33E-06 | 5.73374 |
| GPR158 | -0.78445 | 4.101213 | -6.0524898 | 8.77E-09 | 9.76E-08 | 9.57024 |
| AF131216.5 | -0.78392 | 2.8645634 | -4.4643488 | 1.45E-05 | 7.22E-05 | 2.43266 |
| SCN2A | -0.78377 | 3.1010597 | -4.9669193 | 1.64E-06 | 1.03E-05 | 4.52043 |
| RASGEF1C | -0.78336 | 3.3293168 | -6.2852778 | 2.63E-09 | 3.37E-08 | 10.7389 |
| KIF6 | -0.7818 | 2.6557433 | -4.1524351 | 5.18E-05 | 0.0002223 | 1.22542 |
| ADCY5 | -0.78122 | 4.0389013 | -7.3450421 | 8.05E-12 | 2.03E-10 | 16.3821 |
| SLCO1A2 | -0.78117 | 3.8407875 | -3.8835161 | 0.000147 | 0.0005606 | 0.24206 |
| LINC00844 | -0.78074 | 6.7082191 | -3.7185226 | 0.000271 | 0.0009638 | -0.334 |
| SCAMP5 | -0.78053 | 6.512931 | -6.9973197 | 5.67E-11 | 1.13E-09 | 14.4762 |
| SMPD3 | -0.78045 | 2.9256859 | -6.3371953 | 2.01E-09 | 2.65E-08 | 11.0033 |
| CD300LG | -0.78018 | 0.8747616 | -6.0857446 | 7.40E-09 | 8.40E-08 | 9.73547 |
| CTD-2545M3.8 | -0.78007 | 1.7989727 | -5.2956916 | 3.61E-07 | 2.66E-06 | 5.97569 |
| RP11-285F16.1 | -0.77896 | 1.9319537 | -5.4029174 | 2.17E-07 | 1.69E-06 | 6.4648 |
| CDH20 | -0.77826 | 4.6462005 | -5.2183351 | 5.18E-07 | 3.67E-06 | 5.62719 |
| SCN8A | -0.77797 | 2.6271582 | -6.3104828 | 2.31E-09 | 3.00E-08 | 10.8671 |
| SLC30A3 | -0.77559 | 1.899984 | -3.3060902 | 0.001153 | 0.0034488 | -1.6798 |
| BZRAP1 | -0.7754 | 5.2785667 | -5.5915687 | 8.76E-08 | 7.56E-07 | 7.34198 |
| SPRN | -0.77525 | 2.8610576 | -7.0407537 | 4.45E-11 | 9.21E-10 | 14.7116 |
| OLIG1 | -0.77511 | 8.3135146 | -3.5713589 | 0.000461 | 0.001539 | -0.8299 |
| SLC17A8 | -0.77502 | 1.3144223 | -4.6016413 | 8.14E-06 | 4.31E-05 | 2.98593 |
| SNCG | -0.77429 | 5.0560284 | -3.0005133 | 0.003099 | 0.0081561 | -2.5871 |
| NALCN | -0.77332 | 3.6848136 | -6.2730495 | 2.81E-09 | 3.57E-08 | 10.6768 |
| WBSCR17 | -0.77164 | 2.9506395 | -3.3095188 | 0.00114 | 0.0034135 | -1.6692 |
| VEPH1 | -0.77082 | 2.787763 | -3.4365515 | 0.00074 | 0.0023239 | -1.2689 |
| ADRA1A | -0.76957 | 2.4574804 | -5.4351076 | 1.86E-07 | 1.47E-06 | 6.61299 |
| KCNN2 | -0.7687 | 4.1036676 | -4.8370654 | 2.93E-06 | 1.72E-05 | 3.96473 |
| CHST1 | -0.76835 | 4.8115369 | -4.9793362 | 1.55E-06 | 9.77E-06 | 4.57414 |
| RBP4 | -0.76821 | 2.1337483 | -3.5317232 | 0.000531 | 0.0017405 | -0.9605 |
| TMEM196 | -0.76818 | 1.6269422 | -5.0830855 | 9.67E-07 | 6.39E-06 | 5.02682 |
| SCG5 | -0.76711 | 6.0160338 | -3.72511 | 0.000265 | 0.0009446 | -0.3114 |
| SLC24A2 | -0.7669 | 3.0743314 | -3.9762391 | 0.000103 | 0.0004095 | 0.57497 |
| AKR1C3 | -0.76515 | 4.7003111 | -4.24807 | 3.54E-05 | 0.0001582 | 1.5881 |
| LINC00672 | -0.76213 | 3.5455615 | -4.6640922 | 6.23E-06 | 3.38E-05 | 3.24192 |
| RP11-81K13.1 | -0.76162 | 1.1250326 | -4.700255 | 5.32E-06 | 2.94E-05 | 3.39137 |
| KBTBD11 | -0.76107 | 4.4822754 | -5.6270755 | 7.37E-08 | 6.49E-07 | 7.50941 |
| SLC14A2 | -0.76 | 1.1577174 | -5.3216518 | 3.19E-07 | 2.39E-06 | 6.09347 |
| GLRB | -0.75912 | 4.3475773 | -5.9258011 | 1.67E-08 | 1.73E-07 | 8.94616 |
| PRKAR1B | -0.75886 | 5.7193354 | -4.4708848 | 1.41E-05 | 7.05E-05 | 2.4587 |
| RP11-21A7A.2 | -0.75877 | 2.0520083 | -6.2253787 | 3.60E-09 | 4.45E-08 | 10.4355 |
| GNAO1 | -0.75841 | 6.5554643 | -6.971703 | 6.53E-11 | 1.28E-09 | 14.3378 |
| CXorf57 | -0.75766 | 3.2071156 | -4.9941193 | 1.45E-06 | 9.22E-06 | 4.63822 |
| FAM19A1 | -0.75745 | 1.7284169 | -4.42472 | 1.72E-05 | 8.33E-05 | 2.27541 |
| GDF10 | -0.75697 | 2.247942 | -4.0196968 | 8.72E-05 | 0.0003534 | 0.73324 |
| AC004158.3 | -0.75688 | 3.4672275 | -6.7604085 | 2.08E-10 | 3.53E-09 | 13.2069 |
| GPR37L1 | -0.7563 | 4.9419456 | -4.5857894 | 8.71E-06 | 4.58E-05 | 2.92138 |
| AC112229.7 | -0.7563 | 1.899446 | -3.1923383 | 0.00168 | 0.0048057 | -2.0267 |
| TMEM132D | -0.75626 | 1.3208084 | -4.8737041 | 2.49E-06 | 1.49E-05 | 4.1204 |
| C1orf95 | -0.7557 | 3.5678423 | -4.6793261 | 5.83E-06 | 3.19E-05 | 3.30477 |
| FAM13C | -0.75543 | 4.6949466 | -6.6832616 | 3.17E-10 | 5.07E-09 | 12.799 |
| LRRTM1 | -0.75419 | 2.9580435 | -5.2969598 | 3.59E-07 | 2.65E-06 | 5.98143 |
| APOD | -0.75404 | 7.7262283 | -4.4008604 | 1.89E-05 | 9.09E-05 | 2.18128 |
| ZNF488 | -0.75381 | 4.1532103 | -3.649559 | 0.000349 | 0.0012031 | -0.5685 |
| INHBA-AS1 | -0.75276 | 0.9141146 | -7.1537259 | 2.37E-11 | 5.30E-10 | 15.3274 |
| SPTBN2 | -0.75219 | 5.0495208 | -4.9571162 | 1.71E-06 | 1.07E-05 | 4.47809 |
| CACNA2D2 | -0.75105 | 1.9943919 | -7.068522 | 3.82E-11 | 8.03E-10 | 14.8625 |
| TMEM246 | -0.75037 | 3.6562556 | -5.4603495 | 1.65E-07 | 1.32E-06 | 6.72962 |
| DOCK3 | -0.75034 | 3.3296084 | -6.9575582 | 7.07E-11 | 1.37E-09 | 14.2615 |
| CBS | -0.75027 | 6.5102581 | -5.6763119 | 5.79E-08 | 5.25E-07 | 7.74277 |
| EPHA10 | -0.75012 | 1.7069397 | -5.898773 | 1.91E-08 | 1.96E-07 | 8.81414 |
| NECAB2 | -0.74957 | 3.8650046 | -4.3305175 | 2.53E-05 | 0.0001175 | 1.9061 |
| BRINP2 | -0.74939 | 4.6303419 | -5.2415233 | 4.65E-07 | 3.34E-06 | 5.73127 |
| RAB33A | -0.7489 | 4.1978463 | -5.5658001 | 9.93E-08 | 8.43E-07 | 7.22094 |
| NEFH | -0.74885 | 2.5328833 | -3.3524517 | 0.000986 | 0.0030057 | -1.5354 |
| DUSP9 | -0.74852 | 1.4979617 | -5.8783059 | 2.12E-08 | 2.14E-07 | 8.71442 |
| SERP2 | -0.74838 | 5.1193476 | -6.7486189 | 2.22E-10 | 3.73E-09 | 13.1444 |
| B3GAT1 | -0.74829 | 6.3343261 | -6.2874524 | 2.60E-09 | 3.33E-08 | 10.7499 |
| RP11-133F8.2 | -0.74667 | 1.6219723 | -4.3664603 | 2.18E-05 | 0.0001034 | 2.04626 |
| NRG3 | -0.74573 | 3.5584152 | -4.9747116 | 1.58E-06 | 9.95E-06 | 4.55412 |
| SEMA6B | -0.74546 | 4.7242257 | -5.3973482 | 2.23E-07 | 1.73E-06 | 6.43923 |
| LHFPL4 | -0.7452 | 3.9253822 | -5.5797598 | 9.28E-08 | 7.93E-07 | 7.28646 |
| TMEM35 | -0.74486 | 4.0197625 | -5.6129932 | 7.89E-08 | 6.90E-07 | 7.44292 |
| PHACTR3 | -0.74484 | 5.2919546 | -4.8070575 | 3.34E-06 | 1.94E-05 | 3.8379 |
| ZBTB16 | -0.74374 | 4.4143356 | -3.6709653 | 0.000323 | 0.0011217 | -0.4961 |
| SNCA | -0.74334 | 5.3346131 | -3.3152632 | 0.001118 | 0.0033566 | -1.6514 |
| SLC4A4 | -0.74265 | 5.5982495 | -4.4101634 | 1.82E-05 | 8.79E-05 | 2.21793 |
| KCNH8 | -0.74243 | 2.7970534 | -5.1213048 | 8.12E-07 | 5.46E-06 | 5.1953 |
| NSG1 | -0.74218 | 4.5962962 | -3.619276 | 0.000389 | 0.0013222 | -0.6703 |
| RP11-1C8.4 | -0.74218 | 1.016701 | -7.1646905 | 2.23E-11 | 5.01E-10 | 15.3874 |
| ANO4 | -0.74144 | 2.1317564 | -5.8607747 | 2.32E-08 | 2.31E-07 | 8.6292 |
| RP4-555D20.2 | -0.74079 | 2.1571141 | -4.3456284 | 2.38E-05 | 0.0001112 | 1.96491 |
| ZDHHC11B | -0.74028 | 3.7271429 | -5.7992991 | 3.15E-08 | 3.03E-07 | 8.33167 |
| SHANK3 | -0.73989 | 3.573047 | -6.6220003 | 4.41E-10 | 6.77E-09 | 12.477 |
| LRRC8A | -0.73894 | 6.6702695 | -6.8020182 | 1.66E-10 | 2.90E-09 | 13.4281 |
| STEAP2 | -0.73814 | 2.1359237 | -5.9976623 | 1.16E-08 | 1.25E-07 | 9.29911 |
| MAPT | -0.73799 | 7.0622559 | -5.9978547 | 1.16E-08 | 1.25E-07 | 9.30005 |
| ASPDH | -0.73783 | 3.3091159 | -4.7373762 | 4.53E-06 | 2.54E-05 | 3.5457 |
| C1QL3 | -0.73673 | 1.4633668 | -3.4206741 | 0.000781 | 0.0024332 | -1.3197 |
| NTNG2 | -0.73665 | 3.8920323 | -7.1236711 | 2.80E-11 | 6.11E-10 | 15.163 |
| RGS7BP | -0.73638 | 2.5132625 | -4.555668 | 9.90E-06 | 5.13E-05 | 2.79921 |
| ADCYAP1R1 | -0.73464 | 7.1536003 | -3.6346825 | 0.000368 | 0.0012607 | -0.6186 |
| GDA | -0.73408 | 2.4613249 | -2.8545481 | 0.004843 | 0.0120707 | -2.9926 |
| C14orf132 | -0.73375 | 5.6764166 | -5.8148537 | 2.91E-08 | 2.84E-07 | 8.40675 |
| MTND2P28 | -0.73295 | 7.9265916 | -5.8629823 | 2.29E-08 | 2.29E-07 | 8.63992 |
| KLHL32 | -0.73175 | 3.6954315 | -5.1486972 | 7.16E-07 | 4.90E-06 | 5.31663 |
| GABRB1 | -0.7306 | 2.8105299 | -4.9183974 | 2.04E-06 | 1.25E-05 | 4.31149 |
| DGKK | -0.73002 | 0.7553159 | -7.0944824 | 3.30E-11 | 7.08E-10 | 15.0038 |
| CTD-2562J17.7 | -0.73 | 3.0373603 | -4.5918366 | 8.49E-06 | 4.48E-05 | 2.94599 |
| RP11-14N7.2 | -0.72945 | 3.947753 | -3.9624579 | 0.000109 | 0.0004295 | 0.52508 |
| NDRG4 | -0.72923 | 7.8387366 | -6.017293 | 1.05E-08 | 1.14E-07 | 9.396 |
| GATSL3 | -0.72912 | 4.30708 | -5.1786647 | 6.23E-07 | 4.33E-06 | 5.4499 |
| RP11-143K11.5 | -0.72773 | 0.9554978 | -7.5659952 | 2.27E-12 | 6.77E-11 | 17.6178 |
| RP3-395M20.12 | -0.72716 | 1.6246203 | -4.5038444 | 1.23E-05 | 6.23E-05 | 2.59048 |
| RP11-496H1.2 | -0.72713 | 2.0554967 | -7.1484948 | 2.44E-11 | 5.41E-10 | 15.2988 |
| GALNT16 | -0.7263 | 4.1804698 | -6.5217991 | 7.55E-10 | 1.10E-08 | 11.9541 |
| RP11-267A15.1 | -0.72593 | 2.1734624 | -3.2419969 | 0.001427 | 0.0041673 | -1.8766 |
| SOWAHA | -0.72561 | 2.5124203 | -3.7246074 | 0.000265 | 0.0009462 | -0.3131 |
| AIFM3 | -0.72548 | 3.2887551 | -3.2139374 | 0.001565 | 0.0045202 | -1.9617 |
| MGAT5B | -0.72529 | 3.3454442 | -4.3874625 | 2.00E-05 | 9.57E-05 | 2.12859 |
| RP11-571M6.8 | -0.72507 | 2.8150486 | -4.2187475 | 3.98E-05 | 0.0001757 | 1.47619 |
| SPOCK3 | -0.7248 | 4.7093456 | -2.9432608 | 0.003699 | 0.0095258 | -2.7483 |
| LINC00237 | -0.72479 | 3.2093972 | -3.5495003 | 0.000499 | 0.001646 | -0.902 |
| ANKRD29 | -0.72451 | 2.0168032 | -5.6687031 | 6.01E-08 | 5.43E-07 | 7.70661 |
| PRDM8 | -0.72325 | 2.7752366 | -4.1516756 | 5.20E-05 | 0.0002229 | 1.22257 |
| TBR1 | -0.72207 | 1.788126 | -3.3884364 | 0.000873 | 0.0026898 | -1.4221 |
| ADAP1 | -0.72178 | 4.6490669 | -3.9594299 | 0.00011 | 0.0004337 | 0.51414 |
| SYBU | -0.72178 | 5.7020324 | -5.8006979 | 3.13E-08 | 3.01E-07 | 8.33842 |
| CGREF1 | -0.72149 | 4.4929294 | -5.2902186 | 3.70E-07 | 2.72E-06 | 5.95091 |
| LINC00263 | -0.7212 | 2.7966573 | -6.9882282 | 5.96E-11 | 1.18E-09 | 14.4271 |
| HAR1B | -0.72069 | 0.8522398 | -8.4355427 | 1.34E-14 | 7.01E-13 | 22.6447 |
| PAPLN | -0.72022 | 3.2003757 | -4.3390969 | 2.44E-05 | 0.0001139 | 1.93947 |
| TPD52L1 | -0.71961 | 4.6022442 | -3.6580595 | 0.000338 | 0.0011685 | -0.5398 |
| ADHFE1 | -0.71872 | 4.6072333 | -6.1752954 | 4.67E-09 | 5.56E-08 | 10.1833 |
| TSPAN7 | -0.71828 | 8.4122503 | -6.3793905 | 1.61E-09 | 2.16E-08 | 11.2191 |
| SCRT2 | -0.71792 | 1.4599247 | -5.2752734 | 3.97E-07 | 2.90E-06 | 5.88334 |
| RERGL | -0.71709 | 2.6532157 | -5.2554444 | 4.36E-07 | 3.15E-06 | 5.79391 |
| NRXN3 | -0.7163 | 3.209051 | -4.3443299 | 2.39E-05 | 0.0001117 | 1.95985 |
| FSTL5 | -0.7161 | 2.2871609 | -3.6194242 | 0.000389 | 0.0013217 | -0.6698 |
| DMTN | -0.7142 | 4.9446991 | -3.1857924 | 0.001716 | 0.0048934 | -2.0463 |
| SNAP25-AS1 | -0.71207 | 1.7759921 | -6.2208869 | 3.68E-09 | 4.52E-08 | 10.4128 |
| RP11-513M16.7 | -0.71146 | 2.5583178 | -5.349823 | 2.80E-07 | 2.12E-06 | 6.22174 |
| TF | -0.71093 | 7.780961 | -2.7114268 | 0.007384 | 0.0174557 | -3.3723 |
| AC131056.3 | -0.70981 | 1.1716652 | -5.3169259 | 3.27E-07 | 2.43E-06 | 6.072 |
| RAP1GAP2 | -0.70945 | 2.9146639 | -4.5820104 | 8.85E-06 | 4.64E-05 | 2.90602 |
| PTPRN2 | -0.70905 | 5.0557284 | -5.8446863 | 2.51E-08 | 2.49E-07 | 8.55113 |
| SLC1A4 | -0.70882 | 5.6067826 | -4.9855896 | 1.51E-06 | 9.53E-06 | 4.60123 |
| HSPA12A | -0.70851 | 3.7531099 | -4.9461273 | 1.80E-06 | 1.12E-05 | 4.43071 |
| ICAM5 | -0.70647 | 2.5456061 | -3.2331566 | 0.001469 | 0.0042737 | -1.9035 |
| MUSTN1 | -0.70543 | 2.5319302 | -4.4101221 | 1.82E-05 | 8.79E-05 | 2.21777 |
| GAD2 | -0.70432 | 1.86024 | -3.1983668 | 0.001647 | 0.0047231 | -2.0086 |
| BCL7A | -0.70425 | 4.2825368 | -5.4375487 | 1.84E-07 | 1.46E-06 | 6.62425 |
| CPLX3 | -0.7037 | 1.509001 | -4.0279128 | 8.45E-05 | 0.0003436 | 0.76333 |
| PRR18 | -0.70271 | 3.519294 | -3.3696355 | 0.00093 | 0.0028537 | -1.4814 |
| TESPA1 | -0.70261 | 1.7700629 | -2.9386377 | 0.003752 | 0.0096477 | -2.7612 |
| PCDHGA10 | -0.70247 | 2.8335266 | -2.4149804 | 0.016791 | 0.0356413 | -4.1015 |
| PPP4R4 | -0.69943 | 2.1168346 | -4.0146826 | 8.90E-05 | 0.0003593 | 0.71491 |
| CTD-2589H19.4 | -0.6991 | 1.9439168 | -3.3128079 | 0.001127 | 0.0033811 | -1.659 |
| TBC1D10A | -0.69848 | 4.6841513 | -5.7280761 | 4.48E-08 | 4.18E-07 | 7.98959 |
| RASD2 | -0.69812 | 2.4132982 | -4.2766799 | 3.15E-05 | 0.0001431 | 1.69789 |
| PLEKHB1 | -0.69743 | 8.7210175 | -4.9882914 | 1.49E-06 | 9.44E-06 | 4.61294 |
| TCEAL2 | -0.6965 | 6.8899923 | -4.7466149 | 4.35E-06 | 2.45E-05 | 3.58425 |
| TOX | -0.69593 | 3.6631297 | -4.6433633 | 6.81E-06 | 3.67E-05 | 3.15666 |
| AP000356.1 | -0.69563 | 3.3469834 | -3.9772686 | 0.000103 | 0.0004082 | 0.57871 |
| SLC25A21-AS1 | -0.69529 | 2.2895447 | -5.5033584 | 1.34E-07 | 1.10E-06 | 6.92922 |
| HAPLN2 | -0.69478 | 5.2690411 | -2.4352025 | 0.015911 | 0.0340657 | -4.0542 |
| RP11-728F11.4 | -0.69432 | 2.3694011 | -4.4337763 | 1.65E-05 | 8.06E-05 | 2.31125 |
| KIF5C | -0.69399 | 6.2671102 | -5.3967244 | 2.24E-07 | 1.73E-06 | 6.43636 |
| CNTN4 | -0.69397 | 1.8865659 | -5.1664942 | 6.59E-07 | 4.55E-06 | 5.39571 |
| MT-ND1 | -0.69386 | 13.24071 | -6.6857725 | 3.13E-10 | 5.01E-09 | 12.8122 |
| KIT | -0.69362 | 2.9260178 | -4.7892513 | 3.61E-06 | 2.08E-05 | 3.76292 |
| LINC00087 | -0.69295 | 2.5215582 | -4.2039766 | 4.22E-05 | 0.0001851 | 1.42005 |
| 5-Sep | -0.6928 | 6.6242705 | -4.339543 | 2.44E-05 | 0.0001137 | 1.94121 |
| EPHB6 | -0.6923 | 3.2781625 | -3.0548045 | 0.002613 | 0.0070384 | -2.4316 |
| RP11-706O15.1 | -0.69205 | 3.4903672 | -4.5229948 | 1.14E-05 | 5.79E-05 | 2.66739 |
| MAP2 | -0.69204 | 7.5284283 | -5.5040637 | 1.34E-07 | 1.09E-06 | 6.9325 |
| SUSD5 | -0.69175 | 3.1194261 | -3.0846578 | 0.002377 | 0.0064926 | -2.3451 |
| ITPK1 | -0.69058 | 6.9389305 | -7.5088025 | 3.16E-12 | 9.04E-11 | 17.2961 |
| OLFM4 | -0.68581 | 0.9421658 | -6.4647515 | 1.02E-09 | 1.43E-08 | 11.6585 |
| NTM | -0.68573 | 6.3379662 | -6.1685966 | 4.83E-09 | 5.75E-08 | 10.1496 |
| STXBP5L | -0.68547 | 1.8237806 | -4.7488819 | 4.31E-06 | 2.43E-05 | 3.59372 |
| DISP2 | -0.6852 | 3.0108927 | -5.4743997 | 1.54E-07 | 1.24E-06 | 6.79471 |
| RP11-513I15.6 | -0.68454 | 3.6869212 | -4.2115458 | 4.09E-05 | 0.0001802 | 1.4488 |
| ARRB1 | -0.6843 | 4.4613813 | -5.4059725 | 2.14E-07 | 1.67E-06 | 6.47884 |
| PAK7 | -0.68244 | 2.064336 | -5.1329167 | 7.69E-07 | 5.23E-06 | 5.24668 |
| GREB1 | -0.68231 | 2.7264372 | -7.0297316 | 4.74E-11 | 9.70E-10 | 14.6518 |
| KNDC1 | -0.68218 | 3.3081793 | -4.3055984 | 2.80E-05 | 0.0001286 | 1.80947 |
| CARNS1 | -0.68194 | 4.3061025 | -2.2928755 | 0.023074 | 0.0468285 | -4.379 |
| GLRA3 | -0.68184 | 1.3414165 | -4.3459345 | 2.37E-05 | 0.0001112 | 1.96611 |
| KIF3C | -0.68142 | 5.5932866 | -5.8635161 | 2.28E-08 | 2.28E-07 | 8.64251 |
| PDIA2 | -0.6812 | 3.4445096 | -4.1507342 | 5.22E-05 | 0.0002236 | 1.21904 |
| TNNT2 | -0.68096 | 1.4597482 | -3.3878423 | 0.000874 | 0.0026943 | -1.424 |
| RP11-74H8.1 | -0.68057 | 1.3380333 | -3.3031501 | 0.001164 | 0.0034776 | -1.6889 |
| MTMR7 | -0.67976 | 2.9293706 | -5.1684371 | 6.53E-07 | 4.51E-06 | 5.40435 |
| LHX2 | -0.67967 | 4.4263165 | -4.084691 | 6.77E-05 | 0.0002815 | 0.9726 |
| MFSD4 | -0.6794 | 3.5809155 | -4.3209052 | 2.63E-05 | 0.0001217 | 1.86877 |
| ALDH1A1 | -0.67796 | 4.7673877 | -3.2383262 | 0.001445 | 0.0042105 | -1.8878 |
| NAP1L3 | -0.67678 | 5.625472 | -4.9925056 | 1.46E-06 | 9.29E-06 | 4.63122 |
| SMAD9 | -0.67658 | 4.2275066 | -5.0467829 | 1.14E-06 | 7.43E-06 | 4.86764 |
| LY6H | -0.67599 | 5.1416158 | -2.8038114 | 0.005635 | 0.0137733 | -3.1292 |
| ANKS1B | -0.67575 | 5.3266967 | -4.2463525 | 3.56E-05 | 0.0001592 | 1.58152 |
| ASPA | -0.67533 | 3.0288023 | -3.6425692 | 0.000358 | 0.0012294 | -0.5921 |
| TMEM59L | -0.67507 | 7.0594965 | -5.6267538 | 7.38E-08 | 6.49E-07 | 7.50789 |
| CPB2-AS1 | -0.6746 | 2.2191739 | -7.6451007 | 1.44E-12 | 4.47E-11 | 18.0646 |
| RP11-31F19.1 | -0.67406 | 2.8027821 | -4.1972301 | 4.34E-05 | 0.0001895 | 1.39446 |
| REEP6 | -0.67303 | 3.7382857 | -6.2279327 | 3.55E-09 | 4.39E-08 | 10.4484 |
| RP1-239B22.5 | -0.67232 | 1.517476 | -6.4241243 | 1.27E-09 | 1.74E-08 | 11.4489 |
| SYNGR1 | -0.67207 | 5.3032622 | -5.2677993 | 4.11E-07 | 2.99E-06 | 5.84961 |
| DIO2 | -0.67183 | 3.1211757 | -3.6971299 | 0.000293 | 0.0010319 | -0.4071 |
| RP11-481A20.10 | -0.67172 | 1.1490422 | -4.2133122 | 4.07E-05 | 0.0001791 | 1.45551 |
| FAM131C | -0.67123 | 2.0979599 | -4.9048456 | 2.16E-06 | 1.32E-05 | 4.25341 |
| MTSS1L | -0.67073 | 8.4466277 | -6.1994757 | 4.12E-09 | 5.00E-08 | 10.3049 |
| MAP7D2 | -0.6704 | 2.1628637 | -2.7482323 | 0.006635 | 0.0158899 | -3.2764 |
| AFAP1L2 | -0.66576 | 3.9968727 | -3.7518064 | 0.00024 | 0.000866 | -0.2195 |
| ALDH6A1 | -0.66543 | 5.5092212 | -6.0729345 | 7.90E-09 | 8.89E-08 | 9.67175 |
| KCNK12 | -0.66491 | 1.5299291 | -5.0879236 | 9.46E-07 | 6.27E-06 | 5.04809 |
| ASIC2 | -0.66482 | 1.3906829 | -4.5124525 | 1.19E-05 | 6.03E-05 | 2.62502 |
| BCYRN1 | -0.66421 | 1.6501309 | -3.5342484 | 0.000526 | 0.0017276 | -0.9522 |
| KCNIP1 | -0.66383 | 4.8605603 | -4.2825213 | 3.08E-05 | 0.00014 | 1.72038 |
| SERPINI1 | -0.66354 | 5.1110871 | -2.6849533 | 0.007968 | 0.018646 | -3.4406 |
| PIP4K2A | -0.66353 | 5.7171268 | -4.9334211 | 1.90E-06 | 1.18E-05 | 4.37602 |
| GAD1 | -0.66345 | 4.5650037 | -3.3053819 | 0.001156 | 0.0034544 | -1.682 |
| SLC32A1 | -0.66316 | 1.8301862 | -3.0943486 | 0.002305 | 0.0063247 | -2.3168 |
| C1orf115 | -0.66224 | 3.0777247 | -3.2264715 | 0.001502 | 0.0043553 | -1.9237 |
| SSTR1 | -0.66204 | 1.9657594 | -3.6572656 | 0.000339 | 0.0011717 | -0.5425 |
| FAM133A | -0.66162 | 2.0019565 | -5.3861786 | 2.35E-07 | 1.81E-06 | 6.38799 |
| KLF15 | -0.66034 | 5.0518225 | -4.1321201 | 5.62E-05 | 0.0002384 | 1.14925 |
| RP11-513M16.8 | -0.65913 | 2.6692848 | -5.6438885 | 6.78E-08 | 6.03E-07 | 7.58894 |
| HMGN2P15 | -0.65889 | 2.8359095 | -3.9053739 | 0.000135 | 0.0005212 | 0.31995 |
| PANX2 | -0.65824 | 2.5285597 | -4.464306 | 1.45E-05 | 7.22E-05 | 2.43249 |
| RP11-547I7.2 | -0.6581 | 3.4434112 | -4.0020735 | 9.34E-05 | 0.0003752 | 0.66889 |
| BCR | -0.65785 | 6.0355597 | -5.7530491 | 3.96E-08 | 3.73E-07 | 8.10921 |
| CTD-2081K17.2 | -0.65783 | 2.2497898 | -3.0263189 | 0.002858 | 0.007602 | -2.5135 |
| PCSK6 | -0.65775 | 4.5666857 | -2.7411357 | 0.006774 | 0.0161744 | -3.295 |
| NEUROD2 | -0.65726 | 1.8888805 | -2.977427 | 0.003329 | 0.0086895 | -2.6524 |
| RASAL1 | -0.65703 | 2.0764902 | -3.1126536 | 0.002174 | 0.006015 | -2.2632 |
| MYL3 | -0.65639 | 2.3969828 | -5.0537076 | 1.11E-06 | 7.23E-06 | 4.89794 |
| AC013402.2 | -0.65631 | 3.4096052 | -4.3734946 | 2.12E-05 | 0.0001008 | 2.0738 |
| FAM107A | -0.65588 | 8.3597841 | -3.5257076 | 0.000542 | 0.001771 | -0.9802 |
| RP11-401P9.4 | -0.65527 | 3.888845 | -3.6875026 | 0.000304 | 0.0010641 | -0.4399 |
| PNMA3 | -0.65504 | 2.7388669 | -3.9223323 | 0.000127 | 0.0004918 | 0.38063 |
| MAGEH1 | -0.65481 | 6.2207206 | -5.5161134 | 1.26E-07 | 1.04E-06 | 6.98862 |
| VIPR1 | -0.65433 | 1.9606536 | -4.8615866 | 2.62E-06 | 1.57E-05 | 4.06882 |
| PTGDS | -0.65426 | 9.4785038 | -2.715612 | 0.007295 | 0.0172726 | -3.3615 |
| CAMK2G | -0.65387 | 5.917931 | -5.5369647 | 1.14E-07 | 9.47E-07 | 7.08594 |
| RAPGEF3 | -0.6538 | 4.1687756 | -5.4138258 | 2.06E-07 | 1.61E-06 | 6.51495 |
| C7orf41 | -0.65366 | 7.3009843 | -4.4396815 | 1.61E-05 | 7.90E-05 | 2.33465 |
| MADCAM1 | -0.65271 | 1.5350029 | -6.3257308 | 2.13E-09 | 2.79E-08 | 10.9448 |
| PCSK1N | -0.65247 | 7.7525216 | -4.4359881 | 1.64E-05 | 8.00E-05 | 2.32001 |
| TCEAL6 | -0.65212 | 3.0423014 | -2.664923 | 0.008438 | 0.0196285 | -3.4918 |
| AC004019.13 | -0.65168 | 1.1543796 | -5.9962387 | 1.17E-08 | 1.26E-07 | 9.29209 |
| BASP1 | -0.65137 | 6.6190289 | -4.237503 | 3.69E-05 | 0.0001643 | 1.54769 |
| MT-RNR2 | -0.65123 | 13.901489 | -6.5155266 | 7.80E-10 | 1.13E-08 | 11.9215 |
| SOHLH1 | -0.65118 | 1.1445504 | -4.0679416 | 7.23E-05 | 0.0002982 | 0.91061 |
| PIANP | -0.65113 | 5.1255178 | -4.6685959 | 6.11E-06 | 3.32E-05 | 3.26048 |
| RP11-701H24.3 | -0.65098 | 2.2673655 | -5.0002771 | 1.41E-06 | 8.99E-06 | 4.66495 |
| DLG2 | -0.65055 | 4.1455162 | -4.7256988 | 4.77E-06 | 2.66E-05 | 3.49705 |
| MT-RNR1 | -0.65026 | 12.550273 | -5.7179471 | 4.71E-08 | 4.37E-07 | 7.94118 |
| CDH10 | -0.65013 | 4.2017738 | -4.4129024 | 1.80E-05 | 8.71E-05 | 2.22874 |
| OLFM3 | -0.65013 | 1.4982496 | -3.5460362 | 0.000505 | 0.0016638 | -0.9134 |
| RP1-35C21.2 | -0.64993 | 0.8122828 | -4.3027593 | 2.83E-05 | 0.0001299 | 1.79849 |
| NCAM2 | -0.649 | 5.4290451 | -5.0555759 | 1.10E-06 | 7.17E-06 | 4.90611 |
| ANK1 | -0.64845 | 2.7355969 | -6.1633483 | 4.96E-09 | 5.87E-08 | 10.1233 |
| NCAN | -0.64779 | 6.9646059 | -4.1395114 | 5.46E-05 | 0.0002325 | 1.17693 |
| SH2D5 | -0.64701 | 1.8162853 | -3.1885873 | 0.001701 | 0.0048564 | -2.0379 |
| FAM81A | -0.64701 | 3.3542251 | -4.2694133 | 3.24E-05 | 0.0001468 | 1.66995 |
| PCDH20 | -0.64669 | 1.9792423 | -4.3872243 | 2.00E-05 | 9.58E-05 | 2.12766 |
| KCNQ2 | -0.64648 | 5.4557864 | -4.078479 | 6.94E-05 | 0.0002875 | 0.94958 |
| APC2 | -0.64587 | 6.4922744 | -5.3017707 | 3.51E-07 | 2.60E-06 | 6.00323 |
| LRRN1 | -0.64532 | 5.7366071 | -3.3812899 | 0.000894 | 0.0027503 | -1.4447 |
| EPHB1 | -0.64492 | 4.6265788 | -3.9159847 | 0.00013 | 0.0005026 | 0.35789 |
| NAPB | -0.64489 | 4.7569619 | -3.1530112 | 0.001909 | 0.0053688 | -2.1441 |
| SNTG1 | -0.64487 | 3.1052882 | -4.4039786 | 1.87E-05 | 9.00E-05 | 2.19356 |
| SEC14L5 | -0.64467 | 2.2101963 | -3.0967949 | 0.002287 | 0.0062829 | -2.3097 |
| PRKCE | -0.64413 | 3.7137375 | -5.2110602 | 5.36E-07 | 3.78E-06 | 5.5946 |
| SYT9 | -0.6438 | 2.2041685 | -5.8124414 | 2.95E-08 | 2.86E-07 | 8.3951 |
| CSMD1 | -0.64308 | 2.6149376 | -4.0638001 | 7.35E-05 | 0.0003026 | 0.89532 |
| DAAM2 | -0.64305 | 6.4560937 | -2.766452 | 0.006291 | 0.0151636 | -3.2284 |
| LYNX1 | -0.64198 | 4.8676701 | -4.698607 | 5.36E-06 | 2.95E-05 | 3.38454 |
| BMP7 | -0.64187 | 5.4781199 | -5.1780196 | 6.25E-07 | 4.34E-06 | 5.44703 |
| KCNJ4 | -0.64181 | 2.5707067 | -2.688154 | 0.007896 | 0.0184933 | -3.4324 |
| ALPL | -0.64176 | 3.6624255 | -6.5689538 | 5.86E-10 | 8.74E-09 | 12.1996 |
| FAM211B | -0.64126 | 4.2390405 | -6.1870812 | 4.39E-09 | 5.27E-08 | 10.2425 |
| TMEM132B | -0.63988 | 3.8625946 | -3.9516786 | 0.000113 | 0.0004453 | 0.48616 |
| VAX2 | -0.63946 | 2.9887671 | -4.1903315 | 4.46E-05 | 0.0001941 | 1.36834 |
| DRG2 | -0.63791 | 4.3907423 | -5.6282302 | 7.33E-08 | 6.46E-07 | 7.51486 |
| NLGN3 | -0.63774 | 5.8214373 | -6.3662713 | 1.72E-09 | 2.30E-08 | 11.1519 |
| TRIM17 | -0.63754 | 1.966131 | -5.7763698 | 3.53E-08 | 3.36E-07 | 8.22123 |
| HSPB8 | -0.63703 | 6.7910517 | -3.1244717 | 0.002093 | 0.0058203 | -2.2285 |
| PPP1R13B | -0.63563 | 3.4257485 | -7.0881472 | 3.42E-11 | 7.28E-10 | 14.9693 |
| GABBR2 | -0.63556 | 4.5973927 | -3.026039 | 0.002861 | 0.007607 | -2.5143 |
| C12orf39 | -0.63533 | 3.3848814 | -2.6694516 | 0.00833 | 0.0194098 | -3.4803 |
| ACSBG1 | -0.63443 | 5.2047771 | -3.8493452 | 0.000167 | 0.0006285 | 0.12103 |
| CACNA1A | -0.63385 | 4.2350309 | -4.6401355 | 6.90E-06 | 3.71E-05 | 3.1434 |
| WDFY3-AS2 | -0.63333 | 3.5252319 | -5.9389467 | 1.56E-08 | 1.63E-07 | 9.01052 |
| TLX1NB | -0.63282 | 0.6625389 | -6.0951724 | 7.05E-09 | 8.05E-08 | 9.78242 |
| DGKB | -0.63242 | 3.3699406 | -4.1662077 | 4.91E-05 | 0.0002115 | 1.27724 |
| LHPP | -0.63241 | 6.2353855 | -3.5746584 | 0.000456 | 0.001524 | -0.8189 |
| MTND1P23 | -0.63205 | 2.6378987 | -2.4185944 | 0.01663 | 0.0353411 | -4.0931 |
| ITPKA | -0.63204 | 2.8029731 | -3.2935058 | 0.001203 | 0.0035774 | -1.7187 |
| USP54 | -0.6316 | 5.8909507 | -4.9113188 | 2.10E-06 | 1.29E-05 | 4.28114 |
| RP11-279F6.1 | -0.63094 | 1.6748473 | -5.4210733 | 1.99E-07 | 1.56E-06 | 6.54831 |
| GPR75 | -0.63077 | 3.9251628 | -4.5111006 | 1.19E-05 | 6.06E-05 | 2.61959 |
| PEA15 | -0.63031 | 10.424343 | -5.7886713 | 3.32E-08 | 3.18E-07 | 8.28045 |
| AC079305.11 | -0.62997 | 0.844044 | -6.2234182 | 3.63E-09 | 4.48E-08 | 10.4256 |
| MAP1A | -0.62983 | 5.963572 | -5.6249944 | 7.44E-08 | 6.54E-07 | 7.49957 |
| DNMBP-AS1 | -0.62916 | 1.0206527 | -5.1908772 | 5.89E-07 | 4.12E-06 | 5.50437 |
| FN3K | -0.62842 | 5.3723573 | -7.4606808 | 4.16E-12 | 1.15E-10 | 17.0265 |
| KCNH3 | -0.62827 | 2.7823037 | -2.9423429 | 0.00371 | 0.0095507 | -2.7509 |
| GLP1R | -0.62826 | 0.7245469 | -4.8138766 | 3.24E-06 | 1.89E-05 | 3.86667 |
| KCNK4 | -0.62823 | 1.6079956 | -4.6680043 | 6.12E-06 | 3.33E-05 | 3.25804 |
| TSPYL2 | -0.62821 | 6.170222 | -5.3041948 | 3.47E-07 | 2.57E-06 | 6.01422 |
| AC140481.7 | -0.62794 | 1.2533277 | -3.7190674 | 0.000271 | 0.0009622 | -0.3321 |
| NRXN2 | -0.62791 | 7.0856463 | -5.6026956 | 8.30E-08 | 7.21E-07 | 7.39437 |
| HTR1A | -0.62778 | 1.3205902 | -3.8416456 | 0.000172 | 0.000645 | 0.09388 |
| FUT9 | -0.62753 | 3.2884007 | -4.365645 | 2.19E-05 | 0.0001037 | 2.04307 |
| TAC1 | -0.62621 | 2.2552552 | -2.3837334 | 0.018234 | 0.0382982 | -4.1738 |
| MAGEE1 | -0.62608 | 3.095549 | -4.6773831 | 5.88E-06 | 3.21E-05 | 3.29674 |
| PAQR6 | -0.62502 | 6.9492479 | -3.8075125 | 0.000195 | 0.0007206 | -0.0259 |
| WASF1 | -0.62483 | 5.331326 | -4.6822565 | 5.76E-06 | 3.15E-05 | 3.31687 |
| RP1-257A7.5 | -0.62475 | 4.4858724 | -4.4273016 | 1.70E-05 | 8.25E-05 | 2.28562 |
| CD8BP | -0.62412 | 0.8652024 | -6.6994196 | 2.90E-10 | 4.71E-09 | 12.8842 |
| CHRNA7 | -0.62315 | 1.6224268 | -5.4762161 | 1.53E-07 | 1.23E-06 | 6.80313 |
| RIMBP2 | -0.62297 | 2.1202195 | -3.4352363 | 0.000743 | 0.002333 | -1.2731 |
| FRMPD1 | -0.62295 | 1.4608796 | -6.2601639 | 3.00E-09 | 3.79E-08 | 10.6115 |
| DMRTC1B | -0.62279 | 2.947569 | -5.1170748 | 8.28E-07 | 5.56E-06 | 5.17661 |
| CTD-2210P24.4 | -0.62205 | 2.0587989 | -4.8701799 | 2.53E-06 | 1.51E-05 | 4.10539 |
| MT-ND2 | -0.62198 | 13.46061 | -6.4543687 | 1.08E-09 | 1.51E-08 | 11.6048 |
| KCNH7 | -0.62177 | 1.1237658 | -5.3741265 | 2.49E-07 | 1.91E-06 | 6.33279 |
| RND1 | -0.62133 | 4.2075124 | -4.3968495 | 1.93E-05 | 9.24E-05 | 2.16549 |
| ABLIM2 | -0.62121 | 3.3116074 | -4.4841766 | 1.34E-05 | 6.71E-05 | 2.51175 |
| RP11-811P12.3 | -0.62088 | 1.5812882 | -4.7898036 | 3.60E-06 | 2.07E-05 | 3.76524 |
| NXPH1 | -0.62084 | 4.3997056 | -2.9818671 | 0.003284 | 0.0085882 | -2.6399 |
| RNF185-AS1 | -0.61999 | 3.3520754 | -3.4322819 | 0.000751 | 0.0023523 | -1.2826 |
| HMGCLL1 | -0.61972 | 2.2244764 | -3.8332636 | 0.000177 | 0.0006628 | 0.06438 |
| CRHR1 | -0.6194 | 1.8254602 | -5.2849576 | 3.80E-07 | 2.79E-06 | 5.92711 |
| IFITM10 | -0.61817 | 4.1015605 | -4.1719071 | 4.80E-05 | 0.0002073 | 1.29872 |
| CTC-458G6.2 | -0.61814 | 0.9127296 | -5.32872 | 3.09E-07 | 2.32E-06 | 6.12561 |
| ACAN | -0.61799 | 1.9975045 | -3.0275391 | 0.002847 | 0.0075762 | -2.51 |
| CTB-119C2.1 | -0.61705 | 1.4135052 | -6.1306003 | 5.88E-09 | 6.84E-08 | 9.95925 |
| GABRA4 | -0.61589 | 1.3837481 | -3.978704 | 0.000102 | 0.0004062 | 0.58391 |
| MYO16 | -0.61544 | 2.3586835 | -4.5099958 | 1.20E-05 | 6.09E-05 | 2.61515 |
| HES6 | -0.61523 | 7.5880993 | -2.904801 | 0.004161 | 0.0105635 | -2.8551 |
| NKAIN4 | -0.61272 | 6.7184132 | -2.7178753 | 0.007247 | 0.0171749 | -3.3556 |
| IQCA1 | -0.61232 | 2.0605112 | -3.7233468 | 0.000267 | 0.0009496 | -0.3175 |
| RP11-728K20.1 | -0.61209 | 1.9469572 | -3.5618378 | 0.000477 | 0.0015849 | -0.8613 |
| RAMP2-AS1 | -0.61197 | 2.3912952 | -4.7768811 | 3.81E-06 | 2.18E-05 | 3.71096 |
| AJAP1 | -0.61156 | 1.918794 | -4.9746994 | 1.58E-06 | 9.95E-06 | 4.55407 |
| DYNC1I1 | -0.61104 | 3.7879688 | -3.2668353 | 0.001314 | 0.0038711 | -1.8007 |
| AC013268.5 | -0.61102 | 1.3540506 | -3.0629492 | 0.002547 | 0.0068815 | -2.4081 |
| ZNF98 | -0.6103 | 1.3343532 | -4.6294835 | 7.22E-06 | 3.87E-05 | 3.09973 |
| RP11-58B17.2 | -0.61011 | 4.8345795 | -4.5896252 | 8.57E-06 | 4.51E-05 | 2.93699 |
| GRIP2 | -0.60901 | 1.4201199 | -5.0720977 | 1.02E-06 | 6.70E-06 | 4.97855 |
| CPNE6 | -0.60847 | 3.3026344 | -2.3787152 | 0.018476 | 0.0387358 | -4.1853 |
| KCTD4 | -0.60804 | 2.4417774 | -3.8483988 | 0.000168 | 0.0006306 | 0.11769 |
| PPP1R14C | -0.60798 | 2.4426976 | -3.7212712 | 0.000269 | 0.0009556 | -0.3246 |
| PDZD8 | -0.60797 | 4.3392567 | -6.4961645 | 8.65E-10 | 1.24E-08 | 11.821 |
| PLEKHA6 | -0.60757 | 3.5655261 | -5.3574408 | 2.70E-07 | 2.05E-06 | 6.25651 |
| HIF3A | -0.60747 | 3.1504425 | -3.0470539 | 0.002678 | 0.0071872 | -2.454 |
| RCAN2 | -0.6072 | 4.3961351 | -3.1861034 | 0.001714 | 0.004889 | -2.0454 |
| CACNA1I | -0.60674 | 1.196906 | -5.1776403 | 6.26E-07 | 4.34E-06 | 5.44534 |
| ANGPTL2 | -0.60659 | 5.9260454 | -3.0428093 | 0.002714 | 0.0072712 | -2.4662 |
| NPPA | -0.60576 | 3.4537946 | -2.407244 | 0.017138 | 0.0362812 | -4.1195 |
| CYFIP2 | -0.60559 | 5.9251 | -6.4310289 | 1.22E-09 | 1.68E-08 | 11.4845 |
| TMEM235 | -0.60543 | 2.5483922 | -2.414389 | 0.016817 | 0.0356941 | -4.1029 |
| MATK | -0.60535 | 2.6055859 | -3.5119699 | 0.000569 | 0.0018497 | -1.0251 |
| LINC00693 | -0.60485 | 3.1883339 | -4.398544 | 1.91E-05 | 9.18E-05 | 2.17216 |
| RGS11 | -0.60483 | 5.0078329 | -4.2304726 | 3.79E-05 | 0.0001685 | 1.52086 |
| GS1-72M22.1 | -0.60473 | 1.6670047 | -4.325637 | 2.58E-05 | 0.0001196 | 1.88714 |
| DDX25 | -0.6047 | 3.9142521 | -3.5595873 | 0.000481 | 0.0015954 | -0.8688 |
| TUBA8 | -0.60408 | 2.5700103 | -3.2798186 | 0.001259 | 0.0037241 | -1.7609 |
| LINC00943 | -0.60392 | 1.2197216 | -4.7825526 | 3.72E-06 | 2.13E-05 | 3.73477 |
| KCNJ10 | -0.60382 | 6.3910424 | -5.119124 | 8.20E-07 | 5.52E-06 | 5.18567 |
| MYOZ1 | -0.60366 | 1.5558992 | -5.7042353 | 5.04E-08 | 4.65E-07 | 7.87573 |
| LINC00632 | -0.60355 | 3.3346855 | -4.3459873 | 2.37E-05 | 0.0001112 | 1.96631 |
| CCNI2 | -0.60297 | 3.4630504 | -3.031103 | 0.002816 | 0.0075059 | -2.4998 |
| PRRT1 | -0.60296 | 4.5971543 | -5.1885337 | 5.95E-07 | 4.16E-06 | 5.49391 |
| PWAR6 | -0.60291 | 4.0603228 | -4.2533876 | 3.46E-05 | 0.0001553 | 1.60846 |
| TMOD2 | -0.60274 | 5.5222152 | -6.3067155 | 2.35E-09 | 3.05E-08 | 10.8479 |
| KCNK1 | -0.60272 | 3.5301818 | -2.8818472 | 0.004461 | 0.011243 | -2.9181 |
| CXXC4 | -0.60208 | 3.4628723 | -4.2181741 | 3.99E-05 | 0.000176 | 1.474 |
| FAM149A | -0.60171 | 4.0199867 | -5.6674087 | 6.05E-08 | 5.45E-07 | 7.70047 |
| STMN3 | -0.60102 | 7.0987406 | -5.9931934 | 1.19E-08 | 1.27E-07 | 9.27708 |
| DSCAM | -0.6009 | 3.3353498 | -3.7131565 | 0.000277 | 0.0009802 | -0.3524 |
| GPM6A | -0.60058 | 9.1674141 | -5.1754794 | 6.32E-07 | 4.38E-06 | 5.43571 |
| SLC2A13 | -0.60004 | 3.4438896 | -3.9595004 | 0.00011 | 0.0004336 | 0.51439 |
| CIT | -0.5998 | 4.5826089 | -4.8244772 | 3.09E-06 | 1.81E-05 | 3.91145 |
| NCAM1 | -0.59939 | 8.1844321 | -5.6676783 | 6.04E-08 | 5.45E-07 | 7.70175 |
| EDA2R | -0.59872 | 2.1453477 | -3.4679431 | 0.000664 | 0.0021154 | -1.168 |
| CTNNA3 | -0.59823 | 2.4462131 | -4.5439379 | 1.04E-05 | 5.36E-05 | 2.7518 |
| DNAJC6 | -0.59763 | 5.0178798 | -4.1895262 | 4.47E-05 | 0.0001946 | 1.36529 |
| PCDH8 | -0.59691 | 2.3579313 | -3.4392743 | 0.000733 | 0.0023036 | -1.2602 |
| RAPGEFL1 | -0.59678 | 3.9565119 | -5.1394357 | 7.47E-07 | 5.09E-06 | 5.27556 |
| LRRC4C | -0.59648 | 4.2108083 | -6.0553591 | 8.65E-09 | 9.65E-08 | 9.58448 |
| RAPGEF5 | -0.59643 | 4.2356037 | -3.3311322 | 0.00106 | 0.0031994 | -1.602 |
| P2RX6 | -0.59562 | 2.9601061 | -4.732687 | 4.62E-06 | 2.58E-05 | 3.52615 |
| C17orf96 | -0.59556 | 3.0379206 | -5.0874846 | 9.48E-07 | 6.28E-06 | 5.04616 |
| FASN | -0.59536 | 6.3836192 | -7.2892025 | 1.10E-11 | 2.69E-10 | 16.0727 |
| NGB | -0.59465 | 1.1044822 | -3.1285014 | 0.002066 | 0.0057527 | -2.2166 |
| ENTPD3 | -0.59398 | 1.2509491 | -4.0951394 | 6.50E-05 | 0.0002713 | 1.01137 |
| BCAS1 | -0.59322 | 6.1498855 | -2.3285462 | 0.021054 | 0.0433108 | -4.2993 |
| LUZP2 | -0.59275 | 4.8355832 | -2.3306311 | 0.02094 | 0.0430927 | -4.2946 |
| RP11-1134I14.4 | -0.59249 | 2.2674496 | -4.2845261 | 3.05E-05 | 0.0001389 | 1.7281 |
| RP11-355I22.7 | -0.59175 | 2.5062541 | -3.2272991 | 0.001498 | 0.0043461 | -1.9212 |
| BTNL9 | -0.59111 | 2.7424277 | -4.494558 | 1.28E-05 | 6.46E-05 | 2.55327 |
| BAI1 | -0.59064 | 5.2424668 | -2.8523169 | 0.004876 | 0.0121388 | -2.9986 |
| CECR6 | -0.58997 | 3.0033947 | -6.4128949 | 1.35E-09 | 1.83E-08 | 11.3911 |
| EMX1 | -0.58949 | 1.3594628 | -3.1809515 | 0.001744 | 0.0049605 | -2.0608 |
| LPL | -0.58938 | 5.5022993 | -2.3626424 | 0.01927 | 0.0401609 | -4.2221 |
| GJD2 | -0.58914 | 0.9233517 | -4.7596987 | 4.11E-06 | 2.33E-05 | 3.63895 |
| GRID2 | -0.58891 | 2.9535435 | -3.5710877 | 0.000462 | 0.0015402 | -0.8308 |
| THRB | -0.58832 | 3.2323341 | -4.5113548 | 1.19E-05 | 6.06E-05 | 2.62061 |
| GPRC5B | -0.58746 | 8.2645813 | -5.6654897 | 6.10E-08 | 5.50E-07 | 7.69135 |
| PVRL1 | -0.58719 | 4.3732014 | -5.0563997 | 1.09E-06 | 7.15E-06 | 4.90972 |
| AMPH | -0.58719 | 3.9697302 | -3.6956605 | 0.000295 | 0.0010367 | -0.4122 |
| STMN4 | -0.58705 | 6.594592 | -3.4802576 | 0.000636 | 0.0020387 | -1.1282 |
| ADAM11 | -0.58678 | 2.6394809 | -3.7454743 | 0.000246 | 0.0008843 | -0.2413 |
| LRRC16B | -0.585 | 2.9009898 | -4.9018938 | 2.19E-06 | 1.33E-05 | 4.24078 |
| RP11-118K6.3 | -0.58406 | 1.8269659 | -5.5481483 | 1.08E-07 | 9.06E-07 | 7.13824 |
| LRRC4 | -0.58375 | 4.4759865 | -5.0711201 | 1.02E-06 | 6.72E-06 | 4.97426 |
| RP13-514E23.1 | -0.58366 | 2.8305649 | -4.4263817 | 1.70E-05 | 8.28E-05 | 2.28198 |
| RP11-275H4.1 | -0.58345 | 1.4711352 | -3.2929818 | 0.001205 | 0.0035822 | -1.7204 |
| STAR | -0.58333 | 1.1845968 | -6.0640374 | 8.27E-09 | 9.27E-08 | 9.62755 |
| MYRF | -0.58292 | 4.4873543 | -2.3459679 | 0.020125 | 0.0416608 | -4.26 |
| GALNT9 | -0.58276 | 3.2277192 | -2.6875936 | 0.007908 | 0.018516 | -3.4338 |
| TMEFF2 | -0.58249 | 4.2020804 | -3.2047465 | 0.001613 | 0.0046418 | -1.9894 |
| SLC24A3 | -0.58229 | 4.3233527 | -4.3865937 | 2.01E-05 | 9.59E-05 | 2.12518 |
| AC005220.3 | -0.58201 | 1.0025697 | -6.5069174 | 8.17E-10 | 1.17E-08 | 11.8768 |
| LDHD | -0.58103 | 3.4536823 | -5.5917902 | 8.75E-08 | 7.55E-07 | 7.34303 |
| OTOG | -0.58009 | 0.7377828 | -4.2911662 | 2.97E-05 | 0.0001355 | 1.75371 |
| PNMAL2 | -0.57993 | 3.3016658 | -3.7564628 | 0.000236 | 0.0008526 | -0.2034 |
| PLCB1 | -0.57991 | 4.5271987 | -4.6066308 | 7.97E-06 | 4.23E-05 | 3.00629 |
| AQP6 | -0.57953 | 1.7538248 | -5.7586907 | 3.85E-08 | 3.64E-07 | 8.13628 |
| FAM196A | -0.57953 | 1.7211098 | -4.1693845 | 4.85E-05 | 0.0002092 | 1.28921 |
| PEG3 | -0.57923 | 4.2694898 | -2.8629672 | 0.004722 | 0.0118074 | -2.9697 |
| OLIG2 | -0.57918 | 7.070906 | -2.6154137 | 0.009709 | 0.0222143 | -3.617 |
| HMGCS1 | -0.57915 | 6.0994982 | -4.7857069 | 3.67E-06 | 2.11E-05 | 3.74802 |
| PON1 | -0.57907 | 1.1403197 | -5.2786964 | 3.91E-07 | 2.86E-06 | 5.89881 |
| AL022344.7 | -0.5786 | 0.9139165 | -5.4766113 | 1.53E-07 | 1.23E-06 | 6.80496 |
| CA11 | -0.57848 | 5.8296932 | -3.2804541 | 0.001256 | 0.0037167 | -1.7589 |
| PREX2 | -0.57827 | 3.0883113 | -5.2935379 | 3.65E-07 | 2.69E-06 | 5.96594 |
| FGF12 | -0.57807 | 4.5112508 | -3.1271166 | 0.002075 | 0.0057763 | -2.2207 |
| PRKCZ | -0.57805 | 5.0659799 | -3.7343556 | 0.000256 | 0.0009166 | -0.2796 |
| RP1-153P14.3 | -0.57798 | 0.749057 | -5.5128119 | 1.28E-07 | 1.05E-06 | 6.97324 |
| CDH9 | -0.57717 | 1.2324079 | -3.8339555 | 0.000177 | 0.0006613 | 0.06681 |
| PIK3R1 | -0.57703 | 6.2992351 | -4.78563 | 3.67E-06 | 2.11E-05 | 3.7477 |
| PON3 | -0.57582 | 1.305827 | -5.501207 | 1.36E-07 | 1.11E-06 | 6.91921 |
| DIRAS2 | -0.57428 | 3.7302924 | -2.9470793 | 0.003656 | 0.0094347 | -2.7377 |
| SBP1 | -0.57401 | 1.0079493 | -3.7532025 | 0.000239 | 0.0008619 | -0.2147 |
| CPAMD8 | -0.57309 | 1.6504605 | -4.0415117 | 8.02E-05 | 0.0003278 | 0.81323 |
| RP11-1134I14.8 | -0.57303 | 2.3361689 | -6.66921 | 3.42E-10 | 5.42E-09 | 12.725 |
| RPRM | -0.57191 | 3.9297388 | -2.5440214 | 0.011844 | 0.0264167 | -3.7937 |
| LRRC37A4P | -0.57158 | 3.1991657 | -5.2275333 | 4.96E-07 | 3.54E-06 | 5.66843 |
| ENDOU | -0.57131 | 1.4739392 | -5.9973677 | 1.16E-08 | 1.25E-07 | 9.29765 |
| MAP7 | -0.57079 | 4.111161 | -3.1920261 | 0.001682 | 0.0048095 | -2.0276 |
| DAGLA | -0.57031 | 3.5166205 | -7.8211136 | 5.17E-13 | 1.81E-11 | 19.0668 |
| TMEM145 | -0.57024 | 4.5948869 | -4.6167362 | 7.63E-06 | 4.07E-05 | 3.04756 |
| SLC6A7 | -0.56996 | 1.2333277 | -2.9234207 | 0.003931 | 0.0100475 | -2.8035 |
| KCNF1 | -0.56974 | 3.7350656 | -3.1257666 | 0.002084 | 0.0057982 | -2.2247 |
| RP11-482M8.3 | -0.56967 | 1.0221837 | -5.6056139 | 8.18E-08 | 7.12E-07 | 7.40812 |
| MPP2 | -0.56925 | 4.212088 | -6.3540697 | 1.84E-09 | 2.44E-08 | 11.0895 |
| LINC00641 | -0.56792 | 4.3474808 | -5.7214943 | 4.63E-08 | 4.30E-07 | 7.95813 |
| RP11-527D7.1 | -0.56785 | 1.1669344 | -4.4237001 | 1.72E-05 | 8.36E-05 | 2.27138 |
| GDNF-AS1 | -0.56774 | 2.0610525 | -3.4476987 | 0.000712 | 0.0022457 | -1.2332 |
| GLT1D1 | -0.56768 | 1.7237609 | -3.0817331 | 0.002399 | 0.0065423 | -2.3536 |
| MGAT3 | -0.56764 | 4.0714465 | -5.1886244 | 5.95E-07 | 4.16E-06 | 5.49432 |
| PAIP2B | -0.56755 | 3.3773504 | -3.4667122 | 0.000666 | 0.0021236 | -1.172 |
| SYT14 | -0.56742 | 1.538482 | -5.9094539 | 1.81E-08 | 1.86E-07 | 8.86626 |
| TGFBR3L | -0.56707 | 1.7785633 | -2.6710317 | 0.008293 | 0.0193298 | -3.4762 |
| XYLT1 | -0.56653 | 3.009182 | -4.7129169 | 5.04E-06 | 2.79E-05 | 3.4439 |
| LPO | -0.56634 | 0.8847134 | -5.8141905 | 2.92E-08 | 2.84E-07 | 8.40355 |
| TRHDE-AS1 | -0.56627 | 0.9972241 | -4.8004087 | 3.44E-06 | 1.99E-05 | 3.80988 |
| BCRP2 | -0.56612 | 2.4854848 | -4.9223886 | 2.00E-06 | 1.23E-05 | 4.32862 |
| NEBL | -0.56547 | 5.0636128 | -3.8221427 | 0.000185 | 0.0006876 | 0.02532 |
| FAM201A | -0.56539 | 1.598608 | -4.2440534 | 3.59E-05 | 0.0001605 | 1.57273 |
| CHL1-AS2 | -0.56528 | 1.9448885 | -3.5855064 | 0.000439 | 0.0014742 | -0.7829 |
| AP003026.1 | -0.56519 | 0.9754293 | -3.7570382 | 0.000236 | 0.0008509 | -0.2014 |
| NKD1 | -0.56518 | 3.6320826 | -3.7002252 | 0.00029 | 0.0010219 | -0.3966 |
| CCT7P2 | -0.56403 | 0.6814296 | -6.7470149 | 2.24E-10 | 3.76E-09 | 13.1359 |
| LDOC1 | -0.56391 | 5.4128719 | -5.002431 | 1.40E-06 | 8.92E-06 | 4.67431 |
| PRCD | -0.56388 | 3.0632026 | -3.5578781 | 0.000484 | 0.0016046 | -0.8744 |
| FGFBP3 | -0.56298 | 3.6298801 | -3.3839788 | 0.000886 | 0.0027276 | -1.4362 |
| TRPC3 | -0.56279 | 1.3847804 | -5.5425228 | 1.11E-07 | 9.26E-07 | 7.11192 |
| LINC00643 | -0.56256 | 2.6810739 | -3.131675 | 0.002045 | 0.0057014 | -2.2073 |
| FOXG1 | -0.5621 | 4.761832 | -2.9309994 | 0.003841 | 0.0098451 | -2.7825 |
| GRIN2B | -0.5614 | 1.1771909 | -3.927859 | 0.000124 | 0.0004828 | 0.40045 |
| RYR2 | -0.56138 | 1.6450395 | -3.2639679 | 0.001327 | 0.0039024 | -1.8095 |
| GPIHBP1 | -0.55978 | 2.7977848 | -3.1503435 | 0.001926 | 0.0054028 | -2.152 |
| RP11-75C9.1 | -0.55963 | 2.7878816 | -4.3304366 | 2.53E-05 | 0.0001175 | 1.90578 |
| SLC25A23 | -0.559 | 6.6545959 | -7.3828374 | 6.49E-12 | 1.68E-10 | 16.5921 |
| CDH8 | -0.55885 | 2.3082801 | -3.4251257 | 0.000769 | 0.0024033 | -1.3055 |
| MADD | -0.55872 | 5.5194799 | -7.0977921 | 3.24E-11 | 6.95E-10 | 15.0218 |
| MT-ND4 | -0.55867 | 14.128217 | -6.8787658 | 1.09E-10 | 2.00E-09 | 13.838 |
| SLC26A4-AS1 | -0.55842 | 1.8239701 | -2.3832004 | 0.01826 | 0.0383419 | -4.175 |
| COL26A1 | -0.55834 | 1.7885823 | -3.7936631 | 0.000206 | 0.0007547 | -0.0743 |
| SREBF2 | -0.55802 | 6.0047873 | -6.8619057 | 1.20E-10 | 2.18E-09 | 13.7477 |
| THSD4 | -0.55801 | 2.0193625 | -4.5985673 | 8.25E-06 | 4.36E-05 | 2.9734 |
| FAT3 | -0.55797 | 3.3508431 | -4.2551569 | 3.44E-05 | 0.0001542 | 1.61524 |
| MB | -0.55686 | 1.4326741 | -4.5104579 | 1.20E-05 | 6.08E-05 | 2.61701 |
| DLGAP1-AS4 | -0.55657 | 1.0221595 | -3.4094446 | 0.000812 | 0.0025222 | -1.3555 |
| P2RX5 | -0.55655 | 1.7557277 | -3.8211015 | 0.000186 | 0.0006897 | 0.02167 |
| RP11-797H7.1 | -0.55645 | 2.1420563 | -4.9857689 | 1.51E-06 | 9.53E-06 | 4.602 |
| JAKMIP3 | -0.5563 | 1.9455631 | -4.6595342 | 6.35E-06 | 3.44E-05 | 3.22314 |
| TTPA | -0.55629 | 1.5966118 | -4.2090901 | 4.14E-05 | 0.0001818 | 1.43947 |
| CYP46A1 | -0.55624 | 4.1045162 | -3.4754554 | 0.000646 | 0.0020679 | -1.1437 |
| RP4-719C8.1 | -0.55616 | 1.3614842 | -4.8000769 | 3.44E-06 | 1.99E-05 | 3.80848 |
| APCDD1 | -0.55611 | 6.2387607 | -4.6994592 | 5.34E-06 | 2.94E-05 | 3.38807 |
| GLUD1 | -0.55604 | 7.8911689 | -4.6744725 | 5.95E-06 | 3.25E-05 | 3.28473 |
| CHN1 | -0.55547 | 6.5399844 | -2.9492032 | 0.003632 | 0.0093872 | -2.7317 |
| FAM167A | -0.55547 | 4.9060933 | -3.7741336 | 0.000221 | 0.0008046 | -0.1422 |
| KIAA1549L | -0.55452 | 3.2341932 | -4.6431357 | 6.81E-06 | 3.67E-05 | 3.15572 |
| AC006449.1 | -0.55438 | 5.0984801 | -4.0601599 | 7.45E-05 | 0.0003066 | 0.88189 |
| HSF2BP | -0.55407 | 2.0676053 | -5.0344534 | 1.21E-06 | 7.83E-06 | 4.81377 |
| PYGM | -0.55292 | 2.6497582 | -3.2103537 | 0.001584 | 0.0045666 | -1.9725 |
| FRMPD2P1 | -0.55266 | 1.4229597 | -3.6157632 | 0.000394 | 0.0013364 | -0.682 |
| SLC25A27 | -0.55175 | 4.5872158 | -4.6914578 | 5.53E-06 | 3.04E-05 | 3.35493 |
| ESRRG | -0.55171 | 1.7221394 | -5.454775 | 1.70E-07 | 1.35E-06 | 6.70383 |
| DHRS2 | -0.55144 | 1.6087781 | -2.8578551 | 0.004795 | 0.0119685 | -2.9836 |
| SCG2 | -0.55115 | 5.8077415 | -2.7363367 | 0.006869 | 0.0163795 | -3.3075 |
| MTATP6P1 | -0.55075 | 11.480799 | -6.325151 | 2.14E-09 | 2.80E-08 | 10.9418 |
| PELI3 | -0.55032 | 3.6301095 | -6.0556797 | 8.63E-09 | 9.64E-08 | 9.58607 |
| TMEM257 | -0.54869 | 1.9499714 | -5.1100959 | 8.55E-07 | 5.72E-06 | 5.14579 |
| FAM153B | -0.54825 | 1.2238202 | -3.2776445 | 0.001268 | 0.003748 | -1.7676 |
| HCN1 | -0.54722 | 1.3106409 | -3.0239774 | 0.002879 | 0.0076505 | -2.5202 |
| DCTN1-AS1 | -0.5472 | 1.2382561 | -4.0184754 | 8.77E-05 | 0.0003548 | 0.72878 |
| FAM171A2 | -0.54664 | 3.9592454 | -4.768731 | 3.95E-06 | 2.25E-05 | 3.67678 |
| HBQ1 | -0.54643 | 1.2676931 | -4.1580854 | 5.07E-05 | 0.0002177 | 1.24667 |
| PDE4A | -0.54574 | 3.4732624 | -4.931005 | 1.93E-06 | 1.19E-05 | 4.36563 |
| ATRNL1 | -0.54527 | 3.6840518 | -3.7067091 | 0.000283 | 0.0010014 | -0.3744 |
| MT-ND4L | -0.54447 | 13.816072 | -5.947036 | 1.50E-08 | 1.57E-07 | 9.05017 |
| NDUFA6-AS1 | -0.54413 | 3.9281939 | -6.0043652 | 1.12E-08 | 1.21E-07 | 9.33217 |
| IGSF21 | -0.54349 | 4.9989293 | -4.3352055 | 2.48E-05 | 0.0001155 | 1.92433 |
| CNTN1 | -0.54336 | 6.5501197 | -2.7403937 | 0.006789 | 0.0162057 | -3.2969 |
| ANXA3 | -0.5432 | 1.674801 | -4.4019386 | 1.88E-05 | 9.06E-05 | 2.18552 |
| MT-ATP6 | -0.54299 | 14.448667 | -6.8528039 | 1.26E-10 | 2.27E-09 | 13.699 |
| POU6F1 | -0.54298 | 4.7104226 | -7.021042 | 4.97E-11 | 1.01E-09 | 14.6047 |
| GNAI1 | -0.54258 | 5.1286138 | -4.1657622 | 4.92E-05 | 0.0002118 | 1.27556 |
| OTUD7A | -0.54244 | 2.6208455 | -5.1149358 | 8.36E-07 | 5.61E-06 | 5.16716 |
| RP11-439C15.4 | -0.54206 | 3.3854679 | -2.9379856 | 0.00376 | 0.009664 | -2.763 |
| FAM222A-AS1 | -0.54195 | 0.6466531 | -5.6792488 | 5.70E-08 | 5.19E-07 | 7.75673 |
| MYRIP | -0.54164 | 2.84998 | -3.9047406 | 0.000136 | 0.0005221 | 0.31769 |
| LINC00086 | -0.54137 | 2.8308233 | -4.0934294 | 6.54E-05 | 0.000273 | 1.00502 |
| HHATL | -0.54136 | 4.6705 | -2.3175705 | 0.021658 | 0.0443775 | -4.3239 |
| SFTPC | -0.54135 | 2.3105876 | -3.7985944 | 0.000202 | 0.0007428 | -0.0571 |
| RAB3IP | -0.5411 | 5.0529826 | -4.0145337 | 8.90E-05 | 0.0003594 | 0.71437 |
| RGS20 | -0.54017 | 2.9942519 | -3.1366254 | 0.002013 | 0.0056196 | -2.1926 |
| RALYL | -0.54002 | 2.7716944 | -2.7687263 | 0.006249 | 0.0150762 | -3.2224 |
| RXFP1 | -0.53973 | 1.1491048 | -3.714067 | 0.000276 | 0.0009776 | -0.3493 |
| GRIP1 | -0.53917 | 1.6833109 | -4.8224047 | 3.12E-06 | 1.83E-05 | 3.90269 |
| PSTPIP1 | -0.53896 | 3.4250649 | -3.6886404 | 0.000303 | 0.00106 | -0.4361 |
| GDAP1 | -0.53885 | 4.8942539 | -5.5478904 | 1.08E-07 | 9.07E-07 | 7.13703 |
| ELAVL4 | -0.53884 | 4.0691039 | -2.9963699 | 0.003139 | 0.0082455 | -2.5989 |
| INSM2 | -0.53877 | 0.8838181 | -4.8730662 | 2.49E-06 | 1.50E-05 | 4.11768 |
| DPP6 | -0.53865 | 5.867957 | -4.5215914 | 1.14E-05 | 5.82E-05 | 2.66174 |
| FRY | -0.53822 | 4.4933582 | -5.5820615 | 9.17E-08 | 7.86E-07 | 7.29728 |
| SPARCL1 | -0.53794 | 10.563375 | -4.2258551 | 3.87E-05 | 0.0001713 | 1.50325 |
| TUBA4A | -0.53773 | 4.8487116 | -2.4234499 | 0.016417 | 0.0349461 | -4.0817 |
| MMP17 | -0.53766 | 3.6571143 | -3.1162157 | 0.002149 | 0.0059542 | -2.2528 |
| VAMP2 | -0.53717 | 7.4965363 | -5.4957911 | 1.39E-07 | 1.13E-06 | 6.89402 |
| KCNS1 | -0.53707 | 1.1945489 | -2.8119008 | 0.005501 | 0.013497 | -3.1076 |
| PGBD5 | -0.5361 | 4.0934522 | -4.7353404 | 4.57E-06 | 2.56E-05 | 3.53721 |
| MT-ATP8 | -0.53523 | 14.899716 | -4.9057992 | 2.16E-06 | 1.32E-05 | 4.25749 |
| FAM155B | -0.53494 | 1.6476993 | -6.1846902 | 4.44E-09 | 5.32E-08 | 10.2305 |
| ZNF385A | -0.53471 | 6.0673672 | -4.3924761 | 1.96E-05 | 9.39E-05 | 2.14829 |
| DAO | -0.5346 | 1.2291499 | -2.8261223 | 0.005273 | 0.013014 | -3.0694 |
| ALDH1L2 | -0.53439 | 2.5062704 | -4.6996911 | 5.34E-06 | 2.94E-05 | 3.38903 |
| MAPT-AS1 | -0.53426 | 2.6987222 | -3.4520883 | 0.000701 | 0.0022187 | -1.2191 |
| ZNF423 | -0.53353 | 3.9349426 | -4.8608451 | 2.63E-06 | 1.57E-05 | 4.06567 |
| RASGRF2 | -0.53322 | 2.5537605 | -3.4165827 | 0.000792 | 0.0024648 | -1.3327 |
| EHD3 | -0.53303 | 4.5576911 | -4.9251339 | 1.98E-06 | 1.22E-05 | 4.34041 |
| LPHN1 | -0.5326 | 5.4338147 | -6.1245764 | 6.06E-09 | 7.03E-08 | 9.92914 |
| GRID1 | -0.53253 | 4.5804152 | -4.9791776 | 1.55E-06 | 9.77E-06 | 4.57345 |
| 11-Mar | -0.53214 | 1.2523564 | -3.5267532 | 0.00054 | 0.0017664 | -0.9768 |
| RP11-713P17.3 | -0.53211 | 2.9302625 | -4.3080179 | 2.77E-05 | 0.0001275 | 1.81883 |
| FGF9 | -0.5316 | 1.7624237 | -4.2369016 | 3.70E-05 | 0.0001647 | 1.5454 |
| LARGE | -0.53102 | 4.1186517 | -5.9821466 | 1.26E-08 | 1.34E-07 | 9.22267 |
| SORCS3 | -0.53015 | 2.8205927 | -3.8630058 | 0.000159 | 0.0006002 | 0.16931 |
| PTGDR2 | -0.52842 | 1.2918706 | -5.3773556 | 2.45E-07 | 1.88E-06 | 6.34757 |
| RAB31 | -0.52834 | 7.2667057 | -4.4271734 | 1.70E-05 | 8.26E-05 | 2.28512 |
| BEST1 | -0.52764 | 4.0200744 | -4.4415266 | 1.60E-05 | 7.84E-05 | 2.34197 |
| NTRK3 | -0.52763 | 5.1147039 | -4.8791642 | 2.43E-06 | 1.46E-05 | 4.14368 |
| TDH | -0.52761 | 1.4544943 | -4.1837292 | 4.58E-05 | 0.0001987 | 1.34336 |
| FRMPD4 | -0.52741 | 1.1277325 | -3.4971052 | 0.000599 | 0.0019348 | -1.0735 |
| OPHN1 | -0.52677 | 4.4452008 | -3.3872357 | 0.000876 | 0.0026992 | -1.4259 |
| PPP1R1B | -0.5262 | 5.1625036 | -2.3964152 | 0.017636 | 0.0372013 | -4.1445 |
| HRK | -0.52612 | 1.8803907 | -4.7394626 | 4.49E-06 | 2.52E-05 | 3.5544 |
| NET1 | -0.52602 | 4.7734097 | -4.3535686 | 2.30E-05 | 0.0001083 | 1.99588 |
| PCDHGA3 | -0.52586 | 2.2219756 | -2.947685 | 0.003649 | 0.0094211 | -2.736 |
| AC003102.3 | -0.52574 | 1.0217629 | -7.5035145 | 3.26E-12 | 9.22E-11 | 17.2665 |
| TRHDE | -0.52564 | 1.0841071 | -3.639057 | 0.000362 | 0.0012444 | -0.6039 |
| GOT1 | -0.52541 | 5.3371076 | -3.9560989 | 0.000111 | 0.0004384 | 0.50211 |
| RP11-64B16.2 | -0.52446 | 2.6785734 | -2.9108714 | 0.004085 | 0.0103885 | -2.8383 |
| B4GALT6 | -0.52412 | 2.9559615 | -3.9954654 | 9.58E-05 | 0.0003833 | 0.64482 |
| ZMAT4 | -0.52311 | 1.4575616 | -3.5011195 | 0.000591 | 0.0019109 | -1.0605 |
| FCHSD2 | -0.52305 | 5.6890725 | -4.6460974 | 6.73E-06 | 3.63E-05 | 3.16788 |
| GHRHR | -0.5229 | 2.12218 | -3.8380853 | 0.000174 | 0.0006524 | 0.08134 |
| RASL10B | -0.52285 | 3.6276987 | -5.029478 | 1.23E-06 | 7.99E-06 | 4.79205 |
| HOGA1 | -0.52279 | 2.6166611 | -4.121788 | 5.85E-05 | 0.0002471 | 1.11063 |
| TMEM179 | -0.52276 | 3.8424028 | -2.4608457 | 0.014855 | 0.0321227 | -3.9938 |
| FGF11 | -0.52263 | 4.604813 | -3.7476332 | 0.000244 | 0.0008778 | -0.2339 |
| CMBL | -0.52242 | 4.6940706 | -4.7673698 | 3.97E-06 | 2.26E-05 | 3.67107 |
| NTRK2 | -0.52241 | 8.1579343 | -2.8906982 | 0.004343 | 0.0109787 | -2.8939 |
| AKR1C1 | -0.52178 | 3.2397476 | -2.6880871 | 0.007897 | 0.0184951 | -3.4325 |
| DNER | -0.52152 | 7.382894 | -3.8808336 | 0.000148 | 0.0005654 | 0.23253 |
| IL17D | -0.52055 | 6.7084158 | -3.6667416 | 0.000328 | 0.0011369 | -0.5104 |
| RP11-1081L13.4 | -0.52032 | 2.0546131 | -5.6497315 | 6.59E-08 | 5.90E-07 | 7.61661 |
| TTLL12 | -0.52028 | 4.5317437 | -6.0589255 | 8.49E-09 | 9.51E-08 | 9.60217 |
| CBX7 | -0.52007 | 4.8392598 | -4.4008564 | 1.89E-05 | 9.09E-05 | 2.18126 |
| PLCH2 | -0.51997 | 2.9491545 | -3.4597988 | 0.000683 | 0.0021684 | -1.1943 |
| CACNA1E | -0.51993 | 2.018054 | -3.8753479 | 0.000152 | 0.0005764 | 0.21305 |
| APBB1 | -0.51986 | 6.884784 | -6.0842961 | 7.45E-09 | 8.45E-08 | 9.72826 |
| VIP | -0.51978 | 1.2388979 | -3.0092396 | 0.003015 | 0.007962 | -2.5623 |
| CACNB4 | -0.51974 | 3.0017159 | -4.829059 | 3.03E-06 | 1.78E-05 | 3.93083 |
| RP1-130H16.18 | -0.51948 | 1.7111732 | -4.376309 | 2.10E-05 | 9.98E-05 | 2.08483 |
| LCAT | -0.51862 | 5.5346554 | -3.4308628 | 0.000754 | 0.0023626 | -1.2871 |
| HEY1 | -0.5184 | 6.7970556 | -3.2953719 | 0.001195 | 0.0035577 | -1.713 |
| DGCR10 | -0.51791 | 0.9965558 | -5.1970283 | 5.72E-07 | 4.01E-06 | 5.53185 |
| LRIG1 | -0.51741 | 7.6751193 | -3.5766447 | 0.000453 | 0.0015161 | -0.8123 |
| RP6-201G10.2 | -0.51584 | 2.1351187 | -3.9050158 | 0.000135 | 0.0005217 | 0.31867 |
| ELAVL3 | -0.51582 | 5.2457251 | -4.4891049 | 1.31E-05 | 6.59E-05 | 2.53145 |
| RPRML | -0.51569 | 1.5273929 | -2.6618087 | 0.008514 | 0.0197752 | -3.4998 |
| RP11-266K4.1 | -0.51567 | 2.1067801 | -4.2264901 | 3.86E-05 | 0.0001709 | 1.50567 |
| MT-ND5 | -0.51559 | 12.490346 | -5.5253249 | 1.21E-07 | 9.97E-07 | 7.03158 |
| RAI2 | -0.51558 | 2.8097839 | -5.9811867 | 1.26E-08 | 1.35E-07 | 9.21794 |
| KIF26A | -0.51553 | 2.1066957 | -3.605694 | 0.000408 | 0.0013802 | -0.7157 |
| GPR22 | -0.51537 | 1.3688198 | -3.0656911 | 0.002525 | 0.0068357 | -2.4002 |
| B3GALT2 | -0.51416 | 2.9925797 | -4.0760233 | 7.01E-05 | 0.0002899 | 0.9405 |
| ACVR1C | -0.51394 | 0.9632018 | -5.8490816 | 2.46E-08 | 2.44E-07 | 8.57244 |
| AP006621.1 | -0.51358 | 1.7797455 | -3.8963799 | 0.00014 | 0.0005369 | 0.28786 |
| HMX1 | -0.51346 | 2.863529 | -2.6223081 | 0.009522 | 0.0218365 | -3.5997 |
| BEX5 | -0.51283 | 3.4061924 | -2.3050581 | 0.022365 | 0.0455776 | -4.3519 |
| CCBL1 | -0.51209 | 4.3499899 | -5.7655109 | 3.73E-08 | 3.54E-07 | 8.16903 |
| SUSD4 | -0.51207 | 4.5628416 | -4.1959052 | 4.36E-05 | 0.0001903 | 1.38944 |
| ZDHHC11 | -0.51206 | 3.4963601 | -3.6923989 | 0.000299 | 0.0010481 | -0.4233 |
| HS3ST4 | -0.51106 | 1.9980651 | -2.9336662 | 0.00381 | 0.0097794 | -2.7751 |
| C11orf87 | -0.511 | 1.4492507 | -2.6515253 | 0.008767 | 0.0203025 | -3.5259 |
| SATB1 | -0.5105 | 5.3376994 | -4.2974773 | 2.89E-05 | 0.0001324 | 1.77807 |
| CBFA2T3 | -0.5102 | 2.0119259 | -5.0350306 | 1.20E-06 | 7.81E-06 | 4.81629 |
| DRP2 | -0.51015 | 2.5831753 | -4.6251598 | 7.36E-06 | 3.94E-05 | 3.08202 |
| EPB41L4A-AS1 | -0.50942 | 5.6973743 | -4.8024119 | 3.41E-06 | 1.97E-05 | 3.81832 |
| DUSP8 | -0.50918 | 3.46602 | -4.2031549 | 4.23E-05 | 0.0001857 | 1.41693 |
| PPARGC1A | -0.50915 | 2.6297807 | -4.3173629 | 2.67E-05 | 0.0001233 | 1.85503 |
| PPM1H | -0.50851 | 2.8874247 | -4.5373744 | 1.07E-05 | 5.49E-05 | 2.72531 |
| HERC2P3 | -0.5083 | 4.9808822 | -3.4284355 | 0.000761 | 0.00238 | -1.2949 |
| RAB6B | -0.50812 | 6.0158245 | -4.2662524 | 3.28E-05 | 0.0001484 | 1.6578 |
| ANLN | -0.5073 | 5.0664725 | -2.4431048 | 0.015579 | 0.0334363 | -4.0357 |
| HAPLN4 | -0.50687 | 2.1005368 | -2.5607637 | 0.011309 | 0.025395 | -3.7527 |
| RP11-594N15.3 | -0.50686 | 1.2154366 | -6.9510967 | 7.32E-11 | 1.41E-09 | 14.2267 |
| RP11-284F21.7 | -0.50657 | 3.7242087 | -2.2922796 | 0.023109 | 0.0468842 | -4.3803 |
| MT-CO3 | -0.50653 | 14.235345 | -6.5954271 | 5.09E-10 | 7.71E-09 | 12.3378 |
| KIF3A | -0.50581 | 4.7020011 | -5.1807238 | 6.17E-07 | 4.29E-06 | 5.45908 |
| MAST3 | -0.50533 | 3.8402994 | -3.7951112 | 0.000205 | 0.0007511 | -0.0692 |
| MYH7B | -0.5047 | 1.9383129 | -5.0325268 | 1.22E-06 | 7.89E-06 | 4.80536 |
| RP11-551L14.1 | -0.50457 | 1.8803627 | -2.5495722 | 0.011664 | 0.0260751 | -3.7801 |
| CCKBR | -0.50436 | 1.5851791 | -2.6175341 | 0.009651 | 0.0220905 | -3.6117 |
| SEMA3G | -0.50419 | 2.4884738 | -4.5715765 | 9.25E-06 | 4.82E-05 | 2.86366 |
| RP13-238F13.5 | -0.50333 | 0.7683856 | -5.1673098 | 6.57E-07 | 4.54E-06 | 5.39934 |
| GUCY1B3 | -0.50325 | 3.8838032 | -3.4790222 | 0.000638 | 0.0020453 | -1.1322 |
| CPEB3 | -0.50317 | 2.4402053 | -5.0634081 | 1.06E-06 | 6.94E-06 | 4.94043 |
| FOXRED2 | -0.50255 | 4.6368157 | -5.5864831 | 8.98E-08 | 7.72E-07 | 7.31806 |
| CHL1 | -0.50241 | 4.9942403 | -2.4344163 | 0.015944 | 0.0341192 | -4.0561 |
| AC140912.1 | -0.50234 | 1.2557955 | -4.3579788 | 2.26E-05 | 0.0001066 | 2.0131 |
| SASH1 | -0.50215 | 4.7241229 | -5.0645888 | 1.05E-06 | 6.91E-06 | 4.94561 |
| EPN2 | -0.50192 | 6.1719584 | -4.4413182 | 1.60E-05 | 7.85E-05 | 2.34114 |
| ISM1 | -0.50148 | 1.5743967 | -4.6231353 | 7.42E-06 | 3.97E-05 | 3.07374 |
| PHF21B | -0.50145 | 3.2094098 | -3.851656 | 0.000166 | 0.0006237 | 0.12919 |
| RPS6KA2 | -0.50142 | 4.5940714 | -5.8024905 | 3.10E-08 | 2.99E-07 | 8.34706 |
| MYO10 | -0.50096 | 6.0549356 | -4.9095241 | 2.12E-06 | 1.29E-05 | 4.27345 |
| ADAM22 | -0.50007 | 4.7184063 | -4.5899132 | 8.56E-06 | 4.51E-05 | 2.93816 |
| STX1A | -0.50002 | 4.4963771 | -2.5044031 | 0.013203 | 0.0290334 | -3.8898 |
