# Supplementary material for: MAGOH is correlated with poor prognosis and is essential for cell proliferation in lower-grade glioma
Source: Aging (Albany NY). 2023 Jun 30;15(12):5713–33. doi: 10.18632/aging.204823 (PMC10333088; doi:10.18632/aging.204823)
Supplement: Supplementary Table 4 [file aging-15-204823-s005.docx]

**Supplementary Table 4. Up-regulated DEGs in CGGA cohort.**

| **id** | **logFC** | **AveExpr** | **t** | **P.Value** | **adj.P.Val** | **B** |
| --- | --- | --- | --- | --- | --- | --- |
| MDFIC | 0.50067 | 3.0459409 | 3.62484 | 0.000381 | 0.0013003 | -0.6516 |
| VNN2 | 0.50112 | 1.3233495 | 5.04092 | 1.17E-06 | 7.62E-06 | 4.84203 |
| AK4 | 0.50124 | 3.7150139 | 3.71001 | 0.00028 | 0.0009904 | -0.3631 |
| OR52K3P | 0.50128 | 0.9083411 | 4.14104 | 5.42E-05 | 0.0002313 | 1.18267 |
| BMP1 | 0.50139 | 3.9321175 | 4.41878 | 1.76E-05 | 8.51E-05 | 2.25195 |
| RNASEH2A | 0.50144 | 4.3172637 | 4.87182 | 2.51E-06 | 1.50E-05 | 4.11238 |
| ZAP70 | 0.50174 | 1.3971897 | 4.71739 | 4.94E-06 | 2.74E-05 | 3.46249 |
| TMEM60 | 0.50187 | 4.2178608 | 7.51668 | 3.02E-12 | 8.70E-11 | 17.3404 |
| AKR7A2 | 0.50206 | 5.4270723 | 7.99926 | 1.81E-13 | 7.11E-12 | 20.0918 |
| MTCP1 | 0.50241 | 3.8215287 | 3.87761 | 0.00015 | 0.0005717 | 0.22107 |
| RASGRP4 | 0.50243 | 1.6149695 | 5.94346 | 1.53E-08 | 1.60E-07 | 9.03265 |
| GSC | 0.50266 | 1.1744351 | 3.79748 | 0.000203 | 0.0007452 | -0.061 |
| CPSF3L | 0.50272 | 6.1961604 | 8.49812 | 9.17E-15 | 5.02E-13 | 23.0155 |
| SLC10A3 | 0.50289 | 2.9780469 | 7.76365 | 7.23E-13 | 2.41E-11 | 18.7384 |
| NBPF1 | 0.503 | 5.2675022 | 5.55985 | 1.02E-07 | 8.64E-07 | 7.19304 |
| SASS6 | 0.50303 | 1.6027134 | 9.33172 | 5.41E-17 | 4.93E-15 | 28.0502 |
| PHYKPL | 0.50306 | 4.7233393 | 6.78834 | 1.79E-10 | 3.09E-09 | 13.3553 |
| ODF2 | 0.5032 | 4.3938072 | 6.35091 | 1.87E-09 | 2.48E-08 | 11.0733 |
| AC092675.3 | 0.50323 | 1.3080549 | 4.46971 | 1.42E-05 | 7.09E-05 | 2.454 |
| CCL20 | 0.50333 | 0.6215762 | 4.32556 | 2.58E-05 | 0.0001196 | 1.88682 |
| TNIP2 | 0.50341 | 4.6207868 | 5.0255 | 1.26E-06 | 8.12E-06 | 4.77473 |
| CD96 | 0.50344 | 0.7822071 | 4.51978 | 1.15E-05 | 5.86E-05 | 2.65447 |
| ZNF146 | 0.50374 | 5.2502315 | 6.53024 | 7.21E-10 | 1.05E-08 | 11.9979 |
| ROPN1L | 0.50386 | 0.8145057 | 4.25134 | 3.49E-05 | 0.0001564 | 1.60061 |
| SP110 | 0.50391 | 3.9614358 | 4.68283 | 5.74E-06 | 3.14E-05 | 3.31924 |
| NRROS | 0.50401 | 2.4337224 | 4.48942 | 1.31E-05 | 6.59E-05 | 2.5327 |
| FAM110C | 0.50408 | 1.0005112 | 3.83153 | 0.000179 | 0.0006663 | 0.0583 |
| RP11-206L10.9 | 0.50411 | 1.9606866 | 6.4696 | 9.96E-10 | 1.40E-08 | 11.6836 |
| SPIN4 | 0.50412 | 1.6744859 | 6.99591 | 5.71E-11 | 1.14E-09 | 14.4686 |
| CBWD6 | 0.50416 | 4.4042198 | 6.15187 | 5.27E-09 | 6.19E-08 | 10.0657 |
| DTX2 | 0.50419 | 2.9417586 | 6.21986 | 3.70E-09 | 4.54E-08 | 10.4077 |
| ZNF789 | 0.5042 | 3.719215 | 6.61071 | 4.69E-10 | 7.16E-09 | 12.4178 |
| ZNF568 | 0.50427 | 3.181395 | 7.7072 | 1.00E-12 | 3.24E-11 | 18.4169 |
| AP2S1 | 0.50438 | 6.1837957 | 5.96267 | 1.39E-08 | 1.46E-07 | 9.12688 |
| GNB4 | 0.50451 | 4.4474478 | 3.89069 | 0.000143 | 0.0005476 | 0.26757 |
| GINS4 | 0.50456 | 1.9595858 | 5.36073 | 2.65E-07 | 2.02E-06 | 6.27154 |
| AC016629.7 | 0.50468 | 1.2985774 | 2.79295 | 0.005819 | 0.0141519 | -3.1582 |
| CYB5RL | 0.5047 | 2.0802183 | 7.14997 | 2.42E-11 | 5.38E-10 | 15.3068 |
| RP11-876N24.4 | 0.50482 | 1.0058903 | 5.91368 | 1.78E-08 | 1.83E-07 | 8.88691 |
| HOXA11 | 0.50484 | 0.5208452 | 4.68713 | 5.64E-06 | 3.09E-05 | 3.33704 |
| PNKP | 0.50485 | 4.1906334 | 7.79233 | 6.11E-13 | 2.08E-11 | 18.9021 |
| KIF16B | 0.50503 | 2.6253338 | 4.93625 | 1.88E-06 | 1.16E-05 | 4.38818 |
| CARD11 | 0.50514 | 2.0902047 | 5.36788 | 2.57E-07 | 1.96E-06 | 6.30419 |
| GPC3 | 0.50532 | 1.4147057 | 3.3718 | 0.000924 | 0.0028349 | -1.4746 |
| NEDD4 | 0.50538 | 2.4511151 | 4.52075 | 1.15E-05 | 5.84E-05 | 2.65835 |
| TPM3 | 0.50539 | 7.2675425 | 8.05517 | 1.30E-13 | 5.36E-12 | 20.4157 |
| ZC3H12A | 0.5054 | 1.7744147 | 4.28512 | 3.04E-05 | 0.0001386 | 1.73039 |
| CTD-2085J24.4 | 0.50545 | 1.3212642 | 6.42063 | 1.29E-09 | 1.76E-08 | 11.4309 |
| RP11-6O2.3 | 0.50563 | 0.8498788 | 7.15243 | 2.39E-11 | 5.33E-10 | 15.3203 |
| SLC39A7 | 0.50565 | 6.096265 | 8.22175 | 4.83E-14 | 2.19E-12 | 21.3865 |
| IRAK4 | 0.50568 | 3.1293386 | 6.69746 | 2.93E-10 | 4.74E-09 | 12.8739 |
| CTNNAL1 | 0.5057 | 3.608572 | 6.52679 | 7.35E-10 | 1.07E-08 | 11.98 |
| HEATR3 | 0.506 | 3.1112805 | 7.18957 | 1.94E-11 | 4.42E-10 | 15.5239 |
| TMEM43 | 0.50603 | 5.0258503 | 6.78529 | 1.82E-10 | 3.13E-09 | 13.3391 |
| EGLN2 | 0.50608 | 5.3060254 | 7.6573 | 1.34E-12 | 4.20E-11 | 18.1337 |
| RP11-253M7.1 | 0.50616 | 0.6155728 | 6.198 | 4.15E-09 | 5.03E-08 | 10.2975 |
| FOLR2 | 0.50619 | 4.9653774 | 3.00652 | 0.003041 | 0.0080237 | -2.57 |
| SH3PXD2B | 0.50628 | 3.8602167 | 5.05604 | 1.09E-06 | 7.15E-06 | 4.90814 |
| CHAF1B | 0.50645 | 1.3118415 | 5.08803 | 9.46E-07 | 6.27E-06 | 5.04856 |
| SLC8B1 | 0.50659 | 3.7726019 | 5.37394 | 2.49E-07 | 1.91E-06 | 6.33195 |
| ZNF468 | 0.50683 | 2.3050885 | 5.43824 | 1.84E-07 | 1.45E-06 | 6.62746 |
| NLRC4 | 0.50683 | 1.143208 | 6.97613 | 6.38E-11 | 1.25E-09 | 14.3617 |
| RP11-481H12.1 | 0.50696 | 0.5357799 | 5.4698 | 1.58E-07 | 1.27E-06 | 6.77337 |
| RP11-148K1.12 | 0.50701 | 1.3315946 | 6.08879 | 7.29E-09 | 8.29E-08 | 9.75061 |
| AC004067.5 | 0.50725 | 0.7758679 | 6.1944 | 4.23E-09 | 5.11E-08 | 10.2793 |
| LRRC40 | 0.50742 | 3.5667469 | 8.14234 | 7.76E-14 | 3.38E-12 | 20.9226 |
| LINC00665 | 0.50747 | 4.0580363 | 7.19558 | 1.87E-11 | 4.29E-10 | 15.5569 |
| STAT3 | 0.5077 | 5.896513 | 6.03817 | 9.44E-09 | 1.04E-07 | 9.49927 |
| AC018766.4 | 0.50773 | 2.1466252 | 5.42067 | 2.00E-07 | 1.56E-06 | 6.54645 |
| SNRPG | 0.50782 | 6.3715914 | 6.89385 | 1.00E-10 | 1.86E-09 | 13.9188 |
| RP11-71N10.1 | 0.50794 | 1.9560239 | 3.63674 | 0.000365 | 0.001253 | -0.6117 |
| RP11-689K5.3 | 0.50817 | 0.8541453 | 4.53499 | 1.08E-05 | 5.54E-05 | 2.71568 |
| FAM136A | 0.50819 | 4.2746567 | 10.2003 | 2.21E-19 | 3.43E-17 | 33.4498 |
| SRPR | 0.50828 | 5.18028 | 9.20419 | 1.20E-16 | 9.98E-15 | 27.2694 |
| METTL1 | 0.50831 | 4.1144915 | 3.07062 | 0.002486 | 0.0067433 | -2.3859 |
| FUT4 | 0.50845 | 1.3289942 | 7.11868 | 2.88E-11 | 6.25E-10 | 15.1358 |
| PRPF31 | 0.50848 | 5.2719209 | 8.35342 | 2.19E-14 | 1.08E-12 | 22.1598 |
| RP11-733O18.1 | 0.50851 | 1.0326792 | 5.68185 | 5.63E-08 | 5.13E-07 | 7.7691 |
| NKX2-5 | 0.50853 | 0.5644687 | 3.97691 | 0.000103 | 0.0004085 | 0.5774 |
| PAPPA2 | 0.5086 | 0.8723752 | 5.07852 | 9.88E-07 | 6.52E-06 | 5.00674 |
| EFHC2 | 0.50861 | 1.4689234 | 3.96655 | 0.000107 | 0.0004234 | 0.53987 |
| NOX4 | 0.50863 | 1.2538035 | 3.85833 | 0.000162 | 0.0006094 | 0.15276 |
| NCAPG2 | 0.50875 | 3.3158768 | 5.26858 | 4.10E-07 | 2.98E-06 | 5.85312 |
| RFC3 | 0.50877 | 2.8381137 | 6.03608 | 9.54E-09 | 1.05E-07 | 9.48892 |
| HGFAC | 0.50895 | 1.7733331 | 4.5419 | 1.05E-05 | 5.40E-05 | 2.74357 |
| HNRNPR | 0.50896 | 6.5445957 | 8.54738 | 6.80E-15 | 3.82E-13 | 23.3081 |
| GPR141 | 0.509 | 0.733612 | 4.49416 | 1.28E-05 | 6.47E-05 | 2.55169 |
| KCNE4 | 0.50917 | 2.2306797 | 3.20783 | 0.001597 | 0.004599 | -1.9801 |
| CDCA4 | 0.5092 | 2.0765544 | 5.68235 | 5.62E-08 | 5.12E-07 | 7.77148 |
| UBASH3B | 0.50921 | 1.9084362 | 5.27919 | 3.90E-07 | 2.85E-06 | 5.90105 |
| ABCB4 | 0.50938 | 1.2666868 | 5.33412 | 3.01E-07 | 2.27E-06 | 6.15018 |
| RP11-385F5.5 | 0.50943 | 1.4239164 | 5.14061 | 7.43E-07 | 5.06E-06 | 5.28075 |
| HAAO | 0.50947 | 2.0487153 | 5.06788 | 1.04E-06 | 6.81E-06 | 4.96005 |
| CCDC11 | 0.50952 | 1.6049735 | 5.74176 | 4.19E-08 | 3.93E-07 | 8.05511 |
| CLEC11A | 0.50954 | 3.097687 | 5.74614 | 4.10E-08 | 3.85E-07 | 8.07606 |
| CITED4 | 0.50977 | 2.1654105 | 4.35443 | 2.29E-05 | 0.0001079 | 1.99926 |
| LRCH4 | 0.50991 | 5.1537203 | 5.98106 | 1.26E-08 | 1.35E-07 | 9.21731 |
| GMPR | 0.50991 | 3.5604374 | 2.79825 | 0.005728 | 0.0139722 | -3.1441 |
| MLF1 | 0.5101 | 4.6823203 | 4.84789 | 2.79E-06 | 1.65E-05 | 4.01062 |
| NUDT19 | 0.51019 | 2.3278713 | 7.93713 | 2.61E-13 | 9.81E-12 | 19.7332 |
| ACTA2 | 0.51025 | 5.4283302 | 2.58772 | 0.010492 | 0.0237455 | -3.6861 |
| POU4F1 | 0.51039 | 0.573725 | 4.34558 | 2.38E-05 | 0.0001112 | 1.96472 |
| LDHAP4 | 0.51043 | 1.4410841 | 4.48472 | 1.34E-05 | 6.70E-05 | 2.51393 |
| HELZ2 | 0.51047 | 3.0679382 | 4.32717 | 2.56E-05 | 0.0001189 | 1.89311 |
| ZNF816 | 0.51065 | 2.0309305 | 6.9341 | 8.04E-11 | 1.53E-09 | 14.1351 |
| CREG1 | 0.51073 | 5.5923182 | 4.46081 | 1.48E-05 | 7.30E-05 | 2.41856 |
| AC092295.7 | 0.51074 | 2.1186292 | 7.32204 | 9.17E-12 | 2.28E-10 | 16.2545 |
| RIPK2 | 0.51079 | 4.0626047 | 4.74137 | 4.45E-06 | 2.50E-05 | 3.56235 |
| CST7 | 0.51084 | 1.2041233 | 4.43067 | 1.67E-05 | 8.15E-05 | 2.29895 |
| CASC1 | 0.51091 | 1.4165264 | 5.14843 | 7.16E-07 | 4.90E-06 | 5.31543 |
| DYNLRB2 | 0.51096 | 2.4476167 | 3.10125 | 0.002255 | 0.0062047 | -2.2967 |
| SH3BP5-AS1 | 0.51102 | 2.4769731 | 4.88332 | 2.38E-06 | 1.44E-05 | 4.16138 |
| CENPN | 0.51104 | 2.939405 | 4.36226 | 2.22E-05 | 0.000105 | 2.02982 |
| RLF | 0.51105 | 2.8682448 | 8.06987 | 1.19E-13 | 4.97E-12 | 20.501 |
| SRRM1 | 0.51115 | 5.5366151 | 6.69937 | 2.90E-10 | 4.71E-09 | 12.8839 |
| HES1 | 0.51117 | 4.1051341 | 3.97 | 0.000106 | 0.0004182 | 0.55235 |
| VANGL1 | 0.51153 | 1.6154812 | 5.98142 | 1.26E-08 | 1.35E-07 | 9.21908 |
| TRIM73 | 0.51157 | 1.8636585 | 5.26455 | 4.18E-07 | 3.03E-06 | 5.83495 |
| LINC00085 | 0.51165 | 3.1938125 | 5.69101 | 5.38E-08 | 4.93E-07 | 7.8127 |
| RP11-480C22.1 | 0.51167 | 2.1556218 | 3.60701 | 0.000406 | 0.0013745 | -0.7113 |
| EVC2 | 0.51174 | 0.6004892 | 6.77953 | 1.88E-10 | 3.22E-09 | 13.3084 |
| FREM2 | 0.51187 | 1.2182599 | 4.05679 | 7.55E-05 | 0.0003101 | 0.86947 |
| NID1 | 0.51222 | 3.9105904 | 2.9514 | 0.003608 | 0.0093328 | -2.7256 |
| ZNF326 | 0.51243 | 4.4399746 | 7.79431 | 6.04E-13 | 2.07E-11 | 18.9134 |
| KIRREL | 0.51253 | 2.8707898 | 3.99053 | 9.77E-05 | 0.0003899 | 0.62686 |
| PROM1 | 0.51261 | 2.5553675 | 3.56288 | 0.000476 | 0.0015797 | -0.8579 |
| RP11-415J8.3 | 0.5128 | 1.7547615 | 6.21583 | 3.78E-09 | 4.63E-08 | 10.3873 |
| WTIP | 0.51283 | 2.3022524 | 5.0988 | 9.00E-07 | 5.99E-06 | 5.09598 |
| PLXDC1 | 0.51294 | 4.0779974 | 3.87768 | 0.00015 | 0.0005717 | 0.22132 |
| PINLYP | 0.51297 | 2.6916579 | 2.79778 | 0.005736 | 0.013985 | -3.1453 |
| CD226 | 0.51306 | 1.0396814 | 7.21888 | 1.64E-11 | 3.81E-10 | 15.6849 |
| SSFA2 | 0.51319 | 6.0063355 | 5.7098 | 4.91E-08 | 4.54E-07 | 7.90229 |
| AGRN | 0.51323 | 5.8520417 | 5.34542 | 2.85E-07 | 2.16E-06 | 6.20168 |
| COL9A1 | 0.51323 | 2.1855047 | 2.84338 | 0.005008 | 0.0124341 | -3.0229 |
| DYNLT3 | 0.51335 | 3.6909728 | 3.06059 | 0.002566 | 0.0069293 | -2.4149 |
| LINC00960 | 0.51348 | 1.5763891 | 3.62402 | 0.000382 | 0.0013036 | -0.6544 |
| HOXA2 | 0.5136 | 0.5661464 | 3.76407 | 0.00023 | 0.0008323 | -0.1771 |
| SDC2 | 0.51363 | 5.3115485 | 3.47743 | 0.000642 | 0.0020554 | -1.1373 |
| RP11-63M22.2 | 0.51376 | 1.2364192 | 2.5791 | 0.010748 | 0.0242442 | -3.7074 |
| DMAP1 | 0.51389 | 5.3414572 | 9.83896 | 2.22E-18 | 2.74E-16 | 31.1875 |
| GS1-358P8.4 | 0.514 | 2.6272305 | 4.11304 | 6.06E-05 | 0.0002548 | 1.07799 |
| KHNYN | 0.514 | 2.8826402 | 4.49358 | 1.29E-05 | 6.48E-05 | 2.54937 |
| SAMD13 | 0.51423 | 2.1720595 | 4.78593 | 3.66E-06 | 2.11E-05 | 3.74894 |
| OSBPL3 | 0.51434 | 3.3401085 | 3.92694 | 0.000125 | 0.0004843 | 0.39715 |
| CHPF | 0.51439 | 5.6729264 | 4.40641 | 1.85E-05 | 8.91E-05 | 2.20313 |
| SMO | 0.51445 | 3.8293771 | 4.54062 | 1.05E-05 | 5.43E-05 | 2.73841 |
| SEC61A1 | 0.51474 | 6.654414 | 5.97243 | 1.32E-08 | 1.40E-07 | 9.17485 |
| SPRY2 | 0.51474 | 5.1639032 | 4.5871 | 8.66E-06 | 4.55E-05 | 2.92673 |
| ACAP1 | 0.51476 | 2.2636471 | 4.9137 | 2.08E-06 | 1.27E-05 | 4.29135 |
| EFNA1 | 0.51502 | 4.5007479 | 4.52651 | 1.12E-05 | 5.71E-05 | 2.68155 |
| LRWD1 | 0.51506 | 4.6481725 | 7.58186 | 2.07E-12 | 6.21E-11 | 17.7072 |
| PIRT | 0.51507 | 2.1230711 | 2.63245 | 0.009253 | 0.0212862 | -3.5742 |
| CCT6A | 0.51518 | 6.7128757 | 6.34991 | 1.88E-09 | 2.49E-08 | 11.0682 |
| HIPK1 | 0.51551 | 4.7583477 | 7.25144 | 1.37E-11 | 3.24E-10 | 15.8643 |
| ATPIF1 | 0.5156 | 7.1799444 | 6.45678 | 1.07E-09 | 1.49E-08 | 11.6173 |
| HHLA3 | 0.51565 | 4.5592308 | 7.17345 | 2.12E-11 | 4.81E-10 | 15.4355 |
| SNAI2 | 0.51576 | 1.8222192 | 3.50777 | 0.000577 | 0.0018733 | -1.0388 |
| DUSP1 | 0.51589 | 5.7130655 | 2.71854 | 0.007233 | 0.0171452 | -3.3539 |
| CLCC1 | 0.51601 | 4.2237894 | 9.4004 | 3.52E-17 | 3.36E-15 | 28.472 |
| IMPACT | 0.51609 | 4.4097866 | 5.20572 | 5.49E-07 | 3.87E-06 | 5.57069 |
| ACCS | 0.51617 | 3.2626497 | 4.14575 | 5.32E-05 | 0.0002277 | 1.20034 |
| SMCO4 | 0.51618 | 3.8417572 | 4.84001 | 2.89E-06 | 1.70E-05 | 3.9772 |
| CYP27B1 | 0.5162 | 1.1179155 | 3.36347 | 0.00095 | 0.0029064 | -1.5008 |
| WIPF1 | 0.51621 | 4.7998614 | 5.75063 | 4.01E-08 | 3.78E-07 | 8.09759 |
| TTC25 | 0.51624 | 2.1051659 | 5.97287 | 1.32E-08 | 1.40E-07 | 9.17702 |
| CD2AP | 0.51626 | 2.56084 | 8.06355 | 1.24E-13 | 5.13E-12 | 20.4643 |
| TOE1 | 0.51637 | 3.0286724 | 10.3032 | 1.15E-19 | 1.92E-17 | 34.0971 |
| DUSP14 | 0.51647 | 3.9642448 | 7.14987 | 2.42E-11 | 5.38E-10 | 15.3063 |
| HYI | 0.5167 | 4.8714785 | 6.00505 | 1.12E-08 | 1.21E-07 | 9.33557 |
| CROCCP2 | 0.51701 | 4.0473236 | 7.17453 | 2.11E-11 | 4.79E-10 | 15.4414 |
| RSBN1 | 0.51702 | 2.9646585 | 7.01892 | 5.03E-11 | 1.02E-09 | 14.5932 |
| STK17A | 0.51706 | 4.0671208 | 4.86298 | 2.61E-06 | 1.56E-05 | 4.07475 |
| NME1-NME2 | 0.51727 | 8.2089659 | 7.40742 | 5.64E-12 | 1.49E-10 | 16.729 |
| PIK3R6 | 0.5176 | 1.100789 | 6.5514 | 6.44E-10 | 9.51E-09 | 12.1081 |
| MIR155HG | 0.51766 | 0.5634828 | 7.43641 | 4.78E-12 | 1.29E-10 | 16.8908 |
| CCDC8 | 0.5178 | 1.2540357 | 3.64693 | 0.000352 | 0.001213 | -0.5774 |
| PSMC4 | 0.5178 | 5.9680222 | 9.93607 | 1.20E-18 | 1.56E-16 | 31.7934 |
| SOCS6 | 0.51796 | 4.3390093 | 5.55182 | 1.06E-07 | 8.92E-07 | 7.15541 |
| ENTPD1 | 0.51807 | 3.974389 | 5.86382 | 2.28E-08 | 2.28E-07 | 8.644 |
| ABHD15 | 0.51815 | 2.3048206 | 7.48927 | 3.53E-12 | 9.93E-11 | 17.1866 |
| FOXJ3 | 0.51825 | 4.2423388 | 7.22142 | 1.62E-11 | 3.77E-10 | 15.6989 |
| RP11-84C10.2 | 0.51847 | 0.878098 | 3.9745 | 0.000104 | 0.0004118 | 0.56867 |
| LRRCC1 | 0.5185 | 3.8150875 | 5.91092 | 1.80E-08 | 1.85E-07 | 8.8734 |
| HAS3 | 0.51882 | 1.2743915 | 6.06876 | 8.07E-09 | 9.07E-08 | 9.65102 |
| PLA2G5 | 0.51886 | 2.0927559 | 2.464 | 0.014729 | 0.0318946 | -3.9863 |
| LINC00662 | 0.51893 | 3.8999732 | 6.2092 | 3.91E-09 | 4.78E-08 | 10.3539 |
| TRIM34 | 0.51912 | 2.1245566 | 4.67736 | 5.88E-06 | 3.21E-05 | 3.29663 |
| SFPQ | 0.51938 | 7.5875143 | 7.12432 | 2.79E-11 | 6.10E-10 | 15.1666 |
| TMEM194A | 0.51953 | 2.8610973 | 6.0934 | 7.11E-09 | 8.11E-08 | 9.77359 |
| AC002454.1 | 0.51959 | 0.5374798 | 4.59296 | 8.45E-06 | 4.46E-05 | 2.95057 |
| NEDD9 | 0.51963 | 3.5861256 | 3.92876 | 0.000124 | 0.0004813 | 0.40367 |
| HBEGF | 0.51978 | 3.5175344 | 3.72028 | 0.00027 | 0.0009585 | -0.328 |
| ATP1A1 | 0.51985 | 7.1078239 | 5.26874 | 4.09E-07 | 2.98E-06 | 5.85387 |
| SDHDP6 | 0.51997 | 0.9446914 | 5.53773 | 1.14E-07 | 9.44E-07 | 7.08951 |
| CNR1 | 0.51997 | 3.3755639 | 3.08009 | 0.002412 | 0.0065735 | -2.3584 |
| HTATIP2 | 0.52002 | 3.5007671 | 3.62153 | 0.000386 | 0.0013134 | -0.6627 |
| RP11-206L10.11 | 0.52049 | 3.42844 | 6.16741 | 4.86E-09 | 5.77E-08 | 10.1437 |
| PRAME | 0.5207 | 0.56518 | 2.64994 | 0.008806 | 0.0203784 | -3.5299 |
| NAGA | 0.52086 | 3.9555739 | 5.60907 | 8.04E-08 | 7.03E-07 | 7.4244 |
| RP11-495P10.2 | 0.521 | 2.3971279 | 2.41685 | 0.016707 | 0.0354925 | -4.0971 |
| FRMD8 | 0.52102 | 3.7859568 | 6.56323 | 6.05E-10 | 8.98E-09 | 12.1697 |
| H2AFZ | 0.52114 | 7.1822723 | 6.18619 | 4.41E-09 | 5.29E-08 | 10.2381 |
| TTC23 | 0.52129 | 3.6063383 | 6.65187 | 3.75E-10 | 5.88E-09 | 12.6337 |
| FAM117A | 0.52154 | 2.5522827 | 7.78137 | 6.52E-13 | 2.21E-11 | 18.8395 |
| C1orf112 | 0.52172 | 2.2076912 | 6.7785 | 1.89E-10 | 3.24E-09 | 13.303 |
| CD8A | 0.52174 | 1.2877905 | 4.33623 | 2.47E-05 | 0.000115 | 1.92832 |
| LIPE-AS1 | 0.52206 | 3.1912024 | 4.03715 | 8.15E-05 | 0.000333 | 0.79722 |
| CDR2 | 0.5224 | 3.054458 | 5.75562 | 3.91E-08 | 3.69E-07 | 8.12152 |
| RAD9A | 0.5224 | 3.4848869 | 6.91009 | 9.18E-11 | 1.72E-09 | 14.006 |
| HOXC9 | 0.52246 | 0.5035421 | 4.13911 | 5.46E-05 | 0.0002328 | 1.17544 |
| NUCB1 | 0.52266 | 6.84396 | 6.32556 | 2.13E-09 | 2.79E-08 | 10.9439 |
| PPIE | 0.52275 | 5.3344021 | 10.8161 | 4.20E-21 | 1.02E-18 | 37.3462 |
| ARHGAP27 | 0.52304 | 3.3746194 | 6.16515 | 4.92E-09 | 5.83E-08 | 10.1323 |
| MYO1B | 0.52311 | 3.3403289 | 3.7034 | 0.000287 | 0.0010118 | -0.3858 |
| ATP5F1 | 0.52338 | 7.7266596 | 10.3093 | 1.10E-19 | 1.86E-17 | 34.1355 |
| EYA4 | 0.52342 | 0.9056167 | 3.93144 | 0.000122 | 0.0004773 | 0.41329 |
| TMEM53 | 0.52343 | 3.2659541 | 7.74338 | 8.13E-13 | 2.69E-11 | 18.6228 |
| CTD-3220F14.1 | 0.52355 | 1.0904511 | 4.16633 | 4.90E-05 | 0.0002115 | 1.27771 |
| ACAA2 | 0.52356 | 4.668108 | 4.92305 | 2.00E-06 | 1.23E-05 | 4.33145 |
| C6ORF165 | 0.52415 | 0.9030493 | 5.24357 | 4.61E-07 | 3.31E-06 | 5.74045 |
| C11orf82 | 0.52423 | 1.4684631 | 4.9636 | 1.66E-06 | 1.04E-05 | 4.50611 |
| ZFP36L1 | 0.52431 | 6.508333 | 4.04465 | 7.92E-05 | 0.0003243 | 0.82477 |
| AL592494.5 | 0.52441 | 1.3280727 | 6.64126 | 3.98E-10 | 6.17E-09 | 12.578 |
| EBNA1BP2 | 0.52445 | 5.6850069 | 10.5958 | 1.74E-20 | 3.42E-18 | 35.9469 |
| GUSB | 0.5245 | 4.8853982 | 6.38812 | 1.53E-09 | 2.07E-08 | 11.2639 |
| MCM10 | 0.52475 | 1.3649268 | 3.91733 | 0.000129 | 0.0005003 | 0.36272 |
| TMEM59 | 0.52476 | 7.2054967 | 8.63596 | 3.97E-15 | 2.37E-13 | 23.836 |
| PSMB2 | 0.52478 | 5.3937265 | 10.2694 | 1.42E-19 | 2.37E-17 | 33.8845 |
| ISLR | 0.52481 | 3.2267321 | 2.96038 | 0.003509 | 0.0091022 | -2.7004 |
| CDC25C | 0.52488 | 1.040391 | 4.89549 | 2.26E-06 | 1.37E-05 | 4.21339 |
| ZC3HAV1 | 0.52491 | 3.6553678 | 5.96628 | 1.36E-08 | 1.44E-07 | 9.14465 |
| CLEC4A | 0.52494 | 1.9665791 | 5.41627 | 2.04E-07 | 1.59E-06 | 6.52621 |
| H2AFJ | 0.5252 | 3.5608928 | 4.85607 | 2.69E-06 | 1.60E-05 | 4.04538 |
| ENY2 | 0.52528 | 5.9769594 | 8.44556 | 1.26E-14 | 6.65E-13 | 22.704 |
| CYP19A1 | 0.52534 | 1.1648293 | 4.25561 | 3.43E-05 | 0.0001541 | 1.61697 |
| AQP5 | 0.52544 | 0.8823445 | 3.15219 | 0.001914 | 0.0053788 | -2.1466 |
| TRAM1 | 0.52573 | 5.7703937 | 5.60745 | 8.11E-08 | 7.07E-07 | 7.41678 |
| TYW3 | 0.52576 | 3.3018557 | 10.0762 | 4.90E-19 | 6.96E-17 | 32.6703 |
| PEPD | 0.52597 | 5.8375556 | 7.71987 | 9.32E-13 | 3.03E-11 | 18.489 |
| POC1A | 0.5264 | 1.9701301 | 5.13693 | 7.55E-07 | 5.14E-06 | 5.26445 |
| FEM1C | 0.52652 | 3.7162888 | 6.61807 | 4.50E-10 | 6.90E-09 | 12.4564 |
| PDIA3 | 0.5268 | 7.3750607 | 7.06857 | 3.81E-11 | 8.03E-10 | 14.8627 |
| YRDC | 0.52688 | 3.5861207 | 8.71138 | 2.51E-15 | 1.57E-13 | 24.2871 |
| DNAJB4 | 0.52704 | 4.4295608 | 7.80919 | 5.54E-13 | 1.92E-11 | 18.9985 |
| FAM86B3P | 0.52725 | 1.0696467 | 5.43803 | 1.84E-07 | 1.45E-06 | 6.62649 |
| FRZB | 0.5275 | 2.9204871 | 4.03913 | 8.09E-05 | 0.0003305 | 0.80448 |
| RP11-798M19.6 | 0.52773 | 3.7947187 | 5.33386 | 3.01E-07 | 2.27E-06 | 6.14901 |
| AC005550.4 | 0.52776 | 0.627789 | 3.10008 | 0.002263 | 0.006223 | -2.3001 |
| PLA2G15 | 0.52781 | 4.0761375 | 7.47692 | 3.79E-12 | 1.05E-10 | 17.1174 |
| AC009005.2 | 0.52798 | 1.4369629 | 4.74848 | 4.32E-06 | 2.44E-05 | 3.59203 |
| FAM154B | 0.52805 | 1.9428074 | 6.01798 | 1.05E-08 | 1.14E-07 | 9.39939 |
| EXO1 | 0.52853 | 1.3881455 | 4.28269 | 3.07E-05 | 0.0001399 | 1.72101 |
| TMEM156 | 0.52858 | 1.8877631 | 4.6453 | 6.75E-06 | 3.64E-05 | 3.16462 |
| MCM8 | 0.52865 | 1.8837645 | 5.88613 | 2.04E-08 | 2.07E-07 | 8.7525 |
| KCNMB1 | 0.52885 | 1.6193766 | 4.43364 | 1.65E-05 | 8.06E-05 | 2.3107 |
| SOX9 | 0.52888 | 6.014655 | 4.23782 | 3.68E-05 | 0.0001642 | 1.54889 |
| AL357673.1 | 0.52906 | 2.0983617 | 9.42636 | 2.99E-17 | 2.90E-15 | 28.6318 |
| C20orf26 | 0.52916 | 1.8061574 | 4.30118 | 2.85E-05 | 0.0001307 | 1.79238 |
| TMOD3 | 0.5292 | 3.6178408 | 8.08093 | 1.12E-13 | 4.74E-12 | 20.5652 |
| EML4 | 0.52945 | 3.5601082 | 7.50558 | 3.22E-12 | 9.13E-11 | 17.2781 |
| C11orf88 | 0.52952 | 0.5407739 | 3.78042 | 0.000216 | 0.0007876 | -0.1204 |
| WTAP | 0.5296 | 5.8411777 | 6.04861 | 8.95E-09 | 9.93E-08 | 9.55101 |
| RP11-293A21.1 | 0.52963 | 1.7078763 | 3.84784 | 0.000168 | 0.0006318 | 0.11572 |
| MEIS3P1 | 0.52966 | 1.8497973 | 4.5204 | 1.15E-05 | 5.85E-05 | 2.65696 |
| ZNHIT6 | 0.5297 | 3.937898 | 10.1995 | 2.23E-19 | 3.43E-17 | 33.4446 |
| MAGI3 | 0.52979 | 3.0519805 | 5.07863 | 9.87E-07 | 6.52E-06 | 5.00724 |
| GZMK | 0.52983 | 0.7147997 | 5.12401 | 8.02E-07 | 5.41E-06 | 5.20728 |
| BIRC3 | 0.52999 | 1.4690632 | 4.39497 | 1.94E-05 | 9.31E-05 | 2.15811 |
| ISL2 | 0.53026 | 0.6200465 | 4.84999 | 2.76E-06 | 1.64E-05 | 4.01956 |
| PTBP1 | 0.53029 | 6.3163387 | 5.27362 | 4.00E-07 | 2.92E-06 | 5.87588 |
| SLC2A3 | 0.53034 | 3.9002273 | 3.51679 | 0.000559 | 0.0018211 | -1.0094 |
| CALR | 0.53041 | 8.4994357 | 6.52245 | 7.52E-10 | 1.09E-08 | 11.9574 |
| MST1P2 | 0.53063 | 2.1805082 | 4.63984 | 6.91E-06 | 3.72E-05 | 3.1422 |
| PPP1R14B | 0.53068 | 6.856641 | 4.83201 | 2.99E-06 | 1.76E-05 | 3.94332 |
| FAM177B | 0.53115 | 0.7361767 | 6.12935 | 5.91E-09 | 6.88E-08 | 9.953 |
| CDCP1 | 0.53122 | 1.0520161 | 5.96699 | 1.36E-08 | 1.44E-07 | 9.14811 |
| IRAK1 | 0.53128 | 5.6171593 | 7.13257 | 2.67E-11 | 5.83E-10 | 15.2116 |
| TMEM165 | 0.53152 | 5.4695948 | 5.19999 | 5.64E-07 | 3.96E-06 | 5.5451 |
| RND3 | 0.53153 | 3.9833589 | 3.474 | 0.00065 | 0.002076 | -1.1484 |
| KIAA1551 | 0.53153 | 3.4660823 | 8.46251 | 1.14E-14 | 6.08E-13 | 22.8044 |
| PIK3CG | 0.53157 | 1.3934479 | 5.66682 | 6.06E-08 | 5.47E-07 | 7.69768 |
| MAP1LC3C | 0.53191 | 0.5853591 | 4.72693 | 4.74E-06 | 2.65E-05 | 3.50219 |
| SMIM10 | 0.532 | 2.3119034 | 3.94132 | 0.000118 | 0.0004615 | 0.44882 |
| HLA-DRB6 | 0.53204 | 0.7111427 | 4.18664 | 4.52E-05 | 0.0001967 | 1.35437 |
| RPL5 | 0.53218 | 9.4531809 | 6.25517 | 3.08E-09 | 3.87E-08 | 10.5862 |
| PTGR1 | 0.53236 | 4.2429649 | 4.43209 | 1.66E-05 | 8.11E-05 | 2.30458 |
| ZNF566 | 0.53241 | 2.833979 | 8.0364 | 1.46E-13 | 5.88E-12 | 20.3069 |
| CCDC34 | 0.53271 | 3.133533 | 6.25876 | 3.02E-09 | 3.81E-08 | 10.6043 |
| TSPAN12 | 0.53271 | 4.1725147 | 3.13818 | 0.002003 | 0.005594 | -2.188 |
| TMEM64 | 0.53279 | 2.9501339 | 6.33544 | 2.02E-09 | 2.67E-08 | 10.9943 |
| ZFAND6 | 0.53295 | 6.0442043 | 8.25669 | 3.92E-14 | 1.80E-12 | 21.5912 |
| NSUN4 | 0.53337 | 3.4482881 | 9.72003 | 4.71E-18 | 5.48E-16 | 30.4477 |
| REM1 | 0.53356 | 1.4189033 | 3.04238 | 0.002718 | 0.0072777 | -2.4674 |
| LPXN | 0.53373 | 3.4815993 | 6.60337 | 4.87E-10 | 7.41E-09 | 12.3794 |
| ICOSLG | 0.53381 | 3.1276989 | 3.6153 | 0.000394 | 0.0013384 | -0.6836 |
| ALDH1A3 | 0.53399 | 1.0036952 | 3.74904 | 0.000243 | 0.0008736 | -0.229 |
| CTC-297N7.5 | 0.53406 | 1.016707 | 4.44905 | 1.55E-05 | 7.63E-05 | 2.37183 |
| SLC35D2 | 0.53412 | 4.0489118 | 5.88227 | 2.08E-08 | 2.11E-07 | 8.7337 |
| PTHLH | 0.53416 | 2.3152417 | 3.74 | 0.000251 | 0.0008999 | -0.2602 |
| DSN1 | 0.53425 | 2.9392045 | 6.99023 | 5.90E-11 | 1.17E-09 | 14.4379 |
| TLR6 | 0.53427 | 1.481986 | 5.84124 | 2.55E-08 | 2.53E-07 | 8.53445 |
| WDHD1 | 0.53443 | 1.8664309 | 6.26268 | 2.96E-09 | 3.74E-08 | 10.6242 |
| PBXIP1 | 0.53453 | 6.5621888 | 4.75584 | 4.18E-06 | 2.37E-05 | 3.62282 |
| NFATC4 | 0.53474 | 3.8166985 | 3.94921 | 0.000114 | 0.0004492 | 0.47724 |
| HAUS1 | 0.53485 | 4.2088882 | 6.42517 | 1.26E-09 | 1.73E-08 | 11.4543 |
| PLK4 | 0.53493 | 2.1672206 | 4.531 | 1.10E-05 | 5.62E-05 | 2.69962 |
| DKK1 | 0.53512 | 1.2792864 | 3.42882 | 0.00076 | 0.0023774 | -1.2937 |
| CCRL2 | 0.53514 | 1.7394699 | 4.90384 | 2.17E-06 | 1.32E-05 | 4.24911 |
| HLA-DOB | 0.53517 | 0.9682554 | 5.14583 | 7.25E-07 | 4.96E-06 | 5.30389 |
| EMC1 | 0.5352 | 4.5501566 | 7.78139 | 6.52E-13 | 2.21E-11 | 18.8396 |
| EPHA2 | 0.53546 | 1.880031 | 4.26627 | 3.28E-05 | 0.0001484 | 1.65787 |
| SPATA18 | 0.53559 | 0.9750361 | 3.84619 | 0.000169 | 0.0006355 | 0.10991 |
| C19orf18 | 0.53563 | 1.0796089 | 6.69067 | 3.04E-10 | 4.90E-09 | 12.838 |
| SH2B3 | 0.53563 | 3.318688 | 5.5469 | 1.09E-07 | 9.11E-07 | 7.13238 |
| MMRN1 | 0.5357 | 1.1721909 | 4.94189 | 1.83E-06 | 1.14E-05 | 4.41246 |
| CCDC181 | 0.53591 | 2.8163408 | 7.13073 | 2.70E-11 | 5.89E-10 | 15.2016 |
| RP11-65J3.1 | 0.53598 | 2.5147767 | 4.03132 | 8.34E-05 | 0.0003396 | 0.77582 |
| IGHV3-30 | 0.536 | 0.6570723 | 2.88277 | 0.004448 | 0.0112163 | -2.9156 |
| HHEX | 0.53635 | 2.0441615 | 5.18817 | 5.96E-07 | 4.16E-06 | 5.49228 |
| GGACT | 0.53639 | 1.6923641 | 4.40416 | 1.87E-05 | 8.99E-05 | 2.19426 |
| APOBEC3F | 0.53679 | 1.3274177 | 5.50492 | 1.33E-07 | 1.09E-06 | 6.93647 |
| SFT2D2 | 0.53683 | 3.1647589 | 6.48411 | 9.23E-10 | 1.31E-08 | 11.7586 |
| MFAP4 | 0.53713 | 3.7535071 | 3.21038 | 0.001584 | 0.0045666 | -1.9724 |
| DOCK11 | 0.53726 | 2.4035795 | 5.8392 | 2.58E-08 | 2.55E-07 | 8.52454 |
| HOXB-AS1 | 0.5373 | 0.5479741 | 4.4732 | 1.40E-05 | 7.00E-05 | 2.46793 |
| ANGPTL4 | 0.53758 | 3.4941788 | 2.80105 | 0.005681 | 0.013871 | -3.1366 |
| RASSF5 | 0.53771 | 2.338055 | 4.77234 | 3.89E-06 | 2.22E-05 | 3.6919 |
| PRICKLE3 | 0.53788 | 1.3915259 | 5.12175 | 8.10E-07 | 5.46E-06 | 5.19726 |
| SYTL2 | 0.53801 | 4.7637567 | 3.63072 | 0.000373 | 0.0012759 | -0.6319 |
| ZNF181 | 0.53804 | 4.0640285 | 6.91633 | 8.87E-11 | 1.67E-09 | 14.0396 |
| RP11-512H23.2 | 0.53821 | 0.6430024 | 5.90666 | 1.84E-08 | 1.89E-07 | 8.85261 |
| NPHP4 | 0.53823 | 3.0013673 | 7.02152 | 4.96E-11 | 1.01E-09 | 14.6073 |
| C1orf123 | 0.53841 | 5.9597739 | 10.2614 | 1.50E-19 | 2.48E-17 | 33.8337 |
| AC017048.3 | 0.53842 | 1.2627374 | 3.66478 | 0.00033 | 0.0011438 | -0.517 |
| KCNMB3 | 0.53889 | 1.3552377 | 6.90895 | 9.24E-11 | 1.73E-09 | 13.9999 |
| PSMC2 | 0.539 | 6.1968337 | 10.2439 | 1.68E-19 | 2.72E-17 | 33.7239 |
| HEBP1 | 0.53937 | 4.584867 | 4.01967 | 8.73E-05 | 0.0003534 | 0.73315 |
| IPO4 | 0.53975 | 3.6022316 | 6.08762 | 7.33E-09 | 8.33E-08 | 9.74482 |
| B3GNT7 | 0.54011 | 1.2437004 | 4.83768 | 2.92E-06 | 1.72E-05 | 3.96736 |
| C11orf70 | 0.54042 | 1.669828 | 3.98345 | 0.0001 | 0.0003994 | 0.60113 |
| STK33 | 0.54044 | 3.8909413 | 4.71895 | 4.91E-06 | 2.73E-05 | 3.46897 |
| FKBP7 | 0.54056 | 2.9939359 | 5.33869 | 2.95E-07 | 2.22E-06 | 6.17098 |
| GTF2B | 0.54057 | 4.6925145 | 9.94941 | 1.10E-18 | 1.47E-16 | 31.8768 |
| ALDH3B1 | 0.54102 | 2.4492358 | 5.38799 | 2.33E-07 | 1.80E-06 | 6.3963 |
| MPZL2 | 0.54108 | 1.176528 | 4.10511 | 6.25E-05 | 0.0002621 | 1.04846 |
| HAUS8 | 0.54133 | 2.7875807 | 6.00811 | 1.10E-08 | 1.19E-07 | 9.35065 |
| PTOV1-AS1 | 0.54149 | 2.7378733 | 7.90041 | 3.24E-13 | 1.20E-11 | 19.5218 |
| HSD17B3 | 0.54162 | 1.7466052 | 4.40357 | 1.87E-05 | 9.01E-05 | 2.19193 |
| CENPE | 0.54165 | 1.1649806 | 4.57566 | 9.09E-06 | 4.75E-05 | 2.88021 |
| IGLV2-11 | 0.54184 | 0.7410592 | 2.75035 | 0.006594 | 0.0157999 | -3.2708 |
| LILRA1 | 0.54203 | 1.6518942 | 5.15015 | 7.11E-07 | 4.87E-06 | 5.32309 |
| TRPM2 | 0.54207 | 2.6137046 | 4.4729 | 1.40E-05 | 7.01E-05 | 2.46673 |
| CTD-2341M24.1 | 0.54221 | 0.9394108 | 5.89439 | 1.96E-08 | 2.00E-07 | 8.79279 |
| TMEM91 | 0.54247 | 3.1702021 | 5.57982 | 9.27E-08 | 7.93E-07 | 7.28676 |
| LRRC46 | 0.54252 | 1.4990657 | 5.44121 | 1.81E-07 | 1.43E-06 | 6.64114 |
| LILRA2 | 0.54271 | 2.3254438 | 3.95055 | 0.000114 | 0.000447 | 0.48208 |
| TWIST1 | 0.54284 | 1.4490413 | 3.95672 | 0.000111 | 0.0004375 | 0.50435 |
| CAPNS1 | 0.54294 | 7.6277143 | 7.36897 | 7.02E-12 | 1.81E-10 | 16.515 |
| KIF18B | 0.54302 | 2.0344599 | 3.42976 | 0.000757 | 0.0023701 | -1.2907 |
| FANCC | 0.54331 | 2.0379824 | 5.68686 | 5.49E-08 | 5.02E-07 | 7.79293 |
| PRR16 | 0.54338 | 1.4542597 | 4.199 | 4.31E-05 | 0.0001884 | 1.40117 |
| IRAK2 | 0.54338 | 1.8623341 | 5.21038 | 5.38E-07 | 3.79E-06 | 5.59155 |
| DUSP23 | 0.54362 | 3.9618696 | 4.80338 | 3.39E-06 | 1.97E-05 | 3.82241 |
| LCA5L | 0.54366 | 1.3562458 | 6.07968 | 7.63E-09 | 8.64E-08 | 9.70528 |
| CPSF4 | 0.54368 | 5.4529448 | 6.62229 | 4.40E-10 | 6.76E-09 | 12.4785 |
| AKAP12 | 0.54369 | 4.1343461 | 3.0921 | 0.002321 | 0.0063594 | -2.3234 |
| THBS3 | 0.5437 | 4.2001344 | 5.77759 | 3.51E-08 | 3.35E-07 | 8.22712 |
| MGAT4A | 0.54375 | 3.7362526 | 7.0391 | 4.50E-11 | 9.27E-10 | 14.7026 |
| FMNL1 | 0.54375 | 3.6659064 | 4.21751 | 4.00E-05 | 0.0001765 | 1.47147 |
| MR1 | 0.54376 | 3.0476681 | 5.00269 | 1.39E-06 | 8.91E-06 | 4.67545 |
| TXNDC5 | 0.54388 | 5.0290555 | 6.69489 | 2.97E-10 | 4.81E-09 | 12.8603 |
| ANKRD35 | 0.54391 | 1.677823 | 4.25226 | 3.48E-05 | 0.0001559 | 1.60412 |
| KLHL6 | 0.54396 | 1.5538069 | 5.39142 | 2.30E-07 | 1.77E-06 | 6.41202 |
| PLOD3 | 0.54398 | 5.1484259 | 5.42239 | 1.98E-07 | 1.55E-06 | 6.55435 |
| XRCC6BP1 | 0.54429 | 2.198496 | 3.59578 | 0.000423 | 0.0014262 | -0.7487 |
| DESI2 | 0.54434 | 3.6192966 | 6.84749 | 1.30E-10 | 2.33E-09 | 13.6706 |
| TXN | 0.54449 | 6.7787136 | 7.71999 | 9.32E-13 | 3.03E-11 | 18.4896 |
| USP48 | 0.54484 | 4.5018726 | 9.98151 | 8.95E-19 | 1.23E-16 | 32.0775 |
| PLEKHO1 | 0.54488 | 5.594215 | 7.32045 | 9.25E-12 | 2.30E-10 | 16.2457 |
| EIF2B3 | 0.54496 | 4.0543531 | 9.7891 | 3.04E-18 | 3.64E-16 | 30.877 |
| IL10 | 0.5451 | 0.8372458 | 5.24053 | 4.67E-07 | 3.35E-06 | 5.72681 |
| RP11-169K16.7 | 0.54533 | 4.6052804 | 6.78583 | 1.81E-10 | 3.13E-09 | 13.3419 |
| ZNF473 | 0.5455 | 2.5750658 | 7.24061 | 1.45E-11 | 3.42E-10 | 15.8046 |
| TBC1D10C | 0.54557 | 1.3936069 | 5.9339 | 1.60E-08 | 1.67E-07 | 8.98579 |
| MORN3 | 0.54575 | 1.3320255 | 4.7425 | 4.43E-06 | 2.49E-05 | 3.56706 |
| PGM5 | 0.54585 | 2.5815885 | 4.43544 | 1.64E-05 | 8.02E-05 | 2.31784 |
| RP6-99M1.2 | 0.54586 | 1.0480903 | 5.67452 | 5.84E-08 | 5.29E-07 | 7.73424 |
| ARHGEF19 | 0.54599 | 1.2794961 | 5.37964 | 2.43E-07 | 1.87E-06 | 6.35801 |
| CSF2RB | 0.54612 | 1.3283491 | 6.16623 | 4.89E-09 | 5.80E-08 | 10.1378 |
| ATG4C | 0.54614 | 3.6225396 | 6.67492 | 3.31E-10 | 5.27E-09 | 12.755 |
| LINC00998 | 0.54617 | 5.9362591 | 6.17827 | 4.59E-09 | 5.48E-08 | 10.1982 |
| PHYH | 0.54638 | 4.1779258 | 5.16915 | 6.51E-07 | 4.50E-06 | 5.40754 |
| CRLF3 | 0.54657 | 2.8188818 | 9.93519 | 1.20E-18 | 1.56E-16 | 31.7879 |
| KIF11 | 0.54662 | 2.0452718 | 4.15616 | 5.11E-05 | 0.0002191 | 1.23943 |
| MRGPRF | 0.54666 | 1.6289051 | 4.75435 | 4.21E-06 | 2.38E-05 | 3.61656 |
| UBA2 | 0.54676 | 6.0122832 | 7.35685 | 7.53E-12 | 1.92E-10 | 16.4476 |
| AC091171.1 | 0.54684 | 1.6469176 | 3.13572 | 0.002019 | 0.0056347 | -2.1953 |
| NUP62 | 0.54704 | 4.7285102 | 7.03125 | 4.70E-11 | 9.63E-10 | 14.66 |
| RP5-864K19.4 | 0.54718 | 1.4105083 | 6.73747 | 2.36E-10 | 3.93E-09 | 13.0854 |
| IGLV3-19 | 0.54818 | 0.6220054 | 3.08369 | 0.002385 | 0.0065075 | -2.3479 |
| IL4R | 0.54829 | 3.194894 | 5.22791 | 4.96E-07 | 3.53E-06 | 5.67014 |
| DISP1 | 0.5483 | 2.6290047 | 5.68482 | 5.55E-08 | 5.07E-07 | 7.78321 |
| THAP3 | 0.54865 | 4.243291 | 9.3904 | 3.75E-17 | 3.55E-15 | 28.4105 |
| RHPN1-AS1 | 0.54867 | 0.8548438 | 5.87541 | 2.15E-08 | 2.17E-07 | 8.70031 |
| RPN2 | 0.54884 | 6.7007164 | 7.94698 | 2.47E-13 | 9.33E-12 | 19.7899 |
| KPNA6 | 0.5489 | 3.8148427 | 9.3329 | 5.37E-17 | 4.91E-15 | 28.0574 |
| EHD4 | 0.54904 | 3.0286798 | 4.92811 | 1.95E-06 | 1.20E-05 | 4.3532 |
| LIMS1 | 0.54913 | 4.8053875 | 5.42588 | 1.95E-07 | 1.53E-06 | 6.57046 |
| ADAM19 | 0.54915 | 1.9017277 | 4.86752 | 2.56E-06 | 1.53E-05 | 4.09405 |
| TMEM52B | 0.54925 | 1.6424681 | 4.98515 | 1.51E-06 | 9.55E-06 | 4.5993 |
| GPR132 | 0.54931 | 1.2891594 | 6.22053 | 3.69E-09 | 4.53E-08 | 10.411 |
| RUNX3 | 0.54953 | 1.2806444 | 5.87955 | 2.11E-08 | 2.13E-07 | 8.72048 |
| C19orf40 | 0.54954 | 1.375548 | 7.80848 | 5.56E-13 | 1.93E-11 | 18.9944 |
| FZD1 | 0.54975 | 2.7996159 | 6.07365 | 7.87E-09 | 8.87E-08 | 9.67533 |
| MEAF6 | 0.54986 | 5.8852963 | 10.3585 | 8.04E-20 | 1.39E-17 | 34.4458 |
| MAP3K14 | 0.54996 | 3.0777439 | 6.4162 | 1.32E-09 | 1.80E-08 | 11.4082 |
| ALG6 | 0.55006 | 2.9583516 | 7.09158 | 3.36E-11 | 7.18E-10 | 14.988 |
| BDKRB2 | 0.55037 | 0.8189083 | 4.985 | 1.51E-06 | 9.55E-06 | 4.59865 |
| GABRE | 0.5504 | 1.4606701 | 3.88488 | 0.000146 | 0.0005578 | 0.24693 |
| IL15RA | 0.55078 | 1.9605751 | 5.54226 | 1.11E-07 | 9.27E-07 | 7.11069 |
| FNDC1 | 0.5508 | 0.6929905 | 5.11882 | 8.21E-07 | 5.52E-06 | 5.18432 |
| C15orf65 | 0.55087 | 1.9696667 | 5.88202 | 2.08E-08 | 2.11E-07 | 8.73252 |
| ZNF585A | 0.55096 | 3.2557844 | 9.76117 | 3.63E-18 | 4.30E-16 | 30.7033 |
| CD40 | 0.55098 | 2.4063944 | 5.23005 | 4.91E-07 | 3.50E-06 | 5.67973 |
| CDK4 | 0.55117 | 7.1268284 | 3.11237 | 0.002176 | 0.0060191 | -2.2641 |
| TMTC2 | 0.55156 | 3.481629 | 3.9848 | 9.98E-05 | 0.0003976 | 0.60605 |
| RINL | 0.55165 | 2.1518359 | 5.1436 | 7.33E-07 | 5.00E-06 | 5.29403 |
| PTP4A2 | 0.55176 | 6.9699643 | 6.76405 | 2.04E-10 | 3.46E-09 | 13.2263 |
| ZCCHC9 | 0.55178 | 3.7652639 | 8.93197 | 6.47E-16 | 4.73E-14 | 25.6149 |
| TSPAN10 | 0.55192 | 1.5466535 | 7.12004 | 2.86E-11 | 6.21E-10 | 15.1432 |
| RP11-418J17.1 | 0.55208 | 2.8189393 | 8.34378 | 2.33E-14 | 1.13E-12 | 22.103 |
| MT1M | 0.55209 | 3.6160972 | 2.61177 | 0.009809 | 0.0224139 | -3.6261 |
| NAT14 | 0.55211 | 4.9128405 | 6.74597 | 2.25E-10 | 3.77E-09 | 13.1304 |
| LRRC23 | 0.55241 | 4.6677982 | 5.58445 | 9.07E-08 | 7.78E-07 | 7.3085 |
| TMEM39B | 0.55244 | 3.4656895 | 8.80454 | 1.42E-15 | 9.48E-14 | 24.8463 |
| IQCC | 0.55245 | 2.0907979 | 10.6622 | 1.14E-20 | 2.32E-18 | 36.3678 |
| AMY2B | 0.55251 | 3.7852778 | 4.76471 | 4.02E-06 | 2.29E-05 | 3.65993 |
| LCK | 0.55255 | 1.1612001 | 3.90033 | 0.000138 | 0.00053 | 0.30193 |
| FSCN1 | 0.5529 | 6.6734898 | 4.60336 | 8.08E-06 | 4.28E-05 | 2.99292 |
| MICA | 0.55295 | 2.0470855 | 4.34569 | 2.38E-05 | 0.0001112 | 1.96514 |
| PARPBP | 0.55318 | 1.5911463 | 5.65723 | 6.36E-08 | 5.71E-07 | 7.65216 |
| TLR10 | 0.55323 | 1.4879205 | 4.84735 | 2.79E-06 | 1.66E-05 | 4.00833 |
| GAL | 0.55327 | 0.9732388 | 3.19385 | 0.001672 | 0.0047849 | -2.0222 |
| MCM2 | 0.55334 | 3.4188096 | 3.92573 | 0.000125 | 0.000486 | 0.39282 |
| RPGR | 0.55336 | 3.3220782 | 5.82463 | 2.78E-08 | 2.72E-07 | 8.45399 |
| WNT5A-AS1 | 0.55351 | 0.8992292 | 5.95771 | 1.42E-08 | 1.50E-07 | 9.10253 |
| RP11-316M1.12 | 0.55363 | 2.8371689 | 4.00303 | 9.31E-05 | 0.000374 | 0.67236 |
| CHSY1 | 0.55369 | 3.8917674 | 6.19669 | 4.18E-09 | 5.06E-08 | 10.2909 |
| RNF19A | 0.55376 | 5.6361469 | 4.48806 | 1.32E-05 | 6.62E-05 | 2.52728 |
| ZNF571 | 0.55384 | 2.3914341 | 8.30503 | 2.93E-14 | 1.39E-12 | 21.875 |
| MGST1 | 0.55393 | 6.2815745 | 3.0278 | 0.002845 | 0.0075731 | -2.5093 |
| LMNB2 | 0.55401 | 4.3228173 | 5.26289 | 4.21E-07 | 3.05E-06 | 5.82745 |
| NEDD1 | 0.55403 | 3.2176465 | 5.81051 | 2.98E-08 | 2.89E-07 | 8.38577 |
| RSAD2 | 0.55408 | 2.7595217 | 3.5082 | 0.000577 | 0.001871 | -1.0374 |
| NLRP3 | 0.55418 | 2.2207903 | 5.34754 | 2.83E-07 | 2.14E-06 | 6.21133 |
| TRADD | 0.55433 | 3.2143836 | 6.81243 | 1.57E-10 | 2.76E-09 | 13.4835 |
| PALM3 | 0.55435 | 1.773439 | 3.62296 | 0.000384 | 0.0013076 | -0.6579 |
| DCDC2 | 0.55439 | 0.7771332 | 5.75488 | 3.93E-08 | 3.71E-07 | 8.11801 |
| TNXA | 0.5544 | 0.5374109 | 5.04923 | 1.13E-06 | 7.36E-06 | 4.87833 |
| SIPA1L1 | 0.5545 | 4.6598345 | 5.19989 | 5.65E-07 | 3.96E-06 | 5.54464 |
| RP11-809O17.1 | 0.55474 | 1.066624 | 6.71542 | 2.66E-10 | 4.36E-09 | 12.9687 |
| WDR90 | 0.55502 | 4.142039 | 5.70948 | 4.92E-08 | 4.54E-07 | 7.90073 |
| DONSON | 0.55515 | 3.8281341 | 6.8935 | 1.01E-10 | 1.86E-09 | 13.917 |
| STX11 | 0.5553 | 1.3361846 | 5.65647 | 6.38E-08 | 5.72E-07 | 7.64857 |
| TES | 0.55535 | 2.0041843 | 4.55535 | 9.91E-06 | 5.13E-05 | 2.79794 |
| TULP3 | 0.55546 | 3.8819877 | 6.44122 | 1.16E-09 | 1.61E-08 | 11.537 |
| NTN1 | 0.55563 | 4.5611899 | 3.87102 | 0.000154 | 0.0005847 | 0.19769 |
| PROCR | 0.55568 | 2.5981637 | 5.54538 | 1.10E-07 | 9.16E-07 | 7.12526 |
| SNIP1 | 0.5557 | 2.7483323 | 10.4252 | 5.23E-20 | 9.57E-18 | 34.8671 |
| PI4K2B | 0.55604 | 3.0329215 | 8.0431 | 1.40E-13 | 5.70E-12 | 20.3457 |
| GJA1 | 0.55614 | 7.2770346 | 2.44635 | 0.015444 | 0.0332044 | -4.028 |
| HSPBAP1 | 0.55655 | 2.9183747 | 8.43598 | 1.33E-14 | 7.01E-13 | 22.6473 |
| C12orf5 | 0.55697 | 3.791732 | 6.4657 | 1.02E-09 | 1.43E-08 | 11.6633 |
| TNFRSF11A | 0.55724 | 1.2095974 | 6.03674 | 9.51E-09 | 1.05E-07 | 9.49217 |
| MRPL13 | 0.55759 | 4.8262874 | 7.2421 | 1.44E-11 | 3.40E-10 | 15.8127 |
| PQLC3 | 0.55759 | 3.1048588 | 6.44973 | 1.11E-09 | 1.54E-08 | 11.5809 |
| MAPK15 | 0.55761 | 1.3576588 | 2.89078 | 0.004342 | 0.0109772 | -2.8937 |
| NUP37 | 0.55775 | 3.4124976 | 7.53057 | 2.79E-12 | 8.09E-11 | 17.4184 |
| PTPRF | 0.55805 | 5.2564214 | 5.45066 | 1.73E-07 | 1.37E-06 | 6.68481 |
| CCDC57 | 0.55807 | 4.3406096 | 7.13336 | 2.66E-11 | 5.82E-10 | 15.216 |
| DEPDC1B | 0.55814 | 1.3388785 | 4.62196 | 7.46E-06 | 3.98E-05 | 3.06894 |
| PI3 | 0.55824 | 0.7806452 | 2.63679 | 0.009141 | 0.0210588 | -3.5632 |
| ITK | 0.55838 | 0.8097622 | 4.66579 | 6.18E-06 | 3.36E-05 | 3.24892 |
| FZD6 | 0.5585 | 1.9093745 | 4.71806 | 4.93E-06 | 2.74E-05 | 3.46527 |
| RP1-74M1.3 | 0.55861 | 1.0138272 | 4.82522 | 3.08E-06 | 1.81E-05 | 3.91459 |
| ZBTB48 | 0.55864 | 4.1876517 | 8.60605 | 4.76E-15 | 2.80E-13 | 23.6575 |
| UACA | 0.55865 | 3.627981 | 5.31309 | 3.33E-07 | 2.47E-06 | 6.05456 |
| CDCA5 | 0.55869 | 2.8004866 | 3.9574 | 0.000111 | 0.0004366 | 0.50682 |
| RP11-342M1.3 | 0.55872 | 1.3352972 | 6.04615 | 9.06E-09 | 1.00E-07 | 9.53878 |
| PLEK2 | 0.55873 | 0.7705807 | 6.12845 | 5.94E-09 | 6.91E-08 | 9.9485 |
| SEC61B | 0.55882 | 6.8233568 | 8.14111 | 7.82E-14 | 3.40E-12 | 20.9155 |
| AKR1A1 | 0.55907 | 6.3348369 | 8.86079 | 1.00E-15 | 6.97E-14 | 25.1851 |
| RP11-686D22.7 | 0.55912 | 1.0770175 | 5.83348 | 2.66E-08 | 2.61E-07 | 8.49684 |
| CD72 | 0.55915 | 2.0018842 | 5.07025 | 1.03E-06 | 6.75E-06 | 4.97042 |
| BAIAP3 | 0.55934 | 4.3095625 | 2.99365 | 0.003166 | 0.00831 | -2.6066 |
| BTF3L4 | 0.5594 | 5.8206828 | 8.88365 | 8.71E-16 | 6.13E-14 | 25.3229 |
| IL32 | 0.5599 | 4.7446226 | 3.07311 | 0.002466 | 0.0066999 | -2.3787 |
| PIK3CD | 0.56033 | 2.4651022 | 5.20682 | 5.47E-07 | 3.85E-06 | 5.57564 |
| RP11-498C9.2 | 0.56049 | 1.8074101 | 4.35664 | 2.27E-05 | 0.0001071 | 2.00788 |
| RPL22 | 0.56052 | 8.662774 | 7.33984 | 8.29E-12 | 2.08E-10 | 16.3532 |
| DTL | 0.56053 | 2.1616389 | 3.3371 | 0.001039 | 0.0031415 | -1.5834 |
| DNAJB1 | 0.56084 | 6.2187408 | 4.94933 | 1.77E-06 | 1.10E-05 | 4.44453 |
| PPP4C | 0.56087 | 5.9686381 | 8.57197 | 5.86E-15 | 3.35E-13 | 23.4545 |
| GEMIN7 | 0.56115 | 3.7680897 | 8.40698 | 1.59E-14 | 8.16E-13 | 22.4758 |
| CIDEB | 0.56127 | 1.8076586 | 5.96137 | 1.39E-08 | 1.47E-07 | 9.12051 |
| LRIF1 | 0.56139 | 3.5322751 | 8.76949 | 1.76E-15 | 1.13E-13 | 24.6356 |
| ZNF217 | 0.56149 | 2.4116666 | 5.47496 | 1.54E-07 | 1.24E-06 | 6.79732 |
| COL6A1 | 0.56152 | 6.157932 | 4.09985 | 6.38E-05 | 0.0002671 | 1.02887 |
| MAP4K1 | 0.56166 | 2.1313707 | 6.16429 | 4.94E-09 | 5.85E-08 | 10.128 |
| MUTYH | 0.56179 | 4.2628223 | 7.47864 | 3.75E-12 | 1.04E-10 | 17.127 |
| LRRC48 | 0.56181 | 2.9071894 | 5.24883 | 4.49E-07 | 3.23E-06 | 5.76413 |
| ITGB5 | 0.56183 | 5.2978762 | 4.57473 | 9.13E-06 | 4.77E-05 | 2.87647 |
| MIR142 | 0.56198 | 1.1590449 | 5.63568 | 7.06E-08 | 6.25E-07 | 7.55009 |
| MIR22HG | 0.56261 | 3.3174278 | 5.02431 | 1.26E-06 | 8.16E-06 | 4.76951 |
| AL139385.1 | 0.56272 | 2.2379508 | 2.42591 | 0.01631 | 0.0347637 | -4.076 |
| ORC6 | 0.56321 | 2.5645847 | 4.86828 | 2.55E-06 | 1.52E-05 | 4.0973 |
| RPS2P32 | 0.56326 | 0.9561177 | 5.56423 | 1.00E-07 | 8.49E-07 | 7.21355 |
| E2F7 | 0.56357 | 0.8553931 | 6.02775 | 9.96E-09 | 1.09E-07 | 9.44769 |
| RP11-108M9.5 | 0.5639 | 1.797645 | 4.60454 | 8.04E-06 | 4.26E-05 | 2.99776 |
| TNFSF14 | 0.56395 | 0.6841679 | 5.07112 | 1.02E-06 | 6.72E-06 | 4.97428 |
| GNS | 0.56396 | 5.0748498 | 5.82337 | 2.79E-08 | 2.73E-07 | 8.4479 |
| CENPL | 0.56404 | 2.3641719 | 7.68116 | 1.17E-12 | 3.69E-11 | 18.269 |
| ST7L | 0.56427 | 3.7302543 | 9.53105 | 1.55E-17 | 1.61E-15 | 29.2773 |
| MFSD10 | 0.56436 | 5.3737294 | 8.25268 | 4.02E-14 | 1.84E-12 | 21.5677 |
| ANXA2P2 | 0.56441 | 0.5822253 | 5.98995 | 1.21E-08 | 1.29E-07 | 9.26108 |
| CTD-2192J16.22 | 0.56443 | 1.4361036 | 3.14029 | 0.001989 | 0.0055612 | -2.1818 |
| SPN | 0.56447 | 1.7991192 | 4.92538 | 1.97E-06 | 1.22E-05 | 4.34147 |
| FOXD3 | 0.56468 | 0.5822042 | 5.42376 | 1.97E-07 | 1.54E-06 | 6.56069 |
| HIST2H4B | 0.5648 | 1.987572 | 5.13485 | 7.63E-07 | 5.19E-06 | 5.25523 |
| NXT1 | 0.56488 | 3.7585268 | 7.4819 | 3.68E-12 | 1.03E-10 | 17.1453 |
| CHRNA1 | 0.56491 | 0.9343014 | 3.69218 | 0.000299 | 0.0010485 | -0.424 |
| IGLV2-8 | 0.56561 | 0.6246518 | 3.1769 | 0.001767 | 0.0050183 | -2.0729 |
| RP11-986E7.7 | 0.56566 | 1.0292201 | 4.04109 | 8.03E-05 | 0.0003282 | 0.81166 |
| IFT57 | 0.56567 | 4.8178161 | 7.47894 | 3.75E-12 | 1.04E-10 | 17.1287 |
| DDAH2 | 0.56597 | 6.3322503 | 6.65477 | 3.70E-10 | 5.80E-09 | 12.649 |
| TICAM2 | 0.56606 | 1.3314474 | 6.18943 | 4.34E-09 | 5.23E-08 | 10.2543 |
| SLC38A6 | 0.56616 | 3.1591267 | 7.70387 | 1.02E-12 | 3.30E-11 | 18.398 |
| CEBPA | 0.56616 | 2.1613269 | 3.0867 | 0.002362 | 0.0064549 | -2.3391 |
| FGD2 | 0.56654 | 3.289729 | 4.45873 | 1.49E-05 | 7.36E-05 | 2.41029 |
| CALHM2 | 0.5666 | 3.18402 | 5.6943 | 5.30E-08 | 4.86E-07 | 7.82838 |
| RRAD | 0.56684 | 2.1735791 | 4.18388 | 4.57E-05 | 0.0001987 | 1.34393 |
| CLEC17A | 0.56688 | 0.7037066 | 4.92852 | 1.95E-06 | 1.20E-05 | 4.35495 |
| RASSF9 | 0.56696 | 0.6900687 | 5.38905 | 2.32E-07 | 1.79E-06 | 6.40115 |
| FLII | 0.56735 | 6.3297169 | 9.7023 | 5.26E-18 | 6.09E-16 | 30.3375 |
| CTD-2270L9.4 | 0.56751 | 2.0658753 | 6.63335 | 4.15E-10 | 6.41E-09 | 12.5365 |
| PRF1 | 0.56755 | 1.0853291 | 5.39672 | 2.24E-07 | 1.73E-06 | 6.43635 |
| OVGP1 | 0.56755 | 1.7460096 | 5.5228 | 1.22E-07 | 1.01E-06 | 7.01979 |
| TP73 | 0.56761 | 1.0685806 | 4.57833 | 8.99E-06 | 4.70E-05 | 2.89106 |
| FANCD2 | 0.56779 | 2.5697668 | 4.50182 | 1.24E-05 | 6.28E-05 | 2.58236 |
| MUL1 | 0.56803 | 4.3062914 | 9.18349 | 1.36E-16 | 1.13E-14 | 27.143 |
| SYNGR2 | 0.56813 | 5.3192136 | 4.10472 | 6.26E-05 | 0.0002624 | 1.047 |
| SIGLEC1 | 0.56827 | 1.7322564 | 3.67496 | 0.000318 | 0.001108 | -0.4826 |
| SNAI1 | 0.56835 | 1.0552868 | 5.56622 | 9.91E-08 | 8.42E-07 | 7.22292 |
| MORN2 | 0.5685 | 5.2999921 | 6.90074 | 9.67E-11 | 1.80E-09 | 13.9558 |
| BMF | 0.56851 | 2.1575094 | 4.78482 | 3.68E-06 | 2.11E-05 | 3.74428 |
| RP11-303E16.2 | 0.56878 | 1.9190172 | 6.03859 | 9.42E-09 | 1.04E-07 | 9.50135 |
| SP140 | 0.56878 | 1.4937348 | 6.58786 | 5.30E-10 | 8.00E-09 | 12.2983 |
| UAP1 | 0.56884 | 4.284758 | 7.40834 | 5.61E-12 | 1.48E-10 | 16.7342 |
| C7orf73 | 0.56894 | 5.7386028 | 7.3929 | 6.13E-12 | 1.61E-10 | 16.6481 |
| MBD2 | 0.56923 | 4.6693651 | 6.94842 | 7.43E-11 | 1.43E-09 | 14.2123 |
| SNRPD2 | 0.56928 | 7.5139204 | 9.41574 | 3.20E-17 | 3.07E-15 | 28.5664 |
| ACSF2 | 0.56951 | 3.8731408 | 6.24423 | 3.26E-09 | 4.07E-08 | 10.5308 |
| GINS2 | 0.56953 | 2.09265 | 5.0646 | 1.05E-06 | 6.91E-06 | 4.94567 |
| PDIK1L | 0.56954 | 2.8708423 | 8.61765 | 4.44E-15 | 2.63E-13 | 23.7267 |
| AC022182.3 | 0.56959 | 0.8220134 | 6.14932 | 5.34E-09 | 6.26E-08 | 10.053 |
| HIVEP3 | 0.56959 | 2.3864234 | 6.64511 | 3.89E-10 | 6.06E-09 | 12.5983 |
| C15orf48 | 0.5696 | 0.7699047 | 4.89823 | 2.23E-06 | 1.35E-05 | 4.22509 |
| TP53I3 | 0.56965 | 4.0820983 | 4.53759 | 1.07E-05 | 5.49E-05 | 2.72618 |
| MAP3K1 | 0.5697 | 2.6736324 | 5.94354 | 1.53E-08 | 1.60E-07 | 9.03305 |
| USP41 | 0.56981 | 2.2880628 | 2.30978 | 0.022096 | 0.0451348 | -4.3414 |
| AGPAT2 | 0.56991 | 3.1721059 | 5.23567 | 4.78E-07 | 3.42E-06 | 5.70498 |
| PLCG2 | 0.56996 | 2.5424268 | 5.92985 | 1.64E-08 | 1.70E-07 | 8.96596 |
| PLEKHF2 | 0.5701 | 3.4106264 | 5.84086 | 2.56E-08 | 2.53E-07 | 8.5326 |
| CCBL2 | 0.57014 | 4.0211367 | 7.05354 | 4.15E-11 | 8.64E-10 | 14.781 |
| SLC43A3 | 0.57016 | 3.2281751 | 3.4882 | 0.000618 | 0.0019896 | -1.1024 |
| ENPEP | 0.57038 | 1.3720642 | 4.25404 | 3.45E-05 | 0.0001549 | 1.61094 |
| MYO9B | 0.57042 | 5.6471181 | 6.93711 | 7.91E-11 | 1.51E-09 | 14.1513 |
| SIPA1L2 | 0.57052 | 4.4505811 | 5.86349 | 2.28E-08 | 2.28E-07 | 8.64238 |
| CARD6 | 0.57059 | 1.740636 | 6.52139 | 7.56E-10 | 1.10E-08 | 11.9519 |
| EPHA1 | 0.57073 | 1.292887 | 5.24559 | 4.56E-07 | 3.28E-06 | 5.74954 |
| HIST2H2BA | 0.57096 | 1.69643 | 4.98412 | 1.52E-06 | 9.59E-06 | 4.59485 |
| FEZF1-AS1 | 0.57101 | 0.606241 | 3.34247 | 0.00102 | 0.0030958 | -1.5667 |
| CD300C | 0.57107 | 1.3434646 | 6.38296 | 1.58E-09 | 2.12E-08 | 11.2374 |
| AC007362.1 | 0.57112 | 3.0036455 | 4.0951 | 6.50E-05 | 0.0002713 | 1.01122 |
| FAM96A | 0.57117 | 5.349268 | 7.71226 | 9.75E-13 | 3.15E-11 | 18.4457 |
| ADPGK | 0.57124 | 4.5590167 | 8.48163 | 1.01E-14 | 5.44E-13 | 22.9176 |
| CITED2 | 0.57134 | 4.4582984 | 5.25401 | 4.39E-07 | 3.17E-06 | 5.78743 |
| DDB2 | 0.5715 | 3.0511555 | 5.79492 | 3.22E-08 | 3.09E-07 | 8.31056 |
| LRIG3 | 0.57152 | 2.4199978 | 5.06051 | 1.07E-06 | 7.02E-06 | 4.92772 |
| ESCO2 | 0.57156 | 1.4440975 | 4.22272 | 3.92E-05 | 0.0001731 | 1.49131 |
| HIST1H2AG | 0.57208 | 0.7057398 | 7.79536 | 6.00E-13 | 2.06E-11 | 18.9195 |
| ZNF691 | 0.57209 | 3.2535032 | 10.1487 | 3.08E-19 | 4.60E-17 | 33.1255 |
| FAM129B | 0.57222 | 4.9242176 | 5.20397 | 5.54E-07 | 3.90E-06 | 5.56288 |
| DPP4 | 0.57225 | 1.0909945 | 4.30678 | 2.79E-05 | 0.000128 | 1.81404 |
| ALDH16A1 | 0.57241 | 3.3507721 | 8.13647 | 8.03E-14 | 3.47E-12 | 20.8884 |
| MTHFS | 0.57245 | 3.9968603 | 6.18043 | 4.54E-09 | 5.43E-08 | 10.2091 |
| AC147651.4 | 0.57246 | 0.89065 | 6.20527 | 3.99E-09 | 4.87E-08 | 10.3341 |
| WARS | 0.57264 | 6.1241232 | 4.79059 | 3.59E-06 | 2.07E-05 | 3.76854 |
| ERI1 | 0.57267 | 2.8730868 | 6.93854 | 7.85E-11 | 1.50E-09 | 14.159 |
| KIF14 | 0.57288 | 0.8446733 | 6.33256 | 2.06E-09 | 2.71E-08 | 10.9796 |
| AC098614.2 | 0.57293 | 1.3960488 | 6.25675 | 3.06E-09 | 3.85E-08 | 10.5941 |
| POMGNT1 | 0.57296 | 5.6943116 | 10.7921 | 4.91E-21 | 1.15E-18 | 37.1933 |
| AKIRIN1 | 0.57299 | 5.6193593 | 8.49014 | 9.62E-15 | 5.22E-13 | 22.9681 |
| IGKV2-30 | 0.57323 | 0.5933623 | 3.48537 | 0.000625 | 0.0020075 | -1.1116 |
| MAN2B1 | 0.57333 | 4.997569 | 5.77799 | 3.50E-08 | 3.34E-07 | 8.22904 |
| BTN3A1 | 0.57337 | 4.3391712 | 5.21828 | 5.18E-07 | 3.67E-06 | 5.62694 |
| AAED1 | 0.57339 | 2.8754271 | 7.82418 | 5.07E-13 | 1.78E-11 | 19.0843 |
| RP4-758J18.13 | 0.57366 | 2.1030787 | 8.73881 | 2.12E-15 | 1.33E-13 | 24.4515 |
| GPN2 | 0.5738 | 3.8390929 | 9.34125 | 5.10E-17 | 4.68E-15 | 28.1087 |
| MYBPH | 0.57409 | 0.5400962 | 4.80922 | 3.31E-06 | 1.92E-05 | 3.84702 |
| HPSE | 0.57412 | 1.9355429 | 4.93153 | 1.92E-06 | 1.19E-05 | 4.36791 |
| TNFRSF25 | 0.57446 | 3.007596 | 4.83899 | 2.90E-06 | 1.71E-05 | 3.97287 |
| GRN | 0.57457 | 6.5443488 | 5.74961 | 4.03E-08 | 3.79E-07 | 8.09271 |
| KTI12 | 0.57461 | 2.9385918 | 10.892 | 2.57E-21 | 6.94E-19 | 37.8294 |
| MB21D1 | 0.57516 | 1.0615818 | 7.80122 | 5.80E-13 | 2.00E-11 | 18.9529 |
| GBGT1 | 0.57529 | 2.3700187 | 6.45178 | 1.10E-09 | 1.52E-08 | 11.5915 |
| ERAP2 | 0.57529 | 2.6512301 | 3.35636 | 0.000973 | 0.0029711 | -1.5232 |
| PRKCD | 0.5753 | 3.3190674 | 4.9152 | 2.07E-06 | 1.27E-05 | 4.29778 |
| CD83 | 0.57534 | 3.6886429 | 4.01673 | 8.83E-05 | 0.0003568 | 0.72241 |
| LIG1 | 0.57547 | 4.0456809 | 6.54322 | 6.73E-10 | 9.88E-09 | 12.0655 |
| HOXA11-AS | 0.57568 | 0.5095436 | 5.18834 | 5.96E-07 | 4.16E-06 | 5.49303 |
| NUP205 | 0.57568 | 4.4420653 | 7.49782 | 3.36E-12 | 9.50E-11 | 17.2345 |
| RGS3 | 0.57579 | 4.0548186 | 4.67693 | 5.89E-06 | 3.22E-05 | 3.29487 |
| KLHL21 | 0.57597 | 4.0632569 | 7.39087 | 6.20E-12 | 1.62E-10 | 16.6368 |
| ARNTL2 | 0.57624 | 1.9141536 | 5.51438 | 1.27E-07 | 1.05E-06 | 6.98053 |
| GINS1 | 0.57635 | 2.1572874 | 5.57895 | 9.31E-08 | 7.95E-07 | 7.28267 |
| FADD | 0.57635 | 3.7962857 | 7.3659 | 7.15E-12 | 1.84E-10 | 16.4979 |
| SNRPB | 0.57649 | 6.1181119 | 7.18491 | 1.99E-11 | 4.53E-10 | 15.4983 |
| HRH1 | 0.57665 | 2.9377366 | 4.19581 | 4.36E-05 | 0.0001903 | 1.38907 |
| ARHGAP11A | 0.57682 | 2.3549676 | 4.27641 | 3.15E-05 | 0.0001432 | 1.69684 |
| CNTF | 0.57692 | 1.5820365 | 4.79745 | 3.48E-06 | 2.01E-05 | 3.79741 |
| TIFA | 0.57701 | 1.914301 | 5.28587 | 3.78E-07 | 2.77E-06 | 5.93126 |
| FAM109B | 0.57705 | 1.4986487 | 5.87412 | 2.17E-08 | 2.18E-07 | 8.69407 |
| HIST2H4A | 0.57708 | 2.9791586 | 4.80014 | 3.44E-06 | 1.99E-05 | 3.80874 |
| FAM76A | 0.5771 | 2.86036 | 9.59041 | 1.07E-17 | 1.14E-15 | 29.6442 |
| ZNF599 | 0.57717 | 2.789518 | 8.62293 | 4.30E-15 | 2.55E-13 | 23.7582 |
| PPP1CB | 0.57746 | 6.7810197 | 8.6903 | 2.85E-15 | 1.77E-13 | 24.1609 |
| RP11-488L18.10 | 0.57771 | 2.5519503 | 4.81642 | 3.20E-06 | 1.87E-05 | 3.87739 |
| HCG22 | 0.57779 | 1.0329202 | 4.41279 | 1.80E-05 | 8.71E-05 | 2.2283 |
| EFCAB12 | 0.57787 | 0.8795773 | 5.79192 | 3.27E-08 | 3.14E-07 | 8.29608 |
| FANK1 | 0.57795 | 1.9212839 | 5.60841 | 8.07E-08 | 7.04E-07 | 7.42132 |
| DBF4 | 0.57796 | 2.9556227 | 5.11957 | 8.18E-07 | 5.51E-06 | 5.18764 |
| ACTL6A | 0.57811 | 4.701625 | 6.57805 | 5.58E-10 | 8.35E-09 | 12.247 |
| RIBC1 | 0.57812 | 1.8645315 | 4.74285 | 4.42E-06 | 2.49E-05 | 3.56855 |
| FBXW9 | 0.57829 | 3.1737636 | 8.82944 | 1.22E-15 | 8.28E-14 | 24.9962 |
| DHDDS | 0.5783 | 4.4196202 | 10.7686 | 5.71E-21 | 1.30E-18 | 37.0436 |
| AC017116.11 | 0.57885 | 1.5495391 | 4.80967 | 3.30E-06 | 1.92E-05 | 3.84892 |
| TMEM185B | 0.57889 | 3.1754332 | 5.55756 | 1.03E-07 | 8.72E-07 | 7.18229 |
| RPS2 | 0.57908 | 10.044805 | 5.84372 | 2.52E-08 | 2.50E-07 | 8.54644 |
| IGLV1-44 | 0.57945 | 0.7332904 | 3.07873 | 0.002422 | 0.0065961 | -2.3623 |
| IFITM2 | 0.57976 | 5.7374543 | 4.17186 | 4.80E-05 | 0.0002073 | 1.29853 |
| CC2D1B | 0.57987 | 3.9370337 | 8.38902 | 1.77E-14 | 8.92E-13 | 22.3698 |
| OSTF1 | 0.5799 | 4.5174918 | 5.38662 | 2.35E-07 | 1.81E-06 | 6.39 |
| ATP8B4 | 0.57993 | 2.132652 | 6.06757 | 8.12E-09 | 9.11E-08 | 9.64511 |
| NR2F2 | 0.57997 | 3.1664787 | 3.75874 | 0.000234 | 0.0008468 | -0.1955 |
| IER2 | 0.58027 | 5.2542918 | 4.11406 | 6.03E-05 | 0.000254 | 1.08179 |
| VENTX | 0.58029 | 0.7689948 | 7.00879 | 5.32E-11 | 1.07E-09 | 14.5383 |
| LMO2 | 0.58034 | 5.4287162 | 4.02975 | 8.39E-05 | 0.0003414 | 0.77006 |
| TWF2 | 0.58039 | 4.786386 | 7.55475 | 2.42E-12 | 7.17E-11 | 17.5545 |
| UBE2S | 0.58059 | 5.549575 | 5.86371 | 2.28E-08 | 2.28E-07 | 8.64344 |
| HMGB1P1 | 0.58073 | 0.9757965 | 5.72186 | 4.62E-08 | 4.30E-07 | 7.95985 |
| PPP1R42 | 0.58073 | 2.0779216 | 4.78368 | 3.70E-06 | 2.12E-05 | 3.73952 |
| TM2D1 | 0.58077 | 5.2254498 | 9.53436 | 1.52E-17 | 1.58E-15 | 29.2977 |
| RP11-77K12.4 | 0.58081 | 1.3848699 | 4.4581 | 1.49E-05 | 7.37E-05 | 2.40778 |
| PDLIM3 | 0.58106 | 4.5503291 | 3.44894 | 0.000709 | 0.0022381 | -1.2292 |
| RPS16 | 0.58118 | 9.8290192 | 7.34214 | 8.18E-12 | 2.06E-10 | 16.3659 |
| ATP6V0B | 0.58119 | 6.8547898 | 8.42384 | 1.44E-14 | 7.46E-13 | 22.5755 |
| PHLDA2 | 0.58173 | 1.1301993 | 3.77995 | 0.000216 | 0.0007888 | -0.122 |
| BRCA1 | 0.58184 | 2.5791874 | 6.45372 | 1.08E-09 | 1.51E-08 | 11.6015 |
| ROBO2 | 0.58208 | 3.7525638 | 3.32234 | 0.001092 | 0.0032858 | -1.6294 |
| SRM | 0.58208 | 5.9275331 | 8.33816 | 2.41E-14 | 1.17E-12 | 22.0699 |
| EVI5 | 0.58222 | 3.480709 | 7.79831 | 5.90E-13 | 2.02E-11 | 18.9363 |
| SLC39A4 | 0.58236 | 2.5436287 | 4.58766 | 8.64E-06 | 4.54E-05 | 2.92898 |
| SEMA3A | 0.58261 | 1.4310861 | 4.39165 | 1.97E-05 | 9.42E-05 | 2.14502 |
| FABP7 | 0.58267 | 6.6112049 | 2.65441 | 0.008695 | 0.0201537 | -3.5186 |
| LRRC36 | 0.58294 | 0.9779904 | 6.18654 | 4.40E-09 | 5.28E-08 | 10.2398 |
| TP73-AS1 | 0.58325 | 3.146839 | 3.85772 | 0.000162 | 0.0006107 | 0.15062 |
| VWA3A | 0.5833 | 1.8208007 | 3.36615 | 0.000941 | 0.002884 | -1.4924 |
| PTMS | 0.58354 | 8.8908603 | 5.27046 | 4.06E-07 | 2.96E-06 | 5.86159 |
| MT2A | 0.58355 | 8.5424541 | 4.12023 | 5.89E-05 | 0.0002485 | 1.10481 |
| ZNF684 | 0.58369 | 2.183855 | 11.5302 | 4.03E-23 | 1.42E-20 | 41.9127 |
| RILPL2 | 0.58382 | 3.6509354 | 7.80813 | 5.57E-13 | 1.93E-11 | 18.9925 |
| RNPEP | 0.58395 | 4.5493229 | 9.59825 | 1.02E-17 | 1.09E-15 | 29.6927 |
| LAMC3 | 0.58402 | 2.5272637 | 4.01688 | 8.82E-05 | 0.0003567 | 0.72295 |
| PAFAH2 | 0.5843 | 2.9451879 | 9.25796 | 8.57E-17 | 7.37E-15 | 27.5981 |
| RP11-806H10.4 | 0.58433 | 0.5763593 | 6.13631 | 5.71E-09 | 6.67E-08 | 9.98782 |
| DNAJC2 | 0.58444 | 4.2702822 | 9.32236 | 5.73E-17 | 5.19E-15 | 27.9927 |
| CCDC89 | 0.58475 | 1.3310412 | 5.13022 | 7.79E-07 | 5.29E-06 | 5.23475 |
| CTD-2540B15.11 | 0.58479 | 1.701465 | 2.85097 | 0.004896 | 0.0121798 | -3.0023 |
| NFAM1 | 0.58511 | 1.6622773 | 6.72004 | 2.60E-10 | 4.26E-09 | 12.9932 |
| TMEM243 | 0.5853 | 4.0185456 | 8.23581 | 4.44E-14 | 2.02E-12 | 21.4688 |
| COL28A1 | 0.58535 | 2.3267189 | 2.87039 | 0.004618 | 0.0115835 | -2.9495 |
| LRRC2 | 0.58551 | 1.3246234 | 4.1428 | 5.39E-05 | 0.00023 | 1.18924 |
| DPH2 | 0.5856 | 4.2436514 | 9.26718 | 8.09E-17 | 7.06E-15 | 27.6546 |
| HMHA1 | 0.58598 | 3.8952182 | 6.45996 | 1.05E-09 | 1.47E-08 | 11.6337 |
| RP11-767N6.7 | 0.58609 | 1.5959277 | 7.89431 | 3.36E-13 | 1.24E-11 | 19.4867 |
| SSU72 | 0.5861 | 6.3847562 | 10.4239 | 5.28E-20 | 9.58E-18 | 34.8589 |
| FAS | 0.58658 | 2.838233 | 3.76983 | 0.000225 | 0.0008165 | -0.1571 |
| KNTC1 | 0.58689 | 3.0044743 | 5.20297 | 5.57E-07 | 3.91E-06 | 5.55839 |
| HECTD3 | 0.58708 | 4.5233486 | 11.9022 | 3.53E-24 | 1.69E-21 | 44.3056 |
| IQCG | 0.5872 | 4.0120304 | 6.04233 | 9.24E-09 | 1.02E-07 | 9.5199 |
| ITPRIP | 0.58775 | 2.8448649 | 6.01439 | 1.07E-08 | 1.16E-07 | 9.38168 |
| EYA3 | 0.58785 | 2.9282684 | 8.64399 | 3.78E-15 | 2.26E-13 | 23.8839 |
| CD101 | 0.5879 | 1.2904562 | 5.31683 | 3.27E-07 | 2.43E-06 | 6.07154 |
| BVES | 0.58793 | 1.8958999 | 4.81816 | 3.18E-06 | 1.86E-05 | 3.88476 |
| TRPV2 | 0.5881 | 2.575308 | 5.90555 | 1.85E-08 | 1.89E-07 | 8.84721 |
| PLXNA3 | 0.58831 | 4.3960703 | 6.26883 | 2.87E-09 | 3.64E-08 | 10.6554 |
| AC074212.5 | 0.58846 | 1.235987 | 6.67506 | 3.31E-10 | 5.27E-09 | 12.7558 |
| TRIB1 | 0.58867 | 3.5788344 | 5.57713 | 9.40E-08 | 8.01E-07 | 7.27411 |
| ASPN | 0.58881 | 1.1723239 | 5.26166 | 4.23E-07 | 3.07E-06 | 5.82191 |
| F2RL2 | 0.58881 | 0.7916453 | 4.57824 | 8.99E-06 | 4.70E-05 | 2.8907 |
| CTSL | 0.58883 | 6.4325935 | 5.96167 | 1.39E-08 | 1.47E-07 | 9.12197 |
| C19orf10 | 0.58892 | 6.0300983 | 9.03996 | 3.32E-16 | 2.56E-14 | 26.2692 |
| IL15 | 0.58895 | 1.058772 | 6.15085 | 5.29E-09 | 6.22E-08 | 10.0606 |
| MLKL | 0.58896 | 1.8809925 | 6.62739 | 4.28E-10 | 6.61E-09 | 12.5053 |
| CTC-1337H24.1 | 0.58896 | 0.8037547 | 6.04036 | 9.33E-09 | 1.03E-07 | 9.5101 |
| ERF | 0.58923 | 5.3019307 | 7.06288 | 3.94E-11 | 8.24E-10 | 14.8318 |
| MKI67 | 0.58924 | 2.1415795 | 3.62743 | 0.000378 | 0.0012897 | -0.6429 |
| TGFBR2 | 0.58947 | 4.1529157 | 5.20256 | 5.58E-07 | 3.92E-06 | 5.55658 |
| LRRC55 | 0.58971 | 3.240526 | 3.3269 | 0.001075 | 0.0032406 | -1.6152 |
| FBXO17 | 0.58976 | 2.3223467 | 3.47085 | 0.000657 | 0.0020961 | -1.1586 |
| RP5-1057J7.6 | 0.59 | 1.0876681 | 8.13676 | 8.02E-14 | 3.47E-12 | 20.8901 |
| PIPOX | 0.59037 | 3.7082371 | 3.93469 | 0.000121 | 0.000472 | 0.42499 |
| PTAR1 | 0.59042 | 3.8825763 | 7.35617 | 7.55E-12 | 1.92E-10 | 16.4438 |
| CD7 | 0.59083 | 1.0822747 | 6.17527 | 4.67E-09 | 5.56E-08 | 10.1831 |
| RP11-545E17.3 | 0.59115 | 1.2491711 | 6.78193 | 1.85E-10 | 3.19E-09 | 13.3212 |
| SLC17A9 | 0.59127 | 1.6336436 | 5.39162 | 2.29E-07 | 1.77E-06 | 6.41296 |
| RP11-189B4.6 | 0.59165 | 0.5683112 | 3.31539 | 0.001118 | 0.0033555 | -1.651 |
| TRIM36 | 0.59185 | 2.8479942 | 3.96692 | 0.000107 | 0.0004228 | 0.54122 |
| CTA-384D8.34 | 0.59216 | 0.4478291 | 6.70206 | 2.86E-10 | 4.65E-09 | 12.8982 |
| EVA1A | 0.59253 | 1.5103725 | 4.9513 | 1.76E-06 | 1.09E-05 | 4.45301 |
| TIMELESS | 0.59261 | 3.2127535 | 4.83063 | 3.01E-06 | 1.77E-05 | 3.93749 |
| SNHG9 | 0.59295 | 5.3274549 | 5.15641 | 6.90E-07 | 4.75E-06 | 5.35088 |
| ZNRF2 | 0.59295 | 2.8095117 | 7.25415 | 1.35E-11 | 3.20E-10 | 15.8792 |
| RP11-783K16.14 | 0.59301 | 1.2981488 | 5.76321 | 3.77E-08 | 3.57E-07 | 8.158 |
| WDR3 | 0.59306 | 3.4018459 | 5.99323 | 1.19E-08 | 1.27E-07 | 9.27727 |
| ABCD3 | 0.5932 | 5.0111593 | 8.84332 | 1.12E-15 | 7.65E-14 | 25.0798 |
| GCOM1 | 0.59323 | 0.9976805 | 4.14357 | 5.37E-05 | 0.0002294 | 1.19216 |
| NOP2 | 0.59345 | 4.7066527 | 6.75234 | 2.18E-10 | 3.67E-09 | 13.1642 |
| PAQR7 | 0.59354 | 2.8843515 | 8.41711 | 1.50E-14 | 7.74E-13 | 22.5357 |
| DAAM1 | 0.59377 | 4.1246143 | 5.97151 | 1.33E-08 | 1.41E-07 | 9.17035 |
| PQLC2 | 0.59393 | 3.8112484 | 10.7932 | 4.87E-21 | 1.15E-18 | 37.2003 |
| MGME1 | 0.59394 | 3.5354194 | 8.74784 | 2.00E-15 | 1.27E-13 | 24.5057 |
| RP11-159D12.5 | 0.59402 | 2.1836795 | 6.05191 | 8.80E-09 | 9.79E-08 | 9.56737 |
| SUSD2 | 0.59432 | 1.5062894 | 4.62196 | 7.46E-06 | 3.98E-05 | 3.06891 |
| SKA1 | 0.59459 | 0.9966184 | 5.59635 | 8.56E-08 | 7.40E-07 | 7.36448 |
| TEKT1 | 0.59463 | 1.1863494 | 3.67074 | 0.000323 | 0.0011225 | -0.4969 |
| TLN1 | 0.59497 | 6.2159083 | 7.0096 | 5.30E-11 | 1.07E-09 | 14.5427 |
| HSPA1A | 0.595 | 6.1199621 | 3.81421 | 0.000191 | 0.000705 | -0.0025 |
| SMIM5 | 0.59512 | 2.0637501 | 4.33207 | 2.51E-05 | 0.0001168 | 1.91214 |
| RNASE3 | 0.59515 | 0.8915136 | 5.36308 | 2.63E-07 | 2.00E-06 | 6.28228 |
| RIPK3 | 0.59564 | 1.8617907 | 4.80079 | 3.43E-06 | 1.99E-05 | 3.81147 |
| MTF2 | 0.59567 | 3.1791988 | 8.91864 | 7.02E-16 | 5.07E-14 | 25.5343 |
| RCSD1 | 0.59577 | 3.4866873 | 5.61783 | 7.71E-08 | 6.75E-07 | 7.46575 |
| NGFR | 0.5958 | 2.6009008 | 3.09094 | 0.00233 | 0.0063797 | -2.3268 |
| TANC1 | 0.59583 | 4.0732774 | 6.22226 | 3.66E-09 | 4.50E-08 | 10.4198 |
| HEXB | 0.59596 | 5.4298106 | 6.24351 | 3.27E-09 | 4.08E-08 | 10.5272 |
| MND1 | 0.59631 | 1.6398867 | 4.73889 | 4.50E-06 | 2.52E-05 | 3.55201 |
| RSPH1 | 0.59645 | 2.994997 | 3.42575 | 0.000768 | 0.0023993 | -1.3035 |
| A1BG | 0.5965 | 3.7617539 | 4.41845 | 1.76E-05 | 8.52E-05 | 2.25063 |
| RGS18 | 0.5966 | 1.7412797 | 5.97411 | 1.31E-08 | 1.39E-07 | 9.18313 |
| HP1BP3 | 0.5966 | 7.0083614 | 7.16703 | 2.20E-11 | 4.97E-10 | 15.4003 |
| TMBIM1 | 0.59667 | 5.3948306 | 4.26375 | 3.32E-05 | 0.0001496 | 1.64821 |
| TMEM2 | 0.59671 | 3.3196952 | 5.24398 | 4.60E-07 | 3.30E-06 | 5.74231 |
| FAM188B | 0.59689 | 2.1702879 | 5.55521 | 1.05E-07 | 8.81E-07 | 7.17132 |
| UTS2 | 0.59699 | 0.6989667 | 6.05129 | 8.83E-09 | 9.81E-08 | 9.56428 |
| HIST2H2BE | 0.59708 | 3.879263 | 4.58306 | 8.81E-06 | 4.62E-05 | 2.91027 |
| KCNQ1 | 0.59715 | 2.258016 | 5.76416 | 3.75E-08 | 3.56E-07 | 8.16255 |
| PLEKHG4 | 0.59737 | 1.8548567 | 4.22267 | 3.92E-05 | 0.0001731 | 1.49113 |
| HFE | 0.59746 | 1.7978491 | 5.92013 | 1.72E-08 | 1.77E-07 | 8.91843 |
| ISYNA1 | 0.59782 | 4.1928448 | 5.28085 | 3.87E-07 | 2.83E-06 | 5.90856 |
| CAST | 0.5979 | 5.3037819 | 6.7159 | 2.65E-10 | 4.35E-09 | 12.9712 |
| FAM180A | 0.59794 | 1.0324254 | 3.71126 | 0.000279 | 0.0009864 | -0.3589 |
| AKAP2 | 0.59807 | 3.5548499 | 5.11212 | 8.47E-07 | 5.67E-06 | 5.15472 |
| KCNN4 | 0.59814 | 1.2929889 | 5.20724 | 5.46E-07 | 3.85E-06 | 5.57749 |
| ESPL1 | 0.59818 | 1.6036556 | 3.68997 | 0.000301 | 0.0010552 | -0.4315 |
| BLVRB | 0.59819 | 5.1642012 | 5.89816 | 1.92E-08 | 1.96E-07 | 8.81114 |
| ETHE1 | 0.59821 | 4.328146 | 7.99344 | 1.88E-13 | 7.32E-12 | 20.0582 |
| FAIM | 0.59821 | 4.3727306 | 7.38357 | 6.46E-12 | 1.68E-10 | 16.5962 |
| C8orf34 | 0.59834 | 1.5686395 | 3.96193 | 0.000109 | 0.0004301 | 0.52316 |
| RP5-1042I8.7 | 0.5988 | 1.4772929 | 8.34364 | 2.33E-14 | 1.13E-12 | 22.1022 |
| MICB | 0.59892 | 1.1625908 | 7.10806 | 3.06E-11 | 6.59E-10 | 15.0778 |
| NIPSNAP3A | 0.59918 | 3.98661 | 4.86467 | 2.59E-06 | 1.55E-05 | 4.08192 |
| RP4-717I23.3 | 0.59934 | 4.0961919 | 5.89319 | 1.97E-08 | 2.01E-07 | 8.78689 |
| PPM1M | 0.59939 | 3.9028809 | 7.48348 | 3.65E-12 | 1.02E-10 | 17.1541 |
| GCH1 | 0.59942 | 1.260623 | 6.77569 | 1.92E-10 | 3.28E-09 | 13.2881 |
| RP3-369A17.4 | 0.59945 | 1.1468933 | 3.99872 | 9.46E-05 | 0.0003794 | 0.65666 |
| BCAS2 | 0.59951 | 5.3323494 | 10.0586 | 5.48E-19 | 7.70E-17 | 32.5598 |
| MT1L | 0.5996 | 2.3400494 | 4.03327 | 8.28E-05 | 0.0003372 | 0.78296 |
| BCAT2 | 0.59984 | 3.9488446 | 8.03089 | 1.50E-13 | 6.03E-12 | 20.275 |
| ZNF367 | 0.5999 | 2.0058255 | 5.23427 | 4.81E-07 | 3.44E-06 | 5.69867 |
| GPR126 | 0.60026 | 1.5136709 | 4.981 | 1.54E-06 | 9.71E-06 | 4.58135 |
| RASSF8 | 0.60047 | 3.927069 | 5.92898 | 1.64E-08 | 1.70E-07 | 8.9617 |
| BCL10 | 0.60061 | 2.5141116 | 10.7823 | 5.23E-21 | 1.21E-18 | 37.131 |
| RP11-452F19.3 | 0.60123 | 3.2940745 | 6.1513 | 5.28E-09 | 6.21E-08 | 10.0629 |
| RP13-131K19.6 | 0.60126 | 0.9692217 | 5.22406 | 5.05E-07 | 3.58E-06 | 5.65286 |
| ITGA3 | 0.60139 | 3.6831763 | 4.21066 | 4.11E-05 | 0.0001807 | 1.44544 |
| HMMR | 0.60141 | 1.5306977 | 5.00166 | 1.40E-06 | 8.94E-06 | 4.67096 |
| PLIN2 | 0.60141 | 4.11174 | 4.19298 | 4.41E-05 | 0.0001923 | 1.37835 |
| SLC35F5 | 0.60152 | 4.1767828 | 8.30836 | 2.88E-14 | 1.36E-12 | 21.8946 |
| CMPK1 | 0.60154 | 5.8618763 | 11.3691 | 1.15E-22 | 3.79E-20 | 40.8789 |
| IPP | 0.60183 | 3.2348622 | 9.17511 | 1.44E-16 | 1.17E-14 | 27.0918 |
| HPD | 0.60183 | 0.6023374 | 5.93543 | 1.59E-08 | 1.66E-07 | 8.99327 |
| RP11-430G17.3 | 0.60196 | 1.3181302 | 6.11858 | 6.25E-09 | 7.22E-08 | 9.8992 |
| CDCA2 | 0.60203 | 1.007062 | 5.58837 | 8.90E-08 | 7.66E-07 | 7.32693 |
| BAX | 0.60211 | 6.3878382 | 7.72821 | 8.88E-13 | 2.90E-11 | 18.5365 |
| FAM91A1 | 0.60223 | 4.1767761 | 7.76064 | 7.35E-13 | 2.45E-11 | 18.7212 |
| FGFRL1 | 0.60271 | 3.6243146 | 4.64543 | 6.75E-06 | 3.64E-05 | 3.16513 |
| IRF3 | 0.60325 | 5.1771695 | 8.89077 | 8.34E-16 | 5.90E-14 | 25.3659 |
| RBM42 | 0.60344 | 5.580978 | 9.9488 | 1.10E-18 | 1.47E-16 | 31.8729 |
| RP11-66N24.3 | 0.60368 | 1.7287873 | 7.00563 | 5.41E-11 | 1.08E-09 | 14.5212 |
| GADD45B | 0.60371 | 5.4013038 | 3.1531 | 0.001908 | 0.0053679 | -2.1438 |
| SIRPB2 | 0.60378 | 1.5227182 | 5.93241 | 1.62E-08 | 1.68E-07 | 8.9785 |
| CDK11B | 0.60398 | 5.4216535 | 11.33 | 1.49E-22 | 4.83E-20 | 40.6283 |
| FBL | 0.60399 | 6.6719106 | 7.29999 | 1.04E-11 | 2.55E-10 | 16.1324 |
| ALDH4A1 | 0.60412 | 5.0817668 | 4.67248 | 6.00E-06 | 3.27E-05 | 3.27651 |
| MXRA8 | 0.60413 | 4.7534705 | 5.06865 | 1.03E-06 | 6.79E-06 | 4.9634 |
| MAP3K8 | 0.60414 | 2.4649261 | 5.34536 | 2.86E-07 | 2.16E-06 | 6.20139 |
| PLS3 | 0.60449 | 5.2152357 | 4.89165 | 2.30E-06 | 1.39E-05 | 4.19695 |
| CTC-241F20.3 | 0.6045 | 1.4857741 | 9.90937 | 1.42E-18 | 1.80E-16 | 31.6267 |
| ITGA2 | 0.60462 | 1.7341715 | 4.91813 | 2.04E-06 | 1.25E-05 | 4.31035 |
| TMEM107 | 0.60479 | 4.0716405 | 8.7875 | 1.57E-15 | 1.03E-13 | 24.7439 |
| FZD2 | 0.60499 | 1.8963007 | 5.53806 | 1.14E-07 | 9.43E-07 | 7.09107 |
| PLXND1 | 0.60523 | 5.4699784 | 5.57384 | 9.55E-08 | 8.13E-07 | 7.25867 |
| GDPD2 | 0.6053 | 2.9487778 | 4.16517 | 4.93E-05 | 0.0002123 | 1.27333 |
| GCA | 0.60554 | 4.4040137 | 5.70953 | 4.91E-08 | 4.54E-07 | 7.90099 |
| GPR82 | 0.6058 | 0.7554024 | 6.42489 | 1.26E-09 | 1.73E-08 | 11.4529 |
| TKTL1 | 0.60581 | 1.4469106 | 3.98366 | 0.0001 | 0.0003991 | 0.60189 |
| FAM72B | 0.60591 | 1.7981733 | 8.2288 | 4.63E-14 | 2.11E-12 | 21.4278 |
| DENND1C | 0.60595 | 2.0840412 | 5.29211 | 3.67E-07 | 2.70E-06 | 5.95948 |
| GLMN | 0.60628 | 2.8277174 | 8.31985 | 2.68E-14 | 1.28E-12 | 21.9622 |
| KRT7 | 0.60637 | 0.972428 | 4.66785 | 6.13E-06 | 3.33E-05 | 3.25739 |
| CCDC136 | 0.60642 | 5.0341757 | 4.01017 | 9.05E-05 | 0.000365 | 0.69842 |
| TMEM50A | 0.6065 | 6.0507078 | 10.6794 | 1.02E-20 | 2.15E-18 | 36.4775 |
| LILRB2 | 0.60654 | 1.6434305 | 5.46372 | 1.62E-07 | 1.30E-06 | 6.74521 |
| H2AFX | 0.60692 | 5.7738703 | 6.71268 | 2.70E-10 | 4.42E-09 | 12.9542 |
| LZIC | 0.60714 | 4.7919544 | 8.83454 | 1.18E-15 | 8.05E-14 | 25.0269 |
| RP6-42F4.1 | 0.60716 | 1.8243949 | 8.81835 | 1.30E-15 | 8.77E-14 | 24.9294 |
| ACADM | 0.60745 | 5.5288409 | 9.68483 | 5.88E-18 | 6.65E-16 | 30.2292 |
| CHST9 | 0.60792 | 3.4019259 | 2.58018 | 0.010715 | 0.0241776 | -3.7048 |
| AOAH | 0.608 | 2.9722946 | 5.37998 | 2.42E-07 | 1.86E-06 | 6.35958 |
| PALMD | 0.60814 | 2.9980555 | 6.3909 | 1.51E-09 | 2.04E-08 | 11.2782 |
| PUSL1 | 0.60816 | 3.3697106 | 8.76968 | 1.75E-15 | 1.13E-13 | 24.6368 |
| RP11-253E3.3 | 0.60856 | 0.6742083 | 6.53776 | 6.93E-10 | 1.01E-08 | 12.0371 |
| FRRS1 | 0.60875 | 0.7307834 | 7.27772 | 1.18E-11 | 2.84E-10 | 16.0092 |
| TCTN2 | 0.60907 | 2.9700597 | 7.06835 | 3.82E-11 | 8.03E-10 | 14.8615 |
| ALG14 | 0.6091 | 2.2793466 | 7.15123 | 2.40E-11 | 5.35E-10 | 15.3137 |
| LYL1 | 0.60932 | 2.9494808 | 5.01798 | 1.30E-06 | 8.37E-06 | 4.74194 |
| SOCS2-AS1 | 0.60932 | 1.3935872 | 4.69788 | 5.38E-06 | 2.96E-05 | 3.38154 |
| MRPL20 | 0.6094 | 6.7651756 | 11.9086 | 3.39E-24 | 1.65E-21 | 44.3465 |
| RBBP8 | 0.60945 | 3.6975033 | 6.24007 | 3.33E-09 | 4.15E-08 | 10.5098 |
| LYPD6 | 0.60953 | 1.953969 | 4.47277 | 1.40E-05 | 7.01E-05 | 2.46621 |
| MRTO4 | 0.60955 | 4.6085267 | 9.49745 | 1.91E-17 | 1.94E-15 | 29.0699 |
| MRPL37 | 0.60956 | 5.3144983 | 10.6652 | 1.11E-20 | 2.32E-18 | 36.3872 |
| PGM2 | 0.60972 | 3.7351733 | 6.82375 | 1.47E-10 | 2.61E-09 | 13.5439 |
| GSN | 0.60984 | 7.4483538 | 4.4442 | 1.58E-05 | 7.77E-05 | 2.35258 |
| SLC2A4RG | 0.61 | 4.4682808 | 6.84496 | 1.31E-10 | 2.36E-09 | 13.6571 |
| TRIM45 | 0.61002 | 2.5211692 | 7.77267 | 6.86E-13 | 2.31E-11 | 18.7898 |
| RP11-848P1.9 | 0.61007 | 2.2229065 | 4.5607 | 9.69E-06 | 5.03E-05 | 2.81957 |
| MMP2 | 0.61017 | 4.6252775 | 3.81968 | 0.000187 | 0.0006928 | 0.01668 |
| RP11-262H14.1 | 0.61019 | 2.0281177 | 5.27769 | 3.93E-07 | 2.87E-06 | 5.89424 |
| MAP10 | 0.61052 | 1.4227137 | 8.89596 | 8.08E-16 | 5.74E-14 | 25.3973 |
| AP1S2 | 0.61056 | 5.6207094 | 6.66255 | 3.54E-10 | 5.60E-09 | 12.6899 |
| TMC8 | 0.61061 | 2.459614 | 6.11903 | 6.24E-09 | 7.21E-08 | 9.90144 |
| RGS2 | 0.61066 | 4.9299355 | 4.7417 | 4.45E-06 | 2.50E-05 | 3.56374 |
| TMED9 | 0.61087 | 6.5695719 | 8.77149 | 1.73E-15 | 1.12E-13 | 24.6477 |
| OSR2 | 0.61112 | 0.8179752 | 4.22934 | 3.81E-05 | 0.0001692 | 1.51655 |
| CEPT1 | 0.61134 | 4.2403663 | 10.8464 | 3.45E-21 | 8.74E-19 | 37.5392 |
| CCDC77 | 0.61135 | 3.1022068 | 7.22539 | 1.58E-11 | 3.69E-10 | 15.7208 |
| MKNK1 | 0.61156 | 4.7155884 | 6.83216 | 1.41E-10 | 2.51E-09 | 13.5888 |
| HIST2H2AA3 | 0.61175 | 1.8478483 | 5.21646 | 5.23E-07 | 3.70E-06 | 5.6188 |
| FAM198B | 0.61203 | 4.5497289 | 4.28567 | 3.04E-05 | 0.0001384 | 1.7325 |
| EFNB2 | 0.61214 | 3.1008995 | 4.59367 | 8.42E-06 | 4.45E-05 | 2.95345 |
| PRDX6 | 0.61221 | 6.8705785 | 6.27599 | 2.76E-09 | 3.51E-08 | 10.6917 |
| RUNX2 | 0.6123 | 1.5299111 | 6.73458 | 2.40E-10 | 3.98E-09 | 13.07 |
| HSPA5 | 0.61236 | 6.9064955 | 6.87062 | 1.14E-10 | 2.09E-09 | 13.7944 |
| COLGALT1 | 0.61239 | 4.7721316 | 6.24945 | 3.17E-09 | 3.98E-08 | 10.5572 |
| MMP23B | 0.61247 | 1.8021395 | 5.74012 | 4.22E-08 | 3.96E-07 | 8.04725 |
| POP4 | 0.6126 | 5.4853657 | 11.2934 | 1.89E-22 | 6.05E-20 | 40.3939 |
| HAPLN3 | 0.61267 | 2.1769361 | 5.14225 | 7.37E-07 | 5.03E-06 | 5.28804 |
| MCL1 | 0.61267 | 6.5916099 | 6.42324 | 1.27E-09 | 1.74E-08 | 11.4444 |
| TLR3 | 0.61299 | 2.3554681 | 5.43963 | 1.82E-07 | 1.44E-06 | 6.63387 |
| ST20 | 0.61306 | 2.5624292 | 5.32133 | 3.20E-07 | 2.39E-06 | 6.092 |
| OIP5 | 0.61307 | 1.4830679 | 5.63928 | 6.94E-08 | 6.16E-07 | 7.56714 |
| DMPK | 0.61329 | 5.4641466 | 6.53988 | 6.85E-10 | 1.00E-08 | 12.0481 |
| RP4-761J14.10 | 0.61332 | 1.3573792 | 6.67805 | 3.26E-10 | 5.20E-09 | 12.7715 |
| CD99 | 0.6136 | 7.176774 | 3.16916 | 0.001812 | 0.0051312 | -2.0961 |
| VAV3 | 0.61396 | 1.355552 | 4.91486 | 2.07E-06 | 1.27E-05 | 4.29631 |
| NOD1 | 0.61409 | 2.0082708 | 7.94757 | 2.46E-13 | 9.33E-12 | 19.7933 |
| RECQL | 0.61417 | 4.0894086 | 7.5642 | 2.30E-12 | 6.83E-11 | 17.6076 |
| ACOT9 | 0.61447 | 4.0105228 | 6.82367 | 1.48E-10 | 2.61E-09 | 13.5435 |
| DNAH11 | 0.6146 | 0.7475157 | 5.36721 | 2.57E-07 | 1.97E-06 | 6.30115 |
| COL4A6 | 0.61471 | 1.289094 | 5.54493 | 1.10E-07 | 9.17E-07 | 7.12318 |
| C2orf81 | 0.61486 | 2.6448851 | 7.5928 | 1.95E-12 | 5.87E-11 | 17.7689 |
| GALE | 0.61493 | 4.0053998 | 9.35549 | 4.66E-17 | 4.33E-15 | 28.1961 |
| EFNB1 | 0.61496 | 3.1669717 | 7.09012 | 3.38E-11 | 7.23E-10 | 14.98 |
| RB1 | 0.61509 | 5.0337707 | 7.33392 | 8.57E-12 | 2.15E-10 | 16.3204 |
| CYBRD1 | 0.61522 | 4.7591025 | 4.97125 | 1.61E-06 | 1.01E-05 | 4.53913 |
| ETV6 | 0.61526 | 2.631203 | 8.07637 | 1.15E-13 | 4.84E-12 | 20.5388 |
| CBX2 | 0.61529 | 2.4392648 | 4.90149 | 2.20E-06 | 1.34E-05 | 4.23905 |
| TMEM109 | 0.61546 | 4.8245847 | 7.97021 | 2.15E-13 | 8.31E-12 | 19.924 |
| SSX2IP | 0.61548 | 3.35821 | 4.89157 | 2.30E-06 | 1.39E-05 | 4.19662 |
| PSME2 | 0.61558 | 6.259104 | 7.17091 | 2.15E-11 | 4.88E-10 | 15.4215 |
| CDC45 | 0.6156 | 1.8985291 | 4.00412 | 9.27E-05 | 0.0003725 | 0.67637 |
| SPPL2A | 0.61566 | 4.206679 | 7.12135 | 2.84E-11 | 6.18E-10 | 15.1504 |
| MIS18BP1 | 0.61572 | 2.7365959 | 6.9422 | 7.69E-11 | 1.48E-09 | 14.1788 |
| LATS2 | 0.61576 | 1.7684002 | 6.29046 | 2.56E-09 | 3.29E-08 | 10.7652 |
| CABP4 | 0.61618 | 1.0282983 | 6.73221 | 2.43E-10 | 4.03E-09 | 13.0575 |
| EHBP1L1 | 0.61619 | 3.8544514 | 6.97718 | 6.34E-11 | 1.25E-09 | 14.3674 |
| RP5-886K2.3 | 0.61661 | 2.3571757 | 7.41958 | 5.26E-12 | 1.41E-10 | 16.7969 |
| HSPB1 | 0.61665 | 7.3150651 | 3.50997 | 0.000573 | 0.0018609 | -1.0316 |
| TMEM159 | 0.6167 | 2.5168027 | 5.42807 | 1.93E-07 | 1.52E-06 | 6.58056 |
| C10orf11 | 0.61674 | 2.2380841 | 6.21052 | 3.89E-09 | 4.75E-08 | 10.3605 |
| SGOL2 | 0.61679 | 1.857449 | 7.0497 | 4.24E-11 | 8.80E-10 | 14.7602 |
| PPP1R18 | 0.61713 | 5.0898121 | 5.59518 | 8.61E-08 | 7.44E-07 | 7.35898 |
| SUSD3 | 0.61737 | 2.6052853 | 4.63609 | 7.02E-06 | 3.77E-05 | 3.12682 |
| MRPS12 | 0.61746 | 4.3029352 | 9.0796 | 2.60E-16 | 2.05E-14 | 26.51 |
| DPH5 | 0.61759 | 4.6054722 | 9.24904 | 9.06E-17 | 7.74E-15 | 27.5435 |
| CD63 | 0.61767 | 9.1467187 | 6.54022 | 6.84E-10 | 1.00E-08 | 12.0498 |
| LRRC41 | 0.61817 | 5.0301079 | 10.3914 | 6.51E-20 | 1.15E-17 | 34.6534 |
| AC006129.2 | 0.61841 | 1.4257028 | 5.72848 | 4.47E-08 | 4.18E-07 | 7.99154 |
| FLT3LG | 0.61859 | 3.2631397 | 6.97234 | 6.51E-11 | 1.27E-09 | 14.3413 |
| VAMP3 | 0.61908 | 6.0873586 | 7.24403 | 1.43E-11 | 3.37E-10 | 15.8234 |
| UROD | 0.61926 | 6.3134739 | 12.3235 | 2.23E-25 | 1.38E-22 | 47.0224 |
| TXNDC17 | 0.61933 | 5.9243198 | 9.26433 | 8.24E-17 | 7.11E-15 | 27.6372 |
| L3HYPDH | 0.61938 | 4.2599167 | 7.14772 | 2.45E-11 | 5.43E-10 | 15.2945 |
| TRMT13 | 0.6194 | 3.2081813 | 7.70115 | 1.04E-12 | 3.34E-11 | 18.3825 |
| ARC | 0.61959 | 3.5924363 | 2.96749 | 0.003433 | 0.0089256 | -2.6804 |
| TMEM217 | 0.61992 | 1.3953362 | 6.12664 | 6.00E-09 | 6.96E-08 | 9.93945 |
| MED18 | 0.61993 | 3.1674516 | 12.5652 | 4.55E-26 | 3.46E-23 | 48.5831 |
| KLF6 | 0.62017 | 4.7371881 | 5.23293 | 4.84E-07 | 3.46E-06 | 5.69268 |
| PALLD | 0.62022 | 5.3193755 | 5.93663 | 1.58E-08 | 1.65E-07 | 8.99915 |
| UTP11L | 0.6204 | 4.6547714 | 10.9421 | 1.86E-21 | 5.13E-19 | 38.1489 |
| CCDC19 | 0.62047 | 0.7988411 | 4.13075 | 5.65E-05 | 0.0002395 | 1.14413 |
| PARP10 | 0.62079 | 4.5458987 | 5.75032 | 4.02E-08 | 3.78E-07 | 8.09613 |
| IGHV3-33 | 0.62096 | 0.5020055 | 3.84121 | 0.000172 | 0.0006459 | 0.09233 |
| NCAPD2 | 0.6214 | 4.2669643 | 6.25522 | 3.08E-09 | 3.87E-08 | 10.5864 |
| CNTRL | 0.62144 | 2.8973134 | 8.67419 | 3.14E-15 | 1.93E-13 | 24.0645 |
| DAP | 0.62169 | 5.6643984 | 9.13718 | 1.82E-16 | 1.45E-14 | 26.8605 |
| B3GALT6 | 0.62201 | 3.849518 | 9.15261 | 1.65E-16 | 1.33E-14 | 26.9546 |
| PTBP3 | 0.62251 | 2.9661487 | 8.50042 | 9.04E-15 | 4.96E-13 | 23.0291 |
| GNLY | 0.62309 | 1.6074172 | 4.09575 | 6.48E-05 | 0.0002708 | 1.01365 |
| ZNF644 | 0.6231 | 4.3794271 | 10.0831 | 4.69E-19 | 6.74E-17 | 32.7136 |
| ARSJ | 0.62333 | 1.5649628 | 4.45521 | 1.51E-05 | 7.46E-05 | 2.39632 |
| RP11-552D4.1 | 0.6234 | 3.0058264 | 3.06483 | 0.002532 | 0.0068491 | -2.4027 |
| CHST6 | 0.62369 | 2.3885581 | 3.83487 | 0.000176 | 0.0006595 | 0.07004 |
| ECHDC2 | 0.62426 | 3.9286414 | 4.15796 | 5.07E-05 | 0.0002178 | 1.24619 |
| PCDH18 | 0.62443 | 2.7521236 | 5.11321 | 8.42E-07 | 5.65E-06 | 5.15955 |
| GIMAP4 | 0.6251 | 4.2265509 | 6.03472 | 9.61E-09 | 1.06E-07 | 9.48217 |
| CD274 | 0.62543 | 1.468601 | 6.05657 | 8.59E-09 | 9.60E-08 | 9.59048 |
| EFCAB10 | 0.62545 | 1.2650441 | 4.2296 | 3.81E-05 | 0.000169 | 1.51753 |
| BRCA2 | 0.62579 | 1.2180556 | 6.42995 | 1.23E-09 | 1.69E-08 | 11.4789 |
| IFRD1 | 0.62588 | 5.3593746 | 8.01445 | 1.66E-13 | 6.54E-12 | 20.1797 |
| ENG | 0.62617 | 4.7048001 | 4.98806 | 1.49E-06 | 9.44E-06 | 4.61193 |
| PALD1 | 0.62626 | 4.3089343 | 6.22242 | 3.65E-09 | 4.50E-08 | 10.4206 |
| BUB1B | 0.6263 | 1.9800673 | 3.99446 | 9.62E-05 | 0.0003846 | 0.64117 |
| TCEB3 | 0.62634 | 4.1505368 | 9.26472 | 8.22E-17 | 7.11E-15 | 27.6395 |
| GLA | 0.6264 | 4.0236804 | 8.40122 | 1.65E-14 | 8.43E-13 | 22.4418 |
| ANXA2R | 0.62658 | 1.4034065 | 6.95987 | 6.98E-11 | 1.35E-09 | 14.274 |
| RP4-730K3.3 | 0.62679 | 0.7647028 | 8.79389 | 1.51E-15 | 9.97E-14 | 24.7823 |
| THRAP3 | 0.62679 | 5.4019451 | 10.7077 | 8.47E-21 | 1.84E-18 | 36.6572 |
| CXCL11 | 0.6269 | 1.0448344 | 4.8377 | 2.92E-06 | 1.72E-05 | 3.96741 |
| CFH | 0.62697 | 2.7340752 | 4.53732 | 1.07E-05 | 5.49E-05 | 2.72507 |
| EFEMP2 | 0.62724 | 4.5769145 | 3.75782 | 0.000235 | 0.0008492 | -0.1987 |
| IL10RB | 0.62746 | 3.8703368 | 7.74775 | 7.93E-13 | 2.63E-11 | 18.6477 |
| GZMH | 0.62757 | 1.0371139 | 5.93989 | 1.56E-08 | 1.62E-07 | 9.01516 |
| MIB2 | 0.62793 | 5.3143956 | 8.34428 | 2.32E-14 | 1.13E-12 | 22.106 |
| APOL6 | 0.62793 | 2.6208934 | 5.38753 | 2.34E-07 | 1.80E-06 | 6.39418 |
| RTP4 | 0.62815 | 2.1842758 | 6.16797 | 4.85E-09 | 5.76E-08 | 10.1465 |
| IL18BP | 0.62831 | 3.7842642 | 5.43656 | 1.85E-07 | 1.46E-06 | 6.61968 |
| SIKE1 | 0.62862 | 4.2204707 | 9.60322 | 9.84E-18 | 1.06E-15 | 29.7234 |
| CSF2RA | 0.62863 | 3.0727834 | 4.59871 | 8.24E-06 | 4.36E-05 | 2.974 |
| GTSE1 | 0.62903 | 1.8691948 | 4.12338 | 5.82E-05 | 0.0002457 | 1.11658 |
| DNAJC22 | 0.62937 | 1.3447136 | 6.05184 | 8.80E-09 | 9.79E-08 | 9.56702 |
| PHEX | 0.6297 | 1.1192051 | 6.93531 | 7.99E-11 | 1.53E-09 | 14.1417 |
| TPM4 | 0.62989 | 6.8858077 | 5.39433 | 2.26E-07 | 1.75E-06 | 6.42535 |
| GDAP2 | 0.63052 | 2.3673381 | 9.61814 | 8.96E-18 | 9.81E-16 | 29.8158 |
| MORN1 | 0.63067 | 2.7297278 | 8.61468 | 4.52E-15 | 2.67E-13 | 23.709 |
| PARP12 | 0.63078 | 3.3245868 | 5.4611 | 1.65E-07 | 1.31E-06 | 6.73309 |
| HIST1H4H | 0.63119 | 2.0587106 | 5.34945 | 2.80E-07 | 2.12E-06 | 6.22003 |
| SPAG4 | 0.6312 | 1.2863789 | 5.32114 | 3.20E-07 | 2.39E-06 | 6.09116 |
| APLN | 0.63124 | 4.9439804 | 3.56618 | 0.00047 | 0.0015643 | -0.847 |
| GALNT10 | 0.63159 | 4.5044238 | 5.06156 | 1.07E-06 | 6.99E-06 | 4.93232 |
| PIK3R3 | 0.63169 | 3.5821577 | 6.61894 | 4.48E-10 | 6.87E-09 | 12.4609 |
| WDR34 | 0.63182 | 4.9414693 | 8.03596 | 1.46E-13 | 5.88E-12 | 20.3043 |
| MCM3 | 0.63207 | 4.6338054 | 7.38997 | 6.23E-12 | 1.63E-10 | 16.6318 |
| DNTTIP2 | 0.63236 | 4.6941934 | 12.7297 | 1.54E-26 | 1.39E-23 | 49.6462 |
| VMP1 | 0.63239 | 5.6858939 | 6.07779 | 7.71E-09 | 8.71E-08 | 9.6959 |
| CA13 | 0.63273 | 1.8969542 | 7.75477 | 7.61E-13 | 2.53E-11 | 18.6877 |
| CDC7 | 0.63289 | 2.9182648 | 6.56654 | 5.94E-10 | 8.84E-09 | 12.187 |
| NEK11 | 0.63291 | 3.4925342 | 5.52448 | 1.21E-07 | 1.00E-06 | 7.02763 |
| GPX1P1 | 0.63292 | 3.1212266 | 5.34965 | 2.80E-07 | 2.12E-06 | 6.22093 |
| TOR4A | 0.63295 | 1.6435146 | 7.41444 | 5.42E-12 | 1.44E-10 | 16.7682 |
| C9orf64 | 0.63336 | 2.3125658 | 4.86203 | 2.62E-06 | 1.56E-05 | 4.07071 |
| TMEM147 | 0.63338 | 6.3456447 | 10.7669 | 5.77E-21 | 1.30E-18 | 37.0334 |
| LXN | 0.63339 | 2.2243446 | 4.42095 | 1.74E-05 | 8.45E-05 | 2.2605 |
| NDUFS5 | 0.63394 | 8.1363894 | 10.4966 | 3.31E-20 | 6.19E-18 | 35.3185 |
| GLI3 | 0.63433 | 2.0873438 | 6.47366 | 9.75E-10 | 1.38E-08 | 11.7045 |
| SYF2 | 0.63453 | 5.4860895 | 12.3207 | 2.27E-25 | 1.38E-22 | 47.0042 |
| CCDC42B | 0.63453 | 0.710923 | 4.42051 | 1.75E-05 | 8.46E-05 | 2.25876 |
| C11orf63 | 0.63456 | 1.1265402 | 4.8906 | 2.31E-06 | 1.40E-05 | 4.1925 |
| NMNAT3 | 0.63464 | 2.3204483 | 3.94692 | 0.000115 | 0.0004525 | 0.46901 |
| C9orf117 | 0.63465 | 1.2880218 | 4.30249 | 2.84E-05 | 0.00013 | 1.79745 |
| MOB3C | 0.63476 | 3.2072622 | 8.67185 | 3.19E-15 | 1.95E-13 | 24.0505 |
| C17orf72 | 0.63479 | 1.5346655 | 5.00574 | 1.38E-06 | 8.80E-06 | 4.6887 |
| RP11-750H9.5 | 0.635 | 2.5498885 | 4.77523 | 3.84E-06 | 2.19E-05 | 3.70403 |
| STXBP3 | 0.63502 | 5.1838385 | 8.57985 | 5.58E-15 | 3.23E-13 | 23.5013 |
| C16orf93 | 0.63512 | 3.2804096 | 6.16605 | 4.89E-09 | 5.80E-08 | 10.1369 |
| XKR8 | 0.63521 | 1.8925455 | 5.39419 | 2.26E-07 | 1.75E-06 | 6.42472 |
| TAP1 | 0.63526 | 5.0502849 | 4.94843 | 1.78E-06 | 1.11E-05 | 4.44061 |
| CTC-246B18.10 | 0.63535 | 3.4174125 | 2.85354 | 0.004858 | 0.0121036 | -2.9953 |
| RP11-395B7.7 | 0.6355 | 3.5309982 | 6.16467 | 4.93E-09 | 5.84E-08 | 10.1299 |
| LMO4 | 0.63559 | 6.5071752 | 5.99574 | 1.17E-08 | 1.26E-07 | 9.28964 |
| RP4-639F20.1 | 0.63575 | 3.841672 | 6.91571 | 8.90E-11 | 1.68E-09 | 14.0363 |
| FCHSD1 | 0.63605 | 2.6997511 | 5.66309 | 6.18E-08 | 5.56E-07 | 7.67994 |
| TMEM69 | 0.63612 | 4.3387456 | 10.656 | 1.18E-20 | 2.38E-18 | 36.3286 |
| HLA-E | 0.63638 | 7.6470372 | 5.53427 | 1.16E-07 | 9.59E-07 | 7.07334 |
| MS4A14 | 0.63654 | 1.3398332 | 5.75695 | 3.89E-08 | 3.67E-07 | 8.12794 |
| COL12A1 | 0.63668 | 1.7414449 | 5.43198 | 1.89E-07 | 1.49E-06 | 6.59856 |
| RP4-758J18.2 | 0.63679 | 4.1217607 | 12.2475 | 3.67E-25 | 2.18E-22 | 46.5319 |
| CD99P1 | 0.63717 | 1.3020733 | 8.02379 | 1.57E-13 | 6.27E-12 | 20.2338 |
| VKORC1 | 0.63717 | 5.7172333 | 7.91669 | 2.95E-13 | 1.09E-11 | 19.6154 |
| MED8 | 0.63736 | 4.2283214 | 11.7821 | 7.76E-24 | 3.31E-21 | 43.5325 |
| EIF3I | 0.63755 | 6.7528251 | 13.0388 | 2.02E-27 | 2.73E-24 | 51.6428 |
| ABCC1 | 0.63775 | 2.9260769 | 7.49057 | 3.51E-12 | 9.87E-11 | 17.1939 |
| NOC2L | 0.63791 | 5.163028 | 10.7644 | 5.87E-21 | 1.31E-18 | 37.0171 |
| ZBTB8OS | 0.63805 | 4.4935897 | 10.2487 | 1.62E-19 | 2.65E-17 | 33.7544 |
| MX1 | 0.63818 | 4.4122144 | 3.97567 | 0.000103 | 0.0004103 | 0.57293 |
| TMEM167B | 0.63831 | 4.8255315 | 9.81232 | 2.62E-18 | 3.21E-16 | 31.0216 |
| P2RY11 | 0.63834 | 3.8055452 | 8.20645 | 5.29E-14 | 2.37E-12 | 21.2969 |
| IRF8 | 0.63852 | 3.4978481 | 4.58471 | 8.75E-06 | 4.59E-05 | 2.91698 |
| HIST1H2AE | 0.63861 | 1.6763075 | 4.95097 | 1.76E-06 | 1.10E-05 | 4.45159 |
| TMEM106A | 0.63873 | 1.8940649 | 6.27932 | 2.72E-09 | 3.46E-08 | 10.7086 |
| LHFP | 0.63881 | 5.5197415 | 6.15673 | 5.14E-09 | 6.05E-08 | 10.0901 |
| ZNF345 | 0.63895 | 2.1659184 | 9.91793 | 1.34E-18 | 1.73E-16 | 31.6801 |
| DNAAF3 | 0.63896 | 1.3067166 | 4.75569 | 4.18E-06 | 2.37E-05 | 3.62218 |
| ZWINT | 0.63912 | 3.3584755 | 4.23886 | 3.67E-05 | 0.0001636 | 1.55287 |
| SLC44A3 | 0.63951 | 2.7457036 | 5.02834 | 1.24E-06 | 8.03E-06 | 4.78709 |
| GBP1P1 | 0.63983 | 0.9097825 | 5.5822 | 9.17E-08 | 7.86E-07 | 7.29795 |
| DAPP1 | 0.63995 | 1.1535419 | 6.65624 | 3.67E-10 | 5.76E-09 | 12.6567 |
| PDK3 | 0.63996 | 2.0850021 | 6.56449 | 6.01E-10 | 8.92E-09 | 12.1762 |
| HLX | 0.64036 | 2.2524269 | 5.32597 | 3.13E-07 | 2.35E-06 | 6.11308 |
| MVP | 0.64046 | 4.9751761 | 5.17642 | 6.29E-07 | 4.37E-06 | 5.43991 |
| CTGF | 0.64071 | 5.3840484 | 3.48036 | 0.000636 | 0.0020382 | -1.1278 |
| HOXA6 | 0.6408 | 0.6303735 | 4.54783 | 1.02E-05 | 5.28E-05 | 2.76752 |
| WARS2 | 0.64128 | 2.7185247 | 10.6983 | 9.00E-21 | 1.94E-18 | 36.5972 |
| AIM1 | 0.64135 | 0.9744319 | 5.97839 | 1.28E-08 | 1.37E-07 | 9.20416 |
| TTC4 | 0.6415 | 3.9106507 | 13.3455 | 2.69E-28 | 5.04E-25 | 53.6239 |
| GPR160 | 0.64192 | 1.9338639 | 6.75221 | 2.18E-10 | 3.67E-09 | 13.1634 |
| UBE2T | 0.64199 | 3.3415592 | 4.87981 | 2.42E-06 | 1.46E-05 | 4.14645 |
| WDR62 | 0.64199 | 1.345126 | 5.8761 | 2.14E-08 | 2.16E-07 | 8.70369 |
| ITGAX | 0.64202 | 3.8355077 | 4.37219 | 2.13E-05 | 0.0001012 | 2.06869 |
| SPSB2 | 0.64203 | 3.5195108 | 6.55136 | 6.44E-10 | 9.51E-09 | 12.1078 |
| KDELR2 | 0.64232 | 5.6553537 | 7.76295 | 7.26E-13 | 2.42E-11 | 18.7344 |
| EMILIN3 | 0.64244 | 1.5798137 | 3.54173 | 0.000512 | 0.0016877 | -0.9276 |
| CD164 | 0.64312 | 5.9318379 | 7.55031 | 2.49E-12 | 7.33E-11 | 17.5294 |
| FAM126A | 0.64318 | 3.1458337 | 5.48402 | 1.47E-07 | 1.19E-06 | 6.83932 |
| EME1 | 0.64335 | 2.194161 | 5.53853 | 1.13E-07 | 9.41E-07 | 7.09326 |
| CEBPG | 0.64391 | 4.2066126 | 9.26582 | 8.16E-17 | 7.09E-15 | 27.6463 |
| ARHGEF1 | 0.64414 | 5.3179494 | 7.07385 | 3.70E-11 | 7.83E-10 | 14.8914 |
| TXLNB | 0.64452 | 1.2818008 | 4.59381 | 8.42E-06 | 4.45E-05 | 2.954 |
| FUBP1 | 0.64474 | 5.8194481 | 6.80227 | 1.66E-10 | 2.90E-09 | 13.4294 |
| SEC22B | 0.64492 | 3.7971245 | 11.6023 | 2.52E-23 | 9.42E-21 | 42.3758 |
| HAUS5 | 0.64548 | 3.6063912 | 6.68997 | 3.06E-10 | 4.92E-09 | 12.8343 |
| PRAM1 | 0.64551 | 2.3030228 | 5.22462 | 5.03E-07 | 3.57E-06 | 5.65539 |
| STAC | 0.64583 | 0.9513247 | 4.30815 | 2.77E-05 | 0.0001275 | 1.81933 |
| NDE1 | 0.64606 | 4.1338436 | 5.79149 | 3.27E-08 | 3.14E-07 | 8.29403 |
| CTSB | 0.64606 | 8.4713382 | 5.83506 | 2.63E-08 | 2.59E-07 | 8.50449 |
| PTP4A3 | 0.64608 | 4.7642626 | 5.02557 | 1.26E-06 | 8.12E-06 | 4.77501 |
| PTPLAD2 | 0.64618 | 1.5897744 | 7.74135 | 8.23E-13 | 2.70E-11 | 18.6113 |
| KDELR3 | 0.64642 | 1.3164386 | 5.46386 | 1.62E-07 | 1.30E-06 | 6.74588 |
| SHMT1 | 0.64651 | 3.5539214 | 8.79438 | 1.51E-15 | 9.96E-14 | 24.7853 |
| CAPZB | 0.64658 | 7.5534919 | 10.0666 | 5.20E-19 | 7.36E-17 | 32.6105 |
| HJURP | 0.64677 | 1.8729243 | 4.29574 | 2.91E-05 | 0.0001332 | 1.77137 |
| TRIP13 | 0.64736 | 1.9099185 | 5.86318 | 2.29E-08 | 2.29E-07 | 8.6409 |
| SCMH1 | 0.64736 | 3.8139034 | 9.91029 | 1.41E-18 | 1.80E-16 | 31.6324 |
| TMEM255A | 0.64744 | 4.3130228 | 3.97311 | 0.000104 | 0.0004138 | 0.56365 |
| RER1 | 0.64758 | 5.9289476 | 11.033 | 1.03E-21 | 2.98E-19 | 38.7283 |
| CARD8 | 0.648 | 4.469876 | 7.46373 | 4.09E-12 | 1.13E-10 | 17.0436 |
| CD180 | 0.64875 | 1.8521668 | 4.9918 | 1.46E-06 | 9.31E-06 | 4.62814 |
| PLD4 | 0.64882 | 4.1885549 | 4.02058 | 8.69E-05 | 0.0003525 | 0.73646 |
| PGD | 0.64955 | 6.3108295 | 8.91947 | 6.99E-16 | 5.06E-14 | 25.5393 |
| SPINK8 | 0.64966 | 0.9377749 | 4.74587 | 4.37E-06 | 2.46E-05 | 3.58116 |
| PHACTR4 | 0.64979 | 3.4613213 | 9.03089 | 3.51E-16 | 2.70E-14 | 26.2141 |
| CSDE1 | 0.65011 | 7.34022 | 10.8466 | 3.45E-21 | 8.74E-19 | 37.5401 |
| NOTCH2 | 0.65015 | 4.5847165 | 6.3068 | 2.35E-09 | 3.05E-08 | 10.8483 |
| MT1E | 0.65028 | 6.1235404 | 3.44664 | 0.000714 | 0.0022524 | -1.2366 |
| GALM | 0.65038 | 2.4929043 | 6.68928 | 3.07E-10 | 4.93E-09 | 12.8307 |
| TNFAIP3 | 0.65056 | 2.8560246 | 5.2566 | 4.33E-07 | 3.13E-06 | 5.79912 |
| POLD1 | 0.65061 | 3.3372547 | 7.26557 | 1.26E-11 | 3.02E-10 | 15.9422 |
| ECSCR | 0.65066 | 3.9428081 | 3.37723 | 0.000907 | 0.0027858 | -1.4575 |
| RNFT1 | 0.65093 | 3.4147705 | 8.32492 | 2.60E-14 | 1.25E-12 | 21.992 |
| RP11-848P1.7 | 0.65114 | 1.8235786 | 2.36871 | 0.018966 | 0.0396238 | -4.2082 |
| MIR24-2 | 0.65114 | 2.3351669 | 5.00859 | 1.36E-06 | 8.70E-06 | 4.7011 |
| ADAMTS7 | 0.65134 | 1.2352127 | 5.91145 | 1.80E-08 | 1.85E-07 | 8.87602 |
| RP11-347C12.3 | 0.65172 | 1.263646 | 6.45141 | 1.10E-09 | 1.53E-08 | 11.5895 |
| KIF18A | 0.65176 | 1.0224418 | 6.23243 | 3.47E-09 | 4.30E-08 | 10.4711 |
| CD52 | 0.65179 | 2.9205065 | 3.30582 | 0.001154 | 0.0034506 | -1.6807 |
| PRMT6 | 0.65207 | 3.4079915 | 10.6625 | 1.13E-20 | 2.32E-18 | 36.3697 |
| RP11-93B14.5 | 0.65211 | 0.8117014 | 5.14383 | 7.32E-07 | 4.99E-06 | 5.29505 |
| NMNAT1 | 0.65245 | 2.5941768 | 12.8289 | 8.04E-27 | 7.52E-24 | 50.2868 |
| TP53I13 | 0.65344 | 4.6561318 | 8.59137 | 5.21E-15 | 3.04E-13 | 23.57 |
| CD84 | 0.65352 | 2.6984746 | 4.7182 | 4.93E-06 | 2.73E-05 | 3.46585 |
| SRSF10 | 0.65395 | 5.5165517 | 10.6306 | 1.39E-20 | 2.78E-18 | 36.1672 |
| CASP7 | 0.65397 | 2.8349873 | 6.68915 | 3.07E-10 | 4.93E-09 | 12.83 |
| TMEM45A | 0.6542 | 3.3796703 | 4.34403 | 2.39E-05 | 0.0001118 | 1.95867 |
| ASB5 | 0.65428 | 0.6906938 | 5.54667 | 1.09E-07 | 9.11E-07 | 7.13131 |
| COL9A3 | 0.65452 | 4.508096 | 3.04706 | 0.002678 | 0.0071872 | -2.454 |
| SCIMP | 0.65453 | 2.0810606 | 5.09063 | 9.34E-07 | 6.20E-06 | 5.06002 |
| OSCP1 | 0.65455 | 3.5634187 | 7.89257 | 3.40E-13 | 1.25E-11 | 19.4767 |
| C6orf118 | 0.65466 | 1.2363376 | 4.50132 | 1.25E-05 | 6.29E-05 | 2.58036 |
| CKLF | 0.65488 | 5.4138487 | 8.33039 | 2.52E-14 | 1.21E-12 | 22.0242 |
| INPPL1 | 0.65535 | 6.3646397 | 6.77131 | 1.96E-10 | 3.36E-09 | 13.2648 |
| EMR2 | 0.65538 | 1.4460422 | 6.01861 | 1.04E-08 | 1.14E-07 | 9.40252 |
| NSUN7 | 0.65546 | 0.8182018 | 6.24432 | 3.26E-09 | 4.07E-08 | 10.5313 |
| DYRK3 | 0.65566 | 2.2572307 | 5.90566 | 1.85E-08 | 1.89E-07 | 8.84776 |
| EFHC1 | 0.65571 | 3.6182028 | 7.43155 | 4.92E-12 | 1.32E-10 | 16.8637 |
| MSTN | 0.65573 | 2.8496426 | 2.48453 | 0.013935 | 0.0303913 | -3.9374 |
| MXD3 | 0.65598 | 3.2141299 | 4.84482 | 2.83E-06 | 1.67E-05 | 3.99762 |
| UBE2J2 | 0.65636 | 5.2978206 | 12.4994 | 7.02E-26 | 4.88E-23 | 48.1581 |
| EGF | 0.65649 | 1.2792308 | 7.46554 | 4.05E-12 | 1.12E-10 | 17.0537 |
| PRPS2 | 0.65661 | 2.9530307 | 4.74403 | 4.40E-06 | 2.48E-05 | 3.57345 |
| DRC1 | 0.65681 | 1.0368086 | 4.09622 | 6.47E-05 | 0.0002705 | 1.01537 |
| CXCR4 | 0.65689 | 4.4572647 | 3.9957 | 9.57E-05 | 0.000383 | 0.64566 |
| MGST2 | 0.65701 | 4.4163957 | 6.41685 | 1.32E-09 | 1.80E-08 | 11.4115 |
| LTBP2 | 0.65702 | 1.7775085 | 6.16815 | 4.84E-09 | 5.76E-08 | 10.1474 |
| RP11-834C11.5 | 0.65748 | 0.8098799 | 4.72878 | 4.70E-06 | 2.62E-05 | 3.50987 |
| RP11-497H16.7 | 0.65753 | 1.9711755 | 5.28271 | 3.84E-07 | 2.81E-06 | 5.91696 |
| SNAPC2 | 0.65762 | 3.9590677 | 7.69351 | 1.09E-12 | 3.49E-11 | 18.3391 |
| ZMYM1 | 0.65807 | 2.2903773 | 9.37258 | 4.19E-17 | 3.93E-15 | 28.301 |
| OMA1 | 0.65826 | 3.310679 | 9.89442 | 1.56E-18 | 1.97E-16 | 31.5333 |
| KDM1A | 0.65837 | 5.4751295 | 6.92795 | 8.32E-11 | 1.58E-09 | 14.102 |
| P4HA1 | 0.6584 | 4.6303345 | 6.44241 | 1.15E-09 | 1.60E-08 | 11.5431 |
| EPHB2 | 0.65864 | 3.0592889 | 4.90905 | 2.12E-06 | 1.30E-05 | 4.27141 |
| MLTK | 0.65893 | 3.0632018 | 5.12337 | 8.04E-07 | 5.42E-06 | 5.20444 |
| RP11-155G14.6 | 0.65894 | 0.8611396 | 5.19544 | 5.76E-07 | 4.04E-06 | 5.52475 |
| METTL21B | 0.65896 | 2.7240437 | 4.57411 | 9.15E-06 | 4.77E-05 | 2.87394 |
| ITGB1P1 | 0.6593 | 2.1484385 | 3.85027 | 0.000167 | 0.0006266 | 0.1243 |
| CRABP2 | 0.65939 | 2.5988953 | 5.44545 | 1.77E-07 | 1.41E-06 | 6.66072 |
| ALPK1 | 0.65957 | 2.0810975 | 6.13969 | 5.61E-09 | 6.56E-08 | 10.0047 |
| PLVAP | 0.65994 | 3.05014 | 3.56495 | 0.000472 | 0.0015703 | -0.8511 |
| PCED1B | 0.65997 | 1.8263667 | 6.36312 | 1.75E-09 | 2.34E-08 | 11.1358 |
| WRAP73 | 0.66023 | 4.1074556 | 10.796 | 4.79E-21 | 1.14E-18 | 37.2179 |
| OXTR | 0.66058 | 2.0234078 | 3.81687 | 0.000189 | 0.0006987 | 0.00682 |
| C6orf141 | 0.66068 | 1.0806005 | 4.37225 | 2.13E-05 | 0.0001012 | 2.06892 |
| EHD2 | 0.6612 | 3.9051549 | 5.17581 | 6.31E-07 | 4.38E-06 | 5.43719 |
| RIT1 | 0.6616 | 4.2602326 | 8.5157 | 8.24E-15 | 4.55E-13 | 23.1198 |
| WAS | 0.66186 | 3.415394 | 5.71868 | 4.70E-08 | 4.36E-07 | 7.94466 |
| WARS2-IT1 | 0.66188 | 0.7660242 | 7.95404 | 2.37E-13 | 9.04E-12 | 19.8307 |
| RN7SL138P | 0.66202 | 1.8857832 | 4.75554 | 4.19E-06 | 2.37E-05 | 3.62153 |
| RP11-153M7.1 | 0.66213 | 0.8567799 | 6.69808 | 2.92E-10 | 4.74E-09 | 12.8771 |
| ZNF593 | 0.66266 | 2.7601126 | 11.6407 | 1.96E-23 | 7.56E-21 | 42.623 |
| PIK3AP1 | 0.66294 | 2.8932884 | 5.31324 | 3.32E-07 | 2.47E-06 | 6.05528 |
| ZMYND10 | 0.663 | 2.3346477 | 4.60696 | 7.96E-06 | 4.22E-05 | 3.00765 |
| HN1L | 0.66305 | 3.9044087 | 6.51013 | 8.03E-10 | 1.16E-08 | 11.8935 |
| FBXO42 | 0.66307 | 3.7701903 | 9.95832 | 1.04E-18 | 1.39E-16 | 31.9325 |
| CECR1 | 0.66458 | 3.5317075 | 4.82679 | 3.06E-06 | 1.80E-05 | 3.92124 |
| CLDN7 | 0.66465 | 1.3484315 | 7.27485 | 1.20E-11 | 2.87E-10 | 15.9934 |
| RP11-230B22.1 | 0.66491 | 0.9088751 | 8.43058 | 1.38E-14 | 7.18E-13 | 22.6153 |
| SOCS1 | 0.66525 | 1.3748101 | 6.26425 | 2.94E-09 | 3.71E-08 | 10.6322 |
| ADCK4 | 0.66526 | 4.8216891 | 9.36419 | 4.41E-17 | 4.13E-15 | 28.2495 |
| APOBR | 0.66531 | 1.7578801 | 6.62297 | 4.39E-10 | 6.75E-09 | 12.482 |
| IL16 | 0.66547 | 2.6106594 | 6.19802 | 4.15E-09 | 5.03E-08 | 10.2976 |
| RPS19 | 0.66553 | 10.225052 | 8.4322 | 1.37E-14 | 7.14E-13 | 22.6249 |
| RPL11 | 0.66573 | 10.125116 | 8.8846 | 8.66E-16 | 6.11E-14 | 25.3287 |
| KIF15 | 0.66588 | 2.0919617 | 5.22483 | 5.03E-07 | 3.57E-06 | 5.65632 |
| LIMD2 | 0.66627 | 4.2988521 | 7.23879 | 1.47E-11 | 3.45E-10 | 15.7945 |
| ZYX | 0.66659 | 6.3300547 | 5.12848 | 7.85E-07 | 5.33E-06 | 5.22705 |
| HEBP2 | 0.66699 | 3.5821724 | 7.9145 | 2.99E-13 | 1.11E-11 | 19.6028 |
| FAM132B | 0.66716 | 1.6827681 | 5.12922 | 7.83E-07 | 5.31E-06 | 5.23031 |
| TCF7 | 0.66722 | 2.1472905 | 6.13075 | 5.87E-09 | 6.84E-08 | 9.96 |
| SYTL3 | 0.66748 | 1.9076134 | 6.61995 | 4.46E-10 | 6.84E-09 | 12.4662 |
| NCAPH | 0.66758 | 2.0361579 | 4.66656 | 6.16E-06 | 3.35E-05 | 3.25209 |
| B4GALT1 | 0.66761 | 3.1647769 | 6.24629 | 3.23E-09 | 4.03E-08 | 10.5412 |
| GAP43 | 0.66772 | 7.0369553 | 3.44319 | 0.000723 | 0.0022771 | -1.2477 |
| RP11-439E19.3 | 0.66785 | 1.7856676 | 6.72728 | 2.50E-10 | 4.12E-09 | 13.0314 |
| SLC25A24 | 0.66802 | 2.1152888 | 6.53821 | 6.91E-10 | 1.01E-08 | 12.0394 |
| ATF5 | 0.66825 | 3.9320515 | 5.73663 | 4.30E-08 | 4.02E-07 | 8.0305 |
| CCL8 | 0.66832 | 1.0792728 | 3.97307 | 0.000104 | 0.0004138 | 0.56349 |
| CDCA7L | 0.66846 | 3.7753437 | 4.50083 | 1.25E-05 | 6.30E-05 | 2.5784 |
| FHAD1 | 0.66862 | 1.372112 | 5.97624 | 1.29E-08 | 1.38E-07 | 9.19359 |
| WDFY4 | 0.66867 | 2.1824745 | 6.20757 | 3.95E-09 | 4.81E-08 | 10.3457 |
| MMP11 | 0.66873 | 1.5695221 | 4.25968 | 3.37E-05 | 0.0001518 | 1.63257 |
| C1orf85 | 0.6689 | 4.4947593 | 7.01162 | 5.24E-11 | 1.05E-09 | 14.5536 |
| RP5-940J5.9 | 0.6691 | 7.617988 | 4.66919 | 6.09E-06 | 3.31E-05 | 3.26294 |
| DENND3 | 0.6696 | 4.0035275 | 6.20313 | 4.04E-09 | 4.92E-08 | 10.3233 |
| HIST1H3E | 0.6696 | 1.0429818 | 5.42442 | 1.96E-07 | 1.54E-06 | 6.56373 |
| TMEM173 | 0.66992 | 3.6670623 | 5.79511 | 3.22E-08 | 3.09E-07 | 8.31149 |
| IGHV3-7 | 0.66993 | 0.6053113 | 4.13973 | 5.45E-05 | 0.0002324 | 1.17775 |
| RWDD3 | 0.67007 | 4.1371256 | 9.21204 | 1.14E-16 | 9.54E-15 | 27.3173 |
| LRR1 | 0.6701 | 2.4088965 | 8.19031 | 5.83E-14 | 2.60E-12 | 21.2026 |
| ODF2L | 0.67027 | 3.3865333 | 8.08933 | 1.06E-13 | 4.53E-12 | 20.6141 |
| FUCA1 | 0.67041 | 3.7079622 | 7.54424 | 2.58E-12 | 7.52E-11 | 17.4953 |
| LTC4S | 0.67047 | 3.4277213 | 4.63849 | 6.95E-06 | 3.74E-05 | 3.13666 |
| CTC-360G5.1 | 0.6706 | 1.4651959 | 4.72533 | 4.78E-06 | 2.66E-05 | 3.49551 |
| SDC4 | 0.67129 | 4.3652185 | 3.10809 | 0.002206 | 0.0060883 | -2.2766 |
| CKAP2L | 0.67156 | 1.4651281 | 5.01851 | 1.30E-06 | 8.36E-06 | 4.74426 |
| KIF9 | 0.67165 | 3.5854789 | 6.92223 | 8.59E-11 | 1.63E-09 | 14.0713 |
| PBX3 | 0.67169 | 3.9115925 | 5.6335 | 7.14E-08 | 6.31E-07 | 7.53978 |
| PTCH2 | 0.67172 | 2.0116806 | 6.7275 | 2.49E-10 | 4.12E-09 | 13.0326 |
| PRC1 | 0.67179 | 3.8261959 | 4.56329 | 9.58E-06 | 4.98E-05 | 2.83008 |
| ZNF28 | 0.67252 | 2.7822984 | 7.58826 | 2.00E-12 | 6.01E-11 | 17.7433 |
| NRM | 0.67304 | 3.3771534 | 5.74648 | 4.09E-08 | 3.85E-07 | 8.07772 |
| CTTNBP2NL | 0.67334 | 3.4322209 | 8.9302 | 6.54E-16 | 4.76E-14 | 25.6041 |
| SLC35D1 | 0.67388 | 2.4632126 | 9.72755 | 4.49E-18 | 5.25E-16 | 30.4944 |
| C1orf174 | 0.67393 | 3.6827015 | 13.0102 | 2.44E-27 | 3.13E-24 | 51.4585 |
| C19orf81 | 0.67396 | 2.2393163 | 5.93445 | 1.60E-08 | 1.66E-07 | 8.98851 |
| SBNO2 | 0.67407 | 3.5761734 | 6.98471 | 6.08E-11 | 1.20E-09 | 14.4081 |
| SCARNA2 | 0.67417 | 2.7806811 | 3.44599 | 0.000716 | 0.0022569 | -1.2387 |
| RHOJ | 0.67442 | 3.1796878 | 5.59604 | 8.57E-08 | 7.41E-07 | 7.36301 |
| RBBP4 | 0.67453 | 6.6555341 | 9.47029 | 2.27E-17 | 2.26E-15 | 28.9023 |
| IGHV3-23 | 0.67474 | 0.7628484 | 3.70315 | 0.000287 | 0.0010121 | -0.3866 |
| TREML1 | 0.67562 | 1.8257628 | 5.67813 | 5.74E-08 | 5.21E-07 | 7.75142 |
| CDK11A | 0.67567 | 4.8570293 | 8.75443 | 1.93E-15 | 1.23E-13 | 24.5453 |
| STK38L | 0.67569 | 4.5555216 | 6.96298 | 6.86E-11 | 1.33E-09 | 14.2907 |
| NUMBL | 0.67594 | 4.6496757 | 6.47608 | 9.63E-10 | 1.36E-08 | 11.717 |
| GPR84 | 0.6761 | 1.0242296 | 5.61305 | 7.89E-08 | 6.90E-07 | 7.4432 |
| FTLP3 | 0.67623 | 3.3327226 | 4.09042 | 6.62E-05 | 0.000276 | 0.99383 |
| DAB2 | 0.67624 | 4.2887032 | 5.54551 | 1.10E-07 | 9.15E-07 | 7.1259 |
| AC018766.5 | 0.67661 | 3.2388694 | 6.7169 | 2.64E-10 | 4.33E-09 | 12.9765 |
| IKZF1 | 0.67668 | 2.3899931 | 6.1043 | 6.73E-09 | 7.72E-08 | 9.82789 |
| ADAM28 | 0.67668 | 3.3767692 | 4.92583 | 1.97E-06 | 1.21E-05 | 4.3434 |
| PTK7 | 0.67685 | 4.1193932 | 6.54532 | 6.66E-10 | 9.79E-09 | 12.0764 |
| KCNK13 | 0.67691 | 1.3923562 | 7.77642 | 6.71E-13 | 2.26E-11 | 18.8113 |
| MAP2K3 | 0.67697 | 3.3769995 | 7.21001 | 1.73E-11 | 4.00E-10 | 15.6361 |
| RP11-503N18.1 | 0.67705 | 0.7880353 | 6.25131 | 3.14E-09 | 3.94E-08 | 10.5666 |
| GBX2 | 0.6772 | 2.3571315 | 3.67309 | 0.00032 | 0.0011147 | -0.4889 |
| P2RY6 | 0.67747 | 1.7537568 | 5.20386 | 5.54E-07 | 3.90E-06 | 5.56241 |
| MLIP | 0.67774 | 2.2811057 | 3.92281 | 0.000127 | 0.0004911 | 0.38236 |
| TGFB1I1 | 0.67789 | 3.7791523 | 4.69328 | 5.49E-06 | 3.02E-05 | 3.36247 |
| ARHGAP9 | 0.67812 | 3.1510861 | 6.11105 | 6.50E-09 | 7.48E-08 | 9.86158 |
| PTPN2 | 0.67821 | 3.8027481 | 10.1191 | 3.72E-19 | 5.42E-17 | 32.9393 |
| C19orf55 | 0.67856 | 3.1255803 | 6.65157 | 3.76E-10 | 5.88E-09 | 12.6322 |
| RPA3 | 0.67869 | 4.4489861 | 7.72144 | 9.24E-13 | 3.01E-11 | 18.4979 |
| AGAP2-AS1 | 0.67889 | 1.8278495 | 3.56508 | 0.000472 | 0.0015698 | -0.8506 |
| CNIH4 | 0.67897 | 4.4544878 | 9.79083 | 3.01E-18 | 3.62E-16 | 30.8878 |
| BGN | 0.67927 | 5.3499702 | 3.51501 | 0.000563 | 0.001832 | -1.0152 |
| C1orf86 | 0.67943 | 5.2559614 | 8.48217 | 1.01E-14 | 5.44E-13 | 22.9208 |
| CCDC81 | 0.6796 | 1.1545568 | 8.02323 | 1.57E-13 | 6.28E-12 | 20.2306 |
| MYL6 | 0.67983 | 9.8236879 | 8.82329 | 1.26E-15 | 8.55E-14 | 24.9592 |
| ICMT | 0.67987 | 4.3231466 | 11.0535 | 9.01E-22 | 2.64E-19 | 38.8596 |
| DLGAP1-AS2 | 0.67998 | 1.8190395 | 6.75073 | 2.20E-10 | 3.69E-09 | 13.1556 |
| DCLRE1B | 0.68024 | 2.5304279 | 10.3476 | 8.62E-20 | 1.48E-17 | 34.3772 |
| KDELC2 | 0.68033 | 2.970651 | 5.79294 | 3.25E-08 | 3.12E-07 | 8.30101 |
| HOXD11 | 0.68035 | 0.585237 | 4.53389 | 1.09E-05 | 5.56E-05 | 2.71126 |
| PABPC4 | 0.68045 | 5.9388143 | 11.7062 | 1.28E-23 | 5.09E-21 | 43.0441 |
| ZNF790 | 0.6805 | 2.6894291 | 9.64919 | 7.36E-18 | 8.21E-16 | 30.0081 |
| CSF3R | 0.68076 | 3.7752456 | 5.25113 | 4.45E-07 | 3.20E-06 | 5.77447 |
| RPS8 | 0.68094 | 9.7499128 | 8.46828 | 1.10E-14 | 5.88E-13 | 22.8385 |
| RP11-638I2.6 | 0.68098 | 3.1525145 | 3.52208 | 0.000549 | 0.0017919 | -0.9921 |
| GCLM | 0.68125 | 3.1336309 | 6.96879 | 6.64E-11 | 1.30E-09 | 14.3221 |
| IQGAP3 | 0.68139 | 1.4795773 | 4.9106 | 2.11E-06 | 1.29E-05 | 4.27808 |
| SWAP70 | 0.68152 | 3.5047879 | 5.73035 | 4.43E-08 | 4.14E-07 | 8.00047 |
| IRF5 | 0.68194 | 3.0759997 | 5.70636 | 4.99E-08 | 4.60E-07 | 7.88586 |
| YTHDF2 | 0.68198 | 4.9700054 | 12.1933 | 5.24E-25 | 2.94E-22 | 46.1825 |
| GPX1 | 0.68205 | 7.8038214 | 7.26373 | 1.28E-11 | 3.05E-10 | 15.932 |
| CD97 | 0.68238 | 3.4038252 | 5.30226 | 3.50E-07 | 2.59E-06 | 6.00544 |
| THEMIS2 | 0.68239 | 3.9329639 | 5.39877 | 2.22E-07 | 1.72E-06 | 6.44576 |
| SLC4A7 | 0.68247 | 2.73269 | 7.54415 | 2.58E-12 | 7.52E-11 | 17.4948 |
| TNFSF8 | 0.68247 | 1.5562644 | 6.3774 | 1.62E-09 | 2.18E-08 | 11.2089 |
| FAM95B1 | 0.68258 | 2.2371682 | 4.67834 | 5.85E-06 | 3.20E-05 | 3.30069 |
| SEPN1 | 0.68273 | 5.808786 | 6.50738 | 8.15E-10 | 1.17E-08 | 11.8792 |
| EGR1 | 0.683 | 5.8704144 | 2.7109 | 0.007395 | 0.017472 | -3.3737 |
| ZNF474 | 0.68302 | 0.8129105 | 5.51002 | 1.30E-07 | 1.07E-06 | 6.96023 |
| SPHK1 | 0.68351 | 2.7378891 | 5.03888 | 1.18E-06 | 7.68E-06 | 4.8331 |
| CNN2 | 0.68369 | 3.6587543 | 4.46788 | 1.43E-05 | 7.13E-05 | 2.44674 |
| RHPN2 | 0.68373 | 3.3451351 | 5.80008 | 3.14E-08 | 3.02E-07 | 8.33543 |
| EMB | 0.68376 | 2.0793026 | 6.08337 | 7.49E-09 | 8.48E-08 | 9.72364 |
| TRIM22 | 0.68389 | 5.0846877 | 4.70302 | 5.26E-06 | 2.90E-05 | 3.40282 |
| SLC26A2 | 0.68392 | 2.0630626 | 7.3451 | 8.04E-12 | 2.03E-10 | 16.3824 |
| ITPRIPL2 | 0.68433 | 2.6532343 | 6.92616 | 8.40E-11 | 1.60E-09 | 14.0924 |
| ZBTB42 | 0.68433 | 1.4010635 | 7.16662 | 2.20E-11 | 4.97E-10 | 15.398 |
| GALNT2 | 0.68486 | 5.1794209 | 8.03322 | 1.48E-13 | 5.96E-12 | 20.2885 |
| STIL | 0.685 | 1.4345972 | 8.45245 | 1.21E-14 | 6.40E-13 | 22.7448 |
| CTC-429P9.4 | 0.68503 | 2.5486748 | 2.78253 | 0.006 | 0.0145443 | -3.1859 |
| HOXA3 | 0.68503 | 0.674577 | 4.37596 | 2.10E-05 | 9.99E-05 | 2.08346 |
| MIER1 | 0.68504 | 3.6424167 | 12.846 | 7.19E-27 | 6.99E-24 | 50.3976 |
| C1orf213 | 0.68521 | 3.1338338 | 7.2795 | 1.17E-11 | 2.83E-10 | 16.0191 |
| CCDC24 | 0.68525 | 3.3271895 | 7.40612 | 5.68E-12 | 1.50E-10 | 16.7218 |
| PARP14 | 0.68528 | 3.6073007 | 6.01177 | 1.08E-08 | 1.17E-07 | 9.36871 |
| NFKBIB | 0.68551 | 4.3672397 | 9.21974 | 1.09E-16 | 9.16E-15 | 27.3644 |
| IL2RG | 0.6856 | 1.9284136 | 5.46313 | 1.63E-07 | 1.30E-06 | 6.7425 |
| BTG3 | 0.68572 | 4.7003076 | 6.80012 | 1.68E-10 | 2.93E-09 | 13.418 |
| GPBP1L1 | 0.68614 | 5.1123279 | 11.2205 | 3.04E-22 | 9.36E-20 | 39.9271 |
| SMIM4 | 0.68656 | 4.4233523 | 7.32796 | 8.87E-12 | 2.21E-10 | 16.2873 |
| NRP1 | 0.68658 | 4.149699 | 4.50489 | 1.23E-05 | 6.21E-05 | 2.59468 |
| B9D2 | 0.68661 | 2.5442186 | 8.93659 | 6.29E-16 | 4.61E-14 | 25.6428 |
| CTC-425F1.4 | 0.6867 | 3.305367 | 3.98301 | 0.000101 | 0.0004 | 0.59953 |
| ERMAP | 0.68679 | 3.0321619 | 9.43751 | 2.79E-17 | 2.72E-15 | 28.7004 |
| TBC1D1 | 0.6868 | 5.0051985 | 6.18659 | 4.40E-09 | 5.28E-08 | 10.24 |
| PTGER4 | 0.68693 | 1.6890144 | 5.45667 | 1.68E-07 | 1.34E-06 | 6.71262 |
| DNAJC8 | 0.68697 | 6.5621659 | 13.1192 | 1.19E-27 | 1.71E-24 | 52.1627 |
| EBI3 | 0.68723 | 2.7215407 | 5.77338 | 3.58E-08 | 3.41E-07 | 8.20685 |
| PPP1R15A | 0.68729 | 4.4553825 | 6.127 | 5.99E-09 | 6.95E-08 | 9.94126 |
| RAB27A | 0.68744 | 2.4148061 | 7.61417 | 1.72E-12 | 5.29E-11 | 17.8896 |
| ZMPSTE24 | 0.68749 | 4.6662818 | 11.025 | 1.08E-21 | 3.10E-19 | 38.6776 |
| VMO1 | 0.68756 | 2.3412681 | 7.4508 | 4.40E-12 | 1.20E-10 | 16.9712 |
| RRAGC | 0.68757 | 4.473129 | 11.2795 | 2.07E-22 | 6.54E-20 | 40.3047 |
| LSM14A | 0.6876 | 6.0852476 | 9.30461 | 6.41E-17 | 5.73E-15 | 27.8839 |
| NFIA | 0.68786 | 4.2723088 | 5.63882 | 6.96E-08 | 6.17E-07 | 7.56496 |
| LPAR5 | 0.68799 | 3.1410306 | 4.78063 | 3.75E-06 | 2.15E-05 | 3.72671 |
| STC1 | 0.68807 | 0.943824 | 3.99215 | 9.70E-05 | 0.0003876 | 0.63277 |
| FES | 0.68826 | 2.8041766 | 7.40185 | 5.82E-12 | 1.53E-10 | 16.698 |
| ARMCX2 | 0.68831 | 4.7652972 | 6.75948 | 2.09E-10 | 3.54E-09 | 13.202 |
| MOB1A | 0.68859 | 4.702706 | 8.64464 | 3.76E-15 | 2.26E-13 | 23.8878 |
| MORN5 | 0.68861 | 2.497292 | 4.55084 | 1.01E-05 | 5.23E-05 | 2.77967 |
| HAND2 | 0.68871 | 0.9069083 | 4.0574 | 7.53E-05 | 0.0003094 | 0.87171 |
| SLC37A2 | 0.68936 | 2.1344017 | 5.39164 | 2.29E-07 | 1.77E-06 | 6.41302 |
| STAC3 | 0.68968 | 2.1224237 | 7.13864 | 2.58E-11 | 5.67E-10 | 15.2448 |
| IGKV1-5 | 0.68976 | 0.6925171 | 3.45683 | 0.00069 | 0.0021878 | -1.2038 |
| SOD3 | 0.68997 | 2.8891237 | 3.64872 | 0.00035 | 0.0012065 | -0.5713 |
| IL10RA | 0.69035 | 2.9865035 | 5.34703 | 2.83E-07 | 2.14E-06 | 6.20898 |
| RTCA | 0.6906 | 4.1536188 | 10.7724 | 5.57E-21 | 1.28E-18 | 37.0681 |
| COL15A1 | 0.69092 | 1.2357011 | 4.98359 | 1.52E-06 | 9.61E-06 | 4.59257 |
| CAP1 | 0.69157 | 7.0379482 | 9.7976 | 2.88E-18 | 3.50E-16 | 30.9299 |
| DRAM2 | 0.69179 | 5.0942592 | 8.96442 | 5.30E-16 | 3.96E-14 | 25.8112 |
| SMC5 | 0.69181 | 3.5952662 | 7.72759 | 8.91E-13 | 2.91E-11 | 18.5329 |
| PPP1R3B | 0.69194 | 1.9515869 | 7.13687 | 2.60E-11 | 5.71E-10 | 15.2351 |
| NFATC2 | 0.69268 | 1.8675396 | 7.29549 | 1.07E-11 | 2.60E-10 | 16.1075 |
| GCK | 0.69294 | 1.6166751 | 5.57534 | 9.48E-08 | 8.08E-07 | 7.26569 |
| RP11-69E11.4 | 0.69321 | 3.1496396 | 4.09264 | 6.56E-05 | 0.0002737 | 1.0021 |
| SIGLEC10 | 0.69332 | 3.7696272 | 5.13883 | 7.49E-07 | 5.10E-06 | 5.27286 |
| CLEC12A | 0.69336 | 0.8981326 | 5.64754 | 6.66E-08 | 5.95E-07 | 7.60624 |
| PLXDC2 | 0.69343 | 3.8907194 | 5.01737 | 1.30E-06 | 8.39E-06 | 4.7393 |
| LMCD1 | 0.69343 | 3.5558626 | 5.86866 | 2.23E-08 | 2.23E-07 | 8.66751 |
| RP11-145M9.4 | 0.69377 | 4.809068 | 6.78804 | 1.79E-10 | 3.09E-09 | 13.3537 |
| AC006129.4 | 0.69396 | 2.1331387 | 4.67223 | 6.01E-06 | 3.28E-05 | 3.27545 |
| C1orf109 | 0.694 | 4.3062488 | 9.69386 | 5.55E-18 | 6.34E-16 | 30.2852 |
| STK3 | 0.69438 | 3.1567147 | 8.75116 | 1.96E-15 | 1.24E-13 | 24.5256 |
| PRKD2 | 0.6947 | 3.632699 | 9.44645 | 2.64E-17 | 2.59E-15 | 28.7555 |
| KDELR1 | 0.69495 | 5.9184484 | 8.55484 | 6.50E-15 | 3.68E-13 | 23.3525 |
| STAT1 | 0.69505 | 5.6662087 | 5.84081 | 2.56E-08 | 2.53E-07 | 8.53233 |
| SH2D4A | 0.6951 | 0.9480187 | 5.48152 | 1.49E-07 | 1.20E-06 | 6.82774 |
| PINK1-AS | 0.6956 | 1.4411683 | 12.9904 | 2.78E-27 | 3.36E-24 | 51.3303 |
| FAP | 0.69581 | 1.7065607 | 6.05669 | 8.59E-09 | 9.60E-08 | 9.5911 |
| DUSP5 | 0.69626 | 2.0827842 | 4.36786 | 2.17E-05 | 0.0001029 | 2.05173 |
| PCNA | 0.69652 | 5.8959455 | 7.83983 | 4.63E-13 | 1.65E-11 | 19.1739 |
| CD302 | 0.69708 | 3.0860779 | 5.70005 | 5.15E-08 | 4.73E-07 | 7.85576 |
| MEOX2 | 0.69746 | 0.853159 | 3.58965 | 0.000432 | 0.0014545 | -0.7692 |
| RP11-160O5.1 | 0.6976 | 1.8622956 | 5.55257 | 1.06E-07 | 8.90E-07 | 7.15895 |
| TYMS | 0.69763 | 4.3526966 | 4.02946 | 8.40E-05 | 0.0003416 | 0.76898 |
| SLC25A43 | 0.69771 | 2.3996612 | 5.96882 | 1.34E-08 | 1.42E-07 | 9.15711 |
| SGOL1 | 0.69772 | 1.2874715 | 5.63359 | 7.14E-08 | 6.31E-07 | 7.5402 |
| ARHGAP18 | 0.69784 | 2.575032 | 5.87425 | 2.16E-08 | 2.18E-07 | 8.69471 |
| SNORD3A | 0.69787 | 5.258834 | 2.27645 | 0.024059 | 0.0485454 | -4.4152 |
| C1QTNF6 | 0.69816 | 2.4569184 | 6.0454 | 9.10E-09 | 1.01E-07 | 9.53509 |
| OSR1 | 0.69823 | 1.1206717 | 4.48423 | 1.34E-05 | 6.71E-05 | 2.51197 |
| VAMP5 | 0.69837 | 5.4417949 | 5.70744 | 4.96E-08 | 4.58E-07 | 7.891 |
| MYO1G | 0.69852 | 1.137031 | 6.05253 | 8.77E-09 | 9.76E-08 | 9.57042 |
| CEBPB | 0.69862 | 4.2840553 | 4.91728 | 2.05E-06 | 1.26E-05 | 4.30672 |
| FANCI | 0.69867 | 2.8386254 | 4.79921 | 3.46E-06 | 2.00E-05 | 3.80484 |
| CDC6 | 0.69878 | 1.7474518 | 6.00489 | 1.12E-08 | 1.21E-07 | 9.33476 |
| IGLV1-40 | 0.69938 | 0.8540339 | 3.75789 | 0.000235 | 0.0008491 | -0.1985 |
| WDR16 | 0.69953 | 1.6628014 | 4.4362 | 1.64E-05 | 8.00E-05 | 2.32086 |
| GPC4 | 0.70004 | 3.4999669 | 6.10361 | 6.75E-09 | 7.74E-08 | 9.82449 |
| KCNE3 | 0.70031 | 1.9748599 | 5.59065 | 8.80E-08 | 7.59E-07 | 7.33767 |
| PNRC2 | 0.70044 | 5.6087729 | 10.4415 | 4.71E-20 | 8.69E-18 | 34.97 |
| LGALS1 | 0.70056 | 8.0068365 | 4.11403 | 6.03E-05 | 0.000254 | 1.08169 |
| LPCAT2 | 0.70112 | 3.0439805 | 5.3219 | 3.19E-07 | 2.39E-06 | 6.09459 |
| ANXA5 | 0.70143 | 7.7235347 | 6.49331 | 8.78E-10 | 1.25E-08 | 11.8063 |
| THBD | 0.70158 | 1.508994 | 4.44635 | 1.57E-05 | 7.71E-05 | 2.36109 |
| RDH5 | 0.70165 | 2.8660699 | 6.4709 | 9.90E-10 | 1.40E-08 | 11.6902 |
| C9orf89 | 0.70167 | 4.2006318 | 5.1332 | 7.69E-07 | 5.23E-06 | 5.24791 |
| TOM1L1 | 0.70186 | 1.9437717 | 4.34569 | 2.38E-05 | 0.0001112 | 1.96516 |
| GALNT3 | 0.70196 | 1.1783508 | 6.18544 | 4.43E-09 | 5.31E-08 | 10.2343 |
| WDR77 | 0.70236 | 4.31573 | 11.435 | 7.51E-23 | 2.54E-20 | 41.3015 |
| CEP89 | 0.7027 | 3.427291 | 8.58601 | 5.38E-15 | 3.12E-13 | 23.5381 |
| S100PBP | 0.70274 | 3.694061 | 10.228 | 1.86E-19 | 2.97E-17 | 33.6237 |
| PLBD1 | 0.70314 | 1.8695322 | 5.94512 | 1.51E-08 | 1.59E-07 | 9.04075 |
| SIMC1 | 0.70326 | 2.1536971 | 5.91746 | 1.74E-08 | 1.80E-07 | 8.90536 |
| IGHA2 | 0.70335 | 0.9355525 | 3.44189 | 0.000726 | 0.0022859 | -1.2518 |
| LINC01023 | 0.7034 | 2.2256443 | 6.34168 | 1.96E-09 | 2.59E-08 | 11.0262 |
| WDR96 | 0.70343 | 1.4703028 | 5.1642 | 6.66E-07 | 4.60E-06 | 5.38549 |
| PFN1 | 0.70361 | 8.220854 | 7.97277 | 2.12E-13 | 8.22E-12 | 19.9387 |
| RRAS | 0.70378 | 4.2959216 | 6.68034 | 3.22E-10 | 5.14E-09 | 12.7836 |
| LCTL | 0.70422 | 1.1562023 | 6.91455 | 8.96E-11 | 1.69E-09 | 14.03 |
| USP1 | 0.70434 | 4.2146846 | 10.3287 | 9.73E-20 | 1.65E-17 | 34.258 |
| CEP112 | 0.70435 | 2.462666 | 7.8528 | 4.29E-13 | 1.54E-11 | 19.2483 |
| CCDC30 | 0.70452 | 2.1544091 | 8.89828 | 7.96E-16 | 5.70E-14 | 25.4113 |
| KDM4A | 0.70468 | 4.1787902 | 9.52348 | 1.63E-17 | 1.68E-15 | 29.2305 |
| RP11-386I14.4 | 0.70478 | 1.779145 | 3.48881 | 0.000617 | 0.0019861 | -1.1005 |
| HMGCL | 0.70479 | 4.5063736 | 10.7381 | 6.96E-21 | 1.54E-18 | 36.8502 |
| NUAK2 | 0.7053 | 1.2906985 | 6.76601 | 2.02E-10 | 3.43E-09 | 13.2367 |
| SHFM1 | 0.70533 | 7.3245829 | 9.1815 | 1.38E-16 | 1.13E-14 | 27.1308 |
| FAM111B | 0.70541 | 1.3912743 | 5.58677 | 8.97E-08 | 7.72E-07 | 7.3194 |
| ARHGAP25 | 0.7057 | 2.9733192 | 6.80772 | 1.61E-10 | 2.82E-09 | 13.4584 |
| NRAS | 0.70573 | 4.3117921 | 10.2343 | 1.78E-19 | 2.87E-17 | 33.6635 |
| PAM | 0.70634 | 6.5316835 | 5.94374 | 1.53E-08 | 1.60E-07 | 9.034 |
| HOXB3 | 0.70637 | 0.7706334 | 4.1667 | 4.90E-05 | 0.0002113 | 1.27909 |
| NFE2L3 | 0.70694 | 2.0911559 | 7.37251 | 6.88E-12 | 1.78E-10 | 16.5347 |
| LGALS3BP | 0.70724 | 6.9007392 | 6.16379 | 4.95E-09 | 5.85E-08 | 10.1255 |
| ORC1 | 0.70746 | 0.9923681 | 7.54896 | 2.51E-12 | 7.37E-11 | 17.5218 |
| ST8SIA4 | 0.70752 | 1.9798492 | 7.04162 | 4.43E-11 | 9.18E-10 | 14.7163 |
| NRD1 | 0.70753 | 5.571072 | 14.4459 | 1.98E-31 | 9.64E-28 | 60.7098 |
| TRAC | 0.70793 | 1.3630187 | 5.18412 | 6.07E-07 | 4.23E-06 | 5.47423 |
| RMST | 0.70799 | 2.2941703 | 3.9356 | 0.000121 | 0.0004706 | 0.42826 |
| DERL3 | 0.70804 | 1.3167872 | 7.88772 | 3.50E-13 | 1.28E-11 | 19.4488 |
| C2orf40 | 0.70841 | 2.815846 | 3.78872 | 0.00021 | 0.0007668 | -0.0915 |
| PSMA5 | 0.70842 | 5.8282637 | 12.6188 | 3.20E-26 | 2.60E-23 | 48.9293 |
| ECE1 | 0.70909 | 4.6402126 | 6.20755 | 3.95E-09 | 4.81E-08 | 10.3456 |
| CTPS1 | 0.70915 | 3.995908 | 7.71774 | 9.44E-13 | 3.06E-11 | 18.4769 |
| RPS17L | 0.70936 | 9.065956 | 2.27296 | 0.024274 | 0.0488725 | -4.4229 |
| TMEM51 | 0.7094 | 3.1434242 | 8.12643 | 8.53E-14 | 3.67E-12 | 20.8299 |
| HMG20B | 0.70944 | 5.7282572 | 8.52999 | 7.56E-15 | 4.19E-13 | 23.2047 |
| ELF4 | 0.70964 | 1.4870798 | 7.25716 | 1.32E-11 | 3.15E-10 | 15.8958 |
| BTN2A3P | 0.70984 | 1.5014899 | 7.66459 | 1.29E-12 | 4.04E-11 | 18.175 |
| RP4-798A10.7 | 0.71018 | 2.1713077 | 5.93382 | 1.60E-08 | 1.67E-07 | 8.98541 |
| GNA15 | 0.71056 | 2.2169581 | 6.55705 | 6.25E-10 | 9.25E-09 | 12.1375 |
| IL1R1 | 0.71071 | 2.1815162 | 4.84917 | 2.77E-06 | 1.64E-05 | 4.01605 |
| GGH | 0.71072 | 3.8507372 | 7.19577 | 1.87E-11 | 4.29E-10 | 15.5579 |
| SOAT1 | 0.71074 | 3.7374404 | 7.10771 | 3.07E-11 | 6.59E-10 | 15.0759 |
| RHOH | 0.71091 | 1.3414514 | 7.23072 | 1.54E-11 | 3.60E-10 | 15.7501 |
| WISP1 | 0.71108 | 1.0850527 | 5.33873 | 2.95E-07 | 2.22E-06 | 6.17117 |
| ZCCHC11 | 0.71114 | 4.7410641 | 8.87205 | 9.36E-16 | 6.54E-14 | 25.253 |
| FHL3 | 0.71121 | 3.9028587 | 7.08485 | 3.48E-11 | 7.39E-10 | 14.9513 |
| CACHD1 | 0.71129 | 3.508575 | 7.50762 | 3.18E-12 | 9.06E-11 | 17.2895 |
| MYZAP | 0.71144 | 1.0834549 | 5.3226 | 3.18E-07 | 2.38E-06 | 6.09778 |
| SLFN12 | 0.71176 | 1.6747932 | 7.17086 | 2.15E-11 | 4.88E-10 | 15.4212 |
| RPAP2 | 0.71183 | 2.932748 | 10.1349 | 3.36E-19 | 4.99E-17 | 33.0389 |
| RP11-524D16__A.3 | 0.71215 | 1.0719478 | 4.59054 | 8.53E-06 | 4.50E-05 | 2.9407 |
| RP1-43E13.2 | 0.71252 | 2.0297376 | 7.11866 | 2.88E-11 | 6.25E-10 | 15.1357 |
| CTB-25B13.12 | 0.71259 | 2.5481738 | 7.44673 | 4.51E-12 | 1.22E-10 | 16.9484 |
| OAS2 | 0.7127 | 2.9833626 | 4.75382 | 4.22E-06 | 2.38E-05 | 3.61438 |
| TTK | 0.71287 | 1.3809134 | 5.59957 | 8.42E-08 | 7.30E-07 | 7.37965 |
| GSX2 | 0.71341 | 0.9256389 | 4.49001 | 1.31E-05 | 6.57E-05 | 2.53508 |
| AMIGO2 | 0.71388 | 1.7665529 | 5.80668 | 3.04E-08 | 2.94E-07 | 8.36729 |
| RP3-428L16.2 | 0.71409 | 3.1451377 | 4.50754 | 1.21E-05 | 6.14E-05 | 2.60528 |
| OSCAR | 0.71413 | 1.8137174 | 6.30481 | 2.38E-09 | 3.08E-08 | 10.8382 |
| PRPF38B | 0.71424 | 4.6903377 | 10.4429 | 4.67E-20 | 8.67E-18 | 34.979 |
| RP1-20B21.4 | 0.71449 | 1.0513983 | 4.43608 | 1.64E-05 | 8.00E-05 | 2.32036 |
| WASF2 | 0.71464 | 5.1609194 | 7.87078 | 3.86E-13 | 1.40E-11 | 19.3515 |
| ATAD3A | 0.7151 | 4.0457614 | 10.1229 | 3.63E-19 | 5.32E-17 | 32.9634 |
| SDF4 | 0.7155 | 6.3237537 | 11.6346 | 2.04E-23 | 7.74E-21 | 42.5838 |
| ZZZ3 | 0.71552 | 4.1096938 | 10.6891 | 9.55E-21 | 2.04E-18 | 36.5386 |
| LTBP4 | 0.71571 | 5.5075431 | 4.47205 | 1.41E-05 | 7.03E-05 | 2.46333 |
| E2F2 | 0.71574 | 0.9623458 | 6.20499 | 4.00E-09 | 4.87E-08 | 10.3327 |
| PIK3R5 | 0.71599 | 2.6155311 | 6.3705 | 1.68E-09 | 2.26E-08 | 11.1736 |
| AC064875.2 | 0.71644 | 0.8053663 | 4.99007 | 1.48E-06 | 9.37E-06 | 4.62067 |
| FAM115C | 0.71651 | 1.010876 | 6.42698 | 1.25E-09 | 1.72E-08 | 11.4636 |
| DOCK8 | 0.71693 | 2.8537842 | 6.12412 | 6.08E-09 | 7.04E-08 | 9.92683 |
| DDX20 | 0.71799 | 2.6938141 | 11.8727 | 4.29E-24 | 1.97E-21 | 44.1158 |
| CEP135 | 0.71843 | 1.7545609 | 9.59119 | 1.06E-17 | 1.14E-15 | 29.649 |
| RAB42 | 0.71878 | 1.4144964 | 5.60339 | 8.27E-08 | 7.19E-07 | 7.39765 |
| SH3TC1 | 0.71887 | 3.3829804 | 6.00642 | 1.11E-08 | 1.20E-07 | 9.34232 |
| NDC1 | 0.71912 | 2.8108082 | 10.8095 | 4.38E-21 | 1.06E-18 | 37.3043 |
| SLC34A2 | 0.71984 | 0.6237857 | 7.39091 | 6.20E-12 | 1.62E-10 | 16.6371 |
| C1orf63 | 0.71985 | 5.6877371 | 8.64565 | 3.74E-15 | 2.25E-13 | 23.8939 |
| TRAF3IP3 | 0.71999 | 2.2617786 | 7.03815 | 4.52E-11 | 9.32E-10 | 14.6974 |
| IL1RAP | 0.72043 | 3.0764693 | 4.29768 | 2.89E-05 | 0.0001323 | 1.77885 |
| TLR5 | 0.72056 | 2.0724285 | 6.38888 | 1.53E-09 | 2.06E-08 | 11.2678 |
| CDC42 | 0.72068 | 6.9741986 | 11.852 | 4.91E-24 | 2.21E-21 | 43.9824 |
| C1orf53 | 0.72093 | 2.3075469 | 6.74582 | 2.26E-10 | 3.77E-09 | 13.1296 |
| TTF2 | 0.72101 | 2.4379193 | 8.57654 | 5.70E-15 | 3.29E-13 | 23.4817 |
| ASIP | 0.72127 | 1.2240497 | 6.02869 | 9.91E-09 | 1.09E-07 | 9.45234 |
| TMEM67 | 0.72155 | 3.0406259 | 7.68483 | 1.14E-12 | 3.63E-11 | 18.2898 |
| NCF1C | 0.72156 | 1.5633064 | 6.4347 | 1.20E-09 | 1.66E-08 | 11.5034 |
| TGFB1 | 0.72201 | 4.7075834 | 6.26016 | 3.00E-09 | 3.79E-08 | 10.6114 |
| HMGB2 | 0.7222 | 6.0968002 | 6.8012 | 1.67E-10 | 2.91E-09 | 13.4237 |
| NASP | 0.72239 | 6.3092148 | 9.21536 | 1.12E-16 | 9.38E-15 | 27.3376 |
| SPATS2L | 0.72253 | 5.4740129 | 8.14306 | 7.73E-14 | 3.38E-12 | 20.9268 |
| CCDC23 | 0.72318 | 4.8459613 | 10.4199 | 5.42E-20 | 9.76E-18 | 34.8336 |
| HOXD4 | 0.72342 | 0.9640406 | 5.09225 | 9.27E-07 | 6.16E-06 | 5.06715 |
| ATL3 | 0.72373 | 3.5869735 | 7.91978 | 2.90E-13 | 1.08E-11 | 19.6332 |
| CD300LF | 0.72401 | 1.6576033 | 5.9696 | 1.34E-08 | 1.42E-07 | 9.16095 |
| MSMP | 0.72409 | 0.8945352 | 4.09597 | 6.48E-05 | 0.0002707 | 1.01447 |
| PTAFR | 0.7242 | 2.7304067 | 5.47245 | 1.56E-07 | 1.25E-06 | 6.78565 |
| ANGPT2 | 0.72448 | 2.3694438 | 3.65989 | 0.000336 | 0.0011618 | -0.5336 |
| GATM | 0.72478 | 6.3897256 | 3.67692 | 0.000316 | 0.0011013 | -0.4759 |
| TMEM234 | 0.72502 | 3.5029531 | 11.5146 | 4.46E-23 | 1.53E-20 | 41.8126 |
| NR4A3 | 0.7254 | 1.7199081 | 4.80147 | 3.42E-06 | 1.98E-05 | 3.81433 |
| CENPW | 0.72557 | 2.0168416 | 6.00691 | 1.11E-08 | 1.20E-07 | 9.34472 |
| SLC44A5 | 0.72557 | 2.5258247 | 4.57538 | 9.10E-06 | 4.76E-05 | 2.87911 |
| APOL2 | 0.72602 | 5.2343232 | 6.09159 | 7.18E-09 | 8.18E-08 | 9.76456 |
| RNF19B | 0.7264 | 3.7450945 | 8.7617 | 1.84E-15 | 1.18E-13 | 24.5889 |
| EDNRA | 0.72667 | 3.0433359 | 4.63311 | 7.11E-06 | 3.82E-05 | 3.11457 |
| CDH11 | 0.72668 | 5.1468121 | 5.63583 | 7.06E-08 | 6.25E-07 | 7.55081 |
| CHPF2 | 0.72681 | 3.9446421 | 8.48168 | 1.01E-14 | 5.44E-13 | 22.9179 |
| CRISPLD1 | 0.72742 | 4.7550145 | 4.03637 | 8.18E-05 | 0.0003338 | 0.79435 |
| CLECL1 | 0.72771 | 1.1236294 | 6.5924 | 5.17E-10 | 7.83E-09 | 12.322 |
| DUSP10 | 0.72788 | 2.5913309 | 6.71001 | 2.74E-10 | 4.47E-09 | 12.9401 |
| POLE4 | 0.72815 | 5.472337 | 7.25384 | 1.35E-11 | 3.20E-10 | 15.8774 |
| FZD7 | 0.72863 | 3.0194765 | 4.34657 | 2.37E-05 | 0.000111 | 1.96859 |
| SLC25A19 | 0.72871 | 3.1652598 | 8.4961 | 9.28E-15 | 5.07E-13 | 23.0035 |
| MREG | 0.72904 | 2.9853744 | 5.87572 | 2.15E-08 | 2.17E-07 | 8.70186 |
| TRIM38 | 0.72924 | 1.9833866 | 7.70607 | 1.01E-12 | 3.26E-11 | 18.4105 |
| C4orf47 | 0.72953 | 2.115346 | 5.20493 | 5.51E-07 | 3.88E-06 | 5.56718 |
| IFNGR2 | 0.72977 | 5.2650177 | 8.37172 | 1.97E-14 | 9.80E-13 | 22.2677 |
| IRX1 | 0.73013 | 2.1701085 | 2.98154 | 0.003287 | 0.0085941 | -2.6408 |
| TAF12 | 0.73016 | 4.0341608 | 13.307 | 3.47E-28 | 6.03E-25 | 53.3756 |
| SH3BGRL3 | 0.73019 | 6.522324 | 8.2206 | 4.87E-14 | 2.20E-12 | 21.3798 |
| NEK6 | 0.73045 | 5.0058554 | 6.019 | 1.04E-08 | 1.14E-07 | 9.40443 |
| ZNF518B | 0.73117 | 2.3351106 | 6.3627 | 1.75E-09 | 2.34E-08 | 11.1336 |
| CD109 | 0.73152 | 1.4502052 | 6.76957 | 1.98E-10 | 3.38E-09 | 13.2556 |
| SZRD1 | 0.73155 | 5.625992 | 9.24522 | 9.28E-17 | 7.87E-15 | 27.5202 |
| DEPDC1 | 0.73162 | 0.7714681 | 7.4498 | 4.43E-12 | 1.21E-10 | 16.9656 |
| DLEC1 | 0.7319 | 1.5441765 | 5.60893 | 8.05E-08 | 7.03E-07 | 7.42373 |
| IGFBP4 | 0.73191 | 4.7674204 | 4.48235 | 1.35E-05 | 6.76E-05 | 2.50443 |
| IGKV4-1 | 0.73234 | 0.7944348 | 4.00104 | 9.38E-05 | 0.0003763 | 0.66513 |
| KIAA2013 | 0.73237 | 4.7863604 | 10.4085 | 5.83E-20 | 1.04E-17 | 34.7614 |
| ECT2 | 0.73262 | 2.7978049 | 6.23307 | 3.46E-09 | 4.29E-08 | 10.4744 |
| HOXC10 | 0.73324 | 0.9295332 | 3.70382 | 0.000286 | 0.0010104 | -0.3843 |
| TMEM255B | 0.73331 | 1.9902361 | 7.02286 | 4.92E-11 | 1.00E-09 | 14.6145 |
| XAF1 | 0.73398 | 4.0262147 | 5.12523 | 7.97E-07 | 5.39E-06 | 5.21266 |
| SIPA1 | 0.73404 | 4.4338925 | 7.1627 | 2.25E-11 | 5.06E-10 | 15.3765 |
| IER3 | 0.73422 | 3.5828778 | 3.659 | 0.000337 | 0.0011652 | -0.5366 |
| NMRAL1 | 0.73422 | 4.8148856 | 5.61895 | 7.66E-08 | 6.72E-07 | 7.47104 |
| CD3D | 0.73438 | 1.0745359 | 5.89291 | 1.97E-08 | 2.01E-07 | 8.78553 |
| AC002398.9 | 0.73446 | 2.4626381 | 5.40447 | 2.16E-07 | 1.68E-06 | 6.47194 |
| C1orf226 | 0.7345 | 3.278978 | 5.22632 | 4.99E-07 | 3.55E-06 | 5.66299 |
| PLK1 | 0.7345 | 2.6899783 | 5.30676 | 3.43E-07 | 2.54E-06 | 6.02587 |
| GAS1 | 0.73455 | 3.3437238 | 4.42244 | 1.73E-05 | 8.40E-05 | 2.2664 |
| AGBL2 | 0.73485 | 1.3571602 | 6.12702 | 5.99E-09 | 6.95E-08 | 9.94135 |
| TMEM106C | 0.73487 | 5.3963599 | 7.60555 | 1.81E-12 | 5.51E-11 | 17.8409 |
| RAD51 | 0.7353 | 1.6910913 | 6.22396 | 3.62E-09 | 4.47E-08 | 10.4283 |
| NFKB2 | 0.73568 | 3.2407801 | 7.83274 | 4.83E-13 | 1.71E-11 | 19.1333 |
| C4A-AS1 | 0.73617 | 1.3429206 | 6.51768 | 7.71E-10 | 1.12E-08 | 11.9327 |
| C4B-AS1 | 0.73617 | 1.3429206 | 6.51768 | 7.71E-10 | 1.12E-08 | 11.9327 |
| KLHL4 | 0.73651 | 2.679504 | 4.72419 | 4.80E-06 | 2.67E-05 | 3.49077 |
| EN2 | 0.73657 | 0.8163808 | 7.52497 | 2.88E-12 | 8.32E-11 | 17.387 |
| CXCL9 | 0.73692 | 0.9315016 | 5.27645 | 3.95E-07 | 2.89E-06 | 5.88868 |
| TRNAU1AP | 0.73693 | 3.969548 | 11.9629 | 2.37E-24 | 1.23E-21 | 44.6969 |
| TRIT1 | 0.73714 | 3.8538685 | 9.27445 | 7.74E-17 | 6.82E-15 | 27.6991 |
| GS1-114I9.1 | 0.73765 | 1.5304661 | 6.33685 | 2.01E-09 | 2.65E-08 | 11.0015 |
| ARPC5 | 0.73791 | 6.335345 | 8.95099 | 5.75E-16 | 4.27E-14 | 25.7299 |
| CCDC37 | 0.73792 | 0.8487771 | 4.53437 | 1.08E-05 | 5.55E-05 | 2.71319 |
| KPNA2 | 0.73808 | 5.1085231 | 8.08027 | 1.12E-13 | 4.75E-12 | 20.5614 |
| LRAT | 0.73814 | 1.3692957 | 5.27097 | 4.05E-07 | 2.95E-06 | 5.86392 |
| MCAM | 0.73897 | 4.9165209 | 4.64044 | 6.89E-06 | 3.71E-05 | 3.14466 |
| EXOSC10 | 0.73951 | 5.1345616 | 11.8059 | 6.64E-24 | 2.89E-21 | 43.6853 |
| SMS | 0.73979 | 5.404086 | 7.8335 | 4.80E-13 | 1.70E-11 | 19.1377 |
| OR2I1P | 0.74063 | 0.7366784 | 5.52516 | 1.21E-07 | 9.97E-07 | 7.03082 |
| IGJ | 0.74078 | 1.1614118 | 3.79616 | 0.000204 | 0.0007486 | -0.0656 |
| SPA17 | 0.7408 | 3.533145 | 6.66176 | 3.56E-10 | 5.62E-09 | 12.6858 |
| RP13-401N8.3 | 0.74087 | 3.3566057 | 4.17673 | 4.71E-05 | 0.0002037 | 1.31693 |
| SF3A3 | 0.74123 | 5.58087 | 10.169 | 2.71E-19 | 4.11E-17 | 33.253 |
| CYTIP | 0.7415 | 1.8183351 | 6.8244 | 1.47E-10 | 2.61E-09 | 13.5474 |
| ARAP3 | 0.74153 | 2.5484401 | 5.24597 | 4.56E-07 | 3.27E-06 | 5.75127 |
| CA9 | 0.74183 | 1.3089725 | 3.53136 | 0.000532 | 0.0017415 | -0.9617 |
| ARHGAP6 | 0.74203 | 2.4177097 | 5.9311 | 1.63E-08 | 1.69E-07 | 8.9721 |
| SVIL | 0.74214 | 2.1386254 | 5.94031 | 1.55E-08 | 1.62E-07 | 9.01718 |
| CNPY4 | 0.74261 | 4.4699879 | 8.81479 | 1.33E-15 | 8.94E-14 | 24.908 |
| GFPT2 | 0.74261 | 4.2617933 | 4.93222 | 1.91E-06 | 1.18E-05 | 4.37085 |
| HIST1H2AC | 0.74328 | 4.3279857 | 5.77673 | 3.52E-08 | 3.36E-07 | 8.22295 |
| PKN2 | 0.74371 | 3.7793832 | 9.87949 | 1.71E-18 | 2.15E-16 | 31.4402 |
| OCIAD2 | 0.7442 | 3.6843168 | 3.45392 | 0.000697 | 0.0022069 | -1.2132 |
| ERP27 | 0.74428 | 0.7525294 | 7.53278 | 2.75E-12 | 8.00E-11 | 17.4308 |
| SKAP2 | 0.74452 | 3.2564549 | 5.03383 | 1.21E-06 | 7.84E-06 | 4.81103 |
| IL8 | 0.74491 | 1.8304043 | 2.97457 | 0.003359 | 0.008755 | -2.6605 |
| SERBP1 | 0.7451 | 6.7865773 | 10.9997 | 1.28E-21 | 3.61E-19 | 38.5162 |
| PHF13 | 0.74523 | 3.2604732 | 11.4265 | 7.94E-23 | 2.64E-20 | 41.2472 |
| RAP1A | 0.74523 | 5.8684484 | 10.5237 | 2.78E-20 | 5.23E-18 | 35.4903 |
| SLA | 0.74546 | 3.5796787 | 5.2842 | 3.81E-07 | 2.79E-06 | 5.9237 |
| SCPEP1 | 0.74562 | 5.1812198 | 7.95537 | 2.35E-13 | 8.98E-12 | 19.8383 |
| LAMTOR5 | 0.74597 | 7.0904509 | 12.9841 | 2.90E-27 | 3.36E-24 | 51.2893 |
| SRSF11 | 0.74603 | 6.5430568 | 9.27081 | 7.91E-17 | 6.95E-15 | 27.6768 |
| LAP3 | 0.74622 | 6.1715072 | 7.4633 | 4.10E-12 | 1.13E-10 | 17.0412 |
| CENPH | 0.7464 | 2.9696591 | 6.43454 | 1.20E-09 | 1.66E-08 | 11.5026 |
| ITGA4 | 0.7465 | 1.7595601 | 5.32511 | 3.14E-07 | 2.35E-06 | 6.10918 |
| RGS19 | 0.74667 | 3.9821677 | 7.97164 | 2.13E-13 | 8.26E-12 | 19.9322 |
| MNS1 | 0.74708 | 2.6858815 | 8.01558 | 1.65E-13 | 6.52E-12 | 20.1863 |
| ADPRH | 0.74729 | 2.0430612 | 7.73913 | 8.34E-13 | 2.73E-11 | 18.5986 |
| PPIH | 0.7474 | 4.8061973 | 12.3246 | 2.21E-25 | 1.38E-22 | 47.0295 |
| PHC2 | 0.74747 | 6.4932086 | 9.183 | 1.37E-16 | 1.13E-14 | 27.14 |
| STK38 | 0.74773 | 3.4917082 | 10.3644 | 7.73E-20 | 1.35E-17 | 34.4834 |
| DR1 | 0.74777 | 4.3339983 | 10.5361 | 2.56E-20 | 4.87E-18 | 35.5687 |
| SPEF1 | 0.74813 | 1.8400284 | 5.08688 | 9.51E-07 | 6.29E-06 | 5.04348 |
| GNGT2 | 0.74813 | 1.8753315 | 7.4075 | 5.64E-12 | 1.49E-10 | 16.7295 |
| PBK | 0.74845 | 2.4568849 | 3.78921 | 0.000209 | 0.0007657 | -0.0898 |
| TMX1 | 0.74999 | 4.8318161 | 7.68534 | 1.14E-12 | 3.63E-11 | 18.2928 |
| CFD | 0.75003 | 2.503995 | 5.10197 | 8.87E-07 | 5.91E-06 | 5.10996 |
| MX2 | 0.7506 | 2.3006227 | 5.01042 | 1.35E-06 | 8.64E-06 | 4.70903 |
| CXorf21 | 0.75063 | 1.4973375 | 7.08575 | 3.47E-11 | 7.36E-10 | 14.9562 |
| DMRTA2 | 0.75111 | 0.8532137 | 4.63514 | 7.05E-06 | 3.79E-05 | 3.12293 |
| CSF1R | 0.75116 | 5.7527655 | 5.07065 | 1.02E-06 | 6.74E-06 | 4.97219 |
| CD151 | 0.75136 | 6.2374097 | 6.72674 | 2.50E-10 | 4.13E-09 | 13.0286 |
| JAK3 | 0.75163 | 1.4519743 | 6.25869 | 3.03E-09 | 3.81E-08 | 10.604 |
| RPF1 | 0.75223 | 4.6231502 | 13.2048 | 6.79E-28 | 1.10E-24 | 52.7155 |
| CAV2 | 0.75226 | 3.1747279 | 5.01549 | 1.32E-06 | 8.46E-06 | 4.73112 |
| TMCO4 | 0.75226 | 2.4420757 | 7.76762 | 7.06E-13 | 2.37E-11 | 18.761 |
| C1orf228 | 0.75231 | 2.7092351 | 6.45877 | 1.06E-09 | 1.48E-08 | 11.6276 |
| TUBA1C | 0.75238 | 4.7342671 | 4.00571 | 9.21E-05 | 0.0003703 | 0.68214 |
| DPY19L1 | 0.75267 | 4.4497614 | 6.67859 | 3.25E-10 | 5.19E-09 | 12.7744 |
| OASL | 0.7529 | 1.5391014 | 5.43253 | 1.89E-07 | 1.49E-06 | 6.60111 |
| CD3E | 0.75335 | 1.2452651 | 5.82978 | 2.70E-08 | 2.66E-07 | 8.47895 |
| EXTL2 | 0.75341 | 3.9548138 | 10.6159 | 1.53E-20 | 3.03E-18 | 36.0745 |
| DSE | 0.75405 | 3.0356082 | 7.6849 | 1.14E-12 | 3.63E-11 | 18.2902 |
| SHOX2 | 0.75408 | 0.8749023 | 4.16125 | 5.01E-05 | 0.0002153 | 1.25858 |
| CORO1A | 0.75451 | 5.4050667 | 6.84482 | 1.31E-10 | 2.36E-09 | 13.6564 |
| AC008738.1 | 0.75454 | 2.9804301 | 4.35992 | 2.24E-05 | 0.0001059 | 2.0207 |
| NKG7 | 0.75456 | 1.7271822 | 5.49378 | 1.41E-07 | 1.14E-06 | 6.88469 |
| CX3CR1 | 0.75465 | 4.7983104 | 3.45045 | 0.000705 | 0.0022287 | -1.2243 |
| TLR7 | 0.75502 | 2.1346355 | 5.93919 | 1.56E-08 | 1.63E-07 | 9.01172 |
| SEL1L3 | 0.75611 | 2.815014 | 4.29309 | 2.95E-05 | 0.0001346 | 1.76114 |
| WDR38 | 0.75613 | 0.7686771 | 5.59375 | 8.67E-08 | 7.49E-07 | 7.35226 |
| RASAL3 | 0.75629 | 2.4066381 | 6.78146 | 1.86E-10 | 3.19E-09 | 13.3187 |
| BACE2 | 0.75632 | 3.0282461 | 5.43384 | 1.87E-07 | 1.48E-06 | 6.60716 |
| CMTM6 | 0.75651 | 4.104453 | 8.54576 | 6.87E-15 | 3.82E-13 | 23.2984 |
| DCBLD2 | 0.75726 | 3.1284916 | 7.23026 | 1.54E-11 | 3.60E-10 | 15.7475 |
| IGKV3-11 | 0.75728 | 0.7554051 | 3.70583 | 0.000284 | 0.0010038 | -0.3774 |
| MAP7D3 | 0.7575 | 2.1531555 | 9.57952 | 1.14E-17 | 1.20E-15 | 29.5768 |
| IL1RN | 0.75754 | 1.2339332 | 4.49307 | 1.29E-05 | 6.49E-05 | 2.54733 |
| HTRA3 | 0.75793 | 1.0919747 | 5.39005 | 2.31E-07 | 1.78E-06 | 6.40572 |
| BATF | 0.75824 | 1.0729657 | 6.84559 | 1.31E-10 | 2.36E-09 | 13.6605 |
| ITGB1 | 0.75893 | 6.2521436 | 5.96929 | 1.34E-08 | 1.42E-07 | 9.15944 |
| F3 | 0.75905 | 5.5760977 | 4.12874 | 5.69E-05 | 0.000241 | 1.1366 |
| TNFRSF14 | 0.75917 | 3.8073996 | 6.58171 | 5.48E-10 | 8.22E-09 | 12.2661 |
| IGHG4 | 0.7593 | 0.7157356 | 4.2731 | 3.19E-05 | 0.0001449 | 1.6841 |
| TEAD2 | 0.75956 | 2.5510791 | 5.66278 | 6.18E-08 | 5.57E-07 | 7.67847 |
| SDC1 | 0.7597 | 1.6092514 | 5.7318 | 4.40E-08 | 4.11E-07 | 8.00739 |
| MAP3K19 | 0.7597 | 0.9790695 | 4.9314 | 1.92E-06 | 1.19E-05 | 4.36735 |
| FCGRT | 0.75986 | 6.2954979 | 7.30254 | 1.02E-11 | 2.52E-10 | 16.1465 |
| TRPM8 | 0.76009 | 0.9182732 | 5.17549 | 6.32E-07 | 4.38E-06 | 5.43576 |
| ABRACL | 0.7603 | 3.4508097 | 6.61693 | 4.53E-10 | 6.94E-09 | 12.4504 |
| SPTSSB | 0.7604 | 1.6568905 | 4.9577 | 1.71E-06 | 1.07E-05 | 4.48061 |
| MAP3K6 | 0.7609 | 3.1671522 | 6.01741 | 1.05E-08 | 1.14E-07 | 9.39658 |
| ZFP36L2 | 0.76166 | 5.7633953 | 6.71787 | 2.63E-10 | 4.31E-09 | 12.9817 |
| TGFBR1 | 0.76167 | 4.1622213 | 6.8998 | 9.72E-11 | 1.81E-09 | 13.9508 |
| HOXB7 | 0.76196 | 1.3937028 | 4.22395 | 3.90E-05 | 0.0001724 | 1.496 |
| CROCC | 0.7622 | 3.7801747 | 8.79886 | 1.47E-15 | 9.72E-14 | 24.8122 |
| MNDA | 0.76231 | 3.3414383 | 5.5636 | 1.00E-07 | 8.51E-07 | 7.21061 |
| SLC39A1 | 0.76238 | 5.6856184 | 8.72367 | 2.32E-15 | 1.46E-13 | 24.3608 |
| SIX1 | 0.76243 | 1.8525245 | 4.46899 | 1.43E-05 | 7.10E-05 | 2.45114 |
| LSM10 | 0.76256 | 5.2468707 | 12.5414 | 5.32E-26 | 3.81E-23 | 48.4297 |
| UBA7 | 0.76257 | 5.1743994 | 6.79448 | 1.73E-10 | 3.01E-09 | 13.388 |
| TRH | 0.76297 | 1.4958338 | 3.21925 | 0.001538 | 0.0044488 | -1.9456 |
| HOXA-AS2 | 0.76322 | 0.5318351 | 4.93843 | 1.86E-06 | 1.15E-05 | 4.39758 |
| INMT | 0.76347 | 1.1190989 | 5.28121 | 3.86E-07 | 2.83E-06 | 5.91018 |
| UBXN11 | 0.76385 | 4.3759513 | 7.95603 | 2.34E-13 | 8.96E-12 | 19.8421 |
| SCN7A | 0.76389 | 0.9299584 | 5.80433 | 3.07E-08 | 2.97E-07 | 8.35593 |
| ZNF560 | 0.76435 | 1.1752325 | 4.83573 | 2.94E-06 | 1.73E-05 | 3.95908 |
| SERPINF1 | 0.76454 | 4.7115955 | 5.02483 | 1.26E-06 | 8.14E-06 | 4.77178 |
| ARHGAP15 | 0.76472 | 2.3051197 | 7.06752 | 3.84E-11 | 8.06E-10 | 14.857 |
| HK3 | 0.7652 | 1.7073335 | 5.93943 | 1.56E-08 | 1.63E-07 | 9.01288 |
| CSF1 | 0.76586 | 4.8358341 | 6.40273 | 1.42E-09 | 1.92E-08 | 11.3389 |
| OSBPL9 | 0.76623 | 4.7608836 | 10.5519 | 2.32E-20 | 4.47E-18 | 35.6687 |
| ADAP2 | 0.76627 | 3.6417643 | 6.28643 | 2.62E-09 | 3.35E-08 | 10.7447 |
| SIX5 | 0.7663 | 1.9847164 | 8.05651 | 1.29E-13 | 5.33E-12 | 20.4235 |
| HCP5 | 0.7664 | 2.5550821 | 5.45725 | 1.68E-07 | 1.34E-06 | 6.71526 |
| PSENEN | 0.76658 | 5.9938799 | 9.53909 | 1.47E-17 | 1.54E-15 | 29.3269 |
| SECTM1 | 0.76706 | 1.9647533 | 5.58925 | 8.86E-08 | 7.63E-07 | 7.33106 |
| IGSF6 | 0.76788 | 3.5284589 | 5.60876 | 8.06E-08 | 7.03E-07 | 7.42294 |
| RAD54L | 0.76829 | 1.6527557 | 7.81731 | 5.28E-13 | 1.85E-11 | 19.045 |
| REXO2 | 0.76842 | 5.0738325 | 10.364 | 7.76E-20 | 1.35E-17 | 34.4804 |
| SIGLEC7 | 0.76869 | 1.7487854 | 6.55855 | 6.20E-10 | 9.18E-09 | 12.1453 |
| TBXAS1 | 0.76872 | 4.0889838 | 6.42251 | 1.28E-09 | 1.75E-08 | 11.4406 |
| C1orf54 | 0.76923 | 4.5324788 | 6.32219 | 2.17E-09 | 2.84E-08 | 10.9267 |
| MINOS1 | 0.76939 | 6.1779477 | 8.57348 | 5.80E-15 | 3.34E-13 | 23.4634 |
| NFIL3 | 0.77059 | 3.9596676 | 7.18281 | 2.01E-11 | 4.58E-10 | 15.4868 |
| IER5L | 0.7709 | 3.0605326 | 5.33142 | 3.05E-07 | 2.29E-06 | 6.13789 |
| EZH2 | 0.77103 | 2.9944251 | 4.95666 | 1.72E-06 | 1.07E-05 | 4.47611 |
| CKS2 | 0.77197 | 4.5105516 | 5.36732 | 2.57E-07 | 1.97E-06 | 6.30166 |
| YBX3 | 0.77286 | 5.2746885 | 5.70234 | 5.09E-08 | 4.69E-07 | 7.8667 |
| MATN2 | 0.77303 | 5.502933 | 4.57207 | 9.23E-06 | 4.81E-05 | 2.86566 |
| GZMA | 0.77328 | 1.1632099 | 5.39231 | 2.29E-07 | 1.77E-06 | 6.41609 |
| FSBP | 0.77331 | 1.5251805 | 8.01332 | 1.67E-13 | 6.58E-12 | 20.1732 |
| PLB1 | 0.77345 | 2.1640668 | 6.83833 | 1.36E-10 | 2.44E-09 | 13.6217 |
| ADAMTSL4 | 0.77356 | 1.4794763 | 6.91669 | 8.85E-11 | 1.67E-09 | 14.0415 |
| AVIL | 0.77406 | 2.7391158 | 5.64425 | 6.77E-08 | 6.03E-07 | 7.59066 |
| BLNK | 0.77412 | 2.5750835 | 5.72038 | 4.66E-08 | 4.32E-07 | 7.95278 |
| NFATC1 | 0.77456 | 2.4517759 | 7.15489 | 2.35E-11 | 5.27E-10 | 15.3337 |
| TRDC | 0.77479 | 1.0216653 | 4.76804 | 3.96E-06 | 2.26E-05 | 3.67388 |
| LYPLA2 | 0.77529 | 4.782177 | 12.6598 | 2.44E-26 | 2.06E-23 | 49.1942 |
| PODNL1 | 0.77687 | 1.366832 | 5.67371 | 5.86E-08 | 5.31E-07 | 7.73038 |
| KYNU | 0.77701 | 1.3768674 | 7.14978 | 2.42E-11 | 5.38E-10 | 15.3058 |
| DARC | 0.77714 | 2.7160322 | 4.8797 | 2.42E-06 | 1.46E-05 | 4.14596 |
| H6PD | 0.77726 | 3.2834209 | 9.78386 | 3.14E-18 | 3.75E-16 | 30.8445 |
| MXRA5 | 0.77841 | 1.2817932 | 5.62328 | 7.50E-08 | 6.59E-07 | 7.49148 |
| CDCA3 | 0.77878 | 2.8046398 | 6.07472 | 7.83E-09 | 8.83E-08 | 9.68064 |
| TRAPPC3 | 0.77878 | 5.6741491 | 12.8646 | 6.36E-27 | 6.45E-24 | 50.5173 |
| RAD54B | 0.77947 | 2.01055 | 9.07368 | 2.70E-16 | 2.12E-14 | 26.474 |
| MAD2L2 | 0.77957 | 5.6486999 | 7.55842 | 2.37E-12 | 7.03E-11 | 17.5751 |
| FBP1 | 0.77981 | 1.929687 | 6.2343 | 3.44E-09 | 4.27E-08 | 10.4806 |
| SNX20 | 0.78013 | 1.3500295 | 7.07307 | 3.72E-11 | 7.85E-10 | 14.8872 |
| LINC00339 | 0.78015 | 3.1825062 | 12.1409 | 7.39E-25 | 3.99E-22 | 45.8444 |
| SPATA1 | 0.78035 | 1.0121461 | 8.82756 | 1.23E-15 | 8.36E-14 | 24.9848 |
| AURKA | 0.78084 | 2.0157321 | 6.65405 | 3.71E-10 | 5.82E-09 | 12.6452 |
| HSD3B7 | 0.78117 | 2.5118646 | 7.43632 | 4.78E-12 | 1.29E-10 | 16.8903 |
| PLCB2 | 0.78141 | 4.1621653 | 6.82294 | 1.48E-10 | 2.62E-09 | 13.5396 |
| NCF1B | 0.78194 | 1.6750638 | 7.42127 | 5.21E-12 | 1.39E-10 | 16.8063 |
| TNFRSF1B | 0.78208 | 3.5450922 | 6.57731 | 5.61E-10 | 8.38E-09 | 12.2432 |
| DOCK2 | 0.78214 | 2.9613417 | 6.8345 | 1.39E-10 | 2.49E-09 | 13.6013 |
| NPHP1 | 0.78222 | 2.6839575 | 9.70177 | 5.28E-18 | 6.09E-16 | 30.3343 |
| TPST1 | 0.78236 | 5.223035 | 6.92972 | 8.24E-11 | 1.57E-09 | 14.1115 |
| RAB13 | 0.78301 | 6.6276614 | 7.12003 | 2.86E-11 | 6.21E-10 | 15.1431 |
| LMNB1 | 0.78301 | 3.9615575 | 4.82441 | 3.09E-06 | 1.81E-05 | 3.91116 |
| PSMB8 | 0.78375 | 5.4307057 | 6.56645 | 5.94E-10 | 8.84E-09 | 12.1865 |
| MYO7A | 0.78396 | 2.3413447 | 8.39117 | 1.75E-14 | 8.86E-13 | 22.3825 |
| HLA-DQA2 | 0.78437 | 1.1252447 | 4.55819 | 9.79E-06 | 5.08E-05 | 2.80943 |
| SRSF4 | 0.78504 | 5.7339881 | 13.5358 | 7.72E-29 | 1.73E-25 | 54.8522 |
| ZDHHC12 | 0.78504 | 3.2654507 | 7.20299 | 1.80E-11 | 4.14E-10 | 15.5975 |
| COL14A1 | 0.78517 | 1.6764919 | 5.1572 | 6.88E-07 | 4.73E-06 | 5.3544 |
| SYK | 0.7853 | 2.9069881 | 6.45616 | 1.07E-09 | 1.49E-08 | 11.6141 |
| AC011558.5 | 0.78583 | 2.2357827 | 3.60482 | 0.00041 | 0.001384 | -0.7186 |
| HAS2-AS1 | 0.78593 | 1.7321394 | 6.08537 | 7.41E-09 | 8.42E-08 | 9.73358 |
| BST1 | 0.78654 | 1.8957918 | 7.80666 | 5.62E-13 | 1.94E-11 | 18.9841 |
| HIST1H2BJ | 0.78671 | 1.1333918 | 7.00698 | 5.37E-11 | 1.08E-09 | 14.5285 |
| COLEC12 | 0.78716 | 3.4733895 | 5.54353 | 1.11E-07 | 9.23E-07 | 7.11664 |
| IFIH1 | 0.7879 | 2.8710224 | 7.42774 | 5.02E-12 | 1.35E-10 | 16.8424 |
| MAD2L1 | 0.78812 | 3.240887 | 6.04431 | 9.15E-09 | 1.01E-07 | 9.52968 |
| CMTM3 | 0.78917 | 5.3336995 | 6.76854 | 1.99E-10 | 3.39E-09 | 13.2501 |
| TMEM176A | 0.78949 | 4.5746162 | 4.2208 | 3.95E-05 | 0.0001744 | 1.48399 |
| EMR1 | 0.78964 | 0.929915 | 6.1353 | 5.74E-09 | 6.70E-08 | 9.98276 |
| IL13RA1 | 0.78969 | 4.2764499 | 6.35607 | 1.82E-09 | 2.42E-08 | 11.0997 |
| MINA | 0.78976 | 3.6633715 | 6.72122 | 2.58E-10 | 4.24E-09 | 12.9994 |
| SHKBP1 | 0.79001 | 4.3885555 | 9.1674 | 1.51E-16 | 1.22E-14 | 27.0448 |
| SNRNP40 | 0.79055 | 4.6184504 | 13.636 | 4.00E-29 | 1.08E-25 | 55.4985 |
| CHEK1 | 0.79058 | 2.6650497 | 6.77222 | 1.95E-10 | 3.34E-09 | 13.2696 |
| PROS1 | 0.79112 | 3.9365266 | 5.6786 | 5.72E-08 | 5.20E-07 | 7.75367 |
| LAMC1 | 0.79118 | 3.8736731 | 4.86518 | 2.58E-06 | 1.54E-05 | 4.08409 |
| PDCD5 | 0.79165 | 6.0409677 | 13.1637 | 8.90E-28 | 1.35E-24 | 52.4498 |
| LGALS3 | 0.79179 | 5.0324008 | 3.8946 | 0.000141 | 0.0005401 | 0.2815 |
| RHBDF2 | 0.79203 | 3.900786 | 5.65411 | 6.45E-08 | 5.78E-07 | 7.63737 |
| PLEKHA8P1 | 0.79204 | 2.0777161 | 7.16627 | 2.21E-11 | 4.98E-10 | 15.3961 |
| FNDC3B | 0.79204 | 3.2637355 | 7.03683 | 4.55E-11 | 9.38E-10 | 14.6903 |
| HILPDA | 0.79213 | 4.0185789 | 5.39489 | 2.26E-07 | 1.75E-06 | 6.42793 |
| IRX5 | 0.79215 | 0.8170801 | 6.60751 | 4.77E-10 | 7.27E-09 | 12.4011 |
| PPIC | 0.79234 | 2.8137651 | 6.19201 | 4.28E-09 | 5.16E-08 | 10.2673 |
| AC015987.2 | 0.79309 | 5.0007368 | 3.73089 | 0.000259 | 0.0009264 | -0.2915 |
| COL6A3 | 0.79318 | 1.5182567 | 4.41368 | 1.80E-05 | 8.68E-05 | 2.2318 |
| IGKV1D-39 | 0.79357 | 1.0483319 | 3.14012 | 0.00199 | 0.005563 | -2.1823 |
| RN7SL731P | 0.79363 | 2.5390508 | 4.36268 | 2.22E-05 | 0.0001049 | 2.03149 |
| YAP1 | 0.79369 | 4.0599672 | 5.56012 | 1.02E-07 | 8.63E-07 | 7.19432 |
| DTX3L | 0.79397 | 3.2384757 | 6.79536 | 1.72E-10 | 2.99E-09 | 13.3927 |
| FTL | 0.79417 | 11.063719 | 6.04822 | 8.97E-09 | 9.94E-08 | 9.54907 |
| TK1 | 0.79444 | 2.3036191 | 5.55493 | 1.05E-07 | 8.82E-07 | 7.16998 |
| MRPS15 | 0.79447 | 5.0645434 | 12.191 | 5.32E-25 | 2.94E-22 | 46.1673 |
| RPS6KA1 | 0.79468 | 3.5381601 | 6.89745 | 9.84E-11 | 1.83E-09 | 13.9382 |
| RELB | 0.795 | 2.7398463 | 7.3344 | 8.55E-12 | 2.14E-10 | 16.323 |
| CLSPN | 0.79525 | 1.333174 | 6.88824 | 1.04E-10 | 1.90E-09 | 13.8888 |
| RP11-421L21.3 | 0.79605 | 2.7033529 | 9.46636 | 2.33E-17 | 2.31E-15 | 28.8781 |
| RP11-698N11.2 | 0.7961 | 1.9122014 | 4.85625 | 2.69E-06 | 1.60E-05 | 4.04613 |
| KANK2 | 0.79627 | 4.390596 | 6.91594 | 8.89E-11 | 1.68E-09 | 14.0375 |
| FXYD5 | 0.79686 | 4.8387507 | 5.83884 | 2.58E-08 | 2.55E-07 | 8.52281 |
| CCDC102A | 0.79708 | 2.2192836 | 9.29319 | 6.88E-17 | 6.11E-15 | 27.8139 |
| SERPINB1 | 0.79724 | 3.6523607 | 6.2684 | 2.88E-09 | 3.64E-08 | 10.6532 |
| ELK3 | 0.79739 | 3.4271543 | 7.36697 | 7.10E-12 | 1.83E-10 | 16.5038 |
| CD2 | 0.79766 | 1.163329 | 6.1893 | 4.34E-09 | 5.23E-08 | 10.2537 |
| ADAM9 | 0.79792 | 5.5513522 | 7.33005 | 8.76E-12 | 2.19E-10 | 16.2989 |
| EFNA4 | 0.79809 | 1.690606 | 10.1499 | 3.06E-19 | 4.59E-17 | 33.1328 |
| BIN2 | 0.7987 | 3.1165317 | 6.73008 | 2.46E-10 | 4.07E-09 | 13.0463 |
| IGLV1-51 | 0.79908 | 1.122662 | 4.46237 | 1.47E-05 | 7.27E-05 | 2.42478 |
| PLK3 | 0.79915 | 3.351905 | 7.30207 | 1.03E-11 | 2.53E-10 | 16.1439 |
| LRRC17 | 0.79937 | 3.2360804 | 5.04902 | 1.13E-06 | 7.37E-06 | 4.87744 |
| SPATA6 | 0.79953 | 3.2073628 | 5.5279 | 1.19E-07 | 9.85E-07 | 7.04358 |
| SEC24D | 0.79965 | 3.0096919 | 7.77031 | 6.95E-13 | 2.34E-11 | 18.7764 |
| REEP4 | 0.8 | 3.3664402 | 8.85395 | 1.05E-15 | 7.25E-14 | 25.1438 |
| CLIC2 | 0.80032 | 2.2003066 | 8.60589 | 4.77E-15 | 2.80E-13 | 23.6566 |
| GSAP | 0.80079 | 2.7945957 | 6.35559 | 1.82E-09 | 2.43E-08 | 11.0972 |
| INPP5D | 0.80081 | 4.5391464 | 5.30955 | 3.38E-07 | 2.51E-06 | 6.03851 |
| FAM46B | 0.80133 | 0.9970843 | 6.12962 | 5.91E-09 | 6.87E-08 | 9.95437 |
| TSPO | 0.80155 | 5.5703999 | 6.28861 | 2.59E-09 | 3.31E-08 | 10.7558 |
| MYD88 | 0.80158 | 3.5975738 | 6.31383 | 2.27E-09 | 2.95E-08 | 10.8841 |
| KIAA0226L | 0.80277 | 2.35367 | 6.43326 | 1.21E-09 | 1.67E-08 | 11.496 |
| LCP1 | 0.80294 | 4.0064124 | 6.23503 | 3.42E-09 | 4.25E-08 | 10.4843 |
| KIF20A | 0.80301 | 1.5355651 | 5.42566 | 1.95E-07 | 1.53E-06 | 6.56945 |
| MIIP | 0.80367 | 4.9829304 | 7.98297 | 2.00E-13 | 7.76E-12 | 19.9977 |
| B3GNT5 | 0.80367 | 2.483664 | 6.13524 | 5.74E-09 | 6.70E-08 | 9.98248 |
| RP11-108M9.4 | 0.8044 | 3.3958617 | 5.41422 | 2.06E-07 | 1.61E-06 | 6.51676 |
| GPRC5A | 0.8046 | 1.1413247 | 4.64101 | 6.88E-06 | 3.70E-05 | 3.14699 |
| RP11-783K16.5 | 0.80474 | 1.3613409 | 5.80079 | 3.13E-08 | 3.01E-07 | 8.33884 |
| C21orf88 | 0.80483 | 1.7210108 | 8.39565 | 1.70E-14 | 8.68E-13 | 22.4089 |
| LYN | 0.80536 | 3.8319919 | 6.72321 | 2.55E-10 | 4.20E-09 | 13.0099 |
| DDOST | 0.80573 | 6.3157983 | 12.5841 | 4.02E-26 | 3.16E-23 | 48.7051 |
| EGR2 | 0.80573 | 3.3782526 | 3.4798 | 0.000637 | 0.0020415 | -1.1297 |
| TWSG1 | 0.80587 | 4.1816066 | 8.96159 | 5.39E-16 | 4.02E-14 | 25.794 |
| ISG20 | 0.80599 | 2.3748493 | 5.36194 | 2.64E-07 | 2.01E-06 | 6.27707 |
| CYP21A1P | 0.80753 | 1.4145588 | 6.89501 | 9.98E-11 | 1.85E-09 | 13.9251 |
| LRRC25 | 0.80763 | 2.2237495 | 6.60826 | 4.75E-10 | 7.25E-09 | 12.405 |
| WNT5A | 0.80812 | 2.9419713 | 5.84452 | 2.51E-08 | 2.49E-07 | 8.55032 |
| OTOS | 0.80839 | 0.9639052 | 5.25741 | 4.32E-07 | 3.12E-06 | 5.80276 |
| GIMAP2 | 0.80871 | 3.0758518 | 7.86533 | 3.99E-13 | 1.44E-11 | 19.3202 |
| LGALS9 | 0.80936 | 5.6121397 | 5.3512 | 2.78E-07 | 2.11E-06 | 6.22803 |
| HP | 0.80954 | 1.9262007 | 3.23878 | 0.001442 | 0.0042062 | -1.8864 |
| TNFRSF1A | 0.80986 | 5.4518205 | 5.65781 | 6.34E-08 | 5.69E-07 | 7.65492 |
| HSPB11 | 0.81081 | 4.3383838 | 13.3931 | 1.97E-28 | 3.99E-25 | 53.9313 |
| PDE8A | 0.81132 | 4.4225375 | 6.67451 | 3.32E-10 | 5.28E-09 | 12.7529 |
| RNF122 | 0.81138 | 2.9464334 | 6.24668 | 3.22E-09 | 4.03E-08 | 10.5432 |
| CHEK2 | 0.81161 | 2.080122 | 8.04887 | 1.35E-13 | 5.54E-12 | 20.3792 |
| LOXL3 | 0.81166 | 3.5062216 | 7.41836 | 5.30E-12 | 1.41E-10 | 16.79 |
| PLEKHG2 | 0.81172 | 3.8564742 | 6.20318 | 4.04E-09 | 4.92E-08 | 10.3235 |
| MTMR11 | 0.81207 | 3.2552469 | 7.23762 | 1.48E-11 | 3.47E-10 | 15.7881 |
| FUOM | 0.8123 | 3.0048296 | 7.61994 | 1.66E-12 | 5.12E-11 | 17.9222 |
| TMEM119 | 0.81332 | 3.772623 | 4.64031 | 6.90E-06 | 3.71E-05 | 3.14413 |
| SH3GLB1 | 0.81344 | 5.276078 | 10.1505 | 3.05E-19 | 4.59E-17 | 33.1367 |
| CARD9 | 0.81447 | 2.3197115 | 8.57122 | 5.88E-15 | 3.36E-13 | 23.4499 |
| RGS10 | 0.81514 | 5.3603895 | 6.22576 | 3.59E-09 | 4.44E-08 | 10.4374 |
| RP4-794H19.4 | 0.81598 | 0.6626759 | 5.62362 | 7.49E-08 | 6.58E-07 | 7.49306 |
| GLIPR1 | 0.81598 | 3.6683843 | 5.27025 | 4.07E-07 | 2.96E-06 | 5.86067 |
| BUB1 | 0.81668 | 1.8817455 | 5.70291 | 5.08E-08 | 4.68E-07 | 7.86941 |
| DDX60L | 0.81687 | 2.6986914 | 8.13975 | 7.88E-14 | 3.42E-12 | 20.9075 |
| SLAMF8 | 0.81698 | 1.7197939 | 5.70542 | 5.01E-08 | 4.62E-07 | 7.88137 |
| RP11-565P22.6 | 0.81713 | 2.0110268 | 5.51042 | 1.30E-07 | 1.07E-06 | 6.96209 |
| SRGN | 0.81732 | 5.8640002 | 5.93025 | 1.63E-08 | 1.69E-07 | 8.96795 |
| IRF7 | 0.81824 | 3.6037527 | 6.05548 | 8.64E-09 | 9.65E-08 | 9.58507 |
| GYPC | 0.81884 | 4.7347006 | 6.03131 | 9.78E-09 | 1.07E-07 | 9.4653 |
| PLEKHS1 | 0.81897 | 0.6090025 | 6.06837 | 8.09E-09 | 9.08E-08 | 9.64908 |
| SAT1 | 0.819 | 7.8811949 | 7.56239 | 2.32E-12 | 6.89E-11 | 17.5974 |
| GAL3ST4 | 0.81956 | 4.2287414 | 7.05227 | 4.18E-11 | 8.69E-10 | 14.7741 |
| YBX1 | 0.8199 | 8.4887659 | 8.39583 | 1.70E-14 | 8.68E-13 | 22.41 |
| TMED5 | 0.8203 | 5.0990162 | 9.32484 | 5.65E-17 | 5.13E-15 | 28.0079 |
| NEK2 | 0.82039 | 1.5452771 | 5.90337 | 1.87E-08 | 1.91E-07 | 8.83655 |
| AHR | 0.82089 | 2.9563866 | 6.49165 | 8.86E-10 | 1.26E-08 | 11.7977 |
| FOXD1 | 0.82107 | 0.9858341 | 6.89811 | 9.81E-11 | 1.82E-09 | 13.9417 |
| SPRY1 | 0.82121 | 3.4274935 | 4.73559 | 4.57E-06 | 2.56E-05 | 3.53825 |
| LRRK2 | 0.82139 | 2.6176085 | 5.89373 | 1.96E-08 | 2.00E-07 | 8.78953 |
| NADK | 0.82161 | 5.0810681 | 11.9813 | 2.10E-24 | 1.11E-21 | 44.8153 |
| SYDE1 | 0.82172 | 3.3290796 | 7.74131 | 8.23E-13 | 2.70E-11 | 18.611 |
| HLA-H | 0.82187 | 2.4193034 | 4.02879 | 8.42E-05 | 0.0003425 | 0.76653 |
| FAM81B | 0.82211 | 0.9649815 | 5.2465 | 4.54E-07 | 3.27E-06 | 5.75366 |
| TFEC | 0.8223 | 1.827749 | 7.54782 | 2.52E-12 | 7.40E-11 | 17.5154 |
| GPR183 | 0.82338 | 2.3861666 | 5.66752 | 6.04E-08 | 5.45E-07 | 7.70102 |
| ITPRIPL1 | 0.82365 | 1.6839424 | 6.59874 | 5.00E-10 | 7.59E-09 | 12.3552 |
| HMGN2P5 | 0.82381 | 4.2529454 | 7.14851 | 2.44E-11 | 5.41E-10 | 15.2989 |
| TEX26 | 0.82421 | 1.0769229 | 5.58419 | 9.08E-08 | 7.79E-07 | 7.30728 |
| NDNF | 0.82444 | 1.6123582 | 5.04809 | 1.13E-06 | 7.39E-06 | 4.87333 |
| BCL3 | 0.82455 | 2.6986298 | 5.73563 | 4.32E-08 | 4.04E-07 | 8.02575 |
| TAGLN | 0.82503 | 5.900361 | 4.15958 | 5.04E-05 | 0.0002165 | 1.25228 |
| IGFLR1 | 0.82697 | 3.3915058 | 8.07053 | 1.19E-13 | 4.96E-12 | 20.5048 |
| ANTXR2 | 0.82719 | 3.3738911 | 6.724 | 2.54E-10 | 4.19E-09 | 13.0141 |
| HOXD-AS2 | 0.82734 | 1.0446885 | 5.60559 | 8.18E-08 | 7.12E-07 | 7.40802 |
| SNORD3B-1 | 0.82743 | 1.2219531 | 5.11189 | 8.48E-07 | 5.67E-06 | 5.15373 |
| AR | 0.82815 | 1.7711134 | 7.05198 | 4.18E-11 | 8.70E-10 | 14.7725 |
| CMTM7 | 0.82854 | 2.7923869 | 7.26098 | 1.30E-11 | 3.09E-10 | 15.9168 |
| A2M | 0.82875 | 7.607925 | 6.75886 | 2.10E-10 | 3.55E-09 | 13.1987 |
| ITGAM | 0.82884 | 2.9177536 | 6.63862 | 4.03E-10 | 6.25E-09 | 12.5641 |
| RAD51AP1 | 0.82941 | 2.4023463 | 6.58171 | 5.48E-10 | 8.22E-09 | 12.2662 |
| CASP6 | 0.83037 | 2.9111975 | 9.06841 | 2.78E-16 | 2.18E-14 | 26.442 |
| FAM83D | 0.83093 | 1.4685393 | 6.09258 | 7.14E-09 | 8.14E-08 | 9.76951 |
| FHOD1 | 0.83115 | 3.5045684 | 8.02403 | 1.57E-13 | 6.27E-12 | 20.2352 |
| PRR24 | 0.83124 | 4.1444018 | 6.73559 | 2.39E-10 | 3.97E-09 | 13.0754 |
| CISH | 0.83132 | 1.3057105 | 7.38176 | 6.53E-12 | 1.69E-10 | 16.5861 |
| PLA2G4A | 0.83149 | 2.1905033 | 7.93567 | 2.64E-13 | 9.87E-12 | 19.7247 |
| HMGN2 | 0.83152 | 8.1816064 | 11.5187 | 4.35E-23 | 1.51E-20 | 41.8389 |
| STAT5A | 0.83169 | 2.9836887 | 7.96716 | 2.19E-13 | 8.44E-12 | 19.9064 |
| FKBP9 | 0.83181 | 4.6340002 | 5.60178 | 8.33E-08 | 7.24E-07 | 7.39005 |
| LYZ | 0.83205 | 3.4801571 | 3.33047 | 0.001062 | 0.0032054 | -1.6041 |
| SLC4A2 | 0.83261 | 5.5704817 | 9.29953 | 6.61E-17 | 5.89E-15 | 27.8528 |
| RP11-155G14.5 | 0.83265 | 1.5180696 | 5.19869 | 5.68E-07 | 3.98E-06 | 5.53926 |
| ARL4C | 0.83289 | 4.1355674 | 4.87432 | 2.48E-06 | 1.49E-05 | 4.12302 |
| PRR11 | 0.83342 | 2.6894457 | 6.79877 | 1.69E-10 | 2.94E-09 | 13.4108 |
| GLRX | 0.83354 | 5.0556386 | 7.65603 | 1.35E-12 | 4.22E-11 | 18.1265 |
| CCDC18 | 0.83358 | 1.6438897 | 10.7227 | 7.69E-21 | 1.68E-18 | 36.7523 |
| SLPI | 0.83458 | 2.757856 | 3.93682 | 0.00012 | 0.0004687 | 0.43265 |
| FUCA2 | 0.83472 | 3.9011616 | 6.2469 | 3.22E-09 | 4.03E-08 | 10.5443 |
| GPR34 | 0.8349 | 4.8624562 | 5.23018 | 4.90E-07 | 3.50E-06 | 5.6803 |
| GSDMD | 0.83518 | 3.6622997 | 7.02427 | 4.88E-11 | 9.95E-10 | 14.6222 |
| RP11-79D8.2 | 0.83552 | 2.9110077 | 3.9304 | 0.000123 | 0.0004786 | 0.40957 |
| CLEC18A | 0.83583 | 1.704336 | 7.13926 | 2.57E-11 | 5.66E-10 | 15.2482 |
| SLC16A1 | 0.83589 | 5.2424061 | 8.79937 | 1.46E-15 | 9.72E-14 | 24.8153 |
| F2R | 0.8359 | 4.5553308 | 4.99816 | 1.42E-06 | 9.07E-06 | 4.65574 |
| SERINC2 | 0.836 | 1.666317 | 5.6828 | 5.61E-08 | 5.11E-07 | 7.77361 |
| CD33 | 0.83615 | 2.7826585 | 7.04929 | 4.25E-11 | 8.82E-10 | 14.7579 |
| MMP19 | 0.83623 | 1.467977 | 5.0004 | 1.41E-06 | 8.99E-06 | 4.66548 |
| SPC24 | 0.83642 | 1.9811255 | 5.26494 | 4.17E-07 | 3.03E-06 | 5.83669 |
| PDLIM4 | 0.837 | 3.1511363 | 3.79019 | 0.000208 | 0.0007634 | -0.0864 |
| SLC15A3 | 0.83735 | 4.6005912 | 6.10963 | 6.55E-09 | 7.52E-08 | 9.85451 |
| ITGAL | 0.83775 | 2.3465826 | 6.55086 | 6.46E-10 | 9.53E-09 | 12.1052 |
| CDK6 | 0.83776 | 2.9505743 | 5.55074 | 1.07E-07 | 8.96E-07 | 7.15037 |
| C21orf62 | 0.8387 | 3.2288996 | 3.71276 | 0.000277 | 0.0009815 | -0.3538 |
| PPCS | 0.83903 | 4.4249908 | 7.27498 | 1.20E-11 | 2.87E-10 | 15.9941 |
| VAV1 | 0.8395 | 2.6143124 | 7.38464 | 6.42E-12 | 1.67E-10 | 16.6022 |
| MYO1F | 0.83956 | 3.7730268 | 6.70123 | 2.87E-10 | 4.67E-09 | 12.8938 |
| SAA2 | 0.83969 | 0.7115524 | 3.75252 | 0.00024 | 0.0008639 | -0.217 |
| BARD1 | 0.84069 | 2.4011086 | 7.3901 | 6.23E-12 | 1.63E-10 | 16.6325 |
| AP001007.1 | 0.84095 | 2.1273394 | 6.77037 | 1.97E-10 | 3.37E-09 | 13.2598 |
| CCDC40 | 0.84099 | 2.781418 | 7.28478 | 1.13E-11 | 2.75E-10 | 16.0483 |
| RBP1 | 0.84103 | 4.454514 | 3.47457 | 0.000648 | 0.0020724 | -1.1466 |
| HSPA1B | 0.84159 | 6.3887306 | 4.55476 | 9.94E-06 | 5.14E-05 | 2.79552 |
| VASP | 0.84173 | 4.2386278 | 8.19446 | 5.69E-14 | 2.54E-12 | 21.2269 |
| OLR1 | 0.84214 | 4.2049215 | 4.11686 | 5.97E-05 | 0.0002514 | 1.09222 |
| ZMYM6NB | 0.84232 | 3.741085 | 9.26993 | 7.96E-17 | 6.96E-15 | 27.6714 |
| RP11-344B5.2 | 0.84235 | 1.8734382 | 6.7121 | 2.71E-10 | 4.42E-09 | 12.9511 |
| CAPZA1 | 0.84268 | 5.1141914 | 11.8974 | 3.65E-24 | 1.71E-21 | 44.2744 |
| DPEP1 | 0.84346 | 1.3550573 | 4.72028 | 4.88E-06 | 2.71E-05 | 3.47451 |
| BTBD19 | 0.84361 | 2.0788179 | 8.45889 | 1.16E-14 | 6.20E-13 | 22.7829 |
| RP11-626G11.3 | 0.8437 | 2.1445855 | 6.99824 | 5.64E-11 | 1.13E-09 | 14.4812 |
| RNASE6 | 0.84452 | 3.8760736 | 7.02367 | 4.90E-11 | 9.98E-10 | 14.6189 |
| SPAG1 | 0.8455 | 2.3615537 | 7.4851 | 3.62E-12 | 1.02E-10 | 17.1632 |
| ZFP36 | 0.8467 | 5.9231576 | 4.21285 | 4.07E-05 | 0.0001794 | 1.45376 |
| SHCBP1 | 0.84773 | 1.8453819 | 7.43701 | 4.76E-12 | 1.29E-10 | 16.8941 |
| CDKN3 | 0.8482 | 2.6737781 | 6.50129 | 8.42E-10 | 1.21E-08 | 11.8476 |
| RNF149 | 0.84835 | 3.8264442 | 9.62904 | 8.36E-18 | 9.24E-16 | 29.8833 |
| CASP8 | 0.84914 | 2.3998182 | 8.08831 | 1.07E-13 | 4.55E-12 | 20.6081 |
| CCDC135 | 0.84935 | 1.2539994 | 7.24807 | 1.39E-11 | 3.29E-10 | 15.8457 |
| LY86 | 0.85007 | 4.077839 | 5.50494 | 1.33E-07 | 1.09E-06 | 6.9366 |
| KIF23 | 0.85126 | 1.8144433 | 5.89836 | 1.92E-08 | 1.96E-07 | 8.81213 |
| ASPM | 0.85156 | 1.088986 | 6.97303 | 6.49E-11 | 1.27E-09 | 14.345 |
| OSM | 0.85212 | 1.5096431 | 4.58076 | 8.90E-06 | 4.66E-05 | 2.90094 |
| SLC30A7 | 0.85253 | 2.1670072 | 12.6594 | 2.45E-26 | 2.06E-23 | 49.1917 |
| HOXD10 | 0.85268 | 0.777407 | 4.57934 | 8.95E-06 | 4.68E-05 | 2.89516 |
| CROT | 0.85302 | 3.776482 | 9.81346 | 2.60E-18 | 3.20E-16 | 31.0287 |
| NPC2 | 0.85312 | 7.0476637 | 6.67149 | 3.38E-10 | 5.36E-09 | 12.737 |
| IGHM | 0.85358 | 1.215157 | 4.07235 | 7.11E-05 | 0.0002936 | 0.92692 |
| CDCA7 | 0.85363 | 2.7132084 | 5.46269 | 1.63E-07 | 1.31E-06 | 6.74045 |
| CCNA2 | 0.85436 | 2.0691018 | 6.29811 | 2.46E-09 | 3.17E-08 | 10.8041 |
| LTBR | 0.85437 | 3.4799217 | 7.15936 | 2.30E-11 | 5.14E-10 | 15.3582 |
| ANO6 | 0.85442 | 4.0677123 | 6.76538 | 2.03E-10 | 3.44E-09 | 13.2333 |
| IRF1 | 0.8551 | 3.8055332 | 5.6209 | 7.59E-08 | 6.66E-07 | 7.48024 |
| IL4I1 | 0.8552 | 1.4019765 | 6.65379 | 3.72E-10 | 5.82E-09 | 12.6439 |
| PSMB9 | 0.85534 | 5.1252607 | 6.30331 | 2.40E-09 | 3.10E-08 | 10.8305 |
| BCAT1 | 0.85564 | 2.7212858 | 5.21229 | 5.33E-07 | 3.76E-06 | 5.60012 |
| JUNB | 0.8561 | 6.1090007 | 4.30798 | 2.77E-05 | 0.0001275 | 1.81868 |
| NFKBIZ | 0.85632 | 2.3158469 | 6.50472 | 8.27E-10 | 1.18E-08 | 11.8654 |
| APBB1IP | 0.85647 | 3.9188833 | 5.7801 | 3.46E-08 | 3.31E-07 | 8.23919 |
| TXNDC12 | 0.85668 | 5.0980817 | 15.8219 | 2.61E-35 | 3.17E-31 | 69.476 |
| NCF4 | 0.85692 | 2.9611685 | 7.59577 | 1.91E-12 | 5.80E-11 | 17.7857 |
| CAPS | 0.85797 | 4.3742478 | 6.31965 | 2.20E-09 | 2.87E-08 | 10.9138 |
| TAPSAR1 | 0.85847 | 3.6550554 | 6.85817 | 1.22E-10 | 2.22E-09 | 13.7278 |
| CACNA2D4 | 0.85876 | 1.8985139 | 7.50622 | 3.21E-12 | 9.12E-11 | 17.2816 |
| FOSL1 | 0.85911 | 1.4460561 | 6.31998 | 2.20E-09 | 2.87E-08 | 10.9154 |
| TNFRSF19 | 0.85956 | 3.4492079 | 5.53121 | 1.17E-07 | 9.71E-07 | 7.05905 |
| LIF | 0.86003 | 1.3616605 | 4.82997 | 3.02E-06 | 1.77E-05 | 3.93468 |
| TTC26 | 0.86087 | 2.0461292 | 9.99479 | 8.23E-19 | 1.14E-16 | 32.1605 |
| FAM46A | 0.86143 | 2.1725796 | 7.09865 | 3.23E-11 | 6.92E-10 | 15.0265 |
| GPSM3 | 0.86144 | 3.8572841 | 7.16537 | 2.22E-11 | 5.00E-10 | 15.3911 |
| SERTAD3 | 0.86162 | 3.2458117 | 9.24929 | 9.05E-17 | 7.74E-15 | 27.5451 |
| CYTH4 | 0.86171 | 3.2236492 | 6.55318 | 6.38E-10 | 9.44E-09 | 12.1173 |
| FABP5P7 | 0.86186 | 1.0913766 | 4.44396 | 1.58E-05 | 7.78E-05 | 2.35161 |
| ARHGAP30 | 0.86246 | 2.9409358 | 7.83924 | 4.65E-13 | 1.66E-11 | 19.1706 |
| THBS1 | 0.86255 | 2.4949985 | 4.24962 | 3.51E-05 | 0.0001573 | 1.59402 |
| MYO5B | 0.86266 | 1.5244873 | 6.37424 | 1.65E-09 | 2.22E-08 | 11.1927 |
| UGCG | 0.86284 | 3.5567698 | 9.61737 | 9.00E-18 | 9.82E-16 | 29.811 |
| FOXM1 | 0.86311 | 3.0600544 | 4.7349 | 4.58E-06 | 2.56E-05 | 3.53539 |
| HCK | 0.86316 | 3.5367896 | 6.64577 | 3.88E-10 | 6.04E-09 | 12.6017 |
| AC096579.7 | 0.86398 | 1.5098346 | 4.08877 | 6.66E-05 | 0.0002776 | 0.98773 |
| NUSAP1 | 0.86403 | 3.5138287 | 4.20875 | 4.14E-05 | 0.000182 | 1.43816 |
| ARHGAP4 | 0.86473 | 4.1484102 | 8.71068 | 2.52E-15 | 1.57E-13 | 24.2829 |
| EVA1C | 0.86491 | 3.4606701 | 6.02328 | 1.02E-08 | 1.11E-07 | 9.42558 |
| PDIA4 | 0.86495 | 5.4903583 | 7.56112 | 2.34E-12 | 6.93E-11 | 17.5903 |
| SERTAD1 | 0.865 | 2.7186165 | 8.36471 | 2.05E-14 | 1.02E-12 | 22.2264 |
| BTK | 0.86588 | 2.8732264 | 6.81458 | 1.55E-10 | 2.74E-09 | 13.495 |
| OAS1 | 0.86619 | 3.8641715 | 5.56065 | 1.02E-07 | 8.61E-07 | 7.19679 |
| MARVELD1 | 0.8663 | 2.5401486 | 6.67101 | 3.39E-10 | 5.37E-09 | 12.7344 |
| STXBP2 | 0.86651 | 3.632534 | 8.07674 | 1.15E-13 | 4.84E-12 | 20.5409 |
| OSTC | 0.86652 | 5.1836048 | 9.7964 | 2.90E-18 | 3.51E-16 | 30.9224 |
| EPSTI1 | 0.8667 | 2.4837931 | 6.19127 | 4.29E-09 | 5.18E-08 | 10.2636 |
| CHST2 | 0.86686 | 3.8781469 | 7.57471 | 2.16E-12 | 6.46E-11 | 17.6669 |
| TNFAIP8L2 | 0.86736 | 2.8585945 | 7.75721 | 7.50E-13 | 2.49E-11 | 18.7017 |
| AC093673.5 | 0.86768 | 3.1634354 | 7.82168 | 5.15E-13 | 1.81E-11 | 19.07 |
| FOSL2 | 0.86896 | 3.8232556 | 5.77962 | 3.47E-08 | 3.31E-07 | 8.23689 |
| UNC93B1 | 0.86906 | 3.8054323 | 6.81393 | 1.56E-10 | 2.74E-09 | 13.4915 |
| MICALL2 | 0.86987 | 4.3374077 | 6.78533 | 1.82E-10 | 3.13E-09 | 13.3393 |
| KIAA0101 | 0.87012 | 2.5235794 | 5.03878 | 1.18E-06 | 7.68E-06 | 4.83267 |
| TNXB | 0.87065 | 3.2277694 | 7.37879 | 6.64E-12 | 1.72E-10 | 16.5696 |
| FGL2 | 0.871 | 3.0667895 | 6.31989 | 2.20E-09 | 2.87E-08 | 10.915 |
| LIPG | 0.87105 | 2.1541263 | 6.40273 | 1.42E-09 | 1.92E-08 | 11.3389 |
| MYL12A | 0.87153 | 5.944865 | 6.99463 | 5.75E-11 | 1.14E-09 | 14.4617 |
| KIF4A | 0.87175 | 1.5856378 | 6.33044 | 2.08E-09 | 2.73E-08 | 10.9688 |
| TMEM71 | 0.87214 | 1.362547 | 6.9212 | 8.64E-11 | 1.63E-09 | 14.0658 |
| CD37 | 0.87218 | 4.4446302 | 5.62062 | 7.60E-08 | 6.67E-07 | 7.47893 |
| RSPH4A | 0.87345 | 1.1797433 | 7.09347 | 3.32E-11 | 7.11E-10 | 14.9983 |
| EVI2B | 0.87355 | 3.7268521 | 6.54352 | 6.72E-10 | 9.88E-09 | 12.067 |
| FOS | 0.87384 | 6.9777208 | 3.73214 | 0.000258 | 0.0009231 | -0.2873 |
| ABI3 | 0.87451 | 3.1609767 | 6.83299 | 1.40E-10 | 2.50E-09 | 13.5932 |
| RP11-61L23.2 | 0.87466 | 1.6651177 | 5.2501 | 4.47E-07 | 3.22E-06 | 5.76987 |
| CPVL | 0.87491 | 4.304619 | 6.07942 | 7.64E-09 | 8.64E-08 | 9.704 |
| RNASET2 | 0.87606 | 6.3428625 | 7.04056 | 4.46E-11 | 9.21E-10 | 14.7105 |
| C1orf158 | 0.87683 | 0.8460505 | 5.49013 | 1.43E-07 | 1.16E-06 | 6.86769 |
| 15-Sep | 0.87743 | 7.0229607 | 11.6786 | 1.53E-23 | 5.99E-21 | 42.8665 |
| AK2 | 0.87806 | 5.9917103 | 12.8814 | 5.69E-27 | 6.16E-24 | 50.6263 |
| LAMB2 | 0.8781 | 6.5954519 | 8.53311 | 7.41E-15 | 4.12E-13 | 23.2233 |
| TAF13 | 0.87894 | 5.4571917 | 9.86578 | 1.87E-18 | 2.33E-16 | 31.3547 |
| DHRS3 | 0.87895 | 5.0350128 | 6.65972 | 3.60E-10 | 5.67E-09 | 12.675 |
| TC2N | 0.87902 | 1.3890118 | 7.25752 | 1.32E-11 | 3.15E-10 | 15.8977 |
| NCF1 | 0.87913 | 2.4183393 | 6.82327 | 1.48E-10 | 2.62E-09 | 13.5413 |
| CD86 | 0.87963 | 3.0262245 | 6.67658 | 3.28E-10 | 5.23E-09 | 12.7638 |
| LEPRE1 | 0.87984 | 3.6966976 | 10.1873 | 2.41E-19 | 3.68E-17 | 33.3679 |
| HIST1H2BD | 0.88004 | 3.19886 | 6.74394 | 2.28E-10 | 3.81E-09 | 13.1196 |
| LHFPL2 | 0.88117 | 4.1195952 | 7.4791 | 3.74E-12 | 1.04E-10 | 17.1296 |
| FBLN5 | 0.88126 | 3.4588548 | 4.75541 | 4.19E-06 | 2.37E-05 | 3.62099 |
| LTBP1 | 0.88162 | 3.6505662 | 6.25812 | 3.03E-09 | 3.82E-08 | 10.6011 |
| GAS2L3 | 0.88163 | 1.6126596 | 6.81379 | 1.56E-10 | 2.74E-09 | 13.4908 |
| FAM20A | 0.88229 | 1.8030002 | 5.36289 | 2.63E-07 | 2.00E-06 | 6.2814 |
| CENPK | 0.88271 | 1.9922223 | 6.36253 | 1.76E-09 | 2.34E-08 | 11.1327 |
| CALU | 0.88285 | 5.676656 | 6.68917 | 3.07E-10 | 4.93E-09 | 12.8301 |
| RCN3 | 0.88311 | 3.183625 | 7.77283 | 6.85E-13 | 2.31E-11 | 18.7907 |
| PDLIM7 | 0.88352 | 4.8122116 | 7.31269 | 9.67E-12 | 2.39E-10 | 16.2027 |
| NCAPG | 0.88443 | 1.6805129 | 6.25314 | 3.11E-09 | 3.91E-08 | 10.5759 |
| CD4 | 0.88561 | 4.5088799 | 6.73652 | 2.37E-10 | 3.95E-09 | 13.0804 |
| RBM47 | 0.88618 | 2.168815 | 7.57349 | 2.18E-12 | 6.49E-11 | 17.66 |
| FGFBP2 | 0.88636 | 1.4688101 | 5.53298 | 1.16E-07 | 9.64E-07 | 7.06732 |
| ST14 | 0.88666 | 1.7844644 | 6.07364 | 7.87E-09 | 8.87E-08 | 9.67527 |
| MLF1IP | 0.88676 | 2.5216362 | 5.55459 | 1.05E-07 | 8.83E-07 | 7.16839 |
| EVA1B | 0.8886 | 3.6027606 | 7.63747 | 1.50E-12 | 4.67E-11 | 18.0214 |
| LST1 | 0.88948 | 4.8379222 | 6.9135 | 9.01E-11 | 1.69E-09 | 14.0244 |
| APOL1 | 0.89059 | 2.7961077 | 6.19419 | 4.23E-09 | 5.11E-08 | 10.2783 |
| ACTG2 | 0.89067 | 1.8553335 | 4.05271 | 7.67E-05 | 0.0003145 | 0.85444 |
| REST | 0.8907 | 2.5262907 | 10.5437 | 2.44E-20 | 4.68E-18 | 35.6166 |
| FAM229A | 0.89103 | 2.4594534 | 9.62564 | 8.54E-18 | 9.40E-16 | 29.8623 |
| CALD1 | 0.89229 | 5.7914074 | 7.10778 | 3.06E-11 | 6.59E-10 | 15.0763 |
| FAM114A1 | 0.8926 | 3.1710462 | 6.7103 | 2.74E-10 | 4.46E-09 | 12.9417 |
| GRB14 | 0.89266 | 1.4599498 | 5.21661 | 5.22E-07 | 3.70E-06 | 5.61944 |
| TNFAIP8 | 0.89391 | 1.8221329 | 7.40916 | 5.59E-12 | 1.48E-10 | 16.7387 |
| IFITM3 | 0.89434 | 7.4412628 | 5.23142 | 4.88E-07 | 3.48E-06 | 5.68589 |
| C10orf10 | 0.89461 | 4.4566003 | 5.5722 | 9.62E-08 | 8.19E-07 | 7.25099 |
| IL1B | 0.89515 | 3.0021622 | 3.54666 | 0.000504 | 0.0016604 | -0.9114 |
| SYNC | 0.89545 | 2.886758 | 6.6629 | 3.54E-10 | 5.59E-09 | 12.6918 |
| MYOF | 0.89692 | 2.9085085 | 6.16397 | 4.95E-09 | 5.85E-08 | 10.1264 |
| HPGDS | 0.89755 | 2.7628895 | 6.97306 | 6.48E-11 | 1.27E-09 | 14.3451 |
| PIM1 | 0.89806 | 3.6062914 | 7.62541 | 1.61E-12 | 4.98E-11 | 17.9532 |
| MILR1 | 0.89937 | 2.3253788 | 6.90727 | 9.33E-11 | 1.75E-09 | 13.9909 |
| CAPN5 | 0.89954 | 4.4300682 | 6.4283 | 1.24E-09 | 1.70E-08 | 11.4704 |
| HLA-F | 0.89954 | 5.098506 | 6.52345 | 7.48E-10 | 1.09E-08 | 11.9627 |
| PI16 | 0.89978 | 3.5371153 | 3.58662 | 0.000437 | 0.0014687 | -0.7792 |
| HOXB2 | 0.90078 | 1.1673743 | 5.39992 | 2.20E-07 | 1.71E-06 | 6.45102 |
| HOXD9 | 0.9015 | 0.8210037 | 5.78066 | 3.46E-08 | 3.30E-07 | 8.24186 |
| FYB | 0.90177 | 3.3845764 | 6.18629 | 4.41E-09 | 5.29E-08 | 10.2385 |
| HIST1H1C | 0.90186 | 3.8281424 | 6.436 | 1.19E-09 | 1.65E-08 | 11.5101 |
| NAMPTL | 0.90218 | 1.7633991 | 5.8954 | 1.95E-08 | 1.99E-07 | 8.79768 |
| PLCE1 | 0.90256 | 2.6879934 | 8.97649 | 4.92E-16 | 3.69E-14 | 25.8842 |
| RCC2 | 0.90267 | 4.9894231 | 9.99224 | 8.36E-19 | 1.16E-16 | 32.1446 |
| CCR5 | 0.90295 | 1.3396546 | 7.19203 | 1.91E-11 | 4.37E-10 | 15.5374 |
| RNU1-1 | 0.90316 | 3.9796016 | 2.49925 | 0.013389 | 0.0293637 | -3.9022 |
| RNU1-2 | 0.90316 | 3.9796016 | 2.49925 | 0.013389 | 0.0293637 | -3.9022 |
| RNU1-27P | 0.90316 | 3.9796016 | 2.49925 | 0.013389 | 0.0293637 | -3.9022 |
| RNU1-28P | 0.90316 | 3.9796016 | 2.49925 | 0.013389 | 0.0293637 | -3.9022 |
| RNU1-3 | 0.90316 | 3.9796016 | 2.49925 | 0.013389 | 0.0293637 | -3.9022 |
| RNU1-4 | 0.90316 | 3.9796016 | 2.49925 | 0.013389 | 0.0293637 | -3.9022 |
| RNVU1-18 | 0.90316 | 3.9796016 | 2.49925 | 0.013389 | 0.0293637 | -3.9022 |
| ITGA5 | 0.90373 | 3.3888583 | 5.08191 | 9.72E-07 | 6.43E-06 | 5.02166 |
| FBLIM1 | 0.90382 | 2.3749468 | 5.44376 | 1.79E-07 | 1.42E-06 | 6.65291 |
| SLC2A5 | 0.90463 | 4.8313036 | 5.1021 | 8.87E-07 | 5.91E-06 | 5.11054 |
| CELSR1 | 0.90487 | 1.1075148 | 7.41079 | 5.53E-12 | 1.47E-10 | 16.7478 |
| TRIP6 | 0.90497 | 5.0334778 | 6.5805 | 5.51E-10 | 8.27E-09 | 12.2598 |
| CD48 | 0.90499 | 1.8995038 | 5.28819 | 3.74E-07 | 2.75E-06 | 5.94173 |
| DOK3 | 0.90501 | 2.9631511 | 7.9363 | 2.63E-13 | 9.85E-12 | 19.7283 |
| LOXL1 | 0.90512 | 2.1008733 | 4.85875 | 2.66E-06 | 1.58E-05 | 4.05676 |
| HOXA4 | 0.90577 | 0.7307787 | 5.11492 | 8.36E-07 | 5.61E-06 | 5.16707 |
| G0S2 | 0.90579 | 1.3804873 | 4.50371 | 1.23E-05 | 6.24E-05 | 2.58992 |
| FILIP1L | 0.90596 | 3.3857499 | 5.82949 | 2.71E-08 | 2.66E-07 | 8.47753 |
| CTD-2521M24.9 | 0.90598 | 1.9998559 | 7.39541 | 6.04E-12 | 1.59E-10 | 16.6621 |
| PCOLCE | 0.9061 | 3.8132691 | 5.60176 | 8.34E-08 | 7.24E-07 | 7.38999 |
| FOLR1 | 0.90611 | 1.6229011 | 6.33076 | 2.07E-09 | 2.73E-08 | 10.9704 |
| SERPING1 | 0.90628 | 5.5891096 | 4.99096 | 1.47E-06 | 9.34E-06 | 4.6245 |
| SNORD3D | 0.90836 | 0.9197594 | 5.67859 | 5.72E-08 | 5.20E-07 | 7.7536 |
| KANSL1L | 0.90861 | 3.1525342 | 8.97974 | 4.82E-16 | 3.63E-14 | 25.9039 |
| SERPINB8 | 0.90887 | 2.7717893 | 8.41298 | 1.53E-14 | 7.92E-13 | 22.5113 |
| ITPKC | 0.90933 | 3.1714914 | 9.73791 | 4.20E-18 | 4.96E-16 | 30.5587 |
| NECAP2 | 0.90978 | 5.4491648 | 10.6611 | 1.14E-20 | 2.32E-18 | 36.3607 |
| CD276 | 0.90998 | 5.2916522 | 6.90922 | 9.23E-11 | 1.73E-09 | 14.0013 |
| KIFC1 | 0.91042 | 2.3904021 | 5.15611 | 6.91E-07 | 4.75E-06 | 5.34953 |
| HOXD3 | 0.9107 | 1.2496434 | 5.40416 | 2.16E-07 | 1.68E-06 | 6.47049 |
| IKBIP | 0.91091 | 2.883769 | 8.80427 | 1.42E-15 | 9.48E-14 | 24.8447 |
| SIGLEC9 | 0.91105 | 2.1762321 | 7.35411 | 7.64E-12 | 1.94E-10 | 16.4324 |
| BTN3A2 | 0.91145 | 4.6825526 | 6.80562 | 1.63E-10 | 2.85E-09 | 13.4473 |
| SMC4 | 0.91156 | 3.6262675 | 6.74375 | 2.28E-10 | 3.81E-09 | 13.1186 |
| NCKAP1L | 0.91212 | 3.4496145 | 6.99776 | 5.66E-11 | 1.13E-09 | 14.4786 |
| FPGT | 0.91271 | 2.8119003 | 11.7426 | 1.01E-23 | 4.07E-21 | 43.2783 |
| TEKT2 | 0.91295 | 1.9764706 | 7.42192 | 5.19E-12 | 1.39E-10 | 16.8099 |
| TNFAIP6 | 0.91336 | 1.521055 | 5.95482 | 1.44E-08 | 1.52E-07 | 9.08836 |
| RPS2P5 | 0.91432 | 5.1368158 | 6.58022 | 5.52E-10 | 8.27E-09 | 12.2584 |
| SLC7A7 | 0.91468 | 3.1843456 | 7.6491 | 1.41E-12 | 4.38E-11 | 18.0872 |
| NABP1 | 0.91555 | 2.8012299 | 7.68408 | 1.15E-12 | 3.64E-11 | 18.2856 |
| SNX10 | 0.91627 | 4.1882493 | 4.81342 | 3.25E-06 | 1.89E-05 | 3.86472 |
| RNF135 | 0.91653 | 2.763269 | 7.78379 | 6.42E-13 | 2.19E-11 | 18.8533 |
| SAMD9 | 0.91664 | 2.3234768 | 8.33793 | 2.41E-14 | 1.17E-12 | 22.0685 |
| IL6 | 0.91688 | 1.3029633 | 5.25628 | 4.34E-07 | 3.14E-06 | 5.79765 |
| TRAM2 | 0.91731 | 2.231639 | 9.51372 | 1.73E-17 | 1.78E-15 | 29.1703 |
| CEP55 | 0.91743 | 1.2838384 | 7.35895 | 7.44E-12 | 1.90E-10 | 16.4593 |
| FAM181A-AS1 | 0.91749 | 1.7363748 | 5.45544 | 1.69E-07 | 1.35E-06 | 6.70691 |
| NEXN | 0.91769 | 1.8975956 | 8.67507 | 3.13E-15 | 1.92E-13 | 24.0697 |
| RP11-472N13.3 | 0.91793 | 2.1107193 | 6.57947 | 5.54E-10 | 8.30E-09 | 12.2544 |
| SCP2 | 0.91796 | 6.7797445 | 13.6492 | 3.66E-29 | 1.08E-25 | 55.5839 |
| CD248 | 0.91899 | 2.2061916 | 4.56465 | 9.53E-06 | 4.95E-05 | 2.83558 |
| APITD1 | 0.9192 | 3.778579 | 11.9197 | 3.15E-24 | 1.56E-21 | 44.4183 |
| GJC1 | 0.91934 | 2.315505 | 6.68257 | 3.18E-10 | 5.09E-09 | 12.7954 |
| B2M | 0.92116 | 11.703913 | 7.27803 | 1.18E-11 | 2.84E-10 | 16.011 |
| CENPA | 0.92133 | 1.5406721 | 6.48315 | 9.27E-10 | 1.32E-08 | 11.7536 |
| BTN2A2 | 0.92177 | 3.1969164 | 8.81856 | 1.30E-15 | 8.77E-14 | 24.9307 |
| ADAM12 | 0.92242 | 1.3914261 | 5.68993 | 5.41E-08 | 4.96E-07 | 7.80755 |
| DENND2D | 0.92256 | 2.0145618 | 6.66172 | 3.56E-10 | 5.62E-09 | 12.6855 |
| APOBEC3C | 0.92377 | 2.807077 | 6.76267 | 2.06E-10 | 3.49E-09 | 13.2189 |
| HAVCR2 | 0.92446 | 3.7187061 | 6.61123 | 4.67E-10 | 7.14E-09 | 12.4205 |
| HK2 | 0.92462 | 3.020044 | 6.45793 | 1.06E-09 | 1.48E-08 | 11.6232 |
| MFNG | 0.9249 | 3.4452237 | 7.98289 | 2.00E-13 | 7.76E-12 | 19.9972 |
| MMEL1 | 0.92738 | 1.297378 | 7.03288 | 4.65E-11 | 9.55E-10 | 14.6689 |
| RARRES2 | 0.92751 | 3.4641295 | 3.62329 | 0.000383 | 0.0013064 | -0.6568 |
| TLR1 | 0.92843 | 2.7278667 | 7.13858 | 2.58E-11 | 5.67E-10 | 15.2445 |
| SNX7 | 0.92911 | 4.2243457 | 10.2533 | 1.58E-19 | 2.59E-17 | 33.7833 |
| IGF2BP2 | 0.92929 | 1.0481864 | 5.75634 | 3.90E-08 | 3.68E-07 | 8.12498 |
| FMOD | 0.92961 | 2.0127289 | 4.72429 | 4.80E-06 | 2.67E-05 | 3.49118 |
| BMP8B | 0.92968 | 1.8309096 | 6.85762 | 1.23E-10 | 2.22E-09 | 13.7248 |
| DCTD | 0.93115 | 4.8227042 | 7.67336 | 1.22E-12 | 3.85E-11 | 18.2248 |
| CITED1 | 0.93119 | 3.6482432 | 5.64625 | 6.71E-08 | 5.98E-07 | 7.60012 |
| MOV10 | 0.93149 | 4.4985133 | 10.2253 | 1.89E-19 | 3.00E-17 | 33.6067 |
| LILRB1 | 0.93169 | 2.5815685 | 7.00293 | 5.50E-11 | 1.10E-09 | 14.5066 |
| AC010441.1 | 0.932 | 3.6151974 | 6.83937 | 1.35E-10 | 2.43E-09 | 13.6272 |
| HS2ST1 | 0.93263 | 4.0454347 | 11.7681 | 8.51E-24 | 3.51E-21 | 43.4423 |
| ATF3 | 0.9331 | 4.0070225 | 4.82621 | 3.07E-06 | 1.80E-05 | 3.91879 |
| FBXO32 | 0.93336 | 4.8292856 | 5.84558 | 2.50E-08 | 2.48E-07 | 8.55544 |
| TMSB10 | 0.93402 | 9.8397711 | 7.79229 | 6.11E-13 | 2.08E-11 | 18.9019 |
| ADPRHL2 | 0.93443 | 4.6401621 | 15.0272 | 4.48E-33 | 3.63E-29 | 64.4292 |
| IGFBP7 | 0.93446 | 8.263386 | 6.69785 | 2.93E-10 | 4.74E-09 | 12.8759 |
| GMFG | 0.93474 | 4.5353393 | 8.41194 | 1.54E-14 | 7.95E-13 | 22.5052 |
| SLC2A10 | 0.93506 | 2.1375775 | 8.21949 | 4.90E-14 | 2.20E-12 | 21.3733 |
| ARHGDIB | 0.93529 | 6.295091 | 7.45585 | 4.28E-12 | 1.17E-10 | 16.9995 |
| RCAN1 | 0.93572 | 6.1099358 | 5.84902 | 2.46E-08 | 2.44E-07 | 8.57216 |
| RAC2 | 0.93652 | 2.7935713 | 6.90448 | 9.47E-11 | 1.77E-09 | 13.9759 |
| CCDC146 | 0.93664 | 2.8211015 | 7.21222 | 1.71E-11 | 3.96E-10 | 15.6483 |
| CHIC2 | 0.93696 | 3.7537106 | 8.17311 | 6.46E-14 | 2.86E-12 | 21.1021 |
| HOXA5 | 0.9375 | 0.8785111 | 5.17443 | 6.35E-07 | 4.40E-06 | 5.43106 |
| ARID5A | 0.93884 | 4.1683289 | 8.28919 | 3.23E-14 | 1.51E-12 | 21.782 |
| AJUBA | 0.93885 | 1.9069669 | 9.97746 | 9.19E-19 | 1.25E-16 | 32.0521 |
| SNORA73B | 0.93927 | 3.2207272 | 2.79736 | 0.005743 | 0.0139981 | -3.1465 |
| PTRF | 0.93944 | 4.4216639 | 5.79167 | 3.27E-08 | 3.14E-07 | 8.2949 |
| PDCD1LG2 | 0.94185 | 1.1562951 | 9.48955 | 2.01E-17 | 2.03E-15 | 29.0211 |
| FAM111A | 0.9449 | 3.6027188 | 8.77581 | 1.69E-15 | 1.10E-13 | 24.6736 |
| SQRDL | 0.94512 | 3.0249894 | 7.94359 | 2.52E-13 | 9.51E-12 | 19.7704 |
| TROAP | 0.94543 | 2.0864731 | 5.56346 | 1.00E-07 | 8.51E-07 | 7.20996 |
| CTSZ | 0.9456 | 6.1499446 | 6.93956 | 7.80E-11 | 1.50E-09 | 14.1645 |
| SASH3 | 0.94641 | 3.1499786 | 6.93778 | 7.88E-11 | 1.51E-09 | 14.155 |
| DLGAP5 | 0.94652 | 1.21058 | 7.16284 | 2.25E-11 | 5.06E-10 | 15.3773 |
| VCL | 0.94764 | 4.1873935 | 8.54698 | 6.82E-15 | 3.82E-13 | 23.3057 |
| GBP5 | 0.94892 | 1.2527192 | 7.30036 | 1.04E-11 | 2.55E-10 | 16.1345 |
| SLC47A2 | 0.9493 | 1.5051315 | 5.05949 | 1.08E-06 | 7.05E-06 | 4.92328 |
| IGFBP7-AS1 | 0.94944 | 1.6642269 | 5.44165 | 1.81E-07 | 1.43E-06 | 6.6432 |
| F2RL1 | 0.94952 | 1.7162339 | 7.35415 | 7.64E-12 | 1.94E-10 | 16.4326 |
| CDKN2C | 0.94967 | 4.3326848 | 5.51747 | 1.25E-07 | 1.03E-06 | 6.99495 |
| NUF2 | 0.94986 | 2.1862474 | 6.68312 | 3.17E-10 | 5.07E-09 | 12.7983 |
| LAT2 | 0.95047 | 3.928918 | 6.85503 | 1.24E-10 | 2.25E-09 | 13.7109 |
| ASF1B | 0.95053 | 2.1133588 | 6.43245 | 1.21E-09 | 1.67E-08 | 11.4918 |
| LCP2 | 0.95055 | 4.13471 | 7.50763 | 3.18E-12 | 9.06E-11 | 17.2896 |
| SPATA17 | 0.95095 | 1.3038901 | 8.54917 | 6.73E-15 | 3.79E-13 | 23.3187 |
| TPX2 | 0.95126 | 2.8439044 | 5.55514 | 1.05E-07 | 8.81E-07 | 7.17098 |
| COL8A2 | 0.95319 | 1.8335673 | 7.60315 | 1.83E-12 | 5.58E-11 | 17.8274 |
| RAB34 | 0.9539 | 3.9583711 | 6.01894 | 1.04E-08 | 1.14E-07 | 9.40413 |
| HSPG2 | 0.95395 | 3.10715 | 5.11448 | 8.38E-07 | 5.62E-06 | 5.16517 |
| PTPN6 | 0.95463 | 3.9309532 | 7.76418 | 7.20E-13 | 2.41E-11 | 18.7414 |
| HERC5 | 0.95466 | 2.6176504 | 7.1649 | 2.23E-11 | 5.01E-10 | 15.3886 |
| GNAI3 | 0.95482 | 4.94417 | 12.4645 | 8.82E-26 | 5.96E-23 | 47.9327 |
| RP11-347E10.1 | 0.95512 | 0.9373689 | 7.23131 | 1.53E-11 | 3.59E-10 | 15.7533 |
| MUC1 | 0.95553 | 3.0286839 | 7.44923 | 4.44E-12 | 1.21E-10 | 16.9625 |
| ADORA3 | 0.95605 | 5.1425561 | 6.36299 | 1.75E-09 | 2.34E-08 | 11.1351 |
| DRAXIN | 0.95608 | 1.5515661 | 6.43932 | 1.17E-09 | 1.62E-08 | 11.5272 |
| CCR1 | 0.95839 | 2.9364334 | 6.59152 | 5.20E-10 | 7.86E-09 | 12.3174 |
| VASN | 0.95875 | 1.9530279 | 5.59567 | 8.59E-08 | 7.42E-07 | 7.36131 |
| RRM2 | 0.95886 | 2.7862044 | 4.9122 | 2.09E-06 | 1.28E-05 | 4.28493 |
| PTPN7 | 0.95941 | 1.440329 | 7.94891 | 2.44E-13 | 9.30E-12 | 19.801 |
| SP140L | 0.95974 | 2.1854872 | 9.14377 | 1.74E-16 | 1.40E-14 | 26.9007 |
| WWTR1 | 0.95991 | 4.5340978 | 5.86139 | 2.31E-08 | 2.30E-07 | 8.6322 |
| LINC01057 | 0.96108 | 1.7418245 | 7.88914 | 3.47E-13 | 1.27E-11 | 19.4569 |
| EMILIN2 | 0.96227 | 2.1629512 | 6.38272 | 1.58E-09 | 2.12E-08 | 11.2362 |
| ODF3B | 0.96248 | 3.015488 | 6.28421 | 2.65E-09 | 3.38E-08 | 10.7334 |
| EN1 | 0.96326 | 0.8218616 | 5.8561 | 2.37E-08 | 2.36E-07 | 8.60652 |
| TACC3 | 0.96348 | 3.2446634 | 6.47508 | 9.68E-10 | 1.37E-08 | 11.7119 |
| HMCN2 | 0.96447 | 1.4250051 | 5.19318 | 5.82E-07 | 4.08E-06 | 5.51468 |
| CTBS | 0.96533 | 3.1893357 | 10.2031 | 2.18E-19 | 3.43E-17 | 33.4673 |
| C3AR1 | 0.96533 | 4.1424381 | 6.14696 | 5.40E-09 | 6.33E-08 | 10.0411 |
| SPI1 | 0.96567 | 4.6618747 | 6.89661 | 9.89E-11 | 1.83E-09 | 13.9337 |
| AEBP1 | 0.96604 | 5.4314857 | 3.94797 | 0.000115 | 0.000451 | 0.47277 |
| MIA | 0.96608 | 1.2915274 | 5.84106 | 2.56E-08 | 2.53E-07 | 8.53357 |
| CD300A | 0.96619 | 3.2078219 | 8.07326 | 1.17E-13 | 4.90E-12 | 20.5207 |
| PLEK | 0.96656 | 3.1747547 | 6.65944 | 3.60E-10 | 5.67E-09 | 12.6736 |
| RARRES3 | 0.9673 | 5.7506881 | 6.53103 | 7.18E-10 | 1.05E-08 | 12.002 |
| FAM129A | 0.96761 | 2.2852282 | 6.49505 | 8.70E-10 | 1.24E-08 | 11.8152 |
| CYTL1 | 0.96766 | 3.0622939 | 7.66567 | 1.28E-12 | 4.02E-11 | 18.1811 |
| SLC16A4 | 0.96795 | 2.9607922 | 7.25432 | 1.35E-11 | 3.20E-10 | 15.8801 |
| TCIRG1 | 0.96818 | 4.0289111 | 8.32248 | 2.64E-14 | 1.27E-12 | 21.9776 |
| LRRC42 | 0.96833 | 3.9680974 | 13.7602 | 1.77E-29 | 6.15E-26 | 56.2995 |
| KIAA0040 | 0.96888 | 2.4326944 | 6.58186 | 5.47E-10 | 8.22E-09 | 12.2669 |
| TXLNA | 0.96946 | 4.1667848 | 12.8778 | 5.83E-27 | 6.16E-24 | 50.6031 |
| GNL2 | 0.96953 | 4.5123137 | 14.9227 | 8.84E-33 | 5.38E-29 | 63.7622 |
| FPR3 | 0.96956 | 2.1334371 | 5.83543 | 2.63E-08 | 2.59E-07 | 8.50628 |
| CDK2 | 0.96977 | 3.7813955 | 7.07631 | 3.65E-11 | 7.73E-10 | 14.9048 |
| HCST | 0.97036 | 3.6988837 | 6.62489 | 4.34E-10 | 6.68E-09 | 12.4921 |
| PLAC8 | 0.97149 | 1.340893 | 6.76734 | 2.01E-10 | 3.41E-09 | 13.2437 |
| FCGR1B | 0.97167 | 3.9128151 | 5.87025 | 2.21E-08 | 2.22E-07 | 8.67523 |
| CDK1 | 0.97465 | 2.8674804 | 5.48476 | 1.47E-07 | 1.19E-06 | 6.84279 |
| IGKV3-20 | 0.97534 | 1.1171832 | 3.96833 | 0.000106 | 0.0004207 | 0.54633 |
| FCGR2B | 0.97557 | 1.9500134 | 4.66147 | 6.30E-06 | 3.42E-05 | 3.23111 |
| DEF6 | 0.97577 | 2.8333928 | 8.36211 | 2.08E-14 | 1.03E-12 | 22.211 |
| CTSH | 0.97635 | 6.7076403 | 4.85784 | 2.67E-06 | 1.59E-05 | 4.05288 |
| SNORD3B-2 | 0.97662 | 0.847815 | 6.67702 | 3.28E-10 | 5.22E-09 | 12.7661 |
| TPTEP1 | 0.97709 | 3.5924673 | 2.86428 | 0.004704 | 0.0117733 | -2.9661 |
| PTPRC | 0.97847 | 3.353006 | 7.01189 | 5.23E-11 | 1.05E-09 | 14.5551 |
| VEGFA | 0.97859 | 5.5468283 | 4.11324 | 6.05E-05 | 0.0002546 | 1.07875 |
| PARP9 | 0.979 | 4.263996 | 8.2958 | 3.10E-14 | 1.46E-12 | 21.8208 |
| C7orf57 | 0.98115 | 0.873035 | 5.93906 | 1.56E-08 | 1.63E-07 | 9.01108 |
| NLRC5 | 0.98188 | 3.4433555 | 7.85399 | 4.26E-13 | 1.53E-11 | 19.2551 |
| CHRNA9 | 0.98242 | 0.8859351 | 5.82615 | 2.75E-08 | 2.70E-07 | 8.46138 |
| TNFAIP2 | 0.9826 | 4.3002278 | 6.3493 | 1.88E-09 | 2.50E-08 | 11.0651 |
| LILRB4 | 0.98295 | 4.6260705 | 6.01853 | 1.04E-08 | 1.14E-07 | 9.40212 |
| MMP7 | 0.98309 | 0.7619735 | 5.49465 | 1.40E-07 | 1.14E-06 | 6.88873 |
| PRDX1 | 0.9833 | 8.1673926 | 7.30764 | 9.95E-12 | 2.45E-10 | 16.1747 |
| RP3-460G2.2 | 0.98391 | 0.8114381 | 6.11882 | 6.24E-09 | 7.21E-08 | 9.90039 |
| CLEC7A | 0.98427 | 2.7368009 | 6.47898 | 9.48E-10 | 1.34E-08 | 11.732 |
| CRB2 | 0.98442 | 2.9264034 | 5.4424 | 1.80E-07 | 1.43E-06 | 6.64666 |
| PYCARD | 0.98558 | 3.6868971 | 8.04341 | 1.40E-13 | 5.70E-12 | 20.3475 |
| BCL2L12 | 0.98565 | 2.4775735 | 10.0901 | 4.48E-19 | 6.49E-17 | 32.7574 |
| PTGS1 | 0.98645 | 3.02364 | 6.89222 | 1.01E-10 | 1.87E-09 | 13.9101 |
| HSPB6 | 0.98667 | 4.1951999 | 4.35972 | 2.24E-05 | 0.0001059 | 2.0199 |
| FERMT3 | 0.98688 | 3.555059 | 7.79868 | 5.89E-13 | 2.02E-11 | 18.9384 |
| CYP21A2 | 0.98748 | 1.535245 | 8.39413 | 1.72E-14 | 8.72E-13 | 22.3999 |
| CCL5 | 0.98768 | 2.1465277 | 5.67142 | 5.93E-08 | 5.36E-07 | 7.71951 |
| ZNF662 | 0.98811 | 2.0396514 | 8.45696 | 1.18E-14 | 6.26E-13 | 22.7714 |
| PSORS1C1 | 0.98815 | 1.6595654 | 5.68983 | 5.41E-08 | 4.96E-07 | 7.80707 |
| GBE1 | 0.9886 | 3.8700172 | 9.49728 | 1.92E-17 | 1.94E-15 | 29.0688 |
| FAM20C | 0.98863 | 4.8725473 | 6.90631 | 9.37E-11 | 1.75E-09 | 13.9857 |
| GPNMB | 0.98997 | 4.4209804 | 3.74394 | 0.000247 | 0.000888 | -0.2466 |
| CLEC2B | 0.99082 | 2.1435981 | 8.17564 | 6.36E-14 | 2.82E-12 | 21.1169 |
| TM4SF1 | 0.99096 | 3.840698 | 5.5677 | 9.84E-08 | 8.36E-07 | 7.22985 |
| FRMD3 | 0.9917 | 3.5105072 | 7.21971 | 1.64E-11 | 3.80E-10 | 15.6895 |
| SYNM | 0.9923 | 4.9043535 | 6.79245 | 1.75E-10 | 3.03E-09 | 13.3772 |
| AURKB | 0.99341 | 2.4124184 | 5.87071 | 2.20E-08 | 2.21E-07 | 8.67746 |
| S100A2 | 0.99427 | 2.0944689 | 7.52335 | 2.90E-12 | 8.38E-11 | 17.3778 |
| HSPA6 | 0.99505 | 1.6615489 | 5.98275 | 1.25E-08 | 1.34E-07 | 9.22564 |
| AC008964.1 | 0.99595 | 2.8093459 | 6.9341 | 8.04E-11 | 1.53E-09 | 14.1351 |
| SNHG3 | 0.99752 | 4.276181 | 5.43254 | 1.89E-07 | 1.49E-06 | 6.60116 |
| AL163636.6 | 0.99783 | 0.9586383 | 7.00849 | 5.33E-11 | 1.07E-09 | 14.5367 |
| MAGOH | 0.99797 | 5.1126707 | 18.6224 | 5.47E-43 | 1.33E-38 | 86.7786 |
| CSTA | 0.99851 | 1.4734066 | 6.50869 | 8.09E-10 | 1.16E-08 | 11.886 |
| C5AR1 | 0.99906 | 2.4748998 | 6.28543 | 2.63E-09 | 3.36E-08 | 10.7397 |
| MELK | 1.00021 | 1.7164959 | 6.64853 | 3.82E-10 | 5.96E-09 | 12.6162 |
| WLS | 1.00155 | 6.8269576 | 8.8531 | 1.05E-15 | 7.26E-14 | 25.1387 |
| CCNB1 | 1.00227 | 3.403788 | 7.44814 | 4.47E-12 | 1.22E-10 | 16.9563 |
| CLEC5A | 1.0023 | 1.0614756 | 6.11112 | 6.50E-09 | 7.48E-08 | 9.86194 |
| UPP1 | 1.0028 | 3.9908889 | 7.35504 | 7.60E-12 | 1.93E-10 | 16.4375 |
| HGF | 1.00376 | 1.9721595 | 6.84849 | 1.29E-10 | 2.32E-09 | 13.676 |
| C1RL | 1.00616 | 2.9852465 | 6.59697 | 5.05E-10 | 7.66E-09 | 12.3459 |
| HLA-B | 1.00618 | 7.8154701 | 5.63695 | 7.02E-08 | 6.22E-07 | 7.55609 |
| PHTF1 | 1.00835 | 3.1891739 | 13.8731 | 8.43E-30 | 3.42E-26 | 57.027 |
| MMP9 | 1.00916 | 1.3305377 | 3.83087 | 0.000179 | 0.0006677 | 0.05597 |
| APOBEC3G | 1.00953 | 2.419589 | 8.68782 | 2.89E-15 | 1.79E-13 | 24.146 |
| HS3ST1 | 1.00961 | 2.1008974 | 7.2086 | 1.74E-11 | 4.03E-10 | 15.6284 |
| FCGR1C | 1.01014 | 2.6870541 | 6.34668 | 1.91E-09 | 2.53E-08 | 11.0517 |
| SOCS2 | 1.01031 | 3.5195098 | 5.68265 | 5.61E-08 | 5.11E-07 | 7.77291 |
| HLA-A | 1.01037 | 8.010437 | 5.89434 | 1.96E-08 | 2.00E-07 | 8.79252 |
| PSRC1 | 1.01434 | 5.2377993 | 7.18689 | 1.97E-11 | 4.48E-10 | 15.5092 |
| ADM | 1.01455 | 2.9542049 | 5.04475 | 1.15E-06 | 7.50E-06 | 4.85873 |
| FAM64A | 1.01461 | 2.5920213 | 5.30997 | 3.37E-07 | 2.51E-06 | 6.04043 |
| GLIS3 | 1.01603 | 3.5418177 | 6.64786 | 3.84E-10 | 5.98E-09 | 12.6127 |
| CCNL2 | 1.01618 | 6.4602857 | 11.2217 | 3.02E-22 | 9.36E-20 | 39.9346 |
| TMSB4X | 1.01676 | 11.465476 | 9.43441 | 2.84E-17 | 2.77E-15 | 28.6813 |
| S100A9 | 1.01737 | 4.1877134 | 3.86595 | 0.000157 | 0.0005944 | 0.17975 |
| LAIR1 | 1.01784 | 4.2962082 | 6.62493 | 4.34E-10 | 6.68E-09 | 12.4923 |
| APOC2 | 1.01847 | 5.5479547 | 5.2669 | 4.13E-07 | 3.00E-06 | 5.84556 |
| DDIT4L | 1.02002 | 1.3658268 | 5.61335 | 7.88E-08 | 6.89E-07 | 7.44461 |
| TMEM176B | 1.02059 | 5.3433217 | 5.6546 | 6.44E-08 | 5.77E-07 | 7.6397 |
| PTGFRN | 1.02248 | 3.8566325 | 7.79956 | 5.86E-13 | 2.02E-11 | 18.9435 |
| CD68 | 1.02293 | 6.2660038 | 5.93339 | 1.61E-08 | 1.67E-07 | 8.98331 |
| CD69 | 1.0232 | 1.4750784 | 7.50604 | 3.21E-12 | 9.12E-11 | 17.2806 |
| RBMS1 | 1.02904 | 3.4557851 | 9.35342 | 4.72E-17 | 4.37E-15 | 28.1834 |
| ITGB3BP | 1.02951 | 3.4804701 | 12.2161 | 4.51E-25 | 2.61E-22 | 46.3294 |
| IGLV2-14 | 1.02982 | 1.1110714 | 4.14559 | 5.33E-05 | 0.0002278 | 1.19974 |
| SNHG12 | 1.02991 | 4.7661647 | 7.67774 | 1.19E-12 | 3.76E-11 | 18.2496 |
| IGHG3 | 1.03013 | 1.1883576 | 3.99519 | 9.59E-05 | 0.0003835 | 0.6438 |
| IQGAP1 | 1.03026 | 4.9336358 | 8.79906 | 1.46E-15 | 9.72E-14 | 24.8134 |
| ICAM1 | 1.0314 | 2.9963959 | 5.35554 | 2.72E-07 | 2.07E-06 | 6.24782 |
| ANG | 1.03166 | 1.8779225 | 8.56464 | 6.12E-15 | 3.49E-13 | 23.4108 |
| PARVG | 1.03167 | 3.6413648 | 7.42232 | 5.18E-12 | 1.39E-10 | 16.8121 |
| OLFML3 | 1.03199 | 4.758784 | 8.07371 | 1.17E-13 | 4.90E-12 | 20.5233 |
| AGTRAP | 1.0338 | 4.6927178 | 10.1316 | 3.44E-19 | 5.07E-17 | 33.0177 |
| IFI6 | 1.03475 | 7.1776025 | 5.97302 | 1.32E-08 | 1.40E-07 | 9.17776 |
| TNFSF13B | 1.03746 | 3.0446616 | 5.58023 | 9.26E-08 | 7.92E-07 | 7.28867 |
| HDAC1 | 1.0384 | 5.0348353 | 12.5525 | 4.95E-26 | 3.65E-23 | 48.5015 |
| TMSB4XP6 | 1.03926 | 4.0372318 | 2.26932 | 0.024499 | 0.0492365 | -4.4309 |
| ZNF436 | 1.03994 | 3.8845558 | 11.5849 | 2.82E-23 | 1.02E-20 | 42.2641 |
| IFI16 | 1.04112 | 5.7172437 | 8.37728 | 1.90E-14 | 9.53E-13 | 22.3005 |
| TMSB4XP8 | 1.04192 | 3.5563915 | 6.99453 | 5.76E-11 | 1.14E-09 | 14.4612 |
| RFTN1 | 1.04303 | 4.1130982 | 7.12279 | 2.82E-11 | 6.14E-10 | 15.1582 |
| CDCA8 | 1.04305 | 1.6606404 | 8.00301 | 1.77E-13 | 6.97E-12 | 20.1135 |
| SEC61G | 1.04387 | 7.0289909 | 6.24201 | 3.30E-09 | 4.11E-08 | 10.5196 |
| C2 | 1.04489 | 3.2517265 | 7.27911 | 1.17E-11 | 2.83E-10 | 16.0169 |
| MYCBP | 1.04509 | 2.7257013 | 11.9376 | 2.80E-24 | 1.42E-21 | 44.5337 |
| PLOD1 | 1.04562 | 5.3847024 | 9.5099 | 1.77E-17 | 1.82E-15 | 29.1466 |
| RCC1 | 1.04729 | 3.5478553 | 9.47321 | 2.23E-17 | 2.23E-15 | 28.9204 |
| MAOB | 1.04789 | 4.8869699 | 3.39105 | 0.000865 | 0.0026683 | -1.4138 |
| SLC1A5 | 1.04844 | 3.0731773 | 8.03599 | 1.46E-13 | 5.88E-12 | 20.3045 |
| CTHRC1 | 1.04853 | 2.0483223 | 5.82822 | 2.73E-08 | 2.67E-07 | 8.47139 |
| HOXA7 | 1.04868 | 1.0684119 | 5.211 | 5.36E-07 | 3.78E-06 | 5.59435 |
| NETO2 | 1.04992 | 3.693765 | 6.488 | 9.04E-10 | 1.29E-08 | 11.7787 |
| PDGFD | 1.05076 | 1.4581648 | 7.53906 | 2.65E-12 | 7.73E-11 | 17.4661 |
| LAMA4 | 1.05172 | 4.0171746 | 7.96171 | 2.26E-13 | 8.69E-12 | 19.8749 |
| JUN | 1.05205 | 6.5088645 | 7.58351 | 2.05E-12 | 6.16E-11 | 17.7165 |
| TCEA3 | 1.05228 | 2.2252251 | 7.44079 | 4.66E-12 | 1.26E-10 | 16.9153 |
| CLIC4 | 1.05319 | 6.7539648 | 8.65756 | 3.48E-15 | 2.11E-13 | 23.965 |
| BIRC5 | 1.05346 | 2.4002164 | 5.64632 | 6.70E-08 | 5.98E-07 | 7.60046 |
| LPAR6 | 1.05431 | 4.0462065 | 7.92028 | 2.89E-13 | 1.08E-11 | 19.6361 |
| ALOX5 | 1.05513 | 3.4807623 | 8.00773 | 1.72E-13 | 6.79E-12 | 20.1409 |
| FAM183A | 1.05525 | 0.9588216 | 5.41834 | 2.02E-07 | 1.58E-06 | 6.53571 |
| MYBL2 | 1.05571 | 2.1400749 | 5.27194 | 4.03E-07 | 2.94E-06 | 5.86828 |
| VAMP8 | 1.0571 | 4.7379331 | 8.03883 | 1.43E-13 | 5.82E-12 | 20.3209 |
| GDF15 | 1.05735 | 1.6488478 | 5.53365 | 1.16E-07 | 9.61E-07 | 7.07046 |
| PVT1 | 1.05765 | 1.9391611 | 6.8411 | 1.34E-10 | 2.41E-09 | 13.6365 |
| FNBP1L | 1.05775 | 3.9396006 | 8.16702 | 6.70E-14 | 2.96E-12 | 21.0666 |
| CD53 | 1.05797 | 5.2257932 | 7.28637 | 1.12E-11 | 2.73E-10 | 16.0571 |
| HCLS1 | 1.05828 | 4.8392291 | 8.22443 | 4.76E-14 | 2.16E-12 | 21.4022 |
| SAMD9L | 1.05849 | 2.8685556 | 7.91957 | 2.90E-13 | 1.08E-11 | 19.632 |
| HOXA10 | 1.05957 | 0.9647238 | 5.48158 | 1.49E-07 | 1.20E-06 | 6.82803 |
| UCP2 | 1.05963 | 5.0217077 | 6.22477 | 3.61E-09 | 4.46E-08 | 10.4325 |
| GPX7 | 1.06445 | 3.3780191 | 10.8702 | 2.96E-21 | 7.68E-19 | 37.6903 |
| FN1 | 1.06512 | 6.8610452 | 5.36057 | 2.66E-07 | 2.02E-06 | 6.27079 |
| NEAT1 | 1.06765 | 6.0505311 | 6.22186 | 3.66E-09 | 4.50E-08 | 10.4177 |
| SAMSN1 | 1.06857 | 3.422481 | 7.44739 | 4.49E-12 | 1.22E-10 | 16.9521 |
| CTSS | 1.06928 | 5.1143499 | 6.70006 | 2.89E-10 | 4.69E-09 | 12.8876 |
| ANKRD22 | 1.06938 | 1.8399718 | 6.35223 | 1.85E-09 | 2.46E-08 | 11.0801 |
| ARPC1B | 1.06939 | 5.7980191 | 8.79261 | 1.52E-15 | 1.00E-13 | 24.7746 |
| DDR2 | 1.06949 | 3.6375455 | 6.90291 | 9.55E-11 | 1.78E-09 | 13.9675 |
| GAPT | 1.07212 | 1.5828593 | 8.39488 | 1.71E-14 | 8.70E-13 | 22.4044 |
| COL27A1 | 1.07241 | 2.3840487 | 8.50377 | 8.86E-15 | 4.87E-13 | 23.049 |
| RHOC | 1.07323 | 7.584755 | 10.8959 | 2.51E-21 | 6.85E-19 | 37.8539 |
| NDC80 | 1.07383 | 1.7647267 | 7.50859 | 3.16E-12 | 9.04E-11 | 17.295 |
| GPX8 | 1.07557 | 1.4291422 | 6.52424 | 7.45E-10 | 1.08E-08 | 11.9667 |
| ADAMTS15 | 1.07619 | 1.7767274 | 6.09192 | 7.17E-09 | 8.17E-08 | 9.76621 |
| CD93 | 1.0765 | 2.6673921 | 5.71214 | 4.85E-08 | 4.48E-07 | 7.91345 |
| IGFBP3 | 1.07766 | 4.3863423 | 4.05907 | 7.49E-05 | 0.0003077 | 0.87787 |
| RP4-668G5.1 | 1.07819 | 2.7138356 | 6.5646 | 6.00E-10 | 8.92E-09 | 12.1769 |
| BCL2A1 | 1.079 | 1.9673591 | 5.99686 | 1.17E-08 | 1.25E-07 | 9.29513 |
| S100A8 | 1.07903 | 3.2601478 | 4.22324 | 3.91E-05 | 0.0001729 | 1.4933 |
| IGFBP5 | 1.08094 | 6.1257315 | 5.87827 | 2.12E-08 | 2.14E-07 | 8.71425 |
| SAA1 | 1.0821 | 0.9019101 | 3.88773 | 0.000145 | 0.0005529 | 0.25704 |
| AC002456.2 | 1.08243 | 1.8584437 | 8.36011 | 2.11E-14 | 1.04E-12 | 22.1992 |
| F13A1 | 1.08303 | 2.9257415 | 4.73035 | 4.67E-06 | 2.61E-05 | 3.51642 |
| SPON2 | 1.08421 | 2.6640253 | 5.21353 | 5.30E-07 | 3.74E-06 | 5.60565 |
| KLHDC8A | 1.085 | 4.3014581 | 5.58224 | 9.17E-08 | 7.86E-07 | 7.2981 |
| CRYZ | 1.0866 | 4.17554 | 12.3313 | 2.12E-25 | 1.38E-22 | 47.0727 |
| C1S | 1.0869 | 5.2639993 | 5.97122 | 1.33E-08 | 1.41E-07 | 9.16893 |
| STK40 | 1.08848 | 4.2028904 | 11.5966 | 2.61E-23 | 9.63E-21 | 42.3392 |
| ESM1 | 1.08884 | 1.402349 | 4.62597 | 7.33E-06 | 3.93E-05 | 3.08534 |
| LRRN4CL | 1.08897 | 1.4641113 | 6.83274 | 1.40E-10 | 2.51E-09 | 13.5918 |
| IFI44L | 1.08942 | 4.1575844 | 6.41152 | 1.36E-09 | 1.84E-08 | 11.3841 |
| MAP3K7CL | 1.08967 | 2.2190882 | 6.71226 | 2.71E-10 | 4.42E-09 | 12.952 |
| IBSP | 1.09039 | 0.9362325 | 4.78751 | 3.64E-06 | 2.09E-05 | 3.75561 |
| PLA2G2A | 1.09102 | 0.8817503 | 4.74131 | 4.45E-06 | 2.50E-05 | 3.56209 |
| DPYD | 1.09124 | 2.5761648 | 7.82884 | 4.94E-13 | 1.74E-11 | 19.111 |
| OR4N2 | 1.09342 | 1.3356573 | 5.38742 | 2.34E-07 | 1.80E-06 | 6.39368 |
| LUM | 1.0937 | 2.6927647 | 5.06474 | 1.05E-06 | 6.90E-06 | 4.94625 |
| RAB32 | 1.09555 | 3.0341625 | 8.33069 | 2.52E-14 | 1.21E-12 | 22.026 |
| SP100 | 1.09803 | 3.9377299 | 8.8609 | 1.00E-15 | 6.97E-14 | 25.1857 |
| RNASE4 | 1.09987 | 2.5209032 | 9.05835 | 2.96E-16 | 2.32E-14 | 26.3808 |
| VSIG4 | 1.1017 | 5.2938887 | 5.97655 | 1.29E-08 | 1.38E-07 | 9.19513 |
| BATF3 | 1.10252 | 2.5387898 | 9.58745 | 1.09E-17 | 1.15E-15 | 29.6259 |
| WDR78 | 1.10494 | 1.8368747 | 9.66431 | 6.69E-18 | 7.54E-16 | 30.1019 |
| NMI | 1.10731 | 2.9263954 | 10.4026 | 6.05E-20 | 1.07E-17 | 34.7247 |
| PKIB | 1.10815 | 2.7081696 | 5.97146 | 1.33E-08 | 1.41E-07 | 9.17008 |
| WEE1 | 1.10874 | 3.2998203 | 7.02208 | 4.94E-11 | 1.00E-09 | 14.6103 |
| C1QC | 1.10919 | 7.3471063 | 7.03422 | 4.62E-11 | 9.50E-10 | 14.6761 |
| LAPTM5 | 1.10947 | 6.8406549 | 7.41824 | 5.30E-12 | 1.41E-10 | 16.7894 |
| PGAM2 | 1.10951 | 3.4356527 | 5.2865 | 3.77E-07 | 2.77E-06 | 5.93409 |
| CLCF1 | 1.11107 | 1.1575952 | 7.66271 | 1.30E-12 | 4.07E-11 | 18.1643 |
| IL18 | 1.11164 | 3.5135265 | 7.59274 | 1.95E-12 | 5.87E-11 | 17.7686 |
| AIF1 | 1.11251 | 6.1810353 | 7.55237 | 2.46E-12 | 7.25E-11 | 17.541 |
| HMOX1 | 1.11306 | 4.8362684 | 5.36001 | 2.66E-07 | 2.03E-06 | 6.26826 |
| TOP2A | 1.11462 | 3.1755073 | 5.22297 | 5.07E-07 | 3.60E-06 | 5.64794 |
| PDLIM1 | 1.11488 | 2.995162 | 5.64472 | 6.76E-08 | 6.02E-07 | 7.59289 |
| LAMB1 | 1.11539 | 3.9600489 | 6.27454 | 2.78E-09 | 3.54E-08 | 10.6844 |
| C1orf162 | 1.11776 | 3.7450505 | 8.14755 | 7.52E-14 | 3.30E-12 | 20.953 |
| IGLC1 | 1.11779 | 1.4108008 | 3.72413 | 0.000266 | 0.0009475 | -0.3148 |
| IFI44 | 1.11908 | 4.6988551 | 8.25601 | 3.94E-14 | 1.80E-12 | 21.5872 |
| CENPF | 1.12076 | 2.6709187 | 6.28943 | 2.58E-09 | 3.30E-08 | 10.7599 |
| ITGB4 | 1.12249 | 4.5634806 | 6.47355 | 9.76E-10 | 1.38E-08 | 11.704 |
| DOCK7 | 1.12506 | 4.2292247 | 11.5549 | 3.43E-23 | 1.23E-20 | 42.0713 |
| CAV1 | 1.12545 | 4.036287 | 5.77584 | 3.54E-08 | 3.37E-07 | 8.21868 |
| CCNB2 | 1.12693 | 2.2319047 | 6.24051 | 3.33E-09 | 4.14E-08 | 10.512 |
| MFAP2 | 1.12786 | 1.5406822 | 6.32402 | 2.15E-09 | 2.81E-08 | 10.936 |
| PTTG1 | 1.12912 | 3.9095675 | 6.47644 | 9.61E-10 | 1.36E-08 | 11.7189 |
| SLC16A3 | 1.13021 | 3.6806734 | 7.73694 | 8.44E-13 | 2.76E-11 | 18.5862 |
| LDHA | 1.13067 | 6.9879808 | 7.61085 | 1.75E-12 | 5.37E-11 | 17.8709 |
| ISG15 | 1.13485 | 5.6707554 | 6.29618 | 2.49E-09 | 3.20E-08 | 10.7943 |
| RP11-834C11.4 | 1.13736 | 2.8698742 | 7.89363 | 3.38E-13 | 1.24E-11 | 19.4827 |
| CAPG | 1.13764 | 5.4742043 | 6.30336 | 2.40E-09 | 3.10E-08 | 10.8308 |
| TYROBP | 1.13955 | 6.774016 | 7.89144 | 3.42E-13 | 1.25E-11 | 19.4702 |
| MS4A4A | 1.1411 | 3.5714059 | 6.25246 | 3.13E-09 | 3.92E-08 | 10.5724 |
| CTD-3049M7.1 | 1.1417 | 1.552345 | 4.46769 | 1.43E-05 | 7.14E-05 | 2.44596 |
| IL13RA2 | 1.14453 | 1.7962046 | 4.8644 | 2.59E-06 | 1.55E-05 | 4.08079 |
| PLOD2 | 1.14475 | 4.2557846 | 8.41843 | 1.48E-14 | 7.69E-13 | 22.5435 |
| FPR1 | 1.14545 | 3.641668 | 6.49055 | 8.91E-10 | 1.27E-08 | 11.7919 |
| SERPINA5 | 1.14623 | 1.2841794 | 5.99553 | 1.17E-08 | 1.26E-07 | 9.28857 |
| SRPX2 | 1.14921 | 1.6688826 | 6.05669 | 8.59E-09 | 9.60E-08 | 9.59106 |
| PRSS23 | 1.14956 | 4.3723429 | 6.51479 | 7.83E-10 | 1.13E-08 | 11.9177 |
| HIST2H2AA4 | 1.15103 | 4.3225753 | 8.06482 | 1.23E-13 | 5.10E-12 | 20.4717 |
| OSMR | 1.15214 | 2.762114 | 7.59343 | 1.94E-12 | 5.86E-11 | 17.7725 |
| MS4A7 | 1.15232 | 4.4252195 | 7.03557 | 4.58E-11 | 9.43E-10 | 14.6834 |
| FAM187A | 1.15533 | 2.8005937 | 6.97689 | 6.35E-11 | 1.25E-09 | 14.3658 |
| CEBPD | 1.16208 | 4.5441513 | 6.83027 | 1.42E-10 | 2.53E-09 | 13.5787 |
| CDC20 | 1.1624 | 2.5189837 | 6.84552 | 1.31E-10 | 2.36E-09 | 13.6601 |
| CYBA | 1.16484 | 5.8632532 | 7.83888 | 4.66E-13 | 1.66E-11 | 19.1685 |
| ASAP3 | 1.16614 | 4.3668238 | 10.8341 | 3.74E-21 | 9.28E-19 | 37.4609 |
| PLP2 | 1.1674 | 3.8412215 | 6.52504 | 7.42E-10 | 1.08E-08 | 11.9709 |
| TLR2 | 1.17073 | 2.5228966 | 7.77819 | 6.64E-13 | 2.25E-11 | 18.8214 |
| TEAD4 | 1.17091 | 1.8791616 | 7.20719 | 1.75E-11 | 4.05E-10 | 15.6206 |
| EMILIN1 | 1.17336 | 3.4561643 | 8.0149 | 1.65E-13 | 6.54E-12 | 20.1823 |
| ITGB2 | 1.17618 | 5.24754 | 7.51453 | 3.06E-12 | 8.80E-11 | 17.3283 |
| C1QB | 1.17764 | 7.6157899 | 7.27505 | 1.20E-11 | 2.87E-10 | 15.9945 |
| LY96 | 1.18144 | 3.5643621 | 7.7159 | 9.54E-13 | 3.09E-11 | 18.4664 |
| RP11-161H23.5 | 1.18333 | 1.6064176 | 4.83706 | 2.93E-06 | 1.72E-05 | 3.96472 |
| COL8A1 | 1.18664 | 1.267577 | 6.45265 | 1.09E-09 | 1.52E-08 | 11.596 |
| RP11-124N14.3 | 1.18685 | 1.2460358 | 9.34963 | 4.84E-17 | 4.46E-15 | 28.1601 |
| CASP1 | 1.18928 | 3.7488136 | 9.41442 | 3.22E-17 | 3.09E-15 | 28.5583 |
| CNN3 | 1.18979 | 8.1619363 | 9.97731 | 9.20E-19 | 1.25E-16 | 32.0512 |
| IGHA1 | 1.18994 | 1.7129335 | 4.21964 | 3.96E-05 | 0.0001751 | 1.4796 |
| PLSCR1 | 1.19133 | 4.6934485 | 8.58633 | 5.37E-15 | 3.12E-13 | 23.5399 |
| HIST1H2BK | 1.19211 | 4.1728159 | 9.44724 | 2.62E-17 | 2.58E-15 | 28.7603 |
| FCER1G | 1.19408 | 6.1203314 | 7.59262 | 1.95E-12 | 5.87E-11 | 17.7679 |
| BST2 | 1.19486 | 5.5594707 | 8.38945 | 1.77E-14 | 8.92E-13 | 22.3723 |
| HAS2 | 1.19518 | 1.9131222 | 7.8148 | 5.36E-13 | 1.87E-11 | 19.0306 |
| LEPREL1 | 1.19587 | 2.4311957 | 8.92485 | 6.76E-16 | 4.91E-14 | 25.5718 |
| CIITA | 1.1986 | 2.4326163 | 7.4491 | 4.45E-12 | 1.21E-10 | 16.9617 |
| NAMPT | 1.19967 | 5.6164266 | 6.34287 | 1.95E-09 | 2.58E-08 | 11.0323 |
| PCED1B-AS1 | 1.2088 | 3.9510269 | 8.06234 | 1.25E-13 | 5.16E-12 | 20.4573 |
| PLAUR | 1.20919 | 2.8973814 | 6.98455 | 6.08E-11 | 1.20E-09 | 14.4072 |
| KIF2C | 1.2112 | 1.9987648 | 8.14271 | 7.74E-14 | 3.38E-12 | 20.9248 |
| CYBB | 1.21674 | 4.2836029 | 7.49645 | 3.39E-12 | 9.57E-11 | 17.2269 |
| RDH10 | 1.21675 | 3.5771795 | 6.66374 | 3.52E-10 | 5.57E-09 | 12.6962 |
| TREM1 | 1.21902 | 1.6333434 | 5.66172 | 6.22E-08 | 5.59E-07 | 7.67344 |
| POSTN | 1.22183 | 1.2253866 | 4.42484 | 1.71E-05 | 8.33E-05 | 2.2759 |
| CTSC | 1.22317 | 4.8494932 | 7.94756 | 2.46E-13 | 9.33E-12 | 19.7932 |
| TREM2 | 1.22484 | 5.5417675 | 6.73714 | 2.37E-10 | 3.94E-09 | 13.0836 |
| TNFRSF11B | 1.22763 | 1.4099289 | 7.96124 | 2.27E-13 | 8.70E-12 | 19.8722 |
| LINC00152 | 1.22793 | 3.0101574 | 6.07277 | 7.91E-09 | 8.89E-08 | 9.67095 |
| MSN | 1.22806 | 5.797573 | 7.77678 | 6.69E-13 | 2.26E-11 | 18.8133 |
| COL1A2 | 1.22887 | 4.2555141 | 5.06213 | 1.06E-06 | 6.98E-06 | 4.93483 |
| S1PR3 | 1.2296 | 3.6584679 | 6.77109 | 1.97E-10 | 3.36E-09 | 13.2636 |
| HLA-DQA1 | 1.23201 | 3.2308087 | 3.64987 | 0.000348 | 0.001202 | -0.5674 |
| TUBB6 | 1.23301 | 4.326747 | 8.3469 | 2.28E-14 | 1.12E-12 | 22.1214 |
| TAGLN2 | 1.23386 | 6.1209613 | 6.85609 | 1.24E-10 | 2.24E-09 | 13.7166 |
| HLA-DOA | 1.23478 | 2.9718003 | 6.86671 | 1.17E-10 | 2.13E-09 | 13.7734 |
| RP1-261G23.7 | 1.23538 | 4.0741606 | 3.42233 | 0.000777 | 0.0024215 | -1.3144 |
| PLAT | 1.23553 | 3.39052 | 6.4115 | 1.36E-09 | 1.84E-08 | 11.384 |
| CASP4 | 1.23615 | 3.3507403 | 8.688 | 2.89E-15 | 1.79E-13 | 24.1471 |
| COL5A1 | 1.24015 | 1.9340405 | 6.3693 | 1.69E-09 | 2.27E-08 | 11.1674 |
| C1QA | 1.24041 | 6.954405 | 7.84111 | 4.60E-13 | 1.64E-11 | 19.1813 |
| HLA-DMA | 1.24272 | 5.2190349 | 7.072 | 3.74E-11 | 7.89E-10 | 14.8814 |
| RNASE2 | 1.2459 | 1.8530462 | 7.30169 | 1.03E-11 | 2.53E-10 | 16.1418 |
| TCTEX1D1 | 1.24649 | 1.4163718 | 6.25381 | 3.10E-09 | 3.90E-08 | 10.5793 |
| FAM181A | 1.24649 | 2.4718666 | 6.44876 | 1.11E-09 | 1.55E-08 | 11.5759 |
| AC099522.1 | 1.24919 | 1.9447065 | 9.01066 | 3.98E-16 | 3.05E-14 | 26.0913 |
| GPR65 | 1.254 | 1.6949685 | 8.48633 | 9.84E-15 | 5.32E-13 | 22.9455 |
| IQGAP2 | 1.25482 | 2.8638095 | 7.65152 | 1.39E-12 | 4.32E-11 | 18.101 |
| NAPSB | 1.25845 | 3.2808891 | 5.60585 | 8.17E-08 | 7.12E-07 | 7.40923 |
| NPNT | 1.26033 | 2.8546701 | 6.32216 | 2.17E-09 | 2.84E-08 | 10.9266 |
| FAM26F | 1.26275 | 2.4990955 | 8.35456 | 2.18E-14 | 1.08E-12 | 22.1665 |
| CD58 | 1.26275 | 2.9553248 | 10.822 | 4.04E-21 | 9.94E-19 | 37.3834 |
| STAB1 | 1.2645 | 5.0023522 | 8.04455 | 1.39E-13 | 5.67E-12 | 20.3541 |
| UBE2C | 1.26653 | 3.3329773 | 5.86563 | 2.26E-08 | 2.26E-07 | 8.65279 |
| RGS16 | 1.27037 | 2.6892162 | 6.96042 | 6.95E-11 | 1.35E-09 | 14.2769 |
| EFEMP1 | 1.27118 | 6.5002369 | 6.22453 | 3.61E-09 | 4.46E-08 | 10.4312 |
| ID3 | 1.27446 | 7.3478781 | 6.79712 | 1.71E-10 | 2.97E-09 | 13.402 |
| DNALI1 | 1.27525 | 4.6294598 | 11.1808 | 3.94E-22 | 1.20E-19 | 39.6732 |
| SFRP4 | 1.28663 | 3.0330004 | 6.08502 | 7.43E-09 | 8.43E-08 | 9.73184 |
| HLA-DMB | 1.28718 | 4.9003111 | 7.35408 | 7.64E-12 | 1.94E-10 | 16.4322 |
| RUNX1 | 1.28832 | 2.2886443 | 8.99554 | 4.37E-16 | 3.31E-14 | 25.9997 |
| ACTN1 | 1.29816 | 5.1585491 | 6.86382 | 1.18E-10 | 2.16E-09 | 13.758 |
| FSTL1 | 1.29896 | 4.9914916 | 8.02193 | 1.59E-13 | 6.32E-12 | 20.2231 |
| FCGR1A | 1.30022 | 5.2231149 | 6.94885 | 7.41E-11 | 1.43E-09 | 14.2146 |
| SERPINE1 | 1.30561 | 3.1723137 | 4.81686 | 3.20E-06 | 1.87E-05 | 3.87925 |
| MRC2 | 1.30756 | 4.3994675 | 7.48868 | 3.54E-12 | 9.96E-11 | 17.1833 |
| FCGR2A | 1.309 | 4.3844532 | 7.93999 | 2.57E-13 | 9.68E-12 | 19.7496 |
| HSPA7 | 1.30943 | 1.856909 | 6.68126 | 3.20E-10 | 5.12E-09 | 12.7884 |
| TGFBI | 1.31062 | 4.714059 | 5.86118 | 2.31E-08 | 2.31E-07 | 8.63117 |
| IGF2BP3 | 1.31133 | 1.3419418 | 7.24859 | 1.39E-11 | 3.29E-10 | 15.8485 |
| RP4-792G4.2 | 1.31341 | 1.4535857 | 7.62011 | 1.66E-12 | 5.12E-11 | 17.9232 |
| LEFTY2 | 1.31589 | 1.2704861 | 6.98879 | 5.94E-11 | 1.17E-09 | 14.4301 |
| LOXL2 | 1.31782 | 2.833214 | 7.22817 | 1.56E-11 | 3.64E-10 | 15.736 |
| SCIN | 1.32014 | 3.1645795 | 6.18068 | 4.54E-09 | 5.42E-08 | 10.2103 |
| PTX3 | 1.32073 | 1.8315997 | 6.21088 | 3.88E-09 | 4.74E-08 | 10.3623 |
| RP11-79P5.2 | 1.3228 | 1.9804713 | 6.94954 | 7.39E-11 | 1.43E-09 | 14.2183 |
| LOX | 1.32789 | 1.4349109 | 6.99559 | 5.72E-11 | 1.14E-09 | 14.4669 |
| S100A6 | 1.32915 | 8.1839065 | 8.45283 | 1.21E-14 | 6.40E-13 | 22.747 |
| HLA-DRB1 | 1.33284 | 5.279701 | 5.21028 | 5.38E-07 | 3.79E-06 | 5.5911 |
| FOXJ1 | 1.33525 | 2.3982296 | 6.65962 | 3.60E-10 | 5.67E-09 | 12.6745 |
| ALOX5AP | 1.33567 | 4.824765 | 7.02575 | 4.84E-11 | 9.88E-10 | 14.6302 |
| COL22A1 | 1.33615 | 2.1338829 | 6.51536 | 7.81E-10 | 1.13E-08 | 11.9206 |
| CD74 | 1.33713 | 9.4382638 | 6.54917 | 6.52E-10 | 9.61E-09 | 12.0964 |
| TGIF1 | 1.34007 | 4.1087254 | 10.8877 | 2.64E-21 | 7.06E-19 | 37.8017 |
| RPE65 | 1.34043 | 2.4477632 | 6.82092 | 1.50E-10 | 2.65E-09 | 13.5288 |
| PDIA5 | 1.34201 | 2.4485308 | 11.8382 | 5.37E-24 | 2.38E-21 | 43.8937 |
| UBD | 1.34522 | 2.2042937 | 6.5298 | 7.23E-10 | 1.06E-08 | 11.9956 |
| CARD16 | 1.34923 | 3.0980611 | 9.0272 | 3.59E-16 | 2.76E-14 | 26.1917 |
| IGLC3 | 1.34945 | 1.7356619 | 4.23816 | 3.68E-05 | 0.000164 | 1.5502 |
| GNG5 | 1.35106 | 6.1565497 | 13.5338 | 7.82E-29 | 1.73E-25 | 54.8396 |
| COL5A2 | 1.35222 | 2.8327006 | 7.02747 | 4.80E-11 | 9.80E-10 | 14.6395 |
| HOXD8 | 1.35256 | 1.5654473 | 6.25945 | 3.01E-09 | 3.80E-08 | 10.6079 |
| COL6A2 | 1.35541 | 3.9678172 | 6.0735 | 7.88E-09 | 8.87E-08 | 9.67458 |
| IGHG2 | 1.35767 | 1.6269407 | 4.31895 | 2.65E-05 | 0.0001225 | 1.86117 |
| CLIC1 | 1.35955 | 5.5872651 | 8.44251 | 1.28E-14 | 6.75E-13 | 22.6859 |
| CXCL10 | 1.35999 | 1.8283344 | 5.8472 | 2.48E-08 | 2.46E-07 | 8.56331 |
| CCL2 | 1.36065 | 5.2196445 | 4.78073 | 3.75E-06 | 2.15E-05 | 3.72712 |
| IGLC2 | 1.36479 | 1.580486 | 4.58567 | 8.71E-06 | 4.58E-05 | 2.92089 |
| H19 | 1.36484 | 1.9706634 | 4.81999 | 3.15E-06 | 1.84E-05 | 3.89249 |
| CCDC109B | 1.36697 | 2.652807 | 9.15075 | 1.67E-16 | 1.34E-14 | 26.9433 |
| C1orf194 | 1.36769 | 3.5281582 | 7.02149 | 4.96E-11 | 1.01E-09 | 14.6071 |
| STEAP3 | 1.36828 | 2.5523042 | 7.58158 | 2.08E-12 | 6.22E-11 | 17.7056 |
| SPP1 | 1.36838 | 9.1043593 | 5.25332 | 4.40E-07 | 3.17E-06 | 5.78435 |
| GADD45A | 1.37126 | 5.1631289 | 8.63669 | 3.95E-15 | 2.36E-13 | 23.8403 |
| PTGES3P1 | 1.37153 | 2.3538286 | 7.50374 | 3.25E-12 | 9.22E-11 | 17.2677 |
| SERPINA1 | 1.37358 | 3.8754801 | 7.22728 | 1.57E-11 | 3.65E-10 | 15.7312 |
| HOTAIRM1 | 1.37744 | 1.6697421 | 6.73501 | 2.39E-10 | 3.98E-09 | 13.0723 |
| MAN1C1 | 1.38073 | 4.2466776 | 8.25818 | 3.89E-14 | 1.79E-12 | 21.5999 |
| FABP5 | 1.38139 | 5.055273 | 5.14522 | 7.27E-07 | 4.97E-06 | 5.30119 |
| S100A3 | 1.38764 | 1.5502076 | 8.01603 | 1.64E-13 | 6.51E-12 | 20.1889 |
| FLNC | 1.38966 | 2.9072848 | 6.46362 | 1.03E-09 | 1.44E-08 | 11.6526 |
| HAMP | 1.39004 | 2.7663143 | 6.04608 | 9.07E-09 | 1.00E-07 | 9.53847 |
| SERPINH1 | 1.39148 | 4.6316012 | 7.63671 | 1.51E-12 | 4.68E-11 | 18.0171 |
| MMP14 | 1.39281 | 4.5771187 | 7.47982 | 3.73E-12 | 1.04E-10 | 17.1337 |
| PIFO | 1.39429 | 3.2124279 | 8.27132 | 3.59E-14 | 1.66E-12 | 21.677 |
| CSRP2 | 1.3977 | 6.2859648 | 8.34624 | 2.29E-14 | 1.12E-12 | 22.1175 |
| C4A | 1.39931 | 6.8799881 | 6.50811 | 8.12E-10 | 1.17E-08 | 11.883 |
| SOD2 | 1.40015 | 7.4990217 | 6.74118 | 2.31E-10 | 3.86E-09 | 13.105 |
| OLFML2B | 1.4009 | 3.0991968 | 9.69866 | 5.39E-18 | 6.18E-16 | 30.315 |
| S100A10 | 1.40365 | 6.7733112 | 6.43682 | 1.19E-09 | 1.64E-08 | 11.5143 |
| COL11A1 | 1.40556 | 2.6796494 | 7.55428 | 2.43E-12 | 7.18E-11 | 17.5518 |
| AC015936.3 | 1.40665 | 2.9076926 | 5.80358 | 3.08E-08 | 2.98E-07 | 8.35232 |
| GBP3 | 1.40914 | 3.6904598 | 8.57255 | 5.84E-15 | 3.35E-13 | 23.4579 |
| PYGL | 1.41413 | 3.8890857 | 10.1995 | 2.23E-19 | 3.43E-17 | 33.4446 |
| C4B | 1.41757 | 6.5267978 | 6.5099 | 8.04E-10 | 1.16E-08 | 11.8923 |
| SLC11A1 | 1.41935 | 3.463424 | 7.14277 | 2.52E-11 | 5.56E-10 | 15.2675 |
| RGS1 | 1.42235 | 5.0462919 | 5.36939 | 2.55E-07 | 1.95E-06 | 6.31112 |
| S100A11 | 1.4226 | 6.4945007 | 8.20632 | 5.30E-14 | 2.37E-12 | 21.2962 |
| GEM | 1.4291 | 3.6365327 | 8.66673 | 3.29E-15 | 2.01E-13 | 24.0198 |
| FCGR2C | 1.43169 | 2.165822 | 6.702 | 2.86E-10 | 4.65E-09 | 12.8978 |
| FLNA | 1.43473 | 6.7052888 | 9.58902 | 1.08E-17 | 1.14E-15 | 29.6355 |
| ECM2 | 1.43828 | 3.2104914 | 9.48324 | 2.09E-17 | 2.10E-15 | 28.9822 |
| CD14 | 1.43994 | 5.4357323 | 6.96362 | 6.83E-11 | 1.33E-09 | 14.2942 |
| HLA-DPB1 | 1.45793 | 5.4853046 | 6.67172 | 3.37E-10 | 5.36E-09 | 12.7382 |
| COL4A2 | 1.46283 | 4.2502669 | 5.77531 | 3.55E-08 | 3.38E-07 | 8.21611 |
| MOXD1 | 1.46893 | 2.1615849 | 5.65665 | 6.37E-08 | 5.72E-07 | 7.64942 |
| ABCC3 | 1.47129 | 1.9278521 | 6.95621 | 7.12E-11 | 1.38E-09 | 14.2542 |
| CLEC18B | 1.4759 | 2.1551379 | 9.3994 | 3.54E-17 | 3.37E-15 | 28.4659 |
| MIR4435-1HG | 1.4769 | 3.174591 | 7.13262 | 2.67E-11 | 5.83E-10 | 15.2119 |
| CA3 | 1.48107 | 1.4720702 | 7.19729 | 1.86E-11 | 4.26E-10 | 15.5663 |
| CD44 | 1.48119 | 6.0973671 | 6.35662 | 1.81E-09 | 2.42E-08 | 11.1025 |
| CD163 | 1.48915 | 3.8510396 | 5.96478 | 1.37E-08 | 1.45E-07 | 9.13726 |
| MSR1 | 1.48916 | 3.5918826 | 7.64506 | 1.44E-12 | 4.47E-11 | 18.0644 |
| COL1A1 | 1.49152 | 3.2812798 | 5.36001 | 2.66E-07 | 2.03E-06 | 6.26824 |
| SOCS3 | 1.4925 | 2.9816767 | 5.84077 | 2.56E-08 | 2.53E-07 | 8.53217 |
| DIRAS3 | 1.51086 | 2.3639103 | 9.69011 | 5.69E-18 | 6.46E-16 | 30.2619 |
| C1R | 1.51334 | 6.1571313 | 7.22732 | 1.57E-11 | 3.65E-10 | 15.7313 |
| S100A4 | 1.54266 | 3.5984656 | 7.31496 | 9.55E-12 | 2.37E-10 | 16.2153 |
| MS4A6A | 1.55953 | 5.1271829 | 6.29465 | 2.51E-09 | 3.22E-08 | 10.7865 |
| CFI | 1.56148 | 3.5676094 | 7.33694 | 8.43E-12 | 2.12E-10 | 16.3371 |
| HLA-DRA | 1.57358 | 7.3498213 | 7.01818 | 5.05E-11 | 1.02E-09 | 14.5892 |
| HLA-DPA1 | 1.57809 | 6.8948037 | 7.06486 | 3.89E-11 | 8.17E-10 | 14.8425 |
| SULF1 | 1.58061 | 3.8109623 | 7.61391 | 1.72E-12 | 5.29E-11 | 17.8882 |
| TNC | 1.58322 | 5.4347889 | 7.63038 | 1.57E-12 | 4.84E-11 | 17.9813 |
| C3 | 1.58556 | 7.6988823 | 8.452 | 1.21E-14 | 6.41E-13 | 22.7421 |
| APOL4 | 1.5865 | 2.2388329 | 8.39012 | 1.76E-14 | 8.90E-13 | 22.3763 |
| IFI30 | 1.59351 | 4.9901313 | 7.27686 | 1.18E-11 | 2.85E-10 | 16.0045 |
| MGP | 1.59549 | 4.9746173 | 5.80521 | 3.06E-08 | 2.96E-07 | 8.36021 |
| SLN | 1.60831 | 2.3013261 | 5.30135 | 3.51E-07 | 2.60E-06 | 6.00131 |
| GBP1 | 1.61068 | 3.9195096 | 8.07293 | 1.17E-13 | 4.91E-12 | 20.5188 |
| C8orf4 | 1.61249 | 3.5533569 | 8.22079 | 4.86E-14 | 2.20E-12 | 21.3809 |
| METTL7B | 1.61536 | 3.0190855 | 7.05631 | 4.08E-11 | 8.52E-10 | 14.7961 |
| TGFB2 | 1.61849 | 3.529529 | 8.0864 | 1.08E-13 | 4.59E-12 | 20.5971 |
| EMP1 | 1.63566 | 5.0162883 | 8.29856 | 3.05E-14 | 1.44E-12 | 21.837 |
| IGKC | 1.63986 | 3.1039646 | 4.34622 | 2.37E-05 | 0.0001111 | 1.96722 |
| COL3A1 | 1.64338 | 3.1014326 | 5.57821 | 9.35E-08 | 7.98E-07 | 7.2792 |
| IGHG1 | 1.64672 | 2.4733541 | 4.41002 | 1.82E-05 | 8.79E-05 | 2.21738 |
| CYR61 | 1.64916 | 4.7155813 | 7.59783 | 1.89E-12 | 5.74E-11 | 17.7973 |
| PLAU | 1.64925 | 2.6317629 | 7.82605 | 5.02E-13 | 1.77E-11 | 19.095 |
| PLEKHA4 | 1.64965 | 3.7634224 | 9.92862 | 1.25E-18 | 1.62E-16 | 31.7469 |
| TYMP | 1.67705 | 3.8130065 | 8.62259 | 4.31E-15 | 2.55E-13 | 23.7561 |
| COL4A1 | 1.68469 | 3.9792357 | 6.06915 | 8.06E-09 | 9.06E-08 | 9.65292 |
| EMP3 | 1.71242 | 3.9527548 | 6.9563 | 7.11E-11 | 1.38E-09 | 14.2547 |
| FCGR3A | 1.73055 | 5.4550603 | 8.48678 | 9.82E-15 | 5.32E-13 | 22.9482 |
| GNG12 | 1.73682 | 3.9430405 | 10.8341 | 3.74E-21 | 9.28E-19 | 37.4604 |
| TIMP1 | 1.79605 | 5.2935639 | 5.94244 | 1.54E-08 | 1.61E-07 | 9.02765 |
| ANXA2 | 1.80182 | 5.9688173 | 8.3567 | 2.15E-14 | 1.06E-12 | 22.1791 |
| CP | 1.81025 | 3.3867495 | 7.29709 | 1.06E-11 | 2.58E-10 | 16.1164 |
| GBP2 | 1.83212 | 4.1351999 | 8.14646 | 7.57E-14 | 3.32E-12 | 20.9466 |
| TFPI | 1.89227 | 2.8680792 | 9.88133 | 1.69E-18 | 2.13E-16 | 31.4517 |
| VCAM1 | 1.92442 | 4.0432903 | 8.87902 | 8.96E-16 | 6.28E-14 | 25.295 |
| SERPINA3 | 1.94782 | 6.5594448 | 5.5048 | 1.33E-07 | 1.09E-06 | 6.93594 |
| NNMT | 1.95463 | 2.9720844 | 7.16133 | 2.27E-11 | 5.09E-10 | 15.369 |
| TNFRSF12A | 2.00087 | 3.6140444 | 9.24307 | 9.41E-17 | 7.94E-15 | 27.507 |
| IGFBP2 | 2.01955 | 5.0947798 | 7.52633 | 2.86E-12 | 8.27E-11 | 17.3946 |
| FCGBP | 2.02633 | 2.7502947 | 9.27948 | 7.50E-17 | 6.63E-15 | 27.7299 |
| CRNDE | 2.0404 | 2.4009816 | 9.73303 | 4.33E-18 | 5.09E-16 | 30.5284 |
| PDPN | 2.04301 | 3.6800375 | 8.17976 | 6.21E-14 | 2.76E-12 | 21.141 |
| SPOCD1 | 2.22283 | 2.7121417 | 7.9096 | 3.07E-13 | 1.14E-11 | 19.5746 |
| VIM | 2.26773 | 9.1429172 | 11.0585 | 8.72E-22 | 2.59E-19 | 38.8917 |
| LTF | 2.29002 | 2.2567842 | 6.75908 | 2.10E-10 | 3.54E-09 | 13.1999 |
| ANXA1 | 2.36403 | 5.2101581 | 9.31472 | 6.02E-17 | 5.42E-15 | 27.9459 |
| CHI3L2 | 2.61605 | 4.5610928 | 7.79401 | 6.05E-13 | 2.07E-11 | 18.9118 |
| CHI3L1 | 2.90201 | 5.3078124 | 6.94508 | 7.57E-11 | 1.46E-09 | 14.1943 |
