# Supplementary material for: MAGOH is correlated with poor prognosis and is essential for cell proliferation in lower-grade glioma
Source: Aging (Albany NY). 2023 Jun 30;15(12):5713–33. doi: 10.18632/aging.204823 (PMC10333088; doi:10.18632/aging.204823)
Supplement: Supplementary Tables 5 and 6 [file aging-15-204823-s006.pdf]

SUPPLEMENTARY TABLES

Supplementary Table 5. Clinical features of LGG patients from TCGA.

Clinical features		Total (477)	%
Age	Age ≤45	287	60.17%
	Age >45	190	39.83 %
Gender	Female	216	45.28%
	Male	261	54.72%
Grade	WHO II	231	48.43%
	WHO III	246	51.57%
1p/19q	Non-codel	321	67.30%
	Codel	156	32.70%
IDH	Mutant	389	81.55%
	Wildtype	85	17.82%
	Unknow	3	0.63%
MGMT	Unmethylated	82	17.19%
	Methylated	395	82.81%

Supplementary Table 6. Clinical features of LGG patients from CGGA.

Clinical features		Total (170)	%
Age	Age ≤45	129	75.88%
	Age >45	41	24.12%
Gender	Female	65	38.24%
	Male	105	61.76%
Grade	WHO II	97	57.06%
	WHO III	73	42.94%
1p/19q	Non-codel	113	66.47%
	Codel	55	32.35%
	Unknow	2	1.18%
IDH	Mutant	125	73.53%
	Wildtype	44	25.88%
	Unknow	1	0.59%
MGMT	Unmethylated	70	41.18%
	Methylated	84	49.41%
	Unknow	16	9.41%
